# Detection of gene fusions using targeted next-generation sequencing: a comparative evaluation

**DOI:** 10.1186/s12920-021-00909-y

**Published:** 2021-02-27

**Authors:** Carina Heydt, Christina B. Wölwer, Oscar Velazquez Camacho, Svenja Wagener-Ryczek, Roberto Pappesch, Janna Siemanowski, Jan Rehker, Florian Haller, Abbas Agaimy, Karl Worm, Thomas Herold, Nicole Pfarr, Wilko Weichert, Thomas Kirchner, Andreas Jung, Jörg Kumbrink, Wolfgang Goering, Irene Esposito, Reinhard Buettner, Axel M. Hillmer, Sabine Merkelbach-Bruse

**Affiliations:** 1 Institute of Pathology, University Hospital Cologne, Kerpener Str. 62, 50937 Cologne, Germany; 2 Institute of Pathology, University Hospital Erlangen, Erlangen, Germany; 3 Institute of Pathology, University Hospital Essen, University Duisburg-Essen, Essen, Germany; 4 Institute of Pathology, Technical University Munich (TUM), Munich, Germany; 5 Institute of Pathology, LMU Munich, Munich, Germany; 6 Institute of Pathology, Medical Faculty, Heinrich-Heine-University and University Hospital Duesseldorf, Düesseldorf, Germany

**Keywords:** NGS, FISH, Gene fusion, DNA-Seq, RNA-seq

## Abstract

**Background:**

Gene fusions represent promising targets for cancer therapy in lung cancer. Reliable detection of multiple gene fusions is therefore essential.

**Methods:**

Five commercially available parallel sequencing assays were evaluated for their ability to detect gene fusions in eight cell lines and 18 FFPE tissue samples carrying a variety of known gene fusions. Four RNA-based assays and one DNA-based assay were compared; two were hybrid capture-based, TruSight Tumor 170 Assay (Illumina) and SureSelect XT HS Custom Panel (Agilent), and three were amplicon-based, Archer FusionPlex Lung Panel (ArcherDX), QIAseq RNAscan Custom Panel (Qiagen) and Oncomine Focus Assay (Thermo Fisher Scientific).

**Results:**

The Illumina assay detected all tested fusions and showed the smallest number of false positive results. Both, the ArcherDX and Qiagen panels missed only one fusion event. Among the RNA-based assays, the Qiagen panel had the highest number of false positive events. The Oncomine Focus Assay (Thermo Fisher Scientific) was the least adequate assay for our purposes, seven fusions were not covered by the assay and two fusions were classified as uncertain. The DNA-based SureSelect XT HS Custom Panel (Agilent) missed three fusions and nine fusions were only called by one software version. Additionally, many false positive fusions were observed.

**Conclusions:**

In summary, especially RNA-based parallel sequencing approaches are potent tools for reliable detection of targetable gene fusions in clinical diagnostics.

## Background

Chromosomal rearrangements leading to the fusion of coding regions of two genes can result in expression of oncogenic hybrid proteins driving tumor progression. The first fusion gene, *BCR*-*ABL* was discovered in chronic myeloid leukemia patients [1]. Subsequently, many fusion genes were identified in a variety of cancer types since, including lung cancer and many other solid tumors. Non-small cell lung cancer (NSCLC) accounts for approximately 85% of all lung cancers. Lung adenocarcinoma (ADC) and lung squamous cell carcinoma (SqCC) are the two most commonly occurring NSCLC subgroups [2, 3]. Unfortunately, around 75% of patients with lung cancer present with non-resectable, advanced stages of disease and thus most patients are not eligible for curative therapy [4]. A characteristic feature of NSCLCs is the addiction to single oncogenic driver events, which render these cancer types vulnerable to drugs targeting corresponding kinases [5, 6]. Indeed, despite numerous resistance mechanisms associated with targeted therapies, treatment with EGFR (epidermal growth factor receptor) or ALK (ALK receptor tyrosine kinase) tyrosine kinase inhibitors has been shown to significantly improve progression-free and overall survival compared to standard chemotherapy in patients with advanced lung cancer harboring activating *EGFR* mutations [7, 8] or *ALK* rearrangements [9–11].

*ALK* rearrangements occur in 3–8% of NSCLC cases, are generally mutually exclusive with *EGFR* or *KRAS* mutations and commonly found in adenocarcinomas of younger patients with light or no smoking history [12–14]. Twenty *ALK* fusion partners have been described to date, including kinesin family member 5B (*KIF5B*), trafficking from ER to golgi regulator (*TFG*) and kinesin light chain 1 (*KLC1*) [15]. Other, recurrent and therapeutically targetable fusion genes involving ROS proto-oncogene 1, receptor tyrosine kinase (*ROS1*) [16, 17], ret proto-oncogene (*RET*) [18, 19], B-Raf proto-oncogene, serine/threonine kinase (*BRAF*) [20], fibroblast growth factor receptor 1–3 (*FGFR1-3*) [21, 22], neurotrophic receptor tyrosine kinase 1–3 (*NTRK1-3*) [23], *EGFR* [24, 25] and MET proto-oncogene, receptor tyrosine kinase (*MET*) [26, 27] have also been identified in NSCLC patients. Importantly, tumors carrying these rearrangements represent unique molecular cohorts of NSCLC as they are eligible for targeted tyrosine kinase inhibitor therapies. Despite recent advances in our understanding of the genetic subgroups of NSCLC, there is still a relatively large subset of “pan-negative” patients, who do not carry any known oncogenic driver variations, suggesting that other driver alterations, including gene fusions or epigenetic alterations are yet to be identified.

Fluorescence in situ hybridization (FISH) and immunohistochemistry (IHC) still represent standard technologies for detecting chromosomal aberrations in routine clinical practice. However, in order to facilitate genetically guided treatment decision-making, and to utilize small biopsies more efficiently, it is mandatory to simultaneously investigate the mutational status of multiple genes in one single assay. Thus, parallel sequencing methods are increasingly used as a tool for new and recurrent gene fusion discovery and a range of commercially available assays are being established in clinical diagnostics laboratories today. Therefore, we here tested and compared five different commercially available assays for their ability to reliably detect gene fusions in cell lines and formalin-fixed and paraffin-embedded (FFPE) tumor tissue, covering RNA- and DNA-based, as well as hybrid capture- and amplicon-based enrichment approaches for targeted parallel sequencing.

## Methods

### Tumor cohort

A collection of 18 FFPE patient tumor tissue samples, previously tested positive for *ALK*, *ROS1*, *RET*, *FGFR2*, *NTRK1/3*, *MET* or *BRAF* rearrangements by fluorescence in situ hybridization (FISH), immunohistochemistry (IHC) or targeted RNA-based parallel sequencing (Archer FusionPlex panel (ArcherDX, Boulder, CO, USA)) during clinical diagnostics was selected. FFPE tissue samples were obtained as part of routine clinical care under approved ethical protocols complied with the Ethics Committee of the Medical Faculty of the University of Cologne, Germany and informed consent was given from each patient. In addition, eight different cell lines harboring known gene fusions were analyzed. For this, two cell lines were combined at five different ratios (90:10, 80:20, 50:50, 20:80, 10:90) respectively resulting in 40 cell line mixtures. SJ-GBM2 cells (harboring a *CLIP*-*MET* fusion) were mixed with RT112 cells (harboring a *FGFR3*-*TACC3* fusion), KM-12 cells (harboring a *TPM3*-*NTRK1* fusion) were mixed with H2228 cells (harboring a *EML4*-*ALK* fusion), RT4 cells (harboring a *FGFR3*-*TACC3* fusion) were mixed with HCC-78 cells (harboring a *SLC34A2*-*ROS1* fusion) and SW780 cells (harboring a *FGFR3*-*BAIAP2L1* fusion) were mixed with KG-1 cells (harboring a *FGFR1OP2*-*FGFR1* fusion). H2228, RT4 and SW780 cells were provided kindly by Roman Thomas, Institute of Translational Cancer Research, Center of Integrated Oncology, University Hospital of Cologne, Cologne, Germany. RT112 cells were purchased from Cell Lines Service (Eppelheim, Germany). KM-12 cells were purchased from the DCTD Tumor Repository, National Cancer Institute (Frederick, Maryland, USA). HCC-78 cells were purchased from the Leibniz Institute DSMZ (Braunschweig, Germany). KG-1 (ATCC CCL246) were purchased from ATCC (Manassas, Virgina, USA). SJ-GMB2 were provided by Children’s Oncology Group Cell Culture Repository. All cells were cultured according to the providers’ instructions. Cell pools were fixed in 4% formalin overnight and subsequently pelleted and stored at − 20 °C until further use.

### DNA, total nucleic acid (tNA) and RNA extraction

For DNA, total nucleic acid (tNA) and RNA extractions from FFPE samples, tumor areas were marked on hematoxylin–eosin (H&E) stained slides by a pathologist. The corresponding area was macrodissected from two to three 10 µm sections. DNA was extracted using the Maxwell 16 FFPE Plus Tissue LEV DNA Purification Kit (Promega, Mannheim, Germany). DNA from cell lines was extracted using the Maxwell RSC Blood DNA Kit (Promega). Extracted DNA was quantified with the Qubit dsDNA HS Assay Kit (Thermo Fisher Scientific, Waltham, MA, USA). tNA from both FFPE sections and cell lines was extracted using the Maxwell RSC RNA FFPE Purification Kit (Promega). For DNA-free RNA, the genomic DNA was removed from the tNA extracts using the TURBO DNA-free Kit (Thermo Fisher Scientific). tNA and RNA were quantified using the Qubit RNA HS Assay Kit (Thermo Fisher). DNA and RNA integrity was analyzed using the 4200 TapeStation System (Agilent Technologies, Santa Clara, CA, USA).

### Archer FusionPlex Lung Panel (ArcherDX)

For the detection of known and novel gene fusions in 13 genes including *ALK*, *ROS1*, *RET*, *BRAF*, *MET*, *FGFR1-3*, *NTRK1-3* the Archer FusionPlex Lung Panel (ArcherDX) was used according to manufacturer’s instructions. For this assay, 200 ng tNA input per sample was used for the preparation of each library. Purified libraries were quantified using the KAPA Library Quantification Kit (Roche, Pleasanton, CA, USA) and pooled to equimolar concentrations. Sequencing was performed on the MiSeq System (Illumina, San Diego, CA, USA) and results were analyzed using the Archer Suite Analysis v5.0.4 as well as the Archer Suite Analysis v5.1.3 software (ArcherDX). A fusion was classified as strong with among others the following setting: Minimal reads for valid structural variation > 5; Structural variation percent of GSP2 reads > 10. The time for analysis heavily relied on the used hardware. On our local hardware one sample took 2–3 h.

### QIAseq RNAscan Custom Panel (Qiagen)

A QIAseq RNAscan Custom Panel was designed targeting known fusion breakpoints and novel fusions in *ALK*, *ROS1*, *RET*, *BRAF*, *MET*, *FGFR1-3*, *NTRK1-3* (Additional file 1: Table S1). For each sample, 200 ng of DNase digested RNA was used for cDNA synthesis by reverse transcription. For library preparation the QIAseq RNAscan Custom Panel (Qiagen) was used following manufacturer’s instructions. Purified libraries were quantified using the Qubit dsDNA Assay Kit (Thermo Fisher Scientific) and library quality was assessed using the Fragment Analyzer (Agilent). Libraries were pooled to equimolar concentration and sequenced using a NextSeq 500 (Illumina). Results were analyzed using the CLC Genomics Workbench v12.0 (Qiagen). A fusion was classified as “PASS” with a p-value below 0.005 and a minimum number of 2 fusion-supporting reads. The time for analysis heavily relied on the used hardware. On our local hardware one sample took around 15 h.

### Oncomine Focus Assay (Thermo Fisher Scientific)

The Oncomine Focus Assay (Thermo Fisher Scientific) was used for the detection of gene fusions in 23 genes including *ALK*, *ROS1*, *RET*, *BRAF*, *MET*, *FGFR1-3*, *NTRK1-3*. 10–50 ng of DNase digested RNA was used for library preparation according to manufacturer’s instructions. Final libraries were diluted, pooled and further processed on Ion spheres using Ion 520 or 318 Chef Kits (Thermo Fisher Scientific) on the Ion Chef (Thermo Fisher Scientific) or the Ion 520 & Ion 530 Kit-OT2 (Thermo Fisher Scientific). Sequencing was performed on Ion S5 System or PGM systems (Thermo Fisher Scientific) using the Ion 520 or 318 Chips (Thermo Fisher Scientific). Sequencing data were analyzed with the Ion Reporter Software v5.10 (Thermo Fisher Scientific). A fusion was classified as present with greater than 20 reads providing evidence for the fusion and/or an 3′/5′ imbalance value thresholds for strong evidence of a fusion: ALK: ≥ 0.0015, RET: ≥ 0.55, ROS1: ≥ 2.1. The 3′/5′ imbalance score calculates the differences in expression between the 5′ assay and the 3′ assay of each gene. In the case of a fusion event, the 3′ portion of the gene is overexpressed in comparison to the 5′ assay. Resulting in a 3′/5′ imbalance score greater than the baseline.

The time for analysis heavily relied on the used hardware. On the specific server hardware delivered with the system one sample took around 15 min.

### TruSight Tumor 170 Assay (Illumina)

The TruSight Tumor 170 Kit (Illumina) offers a DNA as well as RNA-based workflow. Here, only the RNA-based workflow was applied for the identification of gene fusions in 55 genes including *ALK*, *ROS1*, *RET*, *BRAF*, *MET*, *FGFR1-3*, *NTRK1-3*. 85 ng of DNase digested RNA was used for library preparation of each sample. For library preparation and enrichment the TruSight Tumor 170 Kit (Illumina) was used following manufacturer’s instructions. Post-enriched libraries were quantified using the Qubit dsDNA HS Assay Kit (Thermo Fisher Scientific). After bead-based library normalization, sequencing was performed on a NextSeq 500 (Illumina). Sequencing data were analyzed with BaseSpace TruSight Tumor 170 App v1.0.3. (Illumina). A fusion was classified as “PASS” with among others the fusion supporting reads greater than 5. The time for analysis heavily relied on the used hardware. On the cloud-based BaseSpace TruSight Tumor 170 App one sample took 30 min–1.5 h.

### SureSelect XT HS Custom Panel (Agilent)

Custom capture probes were designed using SureDesign (Agilent) targeting known fusion breakpoints and novel fusions in *ALK*, *ROS1*, *RET*, *BRAF*, *MET*, *FGFR1-3*, *NTRK1-3* (Additional file 2: Table S2). 200 ng of DNA was sheared on the Covaris E220 Focused-ultrasonicator (Covaris, Woburn, MA, USA) to a fragment size of 150 bp using the 8 microTUBE–50 Strip AFA Fiber V2 following manufacturer’s instructions. The treatment time was optimized for FFPE material. The treatment settings were the following: Peak Incident Power (W): 175; Duty Factor: 10%; Cycles per Burst: 200; Treatment Time (s): 200; Temperature (°C): 7; Water Level: 6.

For library preparation the SureSelect XT HS Reagent Kit (Agilent) was used according to manufacturer’s instructions. In brief, pre-enriched adapter-ligated libraries were prepared. Subsequently, custom capture probes were hybridized to target sequences to allow for sequence enrichment using streptavidin beads. Post-enriched libraries were quantified using the Qubit dsDNA Assay Kit (Thermo Fisher Scientific) and library quality was assessed using the Fragment Analyzer (Agilent). Libraries were pooled to equimolar concentrations and sequenced on a NextSeq 500 (Illumina). Sequencing data were analyzed with the SureCall Software v4.0.1.46 and v4.1.1.5 (Agilent). The correlation coefficient (R) was calculated with the following formula: Σ [(X − X_m_) * (Y − Y_m_)] / √ [Σ (X − X_m_)^2^ * Σ (Y − Y_m_)^2^]. The coefficient of determination (R^2^) is the squre of the correlation coefficient. A fusion was classified as present with among others the following setting: Minimum number of reads per translocation > 5. The time for analysis heavily relied on the used hardware. On our local hardware one sample took around 1.5–2 h.

## Results

### Study design

**Fig. 1:**
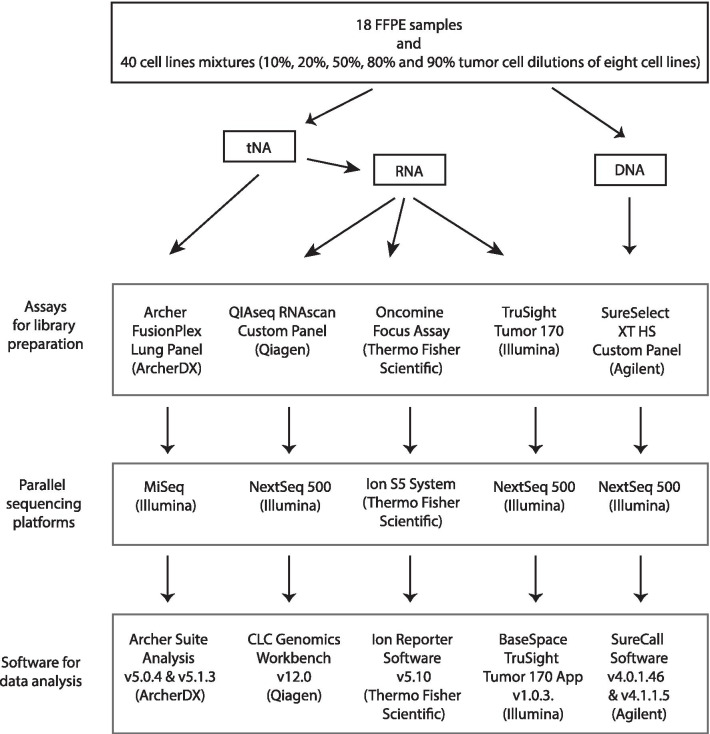
Workflow of the study. DNA, tNA and RNA were extracted from 18 FFPE samples and 40 cell line mixtures (10%, 20%, 50%, 80% and 90% tumor cell dilutions of eight cell lines). All samples were processed with five different assays for library preparation and software for data analysis. Sequencing was performed on three different platforms

In this study, five different commercially available assays were analyzed for their ability to reliably detect gene fusions in cell lines as well as diagnostic FFPE patient tumor tissue samples, covering RNA- and DNA-based, as well as hybrid capture- and amplicon-based enrichment approaches for targeted parallel sequencing (Fig. 1). 18 FFPE samples with known fusions in *ALK*, *ROS1*, *RET*, *FGFR1/3*, *NTRK1/3*, *MET* or *BRAF* were selected. Additionally, eight cell lines harboring known gene fusions (*EML4*-*ALK*, *FGFR3*-*TACC3*, *CLIP*2-*MET*, *TPM3*-*NTRK1*, *SLC34A2*-*ROS1*, *FGFR3*-*BAIAP2L1* and *FGFR1OP2*-*FGFR1*) were formalin fixed. For analysis, two cell lines were combined at five different ratios (90:10, 80:20, 50:50, 20:80, 10:90) in order to investigate the sensitivity of the different assays. For all five assays, libraries were prepared from the same extracted tNA, RNA or DNA. Technical parameters of all five assays were listed in Table 1.

**Table 1:** Comparison of technical parameters

Assay	Input material	Input amount (ng)	No. of samples/run	Sequencer	Flow cell	Costs library preparation/sample	Duration of library preparation (days)	Duration of sequencing run (h)
Archer FusionPlex Lung Panel (Archer DX)	tNA	200	16	MiSeq (Illumina)	MiSeq Reagent Kit v2 (300 cycles)	200 €	2	26
QIAseq Targeted RNAscan Custom Panel (Qiagen)	RNA	200	23	NextSeq 500 (Illumina)	NextSeq 500/550 High Output Kit v2.1 (300 cycles)	190 €	2	29
Oncomine Focus Assay (Thermo Fisher Scientific)	RNA	10–50	20–40	Ion S5 System or Ion PGM System (Thermo Fisher Scientic)	Ion 520 or Ion 316 Chip	130–150 €	1.5	3
TruSight Tumor 170 Assay (Illumina)	RNA	85	20	NextSeq 500 (Illumina)	NextSeq 500/550 Output Kit v2.1 (300 cycles)	160 €	2	24
SureSelect XT HS Custom Panel (Agilent)	DNA	200	23	NextSeq 500 (Illumina)	NextSeq 500/550 High Output Kit v2.1 (300 cycles)	320 €	2	24

*tNA* total nucleic acid

### Cell lines

**Fig. 2:**
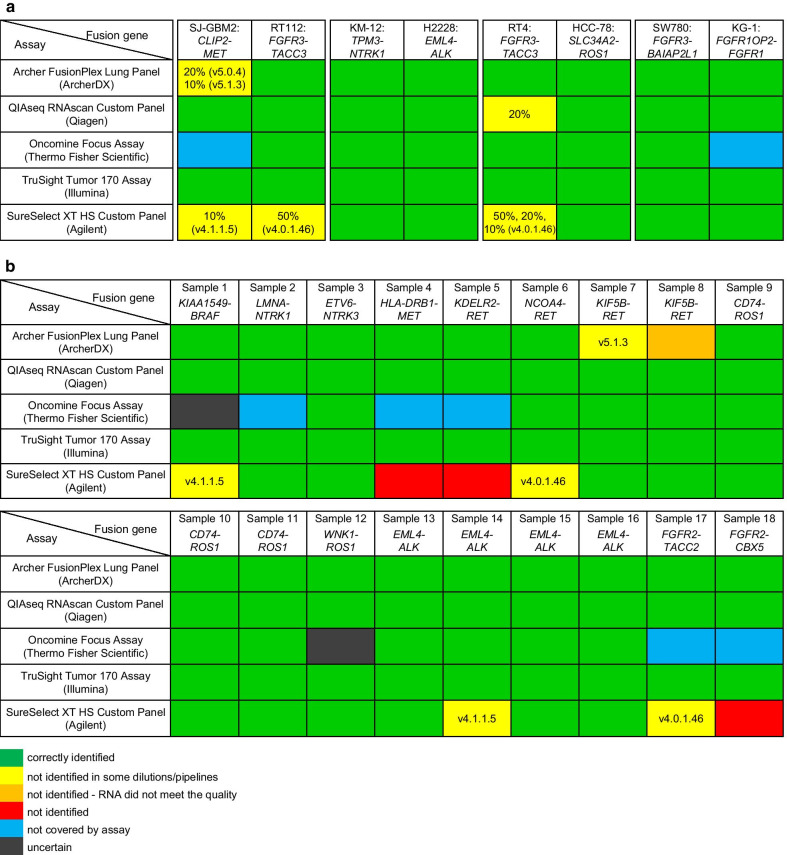
Summary of sequencing results for all five assays and their respective data analysis tools. Results of the 10%, 20%, 50%, 80% and 90% tumor cell dilutions of the eight cell lines (**a**) as well as the FFPE samples (**b**) are shown. Fusions correctly identified in all dilutions are highlighted in green. Fusions not identified in some dilutions and software version are indicated and are highlighted in yellow. Fusion not covered by the assay are highlighted in blue. Fusions classified as uncertain are highlighted in grey. Fusions not identified are colored in red and samples where the fusion was not detected due to RNA quality are highlighted in orange

The results for the cell lines are summarized in Fig. 2a and Additional file 3: Fig. S3, Additional file 4: Fig. S4, Additional file 5: Fig. S5, Additional file 6: Fig. S6, Additional file 7: Fig. S7, Additional file 8: Fig. S8, Additional file 9: Fig. S9, Additional file 10: Fig. S10, Additional file 11: Fig. S11, Additional file 12: Fig. S12, Additional file 13: Fig. S13, Additional file 14: Fig. S14, Additional file 15: Fig. S15, Additional file 16: S16. First, SJ-GBM2 cells harboring a *CLIP*-*MET* fusion were mixed with RT112 cells harboring a *FGFR3*-*TACC3* fusion. With the Archer FusionPlex Lung Panel (ArcherDX), the *CLIP*2-*MET* fusion was not detected at two concentrations (20% and 10%) by one Archer Suite Analysis version each (ArcherDX) (Fig. 2a). In order to identify the *CLIP*2-*MET* fusion at the different concentrations, both software versions had to be utilized. The fusion was also not present in the low confidence fusions. All dilutions of the *FGFR3*-*TACC3* fusion were detected by both software versions. With the SureSelect XT HS Custom Panel (Agilent) the *CLIP*2-*MET* fusion (Fig. 2a) was not detected when diluted at 10% by the SureCall v4.1.1.5 but detected by SureCall v4.0.1.46 (Agilent) (Fig. 2a). On the other hand, the 50% diluted *FGFR3*-*TACC3* fusion, expressed by the RT112 cell line was identified by SureCall v4.1.1.5 but not by v4.0.1.46 (Agilent). Thus, similar to the Archer FusionPlex Lung Panel (ArcherDX), both software versions had to be utilized to detect both fusion genes (*CLIP*2-*MET* and *FGFR3*-*TACC3*) at the different ratios (Fig. 2a). Expectedly, The Oncomine Focus Assay (Thermo Fisher Scientific) did not detect the *CLIP*2-*MET* fusion at any of the dilutions (Fig. 2a), as this fusion is not covered by the panel. Even the larger Oncomine Comprehensive Assay (Thermo Fisher Scienitifc), which covers additional fusion genes, does not cover the *CLIP*2-*MET* fusion either (personal correspondence with Thermo Fisher). Thus, this fusion would be missed by both, the Oncomine Focus as well as Oncomine Comprehensive Assay (Thermo Fisher Scientific). Both, the QIAseq RNAscan Custom Panel (Qiagen) and the TruSight Tumor 170 Assay (Illumina) detected all tested dilutions of the *CLIP-MET* and *FGFR3-TACC3* fusions.

Second, KM-12 cells harboring a *TPM3*-*NTRK1* fusion were mixed with H2228 cells harboring an *EML4*-*ALK* fusion. Both fusions were detected by all five assay and at all five dilutions.

Third, RT4 cells harboring a *FGFR3*-*TACC3* fusion were mixed with HCC-78 cells harboring a *SLC34A2*-*ROS1* fusion. The *FGFR3*-*TACC3* fusion was not detected at the 10% dilution by the QIAseq RNAscan Custom Panel (Qiagen) with the CLC Genomics Workbench v12.0 (Fig. 2a), nor by the SureSelect XT HS Custom Panel (Agilent) and the older SureCall v4.0.1.46 (Agilent) at the lower dilutions (10%, 20% and 50%). With the Archer FusionPlex Lung Panel (ArcherDX), the Oncomine Focus Assay (Thermo Fisher Scientific) and the TruSight Tumor 170 Assay (Illumina) all dilutions of the *FGFR3*-*TACC3* fusion were detected. The *SLC34A2*-*ROS1* fusion was also detected by all assays and at all dilutions.

Fourth, SW780 cells harboring a *FGFR3*-*BAIAP2L1* fusion were mixed with KG-1 cells harboring a *FGFR1OP2*-*FGFR1* fusion. Both fusions were detected by all five assays at all five dilutions except for the *FGFR1OP2*-*FGFR1*, which is not included in the Oncomine Focus Assay (Thermo Fisher Scientific). This fusion is included in the larger Oncomine Comprehensive Assay (Thermo Fisher Scientific) and might have been detected, however, the assay was not tested in this study.

Summarizing the results above, a fraction below or up to 50% of tumor cells can be difficult to detect for certain combinations of panels and evaluation pipelines. This is especially true for the DNA-based SureSelect XT HS Custom Panel (Agilent). When examining the relation of an increasing number of tumor cells and the number of supporting fusion-supporting reads divided by the total read depth a good linear regression with a coefficient of determination (R^2^) of > 0.92 can be observed with the DNA-based custom panel (Additional file 17: Fig. S1) in seven of the eight cell lines. Both SureCall (Agilent) software versions indicate that 50% of tumor cells were needed to receive 5% fusion-supporting reads. This is different to the RNA-based panels, which show no clear association between the number of tumor cells and the supporting fusion-supporting reads. In the RNA-based panels the percentage of fusion-supporting reads is relatively constant, thus for the RNA-based panels it seemed that 10% of tumor cells were enough to generate a sufficient number of fusion-supporting reads.

### FFPE tissue samples

The results of the 18 FFPE tissue samples tested are shown in Fig. 2b and Additional file 18: Fig. S2, Additional file 10: Fig. S10, Additional file 11: Fig. S11, Additional file 12: Fig. S12, Additional file 13: Fig. S13, Additional file 14: Fig. S14, Additional file 15: Fig. S15, Additional file 16: S16. The QIAseq RNAscan Custom Panel (Qiagen) as well as the TruSight Tumor 170 Assay (Illumina) identified all tested gene fusions correctly.

With the Archer FusionPlex Lung Panel (ArcherDX) the *KIF5B-RET* fusion in sample 7 was only detected with the Archer Suite Analysis version 5.0.4 (ArcherDX) and not detected by the newer Archer Suite Analysis version 5.1.3 (ArcherDX) (Fig. 2b). The fusion was also not present in the low confidence fusions. The *KIF5B*-*RET* fusion in sample 8 was not identified at all with this assay. Although the RNA quantity was high in this sample, the RNA quality did not meet the recommended quality standards of the Archer FusionPlex Lung Panel (ArcherDX). Thus, the number of unique RNA GSP2 control start sides was low and the fusion could not be detected.

The Oncomine Focus Assay (Thermo Fisher Scientific) does not cover five of the 18 gene fusions and therefore the following samples tested negative: Sample 2 (*LMNA*-*NTRK1*), sample 4 (*HLA-DRB1*-*MET*), sample 5 (*KDELR2-RET*), sample 17 (*FGFR2*-*TACC2*) and sample 18 (*FGFR2*-CBX5) (Fig. 2b). These *MET*, *RET* and *FGFR2* fusions would also not have been covered by the larger Oncomine Comprehensive Assay (Thermo Fisher Scientific). Thus, these fusions would be missed. Additionally, to these five fusions, two further fusions were classified as uncertain. In sample 1 the number of reads for the *KIAA1549*-*BRAF* fusion was at the set threshold of 20 reads, and thus the fusion was classified as uncertain. The *WNK1*-*ROS1* fusion in sample 12 is not part of the Oncomine Focus Assay (Thermo Fisher Scienitific). However, this fusion could have been detected by the 3′/5′ imbalance score for *ROS1*. In this sample the 3′/5′ imbalance score (0.47) was close but below the threshold (0.5) and thus the fusion was classified as uncertain. Additionally, the *EML4*-*ALK* fusion in sample 16 was only detected by the 3′/5′ imbalance score for *ALK* and not by a specific fusion call, although it was the exact same fusion as in sample 15.

The SureSelect XT HS Custom Panel (Agilent) was the only DNA-based panel included into our study. With the custom panel three fusions were not detected by both SureCall versions, sample 4 (*HLA-DRB1-MET*), sample 5 (*KDELR2*-*RET*) and sample 18 (*FGFR2-CBX5*). Four more fusions in sample 1, 6, 14 and 17 were detected by one SureCall version only. Thus, both versions had to be used to detect these four fusions in *BRAF*, *RET*, *ALK* and *FGFR2* (Fig. 2b).

**Fig. 3:**
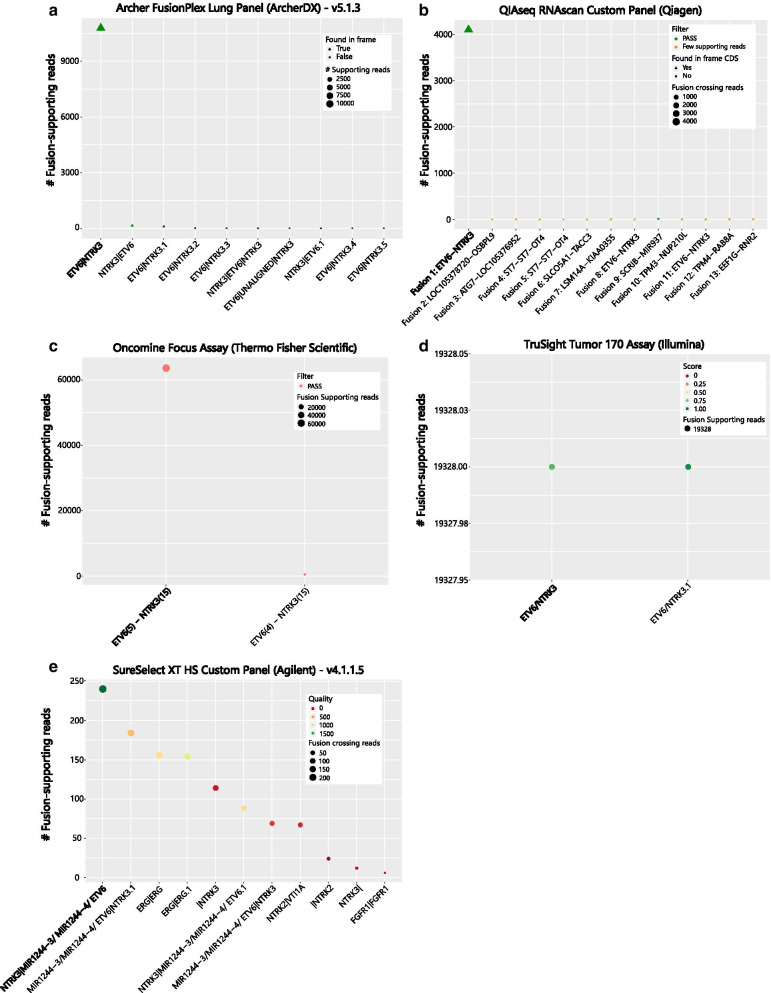
Representative examples of the *ETV6-NTRK3* fusion in sample 3 detected by all five assays and their respective data analysis tools. Metrics such as quality control scores, in-frame status or filter thresholds were plotted when available. In cases where the same fusion was identified more than once within the same sample, a unique numbering scheme was added at the end of the name to differentiate the candidate fusions. For all the kits, the putative detected fusions were arranged in decreasing order based on the number of fusion-supporting reads, with the exception of QIAseq RNAscan, where the software already provides the results with an ordering scheme with the most likely fusion being called “Fusion 1”. The expected fusion for each sample was highlighted in bold. (**a**) Archer FusionPlex Lung Panel (Archer DX), (**b**) QIAseq Targeted RNAscan Custom Panel (Qiagen), (**c**) Oncomine Focus Assay (Thermo Fisher Scientific), (**d**) TruSight Tumor 170 Assay (Illumina) and (**e**) SureSelect XT HS Custom Panel (Agilent)

Analyzing the overall number of fusion events detected in one sample by each software, it became apparent that with the TruSight Tumor 170 Assay (Illumina), the Archer FusionPlex Lung Assay (ArcherDX) and the Oncomine Focus Assay (Thermo Fisher Scientific) the results of the detected fusions were very specific and almost only the expected fusions were called. Inferior results were achieved with both custom panels, the QIAseq RNAscan Custom Panel (Qiagen) and the SureSelect XT HS Custom Panel (Agilent) in comparison with the commercially available pre-designed assays. With both assays, false positive fusion events were called. Additionally, the expected fusion did not necessarily show the highest number of fusion-supporting reads. This was especially true for the DNA-based SureSelect XT HS Custom Panel (Agilent), which makes it difficult to select a defined cut-off and report the correct fusion in an unknown sample (Fig. 3 and Additional file 18: Fig. S2, Additional file 3: Fig. S3, Additional file 4: Fig. S4, Additional file 5: Fig. S5, Additional file 6: Fig. S6, Additional file 7: Fig. S7, Additional file 8: Fig. S8, Additional file 9: Fig. S9, Additional file 10: Fig. S10, Additional file 11: Fig. S11, Additional file 12: Fig. S12, Additional file 13: Fig. S13, Additional file 14: Fig. S14, Additional file 15: Fig. S15, Additional file 16: S16). The SureSelect XT HS Custom Panel (Agilent) was also the only panel not indicating which gene was the 5′ and which gene was the 3′ fusion partner gene.

In summary, only the TruSight Tumor 170 Assay (Illumina) detected all fusions analyzed in both cell line and FFPE tissue samples. Further, this assays showed the smallest number of false positive results and used only up to 85 ng of RNA. In our study, the Archer FusionPlex Lung Assay (ArcherDX) had the highest RNA quality requirements or failed otherwise. Among the RNA-based assays the QIAseq RNAscan Custom Panel (Qiagen) had the highest number of false positive fusion events. Although only 10 ng of RNA were needed, the Oncomine Focus Assay (Thermo Fisher Scientific) was the least adequate assay for our purposes, as seven targetable lung cancer fusion events were not covered by the assay and two fusions were classified as uncertain. Also the DNA-based SureSelect XT HS Custom Panel (Agilent) did not detect three fusions and in nine samples the fusion was only detected in one of the two software version. Further, many false positive fusions were detected, which makes the detection difficult in an unknown sample.

## Discussion

Gene fusions represent promising targets for cancer therapy especially in NSCLC. Many novel compounds inhibiting rearranged, oncogenic proteins have been developed and inhibitors targeting rearranged *ALK*, *ROS1* and *NTRK1-3* received already regulatory approval for clinical use in the United States and the European Union. Thus, a reliable detection of these fusions is essential in patient treatment. To facilitate genetically guided treatment decisions by utilizing small biopsies even more efficiently, the simultaneous investigation of multiple new and recurrent gene fusions becomes more and more important.

Currently, immunohistochemistry and in situ hybridization is still widely used for testing chromosomal rearrangements in FFPE tissue. However, these methods are hampered by their lack of multiplexing opportunity thus requiring one tissue slide per fusion gene assay. Further, small intrachromosomal rearrangements are often missed and the detection of the exact fusion breakpoint and partner is not possible but may become necessary in the future for patient prognosis and prediction of treatment response, which was shown in previous studies [28–30]. Thus, a range of commercially available parallel sequencing approaches have been developed for use in routine diagnostics to cover multiple fusions with one assay.

In this study we analyzed, five different commercially available assays for their ability to reliably detect a variety of known NSCLC specific gene fusions in eight cell lines and 18 FFPE tissue samples, covering RNA and DNA-based as well as hybrid capture- and amplicon-based enrichment approaches for targeted parallel sequencing.

Four RNA-based assays were compared, the hybrid capture-based assay TruSight Tumor 170 Assay (Illumina) and three amplicon-based assays, the Archer FusionPlex Lung Panel (ArcherDX), a QIAseq RNAscan Custom Panel (Qiagen) and the Oncomine Focus Assay (Thermo Fisher Scientific). The Archer FusionPlex Lung Panel (ArcherDX) and the QIAseq RNAscan Custom Panel (Qiagen) use the single primer extension technology where only one gene specific primer is used for the target region, enabling the detection of known and unknown fusion partners [31, 32]. The Oncomine Focus Assay (Thermo Fisher Scientific) utilizes the traditional approach with two opposing primer pairs flanking the target region and fusion partner [33]. 10–200 ng tNA/RNA are recommended for these four assays.

The TruSight Tumor 170 Assay (Illumina) showed the highest reliability and identified all gene fusions in the cell line samples at all dilutions as well as all gene fusions in the FFPE samples. Further, the number of false positive fusion events was very small and only 85 ng RNA had to be used. Second best performed the Archer FusionPlex Lung Panel (ArcherDX) and the QIAseq RNAscan Custom Panel (Qiagen). The Archer FusionPlex Lung Panel (ArcherDX) is susceptible to low quality RNA samples, which has also been reported in previous studies [31, 34, 35]. In our study a *RET* fusion was not detected due to low quality RNA. However, this fusion was identified with all other RNA-based assays. Additionally, two software versions had to be used to find all fusion events reliably, user accessible filter setting for both software versions were the same. As different versions of the software pipelines altered resulting fusion calls it must be emphasized that introduction of a new version of a software pipeline in a clinical setting always requires a thorough re-evaluation of the results. Compared to alterations of sequencing technique or library chemistry however, this can be accomplished with already existing and evaluated sequencing data. Recently, a newer software version v6.2.1 became available and might solve this problem. The number of false positive results was also very low with the Archer FusionPlex Lung Panel (ArcherDX). The QIAseq RNAscan Custom Panel (Qiagen) also only failed in one sample as it did not detect the *FGFR3* gene fusion in the 20% dilution of the RT4 cell line. However, with the QIAseq RNAscan Custom Panel (Qiagen) many false positive fusions were called, which makes the correct diagnosis difficult in an unknown sample. As the Qiagen panel was a custom panel, the panel could be further optimized by removing oligonucleotides in repetitive regions or regions that are not specific to the relevant genes, this might reduce the number of false positive calls. Additionally, some of the false positive calls were repetitive sequences and panel artefacts, which could be filtered out.

The Archer FusionPlex Lung Panel (ArcherDX) used 200 ng of total nucleic acid and the QIAseq RNAscan Custom Panel (Qiagen) used 200 ng of DNase digested RNA, which can be difficult to achieve with small NSCLC biopsies sometimes [31, 33–35].

The Oncomine Focus Assay (Thermo Fisher Scientific) could not identify gene fusions in seven samples including *MET*, *FGFR1*, *NTRK1*, *RET* and *FGFR2* fusions as these fusions were not covered by the assay. Even the larger Oncomine Comprehensive Assay does not include five of these seven fusions. Additionally, two fusions in *BRAF* and *ROS1* were classified as uncertain as they were at the fusion detection threshold, as shown in a recent study [33]. The advantage of the Oncomine Focus Assay (Thermo Fisher Scientific) is that only 10 ng of RNA were needed for library preparation. The Oncomine Focus Assay (Thermo Fisher Scientific) can also be a good approach as a confirmatory and supplemental tool after IHC screening for the detection of gene fusions in already targetable genes like *ALK* and *ROS1* and in parts *NTRK1-3*. Additionally, the identification and confirmation of *MET* Exon 14 skipping events by alternative splicing is possible, as with the other RNA-based assays used in this study. Targeted therapy for this aberration may become approved in the near future [36].

One DNA-based assay was used in our study, a SureSelect XT HS Custom Panel (Agilent). This assay enables the simultaneous detection of somatic gene mutations, gene fusion and copy number alterations with one DNA extract only. However, recently it was shown that DNA-based parallel sequencing approaches can lead to false negative sequencing results in a variety of cases and that additional RNA sequencing is recommended in negative samples [34].

This was in agreement with our study. Using the SureSelect XT HS Custom Panel (Agilent) three of the 18 FFPE tissue samples were false negative, because one *MET,* one *RET* and one *FGFR2* fusion were not found. Here, also two software versions had to be used to find nine of the fusion events, again user accessible filter setting for both software versions were the same. Additionally, many false positive fusions were called by this assay with high fusion-supporting reads, which makes it difficult to select a defined cut-off and report the correct fusion in an unknown sample. In DNA-based panels the fusion break points have to be covered by large intronic regions, often containing repetitive regions, leading to false positive calls and more off-target reads. This also requires a higher coverage than RNA-based sequencing, where only exonic regions are sequenced. The advantage of RNA-based assays in comparison to DNA-based assays is that in case of fusion events the RNA expression is often higher than in a DNA-based assay and thus the assays are more efficient and sensitive, which has also been published recently [34, 37, 38]. Further, the fusion points on the RNA-level are usually at the end and start of exons, respectively, allowing more efficient filtering of technical noise.

In the DNA-based sequencing data, the increasing number of tumor cells and the number of supporting fusion-supporting reads divided by the total read depth showed a good linear correlation in the cell line samples with a coefficient of determination (R^2^) of > 0.92. Both Sure call (Agilent) software versions indicate that at least 50% of tumor cells were needed to receive 5% of fusion-supporting reads. In the RNA-based panels used in this study, the percentage of fusions reads is relatively constant, probably due to high RNA fusion expression. Thus, for the RNA-based panels it seems that 10% of tumor cells is enough to generate a sufficient number of fusion-supporting reads. In another study it was shown that in RNA-based sequencing data the fusion-supporting reads decrease when diluting the RNA from 1:10 to 1:10,000 [38]. In contrast to this, in our study only dilutions down to 1:10 were performed.

The molecular diagnostics of NSCLC samples are hampered by the fact, that most of the samples are small biopsies. In these samples, an additional RNA extraction after the initial DNA extraction is not possible as no material is left or the DNA and RNA amount is very small [34, 37]. Thus, a combined DNA and RNA extraction from the same tissue slides are needed and have been and are currently evaluated [39, 40].

## Conclusions

In the future, the amount of tissue, effort and time required to complete diagnostic tests will become more and more limited. As shown in this study, especially RNA-based parallel sequencing approaches demonstrate the potential to replace the use of in situ hybridization and immunohistochemistry in NSCLC for the reliable detection of the growing diversity of targetable gene fusions in clinical diagnostics.

## Supplementary Information

**Additional file 1: Table S1**. Bed-file of QIAseq Targeted RNAscan Custom Panel (Qiagen).

**Additional file 2: Table S2**. Bed-file of SureSelect XT HS Custom Panel (Agilent).

**Additional file 3: Fig. S3**. Results of Archer FusionPlex Lung Panel (Archer DX) (v5.0.4) for the cell line mixtures. Shown are the number of true positive fusions detected, the number of fusion-supporting reads for this fusion, as well as the number of false positives and missed fusions identified per cell line dilution.

**Additional file 4: Fig. S4**. Results of Archer FusionPlex Lung Panel (Archer DX) (v5.1.3) for the cell line mixtures. Shown are the number of true positive fusions detected, the number of fusion-supporting reads for this fusion, as well as the number of false positives and missed fusions identified per cell line dilution.

**Additional file 5: Fig. S5**. Results of QIAseq Targeted RNAscan Custom Panel (Qiagen) for the cell line mixtures. Shown are the number of true positive fusions detected, the number of fusion-supporting reads for this fusion, as well as the number of false positives and missed fusions identified per cell line dilution.

**Additional file 6: Fig. S6**. Results of Oncomine Focus Assay (Thermo Fisher Scientific) for the cell line mixtures. Shown are the number of true positive fusions detected, the number of fusion-supporting reads for this fusion, as well as the number of false positives and missed fusions identified per cell line dilution.

**Additional file 7: Fig. S7**. Results of TruSight Tumor 170 Assay (Illumina) for all for the cell line mixtures. Shown are the number of true positive fusions detected, the number of fusion-supporting reads for this fusion, as well as the number of false positives and missed fusions identified per cell line dilution.

**Additional file 8: Fig. S8**. Results of SureSelect XT HS Custom Panel (Agilent) (v4.0.1.46) for the cell line mixtures. Shown are the number of true positive fusions detected, the number of fusion-supporting reads for this fusion, as well as the number of false positives and missed fusions identified per cell line dilution.

**Additional file 9: Fig. S9**. Results of SureSelect XT HS Custom Panel (Agilent) (v4.1.1.5) for the cell line mixtures. Shown are the number of true positive fusions detected, the number of fusion-supporting reads for this fusion, as well as the number of false positives and missed fusions identified per cell line dilution.

**Additional file 10: Fig. S10**. Fusions detected with the Archer FusionPlex Lung Panel (Archer DX) (v5.0.4) for all samples. Metrics such as quality control scores, in-frame status or filter thresholds were plotted when available. In cases where the same fusion was identified more than once within the same sample, a unique numbering scheme was added at the end of the name to differentiate the candidate fusions. The numbering however, does not imply any special order or preference over the other fusions with the same name. The putative detected fusions were arranged in decreasing order based on the number of fusion-supporting reads. The expected fusion for each sample was highlighted in bold.

**Additional file 11: Fig. S11**. Fusions detected with the Archer FusionPlex Lung Panel (Archer DX) (v5.1.3) for all samples. Metrics such as quality control scores, in-frame status or filter thresholds were plotted when available. In cases where the same fusion was identified more than once within the same sample, a unique numbering scheme was added at the end of the name to differentiate the candidate fusions. The numbering however, does not imply any special order or preference over the other fusions with the same name. The putative detected fusions were arranged in decreasing order based on the number of fusion-supporting reads. The expected fusion for each sample was highlighted in bold.

**Additional file 12: Fig. S12**. Fusions detected with the QIAseq Targeted RNAscan Custom Panel (Qiagen) for all samples. Metrics such as quality control scores, in-frame status or filter thresholds were plotted when available. In cases where the same fusion was identified more than once within the same sample, a unique numbering scheme was added at the end of the name to differentiate the candidate fusions. The software already provides the results with an ordering scheme with the most likely fusion being “Fusion 1”. The expected fusion for each sample was highlighted in bold.

**Additional file 13: Fig. S13**. Fusions detected with the Oncomine Focus Assay (Thermo Fisher Scientific) for all samples. Metrics such as quality control scores, in-frame status or filter thresholds were plotted when available. In cases where the same fusion was identified more than once within the same sample, a unique numbering scheme was added at the end of the name to differentiate the candidate fusions. The numbering however, does not imply any special order or preference over the other fusions with the same name. The putative detected fusions were arranged in decreasing order based on the number of fusion-supporting reads. The expected fusion for each sample was highlighted in bold.

**Additional file 14: Fig. S14**. Fusions detected with the TruSight Tumor 170 Assay (Illumina) for all samples. Metrics such as quality control scores, in-frame status or filter thresholds were plotted when available. In cases where the same fusion was identified more than once within the same sample, a unique numbering scheme was added at the end of the name to differentiate the candidate fusions. The numbering however, does not imply any special order or preference over the other fusions with the same name. The putative detected fusions were arranged in decreasing order based on the number of fusion-supporting reads. The expected fusion for each sample was highlighted in bold.

**Additional file 15: Fig. S15**. Fusions detected with the SureSelect XT HS Custom Panel (Agilent) (v4.0.1.46) for all samples. Metrics such as quality control scores, in-frame status or filter thresholds were plotted when available. In cases where the same fusion was identified more than once within the same sample, a unique numbering scheme was added at the end of the name to differentiate the candidate fusions. The numbering however, does not imply any special order or preference over the other fusions with the same name. The putative detected fusions were arranged in decreasing order based on the number of fusion-supporting reads. The expected fusion for each sample was highlighted in bold.

**Additional file 16: Fig. S16**. Fusions detected with the SureSelect XT HS Custom Panel (Agilent) (v4.1.1.5) for all samples. Metrics such as quality control scores, in-frame status or filter thresholds were plotted when available. In cases where the same fusion was identified more than once within the same sample, a unique numbering scheme was added at the end of the name to differentiate the candidate fusions. The numbering however, does not imply any special order or preference over the other fusions with the same name. The putative detected fusions were arranged in decreasing order based on the number of fusion-supporting reads. The expected fusion for each sample was highlighted in bold.

**Additional file 17: Fig. S1**. Comparison of cell line dilutions and the number of fusion-supporting reads divided by the total read depth for the SureSelect XT HS Custom Panel (Agilent) v4.0.1.46 (A) and v4.1.1.5 (B). Depicted are the gene fusion-supporting reads divided by the total read depth of the 10%, 20%, 50%, 80% and 90% tumor cell dilutions of the eight cell lines. For each cell line, except the RT4 cells, a regression line and the coefficient of determination (R^2^) is shown. In the RT4 cells in v4.0.1.46 the fusion was only called in the 80% and 90% dilution and in v4.1.1.5 the fusion was only called in one direction by the *TACC3* gene and not the *FGFR3* gene in the 50%, 80% and 90% dilution. Thus, the values are not corresponding to the other values as they are much higher.

**Additional file 18: Fig. S2**. Results of all five assays and their respective data analysis tools for the FFPE samples. Shown are the number of true positive fusions detected, the number of fusion-supporting reads for this fusion, as well as the number of false positives and missed fusions identified per analysis and per FFPE sample.

## Data Availability

The datasets generated and analyzed during the current study are available in the European Genome-phenome Archive (EGA). For data access a data access agreement has to be signed. Once the agreement has been signed the data access committee (DAC) will initiate the data release by EGA. For this please contact Prof. Dr. Axel Hillmer (ahillmer@uni-koeln.de). Study web link: https://www.ebi.ac.uk/ega/studies/EGAS00001004934. Datasets web links: https://www.ebi.ac.uk/ega/datasets/EGAD00001006913, https://ega-archive.org/datasets/EGAD00001006913, https://ega-archive.org/datasets/EGAD00001006913/files.

## Abbreviations

tNA: Total nucleic acid
FFPE: Formalin-fixed paraffin-embedded
FISH: Fluorescence in situ hybridization
IHC: Immunohistochemistry
H&E: Hematoxylin–eosin
