# Supplementary material for: Detection of gene fusions using targeted next-generation sequencing: a comparative evaluation
Source: BMC Med Genomics. 2021 Feb 27;14:62. doi: 10.1186/s12920-021-00909-y (PMC7912891; doi:10.1186/s12920-021-00909-y)
Supplement: Supplementary file 3 — Additional file 3: Fig. S3. Results of Archer FusionPlex Lung Panel (Archer DX) (v5.0.4) for the cell line mixtures. Shown are the number of true positive fusions detected, the number of fusion-supporting reads for this fusion, as well as the number of false positives and missed fusions identified per cell line dilution. [file 12920_2021_909_MOESM3_ESM.pdf]

Archer FusionPlex Lung Panel (Archer DX) v5.0.4)	SJ-GBM2: CLIP2-MET<br>RT112: FGFR3-TACC3	KM-12: TPM3-NTRK1<br>H2228: EML4-ALK	RT4: FGFR3-TACC3<br>HCC-78: SLC34A2-ROS1	SW780: FGFR3-BAIAP2L1<br>KG-1: FGFR1OP2-FGFR1	Dilution
**True Positives**	2	2	2	2	**50:50**
	1	2	2	2	**20:80**
	2	2	2	2	**10:90**
	2	2	2	2	**90:10**
	2	2	2	2	**80:20**
**Fusion-supporting reads**	CLIP2-MET: 328<br>FGFR3-TACC3: 3006	TPM3-NTRK1: 3792<br>EML4-ALK: 148	FGFR3-TACC3: 7905<br>SLC34A2-ROS1: 6054	FGFR3-BAIAP2L1: 4673<br>FGFR1OP2-FGFR1: 3521	**50:50**
	CLIP2-MET: -<br>FGFR3-TACC3: 5164	TPM3-NTRK1: 599<br>EML4-ALK: 351	FGFR3-TACC3: 2292<br>SLC34A2-ROS1: 10599	FGFR3-BAIAP2L1: 1109<br>FGFR1OP2-FGFR1: 4126	**20:80**
	CLIP2-MET: 194<br>FGFR3-TACC3: 10635	TPM3-NTRK1: 348<br>EML4-ALK: 500	FGFR3-TACC3: 481<br>SLC34A2-ROS1: 10824	FGFR3-BAIAP2L1: 1031<br>FGFR1OP2-FGFR1: 10460	**10:90**
	CLIP2-MET: 716<br>FGFR3-TACC3: 656	TPM3-NTRK1: 6092<br>EML4-ALK: 90	FGFR3-TACC3: 16716<br>SLC34A2-ROS1: 2712	FGFR3-BAIAP2L1: 3131<br>FGFR1OP2-FGFR1: 524	**90:10**
	CLIP2-MET: 514<br>FGFR3-TACC3: 933	TPM3-NTRK1: 200<br>EML4-ALK:5005	FGFR3-TACC3: 4504<br>SLC34A2-ROS1: 4183	FGFR3-BAIAP2L1: 3097<br>FGFR1OP2-FGFR1: 990	**80:20**
**False Positives**	12	7	16	10	**50:50**
	12	4	16	9	**20:80**
	13	2	16	6	**10:90**
	3	11	19	9	**90:10**
	6	13	20	7	**80:20**
**Missed Fusions**	0	0	0	0	**50:50**
	1	0	0	0	**20:80**
	0	0	0	0	**10:90**
	0	0	0	0	**90:10**
	0	0	0	0	**80:20**
