# Supplementary material for: Detection of gene fusions using targeted next-generation sequencing: a comparative evaluation
Source: BMC Med Genomics. 2021 Feb 27;14:62. doi: 10.1186/s12920-021-00909-y (PMC7912891; doi:10.1186/s12920-021-00909-y)
Supplement: Supplementary file 4 — Additional file 4: Fig. S4. Results of Archer FusionPlex Lung Panel (Archer DX) (v5.1.3) for the cell line mixtures. Shown are the number of true positive fusions detected, the number of fusion-supporting reads for this fusion, as well as the number of false positives and missed fusions identified per cell line dilution. [file 12920_2021_909_MOESM4_ESM.pdf]

Archer FusionPlex Lung Panel (Archer DX) v5.1.3)	SJ-GBM2: CLIP2-MET<br>RT112: FGFR3-TACC3	KM-12: TPM3-NTRK1<br>H2228: EML4-ALK	RT4: FGFR3-TACC3<br>HCC-78: SLC34A2-ROS1	SW780: FGFR3-BAIAP2L1<br>KG-1: FGFR1OP2-FGFR1	Dilution
**True Positives**	2	2	2	2	**50:50**
	2	2	2	2	**20:80**
	1	2	2	2	**10:90**
	2	2	2	2	**90:10**
	2	2	2	2	**80:20**
**Fusion-supporting reads**	CLIP2-MET: 664	TPM3-NTRK1: 3964	FGFR3-TACC3: 6367	FGFR3-BAIAP2L1: 4822	**50:50**
	FGFR3-TACC3: 4278	EML4-ALK: 326	SLC34A2-ROS1: 8070	FGFR1OP2-FGFR1: 3416	
	CLIP2-MET: 467	TPM3-NTRK1: 1049	FGFR3-TACC3: 1997	FGFR3-BAIAP2L1: 2672	**20:80**
	FGFR3-TACC3: 11769	EML4-ALK: 472	SLC34A2-ROS1: 15207	FGFR1OP2-FGFR1: 7968	
	CLIP2-MET: -	TPM3-NTRK1: 327	FGFR3-TACC3: 922	FGFR3-BAIAP2L1:1609	**10:90**
	FGFR3-TACC3: 12834	EML4-ALK: 498	SLC34A2-ROS1: 16964	FGFR1OP2-FGFR1: 9120	
	CLIP2-MET: 1354	TPM3-NTRK1: 10360	FGFR3-TACC3: 7676	FGFR3-BAIAP2L1: 6588	**90:10**
	FGFR3-TACC3: 688	EML4-ALK: 85	SLC34A2-ROS1: 2717	FGFR1OP2-FGFR1: 521	
	CLIP2-MET: 882	TPM3-NTRK1: 9147	FGFR3-TACC3: 14632	FGFR3-BAIAP2L1: 5906	**80:20**
	FGFR3-TACC3: 1475	EML4-ALK: 190	SLC34A2-ROS1: 4274	FGFR1OP2-FGFR1: 942	
**False Positives**	5	5	9	4	**50:50**
	6	5	8	5	**20:80**
	7	3	7	2	**10:90**
	1	1	9	5	**90:10**
	2	5	11	3	**80:20**
**Missed Fusions**	0	0	0	0	**50:50**
	0	0	0	0	**20:80**
	1	0	0	0	**10:90**
	0	0	0	0	**90:10**
	0	0	0	0	**80:20**
