# Supplementary material for: Detection of gene fusions using targeted next-generation sequencing: a comparative evaluation
Source: BMC Med Genomics. 2021 Feb 27;14:62. doi: 10.1186/s12920-021-00909-y (PMC7912891; doi:10.1186/s12920-021-00909-y)
Supplement: Supplementary file 5 — Additional file 5: Fig. S5. Results of QIAseq Targeted RNAscan Custom Panel (Qiagen) for the cell line mixtures. Shown are the number of true positive fusions detected, the number of fusion-supporting reads for this fusion, as well as the number of false positives and missed fusions identified per cell line dilution. [file 12920_2021_909_MOESM5_ESM.pdf]

QIAseq Targeted RNAscan Custom Panel (Qiagen)	SJ-GBM2: CLIP2-MET<br>RT112: FGFR3-TACC3	KM-12: TPM3-NTRK1<br>H2228: EML4-ALK	RT4: FGFR3-TACC3<br>HCC-78: SLC34A2-ROS1	SW780: FGFR3-BAIAP2L1<br>KG-1: FGFR1OP2-FGFR1	Dilution
**True Positives**	2	2	2	2	**50:50**
	2	2	1	2	**20:80**
	2	2	2	2	**10:90**
	2	2	2	2	**90:10**
	2	2	2	2	**80:20**
**Fusion-supporting reads**	CLIP2-MET: 11<br>FGFR3-TACC3: 990	TPM3-NTRK1: 1106<br>EML4-ALK: 49	FGFR3-TACC3: 6<br>SLC34A2-ROS1: 1622	FGFR3-BAIAP2L1: 1084<br>FGFR1OP2-FGFR1: 386	**50:50**
	CLIP2-MET: 2<br>FGFR3-TACC3: 1011	TPM3-NTRK1: 322<br>EML4-ALK: 73	FGFR3-TACC3: -<br>SLC34A2-ROS1: 2995	FGFR3-BAIAP2L1: 539<br>FGFR1OP2-FGFR1: 800	**20:80**
	CLIP2-MET: 7<br>FGFR3-TACC3: 2938	TPM3-NTRK1: 129<br>EML4-ALK: 90	FGFR3-TACC3: 2<br>SLC34A2-ROS1: 2409	FGFR3-BAIAP2L1: 282<br>FGFR1OP2-FGFR1: 1126	**10:90**
	CLIP2-MET: 400<br>FGFR3-TACC3: 94	TPM3-NTRK1: 2440<br>EML4-ALK: 12	FGFR3-TACC3: 21<br>SLC34A2-ROS1: 987	FGFR3-BAIAP2L1: 1232<br>FGFR1OP2-FGFR1: 53	**90:10**
	CLIP2-MET: 15<br>FGFR3-TACC3: 322	TPM3-NTRK1: 3509<br>EML4-ALK: 17	FGFR3-TACC3: 9<br>SLC34A2-ROS1: 1306	FGFR3-BAIAP2L1: 634<br>FGFR1OP2-FGFR1: 72	**80:20**
**False Positives**	13	19	2	9	**50:50**
	6	16	2	10	**20:80**
	17	9	1	12	**10:90**
	14	17	3	16	**90:10**
	14	14	6	14	**80:20**
**Missed Fusions**	0	0	0	0	**50:50**
	0	0	1	0	**20:80**
	0	0	0	0	**10:90**
	0	0	0	0	**90:10**
	0	0	0	0	**80:20**
