# Supplementary material for: Detection of gene fusions using targeted next-generation sequencing: a comparative evaluation
Source: BMC Med Genomics. 2021 Feb 27;14:62. doi: 10.1186/s12920-021-00909-y (PMC7912891; doi:10.1186/s12920-021-00909-y)
Supplement: Supplementary file 6 — Additional file 6: Fig. S6. Results of Oncomine Focus Assay (Thermo Fisher Scientific) for the cell line mixtures. Shown are the number of true positive fusions detected, the number of fusion-supporting reads for this fusion, as well as the number of false positives and missed fusions identified per cell line dilution. [file 12920_2021_909_MOESM6_ESM.pdf]

Oncomine Focus Assay (Thermo Fisher Scientific)	SJ-GBM2: CLIP2-MET<br>RT112: FGFR3-TACC3	KM-12: TPM3-NTRK1<br>H2228: EML4-ALK	RT4: FGFR3-TACC3<br>HCC-78: SLC34A2-ROS1	SW780: FGFR3-BAIAP2L1<br>KG-1: FGFR1OP2-FGFR1	Dilution
**True Positives**	1	2	2	1	**50:50**
	1	2	2	1	**20:80**
	1	1	2	1	**10:90**
	1	2	2	1	**90:10**
	1	2	2	1	**80:20**
**Fusion-supporting reads**	CLIP2-MET: -<br>FGFR3-TACC3: 320069	TPM3-NTRK1: 67457<br>EML4-ALK: 869	FGFR3-TACC3: 1302<br>SLC34A2-ROS1: 35000	FGFR3-BAIAP2L1: 32678<br>FGFR1OP2-FGFR1: -	**50:50**
	CLIP2-MET: -<br>FGFR3-TACC3: 41159	TPM3-NTRK1: 83085<br>EML4-ALK: 12669	FGFR3-TACC3: 203<br>SLC34A2-ROS1: 32759	FGFR3-BAIAP2L1: 27108<br>FGFR1OP2-FGFR1: -	**20:80**
	CLIP2-MET: -<br>FGFR3-TACC3: 35729	TPM3-NTRK1: 77048<br>EML4-ALK: 61122	FGFR3-TACC3: **5**<br>SLC34A2-ROS1: 26650	FGFR3-BAIAP2L1: 16490<br>FGFR1OP2-FGFR1: -	**10:90**
	CLIP2-MET: -<br>FGFR3-TACC3: 17348	TPM3-NTRK1: 192343<br>EML4-ALK: 3809	FGFR3-TACC3: 14344<br>SLC34A2-ROS1: 159210	FGFR3-BAIAP2L1: 29637<br>FGFR1OP2-FGFR1: -	**90:10**
	CLIP2-MET: -<br>FGFR3-TACC3: 21384	TPM3-NTRK1: 54293<br>EML4-ALK: 256	FGFR3-TACC3: 26037<br>SLC34A2-ROS1: 138724	FGFR3-BAIAP2L1: 29249<br>FGFR1OP2-FGFR1: -	**80:20**
**False Positives**	5	0	2	0	**50:50**
	4	0	2	0	**20:80**
	3	0	1	0	**10:90**
	2	0	3	0	**90:10**
	2	1	6	0	**80:20**
**Missed Fusions**	0	0	0	0	**50:50**
	0	0	0	0	**20:80**
	0	1	0	0	**10:90**
	0	0	0	0	**90:10**
	0	0	0	0	**80:20**
