# Supplementary material for: Detection of gene fusions using targeted next-generation sequencing: a comparative evaluation
Source: BMC Med Genomics. 2021 Feb 27;14:62. doi: 10.1186/s12920-021-00909-y (PMC7912891; doi:10.1186/s12920-021-00909-y)
Supplement: Supplementary file 7 — Additional file 7: Fig. S7. Results of TruSight Tumor 170 Assay (Illumina) for all for the cell line mixtures. Shown are the number of true positive fusions detected, the number of fusion-supporting reads for this fusion, as well as the number of false positives and missed fusions identified per cell line dilution. [file 12920_2021_909_MOESM7_ESM.pdf]

TruSight 170 Assay (Illumina)	SJ-GBM2: CLIP2-MET<br>RT112: FGFR3-TACC3	KM-12: TPM3-NTRK1<br>H2228: EML4-ALK	RT4: FGFR3-TACC3<br>HCC-78: SLC34A2-ROS1	SW780: FGFR3-BAIAP2L1<br>KG-1: FGFR1OP2-FGFR1	Dilution
**True Positives**	2<br>2<br>2<br>2<br>2	2<br>2<br>2<br>2<br>2	2<br>2<br>2<br>2<br>2	2<br>2<br>2<br>2<br>2	**50:50**<br>**20:80**<br>**10:90**<br>**90:10**<br>**80:20**
**Fusion-supporting reads**	CLIP2-MET: 1605<br>FGFR3-TACC3: 4836<br>CLIP2-MET: 767<br>FGFR3-TACC3: 10894<br>CLIP2-MET: 450<br>FGFR3-TACC3: 13905<br>CLIP2-MET: 2145<br>FGFR3-TACC3: 755<br>CLIP2-MET: 1579<br>FGFR3-TACC3: 1430	TPM3-NTRK1: 3681<br>EML4-ALK: 1149<br>TPM3-NTRK1: 1183<br>EML4-ALK: 1401<br>TPM3-NTRK1: 454<br>EML4-ALK: 1317<br>TPM3-NTRK1: 5363<br>EML4-ALK: 166<br>TPM3-NTRK1: 6525<br>EML4-ALK: 541	FGFR3-TACC3: 10826<br>SLC34A2-ROS1: 8527<br>FGFR3-TACC3: 2125<br>SLC34A2-ROS1: 33436<br>FGFR3-TACC3: 702<br>SLC34A2-ROS1:11195<br>FGFR3-TACC3: 16630<br>SLC34A2-ROS1: 6504<br>FGFR3-TACC3: 15615<br>SLC34A2-ROS1: 13413	FGFR3-BAIAP2L1: 6433<br>FGFR1OP2-FGFR1: 2107<br>FGFR3-BAIAP2L1: 3428<br>FGFR1OP2-FGFR1: 4710<br>FGFR3-BAIAP2L1: 1759<br>FGFR1OP2-FGFR1: 4417<br>FGFR3-BAIAP2L1: 9360<br>FGFR1OP2-FGFR1: 504<br>FGFR3-BAIAP2L1: 6916<br>FGFR1OP2-FGFR1: 980	**50:50**<br><br>**20:80**<br><br>**10:90**<br><br>**90:10**<br><br>**80:20**
**False Positives**	1<br>1<br>0<br>0<br>0	2<br>2<br>2<br>3<br>2	1<br>4<br>3<br>3<br>4	2<br>1<br>1<br>0<br>0	**50:50**<br>**20:80**<br>**10:90**<br>**90:10**<br>**80:20**
**Missed Fusions**	0<br>0<br>0<br>0<br>0	0<br>0<br>0<br>0<br>0	0<br>0<br>0<br>0<br>0	0<br>0<br>0<br>0<br>0	**50:50**<br>**20:80**<br>**10:90**<br>**90:10**<br>**80:20**
