# Supplementary material for: Detection of gene fusions using targeted next-generation sequencing: a comparative evaluation
Source: BMC Med Genomics. 2021 Feb 27;14:62. doi: 10.1186/s12920-021-00909-y (PMC7912891; doi:10.1186/s12920-021-00909-y)
Supplement: Supplementary file 8 — Additional file 8: Fig. S8. Results of SureSelect XT HS Custom Panel (Agilent) (v4.0.1.46) for the cell line mixtures. Shown are the number of true positive fusions detected, the number of fusion-supporting reads for this fusion, as well as the number of false positives and missed fusions identified per cell line dilution. [file 12920_2021_909_MOESM8_ESM.pdf]

SureSelect XT HS Custom Panel (Agilent) v4.0.1.46	SJ-GBM2: CLIP2-MET<br>RT112: FGFR3-TACC3	KM-12: TPM3-NTRK1<br>H2228: EML4-ALK	RT4: FGFR3-TACC3<br>HCC-78: SLC34A2-ROS1	SW780: FGFR3-BAIAP2L1<br>KG-1: FGFR1OP2-FGFR1	Dilution
True Positives	1	2	1	2	**50:50**
	2	2	1	2	**20:80**
	2	2	1	2	**10:90**
	2	2	2	2	**90:10**
	2	2	2	2	**80:20**
Fusion-supporting reads	CLIP2-MET: 69<br>FGFR3-TACC3:-	TPM3-NTRK1: 143<br>EML4-ALK: 157	FGFR3-TACC3: -<br>SLC34A2-ROS1: 280	FGFR3-BAIAP2L1: 119<br>FGFR1OP2-FGFR1: 44	**50:50**
	CLIP2-MET: 59<br>FGFR3-TACC3: 754	TPM3-NTRK1: 41<br>EML4-ALK: 168	FGFR3-TACC3:-<br>SLC34A2-ROS1: 450	FGFR3-BAIAP2L1: 69<br>FGFR1OP2-FGFR1: 110	**20:80**
	CLIP2-MET: 21<br>FGFR3-TACC3: 1483	TPM3-NTRK1: 27<br>EML4-ALK: 218	FGFR3-TACC3:-<br>SLC34A2-ROS1: 386	FGFR3-BAIAP2L1: 87<br>FGFR1OP2-FGFR1: 332	**10:90**
	CLIP2-MET: 208<br>FGFR3-TACC3: 63	TPM3-NTRK1: 209<br>EML4-ALK: 23	FGFR3-TACC3: 1943<br>SLC34A2-ROS1: 71	FGFR3-BAIAP2L1: 275<br>FGFR1OP2-FGFR1: 12	**90:10**
	CLIP2-MET: 36<br>FGFR3-TACC3: 33	TPM3-NTRK1: 237<br>EML4-ALK: 62	FGFR3-TACC3: 1833<br>SLC34A2-ROS1: 51	FGFR3-BAIAP2L1: 87<br>FGFR1OP2-FGFR1: 11	**80:20**
False Positives	23	16	45	16	**50:50**
	33	20	23	17	**20:80**
	16	17	32	20	**10:90**
	32	16	38	21	**90:10**
	16	22	30	13	**80:20**
Missed Fusions	1	0	1	0	**50:50**
	0	0	1	0	**20:80**
	0	0	1	0	**10:90**
	0	0	0	0	**90:10**
	0	0	0	0	**80:20**
