# Supplementary material for: Detection of gene fusions using targeted next-generation sequencing: a comparative evaluation
Source: BMC Med Genomics. 2021 Feb 27;14:62. doi: 10.1186/s12920-021-00909-y (PMC7912891; doi:10.1186/s12920-021-00909-y)
Supplement: Supplementary file 9 — Additional file 9: Fig. S9. Results of SureSelect XT HS Custom Panel (Agilent) (v4.1.1.5) for the cell line mixtures. Shown are the number of true positive fusions detected, the number of fusion-supporting reads for this fusion, as well as the number of false positives and missed fusions identified per cell line dilution. [file 12920_2021_909_MOESM9_ESM.pdf]

SureSelect XT HS Custom Panel (Agilent) v4.1.1.5	SJ-GBM2: CLIP2-MET<br>RT112: FGFR3-TACC3	KM-12: TPM3-NTRK1<br>H2228: EML4-ALK	RT4: FGFR3-TACC3<br>HCC-78: SLC34A2-ROS1	SW780: FGFR3-BAIAP2L1<br>KG-1: FGFR1OP2-FGFR1	Dilution
**True Positives**	2	2	2	2	**50:50**
	2	2	2	2	**20:80**
	1	2	2	2	**10:90**
	2	2	2	2	**90:10**
	2	2	2	2	**80:20**
**Fusion-supporting reads**	CLIP2-MET: 69	TPM3-NTRK1: 142	FGFR3-TACC3: 979	FGFR3-BAIAP2L1: 116	**50:50**
	FGFR3-TACC3: 279	EML4-ALK: 155	SLC34A2-ROS1: 271	FGFR1OP2-FGFR1: 44	
	CLIP2-MET: 59	TPM3-NTRK1: 41	FGFR3-TACC3: 477	FGFR3-BAIAP2L1: 67	**20:80**
	FGFR3-TACC3: 739	EML4-ALK: 165	SLC34A2-ROS1: 432	FGFR1OP2-FGFR1: 110	
	CLIP2-MET: -	TPM3-NTRK1: 27	FGFR3-TACC3: 169	FGFR3-BAIAP2L1: 81	**10:90**
	FGFR3-TACC3: 1450	EML4-ALK: 215	SLC34A2-ROS1: 292	FGFR1OP2-FGFR1: 282	
	CLIP2-MET: 208	TPM3-NTRK1: 209	FGFR3-TACC3: 1828	FGFR3-BAIAP2L1: 266	**90:10**
	FGFR3-TACC3: 61	EML4-ALK: 23	SLC34A2-ROS1: 49	FGFR1OP2-FGFR1: 12	
	CLIP2-MET: 36	TPM3-NTRK1: 236	FGFR3-TACC3: 1942	FGFR3-BAIAP2L1: 84	**80:20**
	FGFR3-TACC3: 32	EML4-ALK: 59	SLC34A2-ROS1: 98	FGFR1OP2-FGFR1: 11	
**False Positives**	8	10	11	9	**50:50**
	13	9	10	10	**20:80**
	15	9	11	13	**10:90**
	14	11	14	8	**90:10**
	10	13	21	8	**80:20**
**Missed Fusions**	0	0	0	0	**50:50**
	0	0	0	0	**20:80**
	1	0	0	0	**10:90**
	0	0	0	0	**90:10**
	0	0	0	0	**80:20**
