# Supplementary material for: Detection of gene fusions using targeted next-generation sequencing: a comparative evaluation
Source: BMC Med Genomics. 2021 Feb 27;14:62. doi: 10.1186/s12920-021-00909-y (PMC7912891; doi:10.1186/s12920-021-00909-y)
Supplement: Supplementary file 10 — Additional file 10: Fig. S10. Fusions detected with the Archer FusionPlex Lung Panel (Archer DX) (v5.0.4) for all samples. Metrics such as quality control scores, in-frame status or filter thresholds were plotted when available. In cases where the same fusion was identified more than once within the same sample, a unique numbering scheme was added at the end of the name to differentiate the candidate fusions. The numbering however, does not imply any special order or preference over the other fusions with the same name. The putative detected fusions were arranged in decreasing order based on the number of fusion-supporting reads. The expected fusion for each sample was highlighted in bold. [file 12920_2021_909_MOESM10_ESM.pdf]

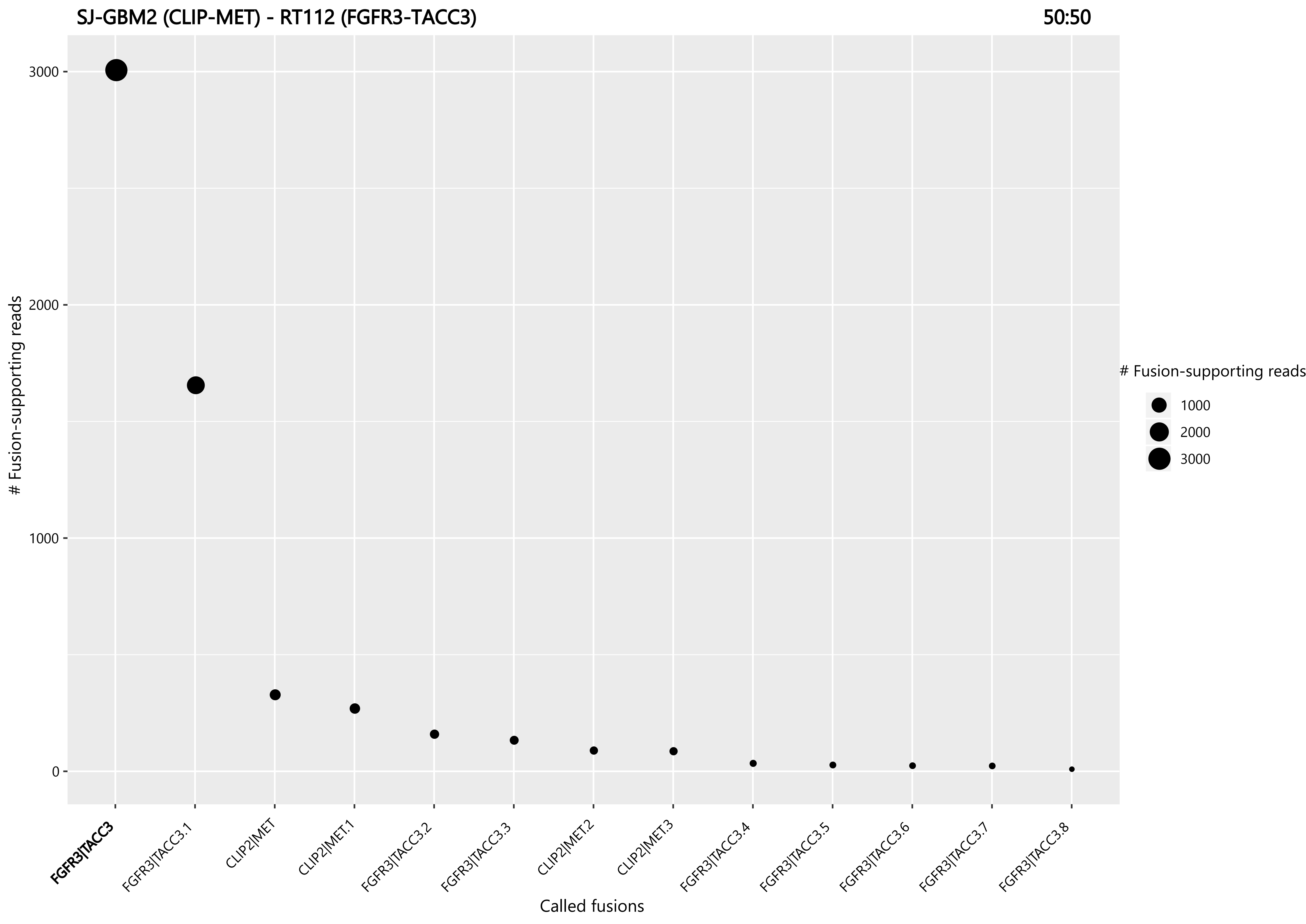
SJ-GBM2 (CLIP-MET) - RT112 (FGFR3-TACC3)
50:50
Fusion-supporting reads
3000
2000
1000
0
Called fusions
FGFR3|TACC3
FGFR3|TACC3.1
CLIP2|MET
CLIP2|MET.1
FGFR3|TACC3.2
FGFR3|TACC3.3
CLIP2|MET.2
CLIP2|MET.3
FGFR3|TACC3.4
FGFR3|TACC3.5
FGFR3|TACC3.6
FGFR3|TACC3.7
FGFR3|TACC3.8
Fusion-supporting reads
1000
2000
3000

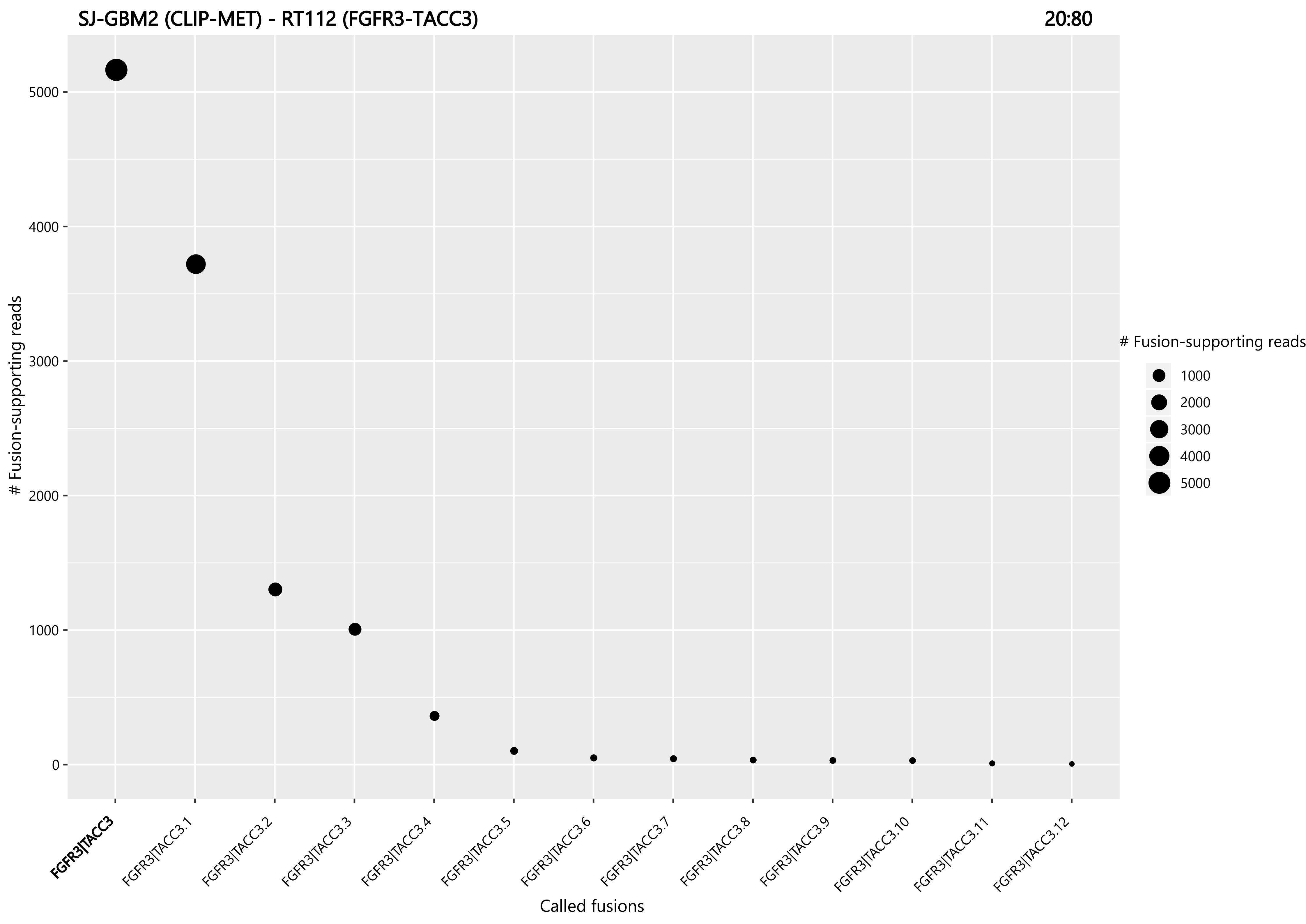

SJ-GBM2 (CLIP-MET) - RT112 (FGFR3-TACC3)
20:80
Fusion-supporting reads
0
1000
2000
3000
4000
5000
Called fusions
FGFR3|TACC3
FGFR3|TACC3.1
FGFR3|TACC3.2
FGFR3|TACC3.3
FGFR3|TACC3.4
FGFR3|TACC3.5
FGFR3|TACC3.6
FGFR3|TACC3.7
FGFR3|TACC3.8
FGFR3|TACC3.9
FGFR3|TACC3.10
FGFR3|TACC3.11
FGFR3|TACC3.12
Fusion-supporting reads
1000
2000
3000
4000
5000

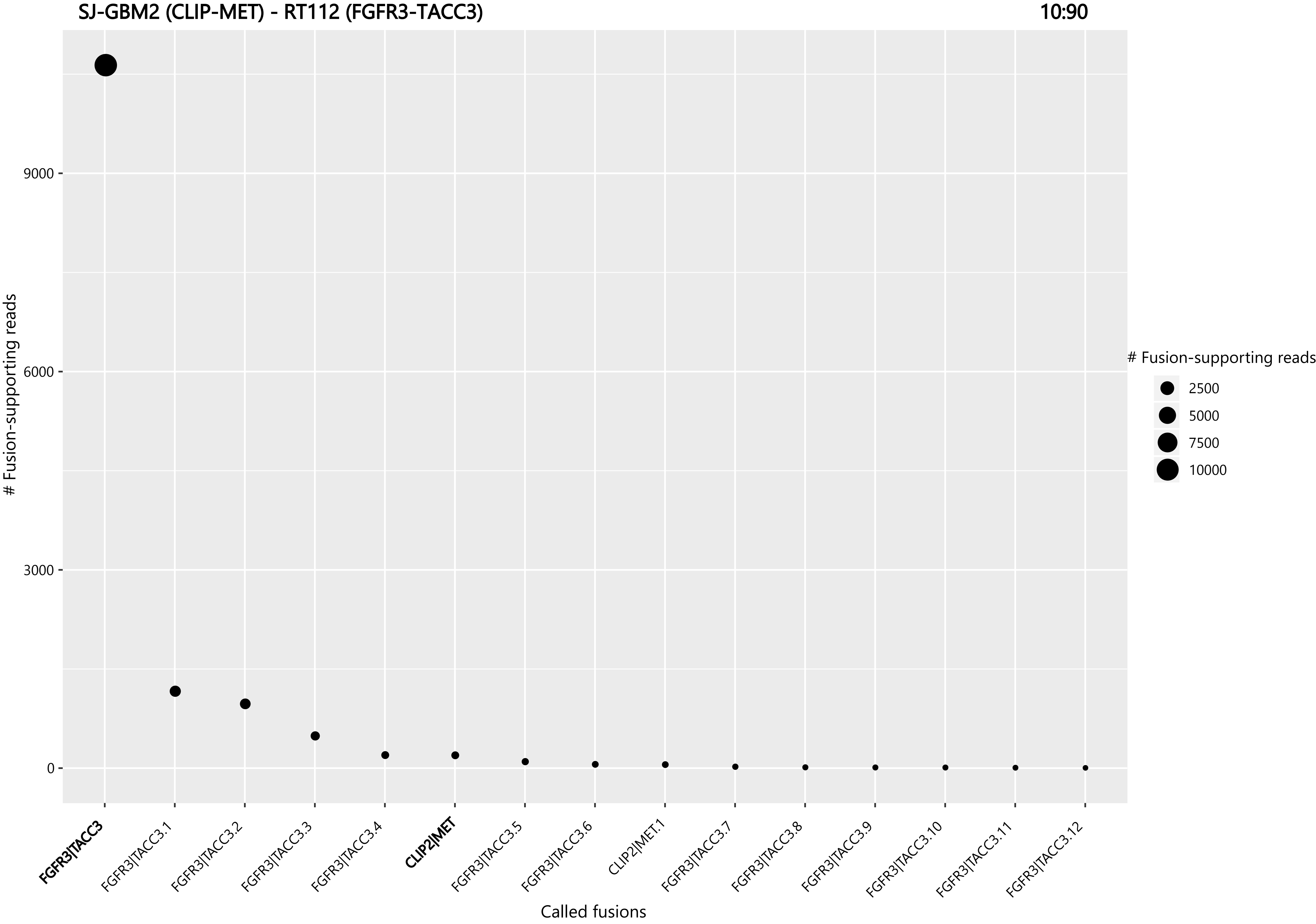

SJ-GBM2 (CLIP-MET) - RT112 (FGFR3-TACC3)
10:90
Fusion-supporting reads
9000
6000
3000
0
Fusion-supporting reads
2500
5000
7500
10000
FGFR3|TACC3
FGFR3|TACC3.1
FGFR3|TACC3.2
FGFR3|TACC3.3
FGFR3|TACC3.4
CLIP2|MET
FGFR3|TACC3.5
FGFR3|TACC3.6
CLIP2|MET.1
FGFR3|TACC3.7
FGFR3|TACC3.8
FGFR3|TACC3.9
FGFR3|TACC3.10
FGFR3|TACC3.11
FGFR3|TACC3.12
Called fusions

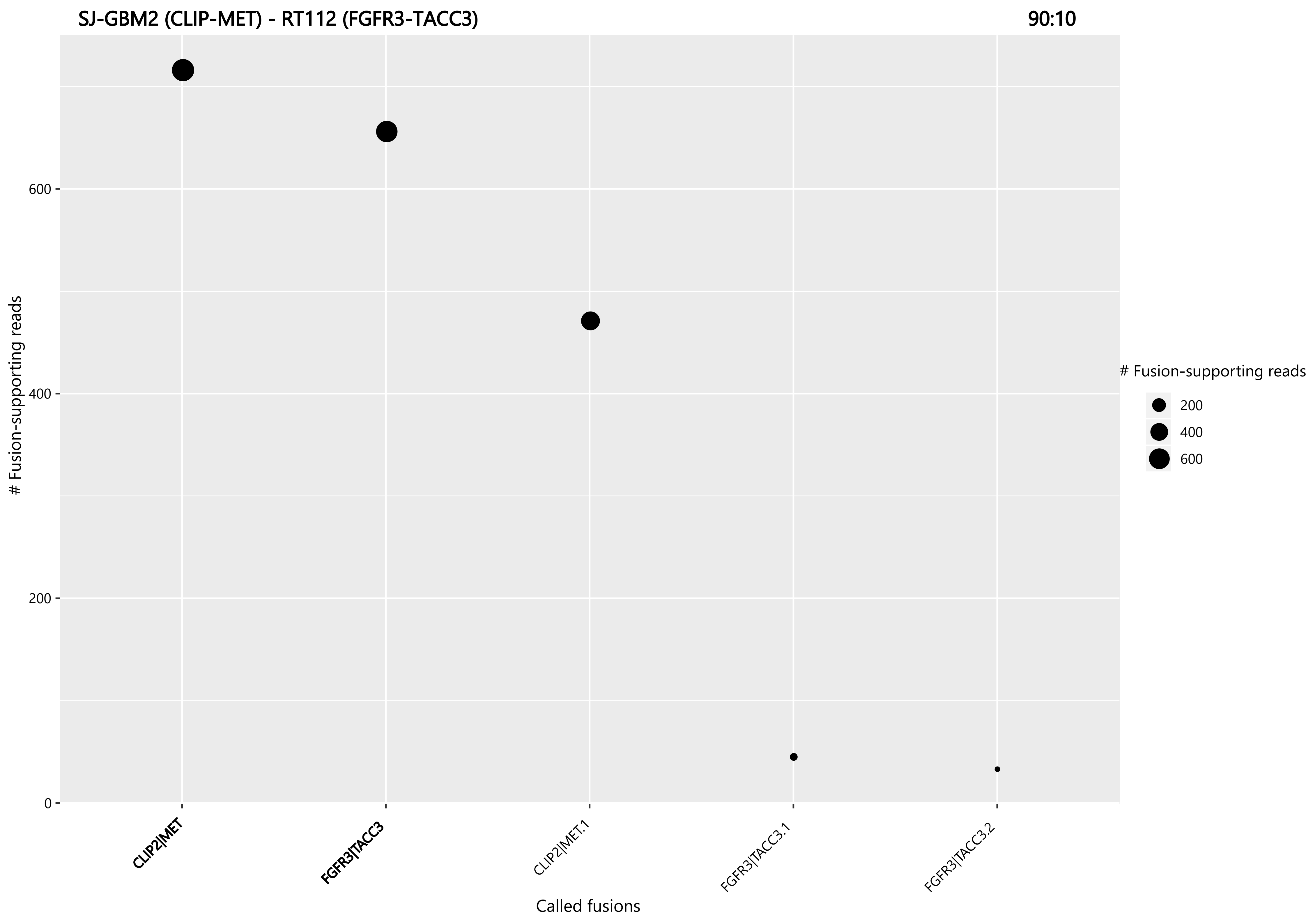
SJ-GBM2 (CLIP-MET) - RT112 (FGFR3-TACC3)
90:10
Fusion-supporting reads
0
200
400
600
Called fusions
CLIP2|MET
FGFR3|TACC3
CLIP2|MET.1
FGFR3|TACC3.1
FGFR3|TACC3.2
Fusion-supporting reads
200
400
600

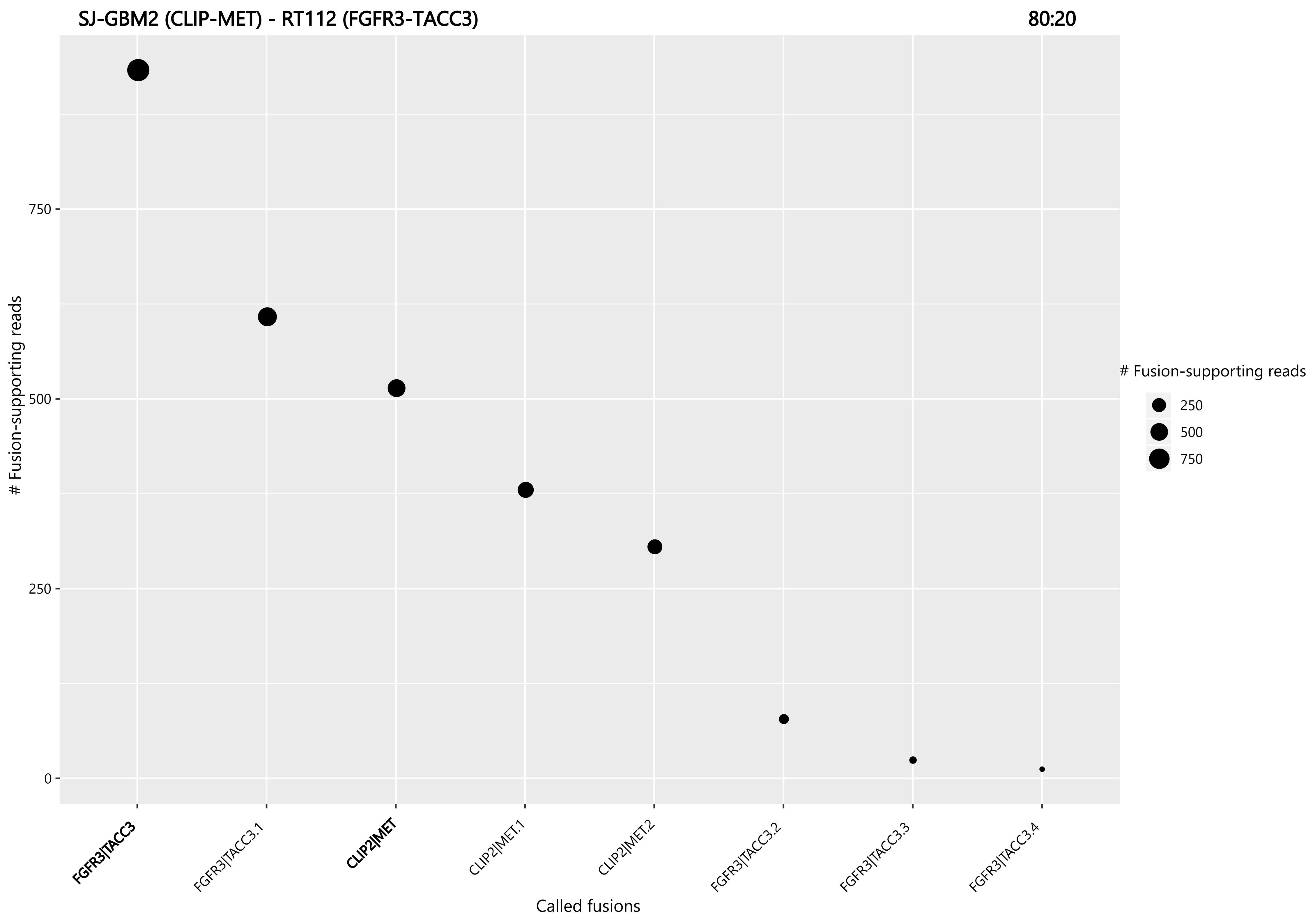

SJ-GBM2 (CLIP-MET) - RT112 (FGFR3-TACC3)
80:20
Fusion-supporting reads
750
500
250
0
FGFR3|TACC3
FGFR3|TACC3.1
CLIP2|MET
CLIP2|MET.1
CLIP2|MET.2
FGFR3|TACC3.2
FGFR3|TACC3.3
FGFR3|TACC3.4
Called fusions
Fusion-supporting reads
250
500
750

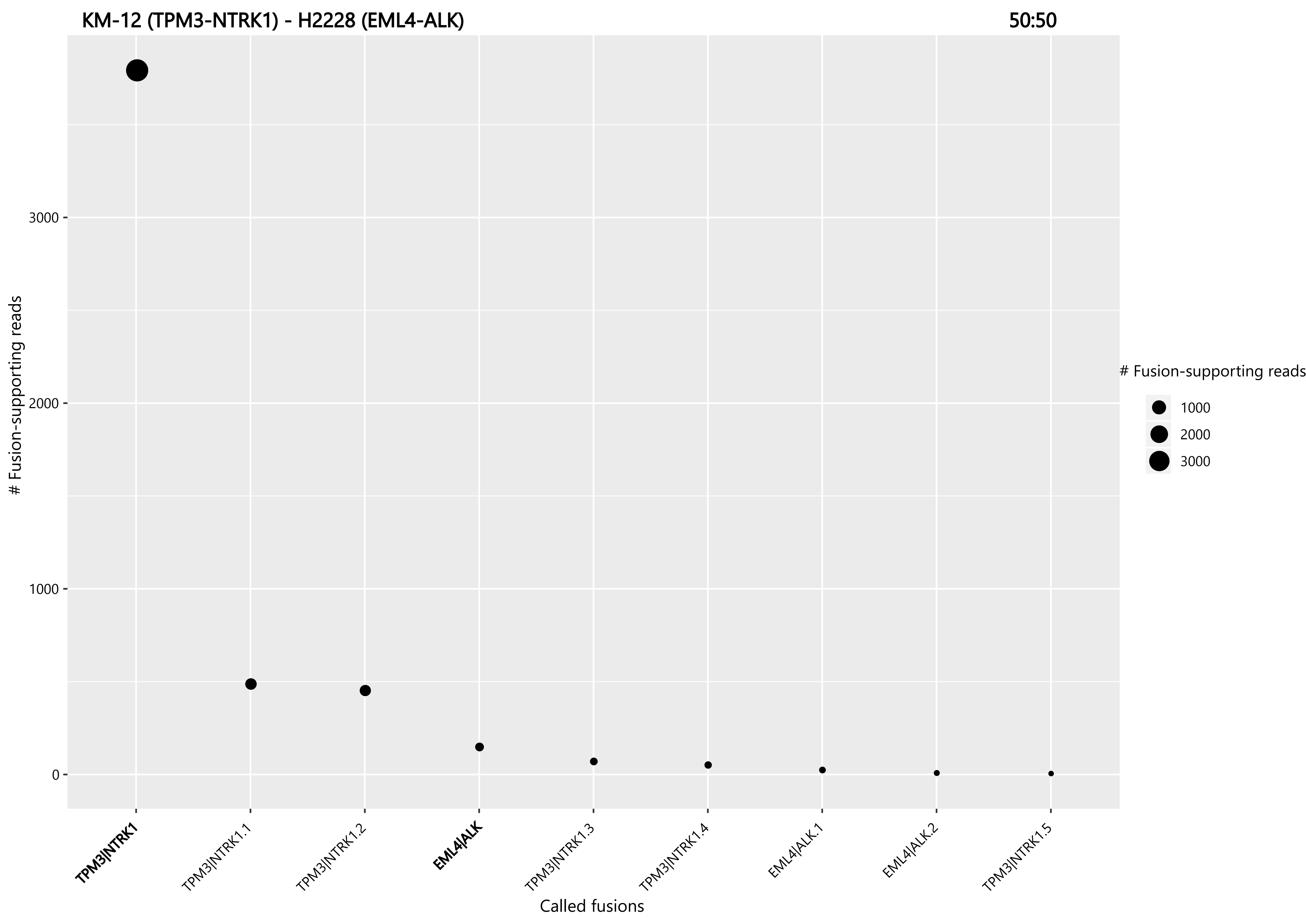
KM-12 (TPM3-NTRK1) - H2228 (EML4-ALK)
50:50
Fusion-supporting reads
3000
2000
1000
0
TPM3|NTRK1
TPM3|NTRK1.1
TPM3|NTRK1.2
EML4|ALK
TPM3|NTRK1.3
TPM3|NTRK1.4
EML4|ALK.1
EML4|ALK.2
TPM3|NTRK1.5
Called fusions
Fusion-supporting reads
1000
2000
3000

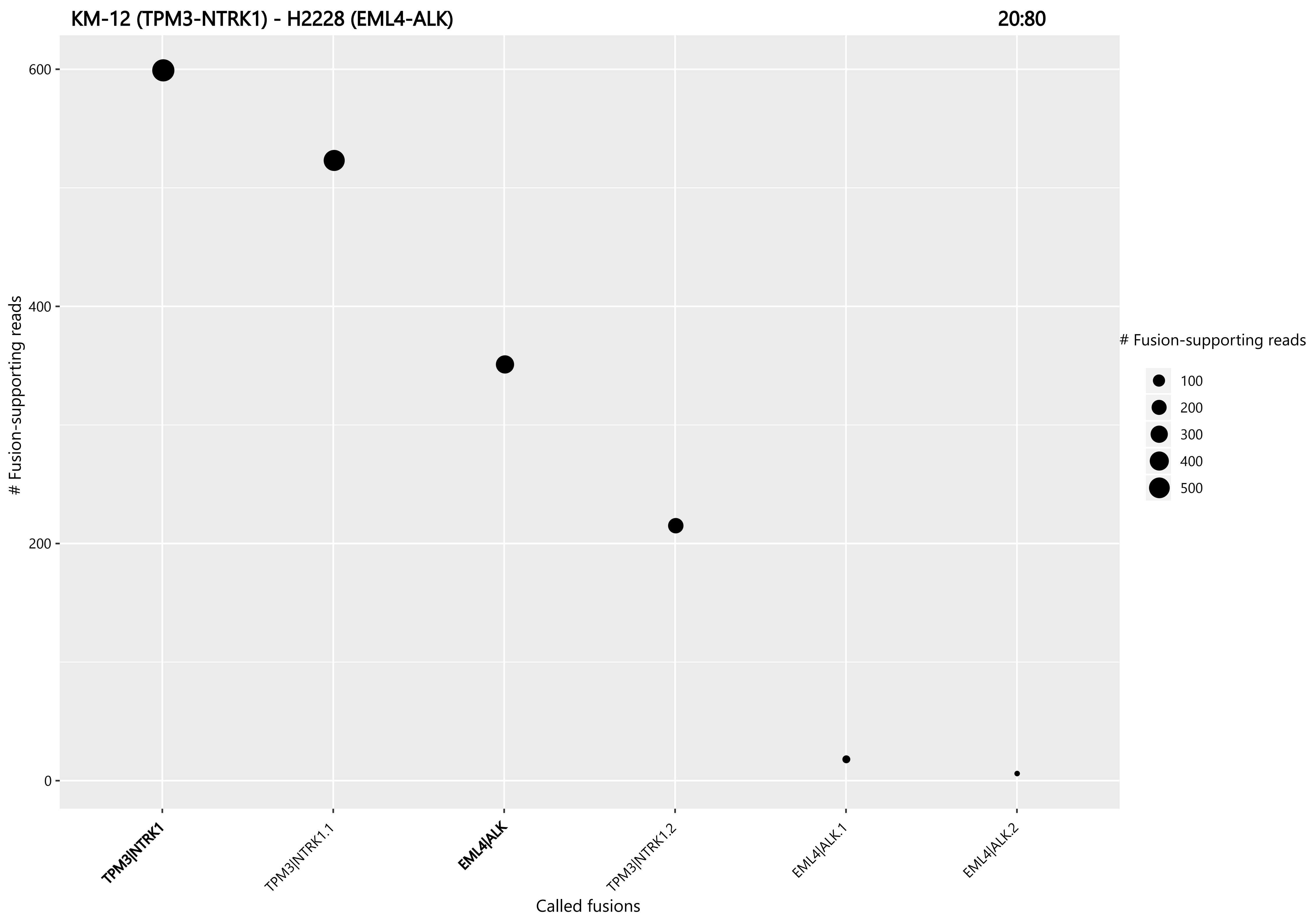
KM-12 (TPM3-NTRK1) - H2228 (EML4-ALK)
20:80
Fusion-supporting reads
600
400
200
0
TPM3|NTRK1
TPM3|NTRK1.1
EML4|ALK
TPM3|NTRK1.2
EML4|ALK.1
EML4|ALK.2
Called fusions
Fusion-supporting reads
100
200
300
400
500

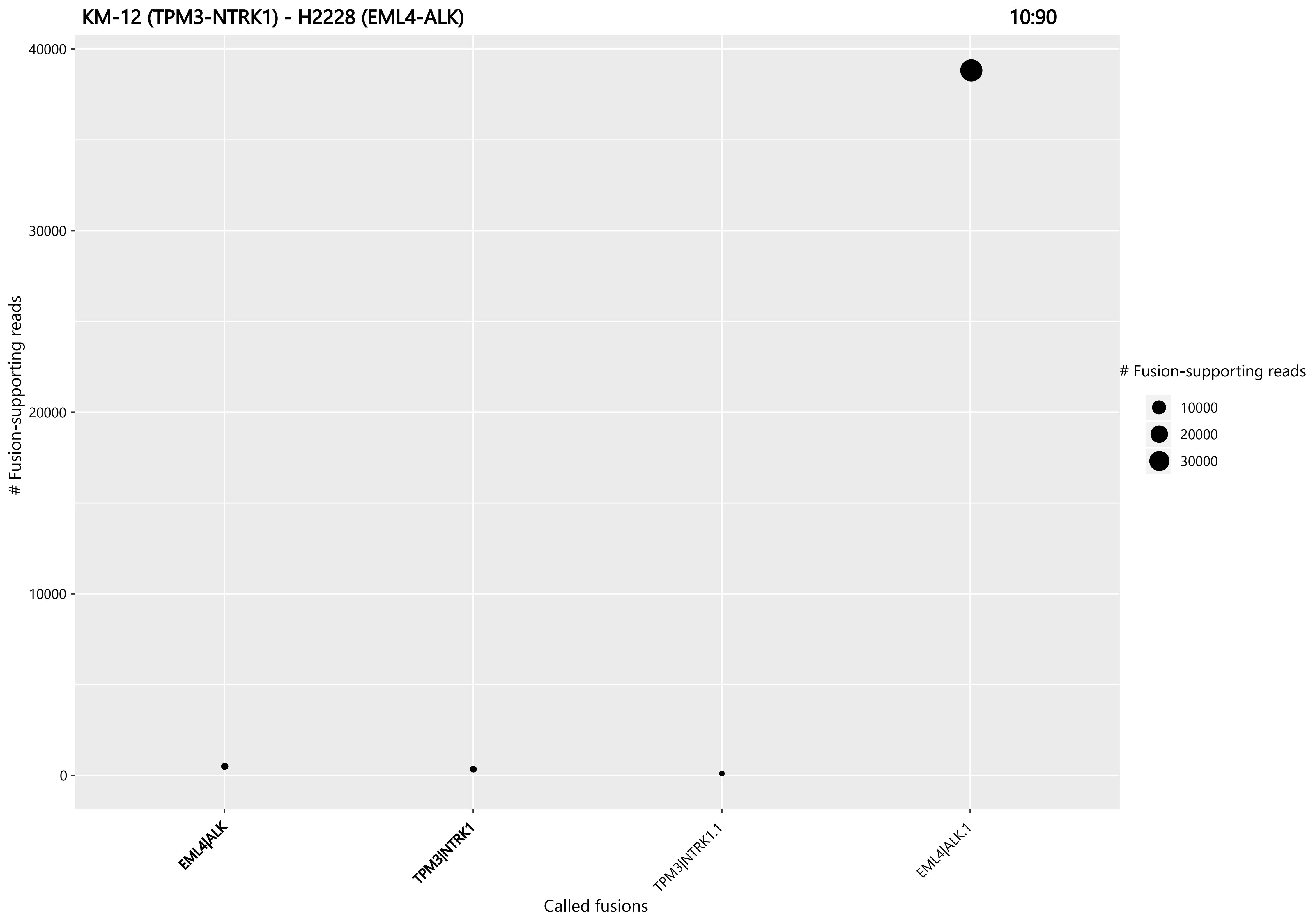

KM-12 (TPM3-NTRK1) - H2228 (EML4-ALK)
10:90
Fusion-supporting reads
40000
30000
20000
10000
0
EML4|ALK
TPM3|NTRK1
TPM3|NTRK1.1
EML4|ALK.1
Called fusions
Fusion-supporting reads
10000
20000
30000

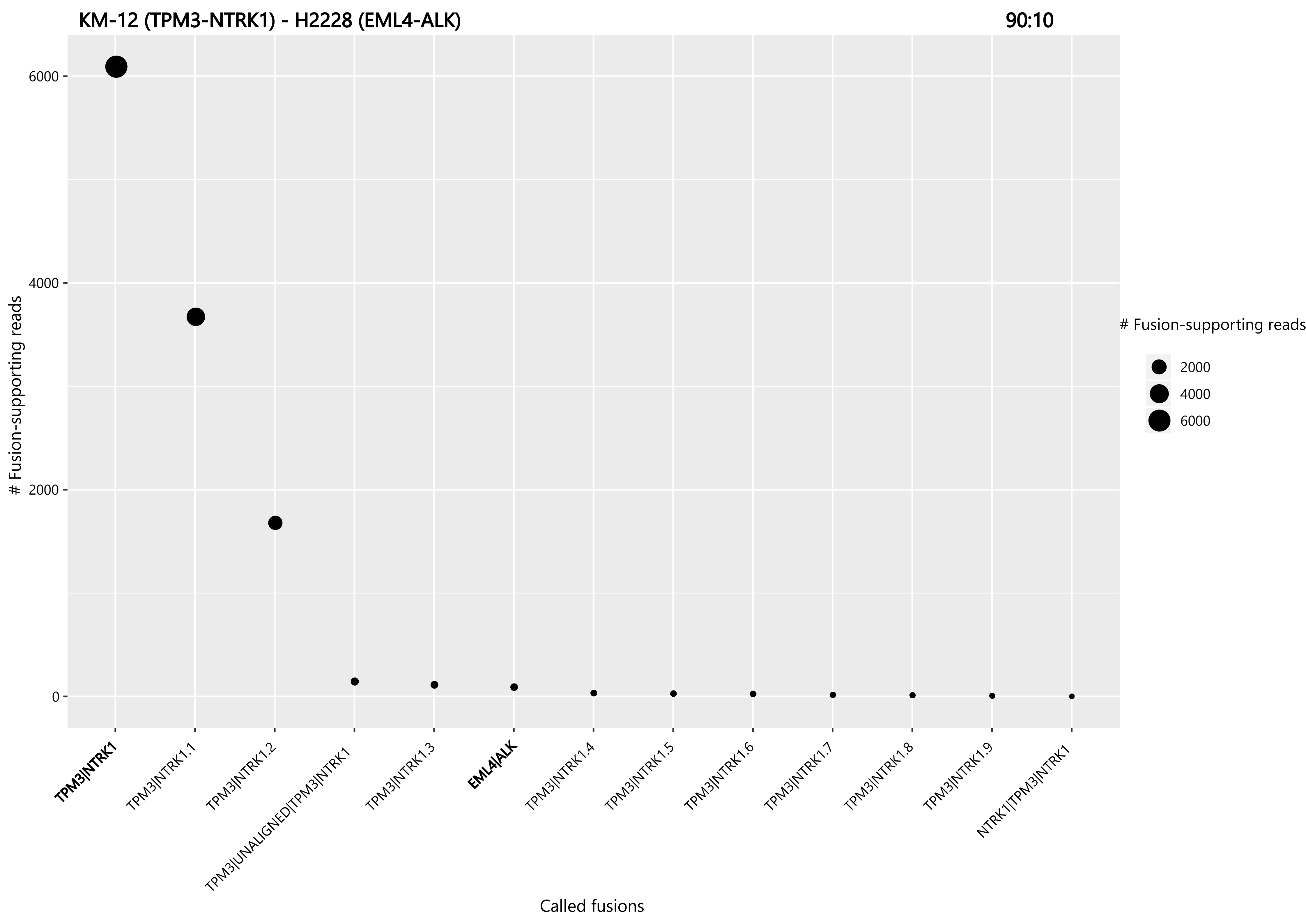

KM-12 (TPM3-NTRK1) - H2228 (EML4-ALK)
90:10
Fusion-supporting reads
6000
4000
2000
0
Fusion-supporting reads
2000
4000
6000
TPM3|NTRK1
TPM3|NTRK1.1
TPM3|NTRK1.2
TPM3|UNALIGNED|TPM3|NTRK1
TPM3|NTRK1.3
EML4|ALK
TPM3|NTRK1.4
TPM3|NTRK1.5
TPM3|NTRK1.6
TPM3|NTRK1.7
TPM3|NTRK1.8
TPM3|NTRK1.9
NTRK1|TPM3|NTRK1
Called fusions

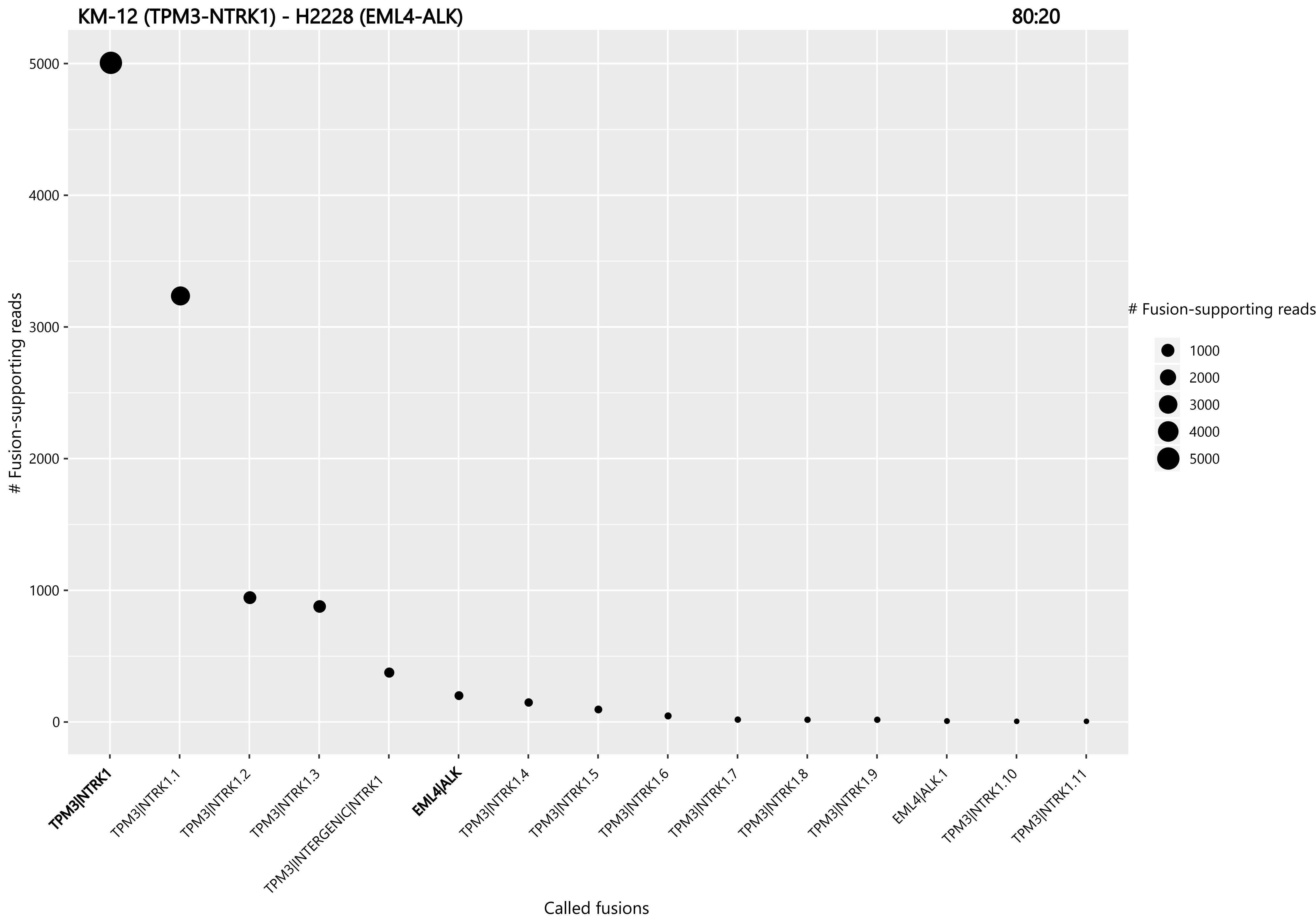

KM-12 (TPM3-NTRK1) - H2228 (EML4-ALK)
80:20
Fusion-supporting reads
5000
4000
3000
2000
1000
0
Called fusions
TPM3|NTRK1
TPM3|NTRK1.1
TPM3|NTRK1.2
TPM3|NTRK1.3
TPM3|INTERGENIC|NTRK1
EML4|ALK
TPM3|NTRK1.4
TPM3|NTRK1.5
TPM3|NTRK1.6
TPM3|NTRK1.7
TPM3|NTRK1.8
TPM3|NTRK1.9
EML4|ALK.1
TPM3|NTRK1.10
TPM3|NTRK1.11
Fusion-supporting reads
1000
2000
3000
4000
5000

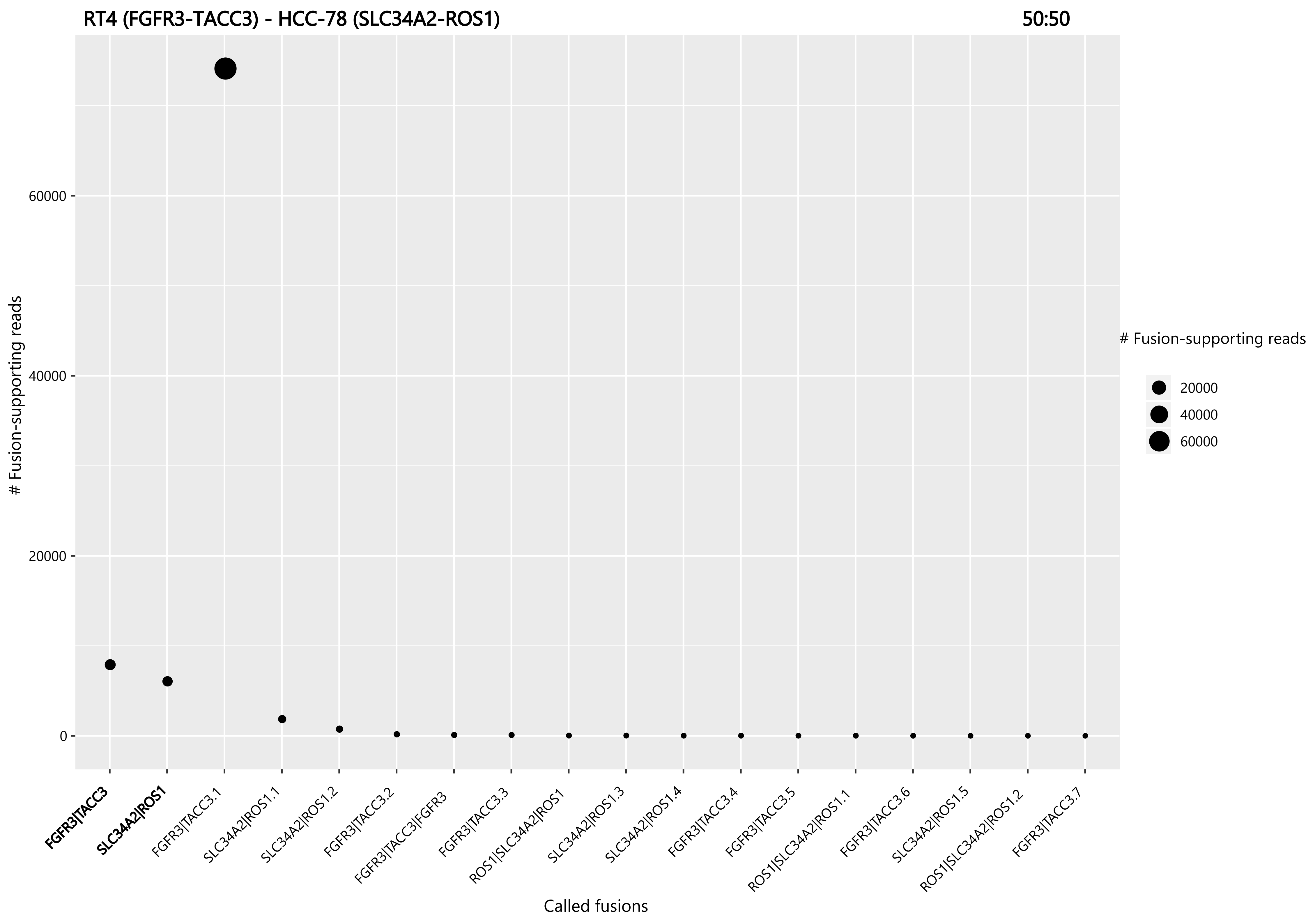
RT4 (FGFR3-TACC3) - HCC-78 (SLC34A2-ROS1)
50:50
Fusion-supporting reads
0
20000
40000
60000
Fusion-supporting reads
20000
40000
60000
FGFR3|TACC3
SLC34A2|ROS1
FGFR3|TACC3.1
SLC34A2|ROS1.1
SLC34A2|ROS1.2
FGFR3|TACC3.2
FGFR3|TACC3|FGFR3
FGFR3|TACC3.3
ROS1|SLC34A2|ROS1
SLC34A2|ROS1.3
SLC34A2|ROS1.4
FGFR3|TACC3.4
FGFR3|TACC3.5
ROS1|SLC34A2|ROS1.1
FGFR3|TACC3.6
SLC34A2|ROS1.5
ROS1|SLC34A2|ROS1.2
FGFR3|TACC3.7
Called fusions

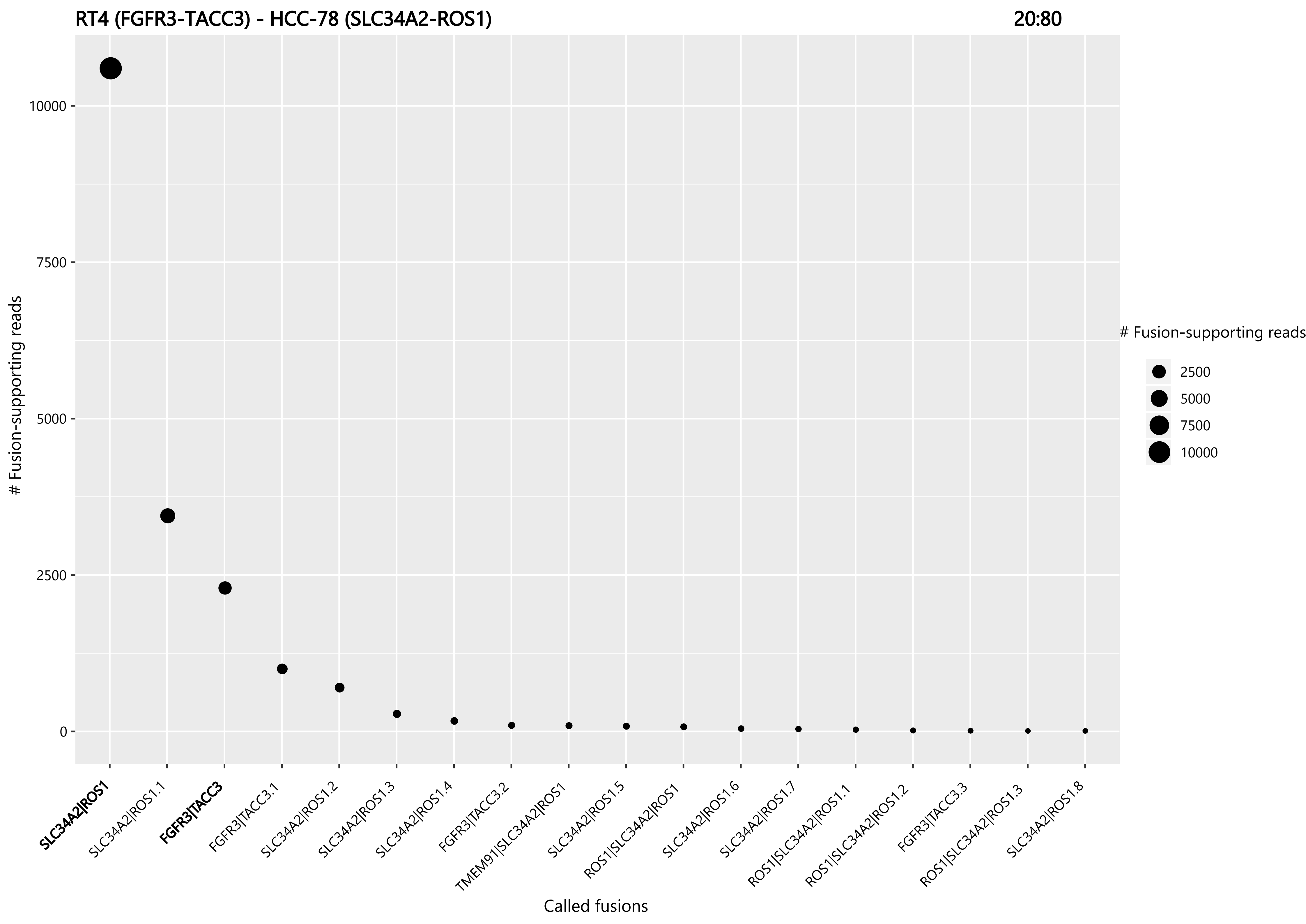

RT4 (FGFR3-TACC3) - HCC-78 (SLC34A2-ROS1)
20:80
Fusion-supporting reads
10000
7500
5000
2500
0
Fusion-supporting reads
2500
5000
7500
10000
SLC34A2|ROS1
SLC34A2|ROS1.1
FGFR3|TACC3
FGFR3|TACC3.1
SLC34A2|ROS1.2
SLC34A2|ROS1.3
SLC34A2|ROS1.4
FGFR3|TACC3.2
TMEM91|SLC34A2|ROS1
SLC34A2|ROS1.5
ROS1|SLC34A2|ROS1
SLC34A2|ROS1.6
SLC34A2|ROS1.7
ROS1|SLC34A2|ROS1.1
ROS1|SLC34A2|ROS1.2
FGFR3|TACC3.3
ROS1|SLC34A2|ROS1.3
SLC34A2|ROS1.8
Called fusions

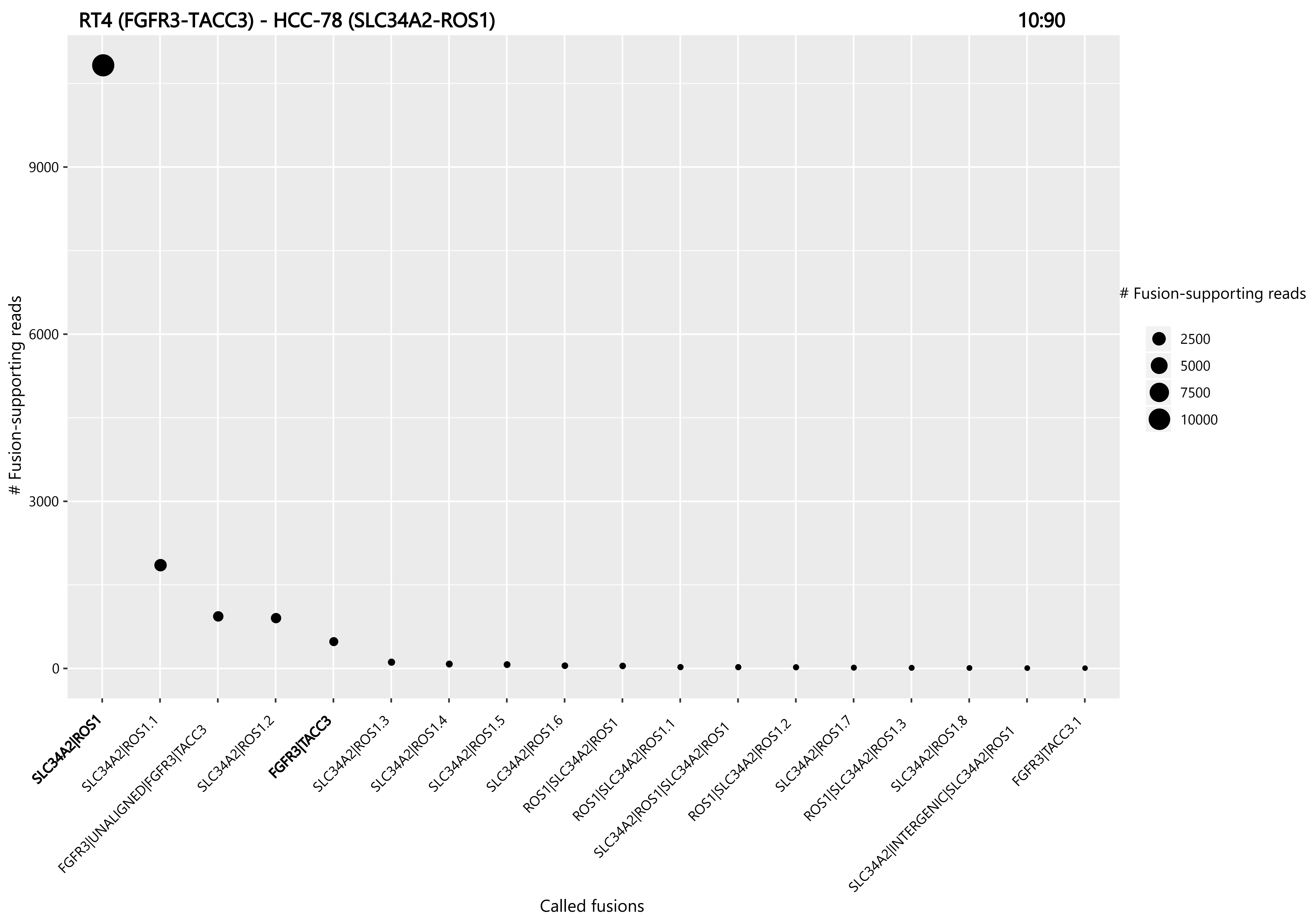
RT4 (FGFR3-TACC3) - HCC-78 (SLC34A2-ROS1)
10:90
Fusion-supporting reads
9000
6000
3000
0
Fusion-supporting reads
2500
5000
7500
10000
SLC34A2|ROS1
SLC34A2|ROS1.1
FGFR3|UNALIGNED|FGFR3|TACC3
SLC34A2|ROS1.2
FGFR3|TACC3
SLC34A2|ROS1.3
SLC34A2|ROS1.4
SLC34A2|ROS1.5
SLC34A2|ROS1.6
ROS1|SLC34A2|ROS1
ROS1|SLC34A2|ROS1.1
SLC34A2|ROS1|SLC34A2|ROS1
ROS1|SLC34A2|ROS1.2
SLC34A2|ROS1.7
ROS1|SLC34A2|ROS1.3
SLC34A2|ROS1.8
SLC34A2|INTERGENIC|SLC34A2|ROS1
FGFR3|TACC3.1
Called fusions

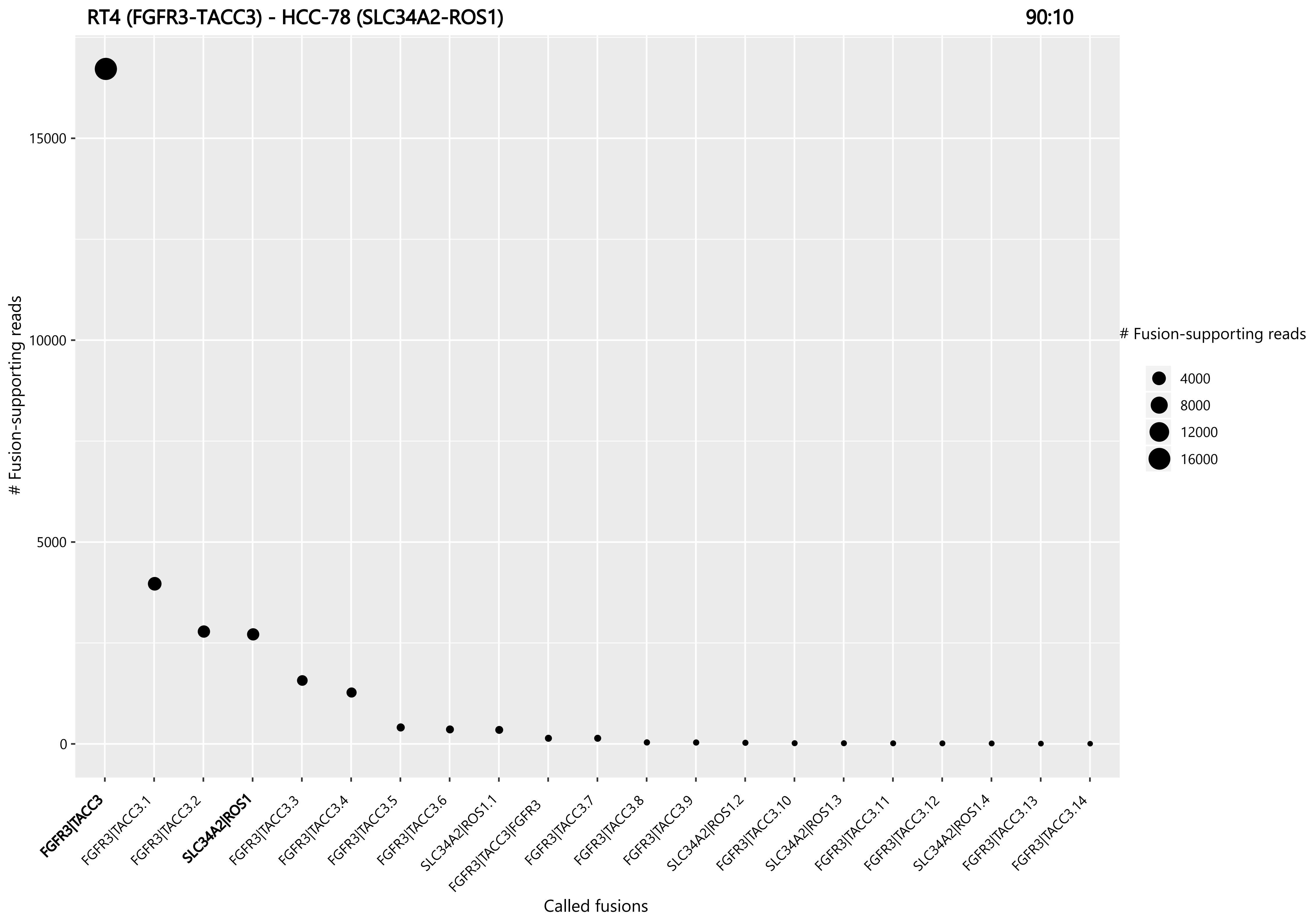

RT4 (FGFR3-TACC3) - HCC-78 (SLC34A2-ROS1)
90:10
Fusion-supporting reads
15000
10000
5000
0
Called fusions
FGFR3|TACC3
FGFR3|TACC3.1
FGFR3|TACC3.2
SLC34A2|ROS1
FGFR3|TACC3.3
FGFR3|TACC3.4
FGFR3|TACC3.5
FGFR3|TACC3.6
SLC34A2|ROS1.1
FGFR3|TACC3|FGFR3
FGFR3|TACC3.7
FGFR3|TACC3.8
FGFR3|TACC3.9
SLC34A2|ROS1.2
FGFR3|TACC3.10
SLC34A2|ROS1.3
FGFR3|TACC3.11
FGFR3|TACC3.12
SLC34A2|ROS1.4
FGFR3|TACC3.13
FGFR3|TACC3.14
Fusion-supporting reads
4000
8000
12000
16000

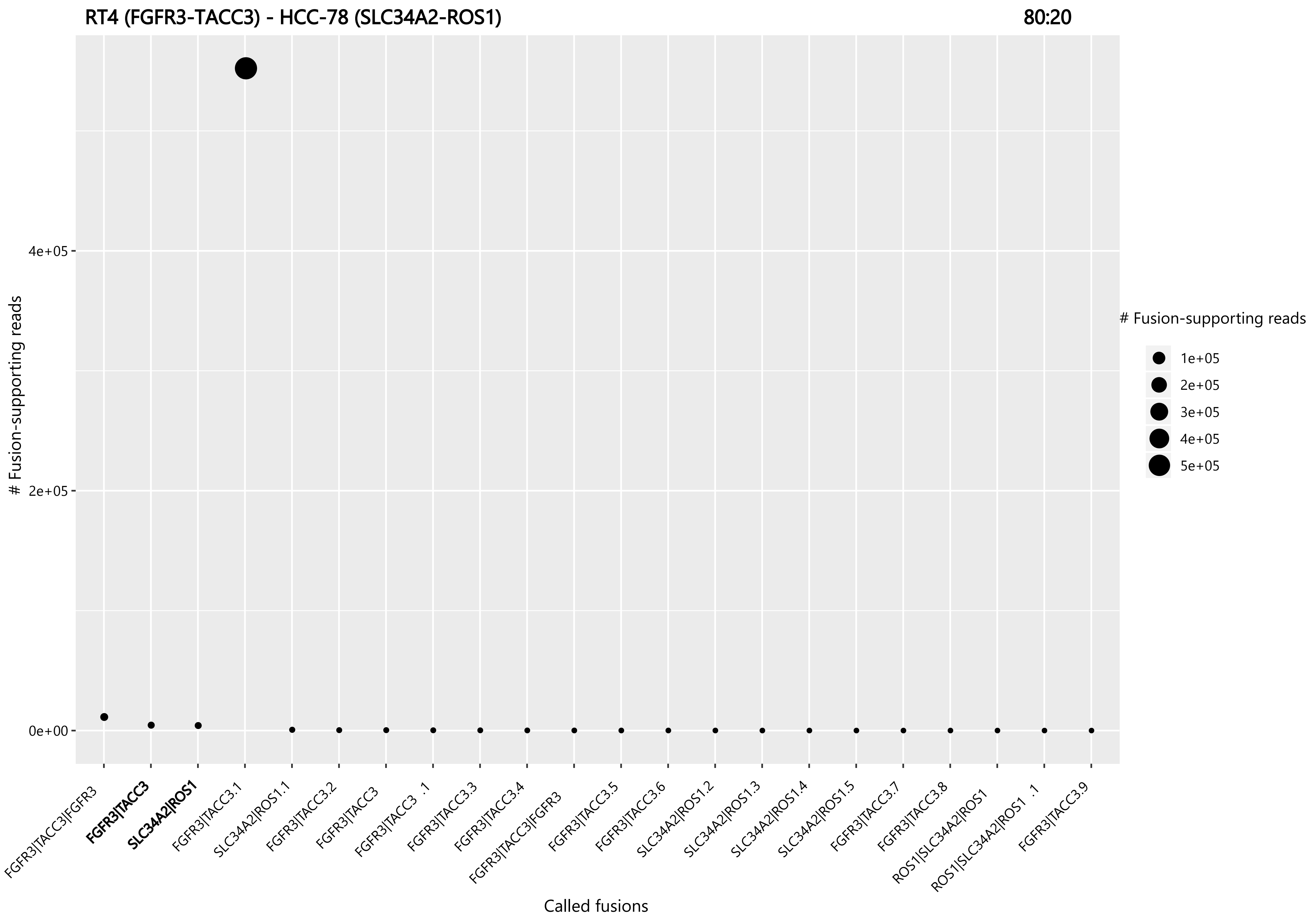

RT4 (FGFR3-TACC3) - HCC-78 (SLC34A2-ROS1)
80:20
Fusion-supporting reads
0e+00
2e+05
4e+05
Called fusions
FGFR3|TACC3|FGFR3
FGFR3|TACC3
SLC34A2|ROS1
FGFR3|TACC3.1
SLC34A2|ROS1.1
FGFR3|TACC3.2
FGFR3|TACC3
FGFR3|TACC3 .1
FGFR3|TACC3.3
FGFR3|TACC3.4
FGFR3|TACC3|FGFR3
FGFR3|TACC3.5
FGFR3|TACC3.6
SLC34A2|ROS1.2
SLC34A2|ROS1.3
SLC34A2|ROS1.4
SLC34A2|ROS1.5
FGFR3|TACC3.7
FGFR3|TACC3.8
ROS1|SLC34A2|ROS1
ROS1|SLC34A2|ROS1 .1
FGFR3|TACC3.9
Fusion-supporting reads
1e+05
2e+05
3e+05
4e+05
5e+05

SW780 (FGFR3-BAIAP2L1) - KG1 (FGFR1OP2-FGFR1) 50:50

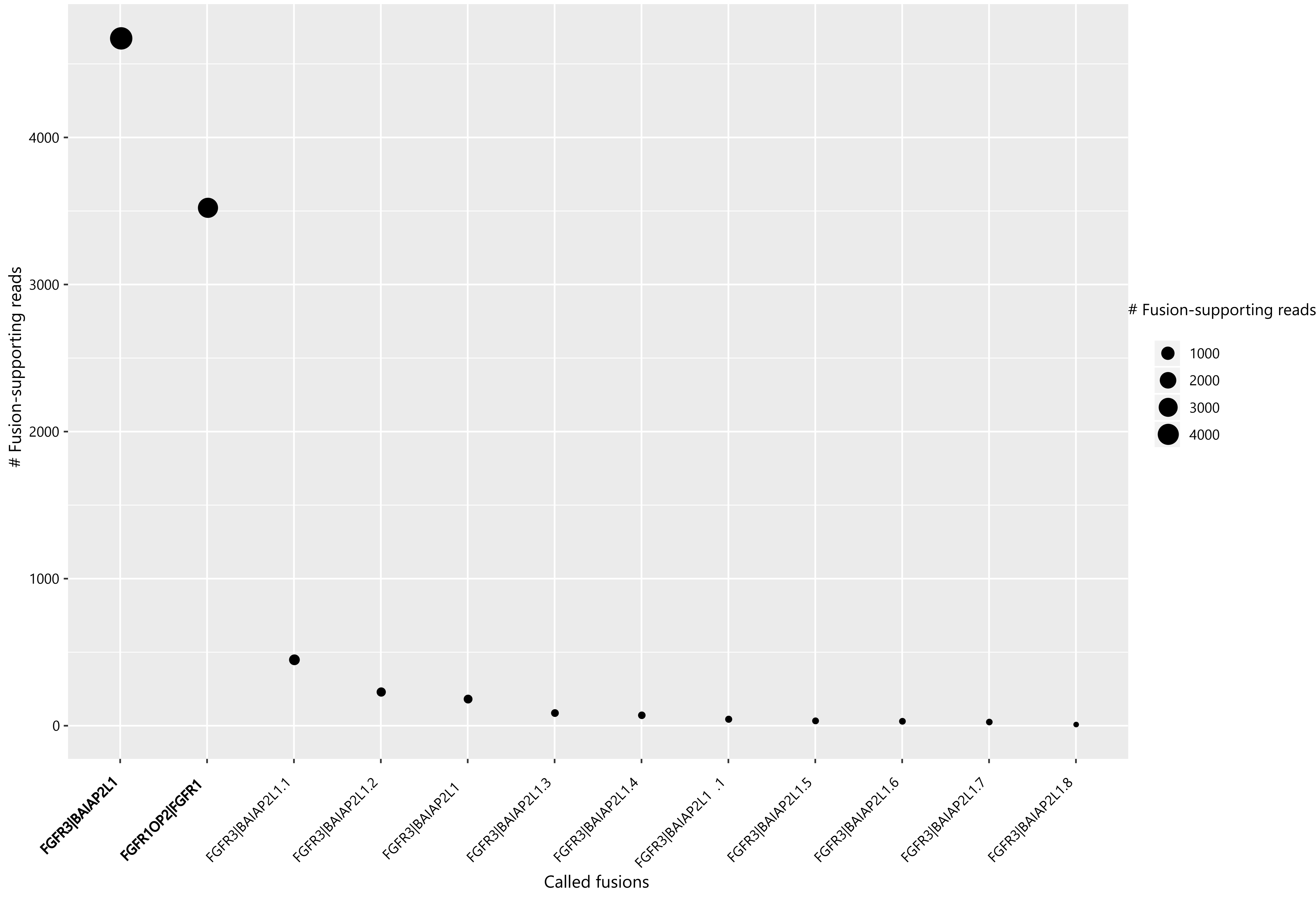

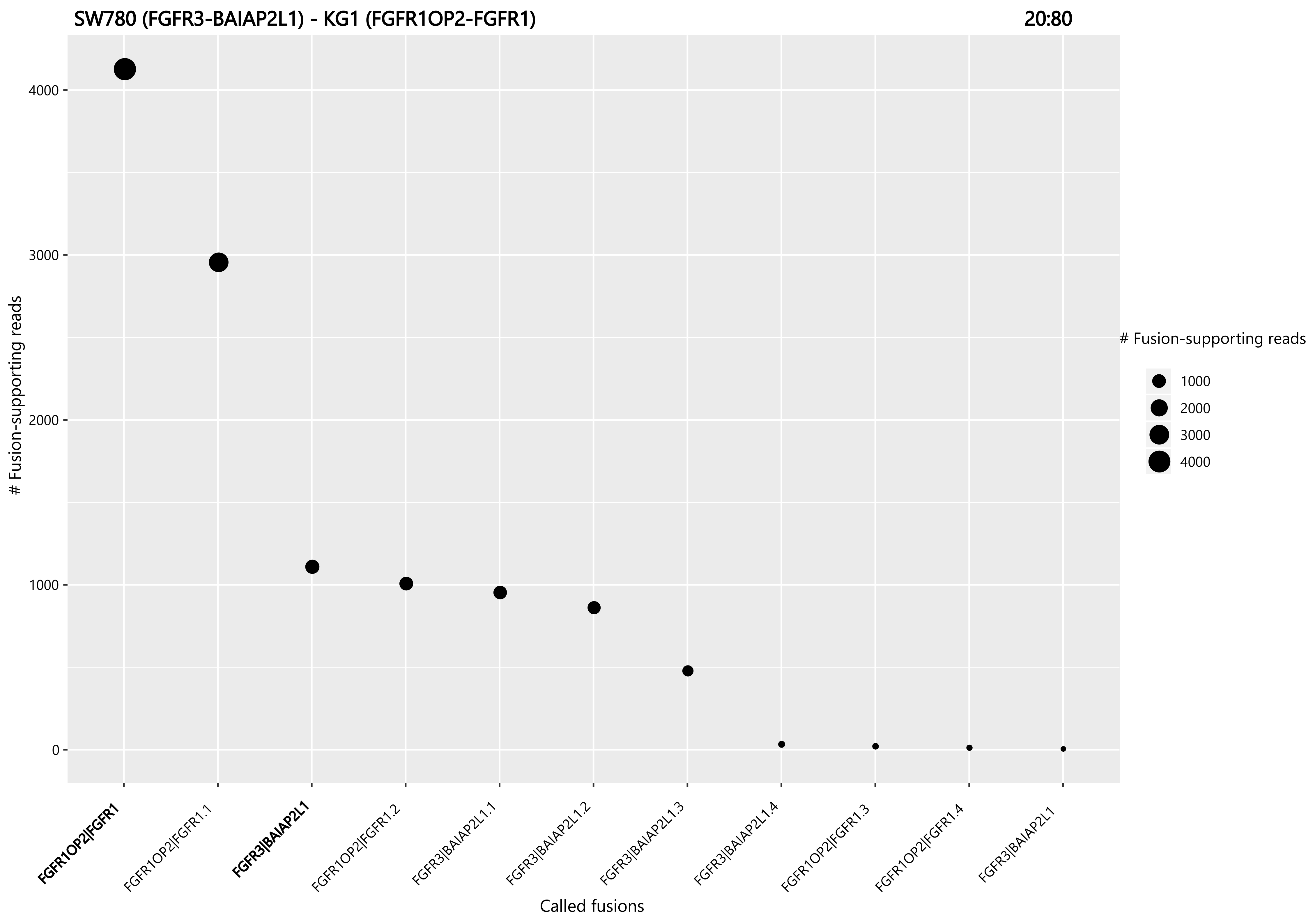
SW780 (FGFR3-BAIAP2L1) - KG1 (FGFR1OP2-FGFR1)
20:80
Fusion-supporting reads
4000
3000
2000
1000
0
Called fusions
FGFR1OP2|FGFR1
FGFR1OP2|FGFR1.1
FGFR3|BAIAP2L1
FGFR1OP2|FGFR1.2
FGFR3|BAIAP2L1.1
FGFR3|BAIAP2L1.2
FGFR3|BAIAP2L1.3
FGFR3|BAIAP2L1.4
FGFR1OP2|FGFR1.3
FGFR1OP2|FGFR1.4
FGFR3|BAIAP2L1
Fusion-supporting reads
1000
2000
3000
4000

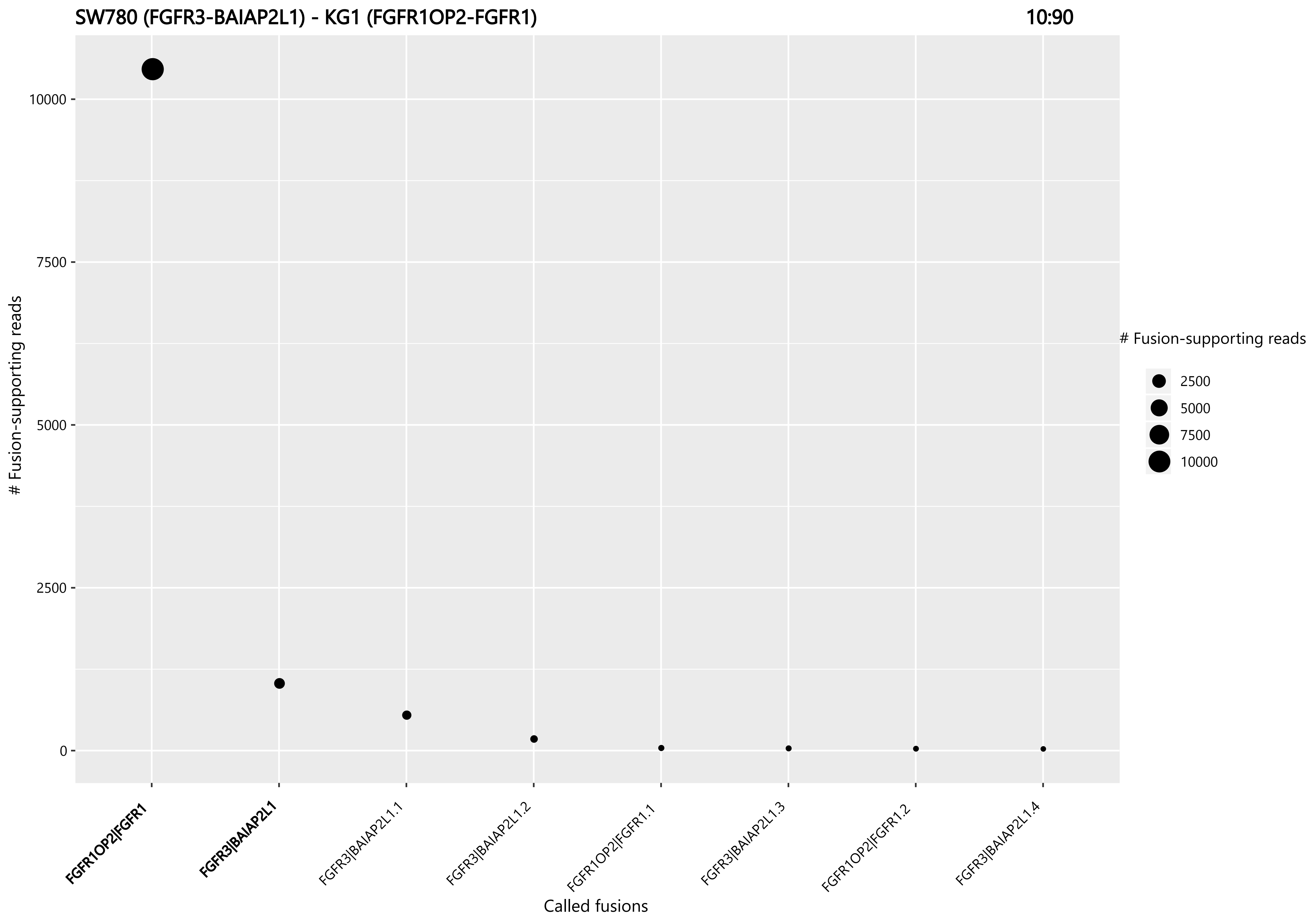
SW780 (FGFR3-BAIAP2L1) - KG1 (FGFR1OP2-FGFR1)
10:90
Fusion-supporting reads
10000
7500
5000
2500
0
FGFR1OP2|FGFR1
FGFR3|BAIAP2L1
FGFR3|BAIAP2L1.1
FGFR3|BAIAP2L1.2
FGFR1OP2|FGFR1.1
FGFR3|BAIAP2L1.3
FGFR1OP2|FGFR1.2
FGFR3|BAIAP2L1.4
Called fusions
Fusion-supporting reads
2500
5000
7500
10000

SW780 (FGFR3-BAIAP2L1) - KG1 (FGFR1OP2-FGFR1) 90:10

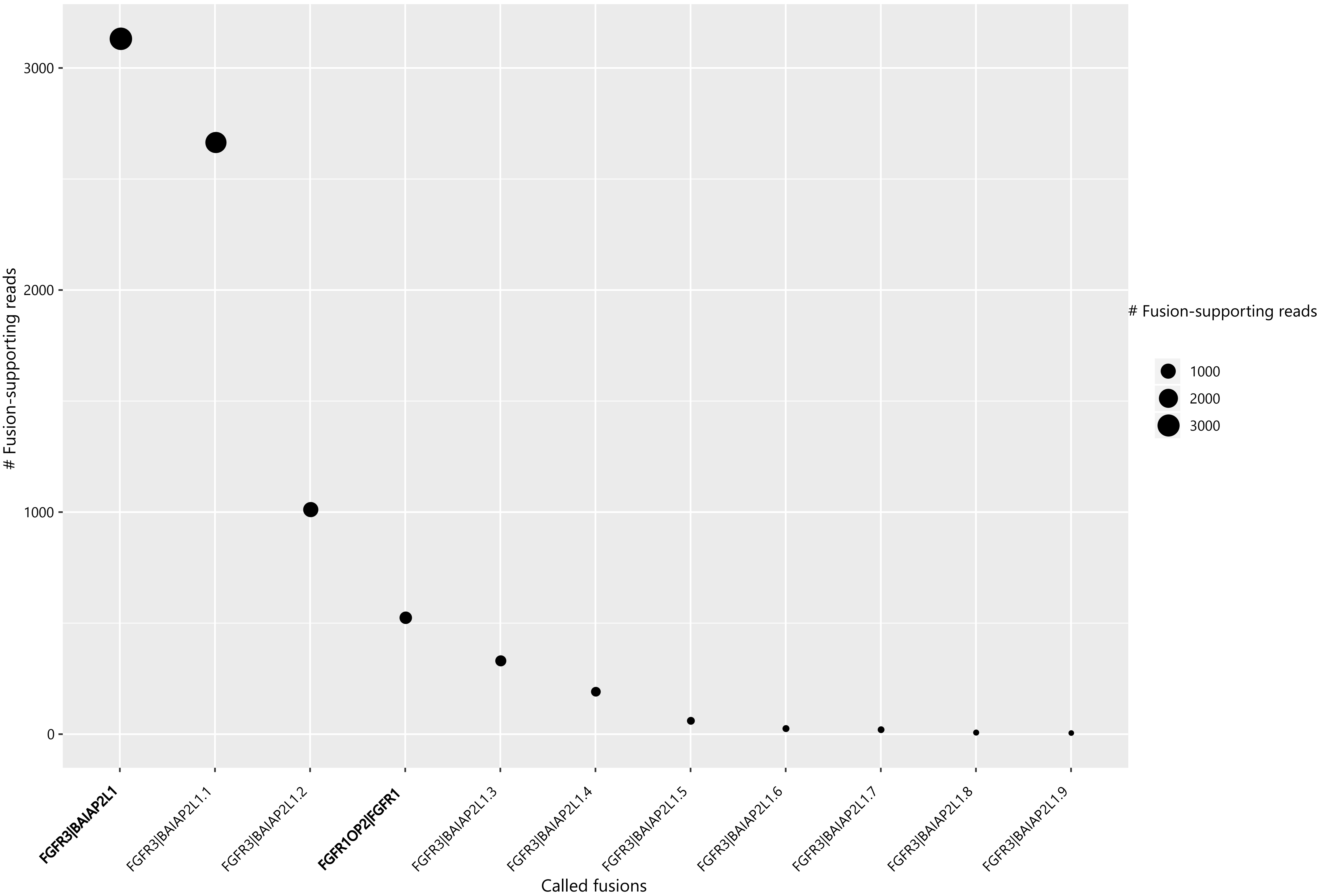

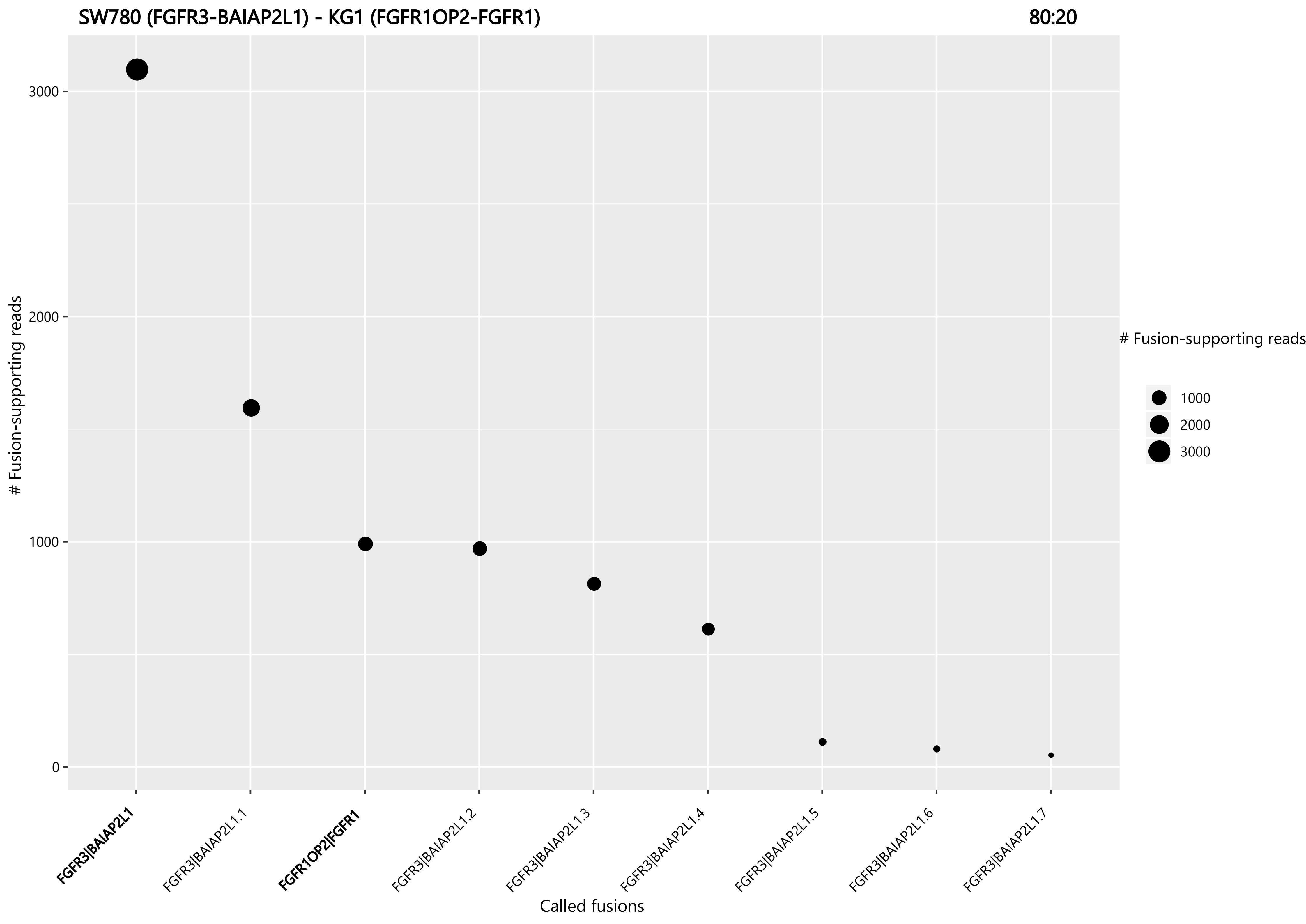

SW780 (FGFR3-BAIAP2L1) - KG1 (FGFR1OP2-FGFR1)
80:20
Fusion-supporting reads
3000
2000
1000
0
FGFR3|BAIAP2L1
FGFR3|BAIAP2L1.1
FGFR1OP2|FGFR1
FGFR3|BAIAP2L1.2
FGFR3|BAIAP2L1.3
FGFR3|BAIAP2L1.4
FGFR3|BAIAP2L1.5
FGFR3|BAIAP2L1.6
FGFR3|BAIAP2L1.7
Called fusions
Fusion-supporting reads
1000
2000
3000

KIA1549-BRAF

Sample 1

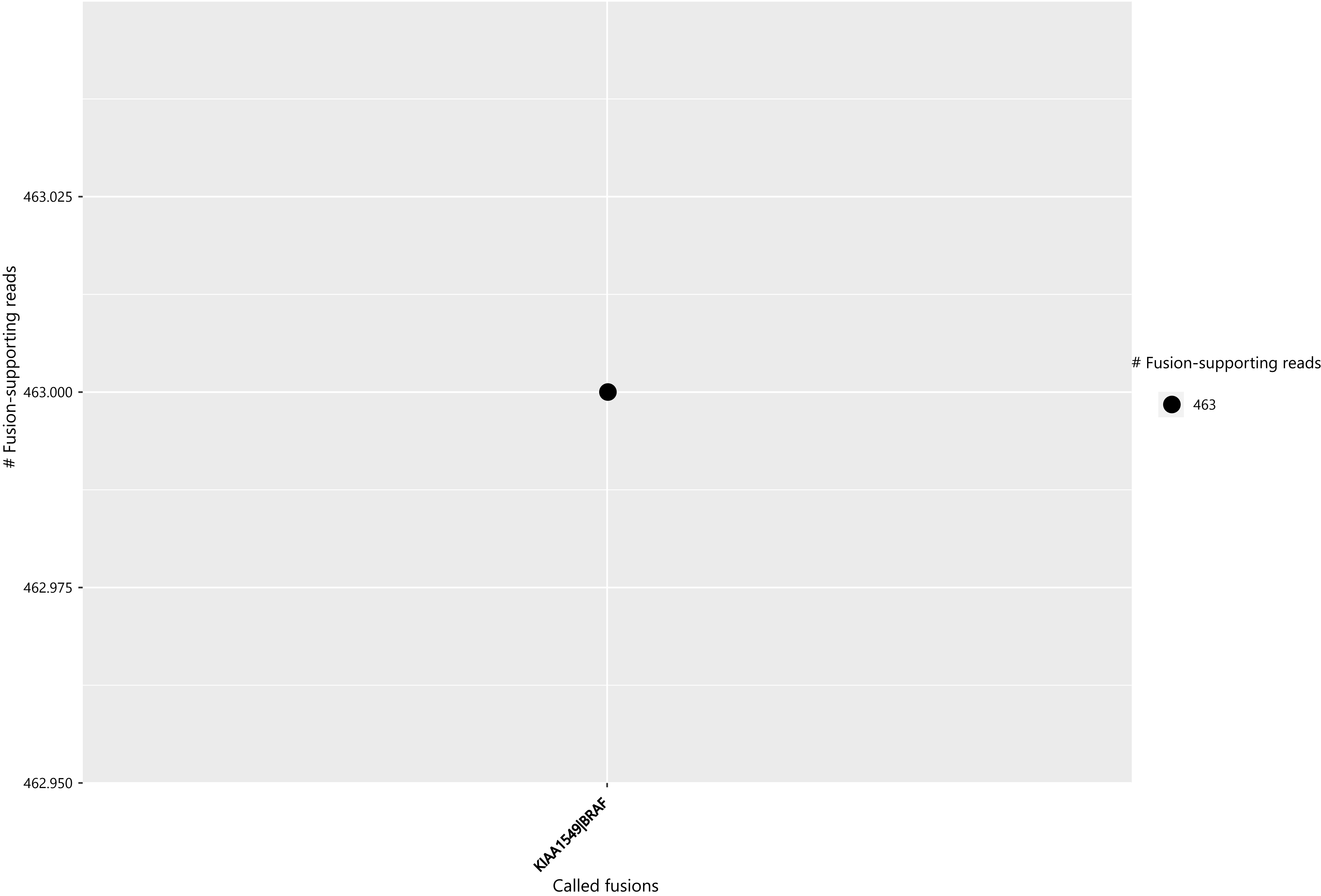

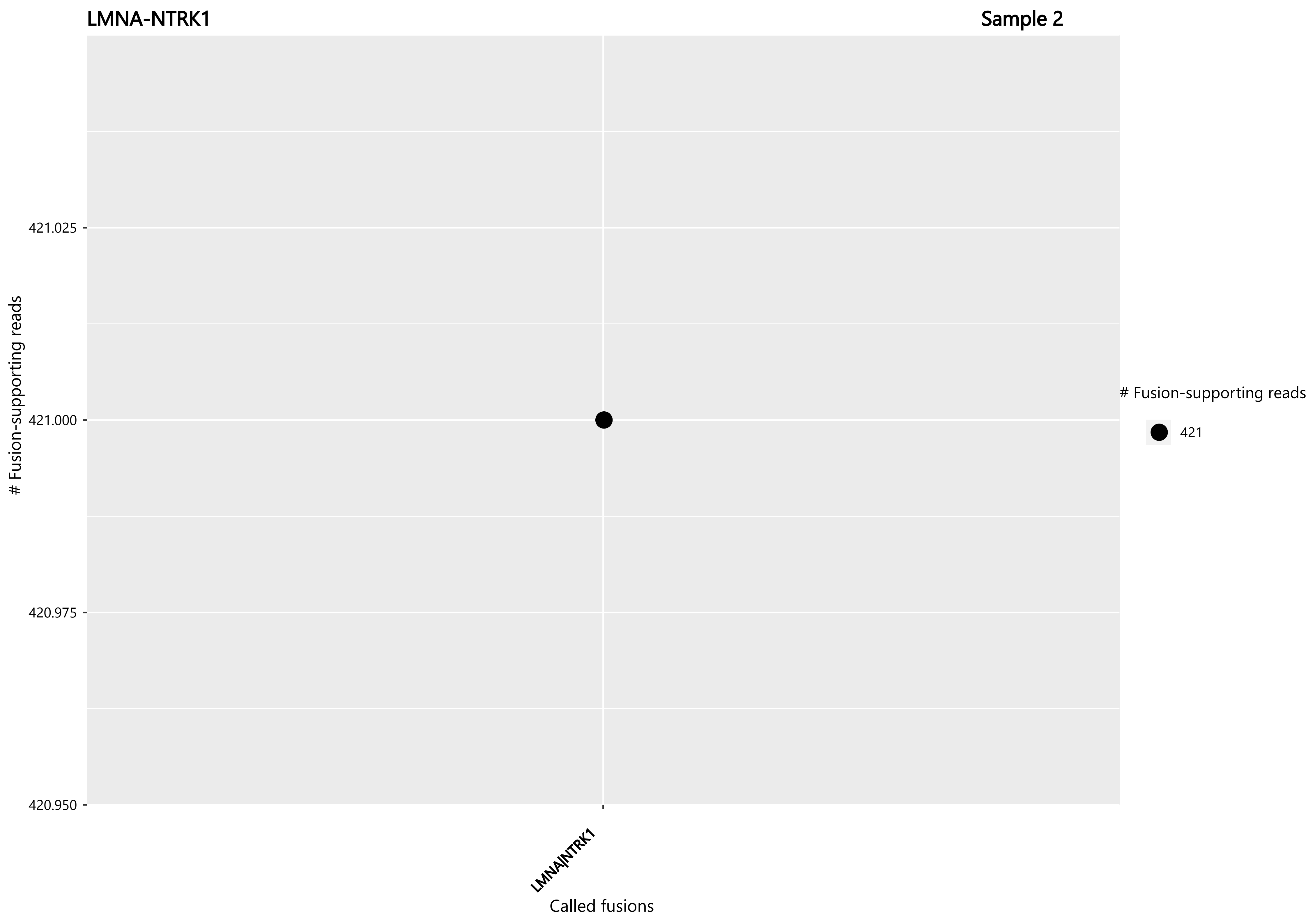
LMNA-NTRK1
Sample 2
421.025
421.000
420.975
420.950
Fusion-supporting reads
LMNA|NTRK1
Called fusions
Fusion-supporting reads
421

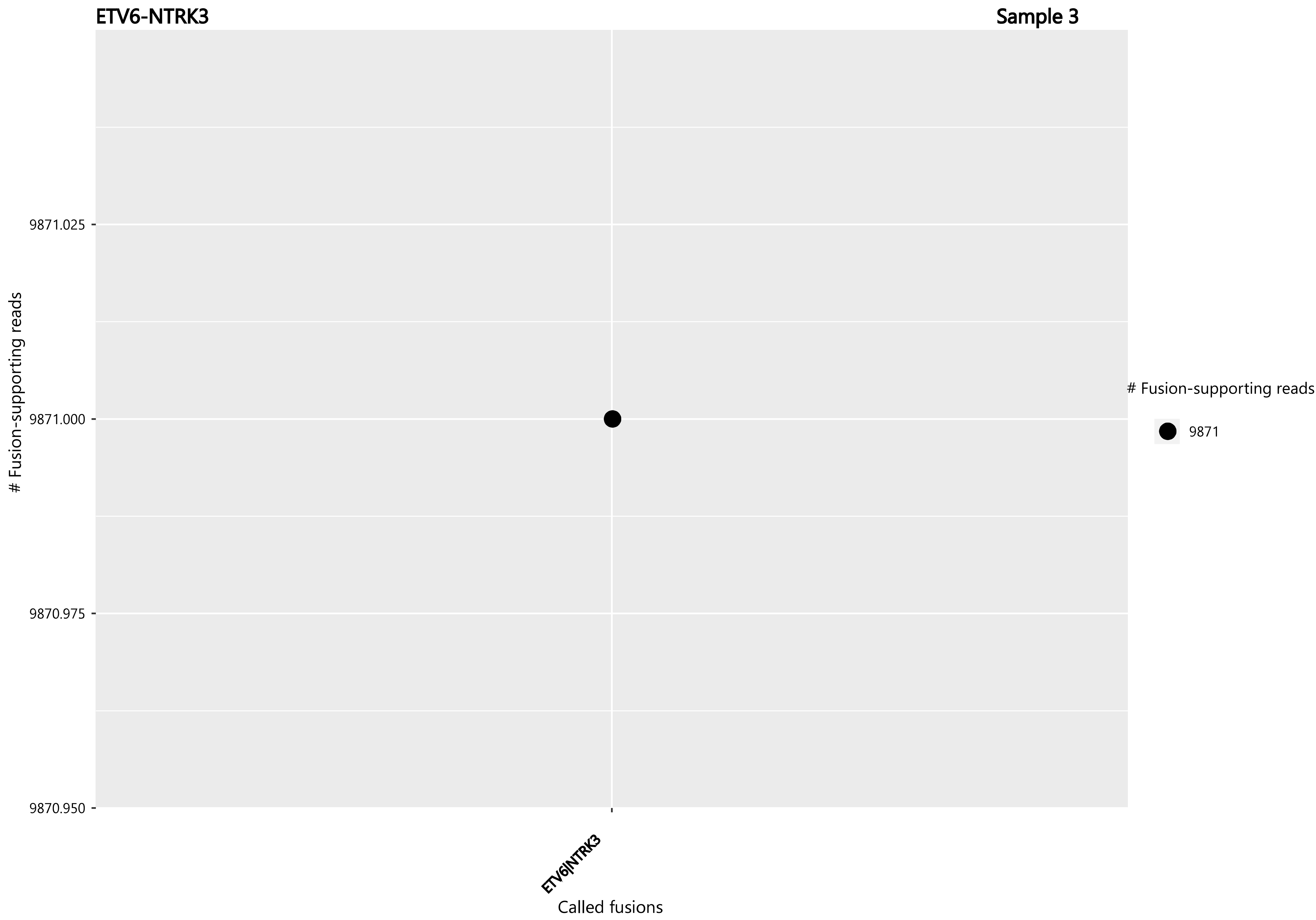

ETV6-NTRK3
Sample 3
Fusion-supporting reads
9871.025
9871.000
9870.975
9870.950
ETV6|NTRK3
Called fusions
Fusion-supporting reads
9871

HLA-DRB1-MET

Sample 4

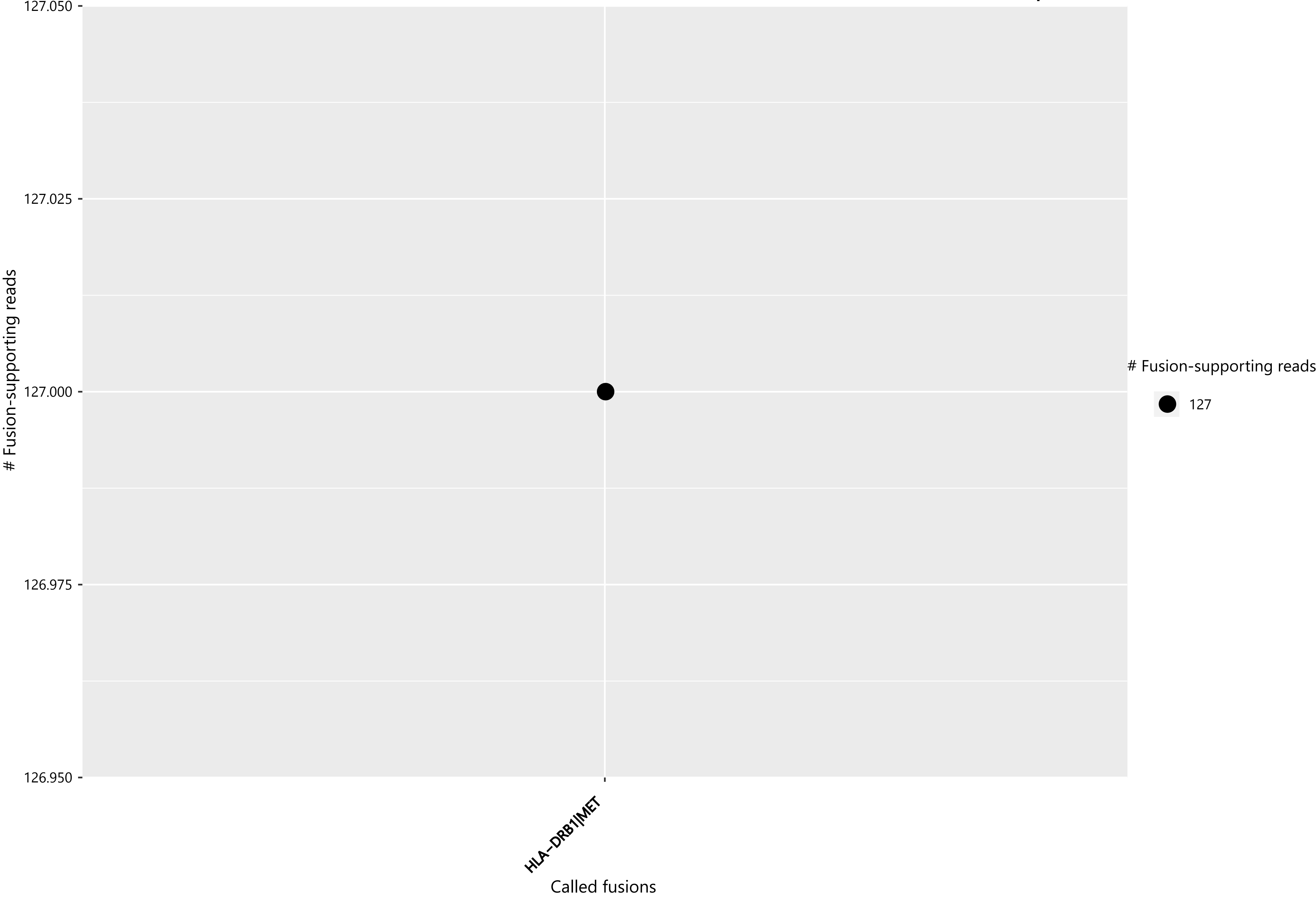

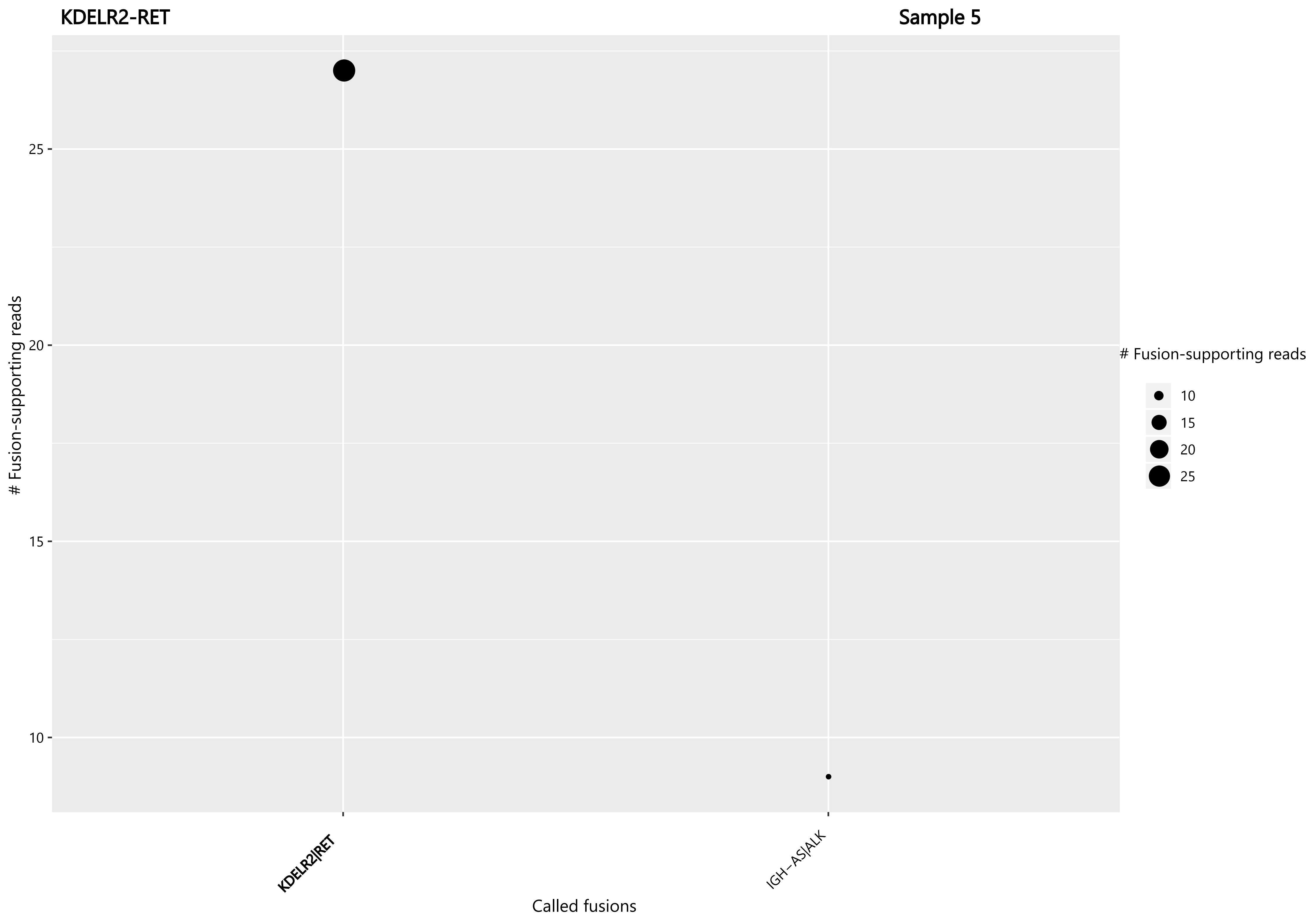
KDELR2-RET
Sample 5
Fusion-supporting reads
25
20
15
10
Fusion-supporting reads
10
15
20
25
KDELR2|RET
IGH−AS|ALK
Called fusions

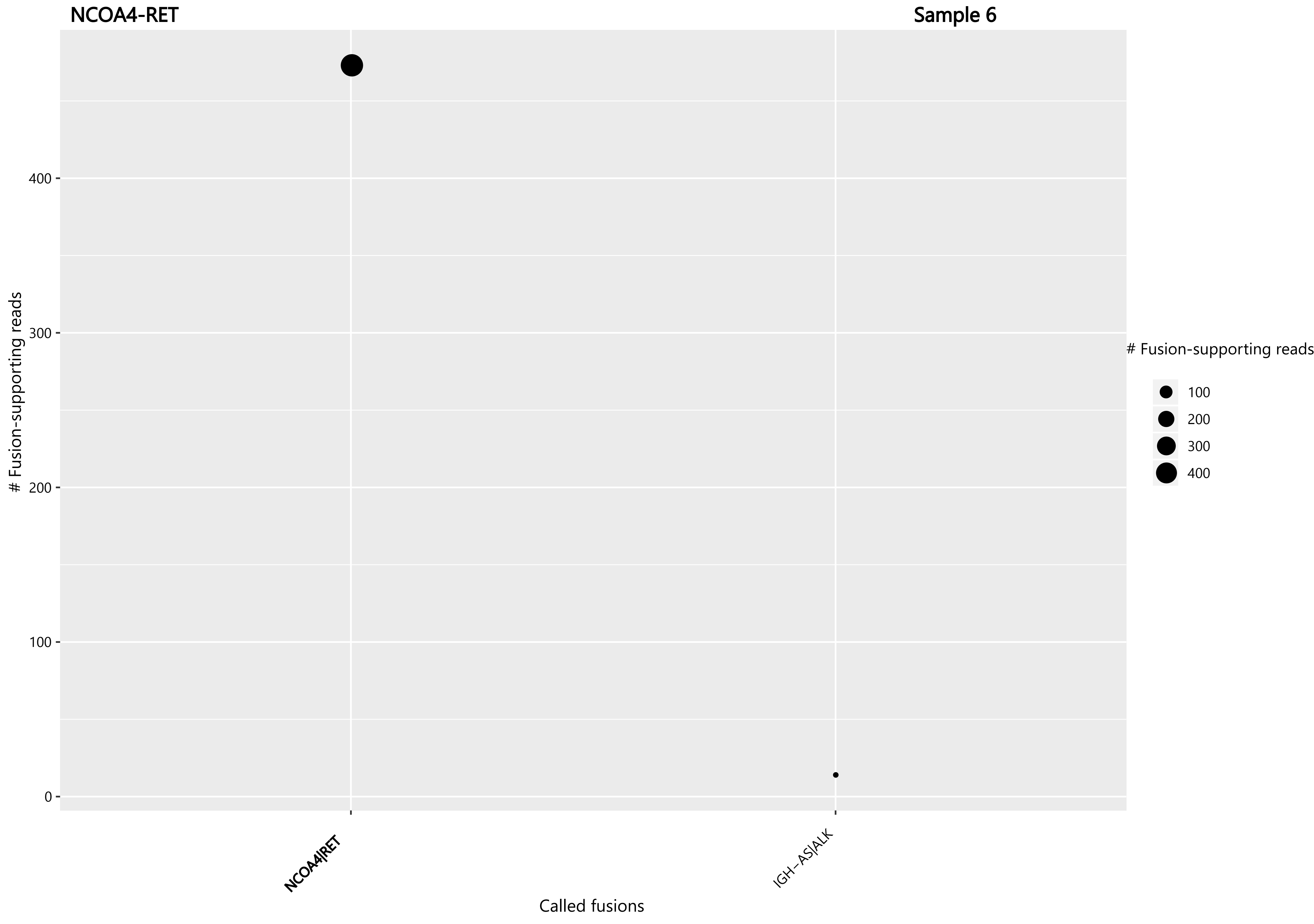
NCOA4-RET
Sample 6
Fusion-supporting reads
400
300
200
100
0
NCOA4|RET
IGH−AS|ALK
Called fusions
Fusion-supporting reads
100
200
300
400

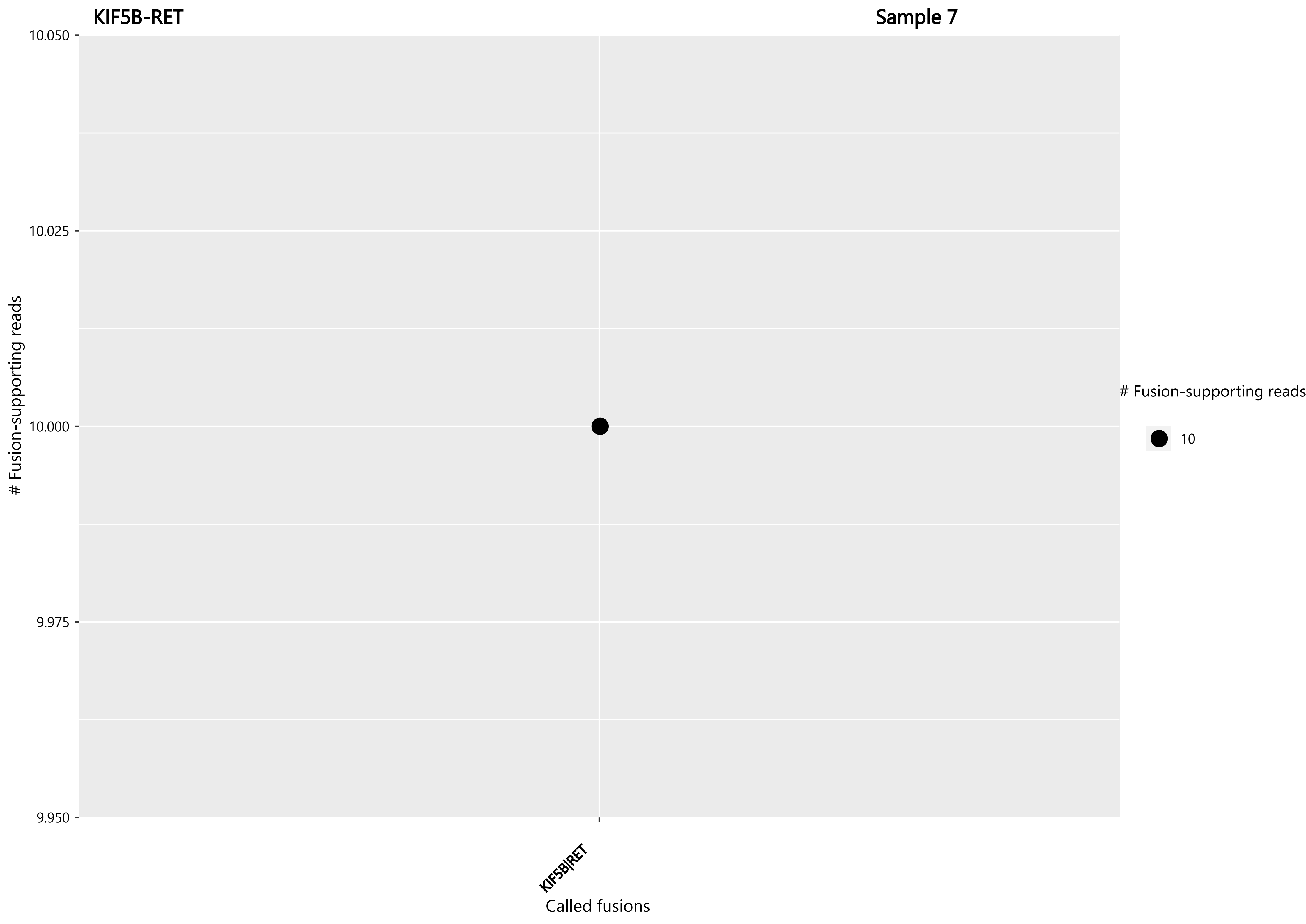

KIF5B-RET
Sample 7
10.050
10.025
10.000
9.975
9.950
Fusion-supporting reads
KIF5B|RET
Called fusions
Fusion-supporting reads
10

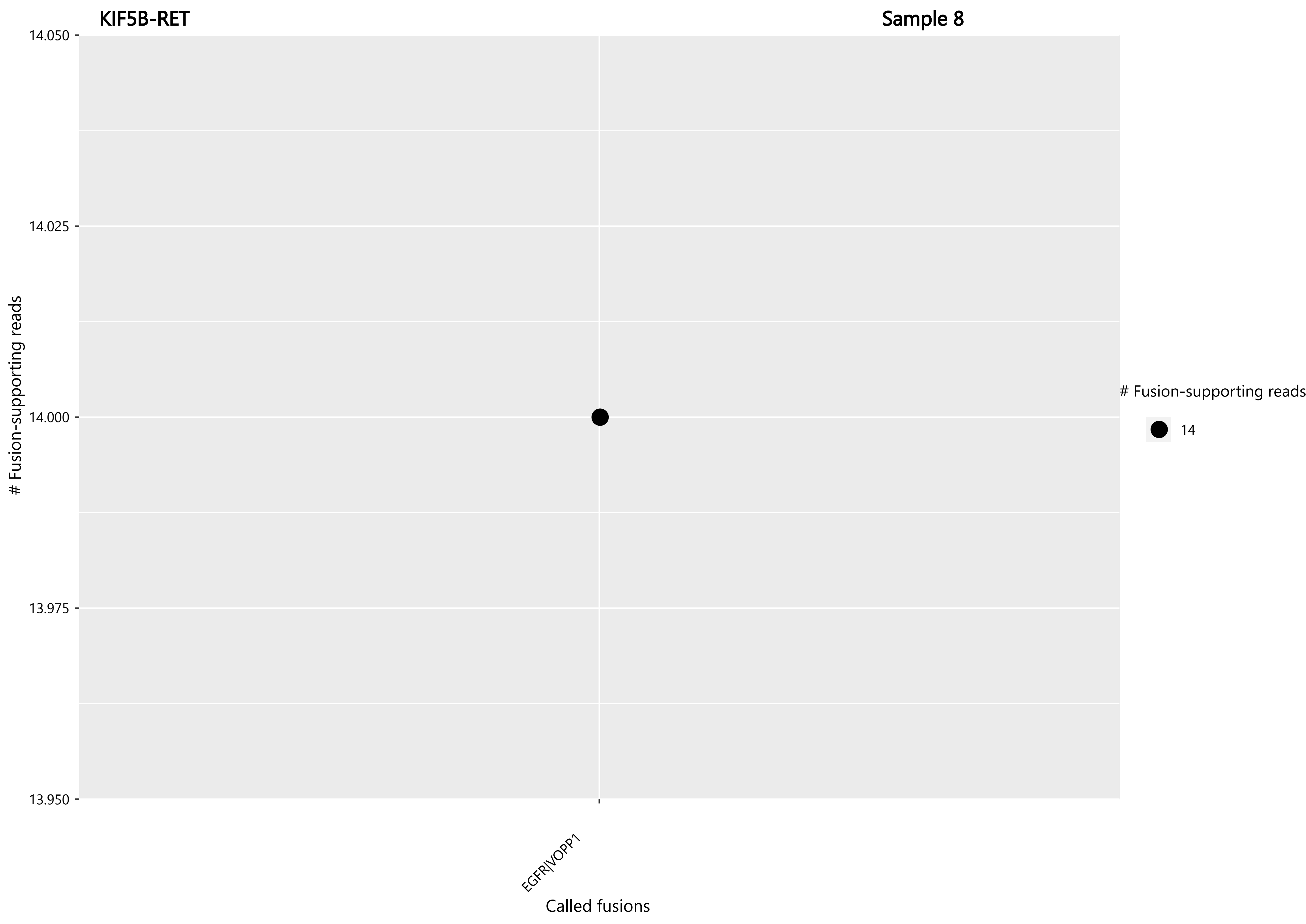
KIF5B-RET
Sample 8
14.050
14.025
14.000
13.975
13.950
Fusion-supporting reads
EGFR|VOPP1
Called fusions
Fusion-supporting reads
14

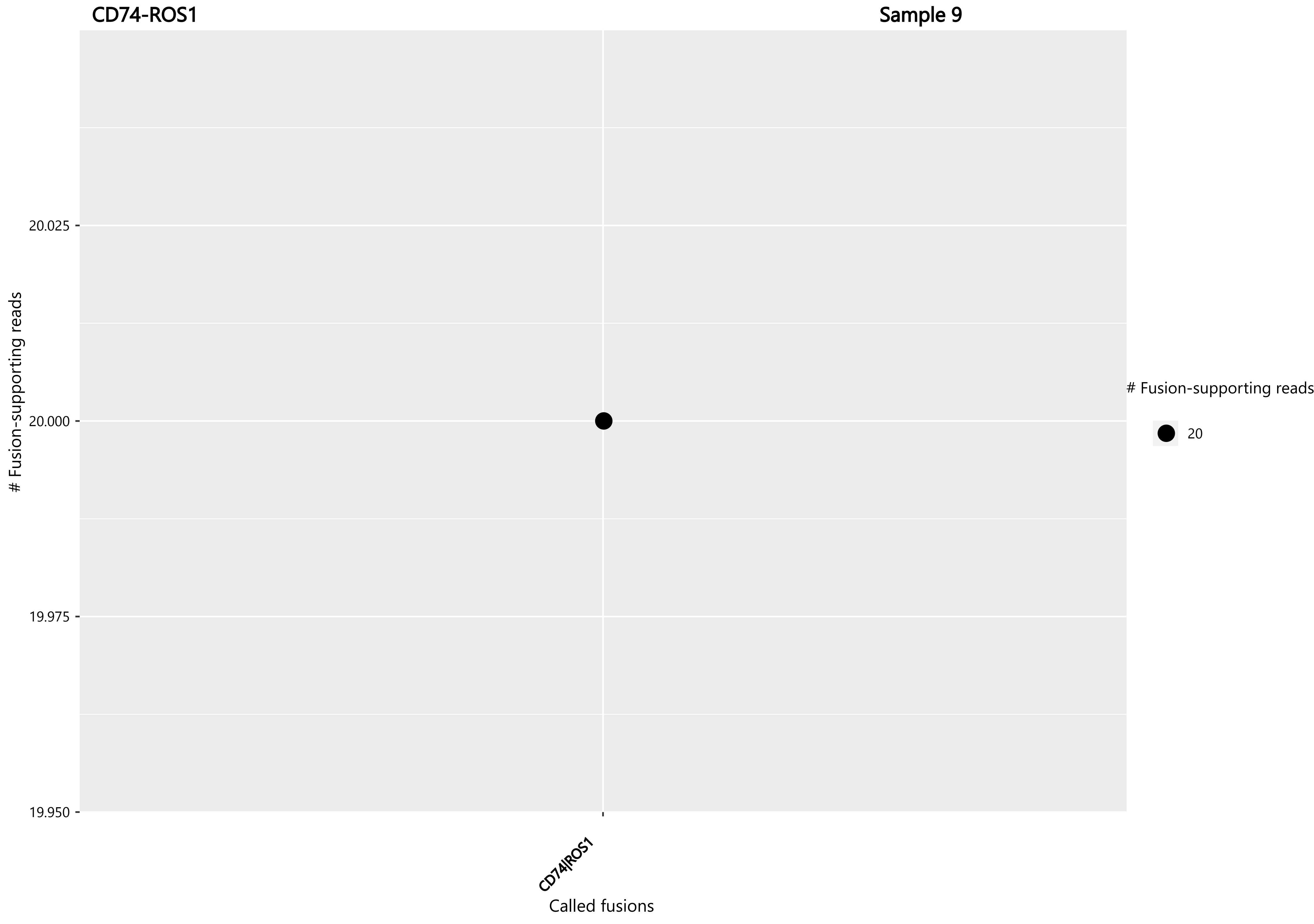
CD74-ROS1
Sample 9
Fusion-supporting reads
20.025
20.000
19.975
19.950
CD74|ROS1
Called fusions
Fusion-supporting reads
20

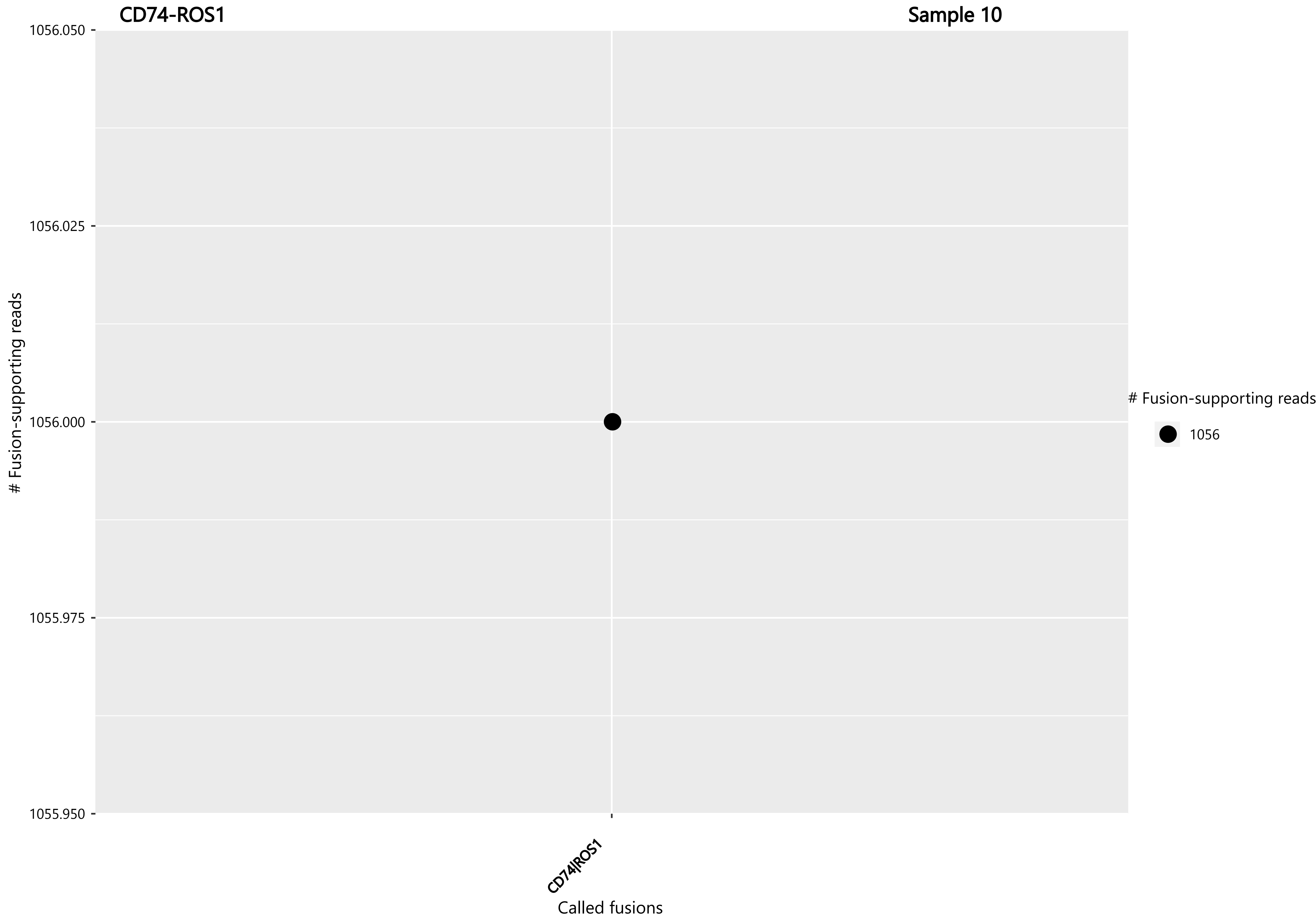

CD74-ROS1
Sample 10
1056.050
1056.025
1056.000
1055.975
1055.950
Fusion-supporting reads
CD74|ROS1
Called fusions
Fusion-supporting reads
1056

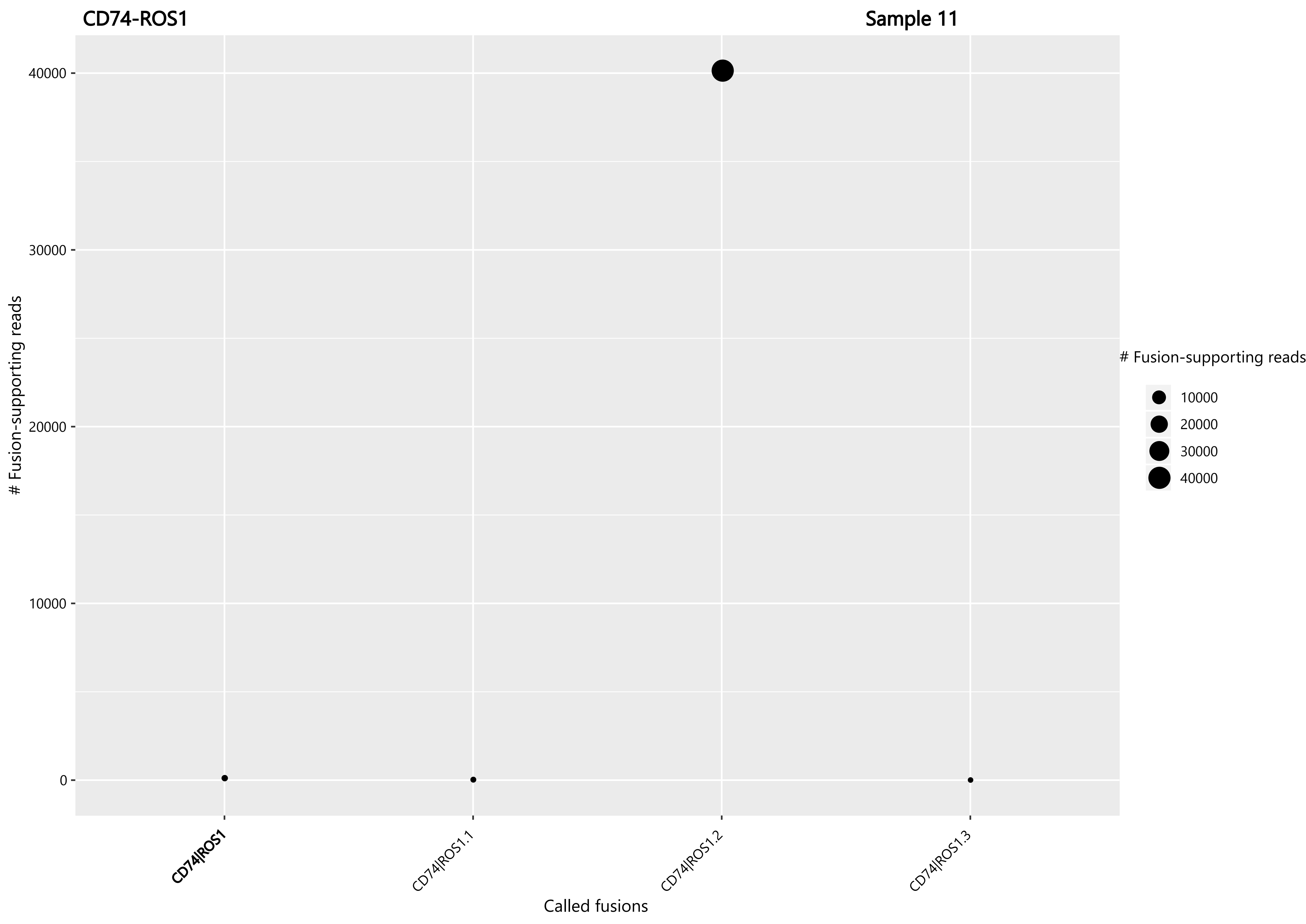
CD74-ROS1
Sample 11
Fusion-supporting reads
40000
30000
20000
10000
0
Fusion-supporting reads
10000
20000
30000
40000
CD74|ROS1
CD74|ROS1.1
CD74|ROS1.2
CD74|ROS1.3
Called fusions

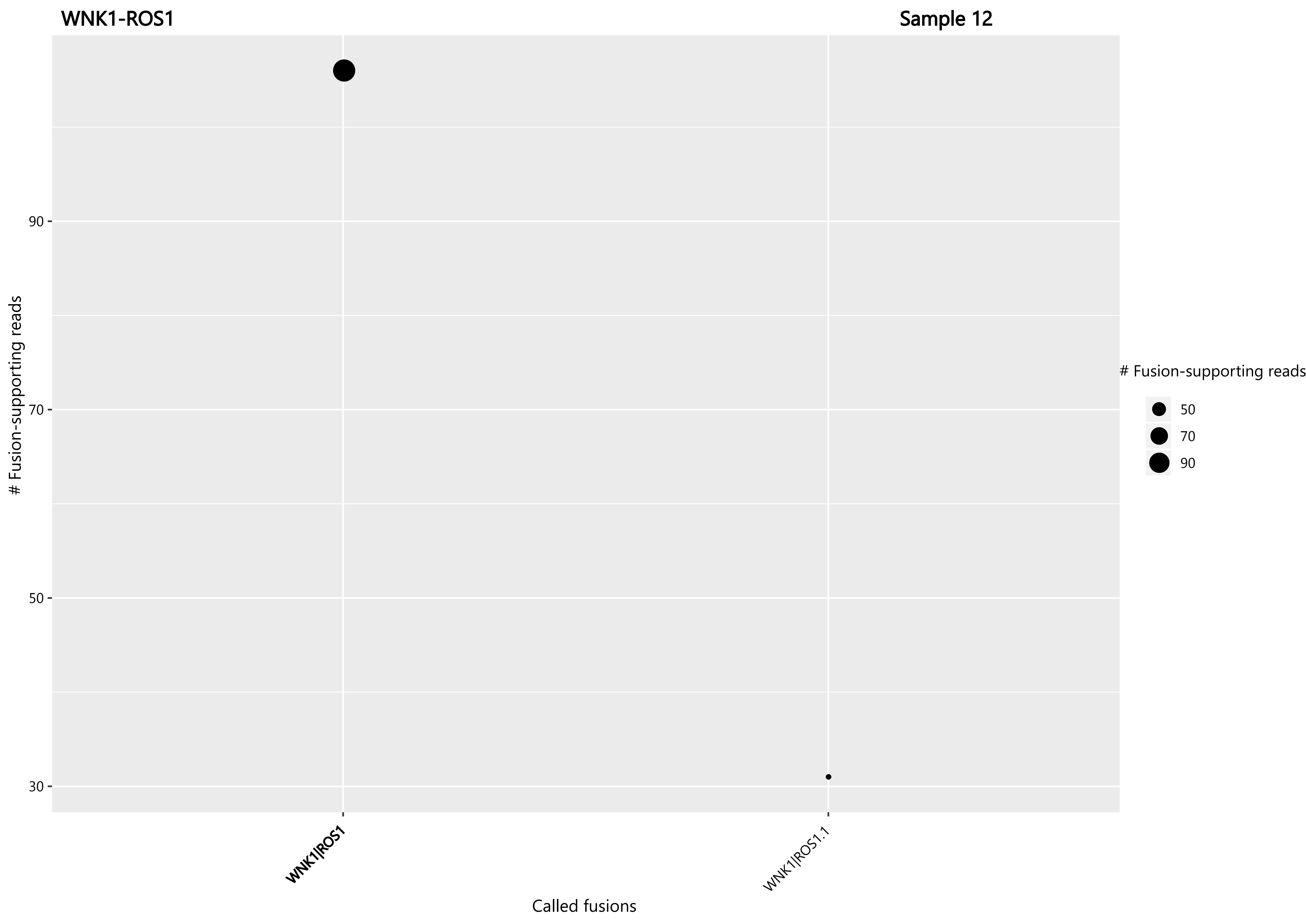
WNK1-ROS1
Sample 12
Fusion-supporting reads
90
70
50
30
WNK1|ROS1
WNK1|ROS1.1
Called fusions
Fusion-supporting reads
50
70
90

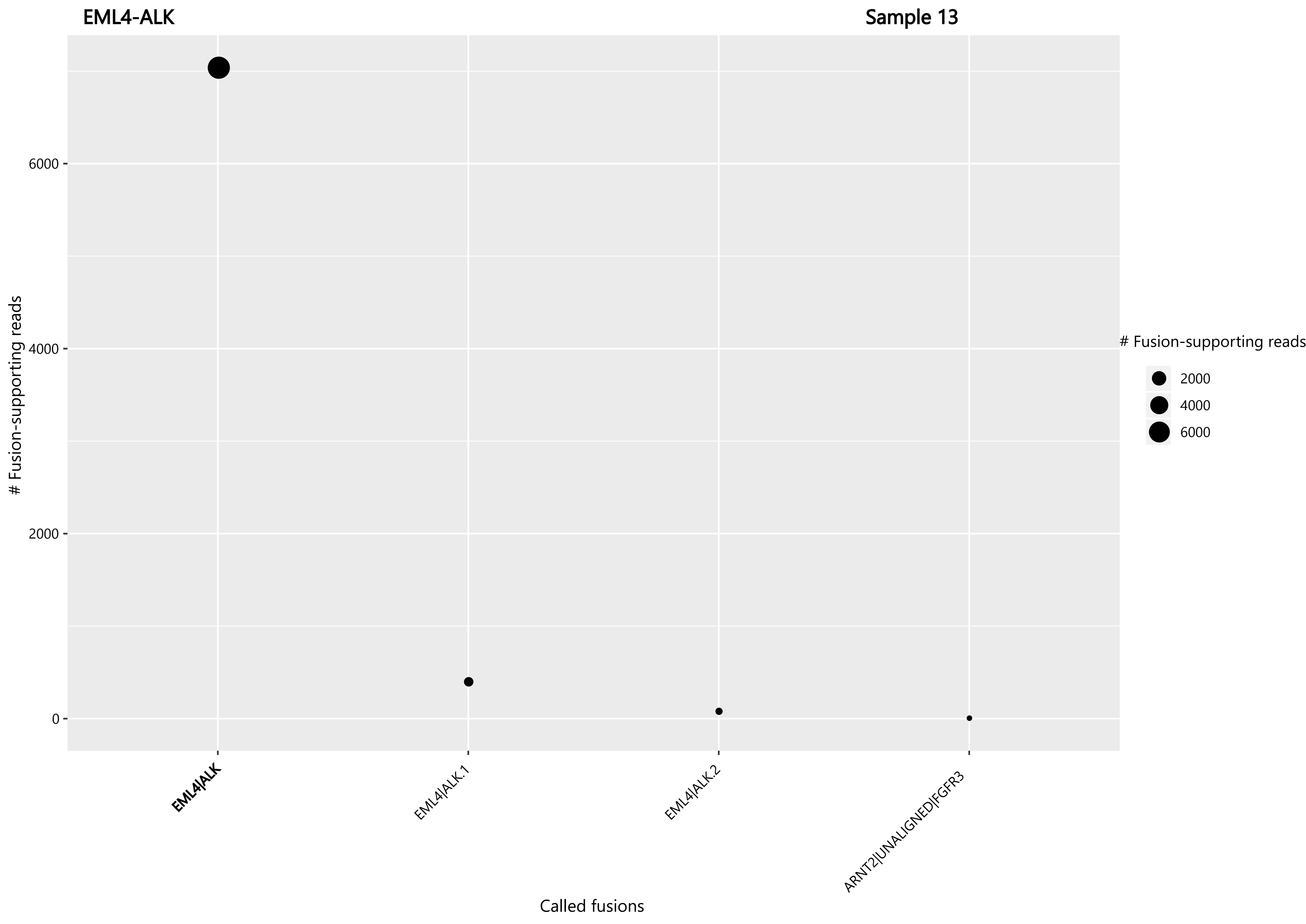

EML4-ALK
Sample 13
Fusion-supporting reads
6000
4000
2000
0
Fusion-supporting reads
2000
4000
6000
EML4|ALK
EML4|ALK.1
EML4|ALK.2
ARNT2|UNALIGNED|FGFR3
Called fusions

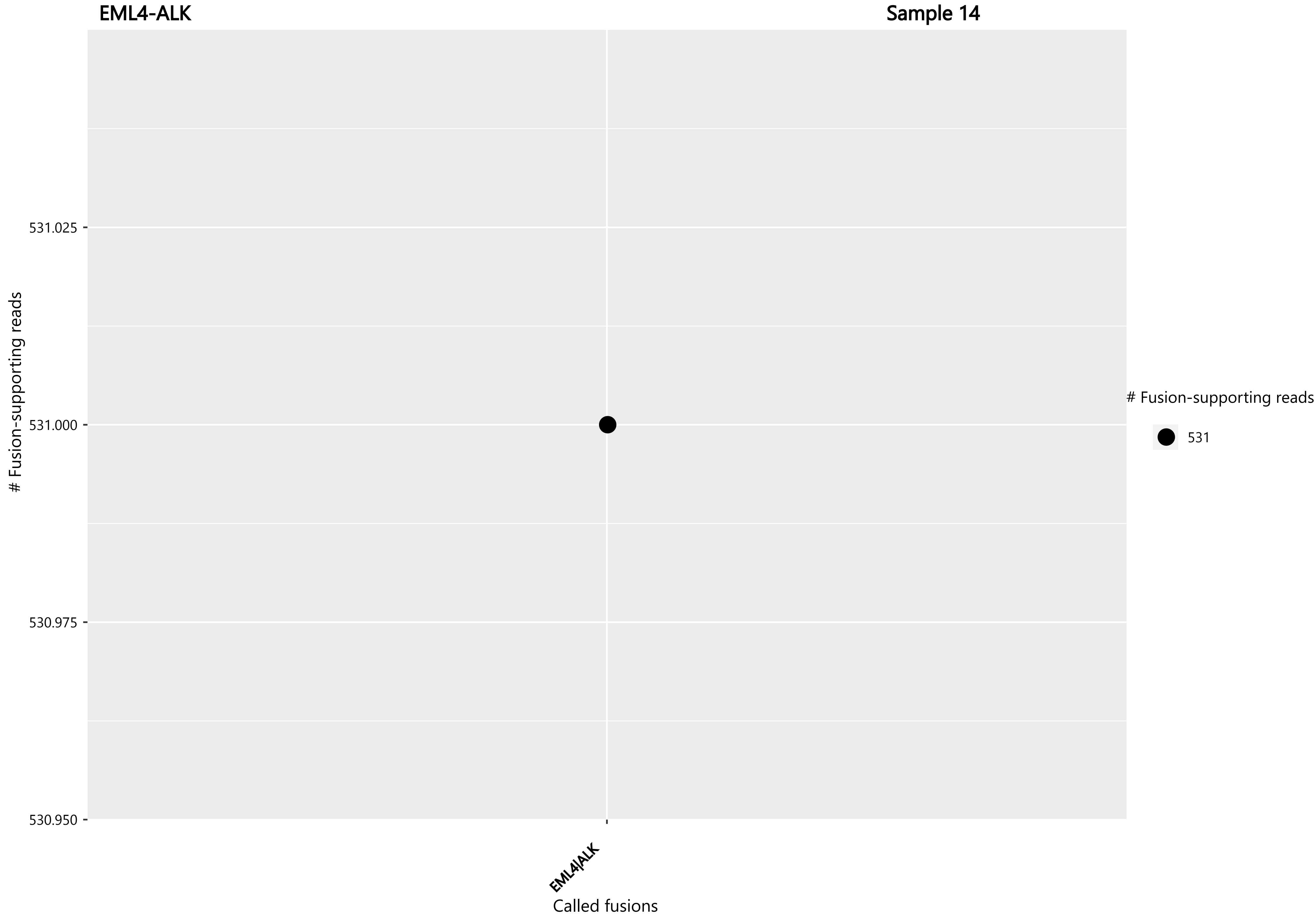

EML4-ALK
Sample 14
531.025
531.000
530.975
530.950
Fusion-supporting reads
EML4|ALK
Called fusions
Fusion-supporting reads
531

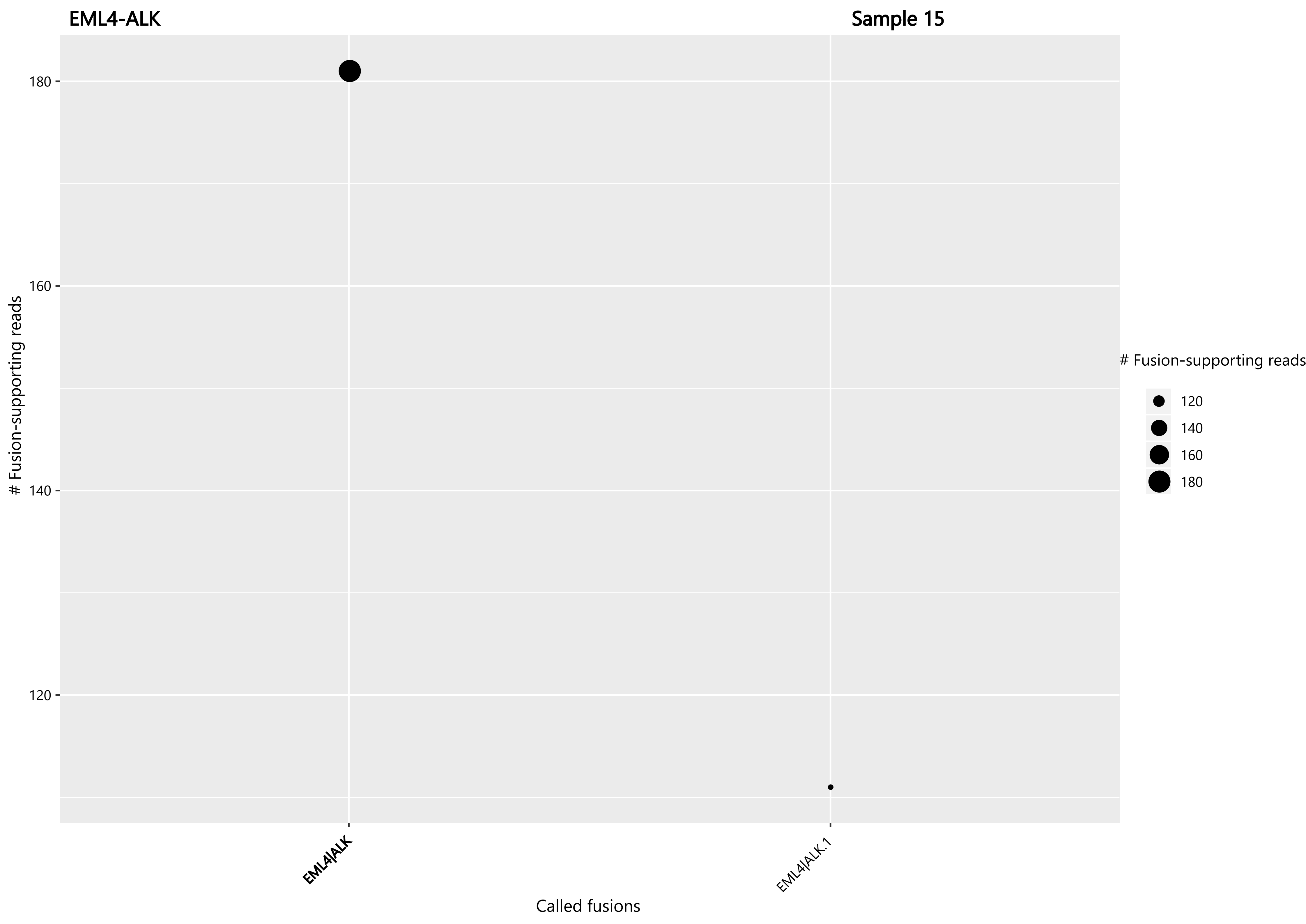
EML4-ALK
Sample 15
Fusion-supporting reads
180
160
140
120
Called fusions
EML4|ALK
EML4|ALK.1
Fusion-supporting reads
120
140
160
180

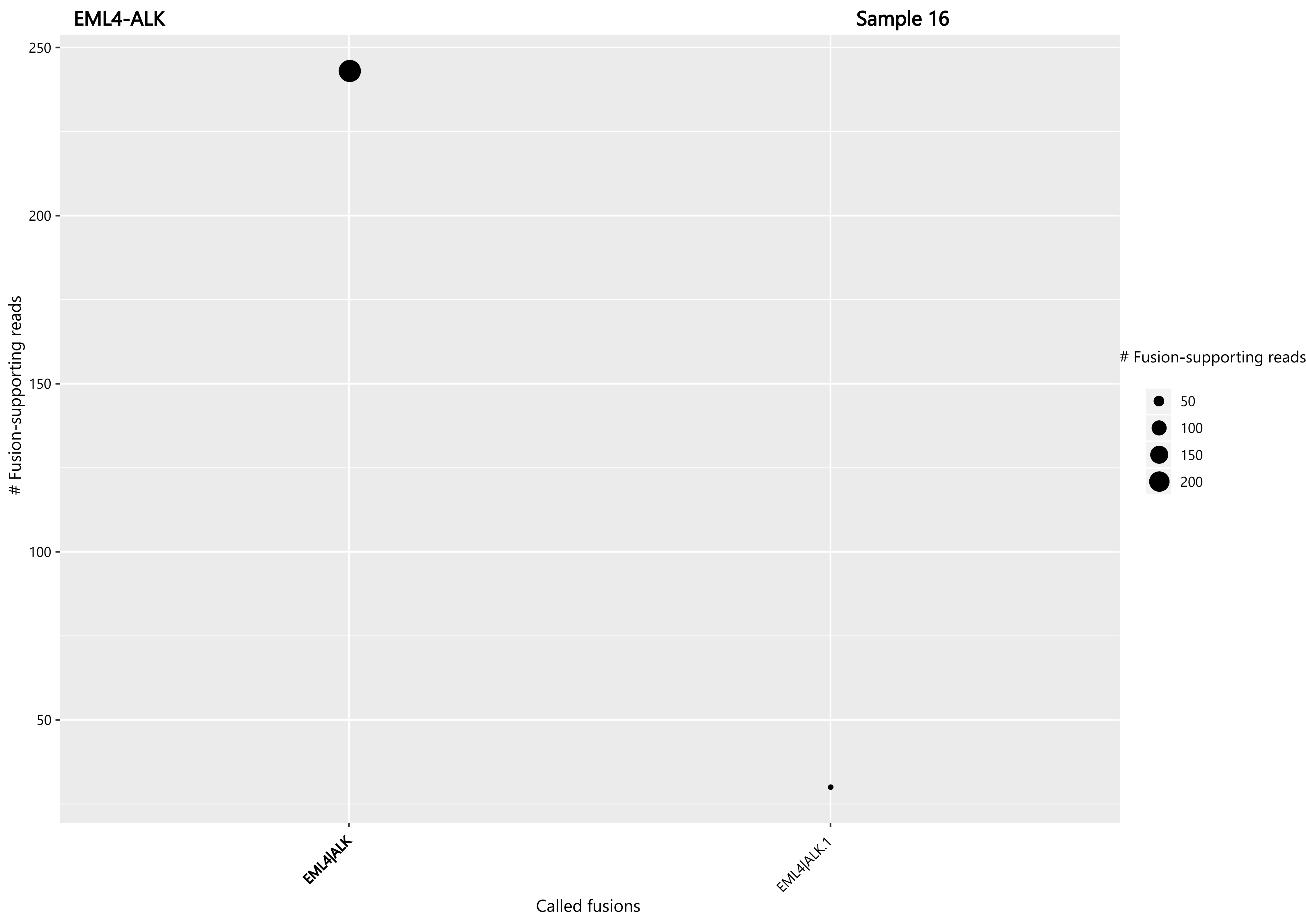

EML4-ALK
Sample 16
250
200
150
100
50
Fusion-supporting reads
EML4|ALK
EML4|ALK.1
Called fusions
Fusion-supporting reads
50
100
150
200

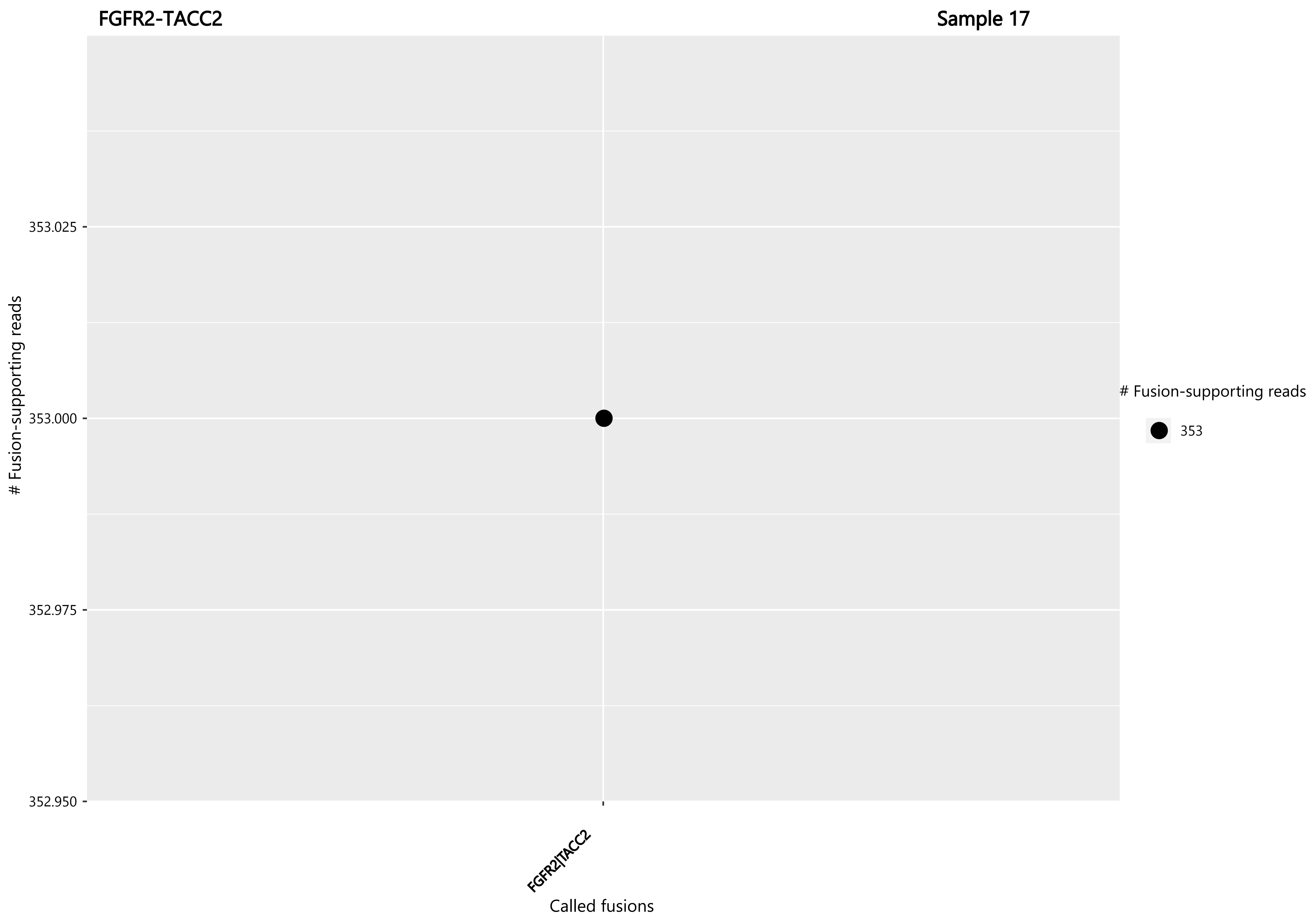

FGFR2-TACC2
Sample 17
Fusion-supporting reads
353.025
353.000
352.975
352.950
FGFR2|TACC2
Called fusions
Fusion-supporting reads
353

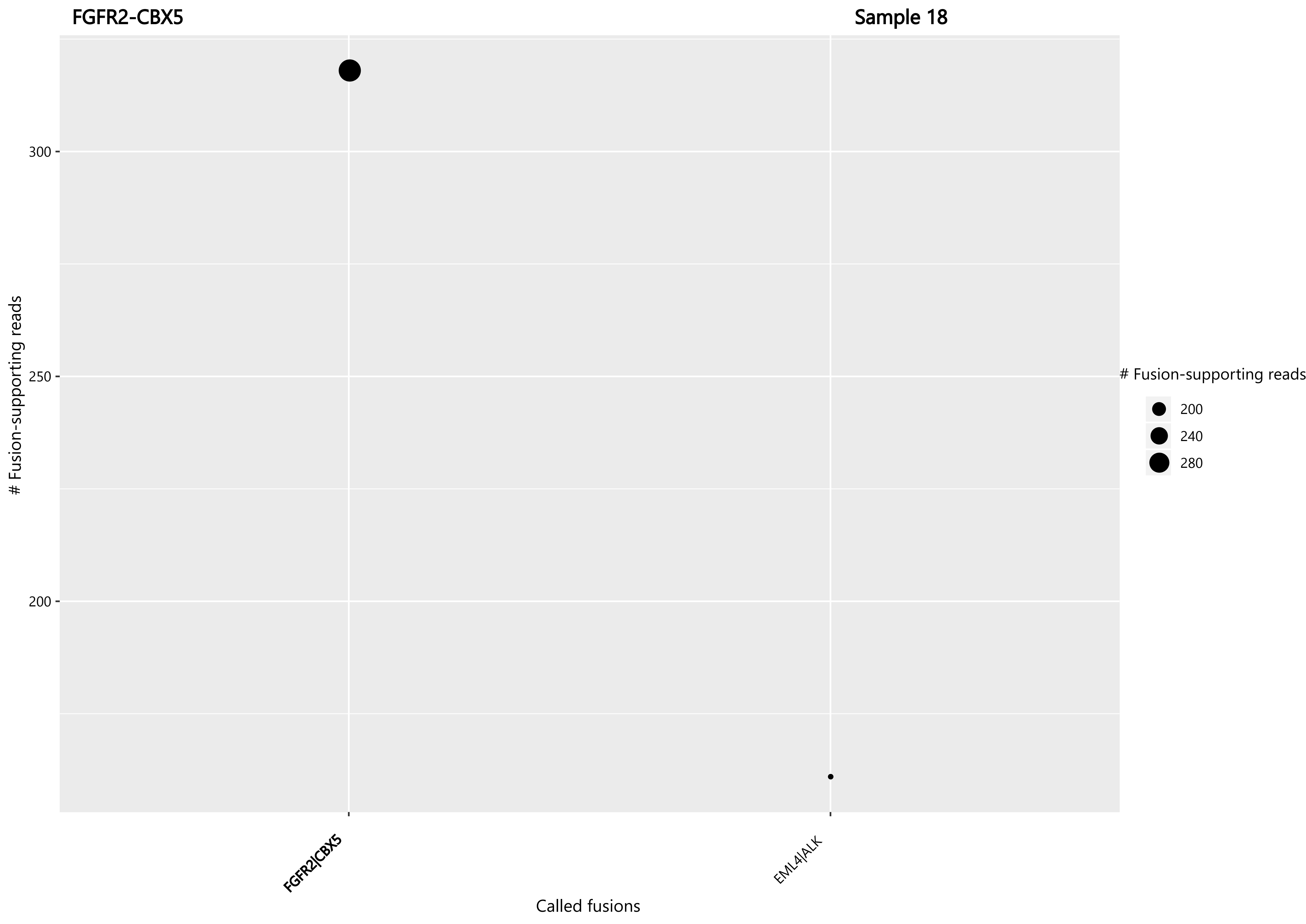
FGFR2-CBX5
Sample 18
300
250
200
Fusion-supporting reads
FGFR2|CBX5
EML4|ALK
Called fusions
Fusion-supporting reads
200
240
280
