# Supplementary material for: Detection of gene fusions using targeted next-generation sequencing: a comparative evaluation
Source: BMC Med Genomics. 2021 Feb 27;14:62. doi: 10.1186/s12920-021-00909-y (PMC7912891; doi:10.1186/s12920-021-00909-y)
Supplement: Supplementary file 11 — Additional file 11: Fig. S11. Fusions detected with the Archer FusionPlex Lung Panel (Archer DX) (v5.1.3) for all samples. Metrics such as quality control scores, in-frame status or filter thresholds were plotted when available. In cases where the same fusion was identified more than once within the same sample, a unique numbering scheme was added at the end of the name to differentiate the candidate fusions. The numbering however, does not imply any special order or preference over the other fusions with the same name. The putative detected fusions were arranged in decreasing order based on the number of fusion-supporting reads. The expected fusion for each sample was highlighted in bold. [file 12920_2021_909_MOESM11_ESM.pdf]

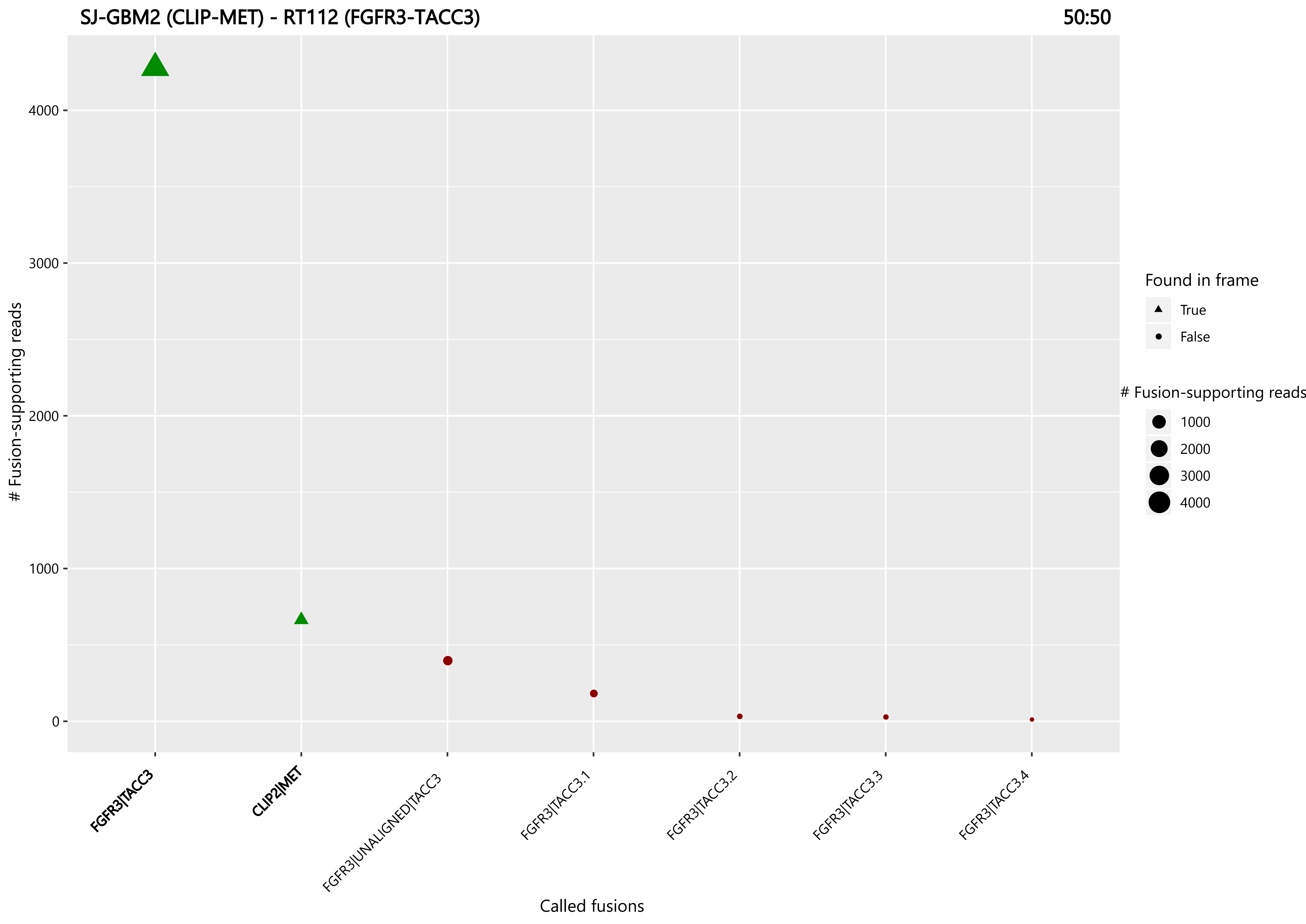
SJ-GBM2 (CLIP-MET) - RT112 (FGFR3-TACC3)
50:50
Fusion-supporting reads
0
1000
2000
3000
4000
Called fusions
FGFR3|TACC3
CLIP2|MET
FGFR3|UNALIGNED|TACC3
FGFR3|TACC3.1
FGFR3|TACC3.2
FGFR3|TACC3.3
FGFR3|TACC3.4
Found in frame
True
False
Fusion-supporting reads
1000
2000
3000
4000

SJ-GBM2 (CLIP-MET) - RT112 (FGFR3-TACC3) 20:80

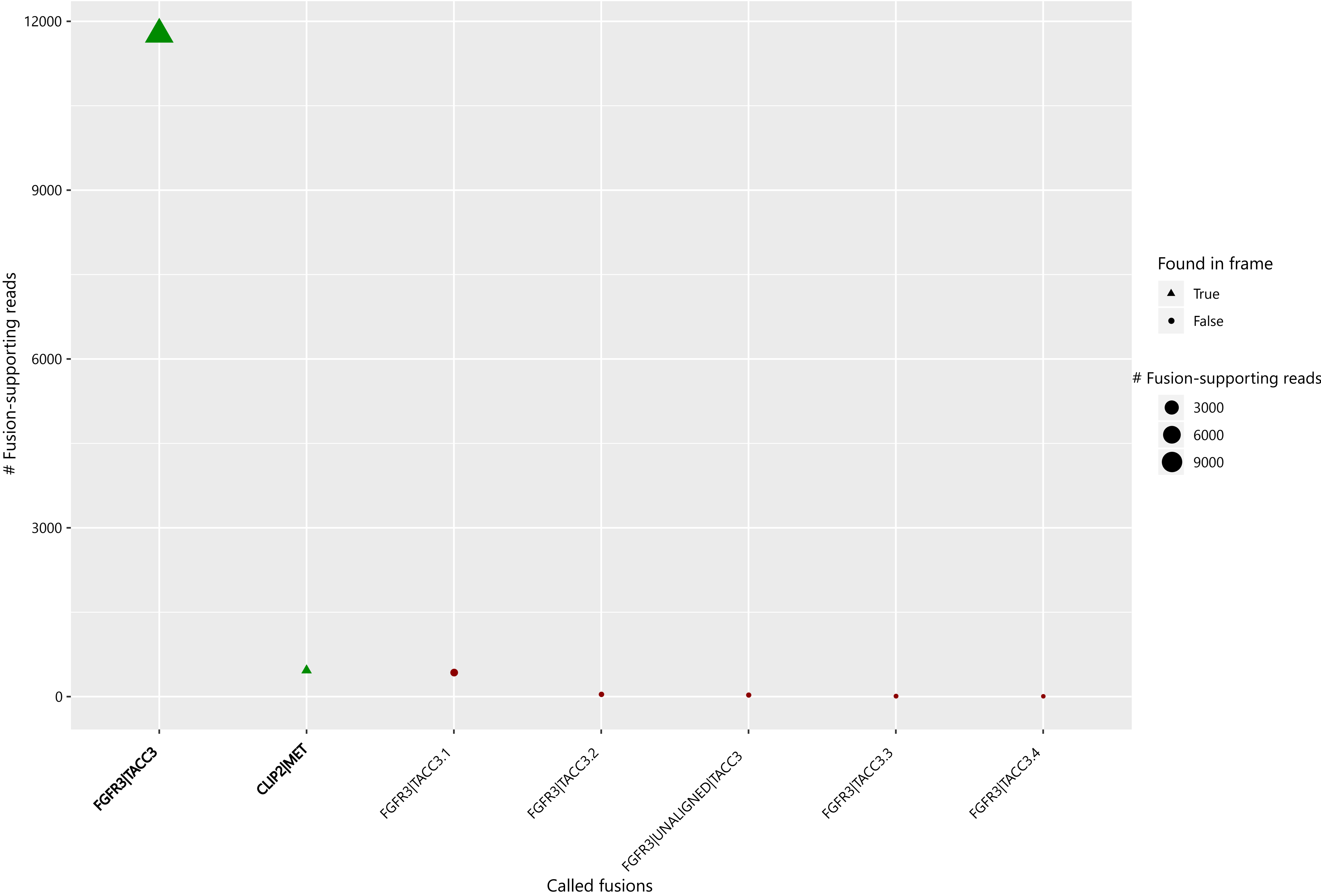

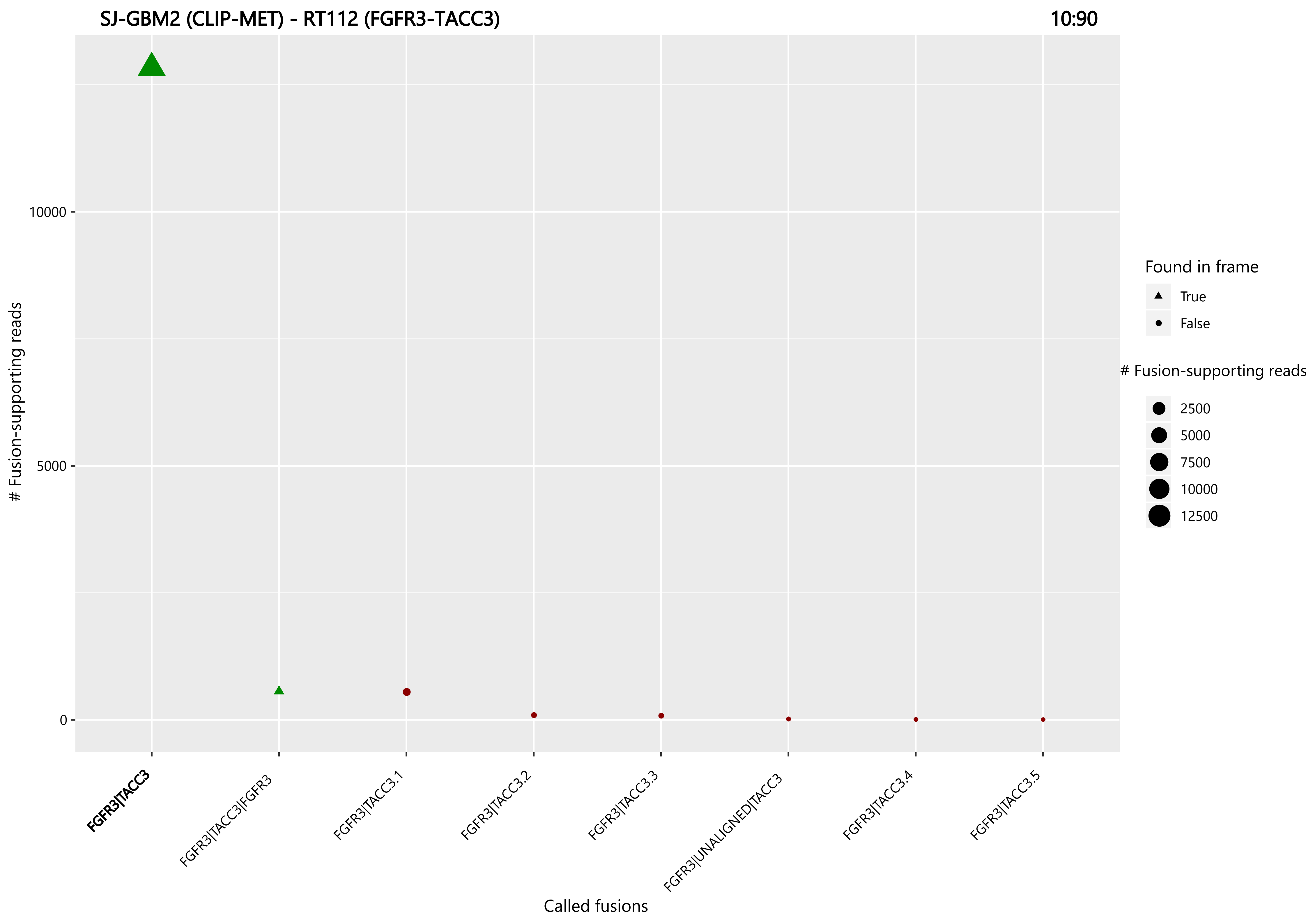
SJ-GBM2 (CLIP-MET) - RT112 (FGFR3-TACC3)
10:90
10000
5000
0
Fusion-supporting reads
FGFR3|TACC3
FGFR3|TACC3|FGFR3
FGFR3|TACC3.1
FGFR3|TACC3.2
FGFR3|TACC3.3
FGFR3|UNALIGNED|TACC3
FGFR3|TACC3.4
FGFR3|TACC3.5
Called fusions
Found in frame
True
False
Fusion-supporting reads
2500
5000
7500
10000
12500

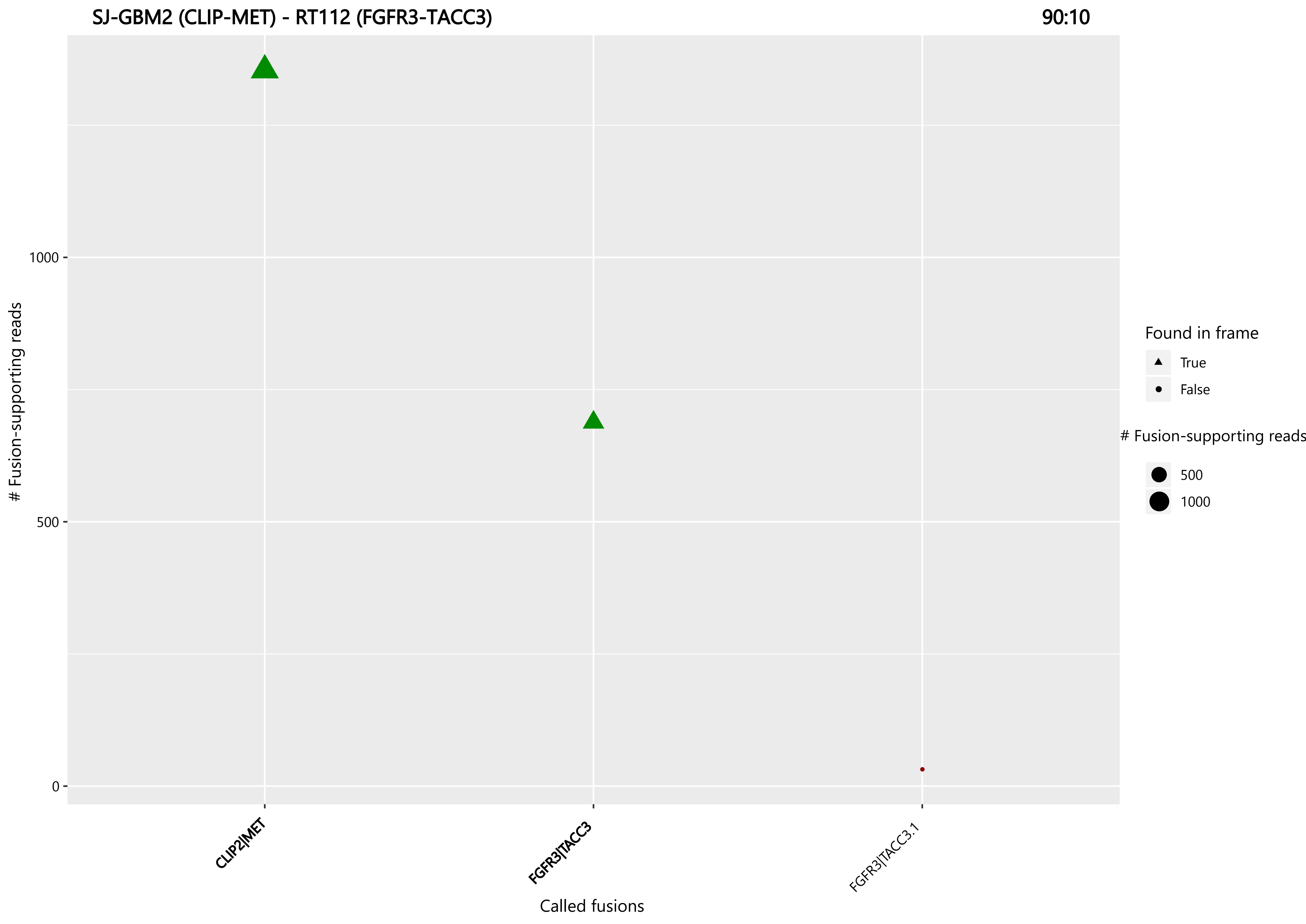
SJ-GBM2 (CLIP-MET) - RT112 (FGFR3-TACC3)
90:10
Fusion-supporting reads
0
500
1000
CLIP2|MET
FGFR3|TACC3
FGFR3|TACC3.1
Called fusions
Found in frame
True
False
Fusion-supporting reads
500
1000

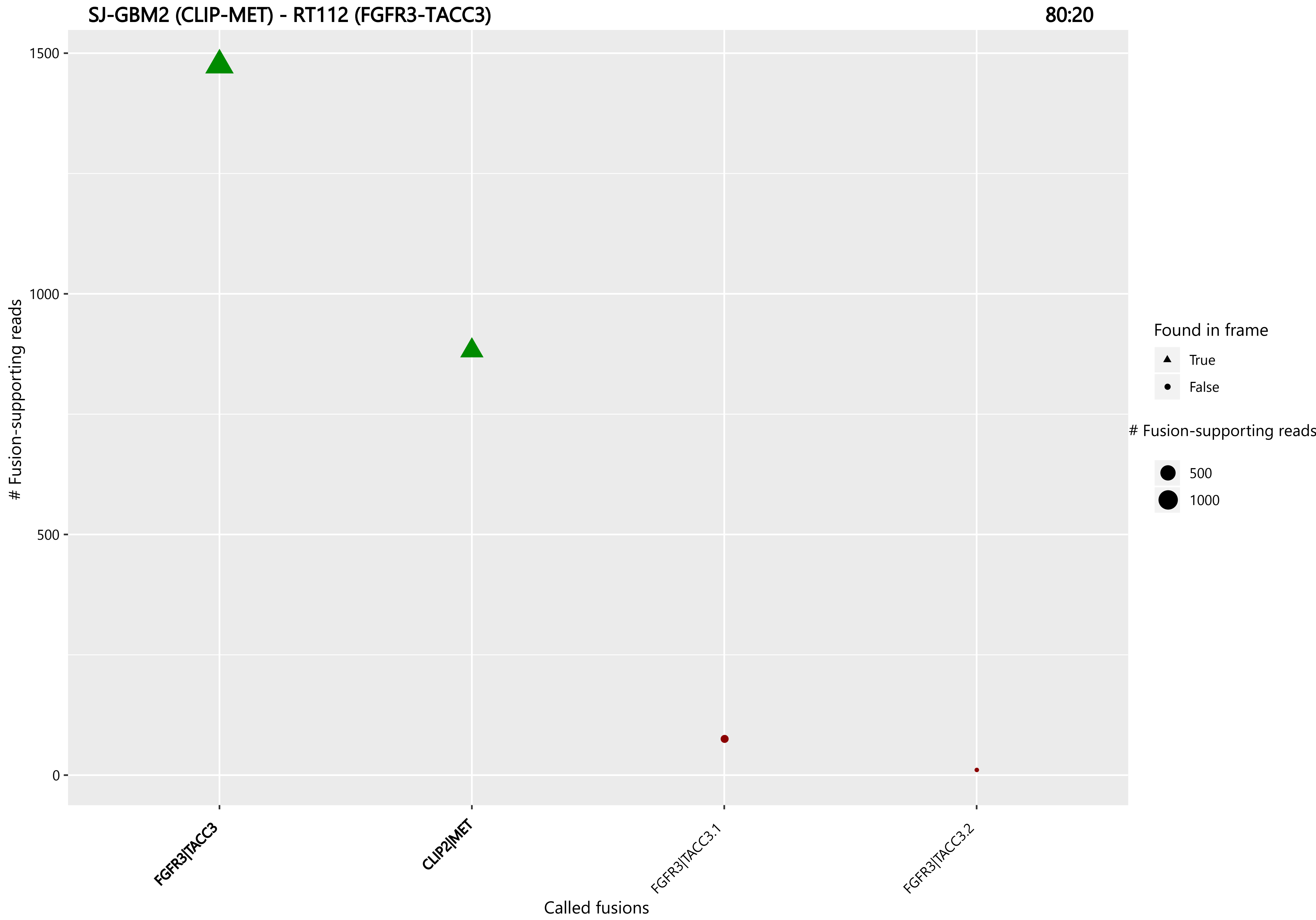

SJ-GBM2 (CLIP-MET) - RT112 (FGFR3-TACC3)
80:20
1500
1000
500
0
Fusion-supporting reads
FGFR3|TACC3
CLIP2|MET
FGFR3|TACC3.1
FGFR3|TACC3.2
Called fusions
Found in frame
True
False
Fusion-supporting reads
500
1000

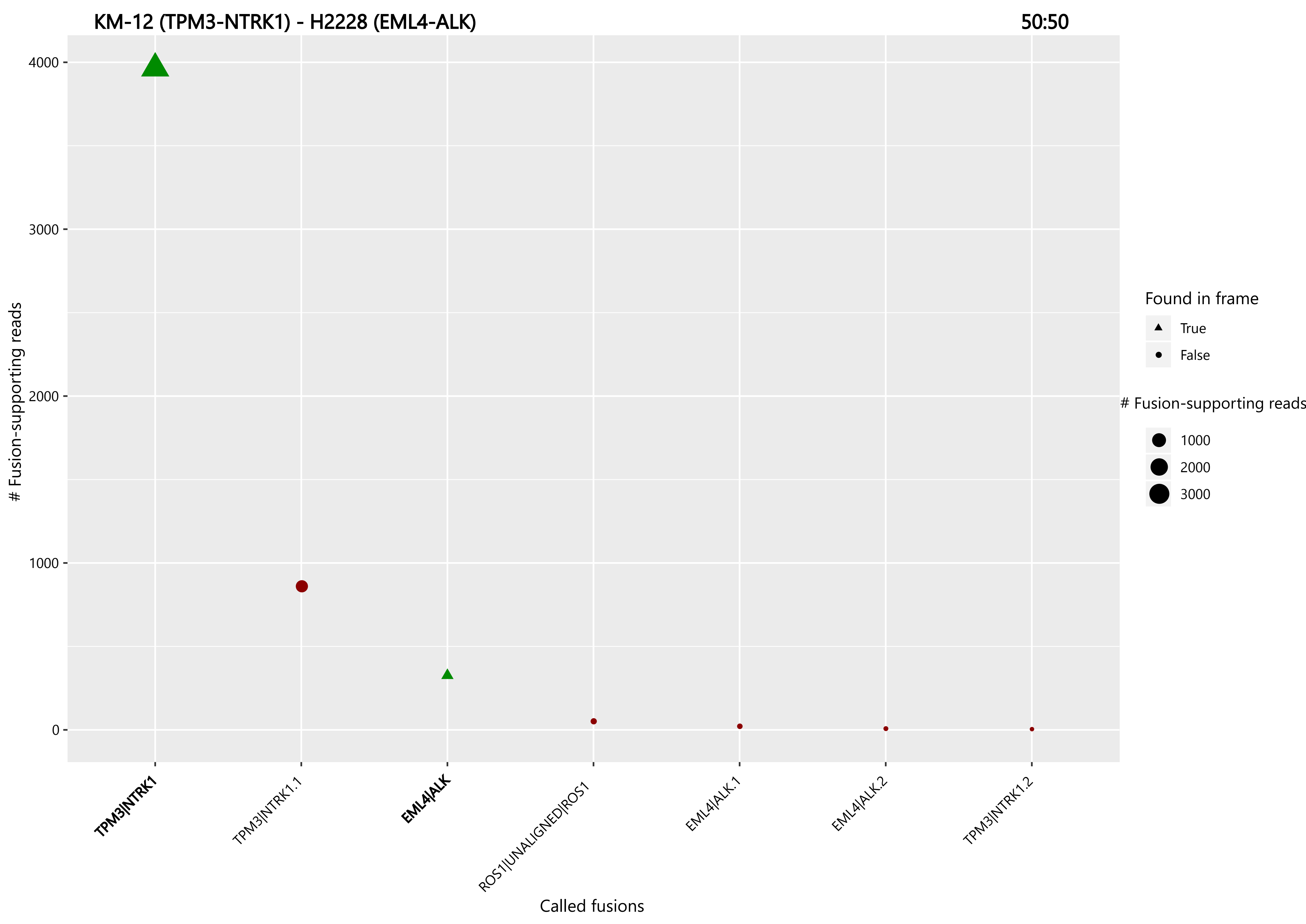

KM-12 (TPM3-NTRK1) - H2228 (EML4-ALK)
50:50
4000
3000
2000
1000
0
Fusion-supporting reads
TPM3|NTRK1
TPM3|NTRK1.1
EML4|ALK
ROS1|UNALIGNED|ROS1
EML4|ALK.1
EML4|ALK.2
TPM3|NTRK1.2
Called fusions
Found in frame
True
False
Fusion-supporting reads
1000
2000
3000

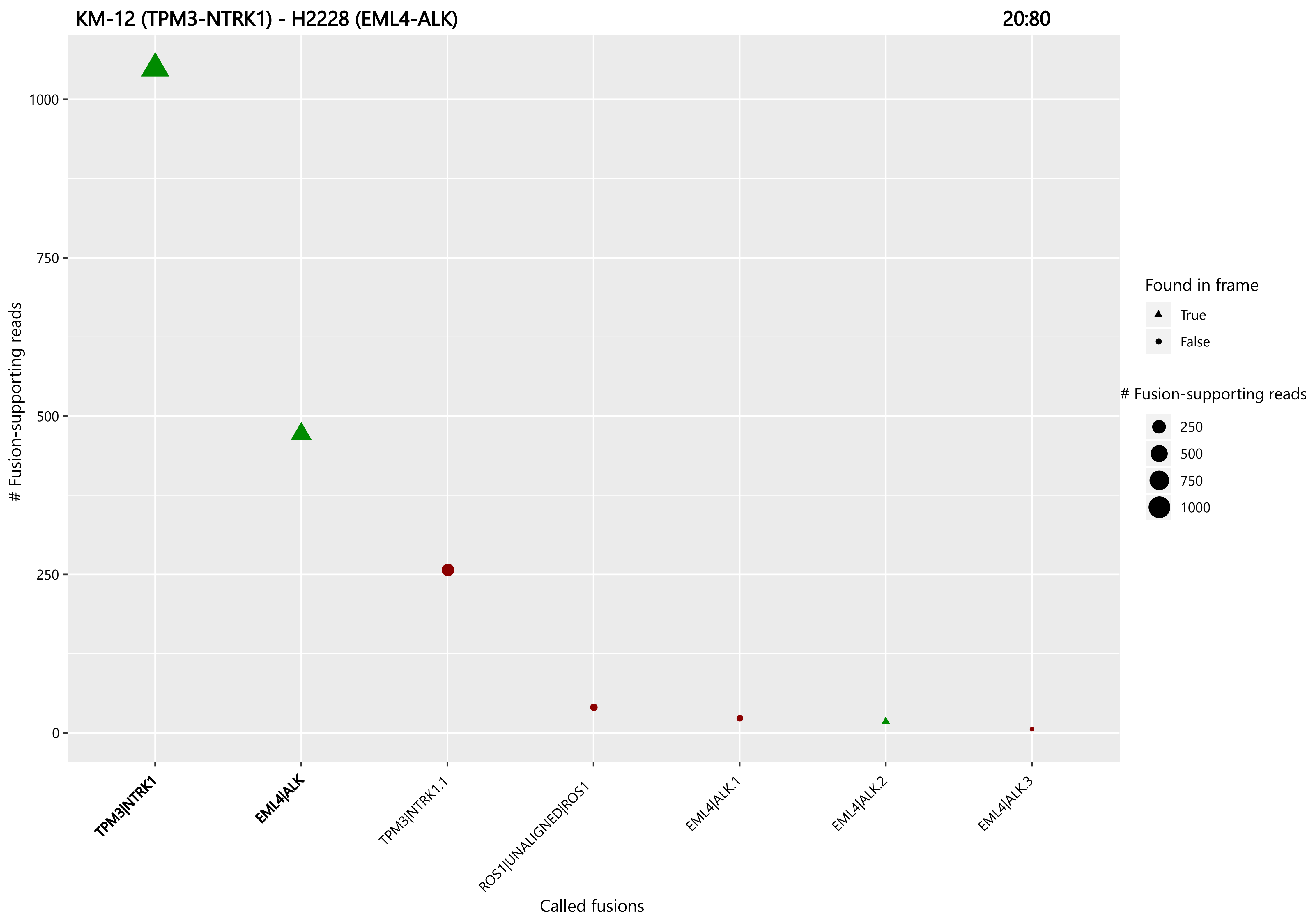
KM-12 (TPM3-NTRK1) - H2228 (EML4-ALK)
20:80
Fusion-supporting reads
1000
750
500
250
0
Called fusions
TPM3|NTRK1
EML4|ALK
TPM3|NTRK1.1
ROS1|UNALIGNED|ROS1
EML4|ALK.1
EML4|ALK.2
EML4|ALK.3
Found in frame
True
False
Fusion-supporting reads
250
500
750
1000

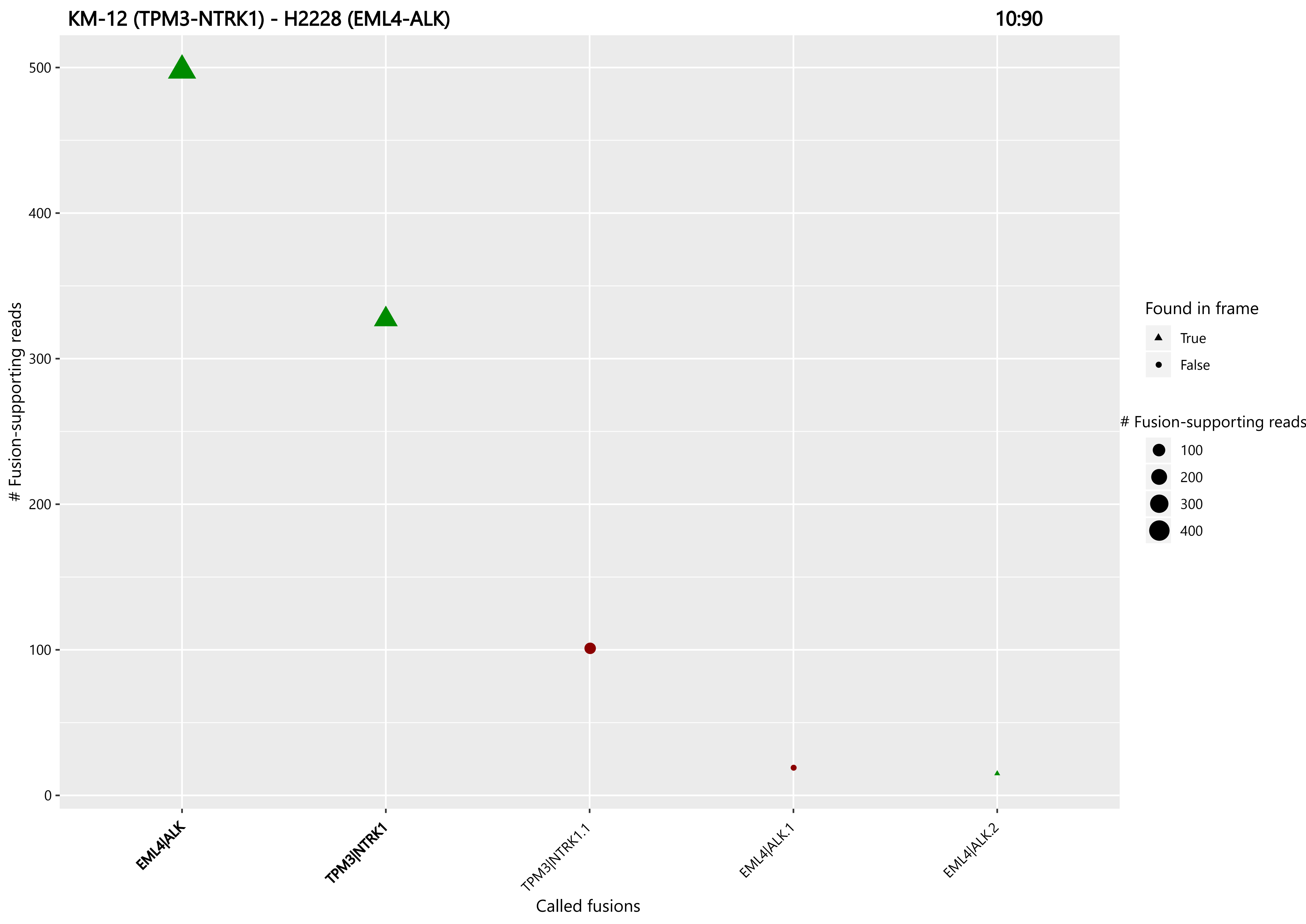
KM-12 (TPM3-NTRK1) - H2228 (EML4-ALK)
10:90
500
400
300
200
100
0
Fusion-supporting reads
EML4|ALK
TPM3|NTRK1
TPM3|NTRK1.1
EML4|ALK.1
EML4|ALK.2
Called fusions
Found in frame
True
False
Fusion-supporting reads
100
200
300
400

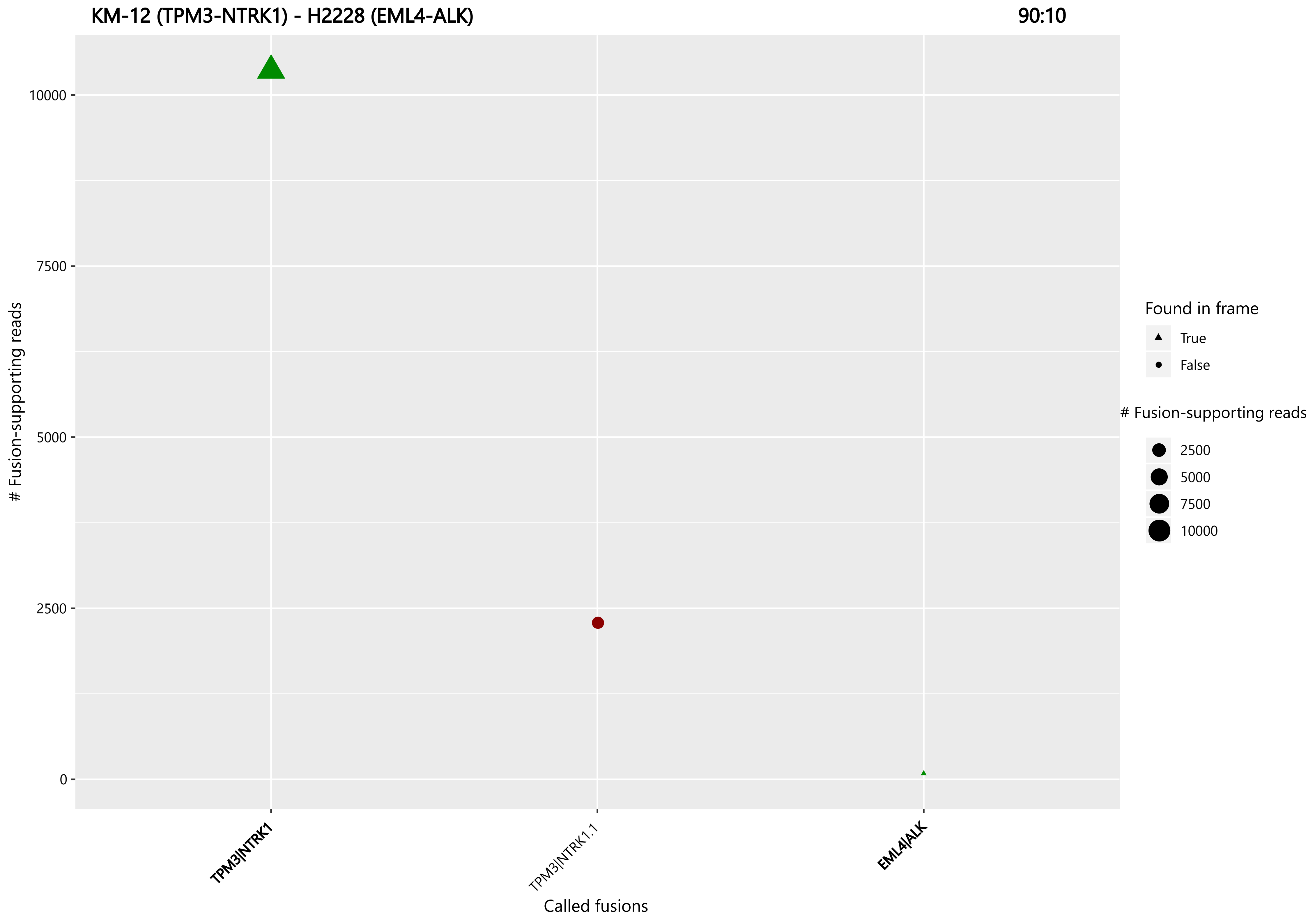
KM-12 (TPM3-NTRK1) - H2228 (EML4-ALK)
90:10
Fusion-supporting reads
10000
7500
5000
2500
0
TPM3|NTRK1
TPM3|NTRK1.1
EML4|ALK
Called fusions
Found in frame
True
False
Fusion-supporting reads
2500
5000
7500
10000

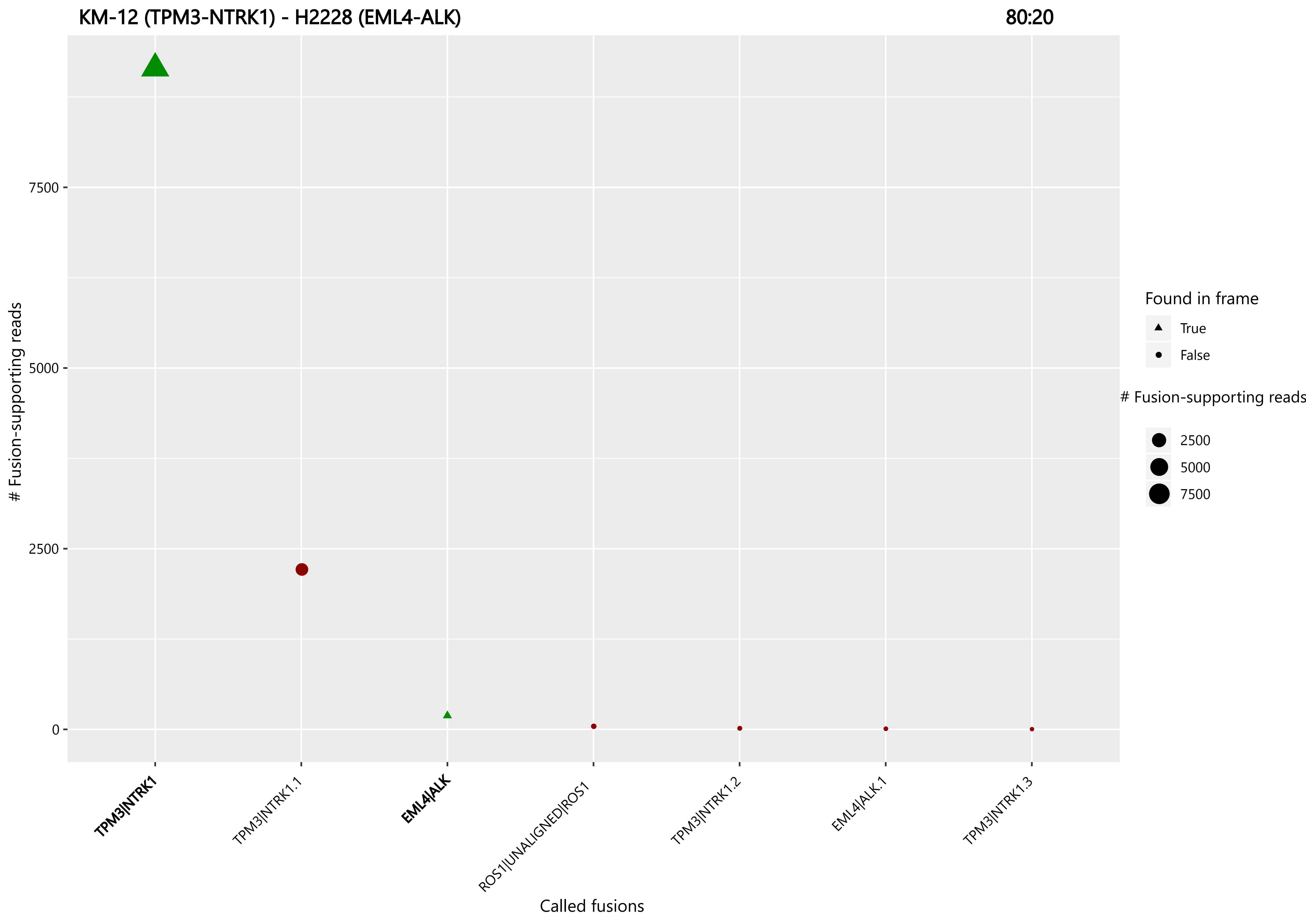
KM-12 (TPM3-NTRK1) - H2228 (EML4-ALK)
80:20
Fusion-supporting reads
7500
5000
2500
0
TPM3|NTRK1
TPM3|NTRK1.1
EML4|ALK
ROS1|UNALIGNED|ROS1
TPM3|NTRK1.2
EML4|ALK.1
TPM3|NTRK1.3
Called fusions
Found in frame
True
False
Fusion-supporting reads
2500
5000
7500

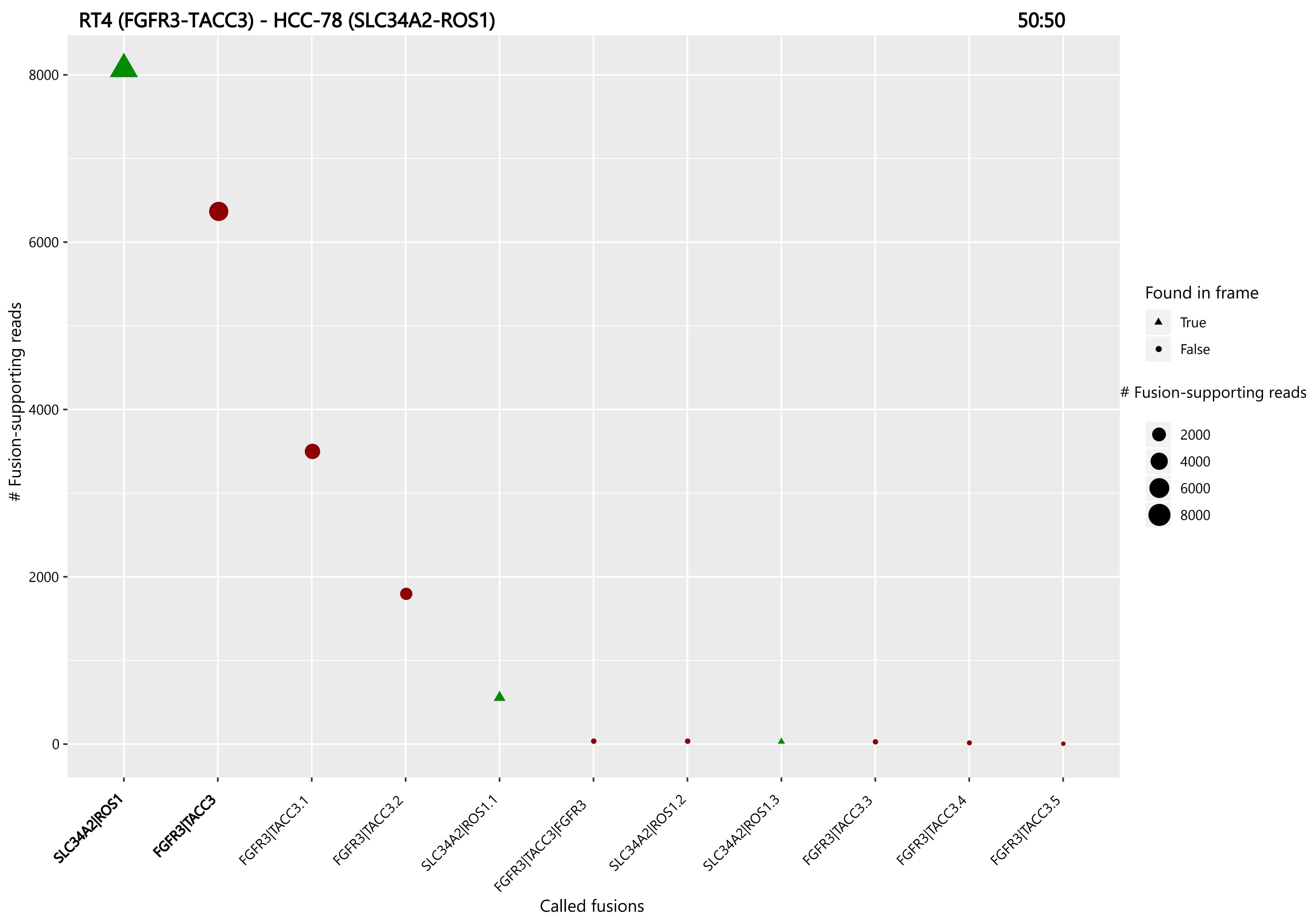

RT4 (FGFR3-TACC3) - HCC-78 (SLC34A2-ROS1)
50:50
Fusion-supporting reads
8000
6000
4000
2000
0
Called fusions
SLC34A2|ROS1
FGFR3|TACC3
FGFR3|TACC3.1
FGFR3|TACC3.2
SLC34A2|ROS1.1
FGFR3|TACC3|FGFR3
SLC34A2|ROS1.2
SLC34A2|ROS1.3
FGFR3|TACC3.3
FGFR3|TACC3.4
FGFR3|TACC3.5
Found in frame
True
False
Fusion-supporting reads
2000
4000
6000
8000

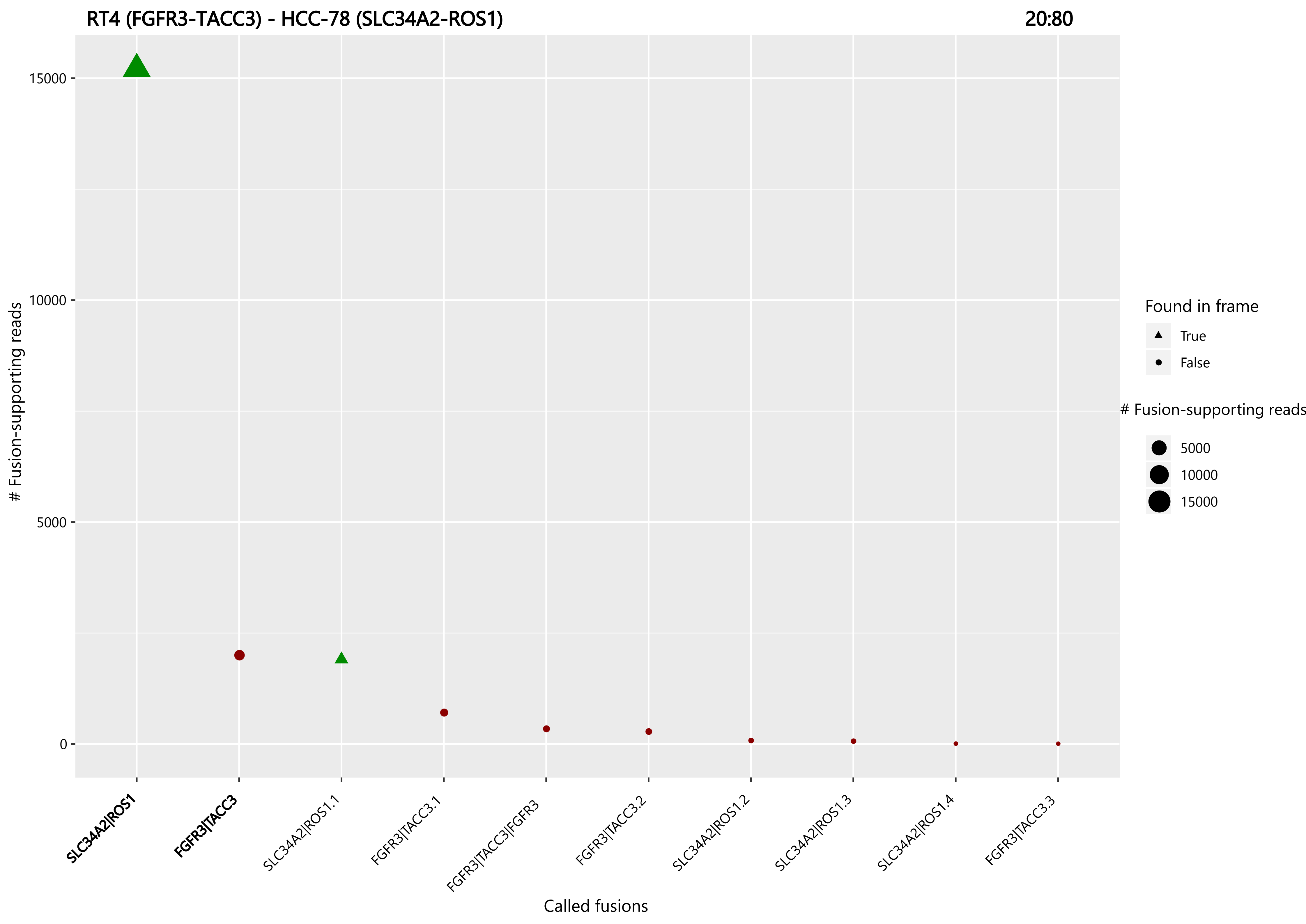

RT4 (FGFR3-TACC3) - HCC-78 (SLC34A2-ROS1)
20:80
15000
10000
5000
0
Fusion-supporting reads
SLC34A2|ROS1
FGFR3|TACC3
SLC34A2|ROS1.1
FGFR3|TACC3.1
FGFR3|TACC3|FGFR3
FGFR3|TACC3.2
SLC34A2|ROS1.2
SLC34A2|ROS1.3
SLC34A2|ROS1.4
FGFR3|TACC3.3
Called fusions
Found in frame
True
False
Fusion-supporting reads
5000
10000
15000

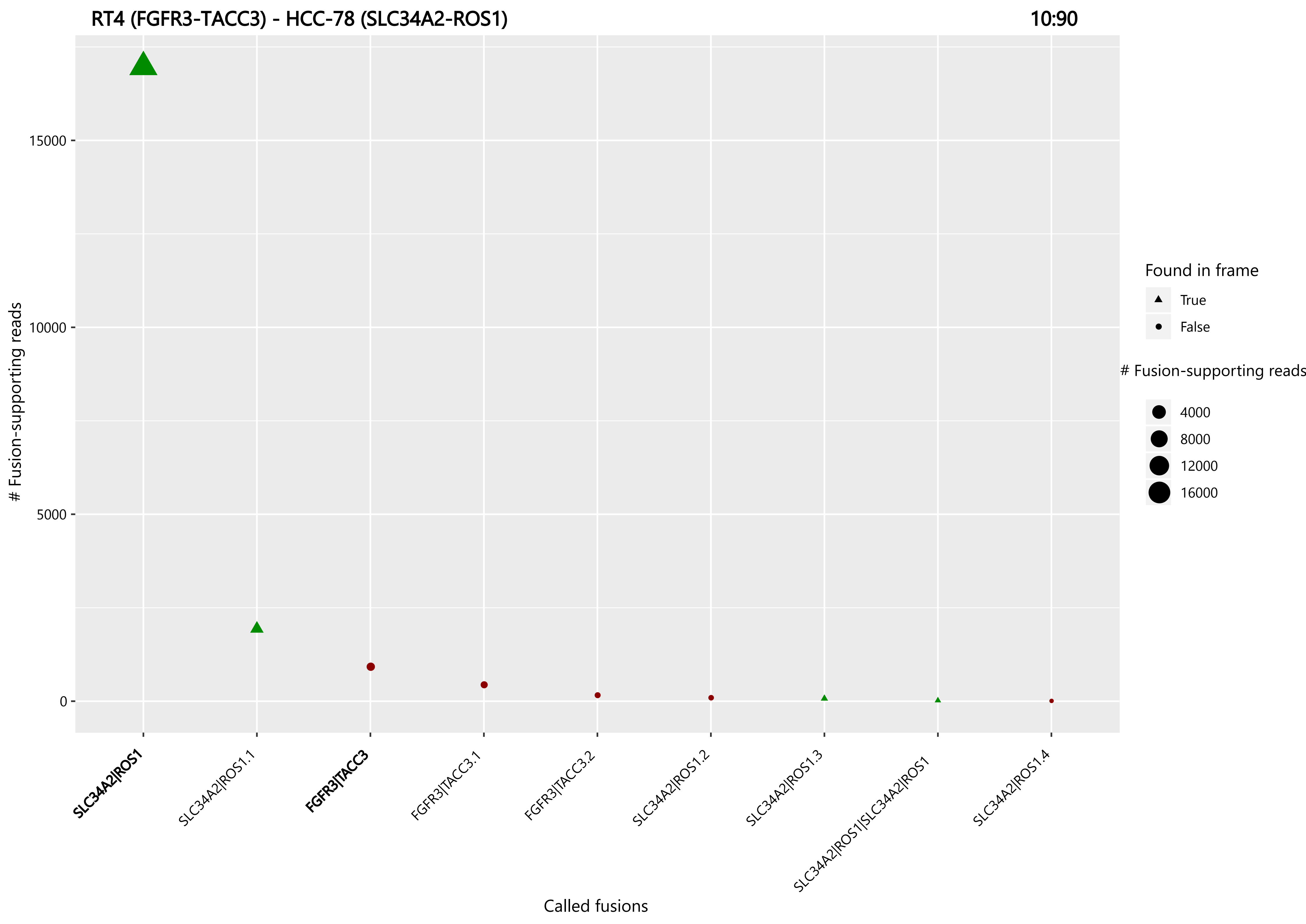
RT4 (FGFR3-TACC3) - HCC-78 (SLC34A2-ROS1)
10:90
15000
10000
5000
0
Fusion-supporting reads
SLC34A2|ROS1
SLC34A2|ROS1.1
FGFR3|TACC3
FGFR3|TACC3.1
FGFR3|TACC3.2
SLC34A2|ROS1.2
SLC34A2|ROS1.3
SLC34A2|ROS1|SLC34A2|ROS1
SLC34A2|ROS1.4
Called fusions
Found in frame
True
False
Fusion-supporting reads
4000
8000
12000
16000

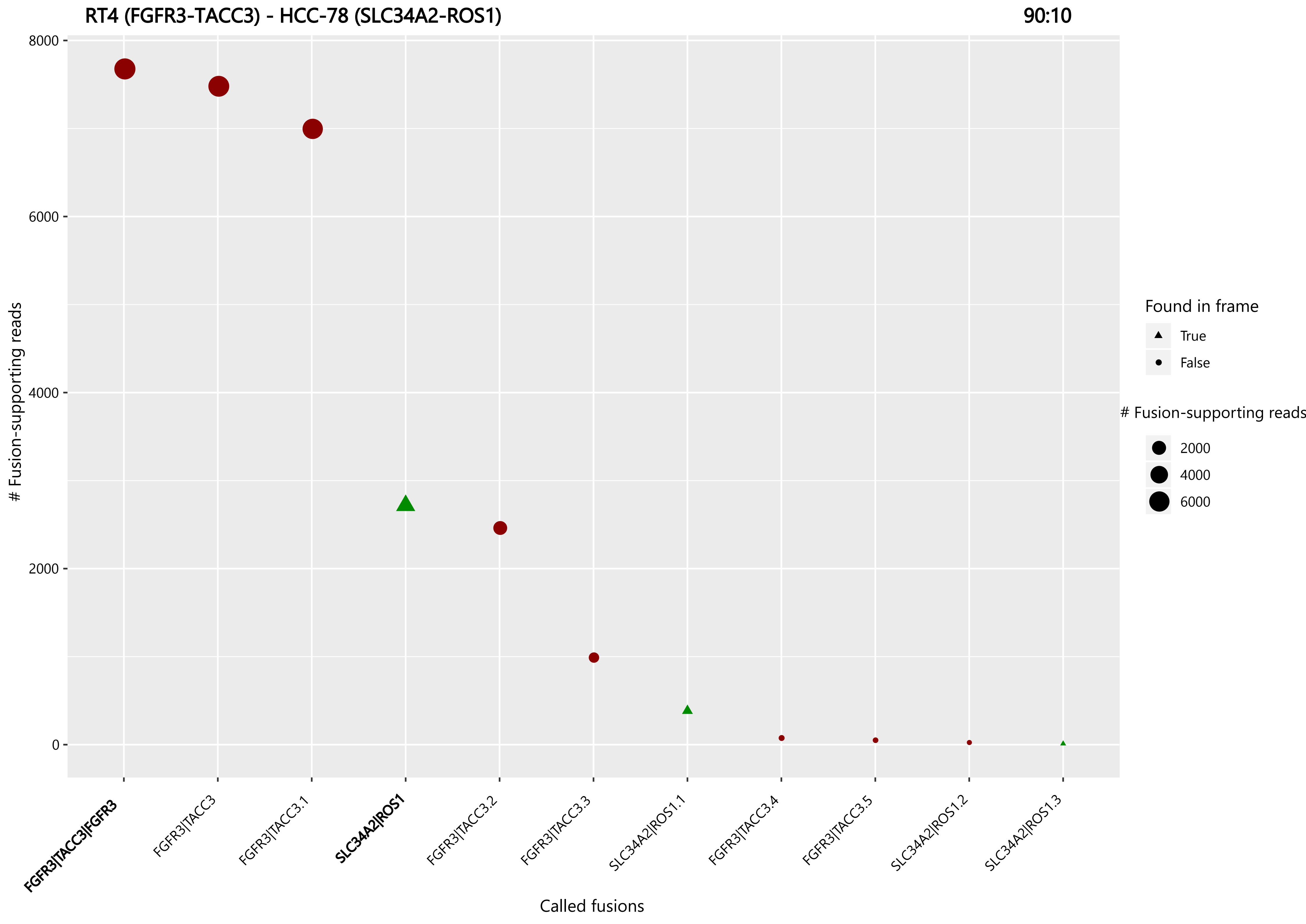

RT4 (FGFR3-TACC3) - HCC-78 (SLC34A2-ROS1)
90:10
8000
6000
4000
2000
0
Fusion-supporting reads
FGFR3|TACC3|FGFR3
FGFR3|TACC3
FGFR3|TACC3.1
SLC34A2|ROS1
FGFR3|TACC3.2
FGFR3|TACC3.3
SLC34A2|ROS1.1
FGFR3|TACC3.4
FGFR3|TACC3.5
SLC34A2|ROS1.2
SLC34A2|ROS1.3
Called fusions
Found in frame
True
False
Fusion-supporting reads
2000
4000
6000

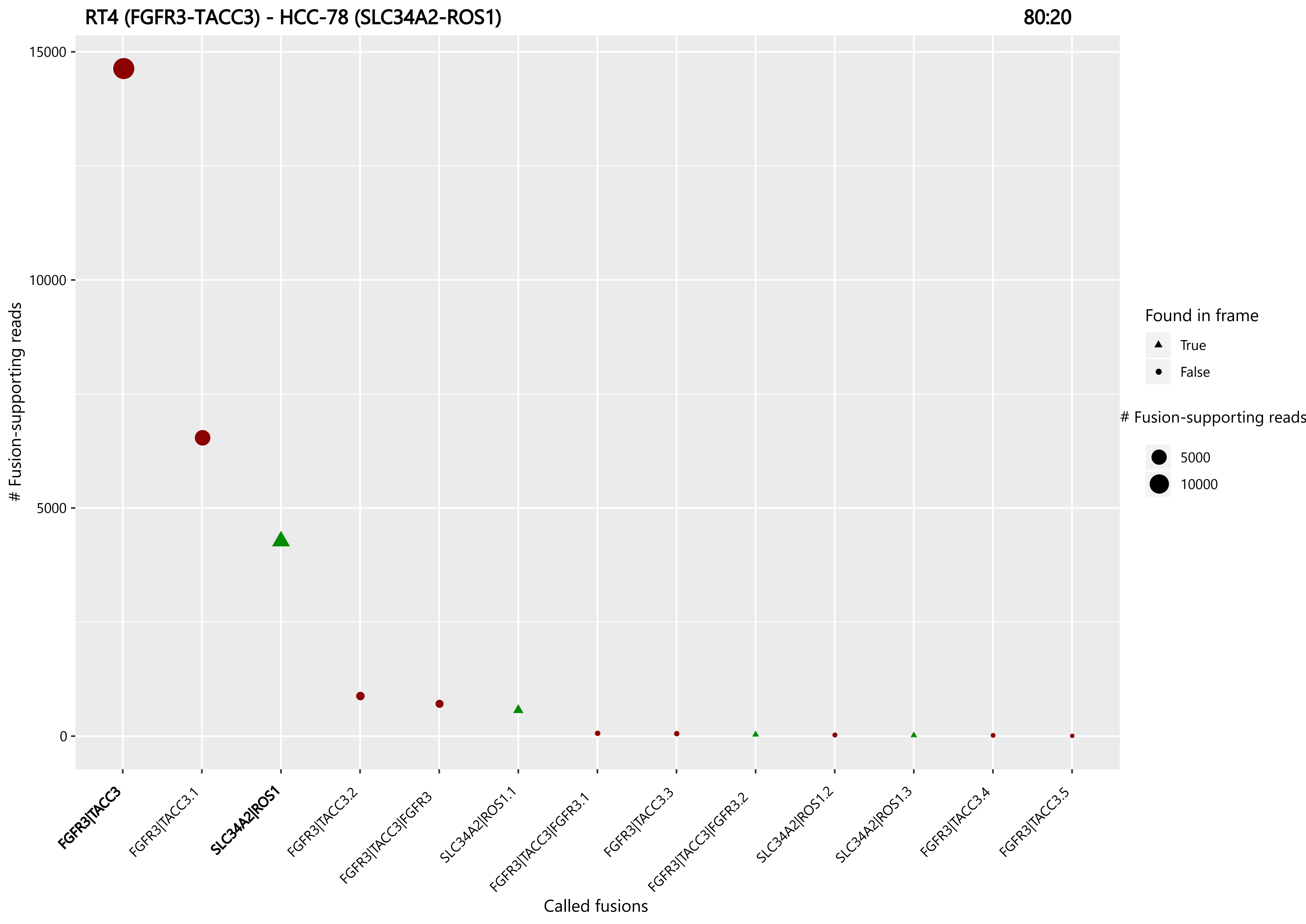

RT4 (FGFR3-TACC3) - HCC-78 (SLC34A2-ROS1)
80:20
15000
10000
5000
0
Fusion-supporting reads
Called fusions
FGFR3|TACC3
FGFR3|TACC3.1
SLC34A2|ROS1
FGFR3|TACC3.2
FGFR3|TACC3|FGFR3
SLC34A2|ROS1.1
FGFR3|TACC3|FGFR3.1
FGFR3|TACC3.3
FGFR3|TACC3|FGFR3.2
SLC34A2|ROS1.2
SLC34A2|ROS1.3
FGFR3|TACC3.4
FGFR3|TACC3.5
Found in frame
True
False
Fusion-supporting reads
5000
10000

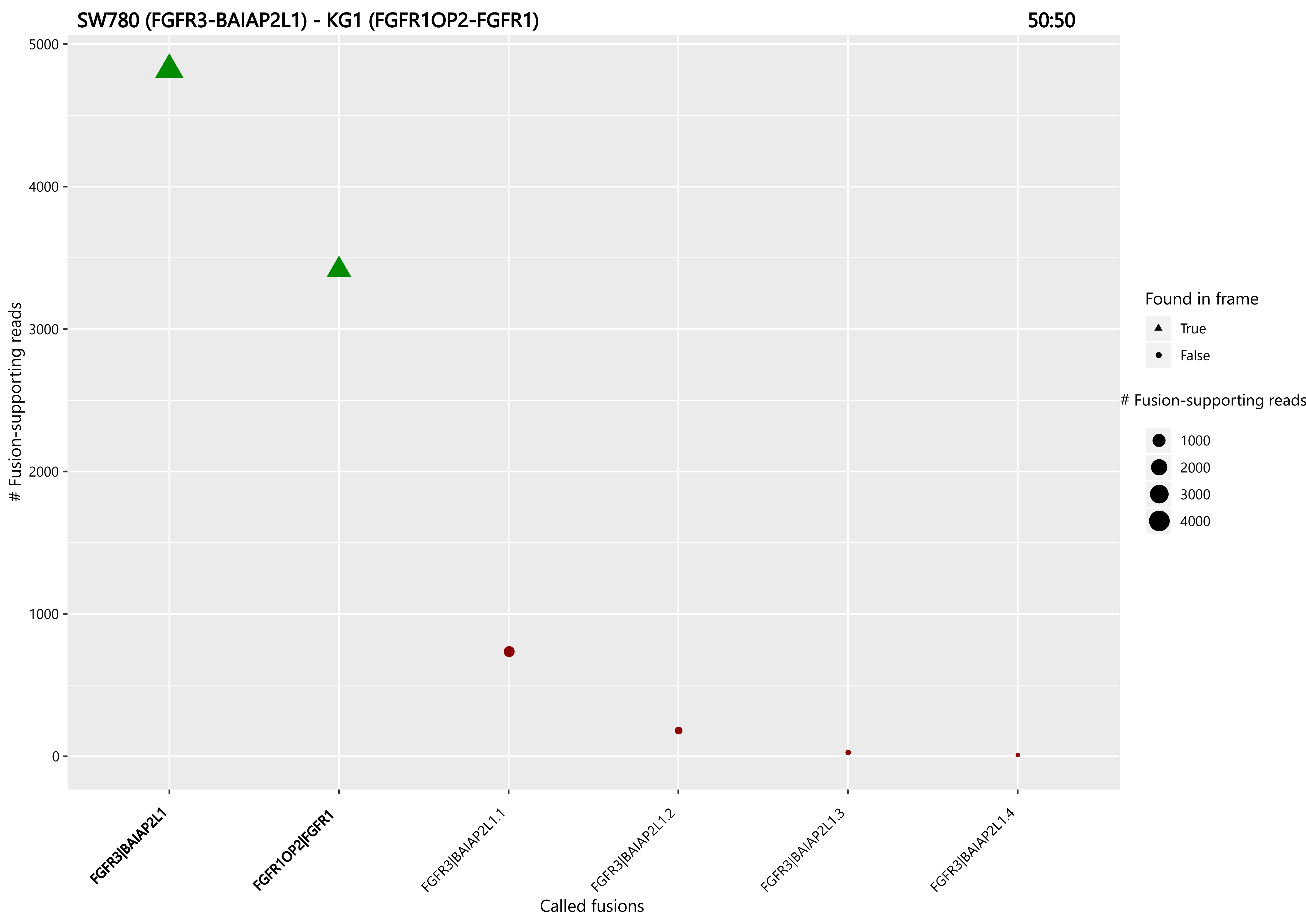

SW780 (FGFR3-BAIAP2L1) - KG1 (FGFR1OP2-FGFR1)
50:50
Fusion-supporting reads
5000
4000
3000
2000
1000
0
FGFR3|BAIAP2L1
FGFR1OP2|FGFR1
FGFR3|BAIAP2L1.1
FGFR3|BAIAP2L1.2
FGFR3|BAIAP2L1.3
FGFR3|BAIAP2L1.4
Called fusions
Found in frame
True
False
Fusion-supporting reads
1000
2000
3000
4000

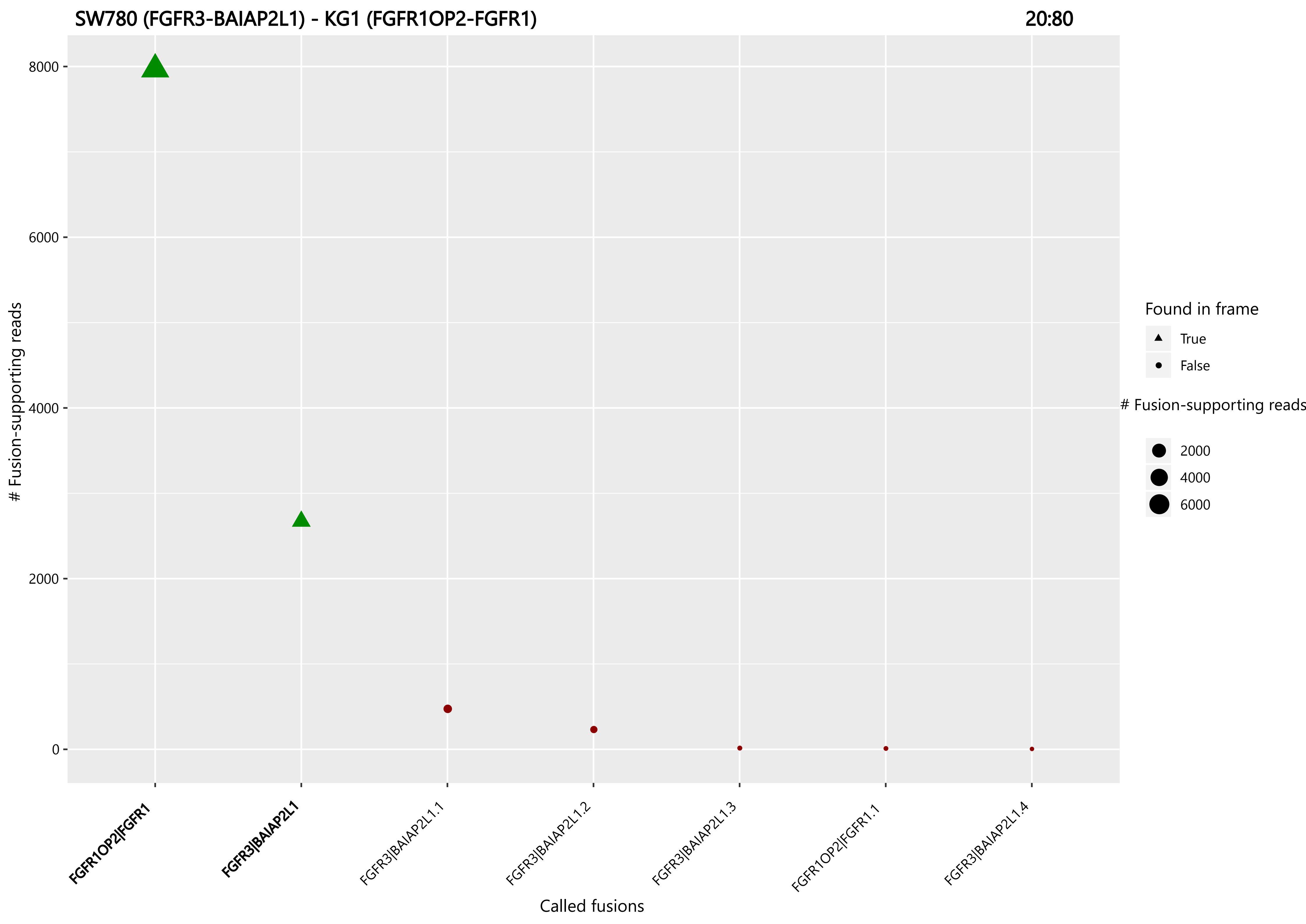

SW780 (FGFR3-BAIAP2L1) - KG1 (FGFR1OP2-FGFR1)
20:80
Fusion-supporting reads
8000
6000
4000
2000
0
FGFR1OP2|FGFR1
FGFR3|BAIAP2L1
FGFR3|BAIAP2L1.1
FGFR3|BAIAP2L1.2
FGFR3|BAIAP2L1.3
FGFR1OP2|FGFR1.1
FGFR3|BAIAP2L1.4
Called fusions
Found in frame
True
False
Fusion-supporting reads
2000
4000
6000

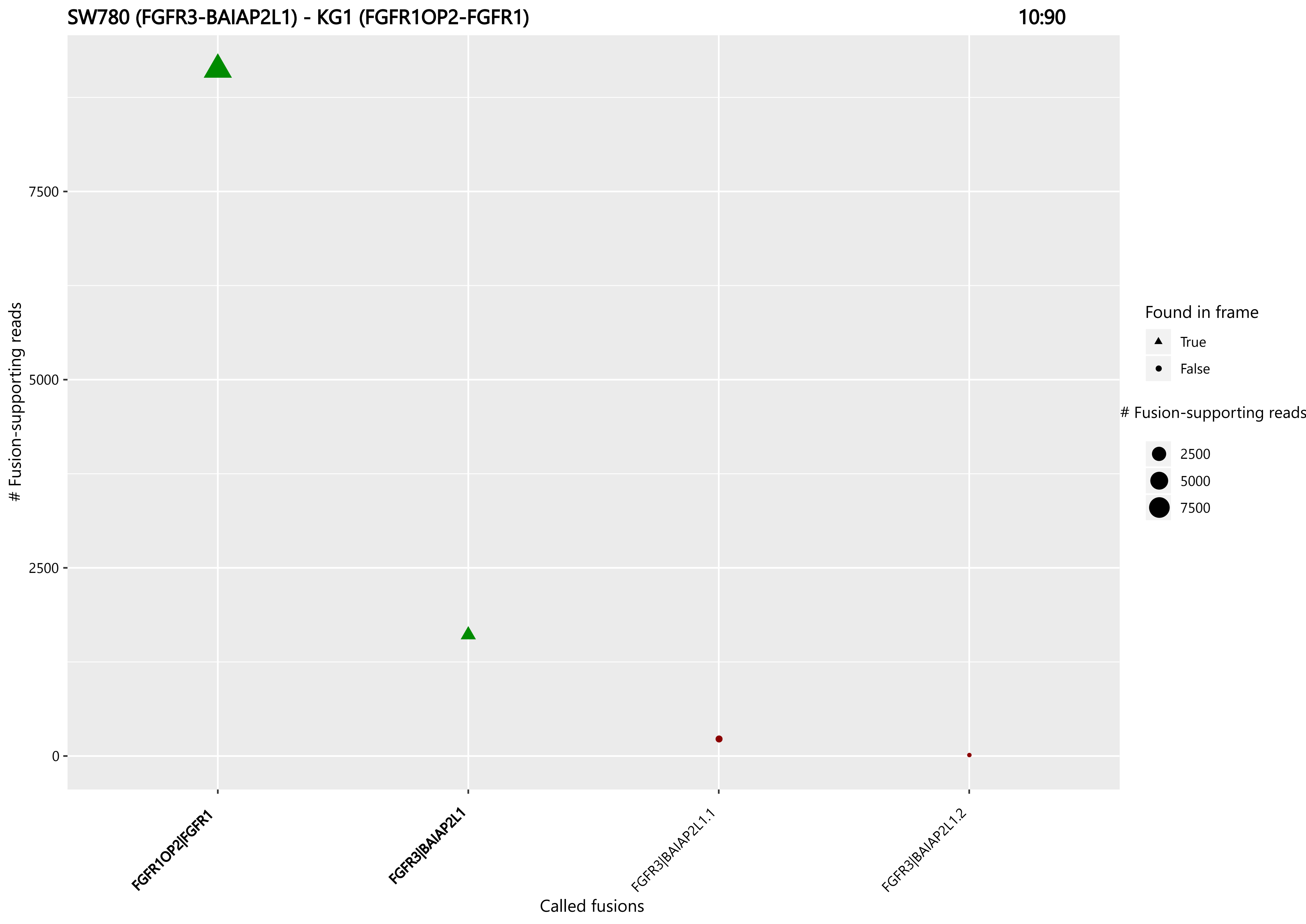
SW780 (FGFR3-BAIAP2L1) - KG1 (FGFR1OP2-FGFR1)
10:90
7500
5000
2500
0
Fusion-supporting reads
FGFR1OP2|FGFR1
FGFR3|BAIAP2L1
FGFR3|BAIAP2L1.1
FGFR3|BAIAP2L1.2
Called fusions
Found in frame
True
False
Fusion-supporting reads
2500
5000
7500

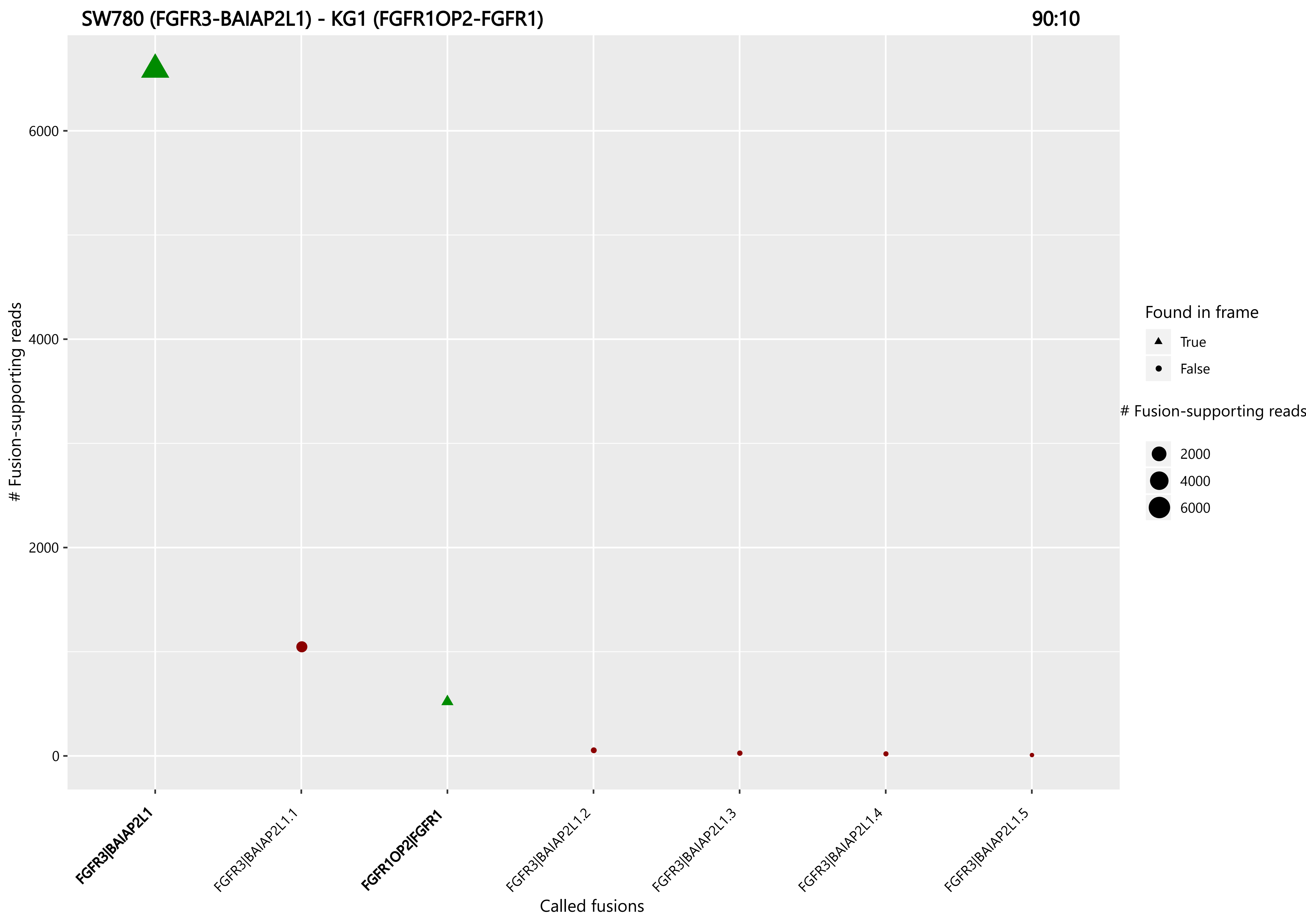

SW780 (FGFR3-BAIAP2L1) - KG1 (FGFR1OP2-FGFR1)
90:10
Fusion-supporting reads
6000
4000
2000
0
FGFR3|BAIAP2L1
FGFR3|BAIAP2L1.1
FGFR1OP2|FGFR1
FGFR3|BAIAP2L1.2
FGFR3|BAIAP2L1.3
FGFR3|BAIAP2L1.4
FGFR3|BAIAP2L1.5
Called fusions
Found in frame
True
False
Fusion-supporting reads
2000
4000
6000

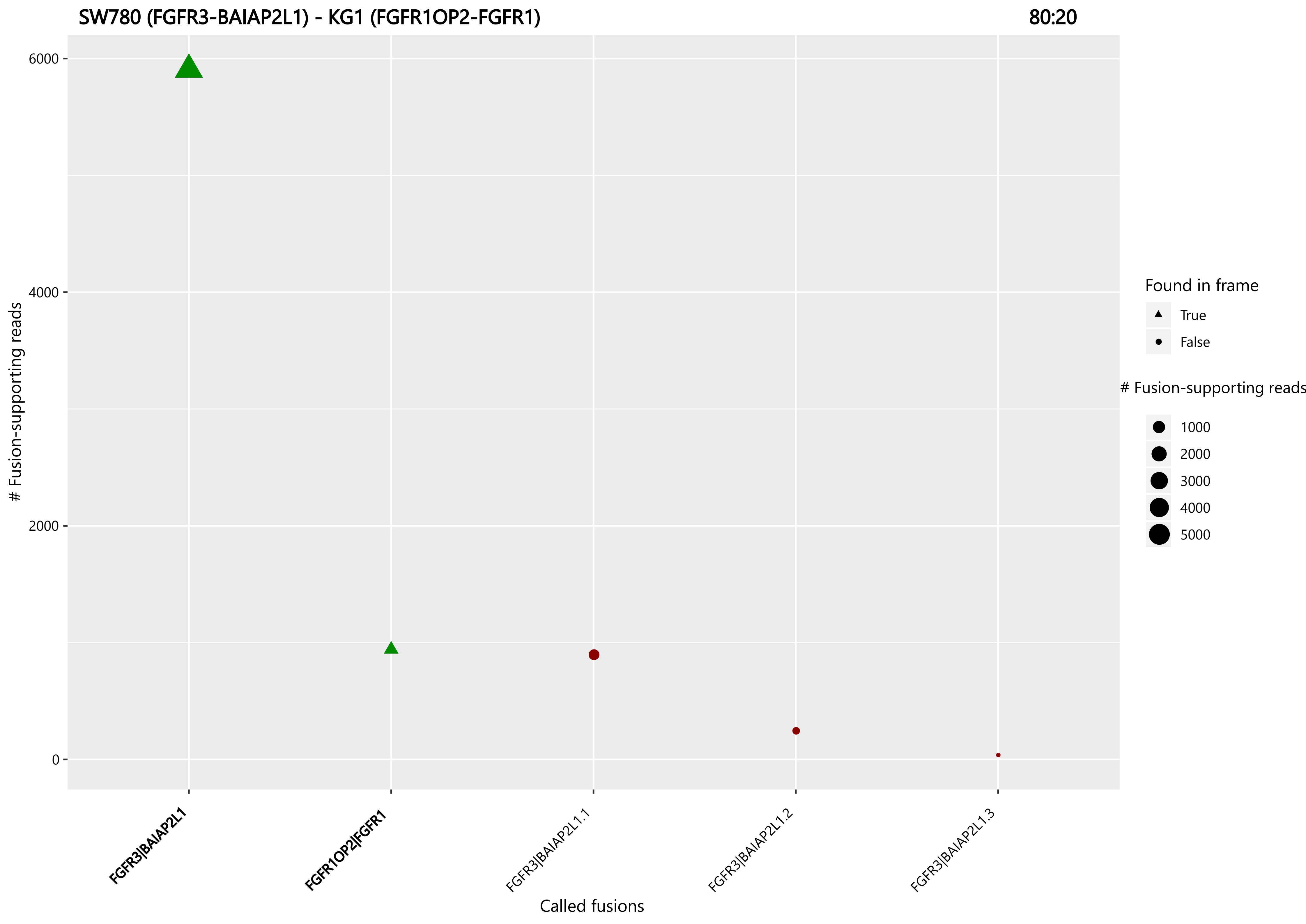
SW780 (FGFR3-BAIAP2L1) - KG1 (FGFR1OP2-FGFR1)
80:20
Fusion-supporting reads
6000
4000
2000
0
Found in frame
True
False
Fusion-supporting reads
1000
2000
3000
4000
5000
FGFR3|BAIAP2L1
FGFR1OP2|FGFR1
FGFR3|BAIAP2L1.1
FGFR3|BAIAP2L1.2
FGFR3|BAIAP2L1.3
Called fusions

KIA1549-BRAF

Sample 1

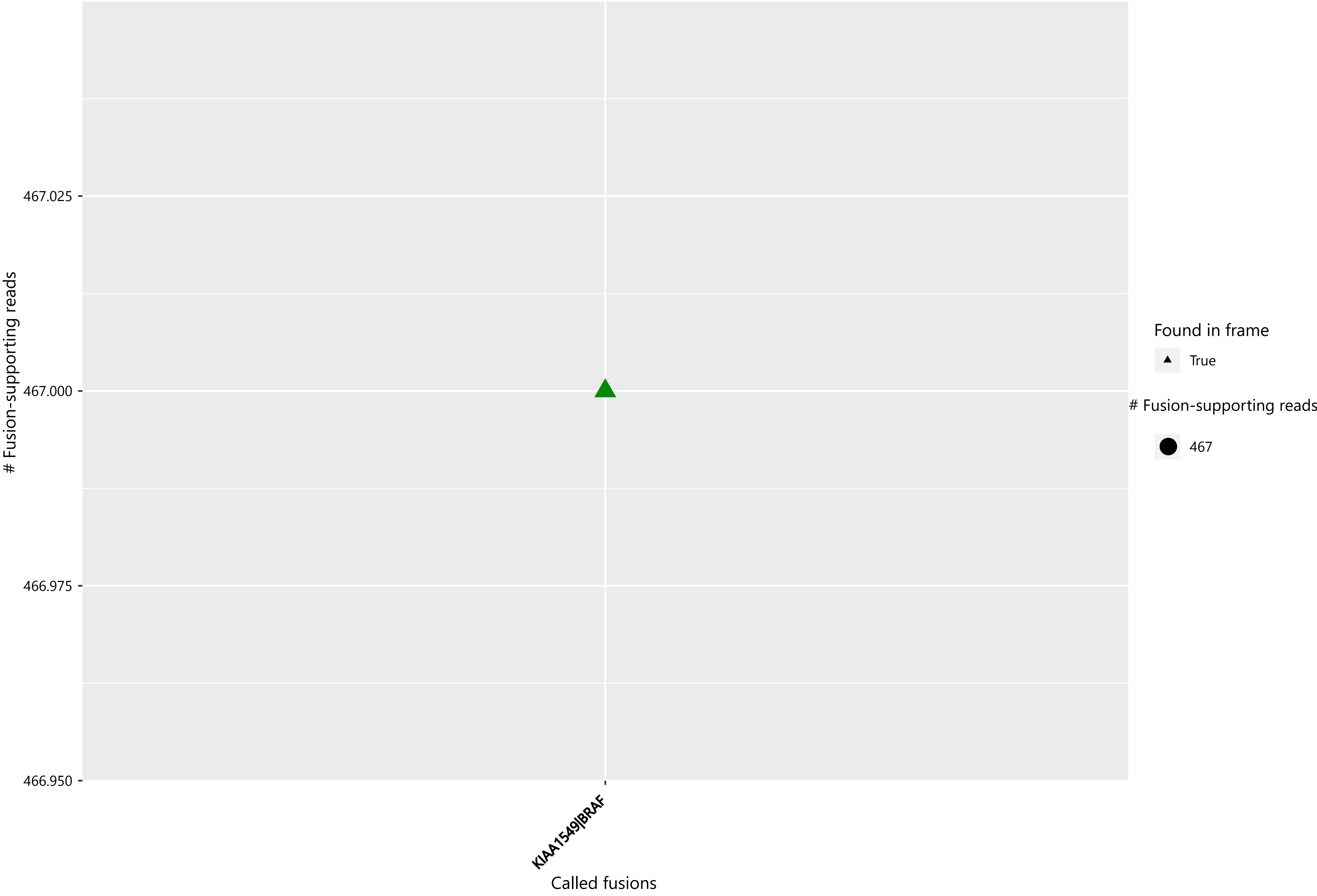

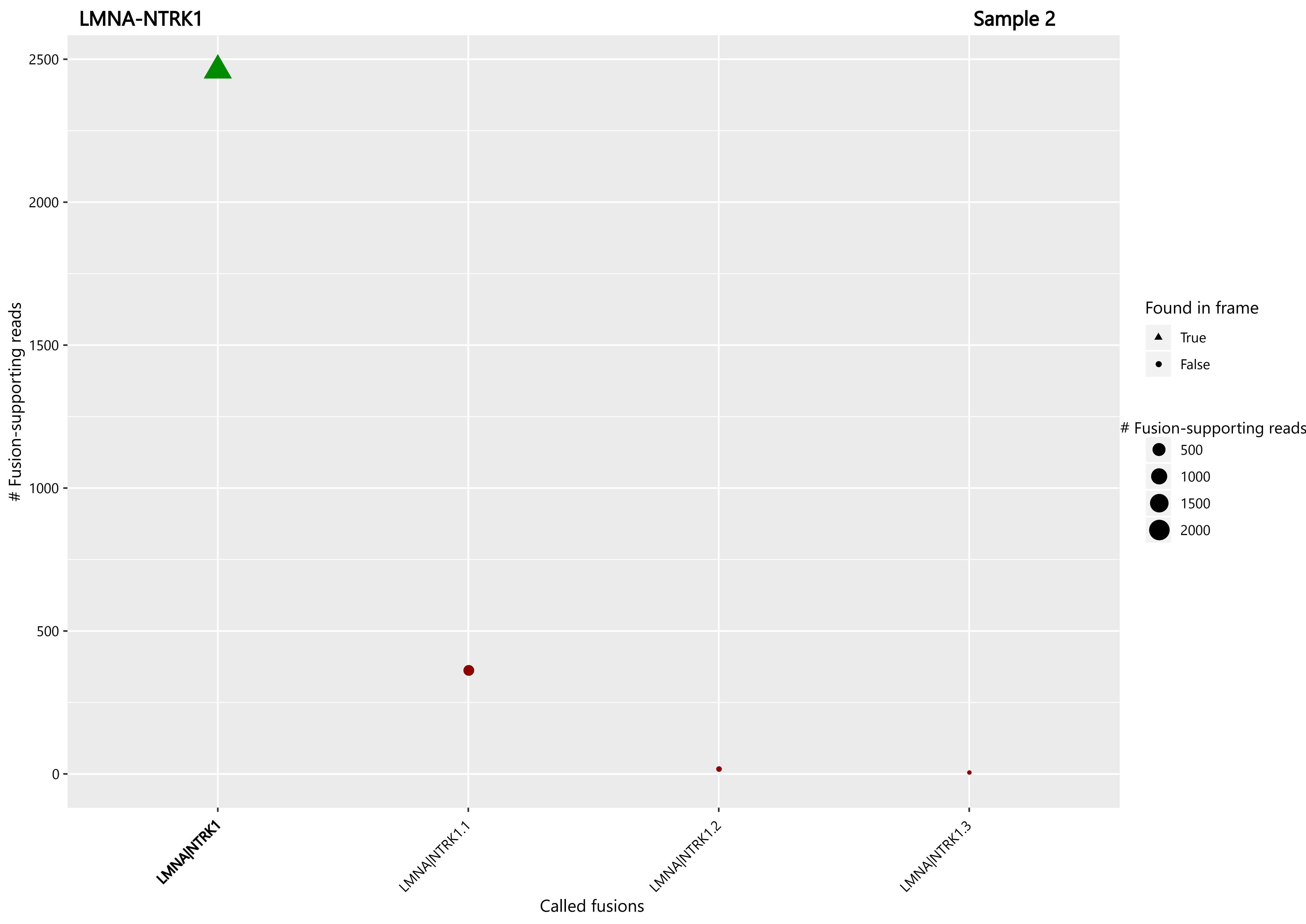

LMNA-NTRK1
Sample 2
Fusion-supporting reads
2500
2000
1500
1000
500
0
LMNA|NTRK1
LMNA|NTRK1.1
LMNA|NTRK1.2
LMNA|NTRK1.3
Called fusions
Found in frame
True
False
Fusion-supporting reads
500
1000
1500
2000

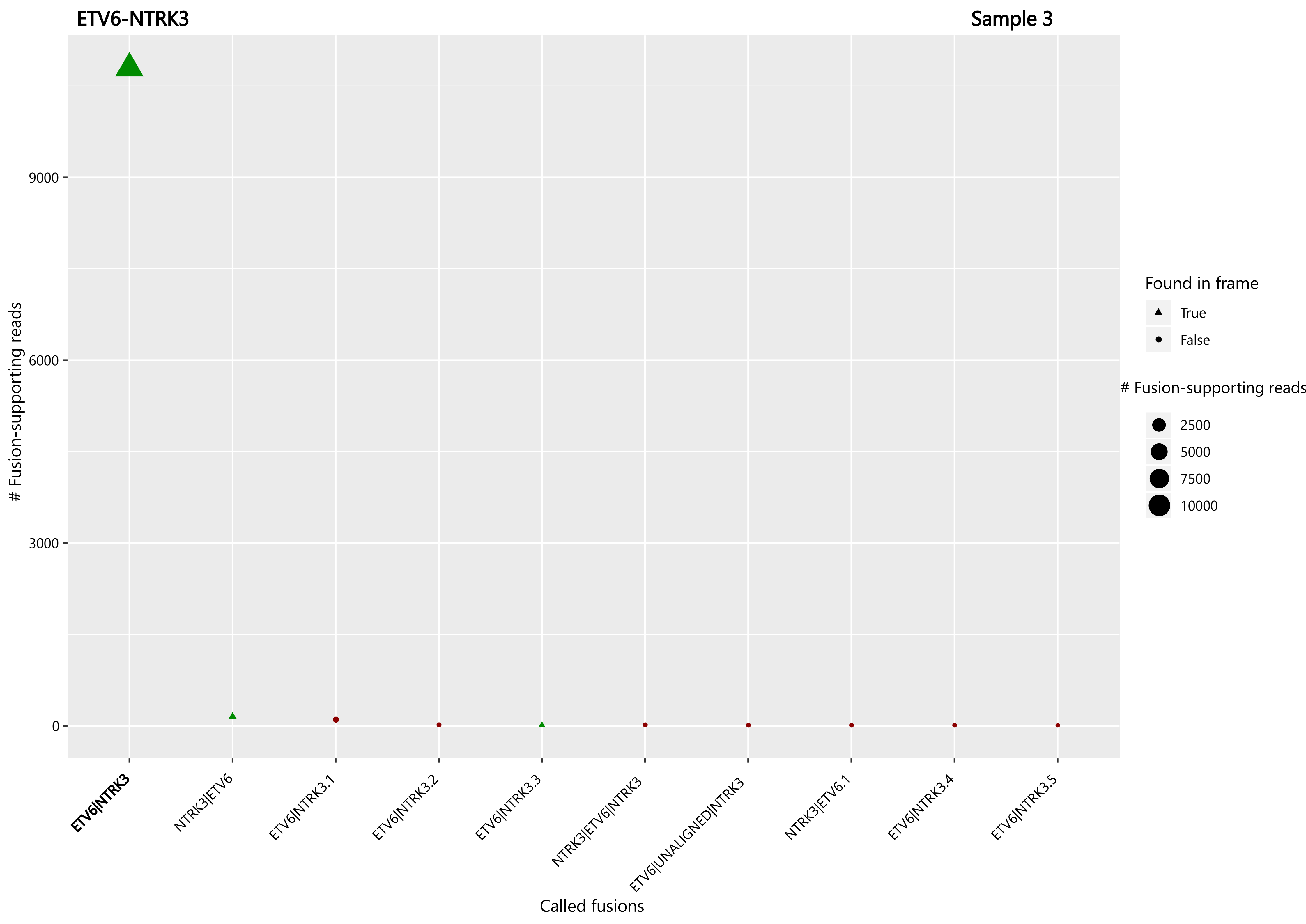

ETV6-NTRK3
Sample 3
Fusion-supporting reads
9000
6000
3000
0
Called fusions
ETV6|NTRK3
NTRK3|ETV6
ETV6|NTRK3.1
ETV6|NTRK3.2
ETV6|NTRK3.3
NTRK3|ETV6|NTRK3
ETV6|UNALIGNED|NTRK3
NTRK3|ETV6.1
ETV6|NTRK3.4
ETV6|NTRK3.5
Found in frame
True
False
Fusion-supporting reads
2500
5000
7500
10000

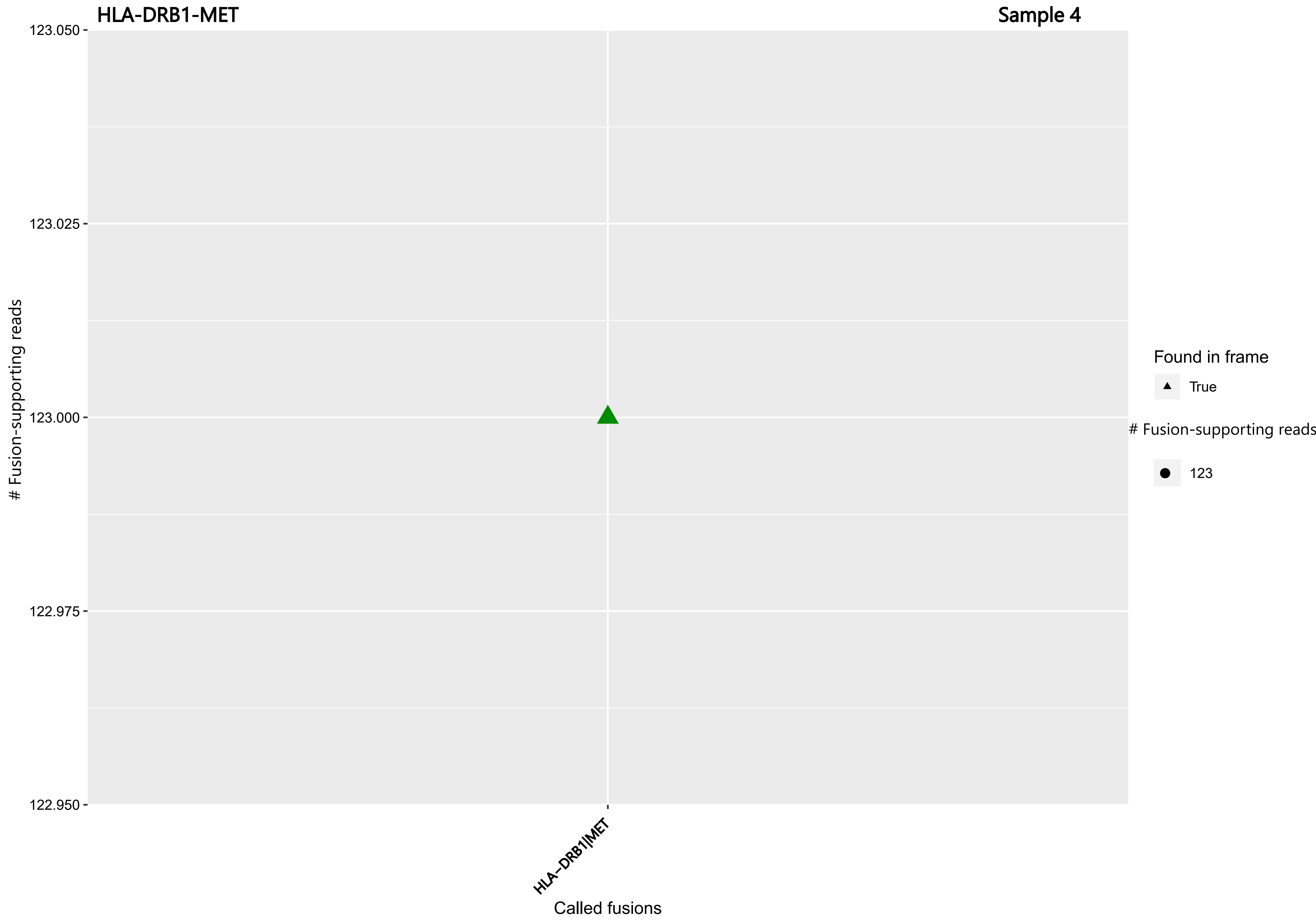
HLA-DRB1-MET
Sample 4
123.050
123.025
123.000
122.975
122.950
Fusion-supporting reads
HLA−DRB1|MET
Called fusions
Found in frame
True
Fusion-supporting reads
123

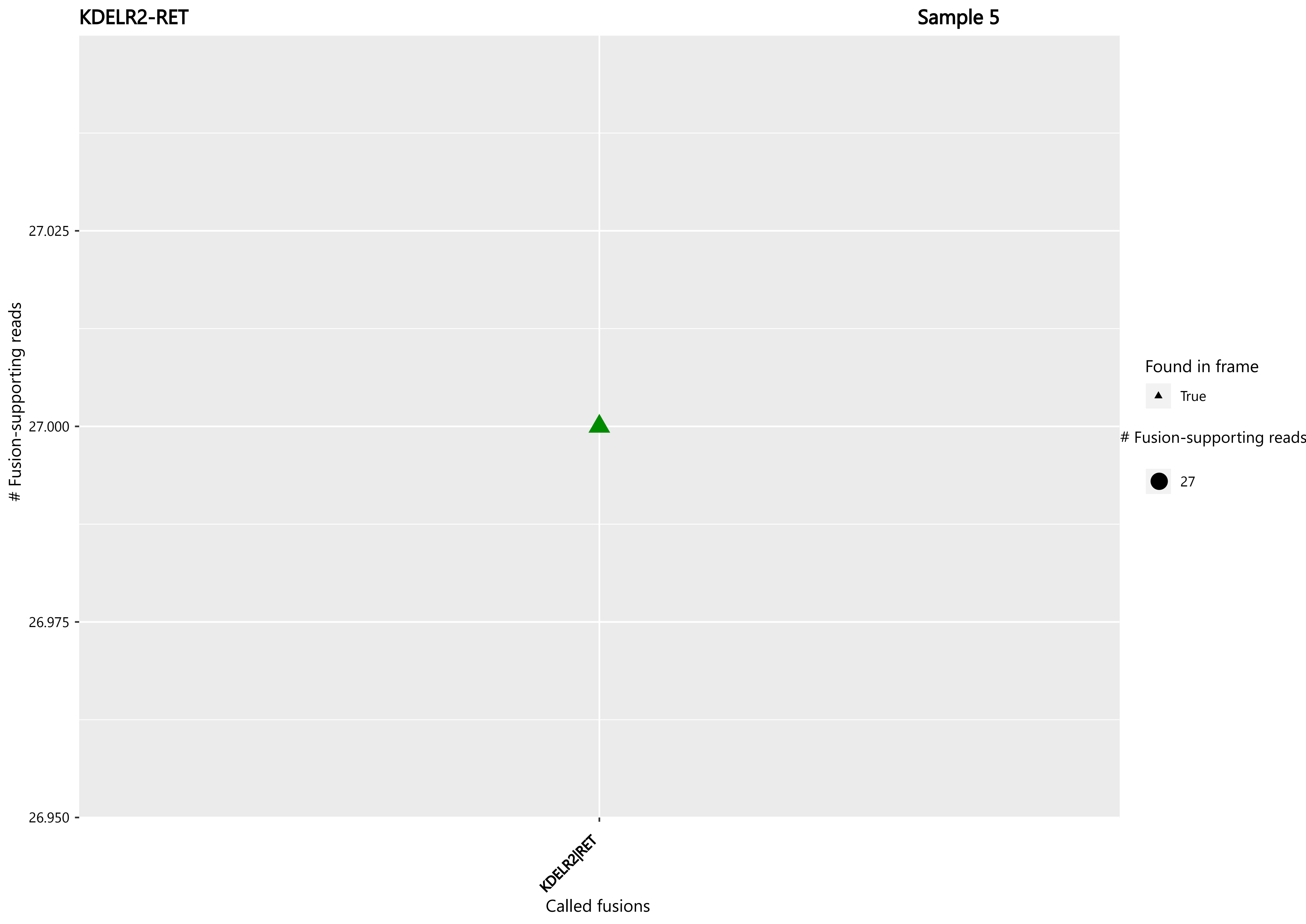
KDELR2-RET
Sample 5
Fusion-supporting reads
27.025
27.000
26.975
26.950
KDELR2|RET
Called fusions
Found in frame
True
Fusion-supporting reads
27

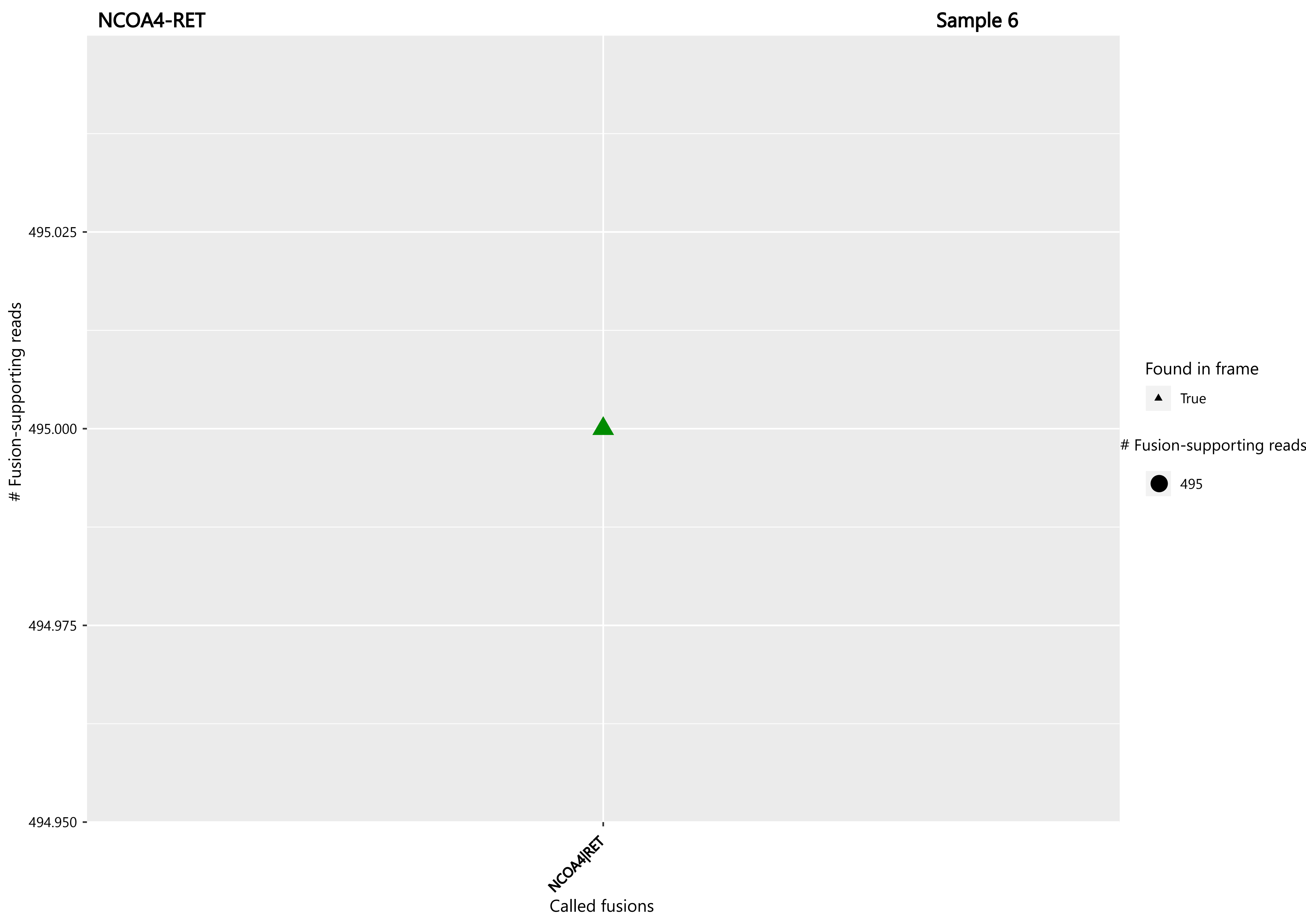
NCOA4-RET
Sample 6
Fusion-supporting reads
495.025
495.000
494.975
494.950
NCOA4|RET
Called fusions
Found in frame
True
Fusion-supporting reads
495

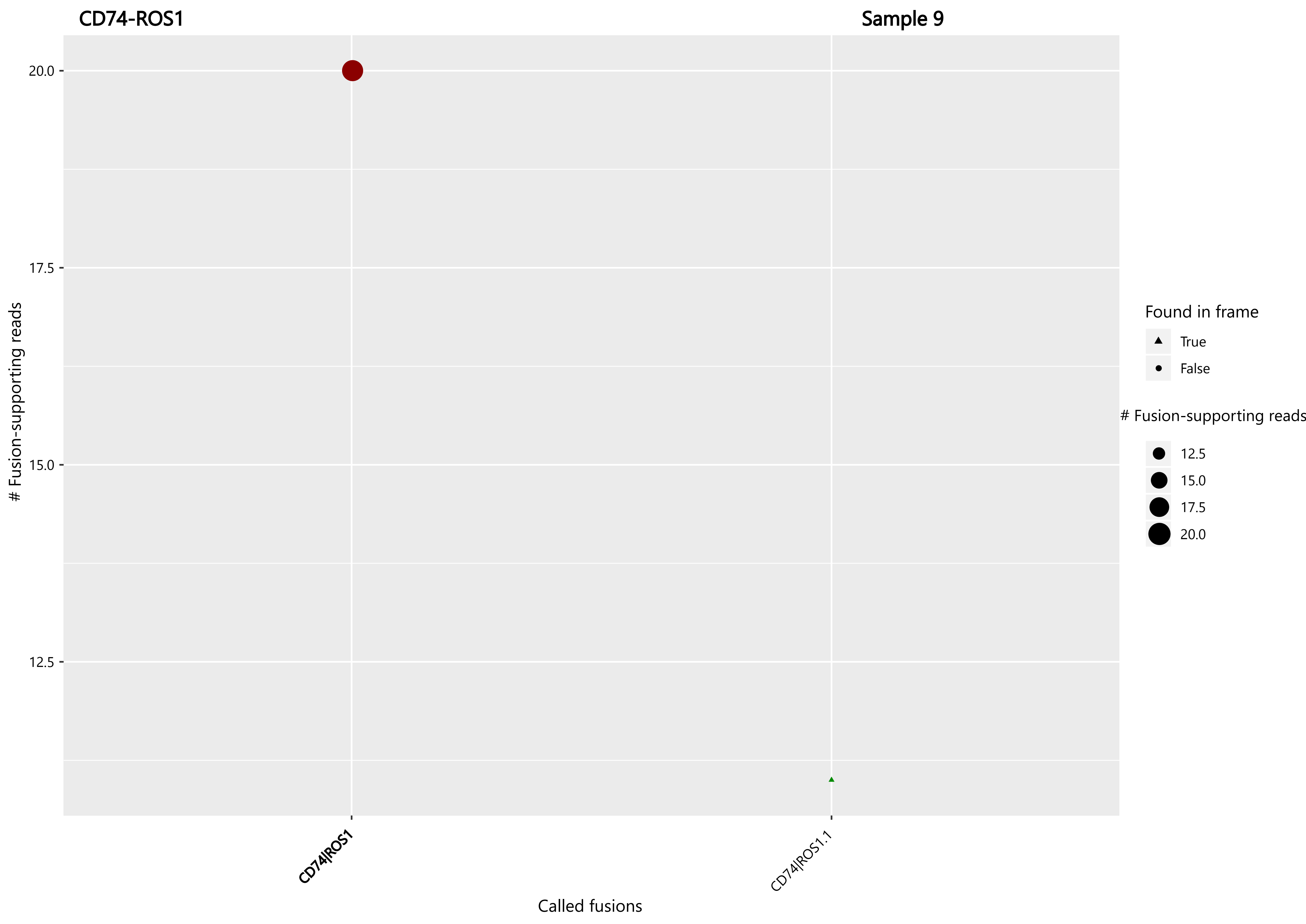
CD74-ROS1
Sample 9
20.0
17.5
15.0
12.5
Fusion-supporting reads
Found in frame
True
False
Fusion-supporting reads
12.5
15.0
17.5
20.0
CD74|ROS1
CD74|ROS1.1
Called fusions

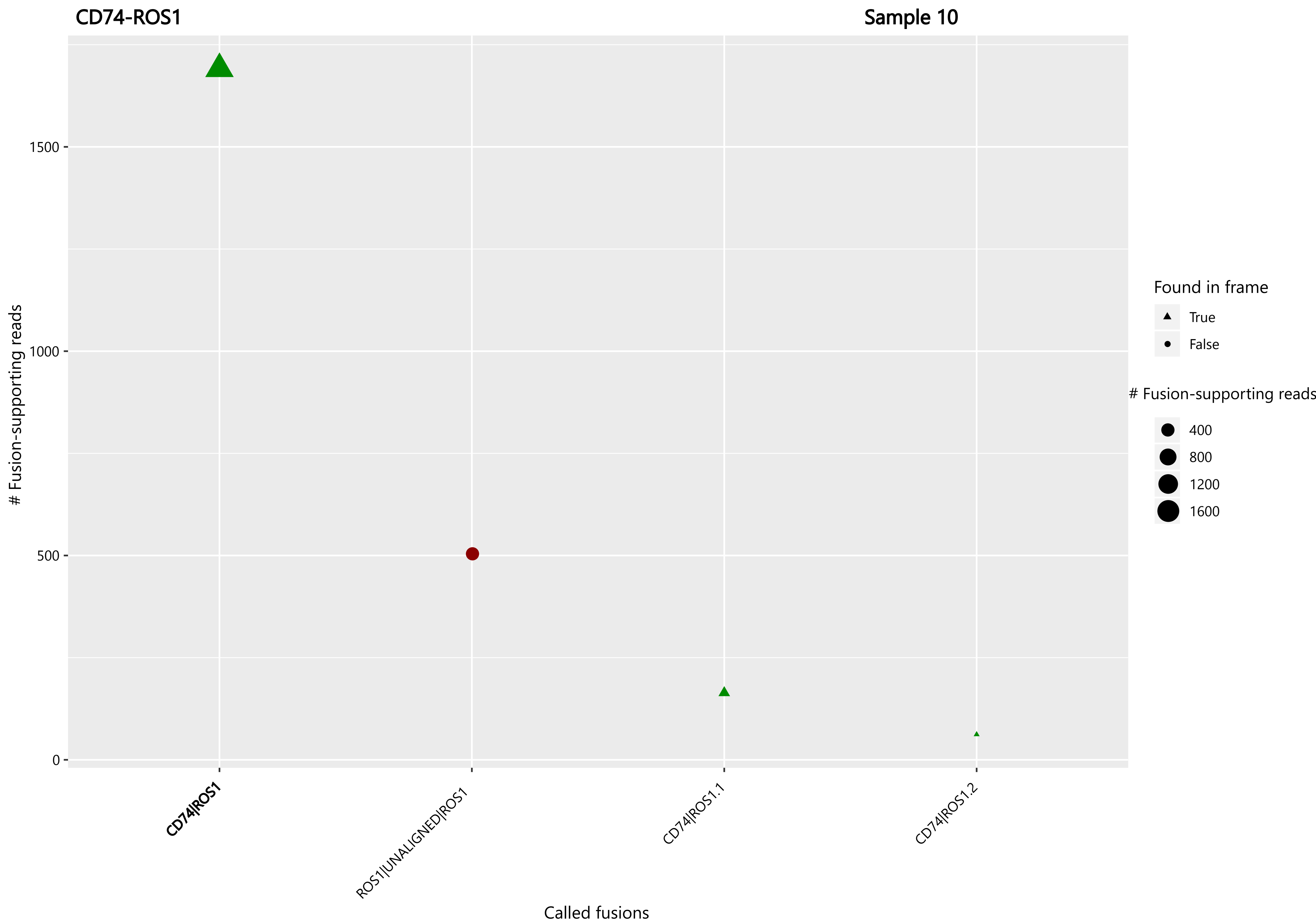
CD74-ROS1
Sample 10
Fusion-supporting reads
1500
1000
500
0
Called fusions
CD74|ROS1
ROS1|UNALIGNED|ROS1
CD74|ROS1.1
CD74|ROS1.2
Found in frame
True
False
Fusion-supporting reads
400
800
1200
1600

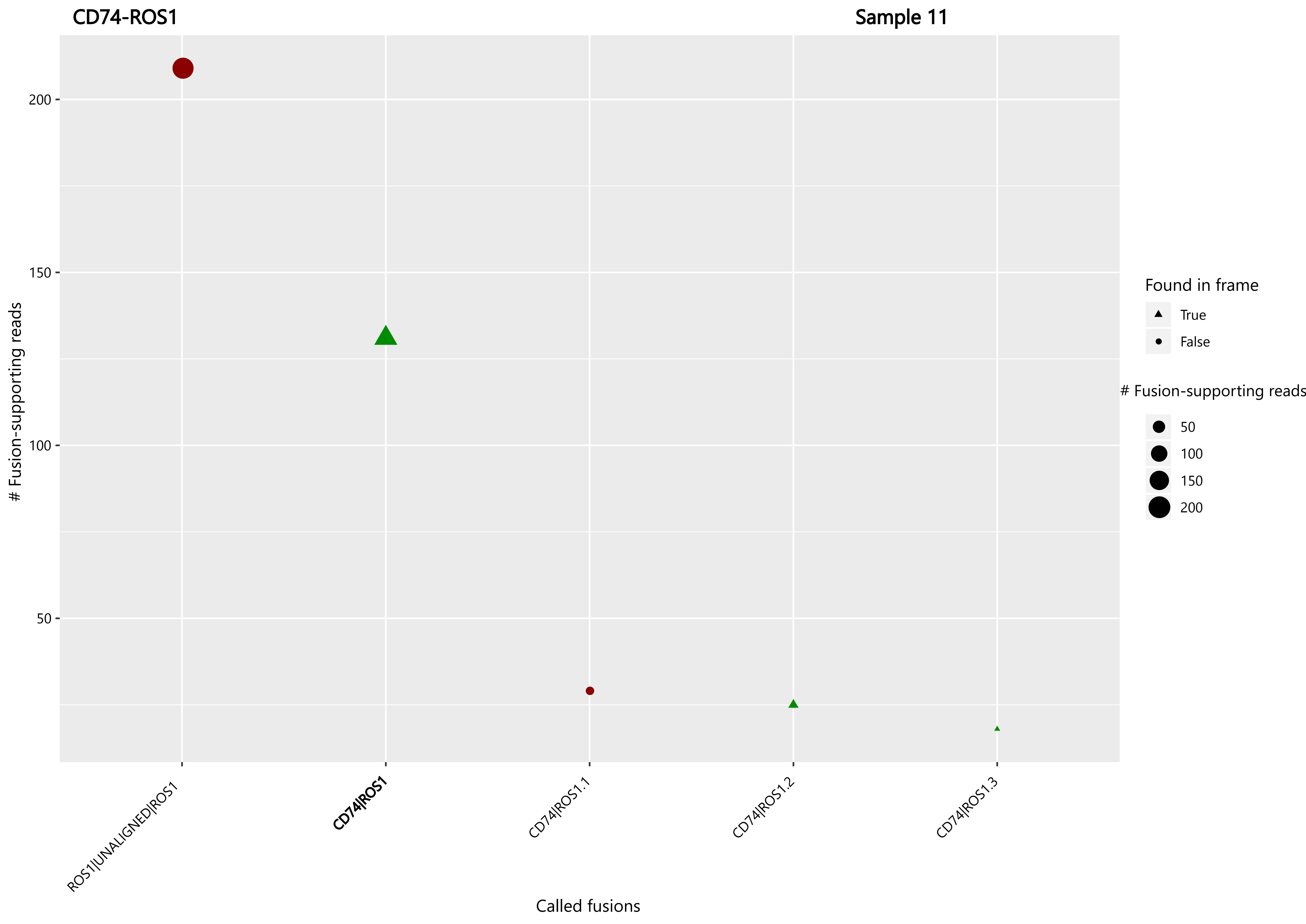

CD74-ROS1
Sample 11
200
150
100
50
Fusion-supporting reads
ROS1|UNALIGNED|ROS1
CD74|ROS1
CD74|ROS1.1
CD74|ROS1.2
CD74|ROS1.3
Called fusions
Found in frame
True
False
Fusion-supporting reads
50
100
150
200

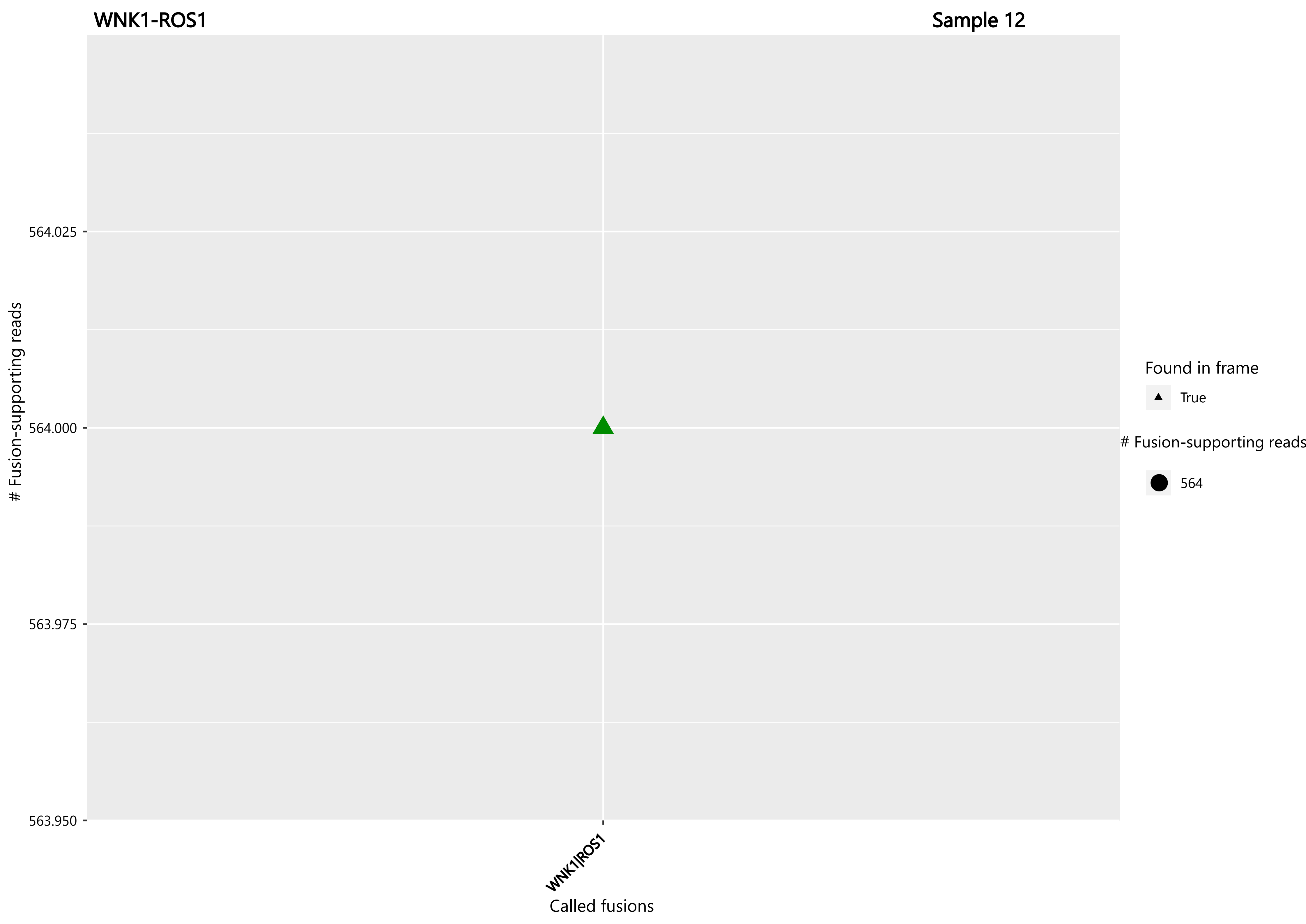

WNK1-ROS1
Sample 12
Fusion-supporting reads
564.025
564.000
563.975
563.950
WNK1|ROS1
Called fusions
Found in frame
True
Fusion-supporting reads
564

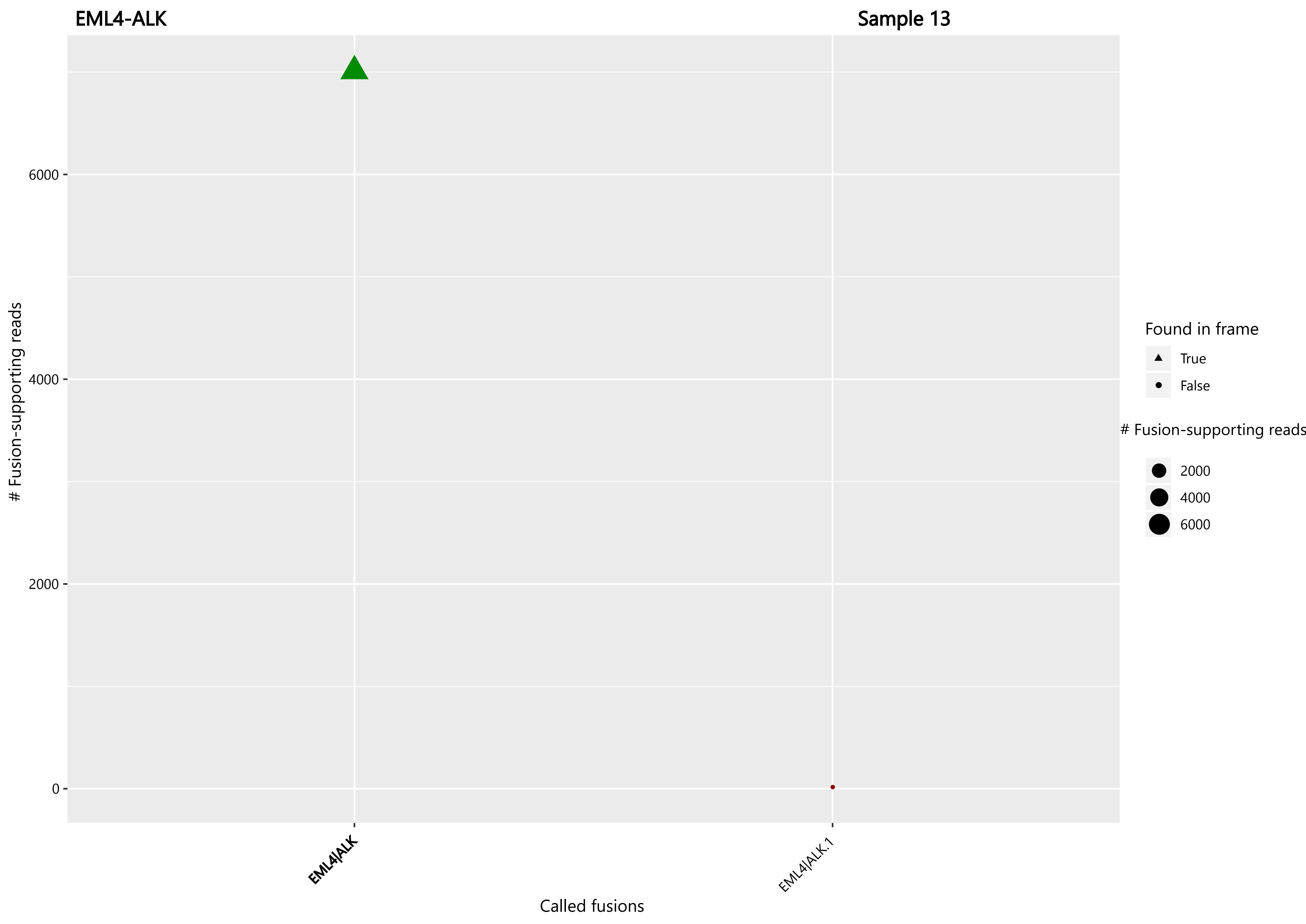
EML4-ALK
Sample 13
Fusion-supporting reads
6000
4000
2000
0
EML4|ALK
EML4|ALK.1
Called fusions
Found in frame
True
False
Fusion-supporting reads
2000
4000
6000

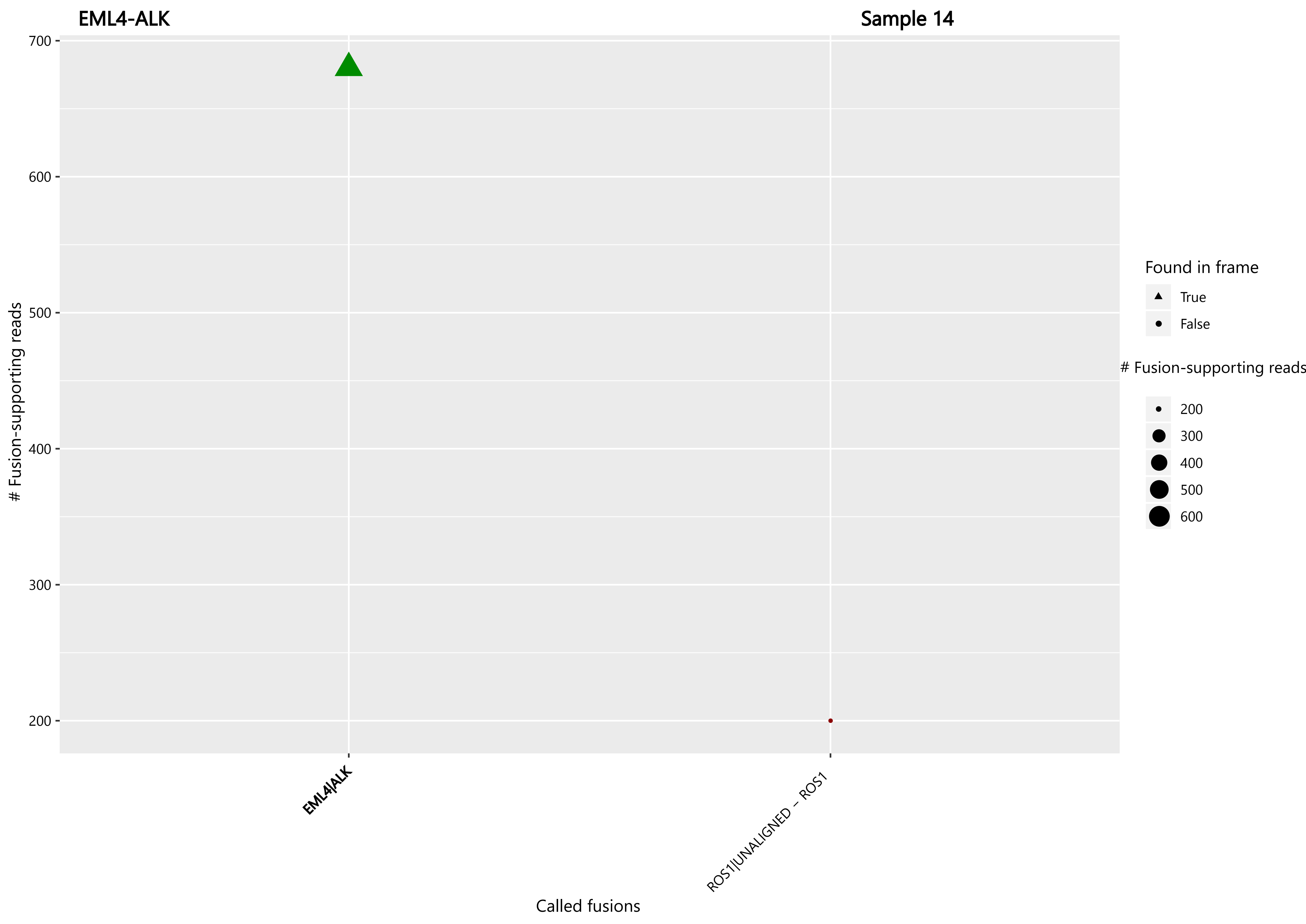
EML4-ALK
Sample 14
Fusion-supporting reads
700
600
500
400
300
200
Called fusions
EML4|ALK
ROS1|UNALIGNED – ROS1
Found in frame
True
False
Fusion-supporting reads
200
300
400
500
600

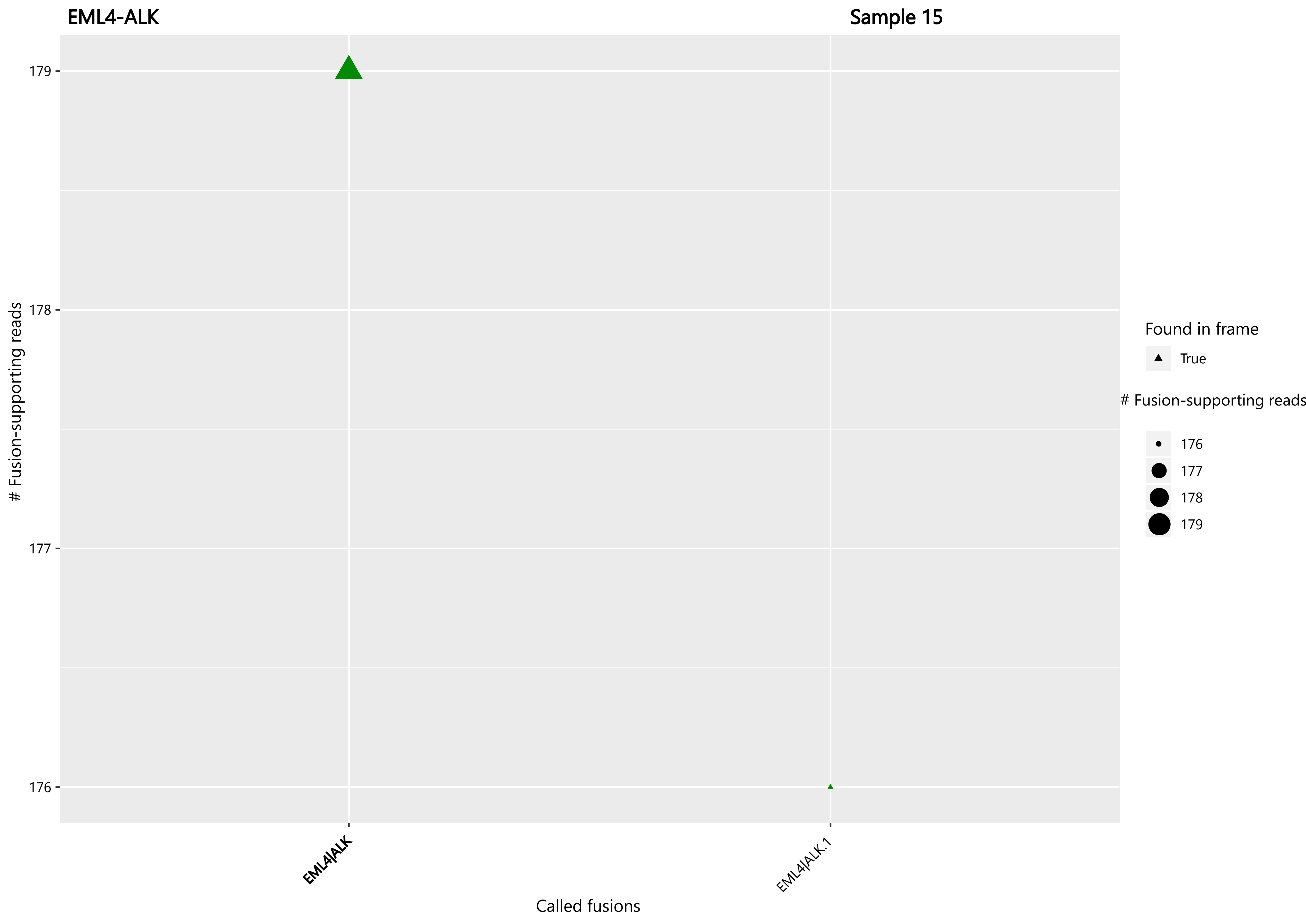
EML4-ALK
Sample 15
179
178
177
176
Fusion-supporting reads
EML4|ALK
EML4|ALK.1
Called fusions
Found in frame
True
Fusion-supporting reads
176
177
178
179

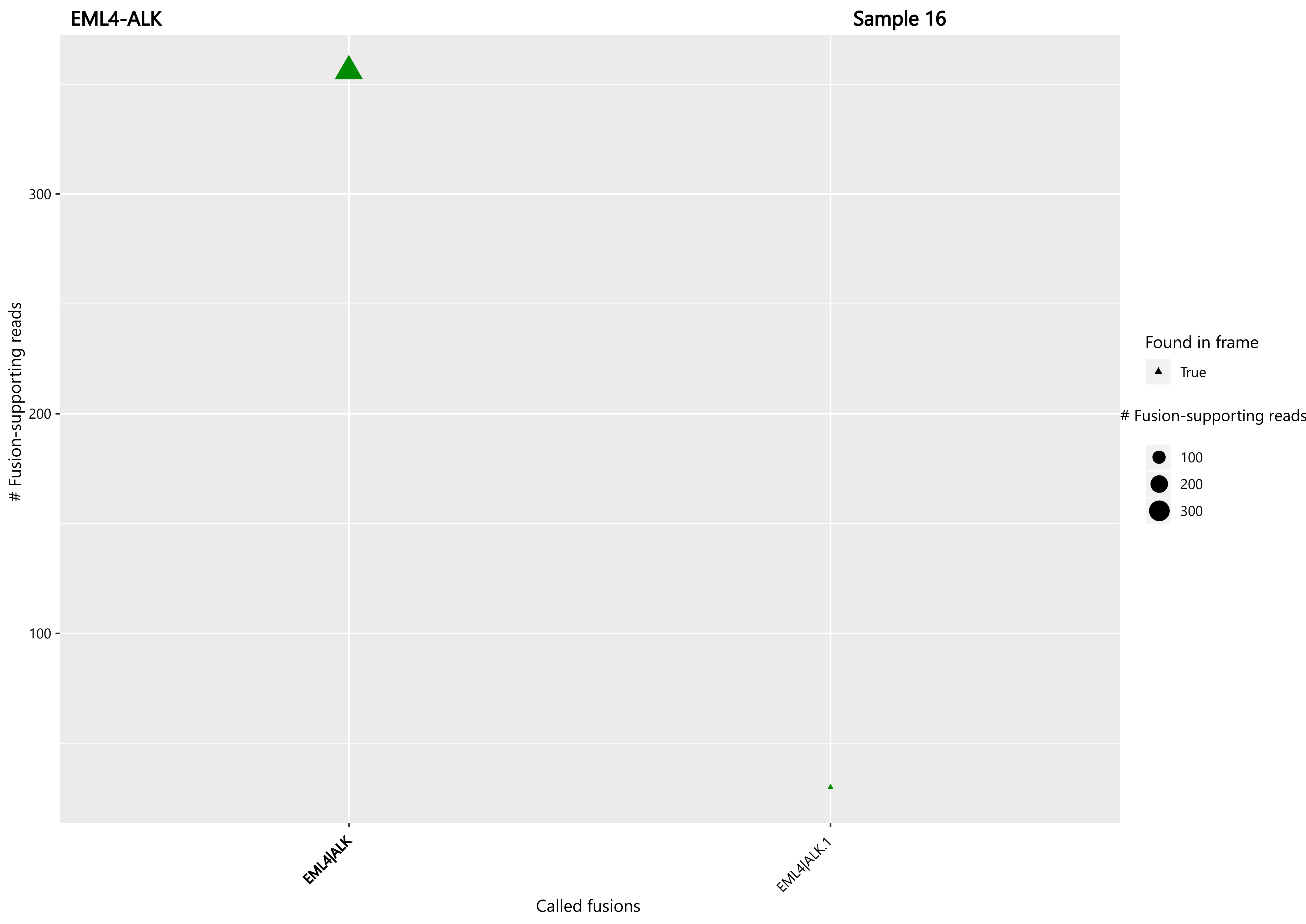
EML4-ALK
Sample 16
300
200
100
Fusion-supporting reads
EML4|ALK
EML4|ALK.1
Called fusions
Found in frame
True
Fusion-supporting reads
100
200
300

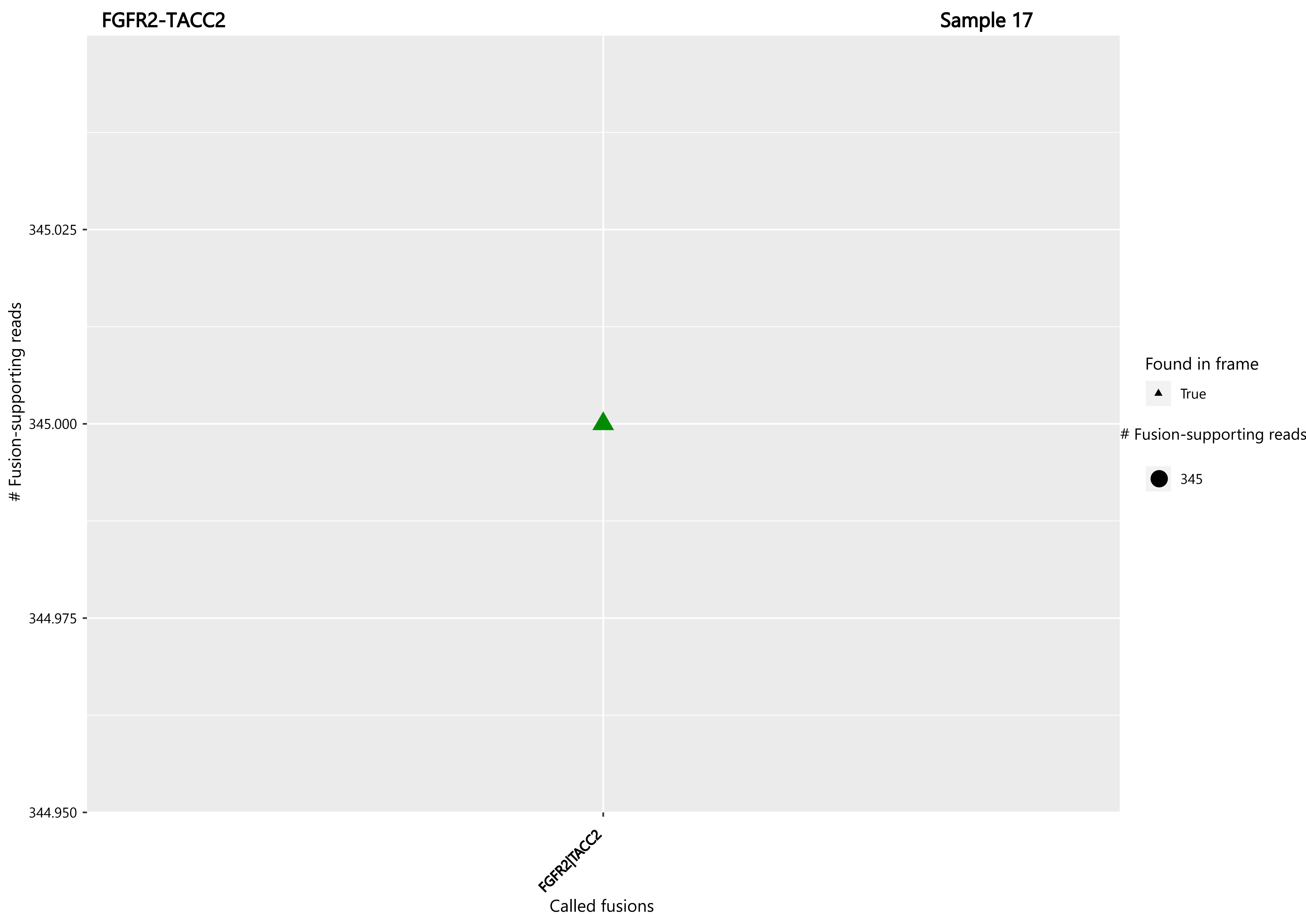

FGFR2-TACC2
Sample 17
Fusion-supporting reads
345.025
345.000
344.975
344.950
FGFR2|TACC2
Called fusions
Found in frame
True
Fusion-supporting reads
345

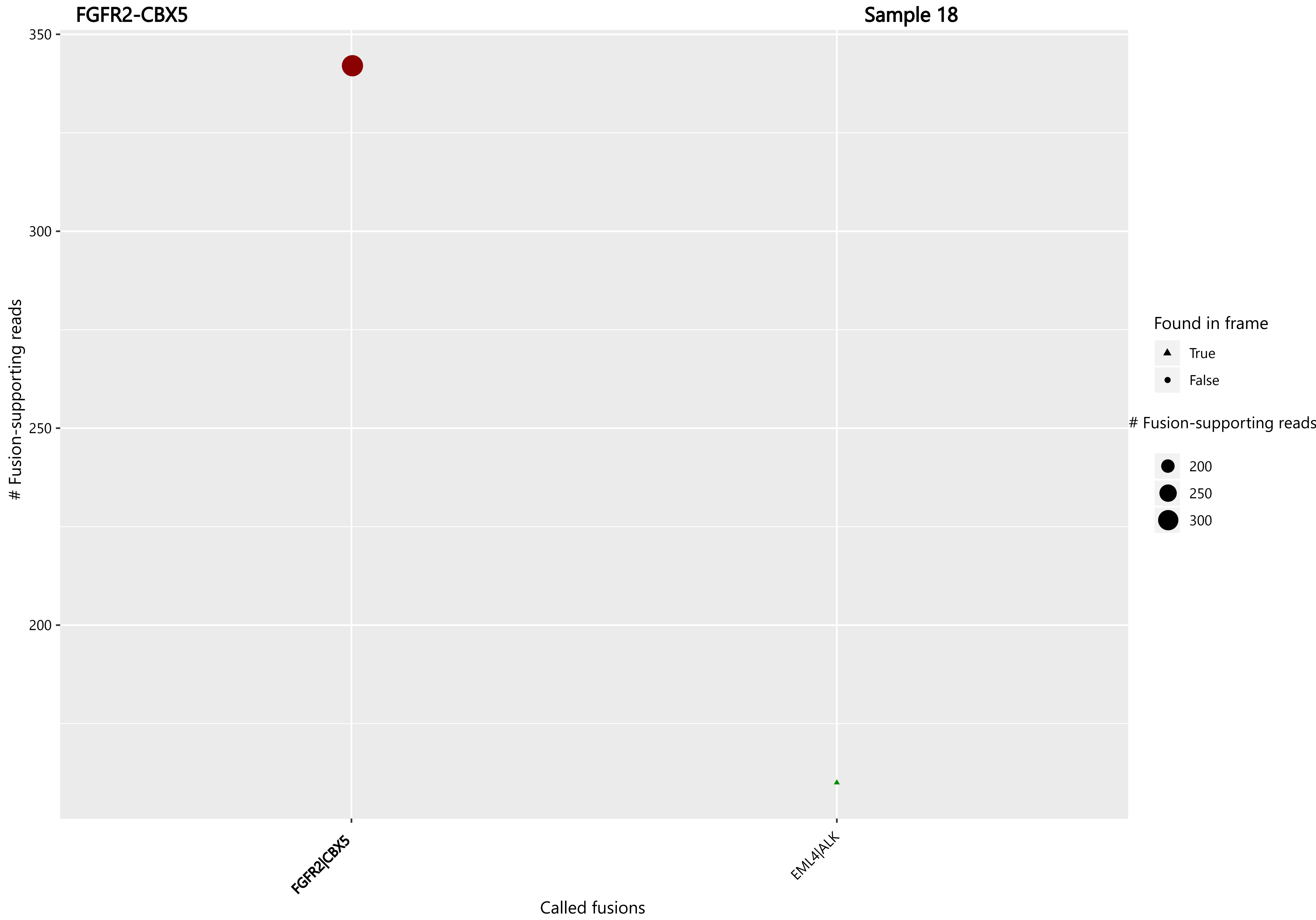
FGFR2-CBX5
Sample 18
350
300
250
200
Fusion-supporting reads
FGFR2|CBX5
EML4|ALK
Called fusions
Found in frame
True
False
Fusion-supporting reads
200
250
300
