# Supplementary material for: Detection of gene fusions using targeted next-generation sequencing: a comparative evaluation
Source: BMC Med Genomics. 2021 Feb 27;14:62. doi: 10.1186/s12920-021-00909-y (PMC7912891; doi:10.1186/s12920-021-00909-y)
Supplement: Supplementary file 12 — Additional file 12: Fig. S12. Fusions detected with the QIAseq Targeted RNAscan Custom Panel (Qiagen) for all samples. Metrics such as quality control scores, in-frame status or filter thresholds were plotted when available. In cases where the same fusion was identified more than once within the same sample, a unique numbering scheme was added at the end of the name to differentiate the candidate fusions. The software already provides the results with an ordering scheme with the most likely fusion being “Fusion 1”. The expected fusion for each sample was highlighted in bold. [file 12920_2021_909_MOESM12_ESM.pdf]

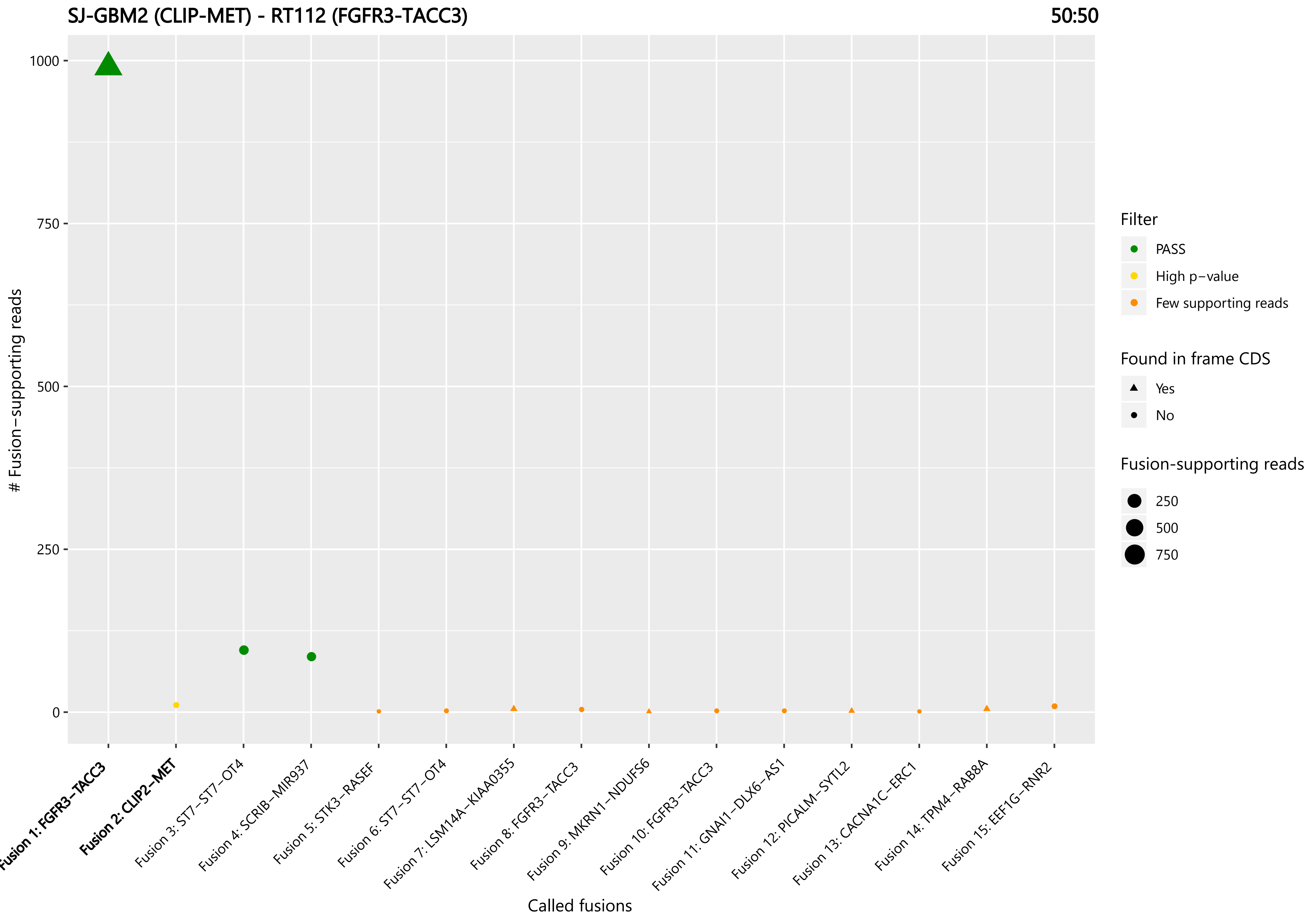

SJ-GBM2 (CLIP-MET) - RT112 (FGFR3-TACC3)
50:50
1000
750
500
250
0
Fusion−supporting reads
Fusion 1: FGFR3−TACC3
Fusion 2: CLIP2−MET
Fusion 3: ST7−ST7−OT4
Fusion 4: SCRIB−MIR937
Fusion 5: STK3−RASEF
Fusion 6: ST7−ST7−OT4
Fusion 7: LSM14A−KIAA0355
Fusion 8: FGFR3−TACC3
Fusion 9: MKRN1−NDUFS6
Fusion 10: FGFR3−TACC3
Fusion 11: GNAI1−DLX6−AS1
Fusion 12: PICALM−SYTL2
Fusion 13: CACNA1C−ERC1
Fusion 14: TPM4−RAB8A
Fusion 15: EEF1G−RNR2
Called fusions
Filter
PASS
High p−value
Few supporting reads
Found in frame CDS
Yes
No
Fusion-supporting reads
250
500
750

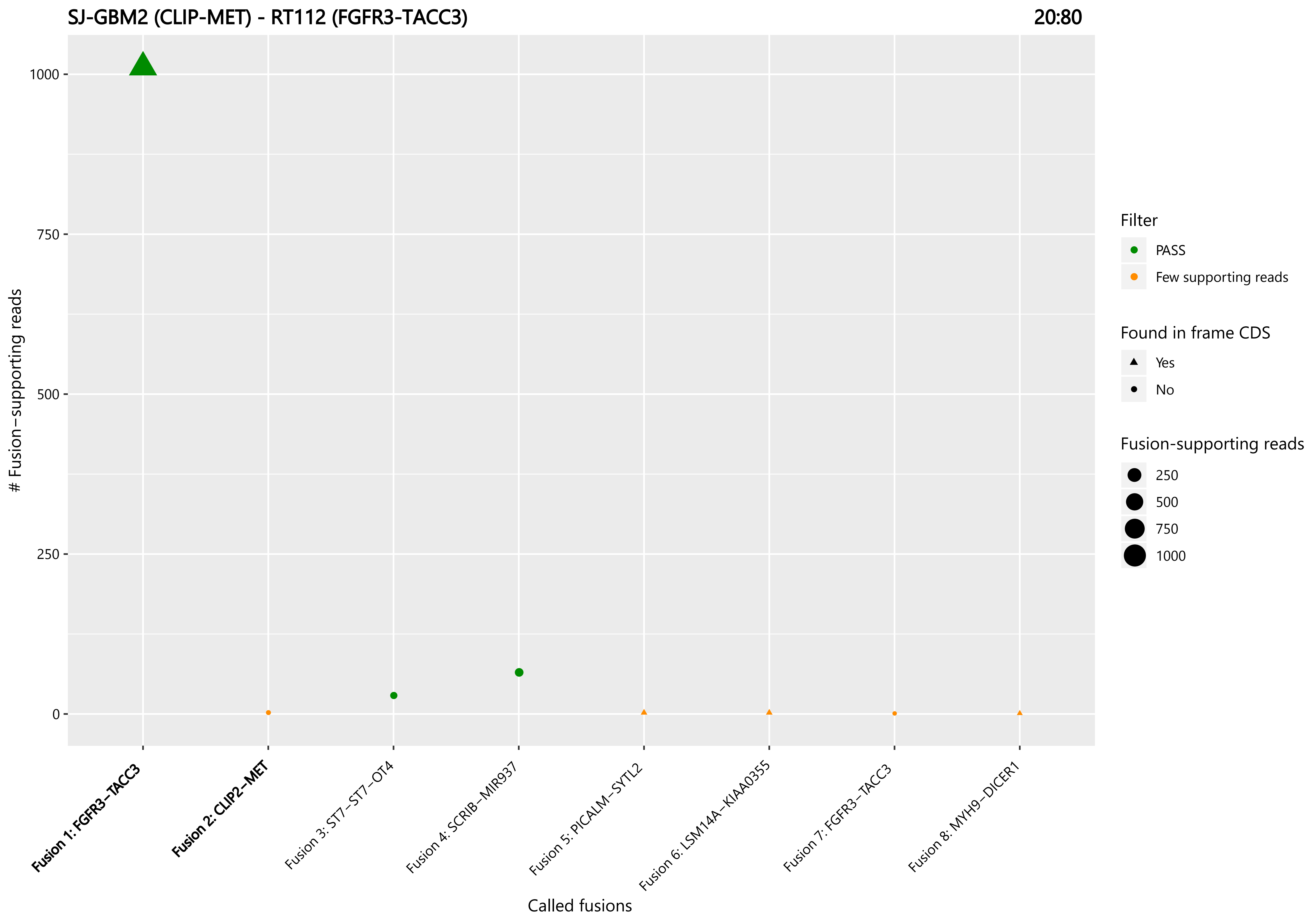

SJ-GBM2 (CLIP-MET) - RT112 (FGFR3-TACC3)
20:80
1000
750
500
250
0
Fusion−supporting reads
Fusion 1: FGFR3−TACC3
Fusion 2: CLIP2−MET
Fusion 3: ST7−ST7−OT4
Fusion 4: SCRIB−MIR937
Fusion 5: PICALM−SYTL2
Fusion 6: LSM14A−KIAA0355
Fusion 7: FGFR3−TACC3
Fusion 8: MYH9−DICER1
Called fusions
Filter
PASS
Few supporting reads
Found in frame CDS
Yes
No
Fusion-supporting reads
250
500
750
1000

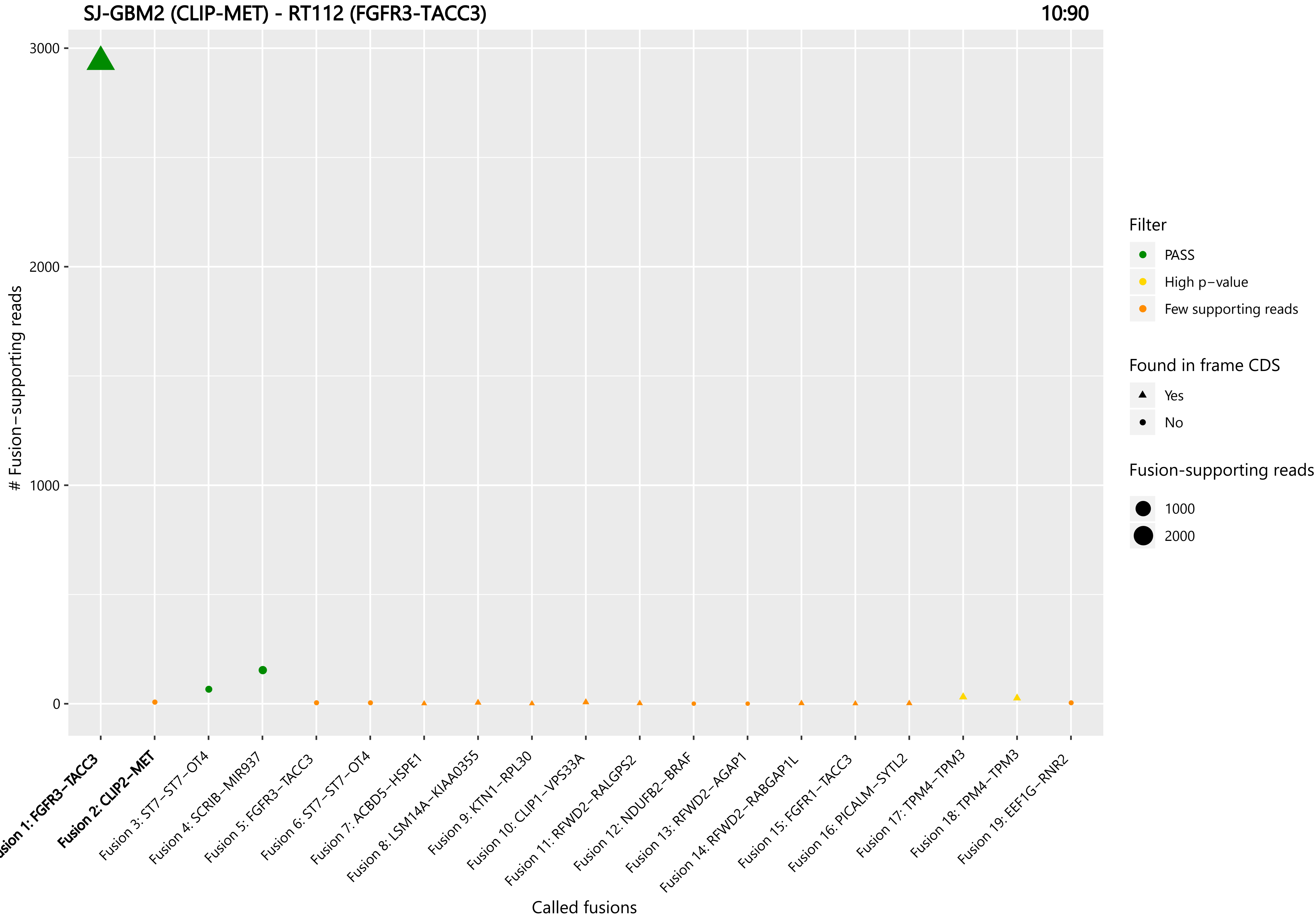

SJ-GBM2 (CLIP-MET) - RT112 (FGFR3-TACC3)
10:90
Fusion−supporting reads
3000
2000
1000
0
Called fusions
Fusion 1: FGFR3−TACC3
Fusion 2: CLIP2−MET
Fusion 3: ST7−ST7−OT4
Fusion 4: SCRIB−MIR937
Fusion 5: FGFR3−TACC3
Fusion 6: ST7−ST7−OT4
Fusion 7: ACBD5−HSPE1
Fusion 8: LSM14A−KIAA0355
Fusion 9: KTN1−RPL30
Fusion 10: CLIP1−VPS33A
Fusion 11: RFWD2−RALGPS2
Fusion 12: NDUFB2−BRAF
Fusion 13: RFWD2−AGAP1
Fusion 14: RFWD2−RABGAP1L
Fusion 15: FGFR1−TACC3
Fusion 16: PICALM−SYTL2
Fusion 17: TPM4−TPM3
Fusion 18: TPM4−TPM3
Fusion 19: EEF1G−RNR2
Filter
PASS
High p−value
Few supporting reads
Found in frame CDS
Yes
No
Fusion-supporting reads
1000
2000

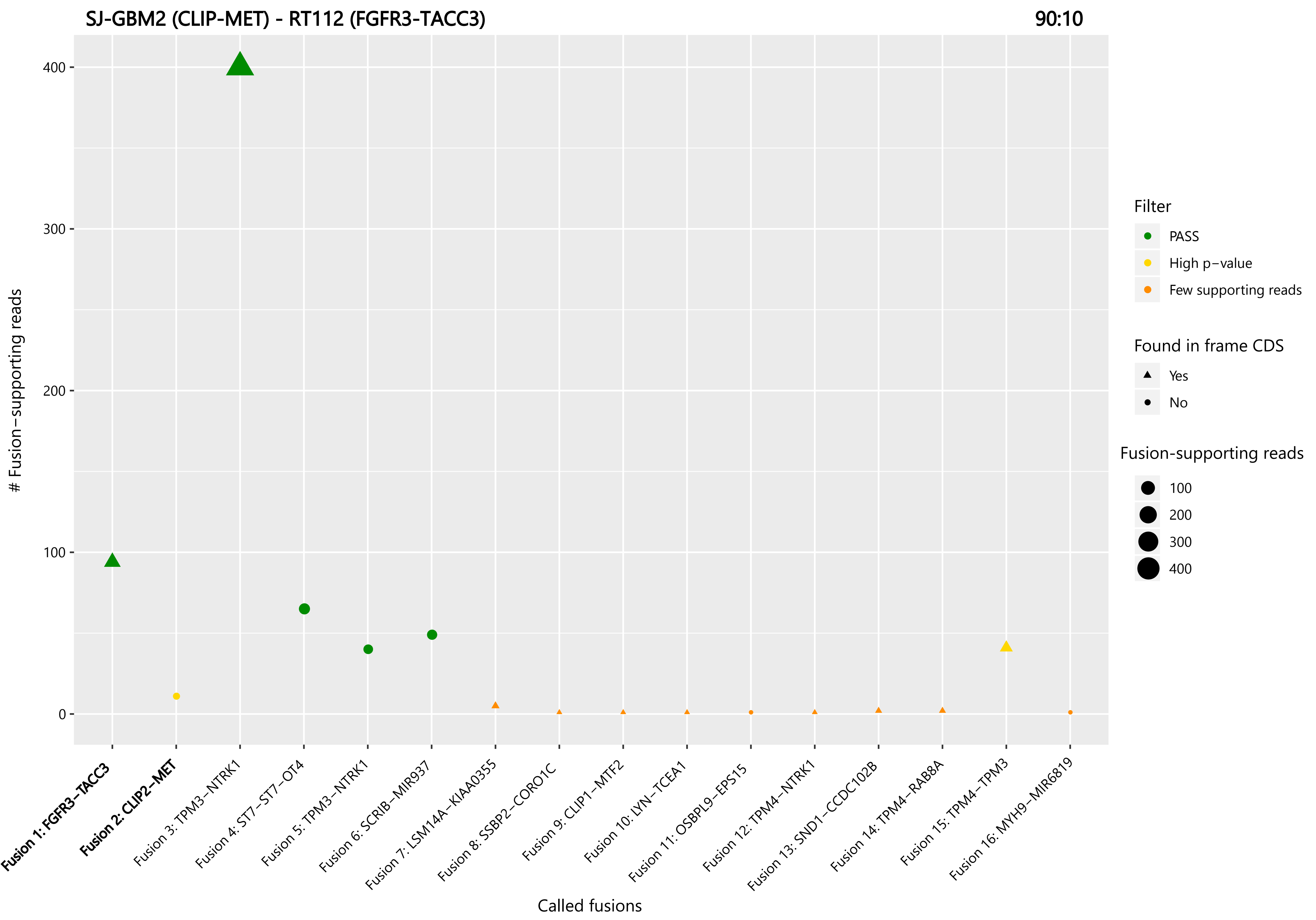
SJ-GBM2 (CLIP-MET) - RT112 (FGFR3-TACC3)
90:10
Fusion−supporting reads
0
100
200
300
400
Called fusions
Fusion 1: FGFR3−TACC3
Fusion 2: CLIP2−MET
Fusion 3: TPM3−NTRK1
Fusion 4: ST7−ST7−OT4
Fusion 5: TPM3−NTRK1
Fusion 6: SCRIB−MIR937
Fusion 7: LSM14A−KIAA0355
Fusion 8: SSBP2−CORO1C
Fusion 9: CLIP1−MTF2
Fusion 10: LYN−TCEA1
Fusion 11: OSBPL9−EPS15
Fusion 12: TPM4−NTRK1
Fusion 13: SND1−CCDC102B
Fusion 14: TPM4−RAB8A
Fusion 15: TPM4−TPM3
Fusion 16: MYH9−MIR6819
Filter
PASS
High p−value
Few supporting reads
Found in frame CDS
Yes
No
Fusion-supporting reads
100
200
300
400

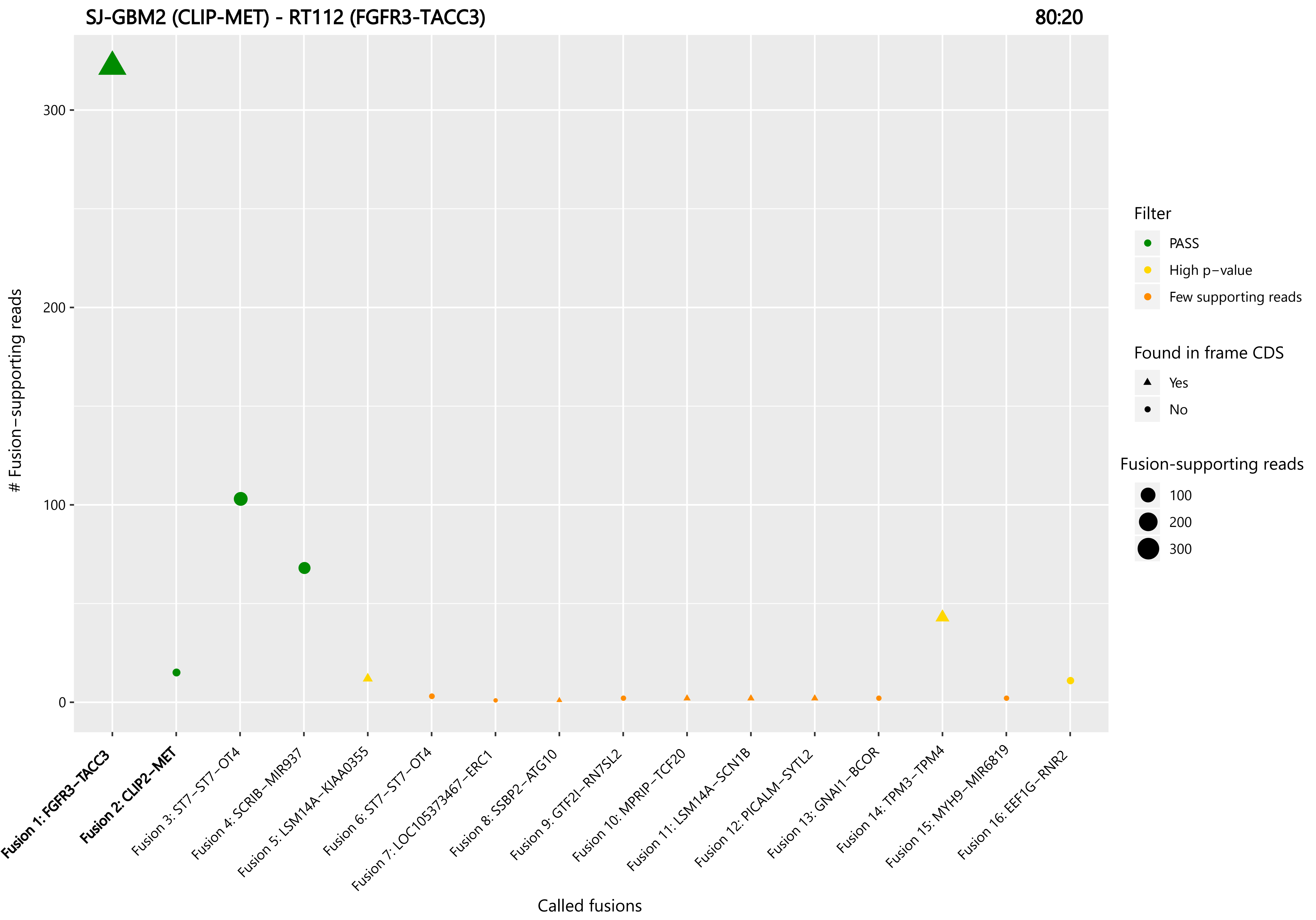

SJ-GBM2 (CLIP-MET) - RT112 (FGFR3-TACC3)
80:20
Fusion−supporting reads
300
200
100
0
Called fusions
Fusion 1: FGFR3−TACC3
Fusion 2: CLIP2−MET
Fusion 3: ST7−ST7−OT4
Fusion 4: SCRIB−MIR937
Fusion 5: LSM14A−KIAA0355
Fusion 6: ST7−ST7−OT4
Fusion 7: LOC105373467−ERC1
Fusion 8: SSBP2−ATG10
Fusion 9: GTF2I−RN7SL2
Fusion 10: MPRIP−TCF20
Fusion 11: LSM14A−SCN1B
Fusion 12: PICALM−SYTL2
Fusion 13: GNAI1−BCOR
Fusion 14: TPM3−TPM4
Fusion 15: MYH9−MIR6819
Fusion 16: EEF1G−RNR2
Filter
PASS
High p−value
Few supporting reads
Found in frame CDS
Yes
No
Fusion-supporting reads
100
200
300

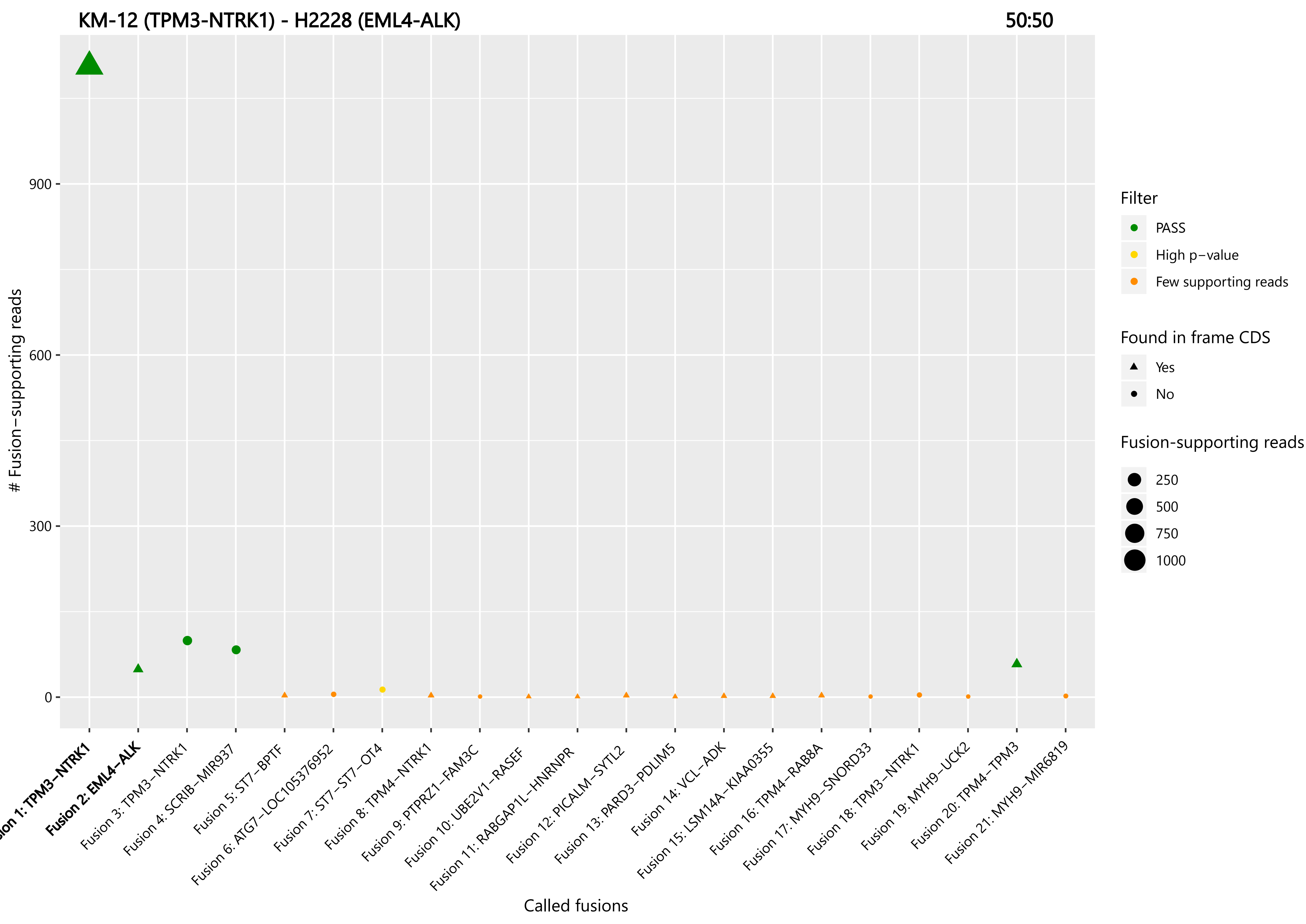

KM-12 (TPM3-NTRK1) - H2228 (EML4-ALK)
50:50
Fusion−supporting reads
900
600
300
0
Called fusions
Fusion 2: EML4−ALK
Fusion 3: TPM3−NTRK1
Fusion 4: SCRIB−MIR937
Fusion 5: ST7−BPTF
Fusion 6: ATG7−LOC105376952
Fusion 7: ST7−ST7−OT4
Fusion 8: TPM4−NTRK1
Fusion 9: PTPRZ1−FAM3C
Fusion 10: UBE2V1−RASEF
Fusion 11: RABGAP1L−HNRNPR
Fusion 12: PICALM−SYTL2
Fusion 13: PARD3−PDLIM5
Fusion 14: VCL−ADK
Fusion 15: LSM14A−KIAA0355
Fusion 16: TPM4−RAB8A
Fusion 17: MYH9−SNORD33
Fusion 18: TPM3−NTRK1
Fusion 19: MYH9−UCK2
Fusion 20: TPM4−TPM3
Fusion 21: MYH9−MIR6819
Filter
PASS
High p−value
Few supporting reads
Found in frame CDS
Yes
No
Fusion-supporting reads
250
500
750
1000

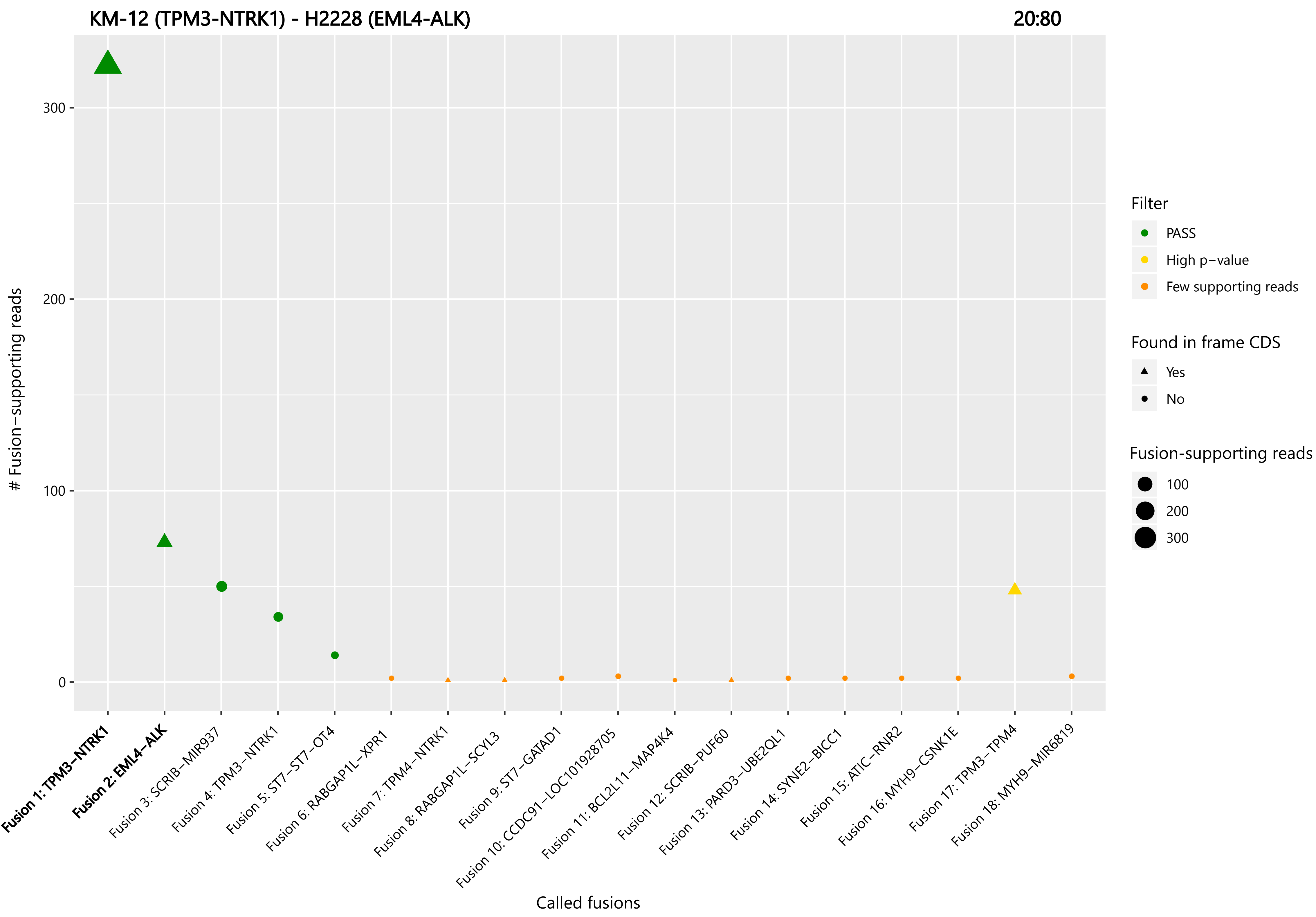
KM-12 (TPM3-NTRK1) - H2228 (EML4-ALK)
20:80
Fusion−supporting reads
300
200
100
0
Called fusions
Fusion 1: TPM3−NTRK1
Fusion 2: EML4−ALK
Fusion 3: SCRIB−MIR937
Fusion 4: TPM3−NTRK1
Fusion 5: ST7−ST7−OT4
Fusion 6: RABGAP1L−XPR1
Fusion 7: TPM4−NTRK1
Fusion 8: RABGAP1L−SCYL3
Fusion 9: ST7−GATAD1
Fusion 10: CCDC91−LOC101928705
Fusion 11: BCL2L11−MAP4K4
Fusion 12: SCRIB−PUF60
Fusion 13: PARD3−UBE2QL1
Fusion 14: SYNE2−BICC1
Fusion 15: ATIC−RNR2
Fusion 16: MYH9−CSNK1E
Fusion 17: TPM3−TPM4
Fusion 18: MYH9−MIR6819
Filter
PASS
High p−value
Few supporting reads
Found in frame CDS
Yes
No
Fusion-supporting reads
100
200
300

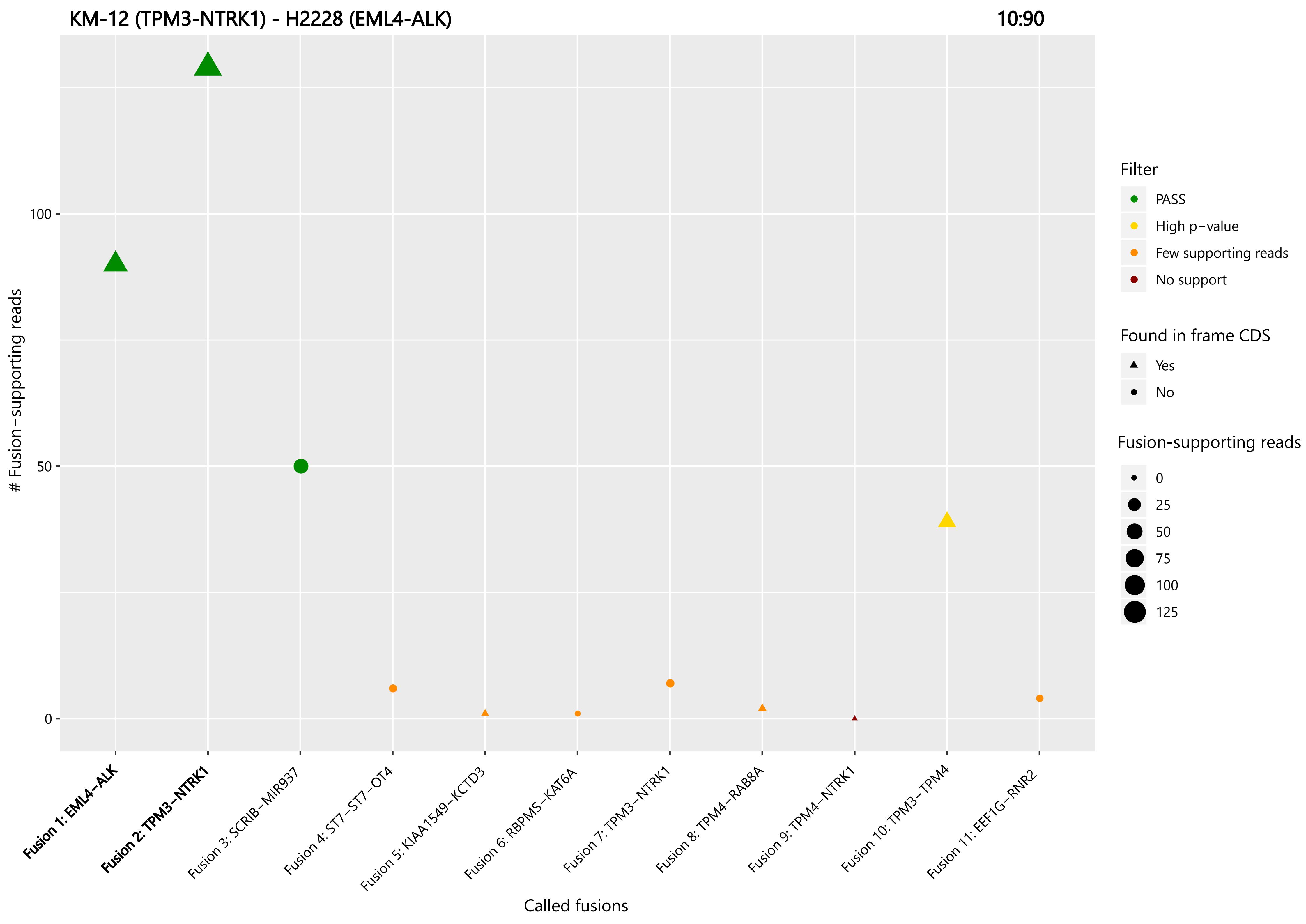
KM-12 (TPM3-NTRK1) - H2228 (EML4-ALK)
10:90
Fusion−supporting reads
100
50
0
Fusion 1: EML4−ALK
Fusion 2: TPM3−NTRK1
Fusion 3: SCRIB−MIR937
Fusion 4: ST7−ST7−OT4
Fusion 5: KIAA1549−KCTD3
Fusion 6: RBPMS−KAT6A
Fusion 7: TPM3−NTRK1
Fusion 8: TPM4−RAB8A
Fusion 9: TPM4−NTRK1
Fusion 10: TPM3−TPM4
Fusion 11: EEF1G−RNR2
Called fusions
Filter
PASS
High p−value
Few supporting reads
No support
Found in frame CDS
Yes
No
Fusion-supporting reads
0
25
50
75
100
125

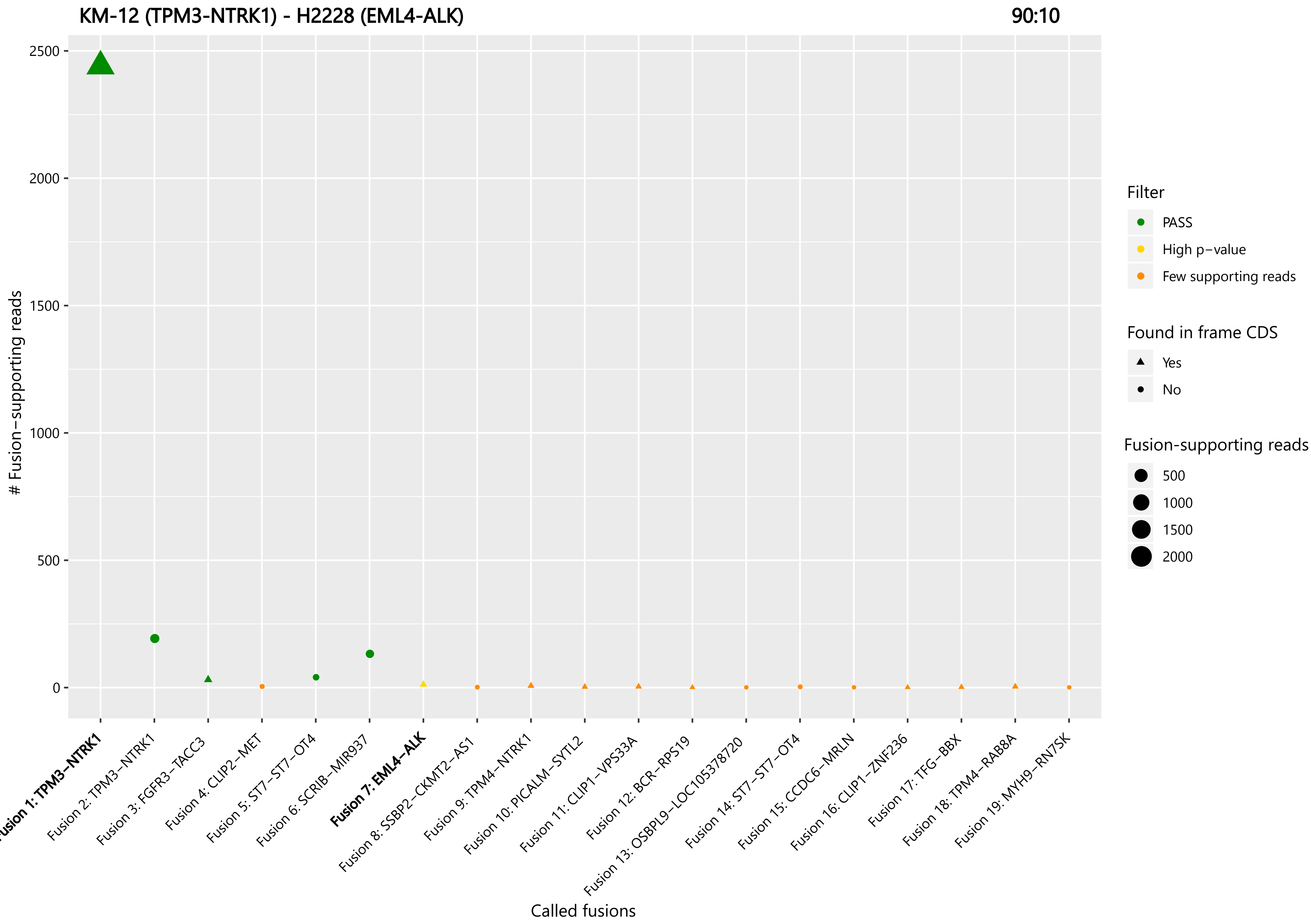

KM-12 (TPM3-NTRK1) - H2228 (EML4-ALK)
90:10
Fusion−supporting reads
2500
2000
1500
1000
500
0
Fusion 1: TPM3−NTRK1
Fusion 2: TPM3−NTRK1
Fusion 3: FGFR3−TACC3
Fusion 4: CLIP2−MET
Fusion 5: ST7−ST7−OT4
Fusion 6: SCRIB−MIR937
Fusion 7: EML4−ALK
Fusion 8: SSBP2−CKMT2−AS1
Fusion 9: TPM4−NTRK1
Fusion 10: PICALM−SYTL2
Fusion 11: CLIP1−VPS33A
Fusion 12: BCR−RPS19
Fusion 13: OSBPL9−LOC105378720
Fusion 14: ST7−ST7−OT4
Fusion 15: CCDC6−MRLN
Fusion 16: CLIP1−ZNF236
Fusion 17: TFG−BBX
Fusion 18: TPM4−RAB8A
Fusion 19: MYH9−RN7SK
Called fusions
Filter
PASS
High p−value
Few supporting reads
Found in frame CDS
Yes
No
Fusion-supporting reads
500
1000
1500
2000

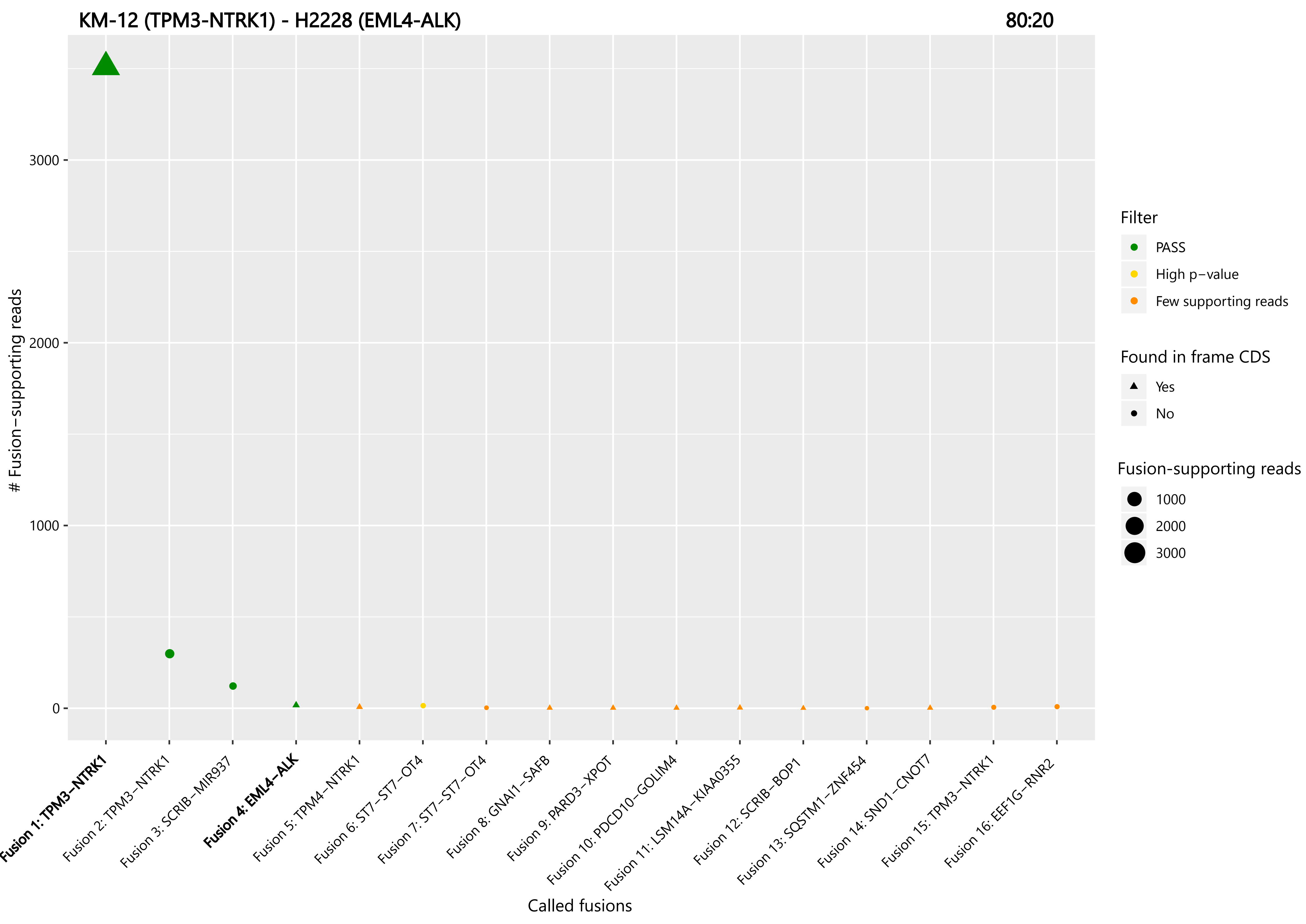

KM-12 (TPM3-NTRK1) - H2228 (EML4-ALK)
80:20
Fusion−supporting reads
3000
2000
1000
0
Called fusions
Fusion 1: TPM3−NTRK1
Fusion 2: TPM3−NTRK1
Fusion 3: SCRIB−MIR937
Fusion 4: EML4−ALK
Fusion 5: TPM4−NTRK1
Fusion 6: ST7−ST7−OT4
Fusion 7: ST7−ST7−OT4
Fusion 8: GNAI1−SAFB
Fusion 9: PARD3−XPOT
Fusion 10: PDCD10−GOLIM4
Fusion 11: LSM14A−KIAA0355
Fusion 12: SCRIB−BOP1
Fusion 13: SQSTM1−ZNF454
Fusion 14: SND1−CNOT7
Fusion 15: TPM3−NTRK1
Fusion 16: EEF1G−RNR2
Filter
PASS
High p−value
Few supporting reads
Found in frame CDS
Yes
No
Fusion-supporting reads
1000
2000
3000

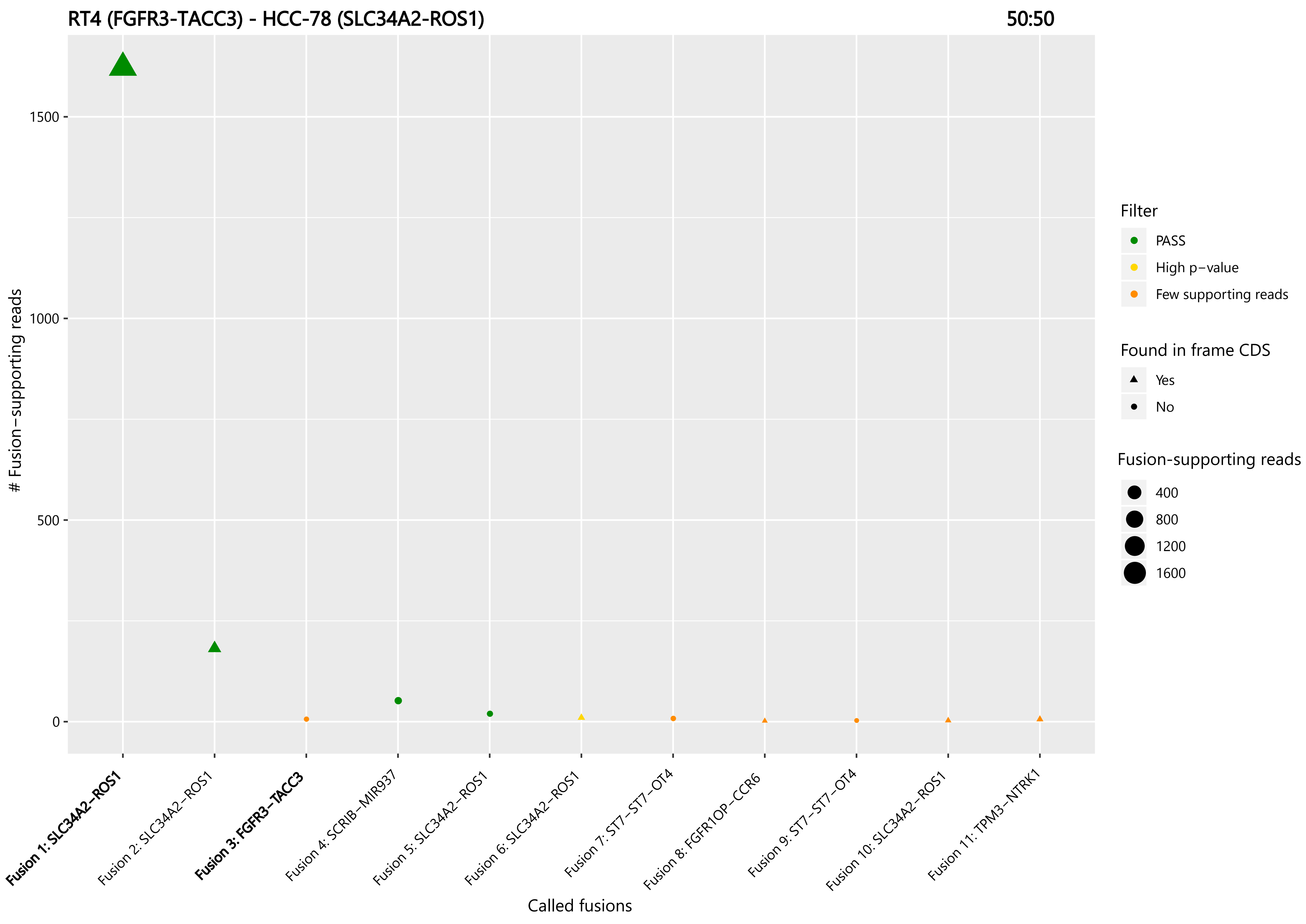
RT4 (FGFR3-TACC3) - HCC-78 (SLC34A2-ROS1)
50:50
Fusion−supporting reads
1500
1000
500
0
Called fusions
Fusion 1: SLC34A2−ROS1
Fusion 2: SLC34A2−ROS1
Fusion 3: FGFR3−TACC3
Fusion 4: SCRIB−MIR937
Fusion 5: SLC34A2−ROS1
Fusion 6: SLC34A2−ROS1
Fusion 7: ST7−ST7−OT4
Fusion 8: FGFR1OP−CCR6
Fusion 9: ST7−ST7−OT4
Fusion 10: SLC34A2−ROS1
Fusion 11: TPM3−NTRK1
Filter
PASS
High p−value
Few supporting reads
Found in frame CDS
Yes
No
Fusion-supporting reads
400
800
1200
1600

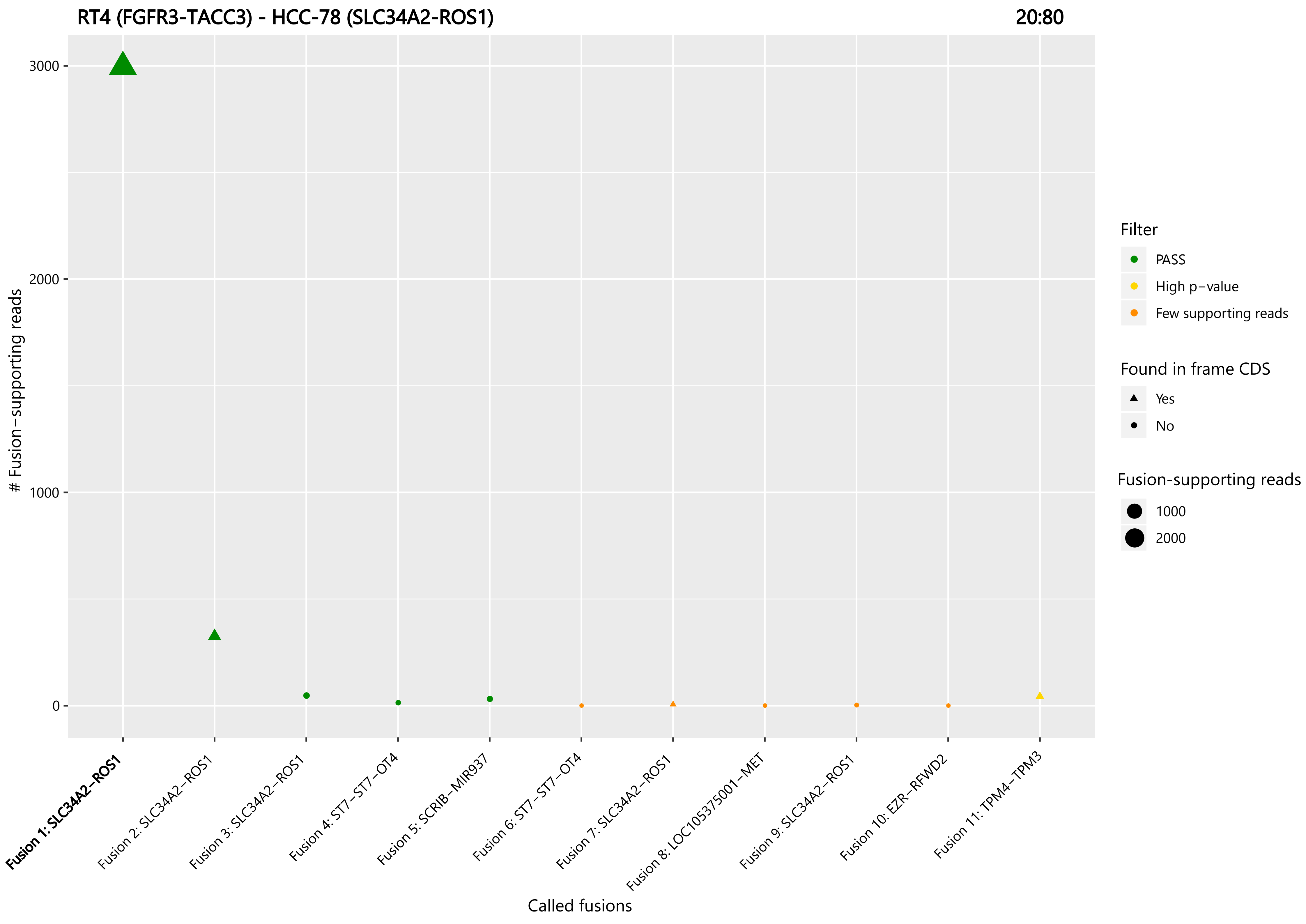

RT4 (FGFR3-TACC3) - HCC-78 (SLC34A2-ROS1)
20:80
Fusion−supporting reads
3000
2000
1000
0
Called fusions
Fusion 1: SLC34A2−ROS1
Fusion 2: SLC34A2−ROS1
Fusion 3: SLC34A2−ROS1
Fusion 4: ST7−ST7−OT4
Fusion 5: SCRIB−MIR937
Fusion 6: ST7−ST7−OT4
Fusion 7: SLC34A2−ROS1
Fusion 8: LOC105375001−MET
Fusion 9: SLC34A2−ROS1
Fusion 10: EZR−RFWD2
Fusion 11: TPM4−TPM3
Filter
PASS
High p−value
Few supporting reads
Found in frame CDS
Yes
No
Fusion-supporting reads
1000
2000

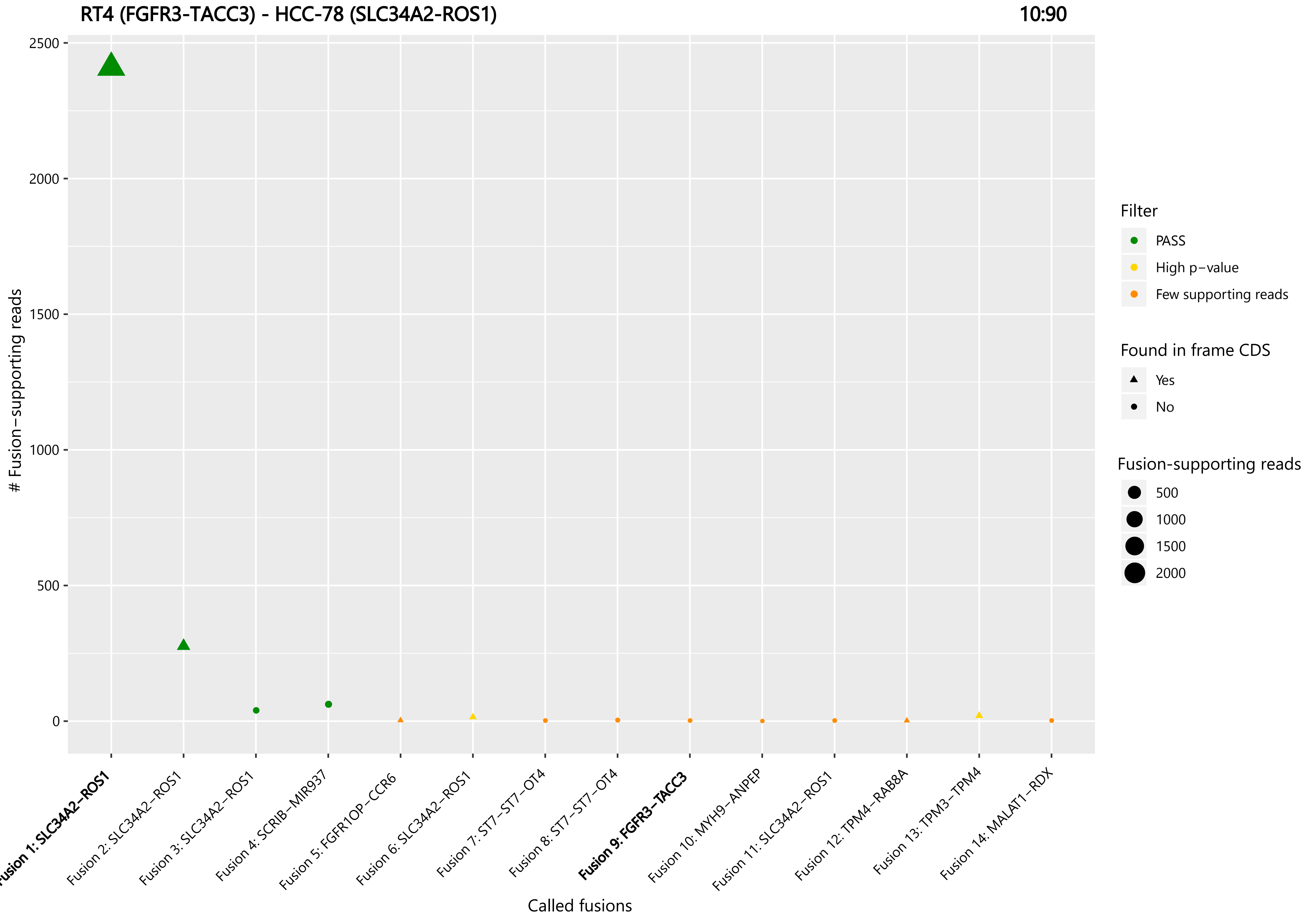
RT4 (FGFR3-TACC3) - HCC-78 (SLC34A2-ROS1)
10:90
Fusion−supporting reads
2500
2000
1500
1000
500
0
Called fusions
Fusion 1: SLC34A2−ROS1
Fusion 2: SLC34A2−ROS1
Fusion 3: SLC34A2−ROS1
Fusion 4: SCRIB−MIR937
Fusion 5: FGFR1OP−CCR6
Fusion 6: SLC34A2−ROS1
Fusion 7: ST7−ST7−OT4
Fusion 8: ST7−ST7−OT4
Fusion 9: FGFR3−TACC3
Fusion 10: MYH9−ANPEP
Fusion 11: SLC34A2−ROS1
Fusion 12: TPM4−RAB8A
Fusion 13: TPM3−TPM4
Fusion 14: MALAT1−RDX
Filter
PASS
High p−value
Few supporting reads
Found in frame CDS
Yes
No
Fusion-supporting reads
500
1000
1500
2000

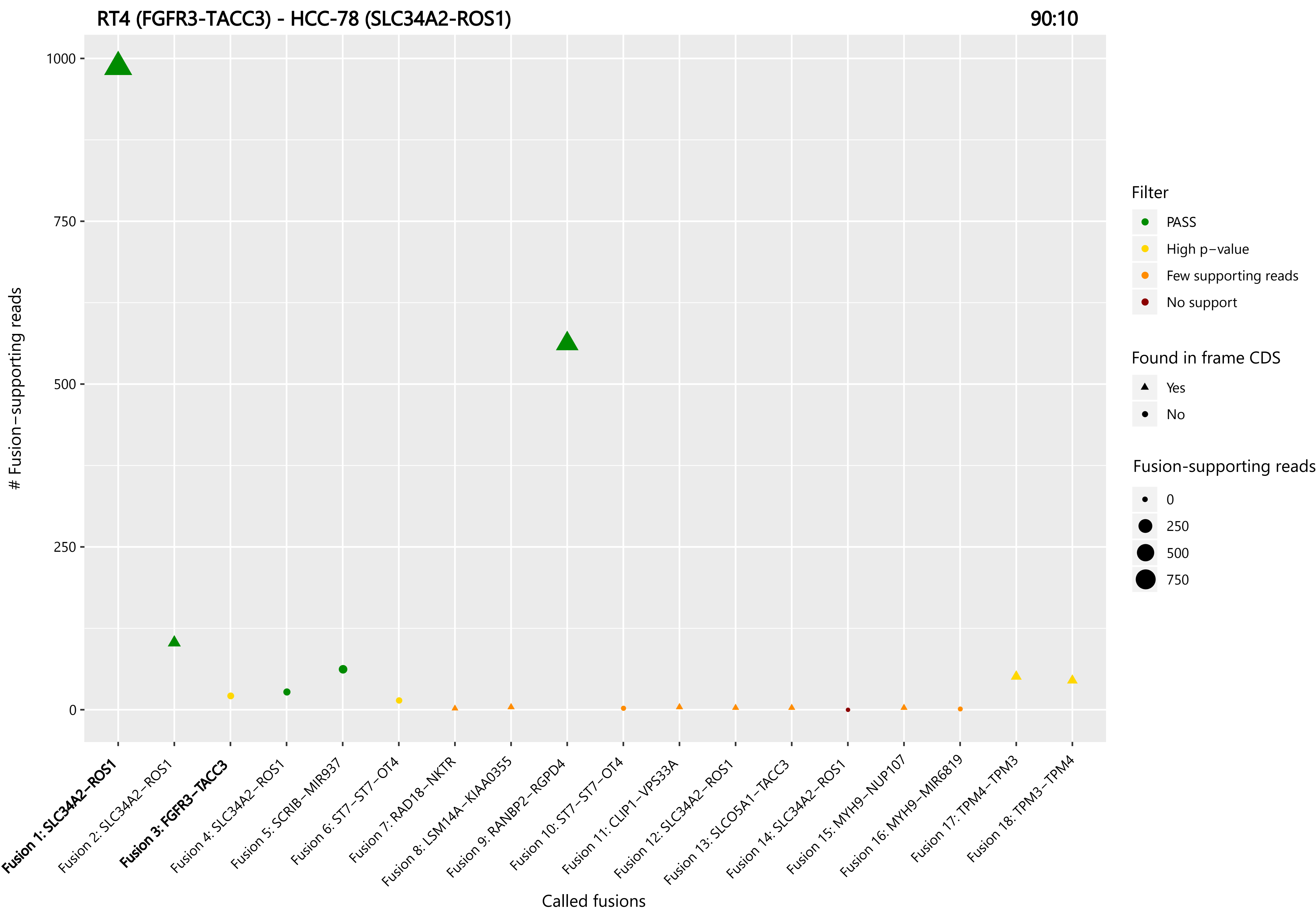

RT4 (FGFR3-TACC3) - HCC-78 (SLC34A2-ROS1)
90:10
Fusion−supporting reads
1000
750
500
250
0
Fusion 1: SLC34A2−ROS1
Fusion 2: SLC34A2−ROS1
Fusion 3: FGFR3−TACC3
Fusion 4: SLC34A2−ROS1
Fusion 5: SCRIB−MIR937
Fusion 6: ST7−ST7−OT4
Fusion 7: RAD18−NKTR
Fusion 8: LSM14A−KIAA0355
Fusion 9: RANBP2−RGPD4
Fusion 10: ST7−ST7−OT4
Fusion 11: CLIP1−VPS33A
Fusion 12: SLC34A2−ROS1
Fusion 13: SLCO5A1−TACC3
Fusion 14: SLC34A2−ROS1
Fusion 15: MYH9−NUP107
Fusion 16: MYH9−MIR6819
Fusion 17: TPM4−TPM3
Fusion 18: TPM3−TPM4
Called fusions
Filter
PASS
High p−value
Few supporting reads
No support
Found in frame CDS
Yes
No
Fusion-supporting reads
0
250
500
750

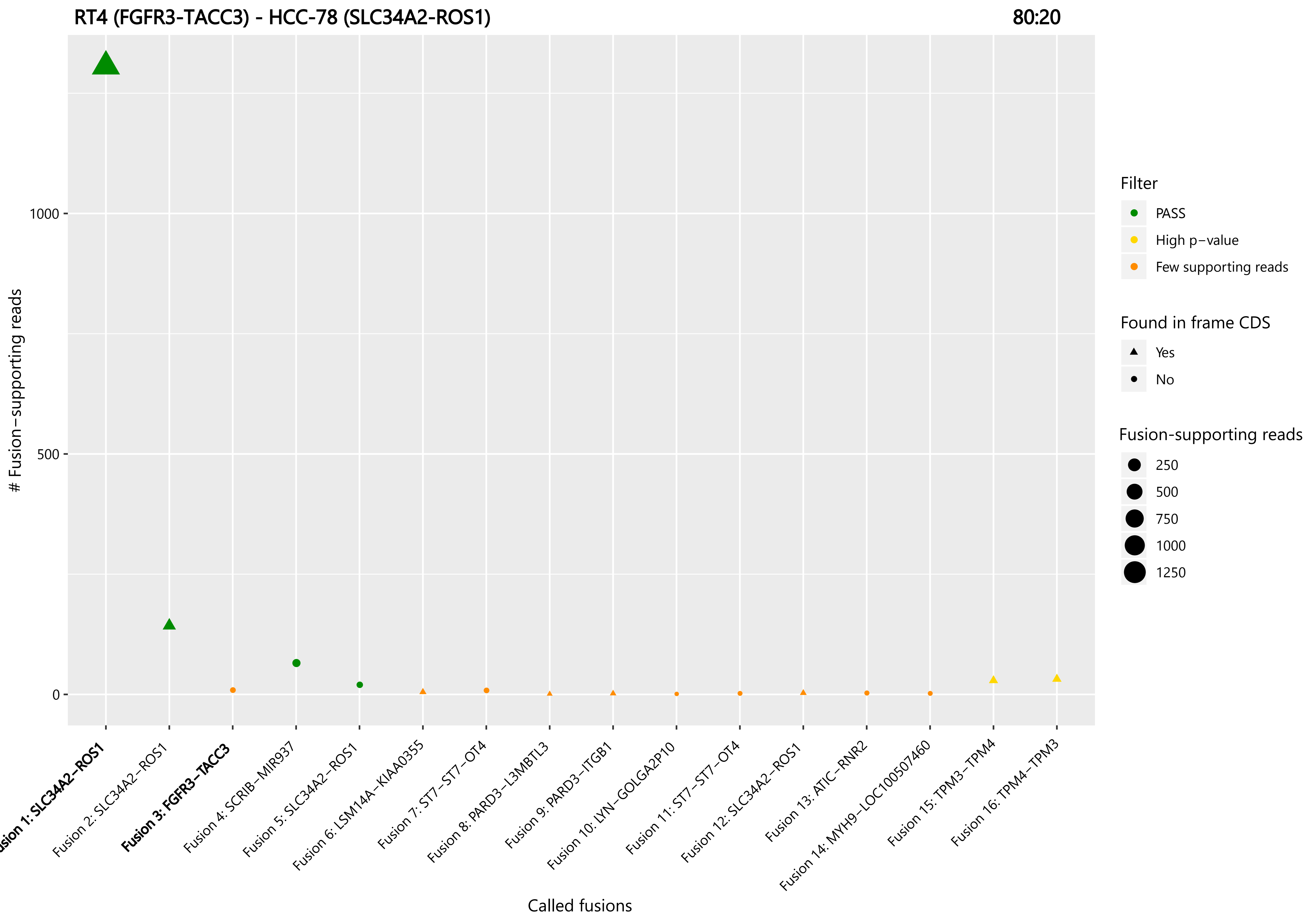
RT4 (FGFR3-TACC3) - HCC-78 (SLC34A2-ROS1)
80:20
Fusion−supporting reads
1000
500
0
Called fusions
Fusion 1: SLC34A2−ROS1
Fusion 2: SLC34A2−ROS1
Fusion 3: FGFR3−TACC3
Fusion 4: SCRIB−MIR937
Fusion 5: SLC34A2−ROS1
Fusion 6: LSM14A−KIAA0355
Fusion 7: ST7−ST7−OT4
Fusion 8: PARD3−L3MBTL3
Fusion 9: PARD3−ITGB1
Fusion 10: LYN−GOLGA2P10
Fusion 11: ST7−ST7−OT4
Fusion 12: SLC34A2−ROS1
Fusion 13: ATIC−RNR2
Fusion 14: MYH9−LOC100507460
Fusion 15: TPM3−TPM4
Fusion 16: TPM4−TPM3
Filter
PASS
High p−value
Few supporting reads
Found in frame CDS
Yes
No
Fusion-supporting reads
250
500
750
1000
1250

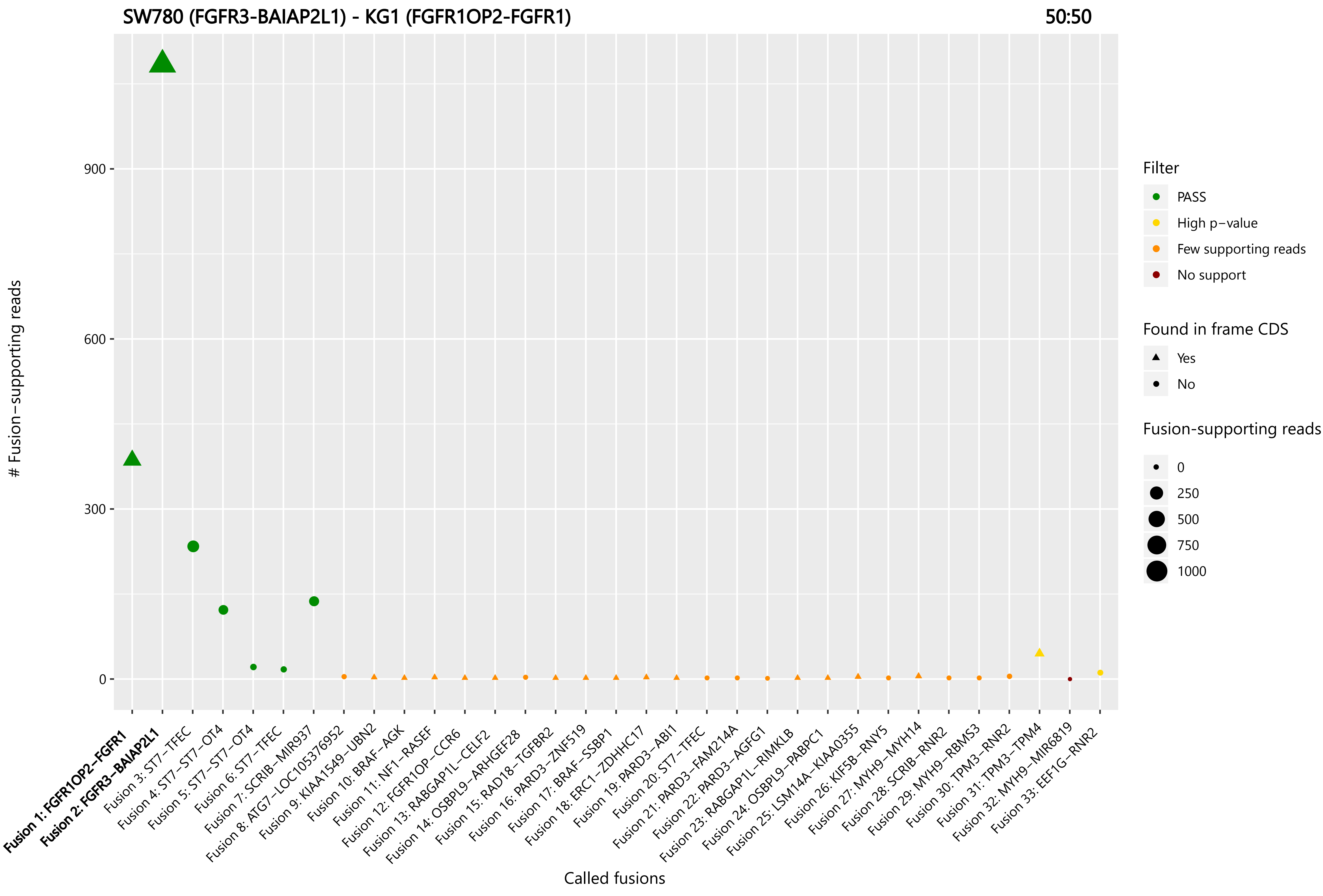

SW780 (FGFR3-BAIAP2L1) - KG1 (FGFR1OP2-FGFR1)
50:50
Fusion−supporting reads
900
600
300
0
Called fusions
Fusion 1: FGFR1OP2−FGFR1
Fusion 2: FGFR3−BAIAP2L1
Fusion 3: ST7−TFEC
Fusion 4: ST7−ST7−OT4
Fusion 5: ST7−ST7−OT4
Fusion 6: ST7−TFEC
Fusion 7: SCRIB−MIR937
Fusion 8: ATG7−LOC105376952
Fusion 9: KIAA1549−UBN2
Fusion 10: BRAF−AGK
Fusion 11: NF1−RASEF
Fusion 12: FGFR1OP−CCR6
Fusion 13: RABGAP1L−CELF2
Fusion 14: OSBPL9−ARHGEF28
Fusion 15: RAD18−TGFBR2
Fusion 16: PARD3−ZNF519
Fusion 17: BRAF−SSBP1
Fusion 18: ERC1−ZDHHC17
Fusion 19: PARD3−ABI1
Fusion 20: ST7−TFEC
Fusion 21: PARD3−FAM214A
Fusion 22: PARD3−AGFG1
Fusion 23: RABGAP1L−RIMKLB
Fusion 24: OSBPL9−PABPC1
Fusion 25: LSM14A−KIAA0355
Fusion 26: KIF5B−RNY5
Fusion 27: MYH9−MYH14
Fusion 28: SCRIB−RNR2
Fusion 29: MYH9−RBMS3
Fusion 30: TPM3−RNR2
Fusion 31: TPM3−TPM4
Fusion 32: MYH9−MIR6819
Fusion 33: EEF1G−RNR2
Filter
PASS
High p−value
Few supporting reads
No support
Found in frame CDS
Yes
No
Fusion-supporting reads
0
250
500
750
1000

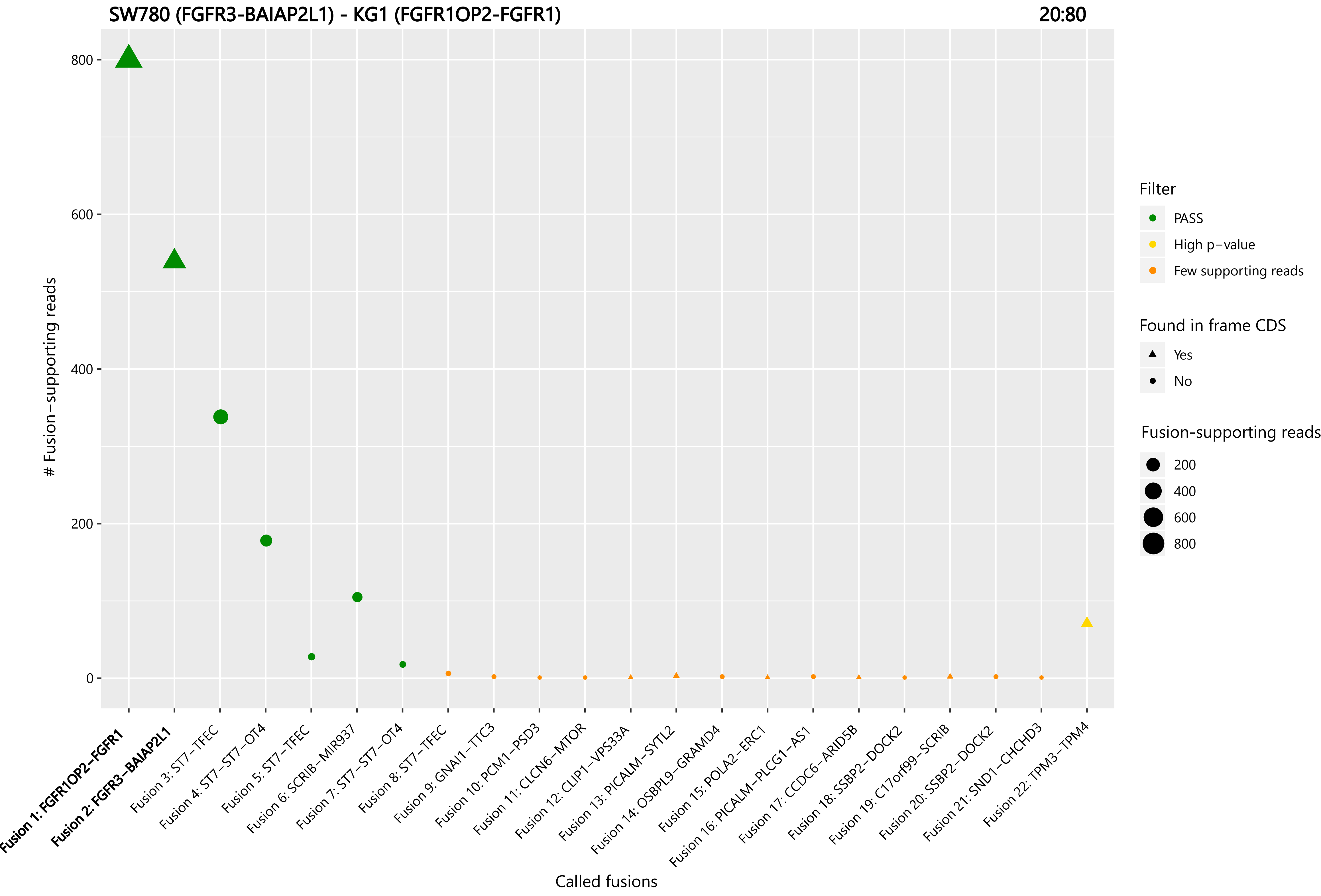

SW780 (FGFR3-BAIAP2L1) - KG1 (FGFR1OP2-FGFR1)
20:80
Fusion−supporting reads
800
600
400
200
0
Called fusions
Fusion 1: FGFR1OP2−FGFR1
Fusion 2: FGFR3−BAIAP2L1
Fusion 3: ST7−TFEC
Fusion 4: ST7−ST7−OT4
Fusion 5: ST7−TFEC
Fusion 6: SCRIB−MIR937
Fusion 7: ST7−ST7−OT4
Fusion 8: ST7−TFEC
Fusion 9: GNAI1−TTC3
Fusion 10: PCM1−PSD3
Fusion 11: CLCN6−MTOR
Fusion 12: CLIP1−VPS33A
Fusion 13: PICALM−SYTL2
Fusion 14: OSBPL9−GRAMD4
Fusion 15: POLA2−ERC1
Fusion 16: PICALM−PLCG1−AS1
Fusion 17: CCDC6−ARID5B
Fusion 18: SSBP2−DOCK2
Fusion 19: C17orf99−SCRIB
Fusion 20: SSBP2−DOCK2
Fusion 21: SND1−CHCHD3
Fusion 22: TPM3−TPM4
Filter
PASS
High p−value
Few supporting reads
Found in frame CDS
Yes
No
Fusion-supporting reads
200
400
600
800

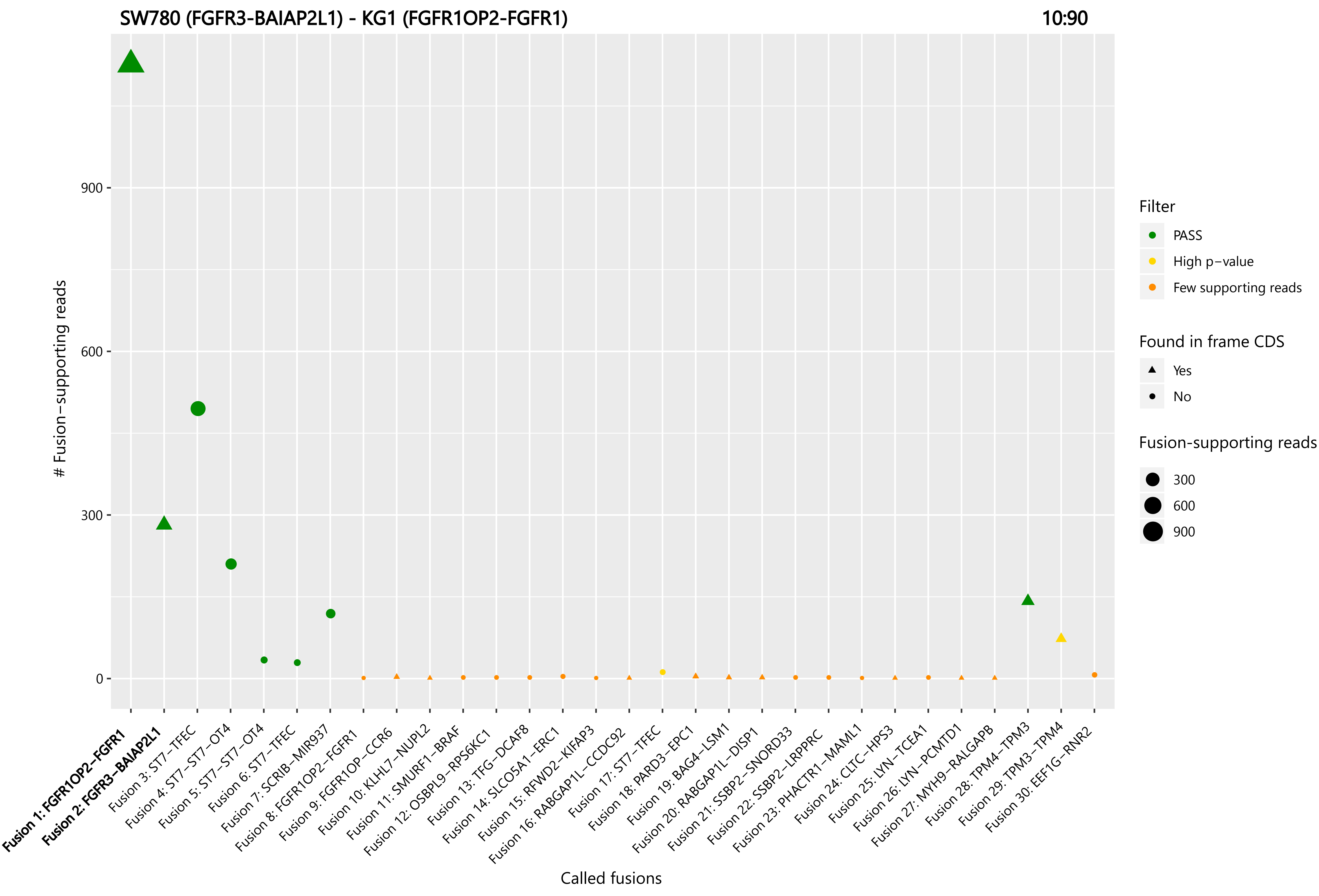

SW780 (FGFR3-BAIAP2L1) - KG1 (FGFR1OP2-FGFR1)
10:90
Fusion−supporting reads
900
600
300
0
Called fusions
Fusion 1: FGFR1OP2−FGFR1
Fusion 2: FGFR3−BAIAP2L1
Fusion 3: ST7−TFEC
Fusion 4: ST7−ST7−OT4
Fusion 5: ST7−ST7−OT4
Fusion 6: ST7−TFEC
Fusion 7: SCRIB−MIR937
Fusion 8: FGFR1OP2−FGFR1
Fusion 9: FGFR1OP−CCR6
Fusion 10: KLHL7−NUPL2
Fusion 11: SMURF1−BRAF
Fusion 12: OSBPL9−RPS6KC1
Fusion 13: TFG−DCAF8
Fusion 14: SLCO5A1−ERC1
Fusion 15: RFWD2−KIFAP3
Fusion 16: RABGAP1L−CCDC92
Fusion 17: ST7−TFEC
Fusion 18: PARD3−EPC1
Fusion 19: BAG4−LSM1
Fusion 20: RABGAP1L−DISP1
Fusion 21: SSBP2−SNORD33
Fusion 22: SSBP2−LRPPRC
Fusion 23: PHACTR1−MAML1
Fusion 24: CLTC−HPS3
Fusion 25: LYN−TCEA1
Fusion 26: LYN−PCMTD1
Fusion 27: MYH9−RALGAPB
Fusion 28: TPM4−TPM3
Fusion 29: TPM3−TPM4
Fusion 30: EEF1G−RNR2
Filter
PASS
High p−value
Few supporting reads
Found in frame CDS
Yes
No
Fusion-supporting reads
300
600
900

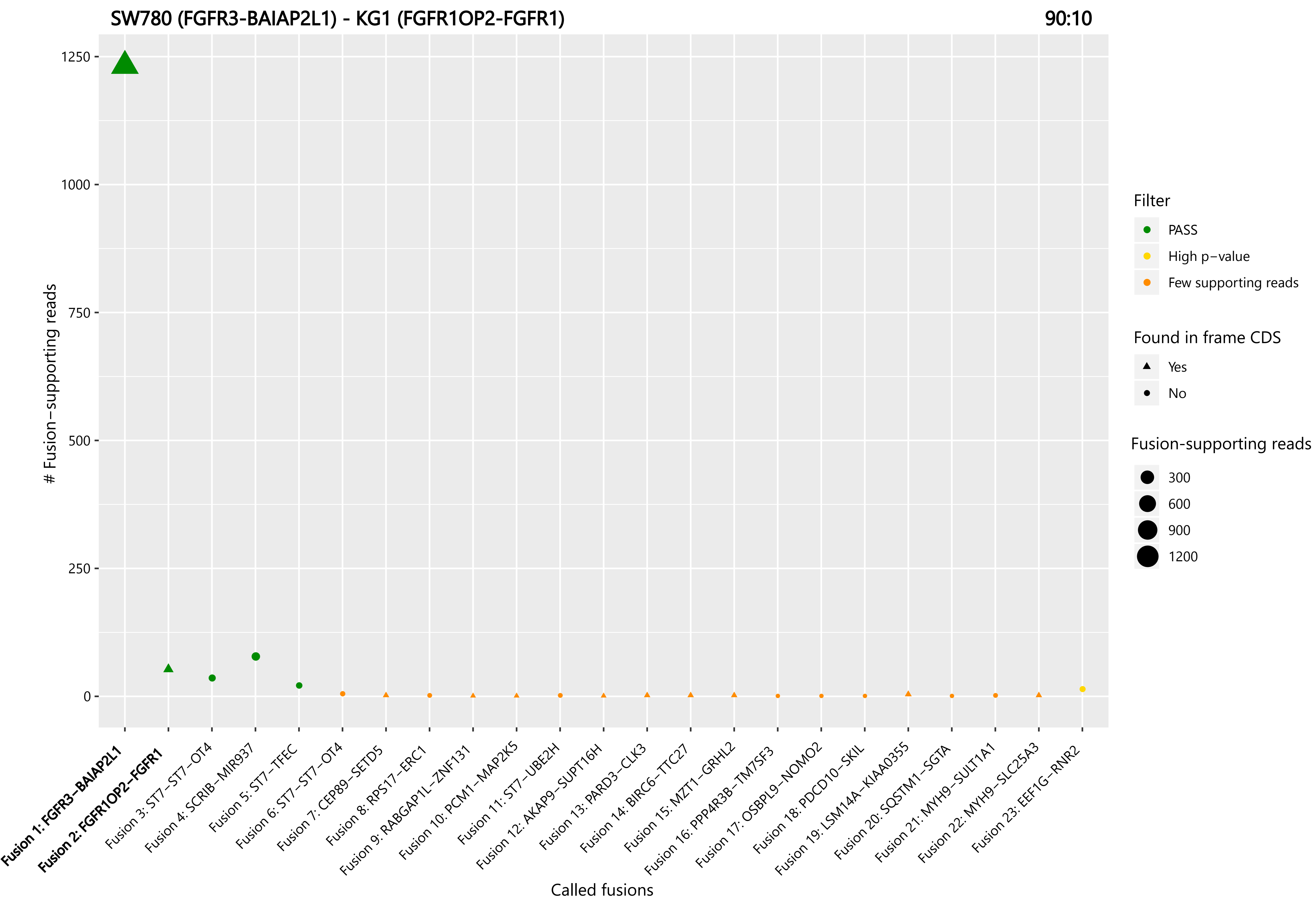

SW780 (FGFR3-BAIAP2L1) - KG1 (FGFR1OP2-FGFR1)
90:10
Fusion−supporting reads
1250
1000
750
500
250
0
Called fusions
Fusion 1: FGFR3−BAIAP2L1
Fusion 2: FGFR1OP2−FGFR1
Fusion 3: ST7−ST7−OT4
Fusion 4: SCRIB−MIR937
Fusion 5: ST7−TFEC
Fusion 6: ST7−ST7−OT4
Fusion 7: CEP89−SETD5
Fusion 8: RPS17−ERC1
Fusion 9: RABGAP1L−ZNF131
Fusion 10: PCM1−MAP2K5
Fusion 11: ST7−UBE2H
Fusion 12: AKAP9−SUPT16H
Fusion 13: PARD3−CLK3
Fusion 14: BIRC6−TTC27
Fusion 15: MZT1−GRHL2
Fusion 16: PPP4R3B−TM7SF3
Fusion 17: OSBPL9−NOMO2
Fusion 18: PDCD10−SKIL
Fusion 19: LSM14A−KIAA0355
Fusion 20: SQSTM1−SGTA
Fusion 21: MYH9−SULT1A1
Fusion 22: MYH9−SLC25A3
Fusion 23: EEF1G−RNR2
Filter
PASS
High p−value
Few supporting reads
Found in frame CDS
Yes
No
Fusion-supporting reads
300
600
900
1200

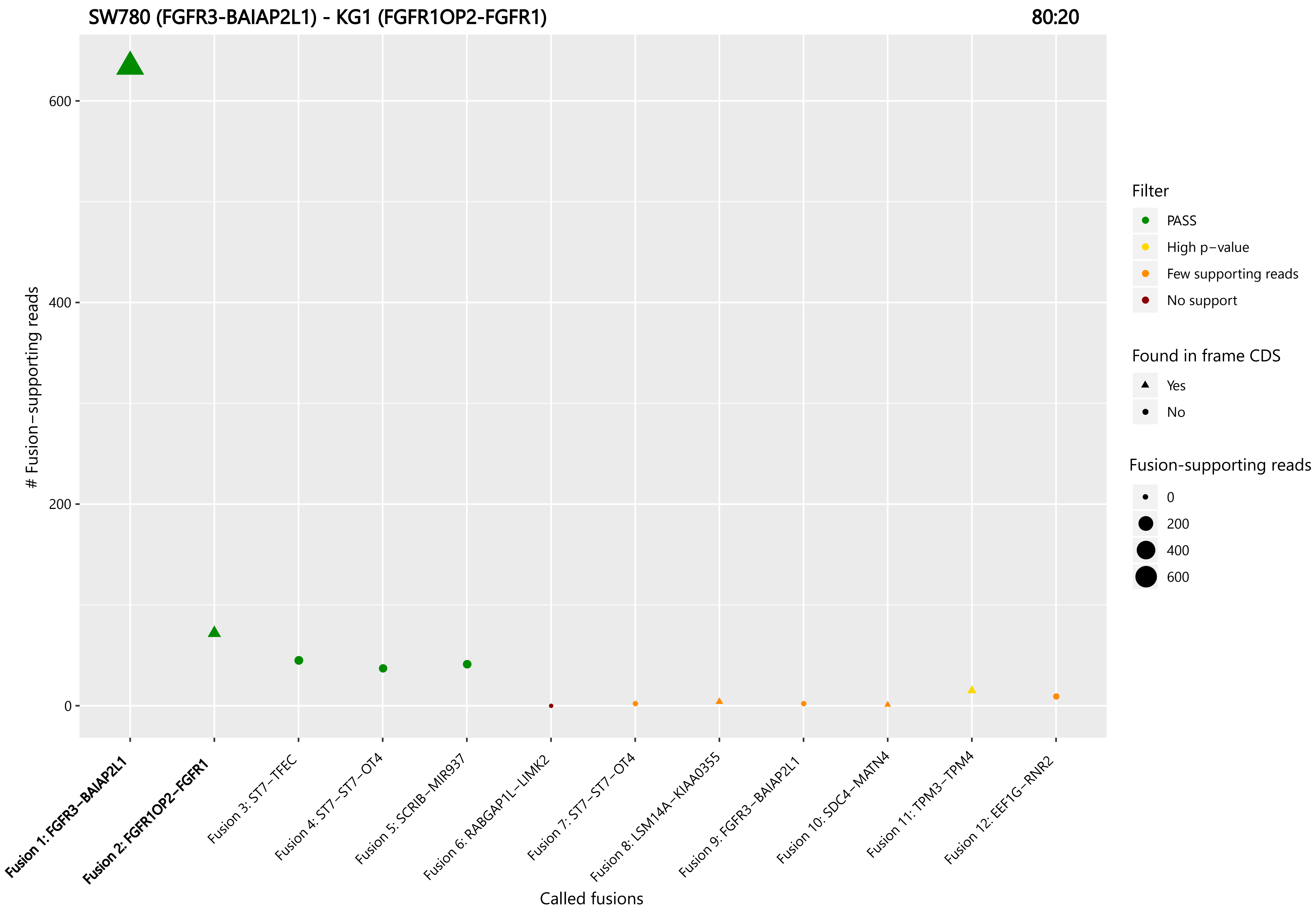
SW780 (FGFR3-BAIAP2L1) - KG1 (FGFR1OP2-FGFR1)
80:20
Fusion−supporting reads
600
400
200
0
Called fusions
Fusion 1: FGFR3−BAIAP2L1
Fusion 2: FGFR1OP2−FGFR1
Fusion 3: ST7−TFEC
Fusion 4: ST7−ST7−OT4
Fusion 5: SCRIB−MIR937
Fusion 6: RABGAP1L−LIMK2
Fusion 7: ST7−ST7−OT4
Fusion 8: LSM14A−KIAA0355
Fusion 9: FGFR3−BAIAP2L1
Fusion 10: SDC4−MATN4
Fusion 11: TPM3−TPM4
Fusion 12: EEF1G−RNR2
Filter
PASS
High p−value
Few supporting reads
No support
Found in frame CDS
Yes
No
Fusion-supporting reads
0
200
400
600

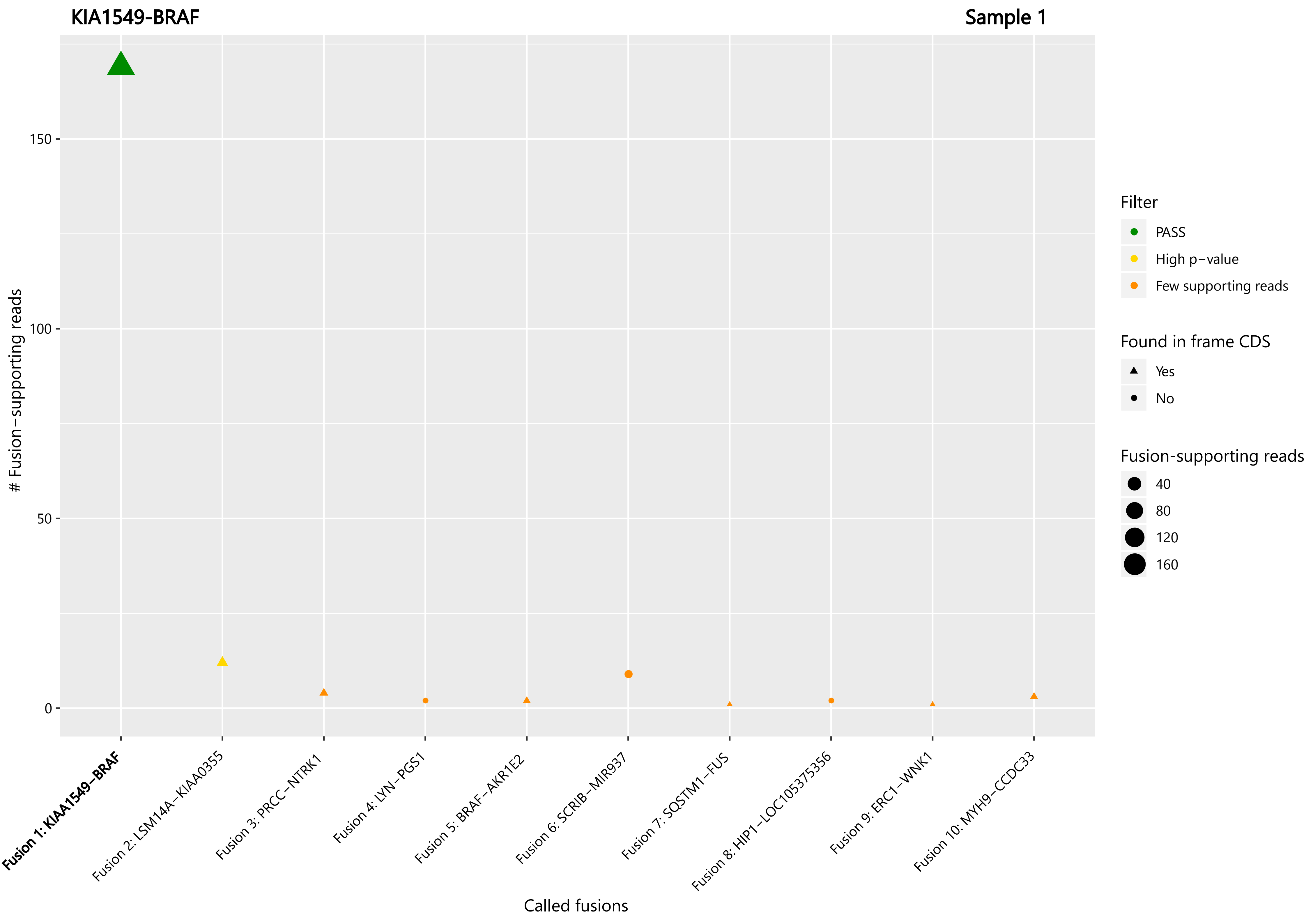
KIA1549-BRAF
Sample 1
Fusion−supporting reads
150
100
50
0
Called fusions
Fusion 1: KIAA1549−BRAF
Fusion 2: LSM14A−KIAA0355
Fusion 3: PRCC−NTRK1
Fusion 4: LYN−PGS1
Fusion 5: BRAF−AKR1E2
Fusion 6: SCRIB−MIR937
Fusion 7: SQSTM1−FUS
Fusion 8: HIP1−LOC105375356
Fusion 9: ERC1−WNK1
Fusion 10: MYH9−CCDC33
Filter
PASS
High p−value
Few supporting reads
Found in frame CDS
Yes
No
Fusion-supporting reads
40
80
120
160

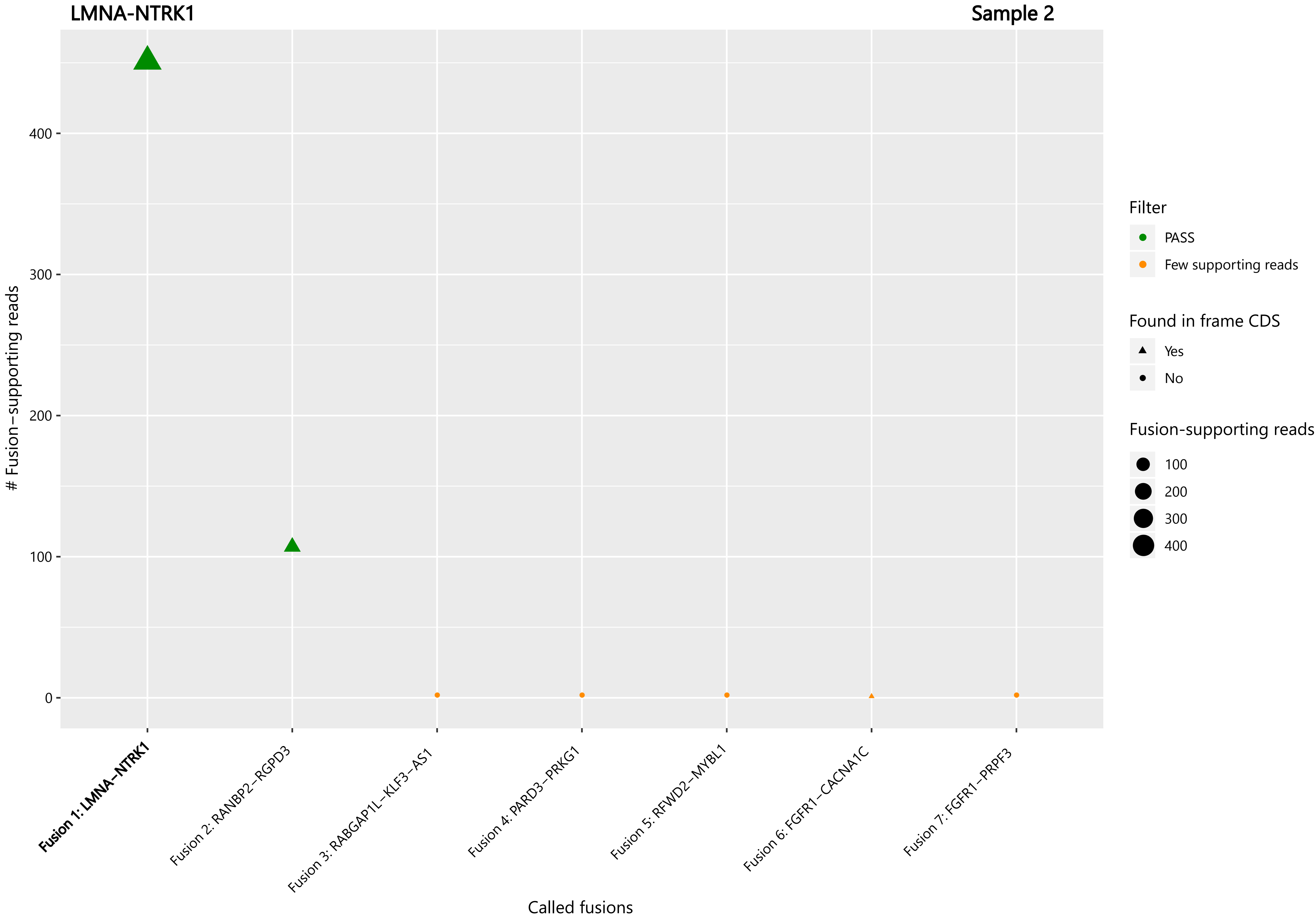

LMNA-NTRK1
Sample 2
Fusion−supporting reads
0
100
200
300
400
Fusion 1: LMNA−NTRK1
Fusion 2: RANBP2−RGPD3
Fusion 3: RABGAP1L−KLF3−AS1
Fusion 4: PARD3−PRKG1
Fusion 5: RFWD2−MYBL1
Fusion 6: FGFR1−CACNA1C
Fusion 7: FGFR1−PRPF3
Called fusions
Filter
PASS
Few supporting reads
Found in frame CDS
Yes
No
Fusion-supporting reads
100
200
300
400

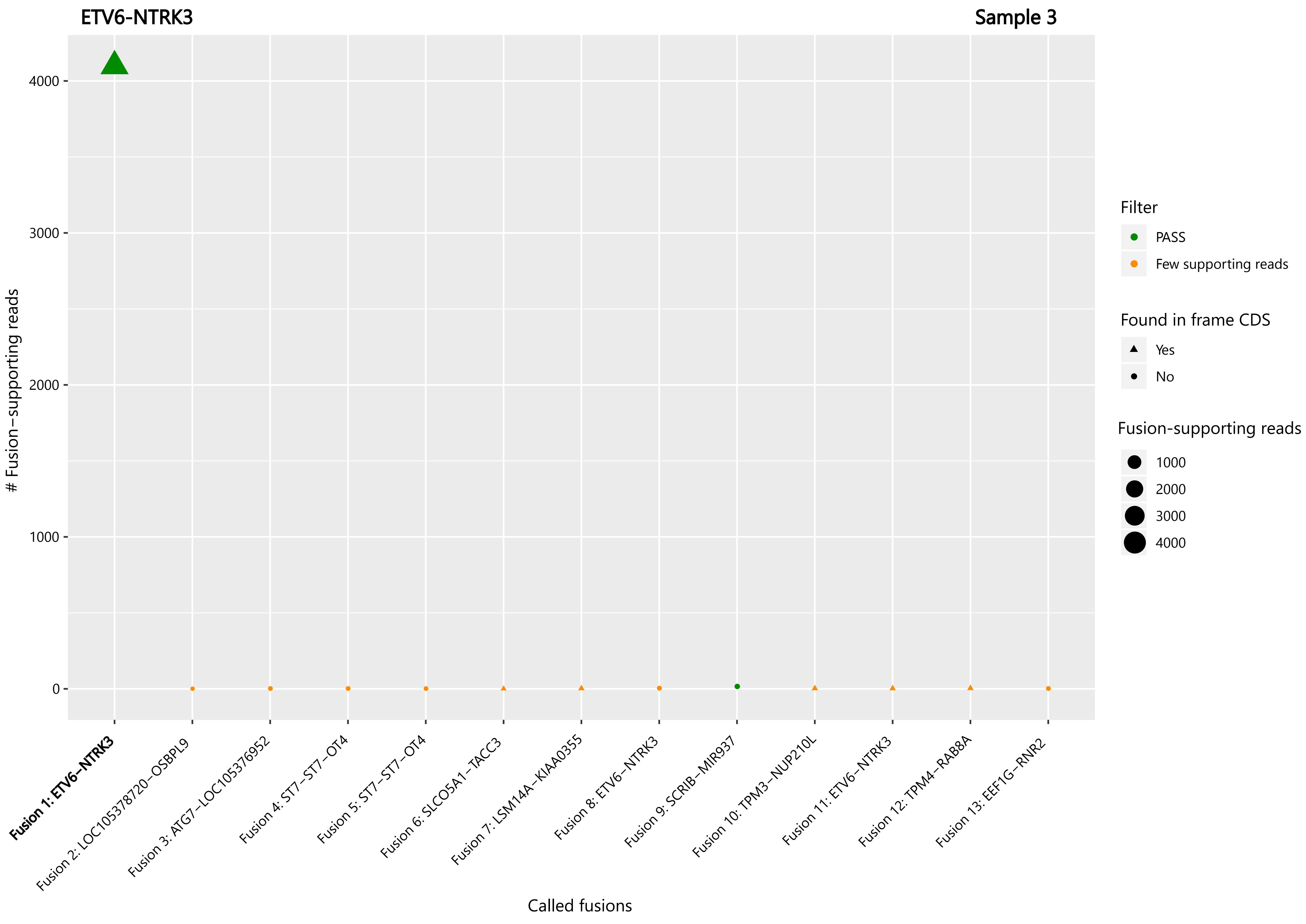

ETV6-NTRK3
Sample 3
Fusion−supporting reads
4000
3000
2000
1000
0
Fusion 1: ETV6−NTRK3
Fusion 2: LOC105378720−OSBPL9
Fusion 3: ATG7−LOC105376952
Fusion 4: ST7−ST7−OT4
Fusion 5: ST7−ST7−OT4
Fusion 6: SLCO5A1−TACC3
Fusion 7: LSM14A−KIAA0355
Fusion 8: ETV6−NTRK3
Fusion 9: SCRIB−MIR937
Fusion 10: TPM3−NUP210L
Fusion 11: ETV6−NTRK3
Fusion 12: TPM4−RAB8A
Fusion 13: EEF1G−RNR2
Called fusions
Filter
PASS
Few supporting reads
Found in frame CDS
Yes
No
Fusion-supporting reads
1000
2000
3000
4000

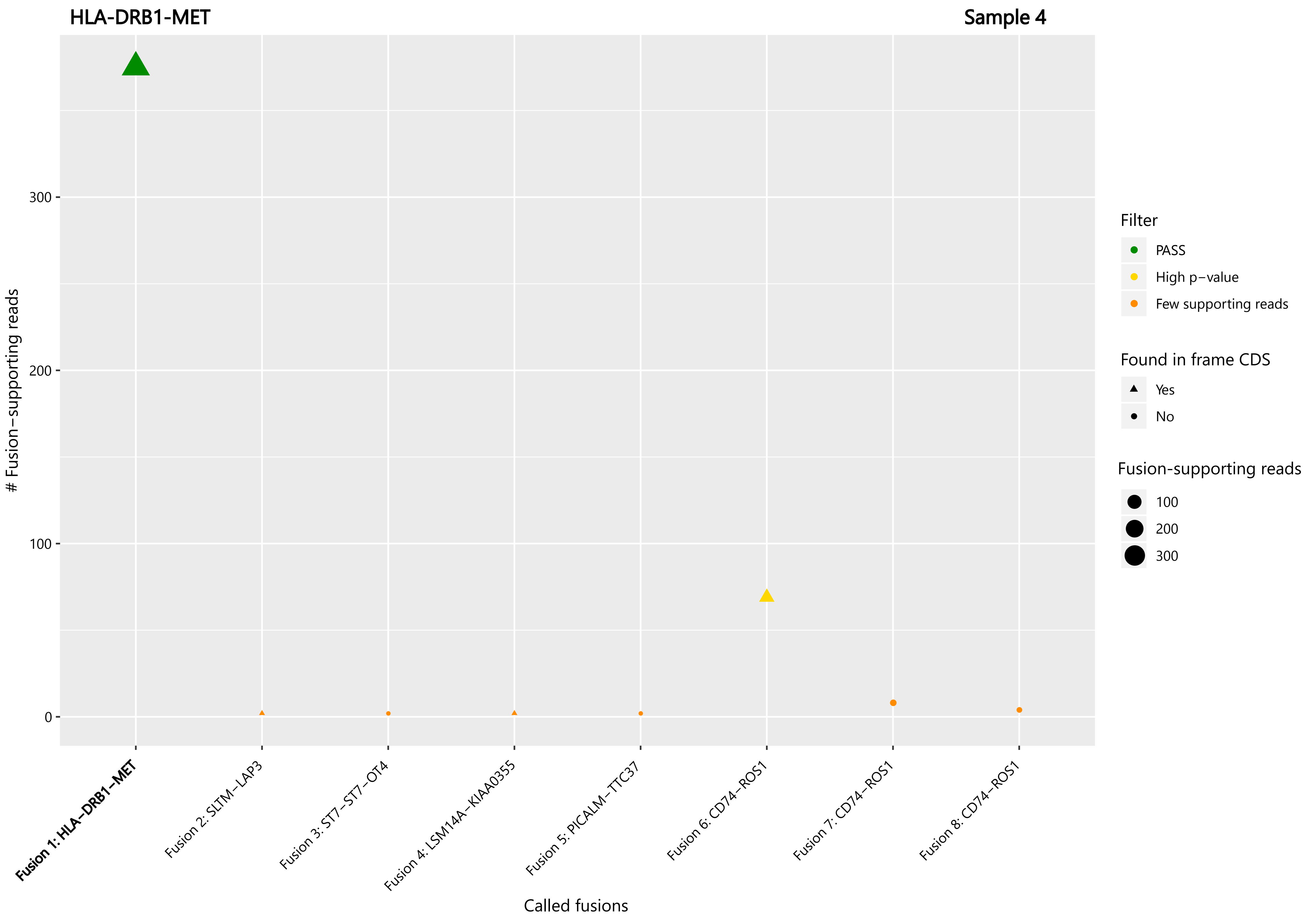
HLA-DRB1-MET
Sample 4
Fusion−supporting reads
0
100
200
300
Called fusions
Fusion 1: HLA−DRB1−MET
Fusion 2: SLTM−LAP3
Fusion 3: ST7−ST7−OT4
Fusion 4: LSM14A−KIAA0355
Fusion 5: PICALM−TTC37
Fusion 6: CD74−ROS1
Fusion 7: CD74−ROS1
Fusion 8: CD74−ROS1
Filter
PASS
High p−value
Few supporting reads
Found in frame CDS
Yes
No
Fusion-supporting reads
100
200
300

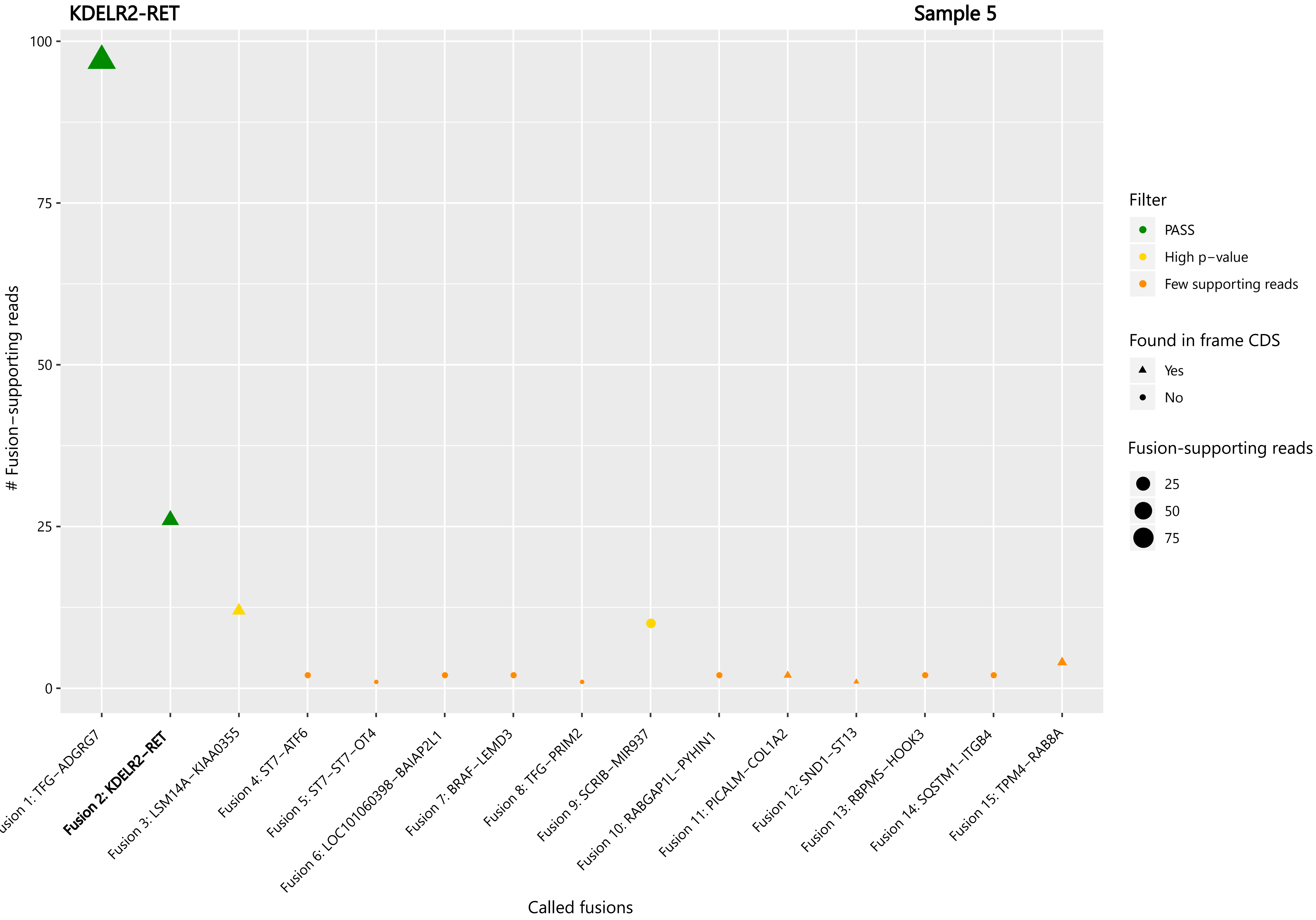
KDELR2-RET
Sample 5
100
75
50
25
0
Fusion−supporting reads
Called fusions
usion 1: TFG−ADGRG7
Fusion 2: KDELR2−RET
Fusion 3: LSM14A−KIAA0355
Fusion 4: ST7−ATF6
Fusion 5: ST7−ST7−OT4
Fusion 6: LOC101060398−BAIAP2L1
Fusion 7: BRAF−LEMD3
Fusion 8: TFG−PRIM2
Fusion 9: SCRIB−MIR937
Fusion 10: RABGAP1L−PYHIN1
Fusion 11: PICALM−COL1A2
Fusion 12: SND1−ST13
Fusion 13: RBPMS−HOOK3
Fusion 14: SQSTM1−ITGB4
Fusion 15: TPM4−RAB8A
Filter
PASS
High p−value
Few supporting reads
Found in frame CDS
Yes
No
Fusion-supporting reads
25
50
75

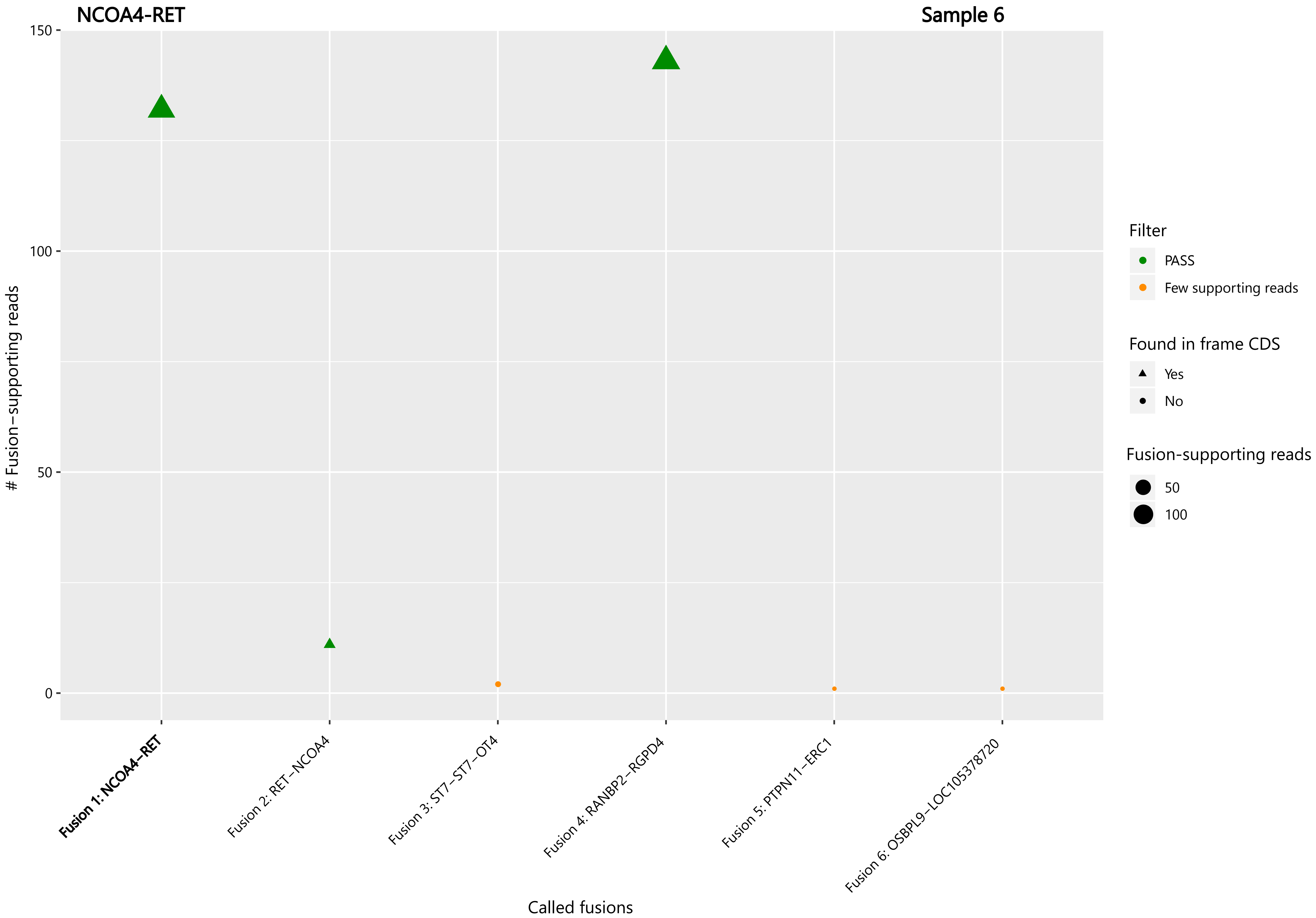
NCOA4-RET
Sample 6
150
100
50
0
Fusion−supporting reads
Fusion 1: NCOA4−RET
Fusion 2: RET−NCOA4
Fusion 3: ST7−ST7−OT4
Fusion 4: RANBP2−RGPD4
Fusion 5: PTPN11−ERC1
Fusion 6: OSBPL9−LOC105378720
Called fusions
Filter
PASS
Few supporting reads
Found in frame CDS
Yes
No
Fusion-supporting reads
50
100

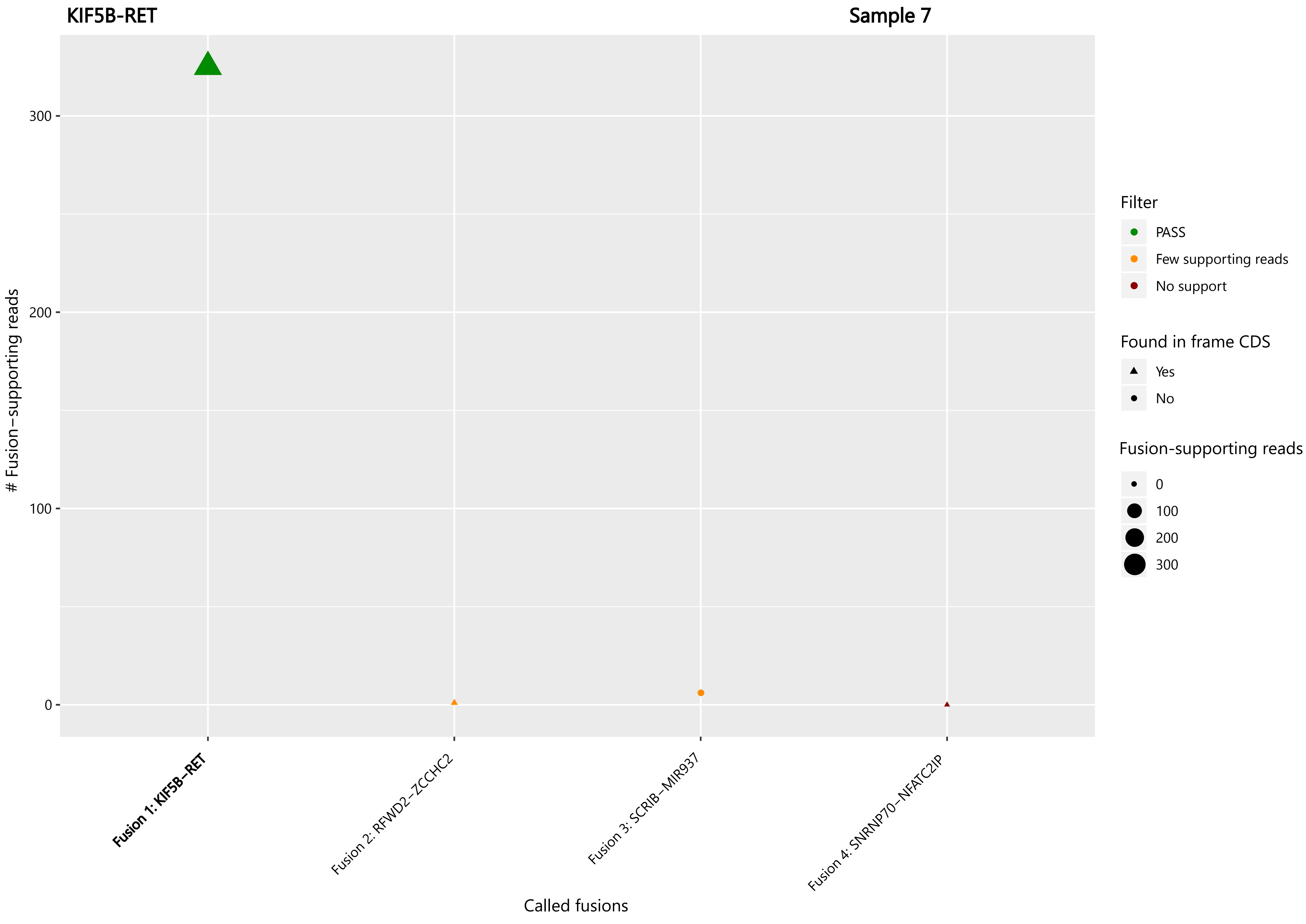
KIF5B-RET
Sample 7
Fusion−supporting reads
300
200
100
0
Fusion 1: KIF5B−RET
Fusion 2: RFWD2−ZCCHC2
Fusion 3: SCRIB−MIR937
Fusion 4: SNRNP70−NFATC2IP
Called fusions
Filter
PASS
Few supporting reads
No support
Found in frame CDS
Yes
No
Fusion-supporting reads
0
100
200
300

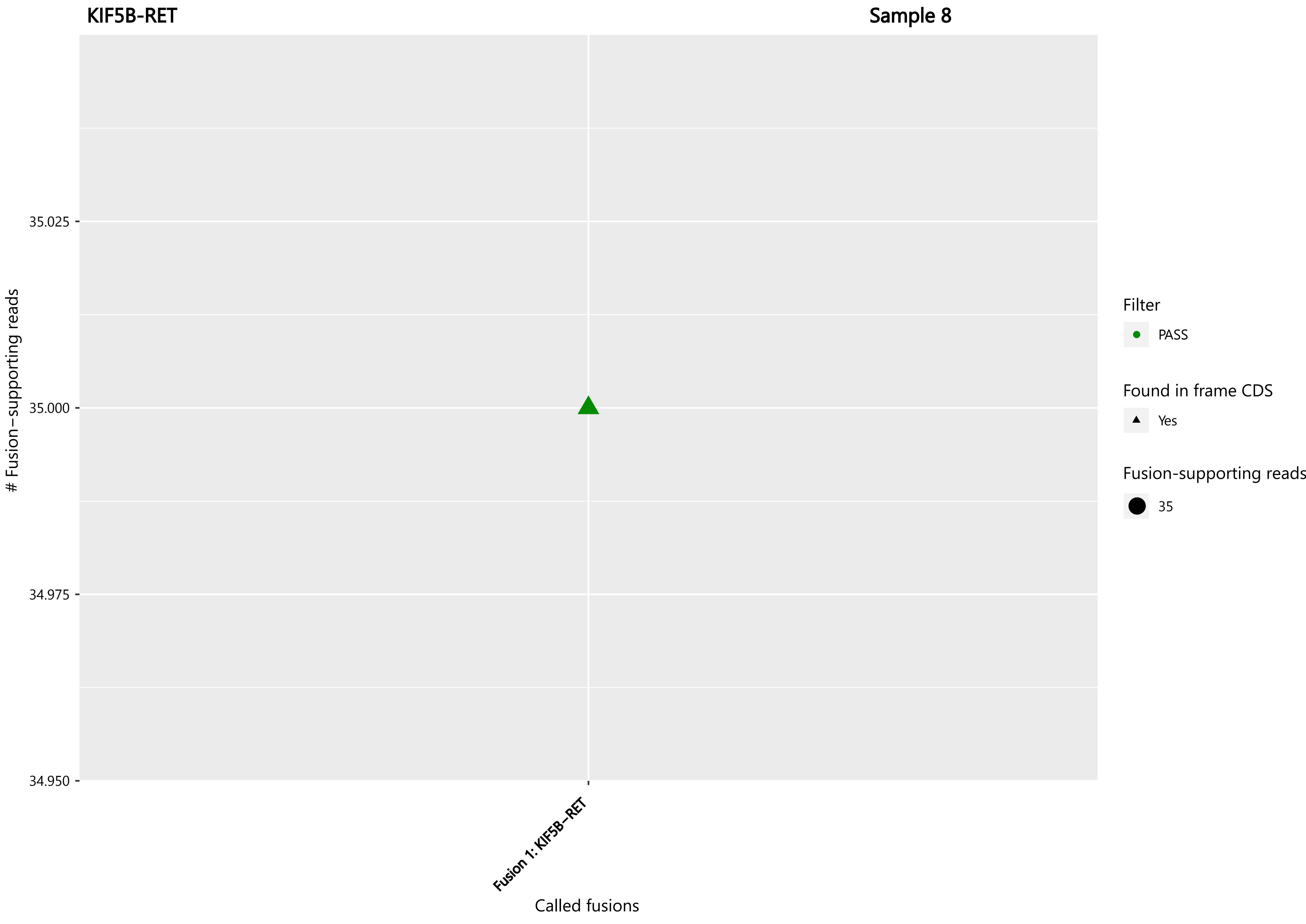
KIF5B-RET
Sample 8
Fusion−supporting reads
35.025
35.000
34.975
34.950
Fusion 1: KIF5B−RET
Called fusions
Filter
PASS
Found in frame CDS
Yes
Fusion-supporting reads
35

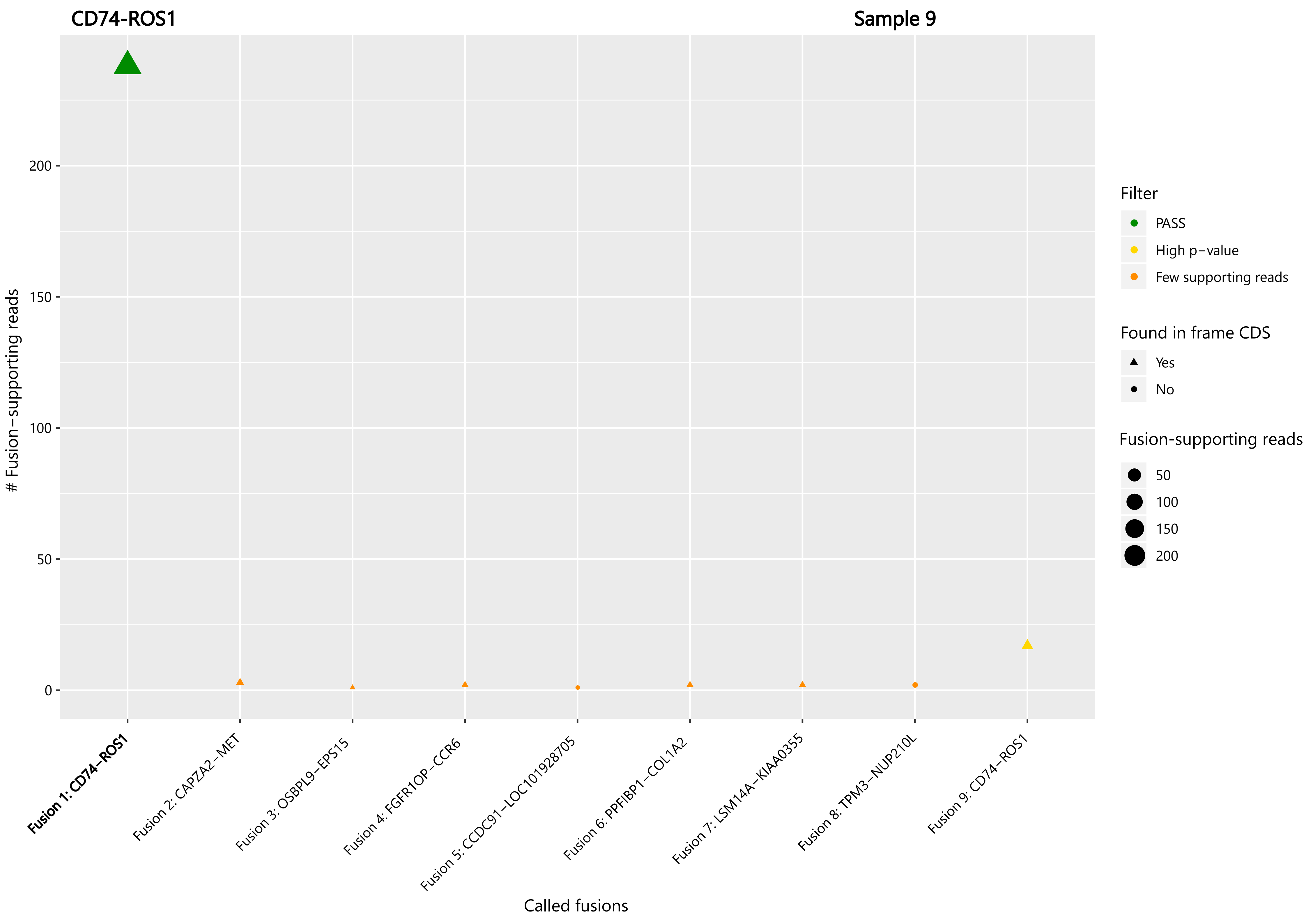
CD74-ROS1
Sample 9
Fusion−supporting reads
0
50
100
150
200
Called fusions
Fusion 1: CD74−ROS1
Fusion 2: CAPZA2−MET
Fusion 3: OSBPL9−EPS15
Fusion 4: FGFR1OP−CCR6
Fusion 5: CCDC91−LOC101928705
Fusion 6: PPFIBP1−COL1A2
Fusion 7: LSM14A−KIAA0355
Fusion 8: TPM3−NUP210L
Fusion 9: CD74−ROS1
Filter
PASS
High p−value
Few supporting reads
Found in frame CDS
Yes
No
Fusion-supporting reads
50
100
150
200

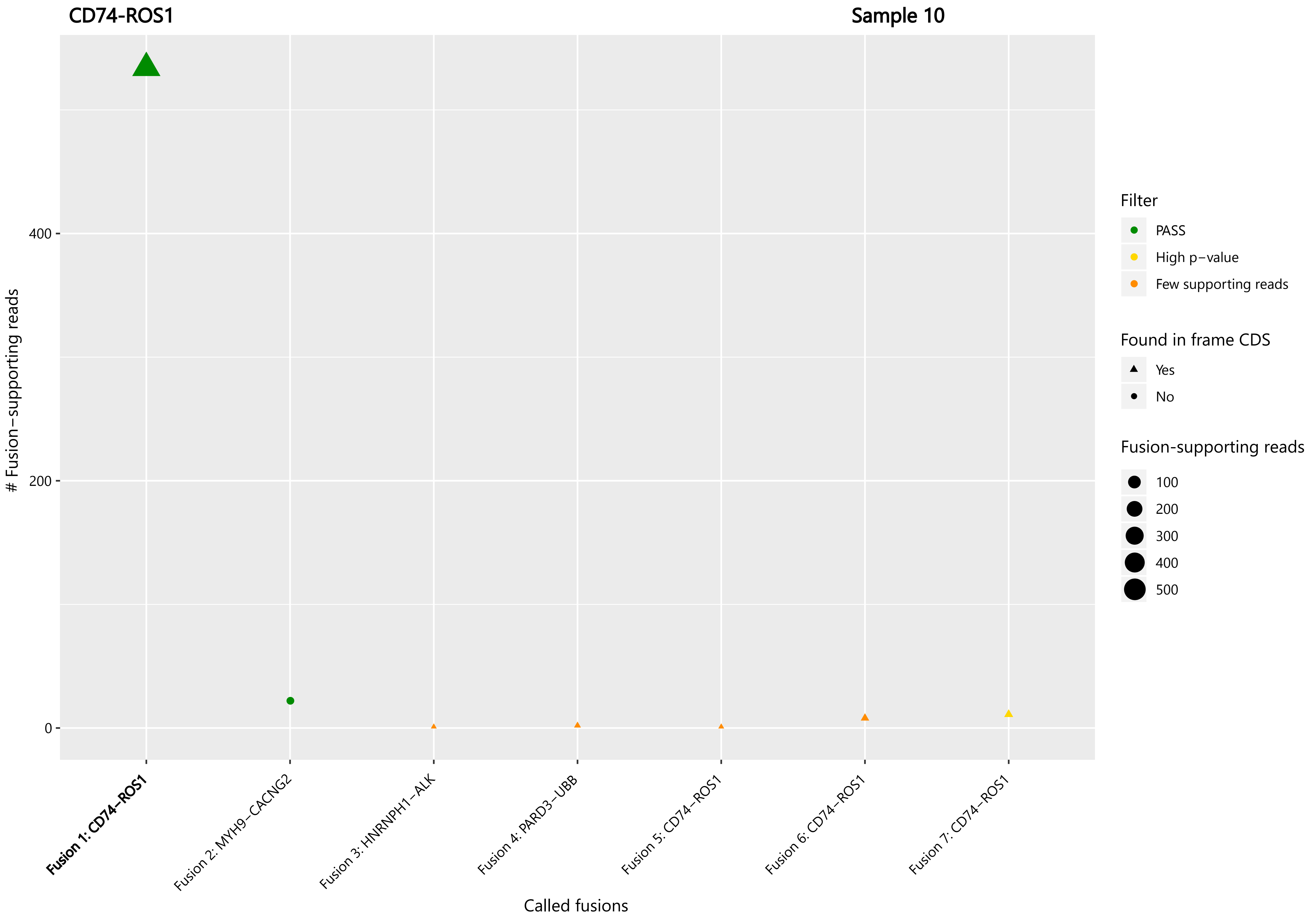
CD74-ROS1
Sample 10
Fusion−supporting reads
0
200
400
Fusion 1: CD74−ROS1
Fusion 2: MYH9−CACNG2
Fusion 3: HNRNPH1−ALK
Fusion 4: PARD3−UBB
Fusion 5: CD74−ROS1
Fusion 6: CD74−ROS1
Fusion 7: CD74−ROS1
Called fusions
Filter
PASS
High p−value
Few supporting reads
Found in frame CDS
Yes
No
Fusion-supporting reads
100
200
300
400
500

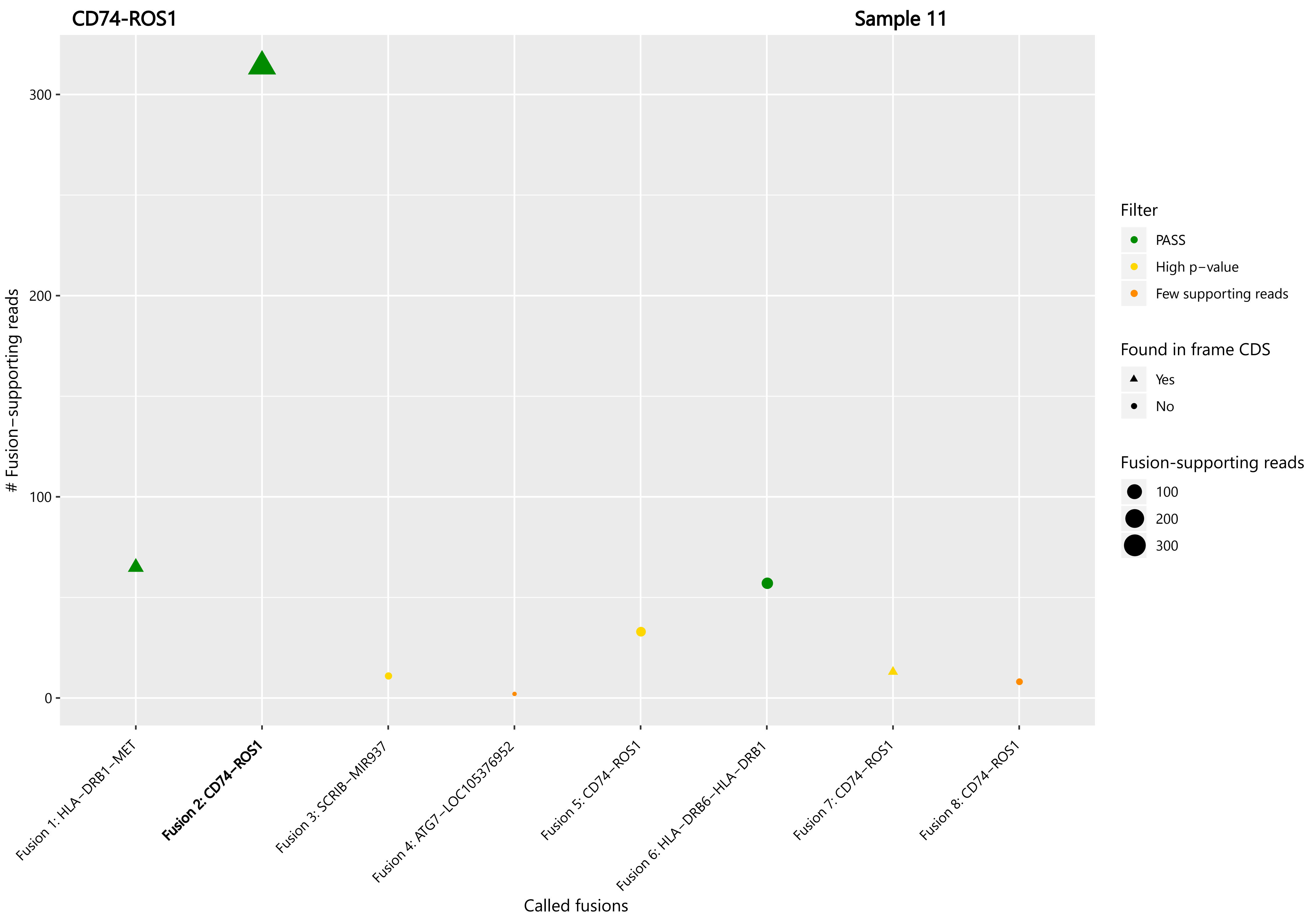
CD74-ROS1
Sample 11
Fusion−supporting reads
300
200
100
0
Fusion 1: HLA−DRB1−MET
Fusion 2: CD74−ROS1
Fusion 3: SCRIB−MIR937
Fusion 4: ATG7−LOC105376952
Fusion 5: CD74−ROS1
Fusion 6: HLA−DRB6−HLA−DRB1
Fusion 7: CD74−ROS1
Fusion 8: CD74−ROS1
Called fusions
Filter
PASS
High p−value
Few supporting reads
Found in frame CDS
Yes
No
Fusion-supporting reads
100
200
300

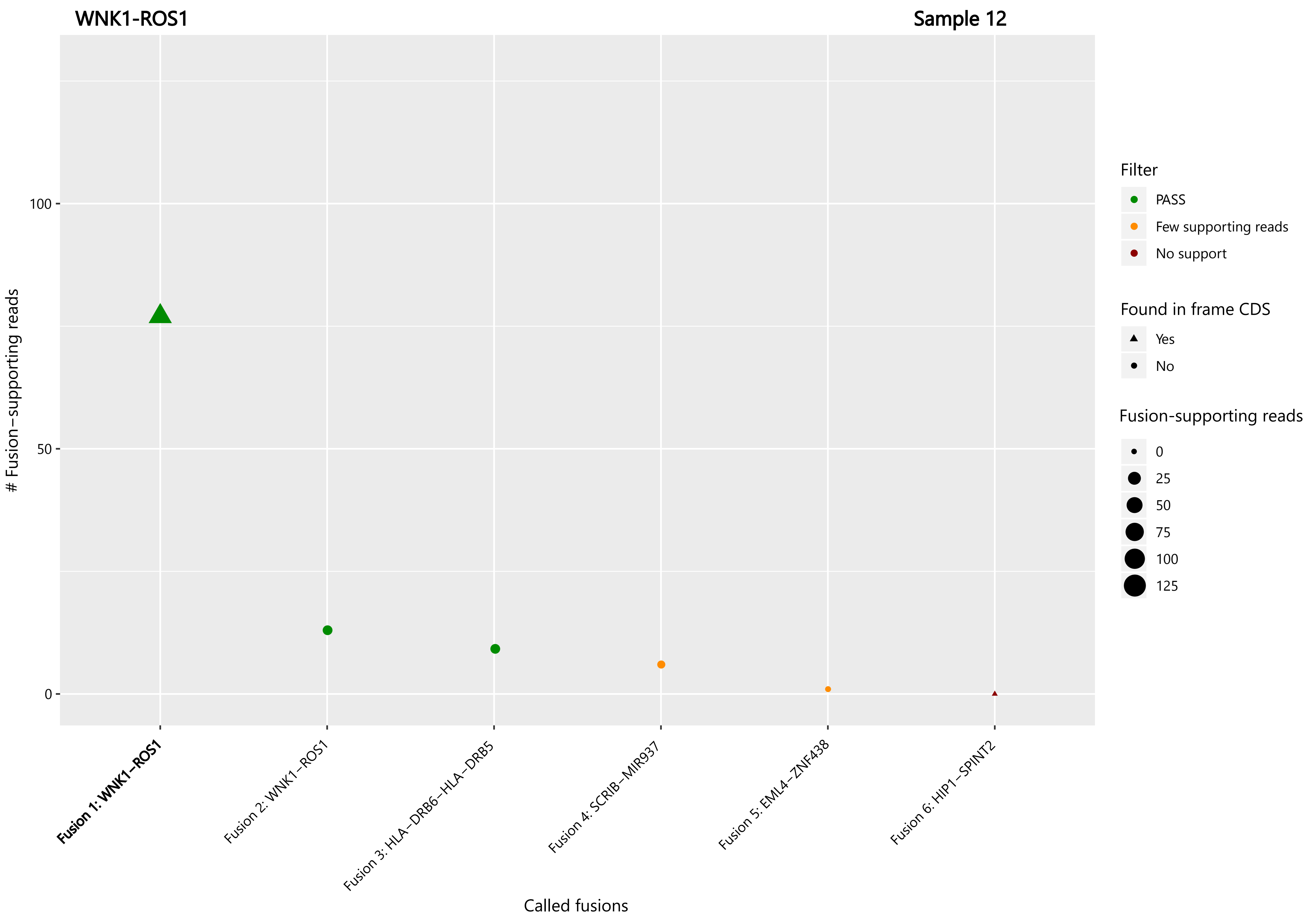
WNK1-ROS1
Sample 12
Fusion−supporting reads
100
50
0
Fusion 1: WNK1−ROS1
Fusion 2: WNK1−ROS1
Fusion 3: HLA−DRB6−HLA−DRB5
Fusion 4: SCRIB−MIR937
Fusion 5: EML4−ZNF438
Fusion 6: HIP1−SPINT2
Called fusions
Filter
PASS
Few supporting reads
No support
Found in frame CDS
Yes
No
Fusion-supporting reads
0
25
50
75
100
125

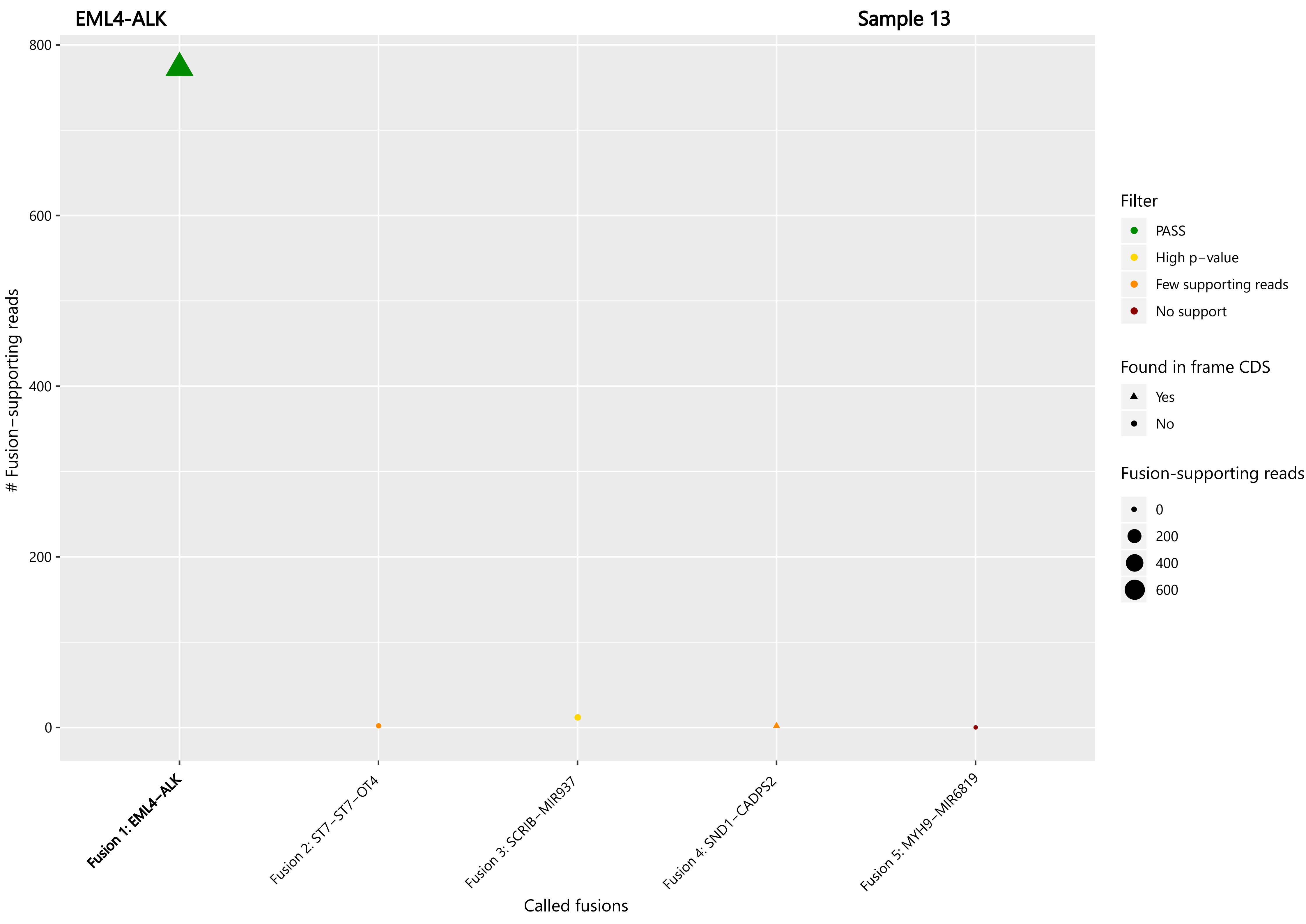
EML4-ALK
Sample 13
800
600
400
200
0
Fusion−supporting reads
Fusion 1: EML4−ALK
Fusion 2: ST7−ST7−OT4
Fusion 3: SCRIB−MIR937
Fusion 4: SND1−CADPS2
Fusion 5: MYH9−MIR6819
Called fusions
Filter
PASS
High p−value
Few supporting reads
No support
Found in frame CDS
Yes
No
Fusion-supporting reads
0
200
400
600

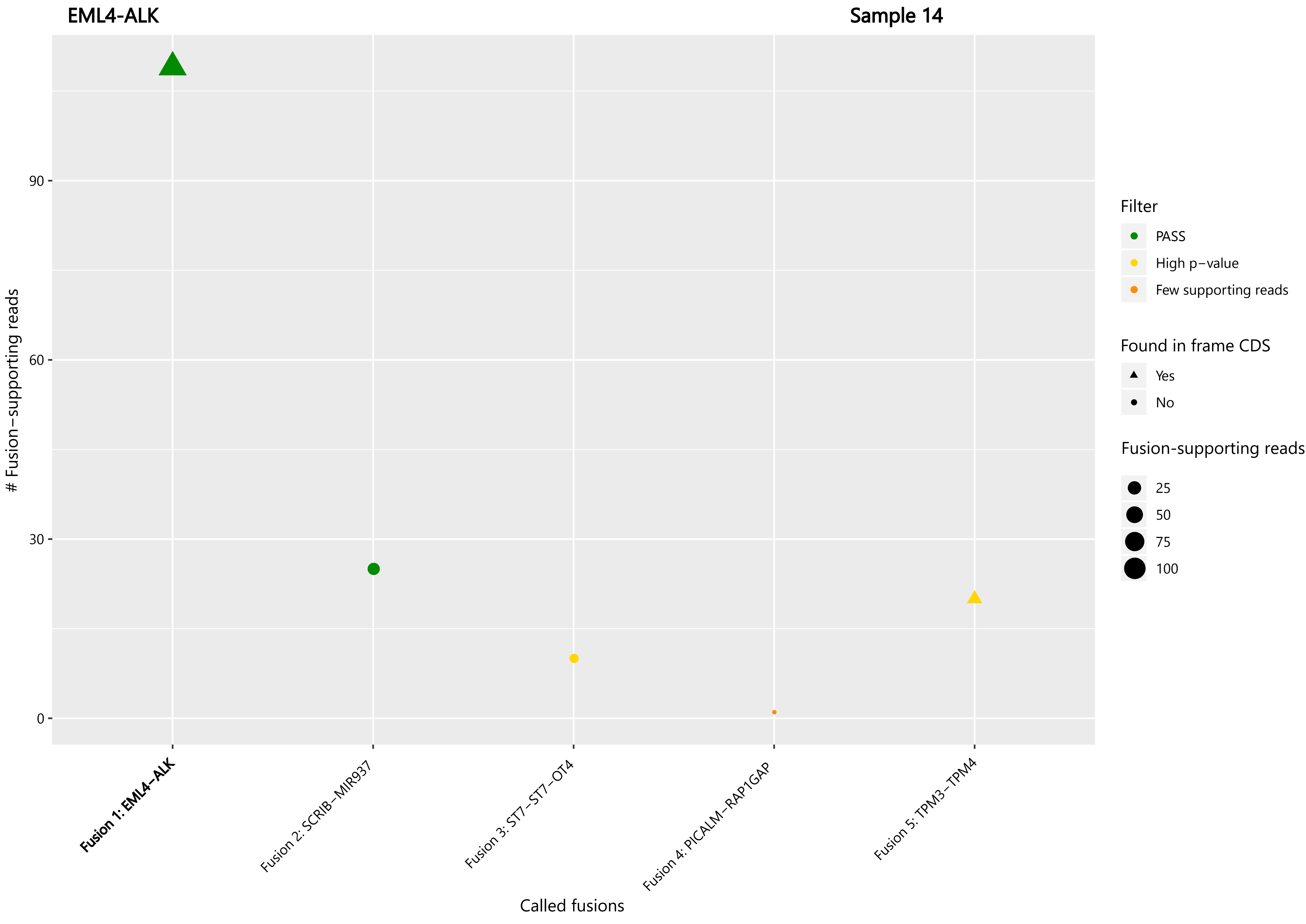

EML4-ALK
Sample 14
Fusion−supporting reads
0
30
60
90
Fusion 1: EML4−ALK
Fusion 2: SCRIB−MIR937
Fusion 3: ST7−ST7−OT4
Fusion 4: PICALM−RAP1GAP
Fusion 5: TPM3−TPM4
Called fusions
Filter
PASS
High p−value
Few supporting reads
Found in frame CDS
Yes
No
Fusion-supporting reads
25
50
75
100

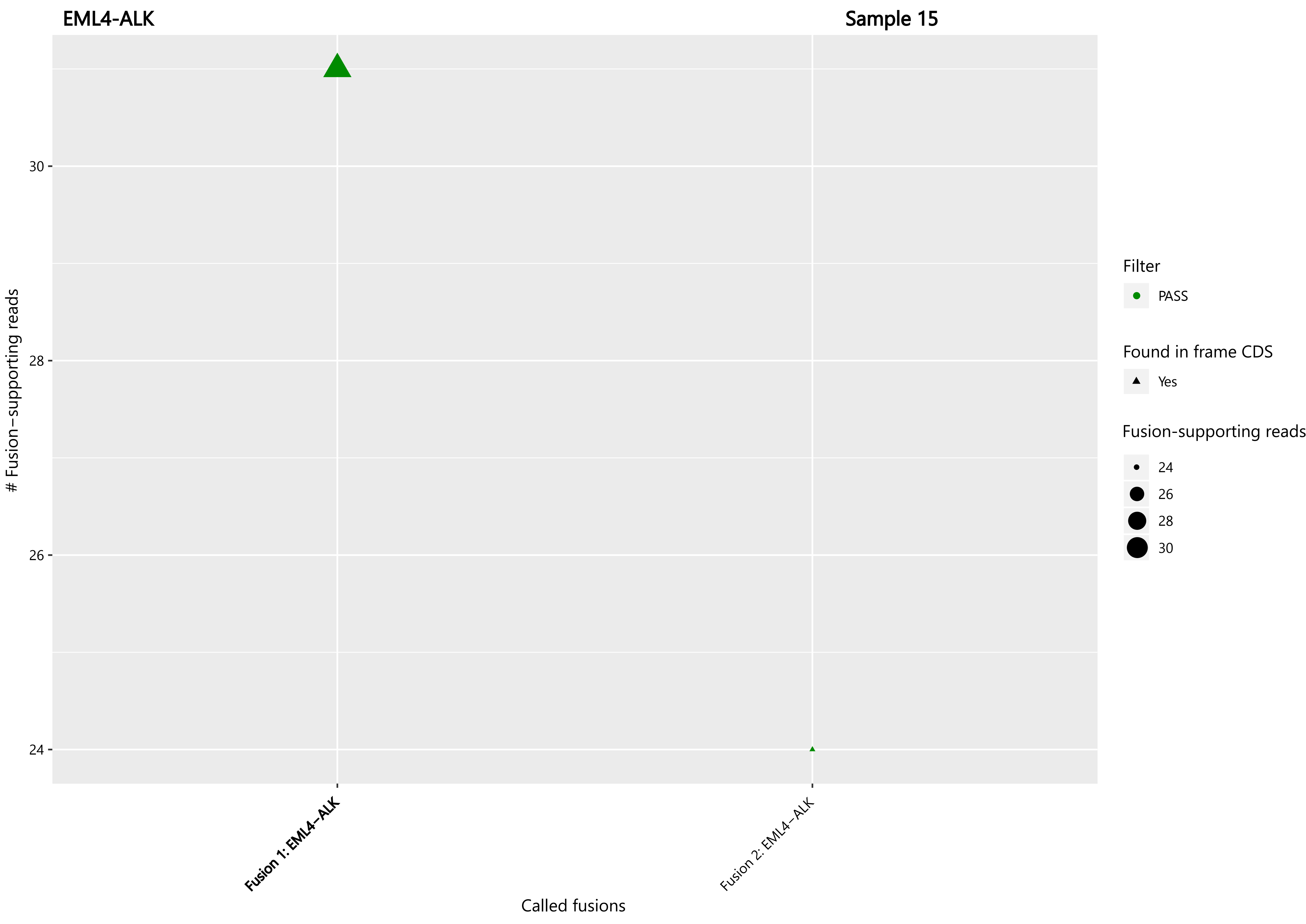

EML4-ALK
Sample 15
Fusion−supporting reads
30
28
26
24
Fusion 1: EML4−ALK
Fusion 2: EML4−ALK
Called fusions
Filter
PASS
Found in frame CDS
Yes
Fusion-supporting reads
24
26
28
30

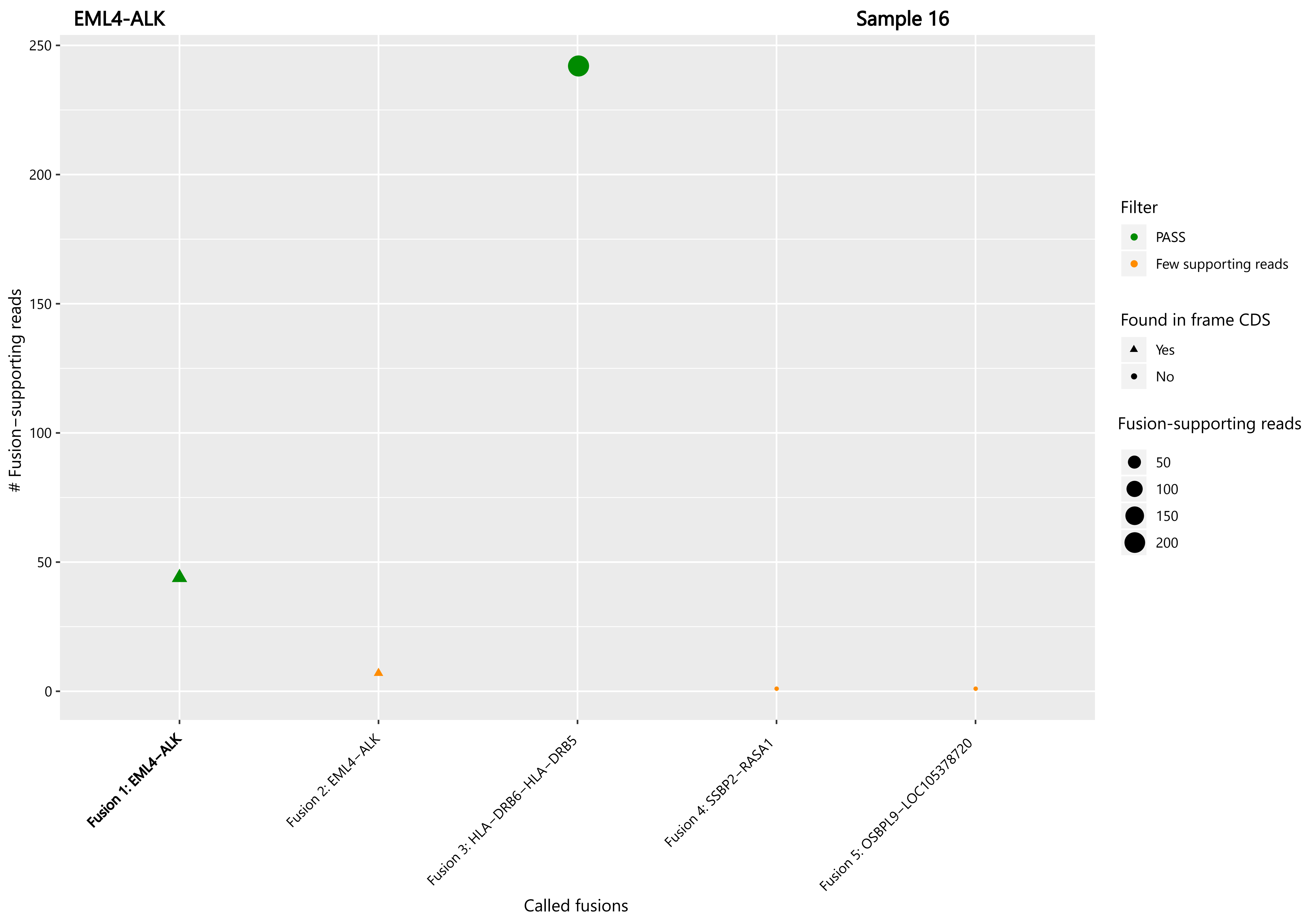
EML4-ALK
Sample 16
Fusion−supporting reads
250
200
150
100
50
0
Fusion 1: EML4−ALK
Fusion 2: EML4−ALK
Fusion 3: HLA−DRB6−HLA−DRB5
Fusion 4: SSBP2−RASA1
Fusion 5: OSBPL9−LOC105378720
Called fusions
Filter
PASS
Few supporting reads
Found in frame CDS
Yes
No
Fusion-supporting reads
50
100
150
200

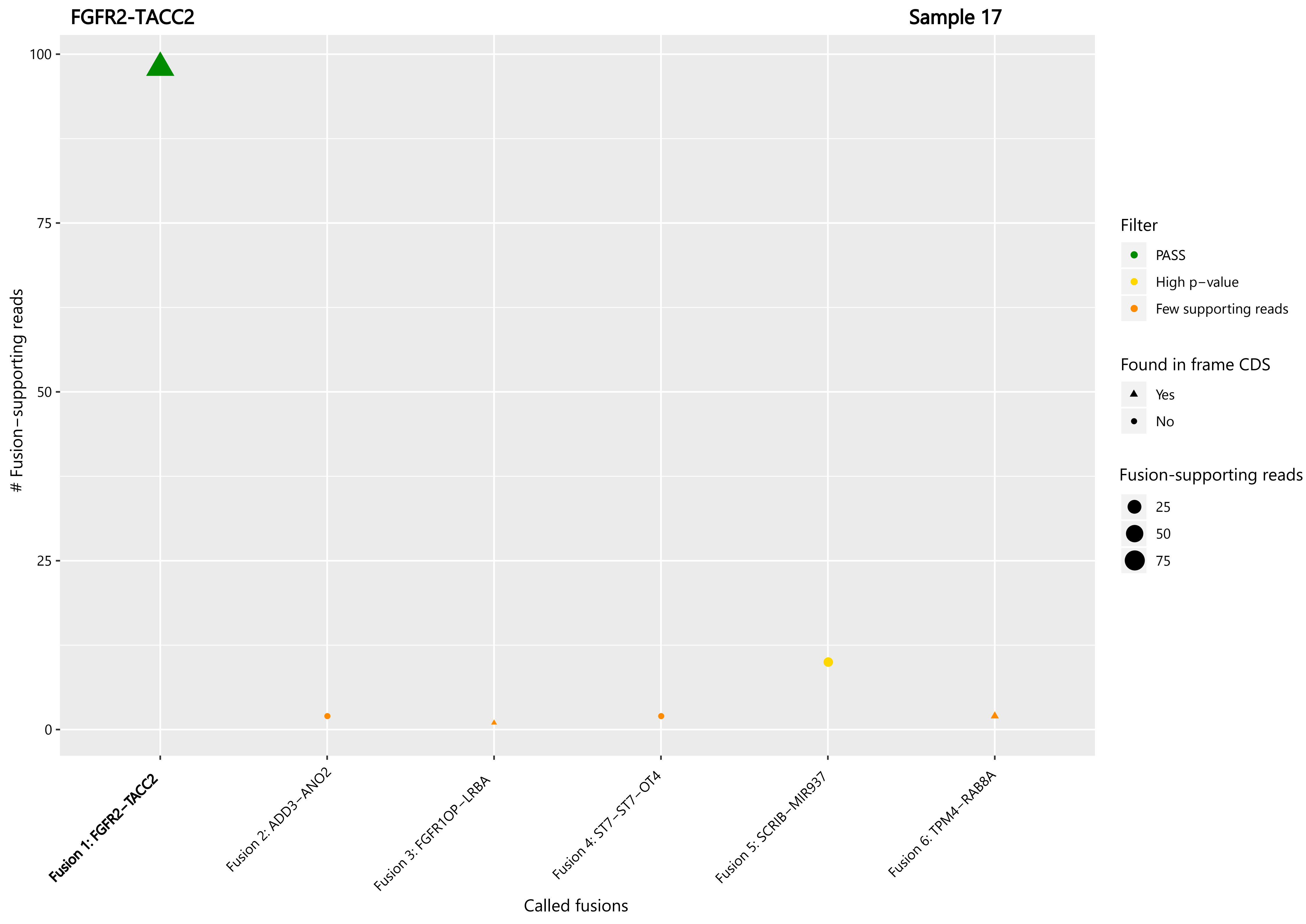
FGFR2-TACC2
Sample 17
Fusion−supporting reads
100
75
50
25
0
Fusion 1: FGFR2−TACC2
Fusion 2: ADD3−ANO2
Fusion 3: FGFR1OP−LRBA
Fusion 4: ST7−ST7−OT4
Fusion 5: SCRIB−MIR937
Fusion 6: TPM4−RAB8A
Called fusions
Filter
PASS
High p−value
Few supporting reads
Found in frame CDS
Yes
No
Fusion-supporting reads
25
50
75

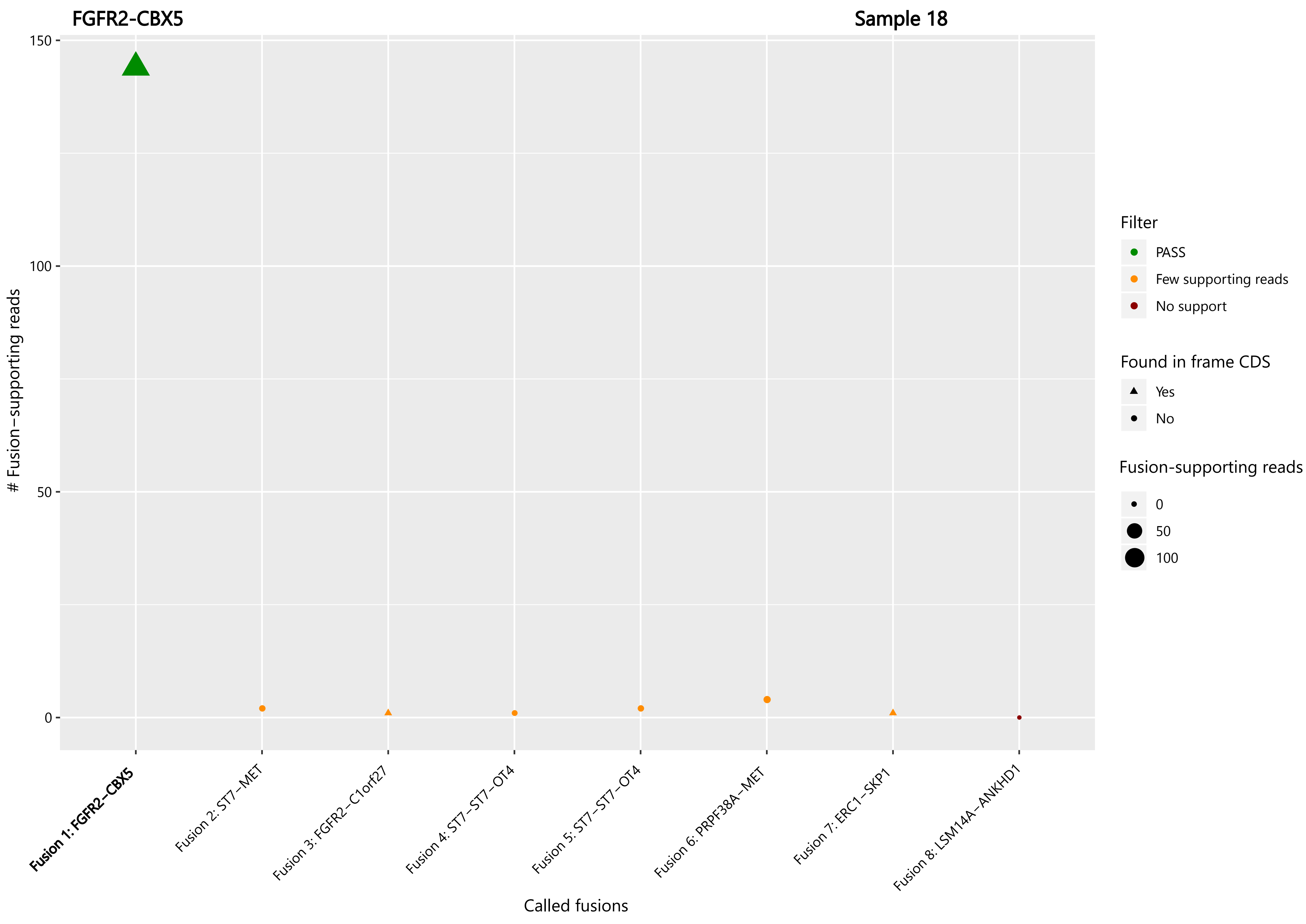
FGFR2-CBX5
Sample 18
150
100
50
0
Fusion−supporting reads
Fusion 1: FGFR2−CBX5
Fusion 2: ST7−MET
Fusion 3: FGFR2−C1orf27
Fusion 4: ST7−ST7−OT4
Fusion 5: ST7−ST7−OT4
Fusion 6: PRPF38A−MET
Fusion 7: ERC1−SKP1
Fusion 8: LSM14A−ANKHD1
Called fusions
Filter
PASS
Few supporting reads
No support
Found in frame CDS
Yes
No
Fusion-supporting reads
0
50
100
