# Supplementary material for: Detection of gene fusions using targeted next-generation sequencing: a comparative evaluation
Source: BMC Med Genomics. 2021 Feb 27;14:62. doi: 10.1186/s12920-021-00909-y (PMC7912891; doi:10.1186/s12920-021-00909-y)
Supplement: Supplementary file 13 — Additional file 13: Fig. S13. Fusions detected with the Oncomine Focus Assay (Thermo Fisher Scientific) for all samples. Metrics such as quality control scores, in-frame status or filter thresholds were plotted when available. In cases where the same fusion was identified more than once within the same sample, a unique numbering scheme was added at the end of the name to differentiate the candidate fusions. The numbering however, does not imply any special order or preference over the other fusions with the same name. The putative detected fusions were arranged in decreasing order based on the number of fusion-supporting reads. The expected fusion for each sample was highlighted in bold. [file 12920_2021_909_MOESM13_ESM.pdf]

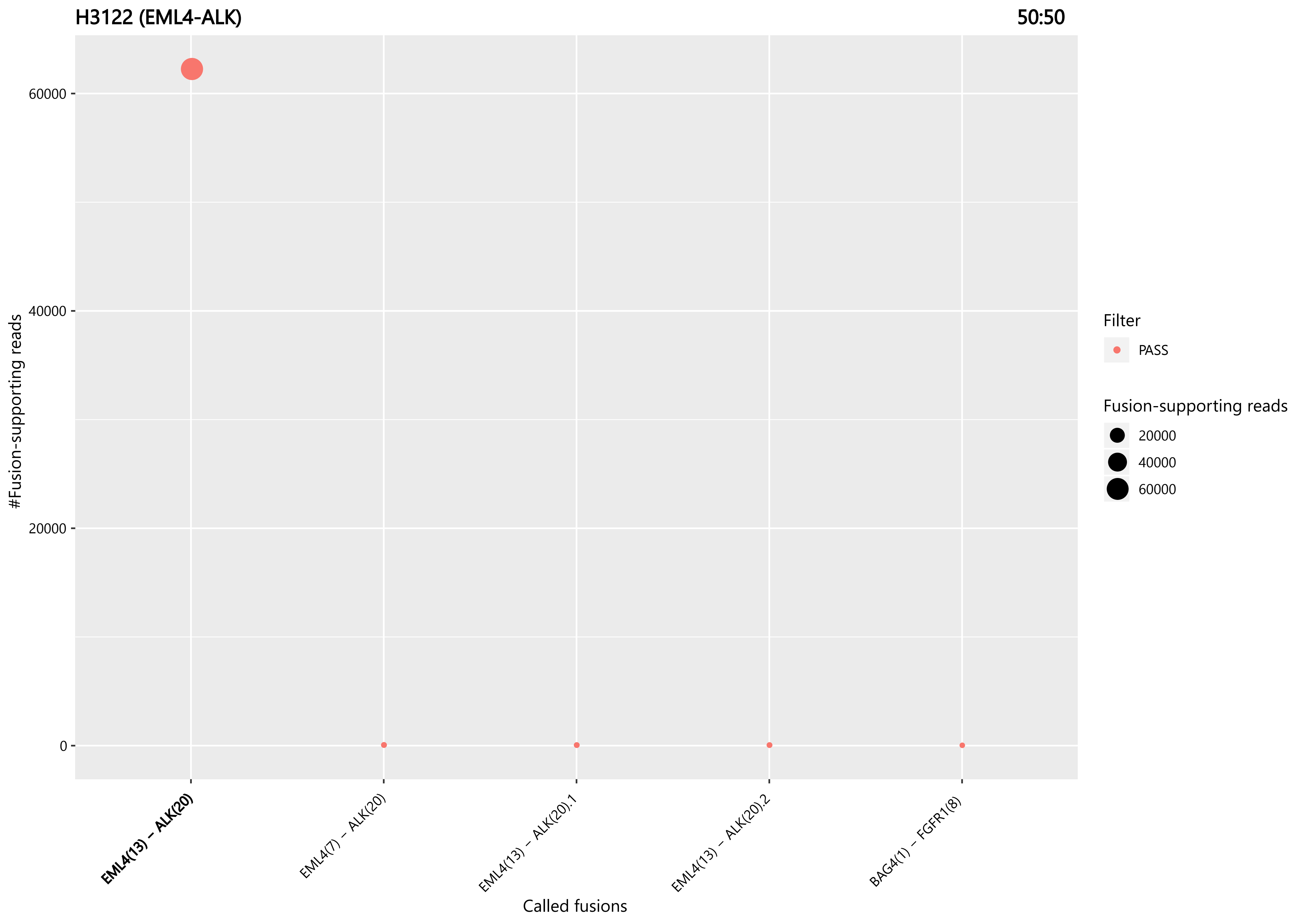

H3122 (EML4-ALK)
50:50
#Fusion-supporting reads
60000
40000
20000
0
EML4(13) – ALK(20)
EML4(7) – ALK(20)
EML4(13) – ALK(20).1
EML4(13) – ALK(20).2
BAG4(1) – FGFR1(8)
Called fusions
Filter
PASS
Fusion-supporting reads
20000
40000
60000

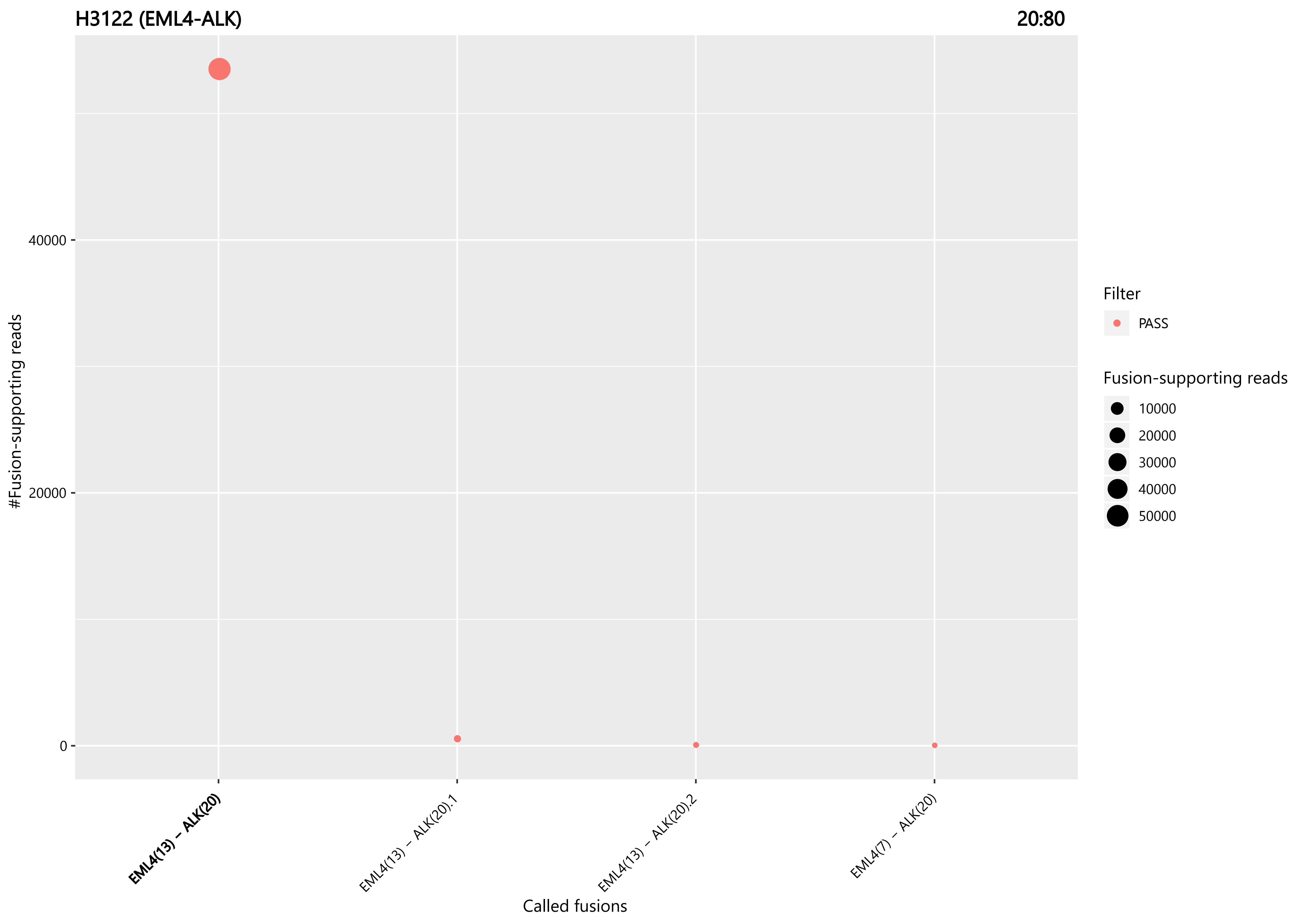

H3122 (EML4-ALK)
20:80
#Fusion-supporting reads
40000
20000
0
EML4(13) – ALK(20)
EML4(13) – ALK(20).1
EML4(13) – ALK(20).2
EML4(7) – ALK(20)
Called fusions
Filter
PASS
Fusion-supporting reads
10000
20000
30000
40000
50000

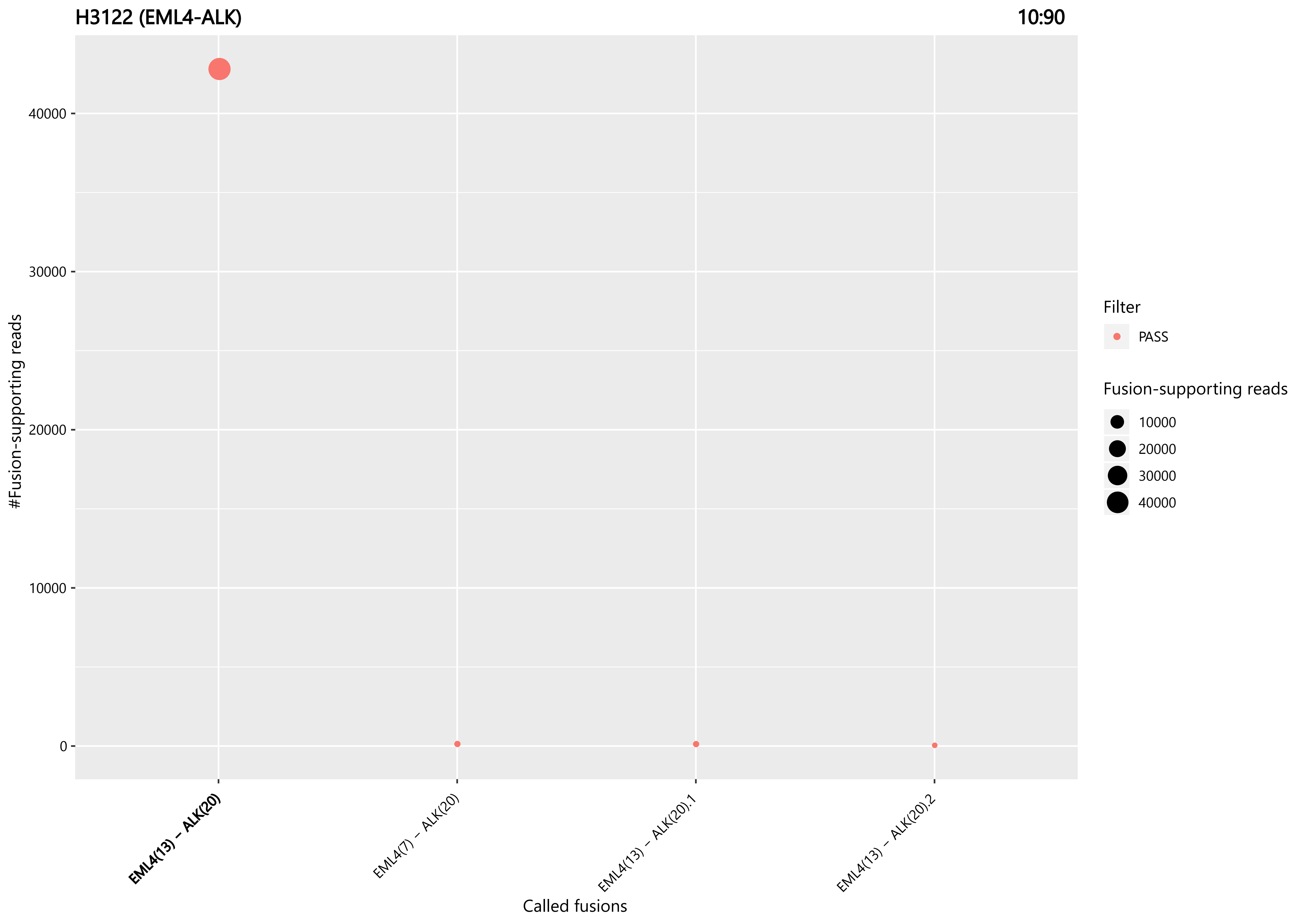
H3122 (EML4-ALK)
10:90
#Fusion-supporting reads
40000
30000
20000
10000
0
EML4(13) – ALK(20)
EML4(7) – ALK(20)
EML4(13) – ALK(20).1
EML4(13) – ALK(20).2
Called fusions
Filter
PASS
Fusion-supporting reads
10000
20000
30000
40000

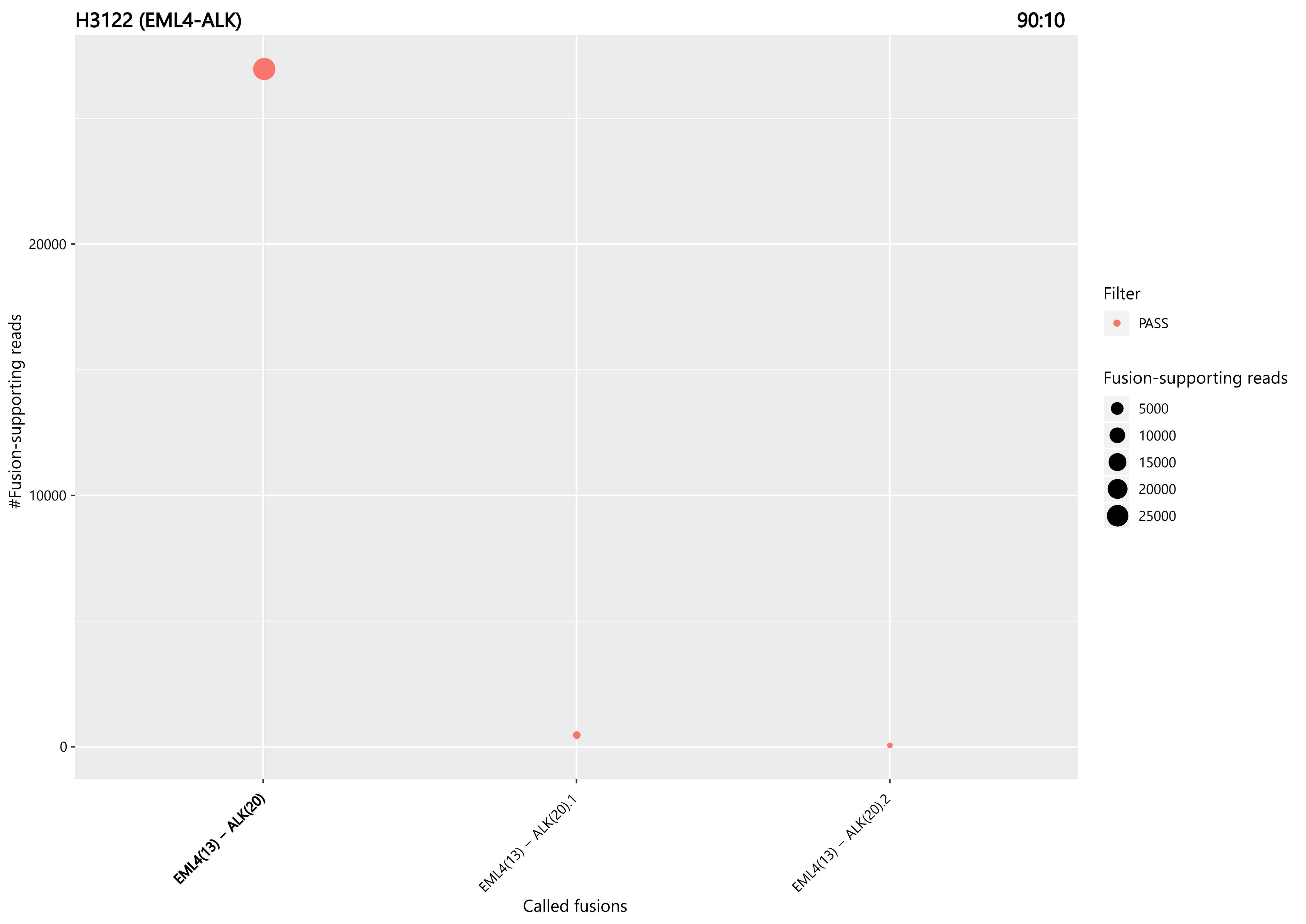

H3122 (EML4-ALK)
90:10
#Fusion-supporting reads
20000
10000
0
EML4(13) – ALK(20)
EML4(13) – ALK(20).1
EML4(13) – ALK(20).2
Called fusions
Filter
PASS
Fusion-supporting reads
5000
10000
15000
20000
25000

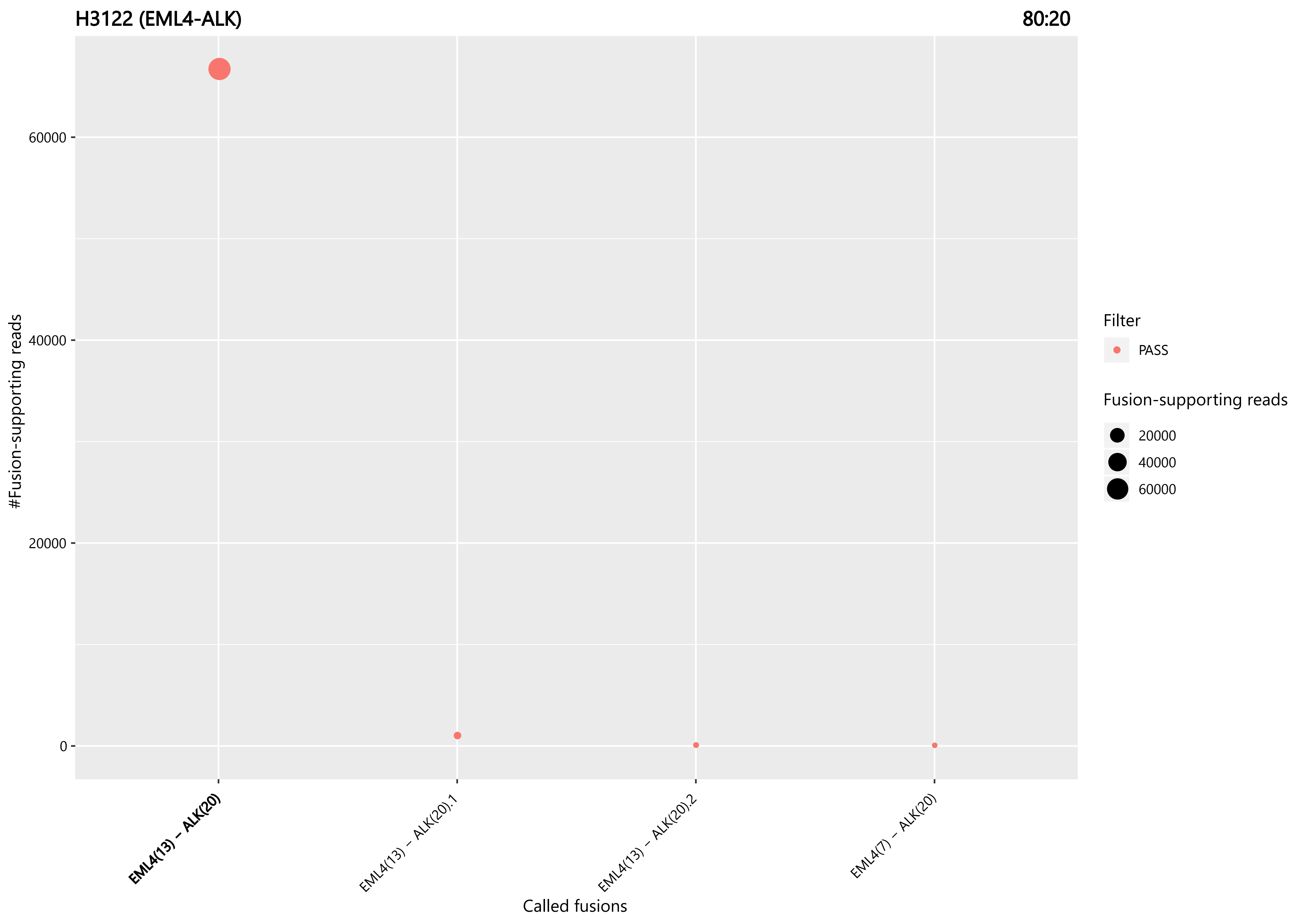

H3122 (EML4-ALK)
80:20
60000
40000
20000
0
#Fusion-supporting reads
EML4(13) – ALK(20)
EML4(13) – ALK(20).1
EML4(13) – ALK(20).2
EML4(7) – ALK(20)
Called fusions
Filter
PASS
Fusion-supporting reads
20000
40000
60000

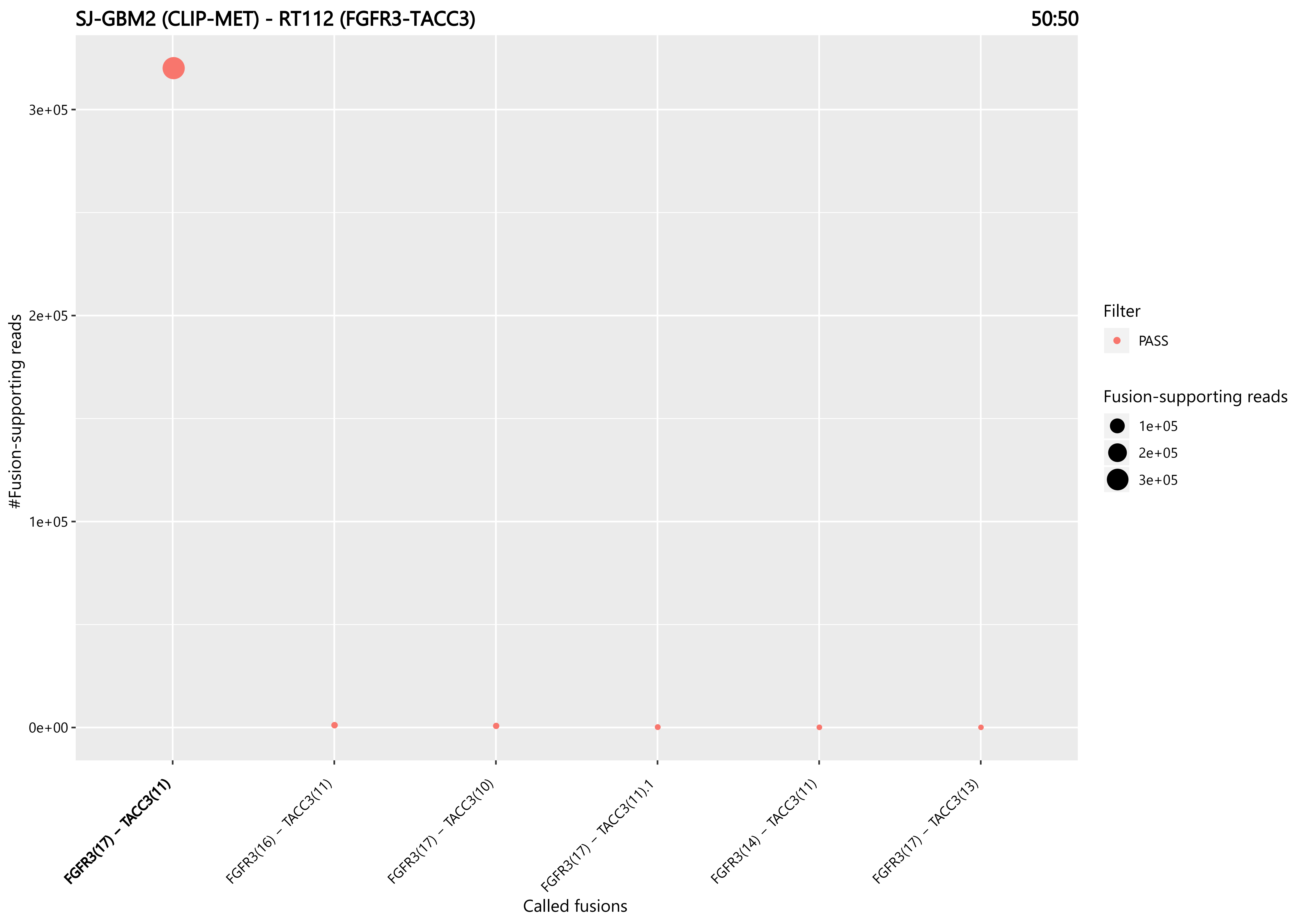

SJ-GBM2 (CLIP-MET) - RT112 (FGFR3-TACC3)
50:50
#Fusion-supporting reads
3e+05
2e+05
1e+05
0e+00
FGFR3(17) – TACC3(11)
FGFR3(16) – TACC3(11)
FGFR3(17) – TACC3(10)
FGFR3(17) – TACC3(11).1
FGFR3(14) – TACC3(11)
FGFR3(17) – TACC3(13)
Called fusions
Filter
PASS
Fusion-supporting reads
1e+05
2e+05
3e+05

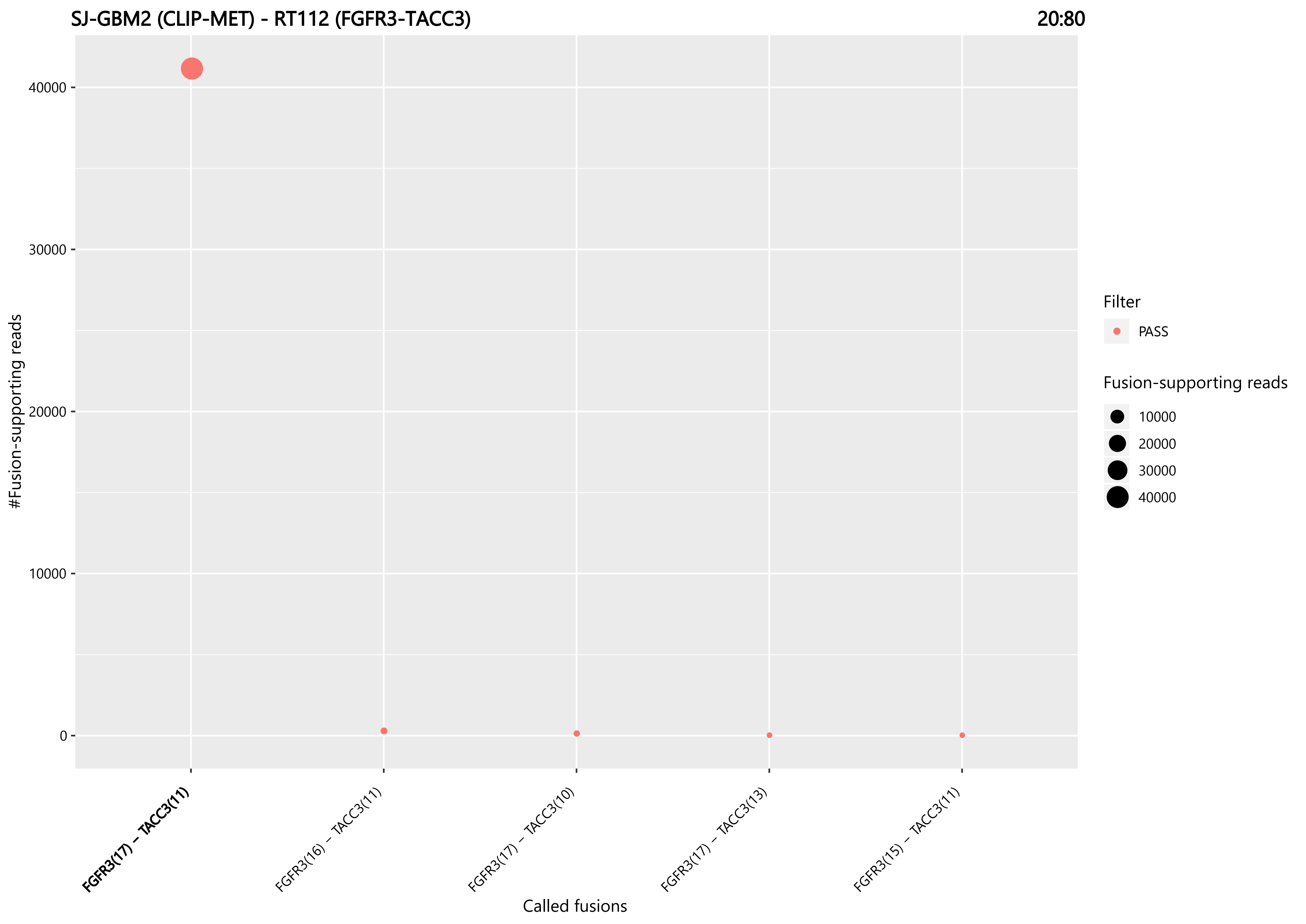
SJ-GBM2 (CLIP-MET) - RT112 (FGFR3-TACC3)
20:80
#Fusion-supporting reads
40000
30000
20000
10000
0
FGFR3(17) – TACC3(11)
FGFR3(16) – TACC3(11)
FGFR3(17) – TACC3(10)
FGFR3(17) – TACC3(13)
FGFR3(15) – TACC3(11)
Called fusions
Filter
PASS
Fusion-supporting reads
10000
20000
30000
40000

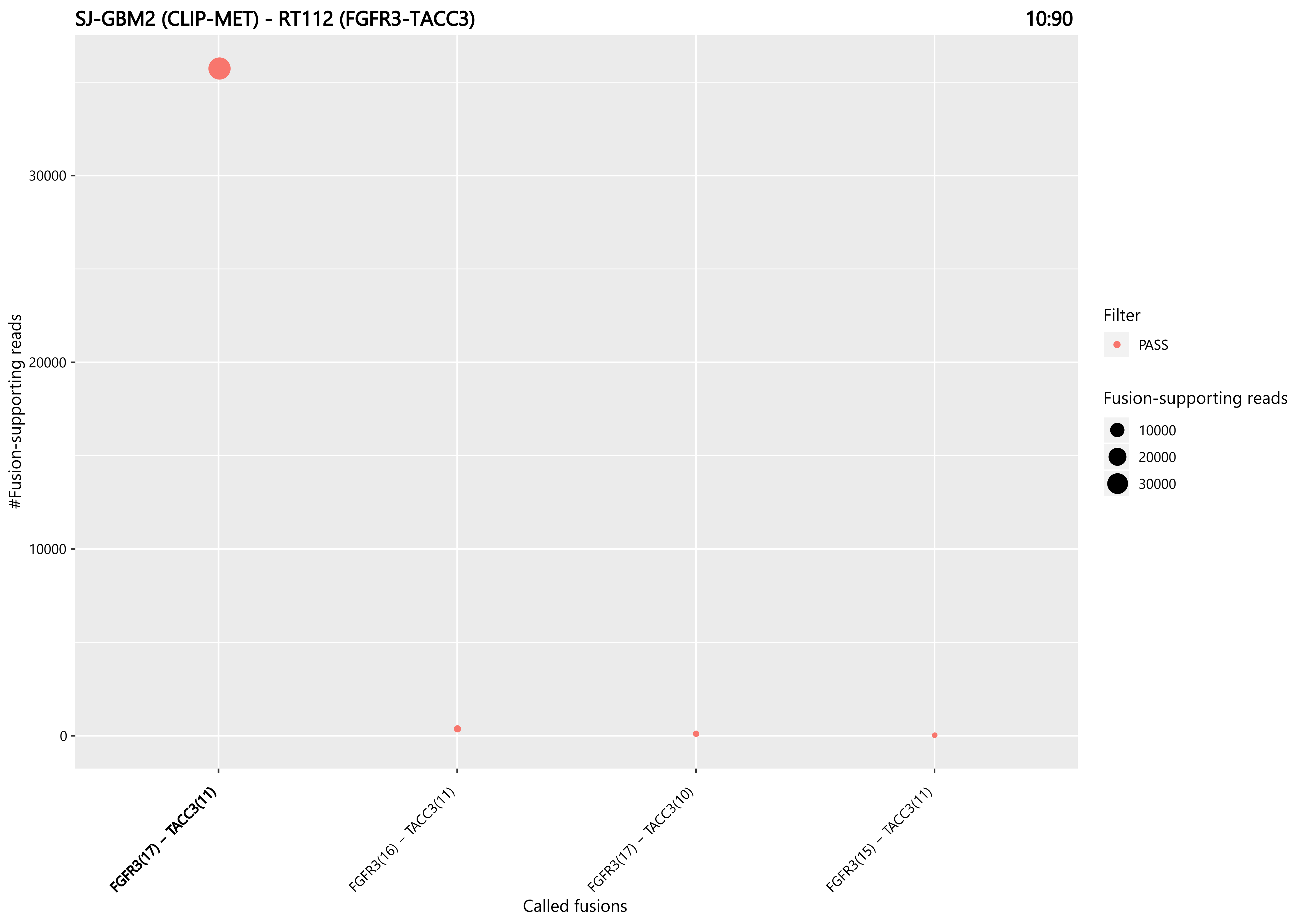

SJ-GBM2 (CLIP-MET) - RT112 (FGFR3-TACC3)
10:90
#Fusion-supporting reads
30000
20000
10000
0
FGFR3(17) – TACC3(11)
FGFR3(16) – TACC3(11)
FGFR3(17) – TACC3(10)
FGFR3(15) – TACC3(11)
Called fusions
Filter
PASS
Fusion-supporting reads
10000
20000
30000

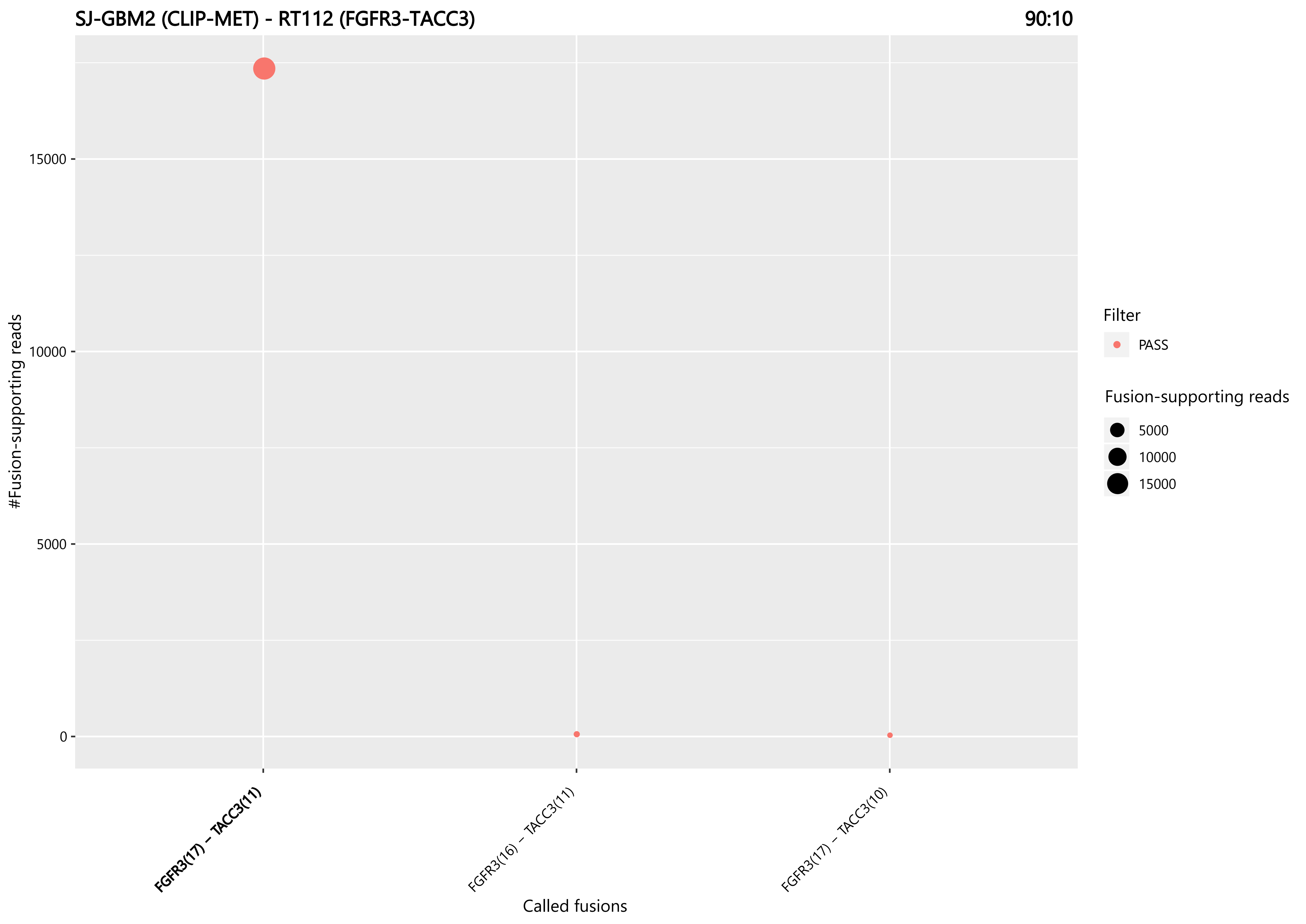
SJ-GBM2 (CLIP-MET) - RT112 (FGFR3-TACC3)
90:10
#Fusion-supporting reads
15000
10000
5000
0
FGFR3(17) – TACC3(11)
FGFR3(16) – TACC3(11)
FGFR3(17) – TACC3(10)
Called fusions
Filter
PASS
Fusion-supporting reads
5000
10000
15000

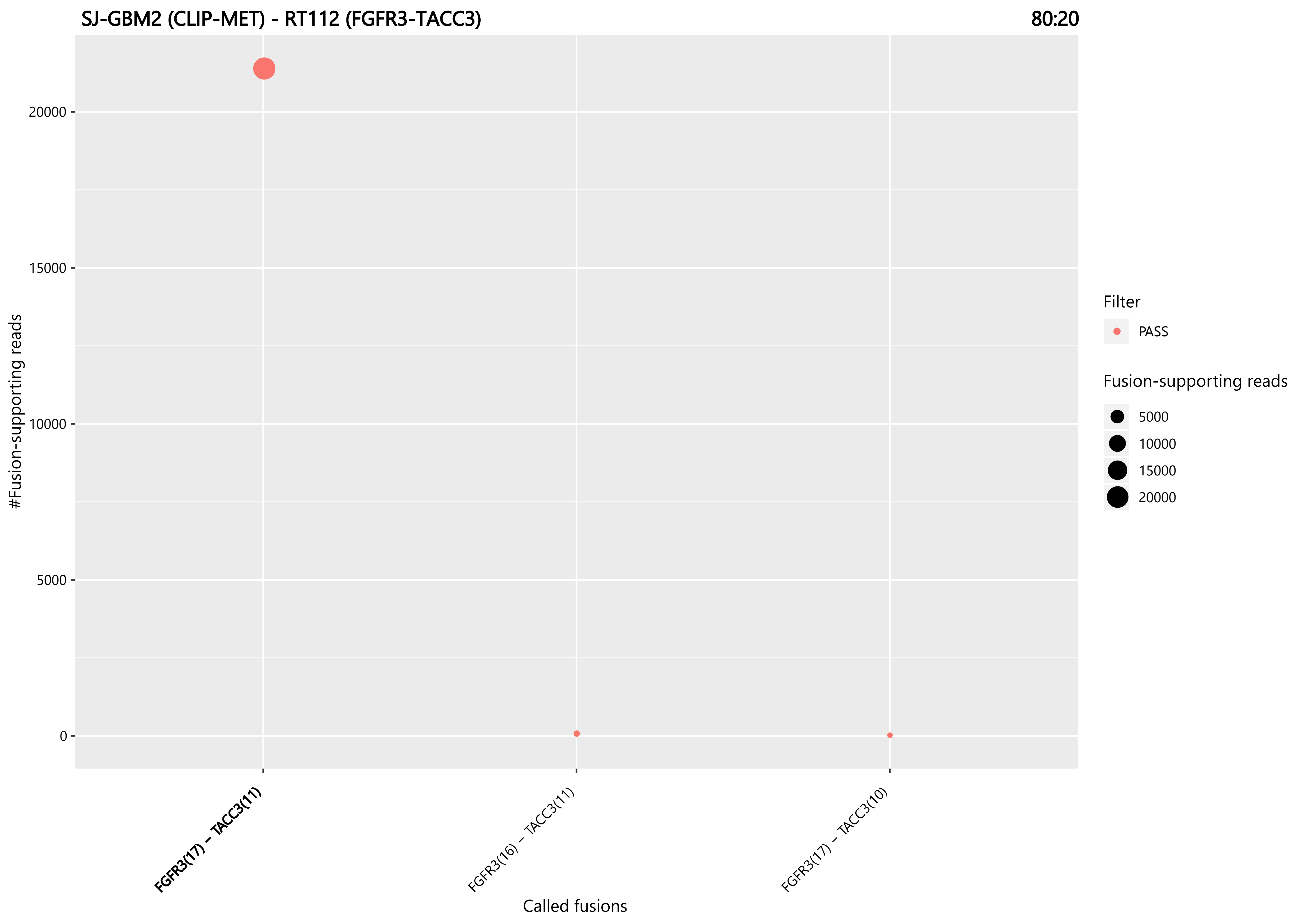

SJ-GBM2 (CLIP-MET) - RT112 (FGFR3-TACC3)
80:20
#Fusion-supporting reads
20000
15000
10000
5000
0
FGFR3(17) – TACC3(11)
FGFR3(16) – TACC3(11)
FGFR3(17) – TACC3(10)
Called fusions
Filter
PASS
Fusion-supporting reads
5000
10000
15000
20000

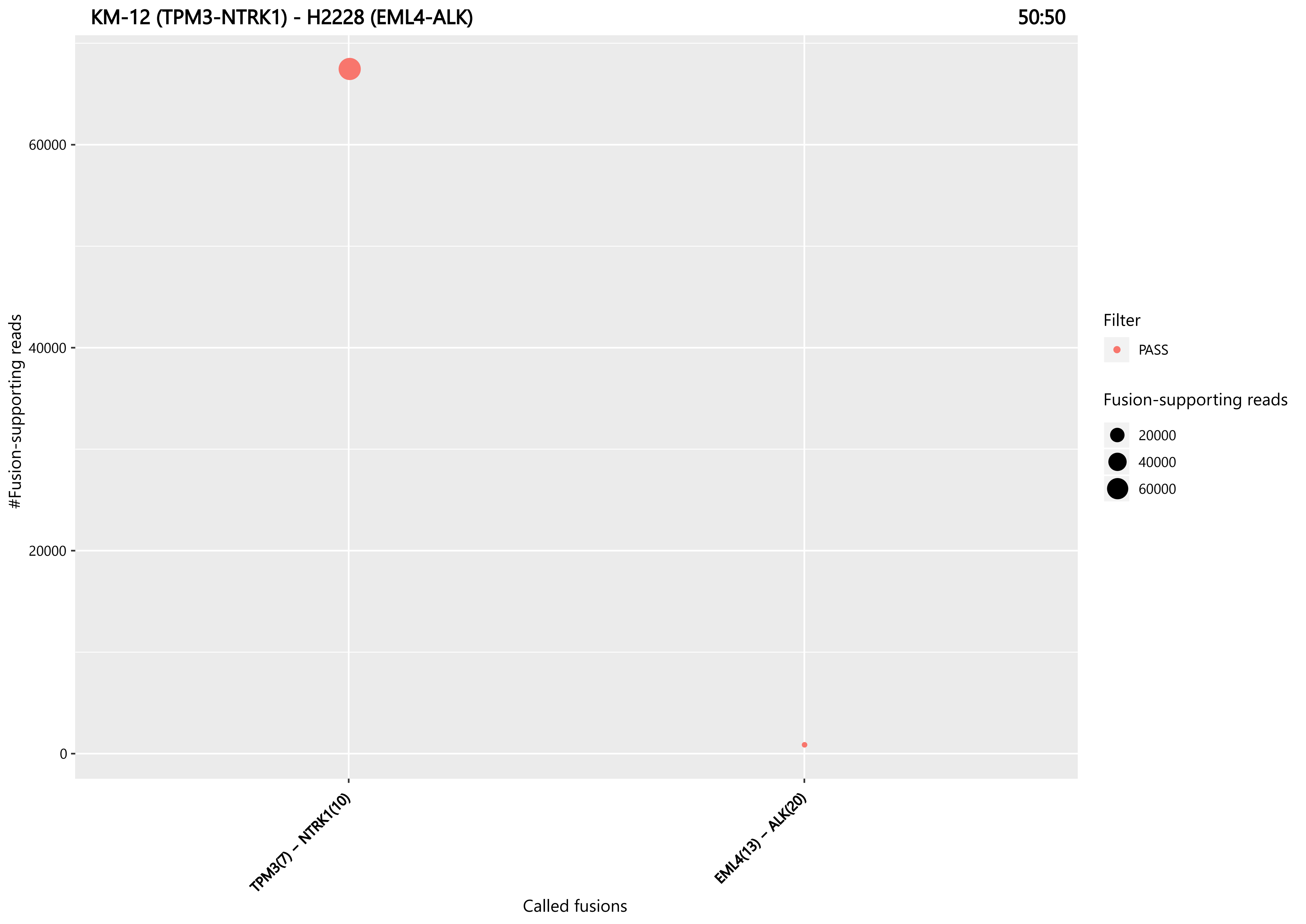

KM-12 (TPM3-NTRK1) - H2228 (EML4-ALK)
50:50
#Fusion-supporting reads
60000
40000
20000
0
TPM3(7) – NTRK1(10)
EML4(13) – ALK(20)
Called fusions
Filter
PASS
Fusion-supporting reads
20000
40000
60000

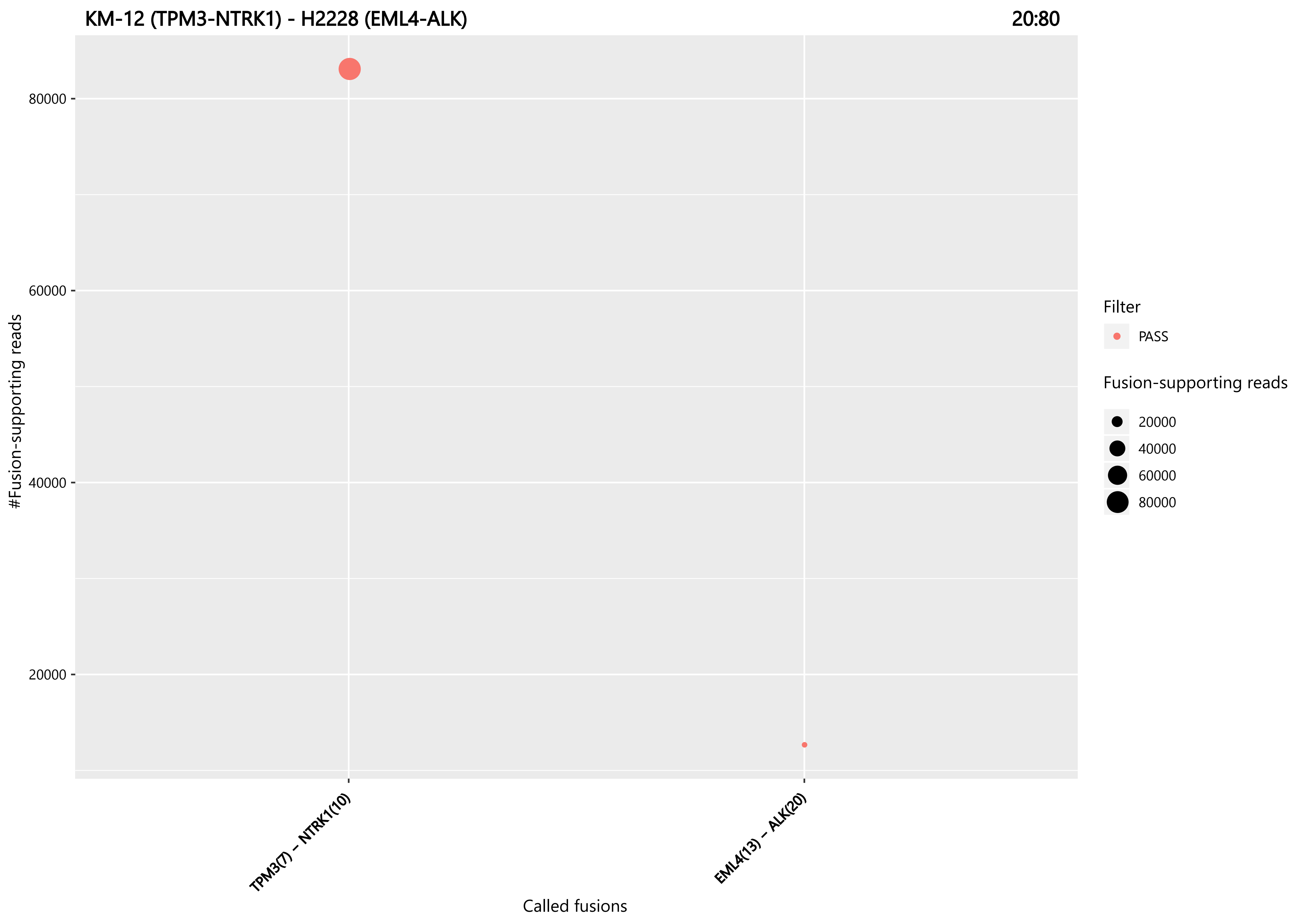

KM-12 (TPM3-NTRK1) - H2228 (EML4-ALK)
20:80
#Fusion-supporting reads
80000
60000
40000
20000
TPM3(7) – NTRK1(10)
EML4(13) – ALK(20)
Called fusions
Filter
PASS
Fusion-supporting reads
20000
40000
60000
80000

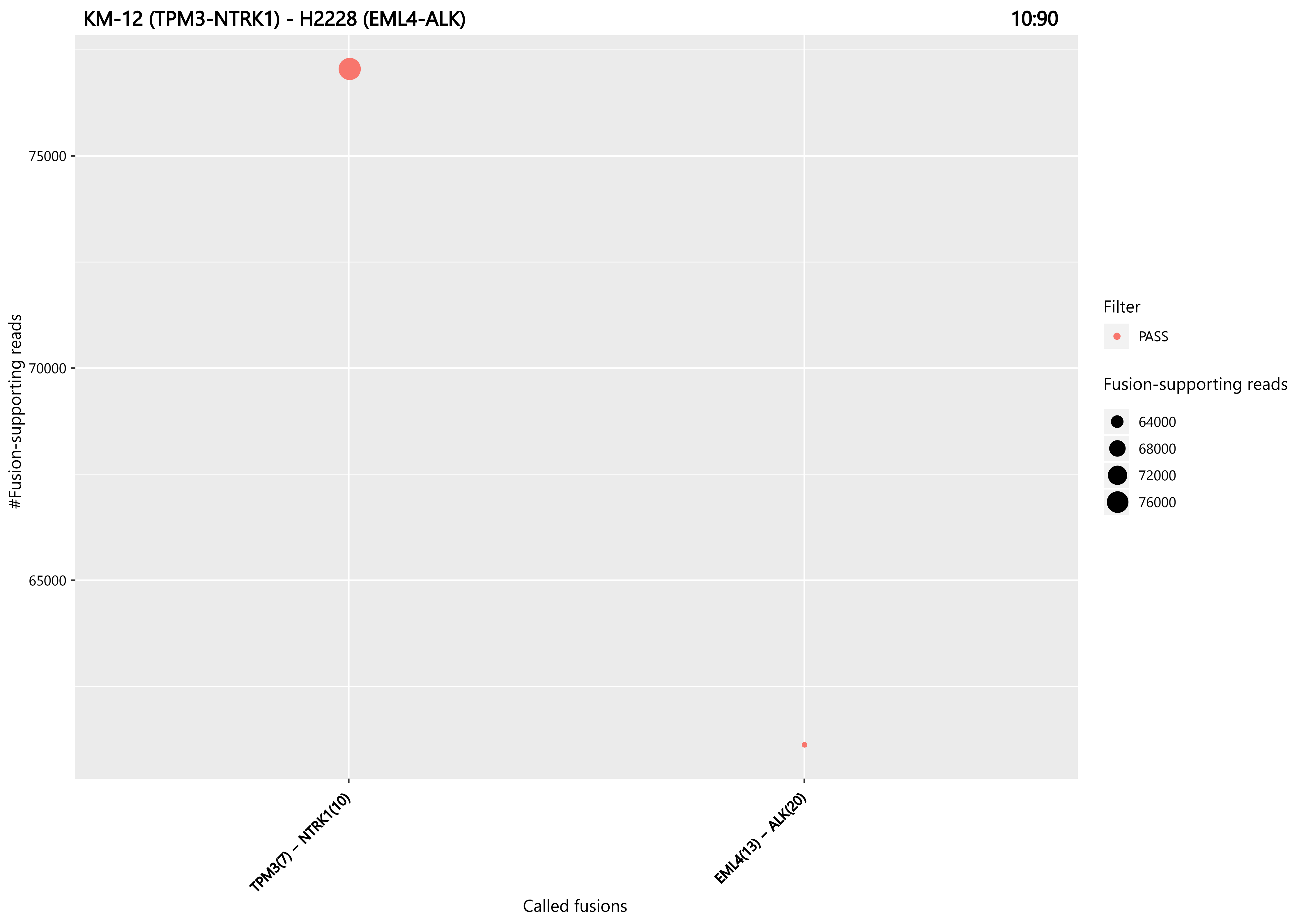
KM-12 (TPM3-NTRK1) - H2228 (EML4-ALK)
10:90
75000
70000
65000
#Fusion-supporting reads
TPM3(7) – NTRK1(10)
EML4(13) – ALK(20)
Called fusions
Filter
PASS
Fusion-supporting reads
64000
68000
72000
76000

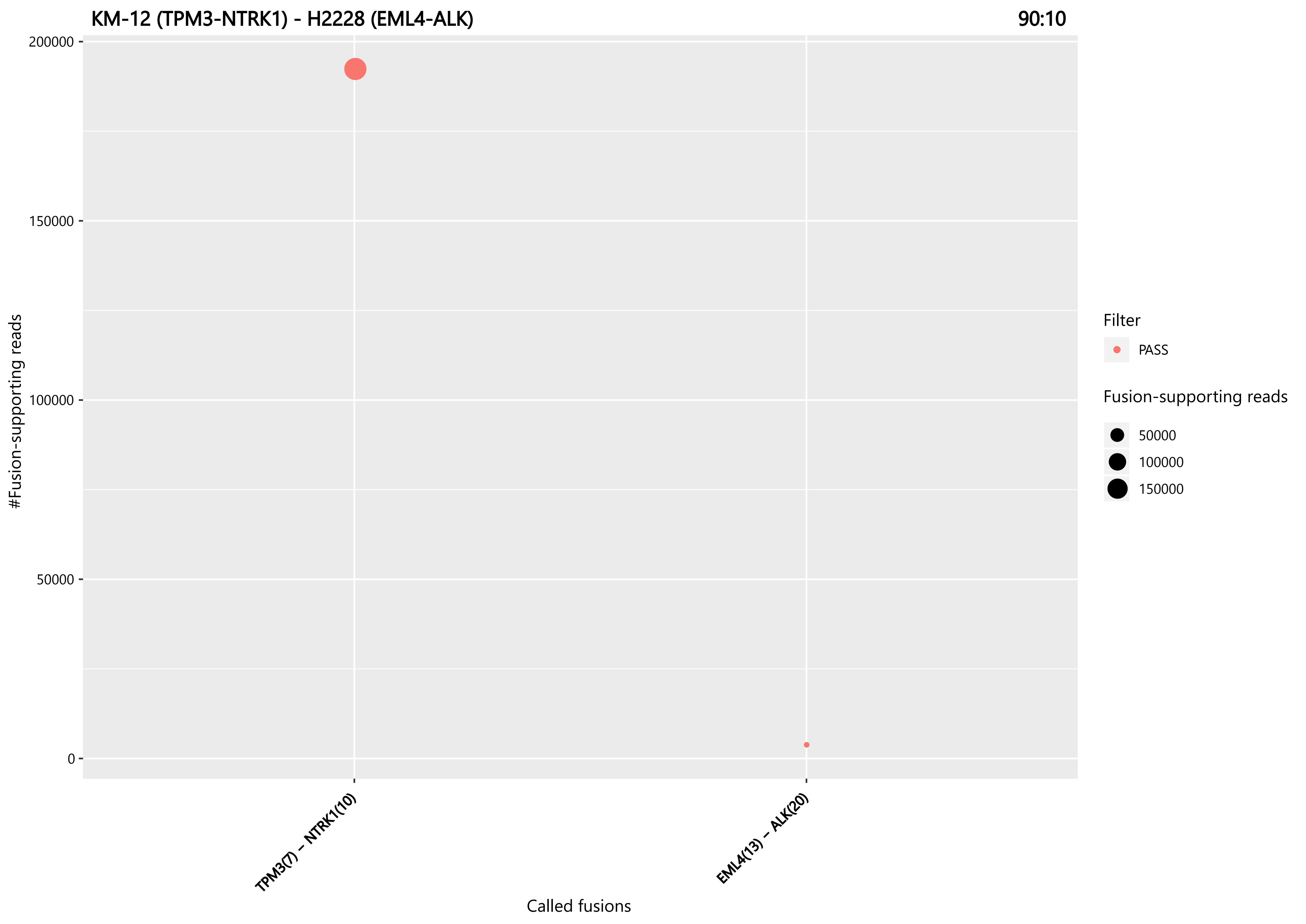

KM-12 (TPM3-NTRK1) - H2228 (EML4-ALK)
90:10
200000
150000
100000
50000
0
#Fusion-supporting reads
TPM3(7) – NTRK1(10)
EML4(13) – ALK(20)
Called fusions
Filter
PASS
Fusion-supporting reads
50000
100000
150000

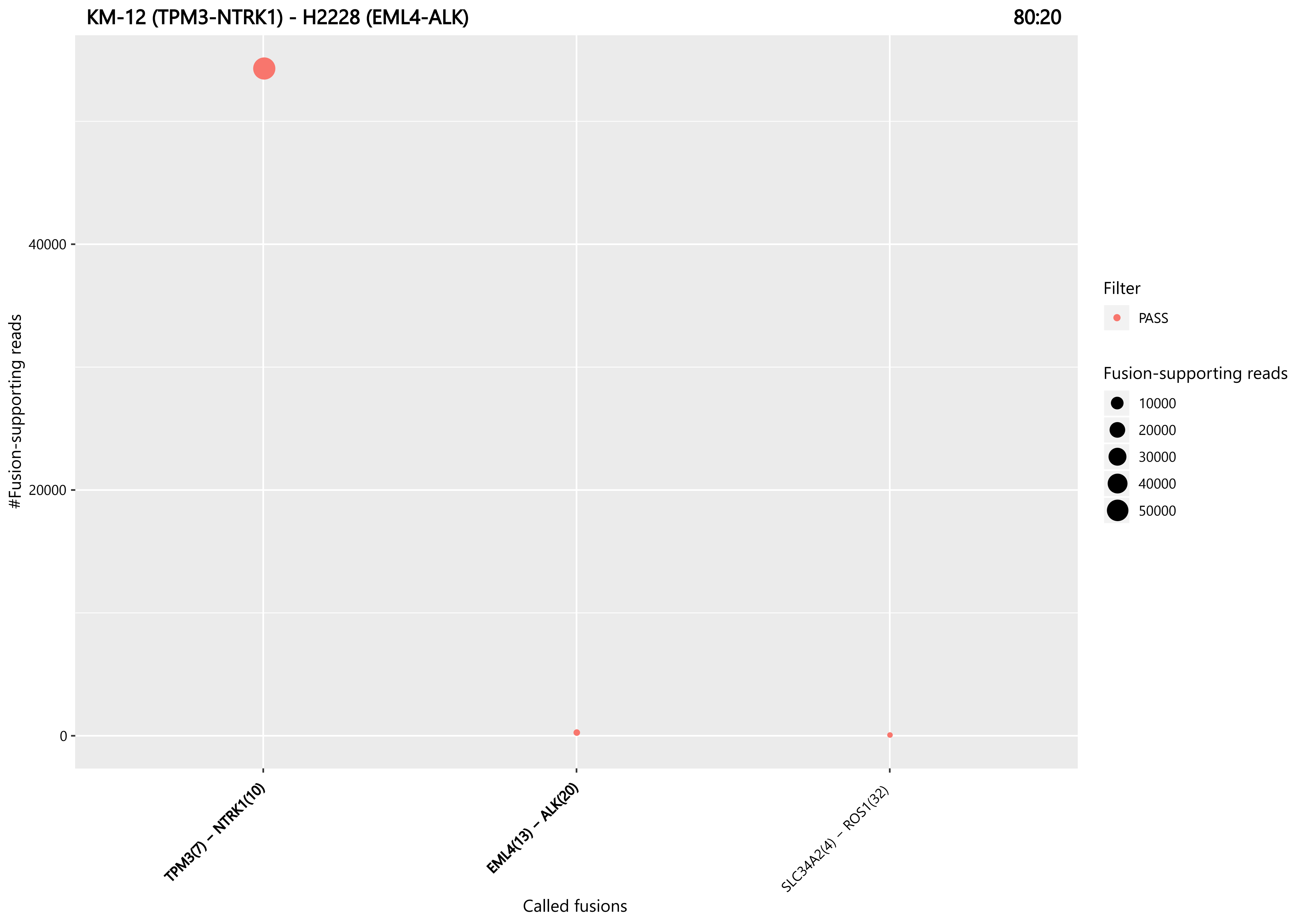

KM-12 (TPM3-NTRK1) - H2228 (EML4-ALK)
80:20
#Fusion-supporting reads
40000
20000
0
TPM3(7) – NTRK1(10)
EML4(13) – ALK(20)
SLC34A2(4) – ROS1(32)
Called fusions
Filter
PASS
Fusion-supporting reads
10000
20000
30000
40000
50000

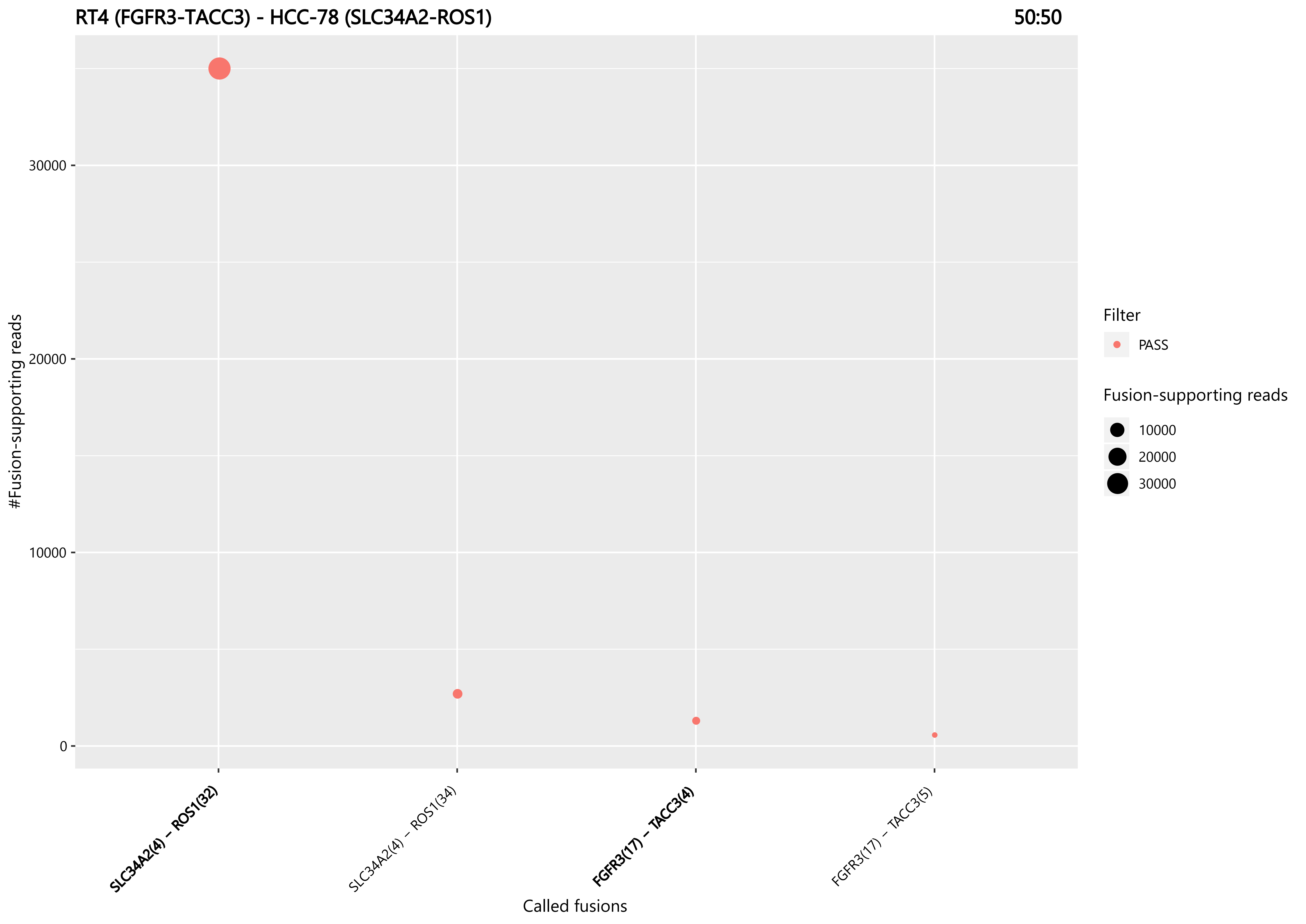

RT4 (FGFR3-TACC3) - HCC-78 (SLC34A2-ROS1)
50:50
#Fusion-supporting reads
30000
20000
10000
0
SLC34A2(4) – ROS1(32)
SLC34A2(4) – ROS1(34)
FGFR3(17) – TACC3(4)
FGFR3(17) – TACC3(5)
Called fusions
Filter
PASS
Fusion-supporting reads
10000
20000
30000

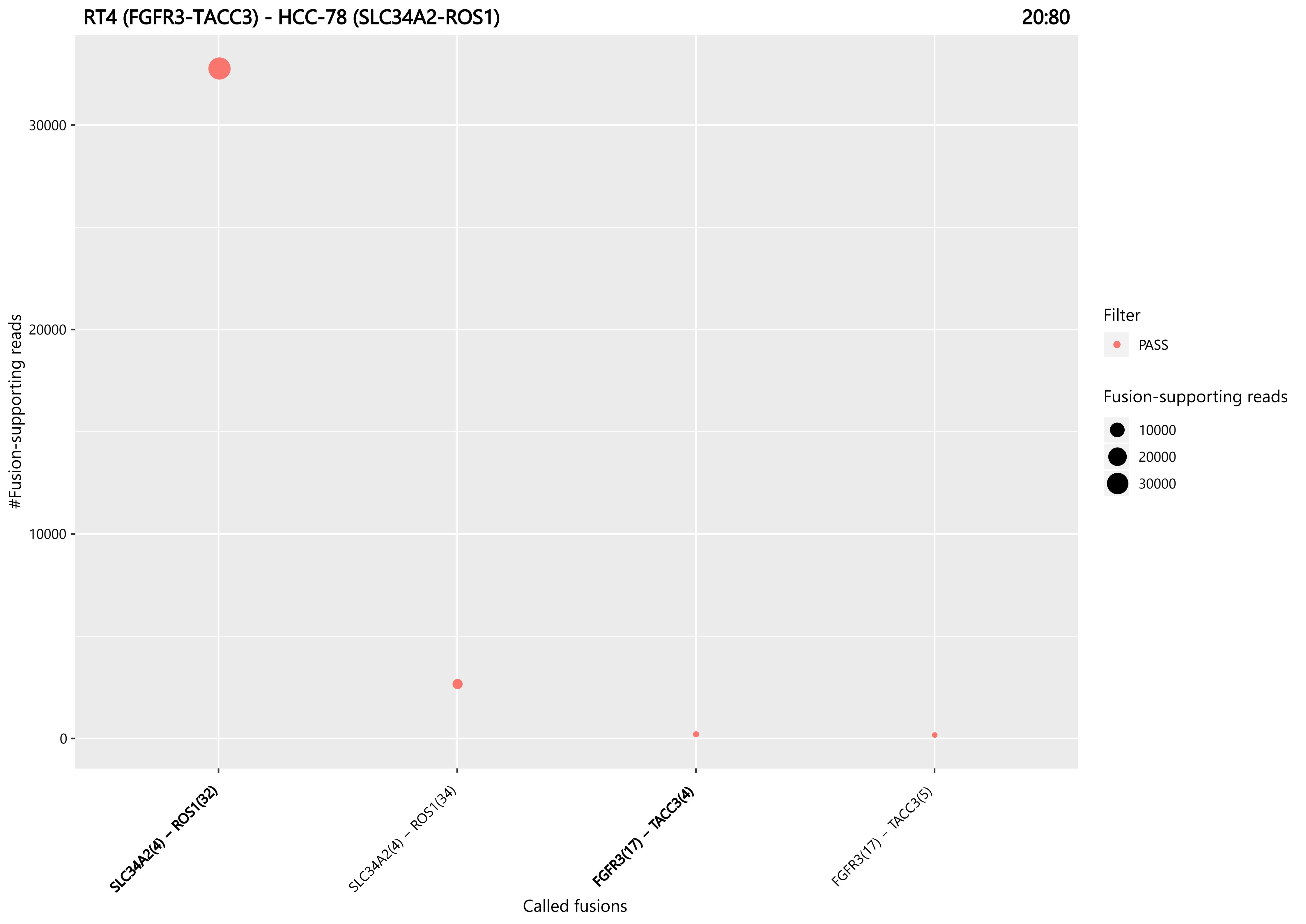

RT4 (FGFR3-TACC3) - HCC-78 (SLC34A2-ROS1)
20:80
#Fusion-supporting reads
30000
20000
10000
0
SLC34A2(4) – ROS1(32)
SLC34A2(4) – ROS1(34)
FGFR3(17) – TACC3(4)
FGFR3(17) – TACC3(5)
Called fusions
Filter
PASS
Fusion-supporting reads
10000
20000
30000

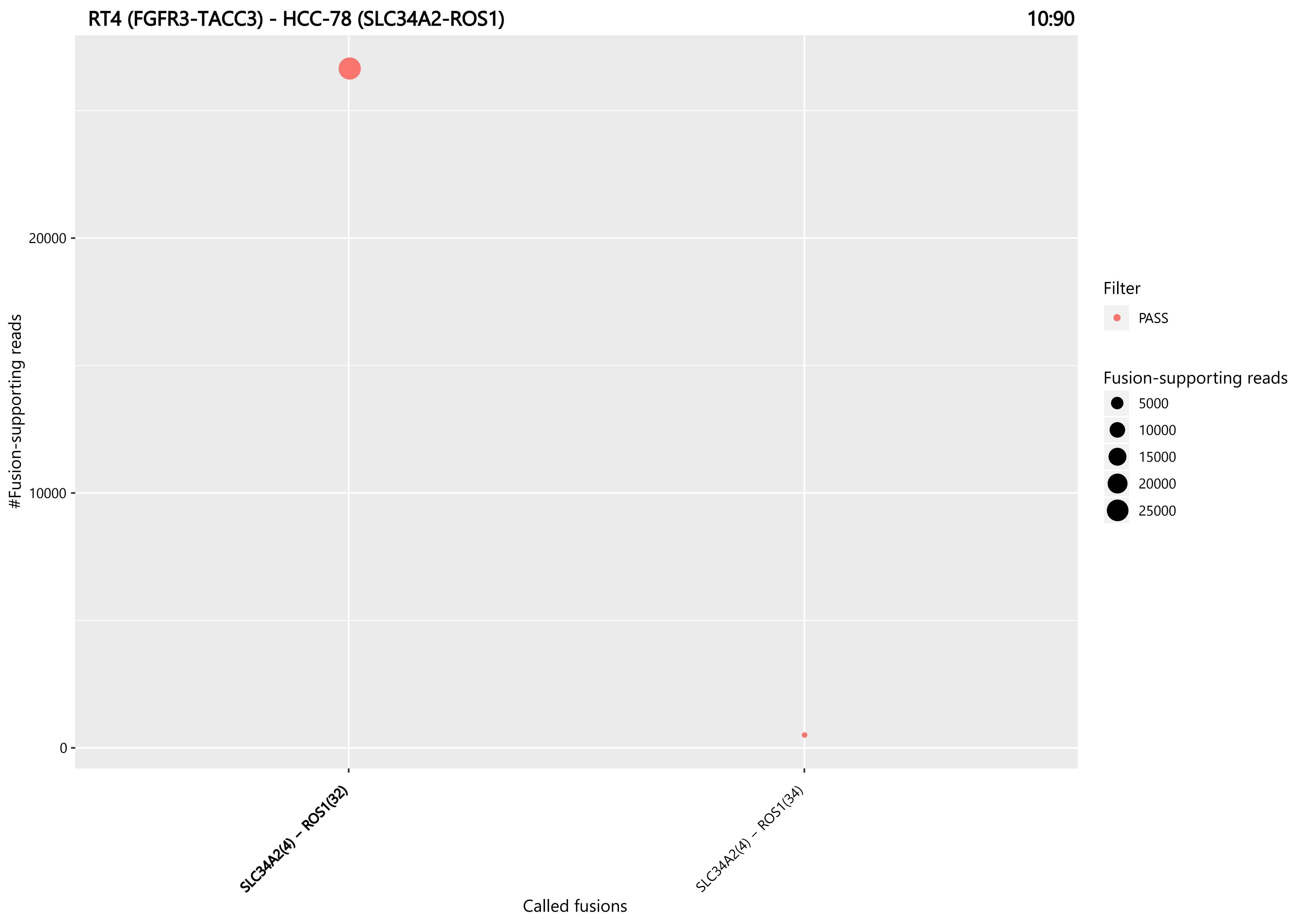

RT4 (FGFR3-TACC3) - HCC-78 (SLC34A2-ROS1)
10:90
#Fusion-supporting reads
20000
10000
0
Called fusions
SLC34A2(4) – ROS1(32)
SLC34A2(4) – ROS1(34)
Filter
PASS
Fusion-supporting reads
5000
10000
15000
20000
25000

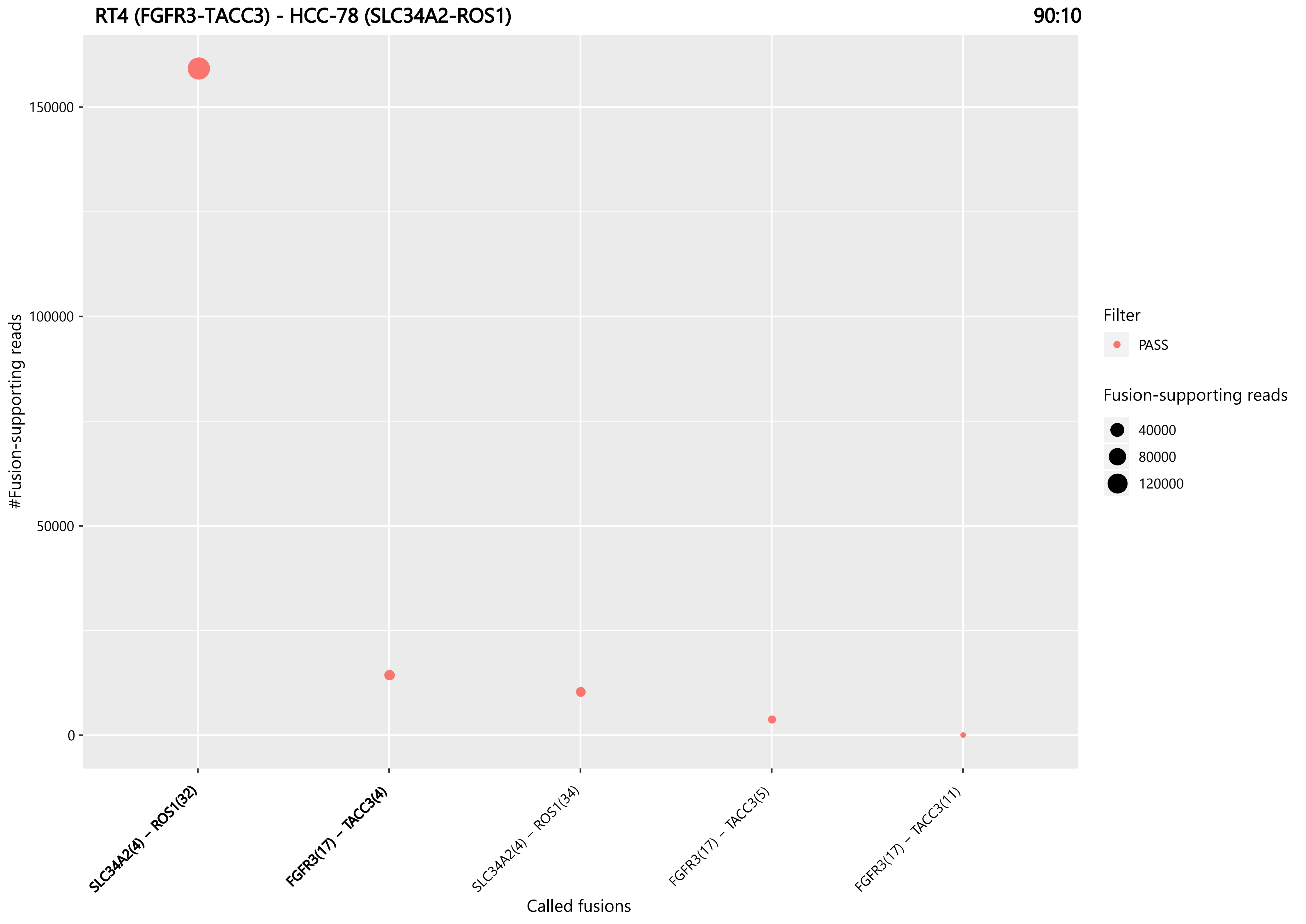
RT4 (FGFR3-TACC3) - HCC-78 (SLC34A2-ROS1)
90:10
#Fusion-supporting reads
150000
100000
50000
0
Called fusions
SLC34A2(4) – ROS1(32)
FGFR3(17) – TACC3(4)
SLC34A2(4) – ROS1(34)
FGFR3(17) – TACC3(5)
FGFR3(17) – TACC3(11)
Filter
PASS
Fusion-supporting reads
40000
80000
120000

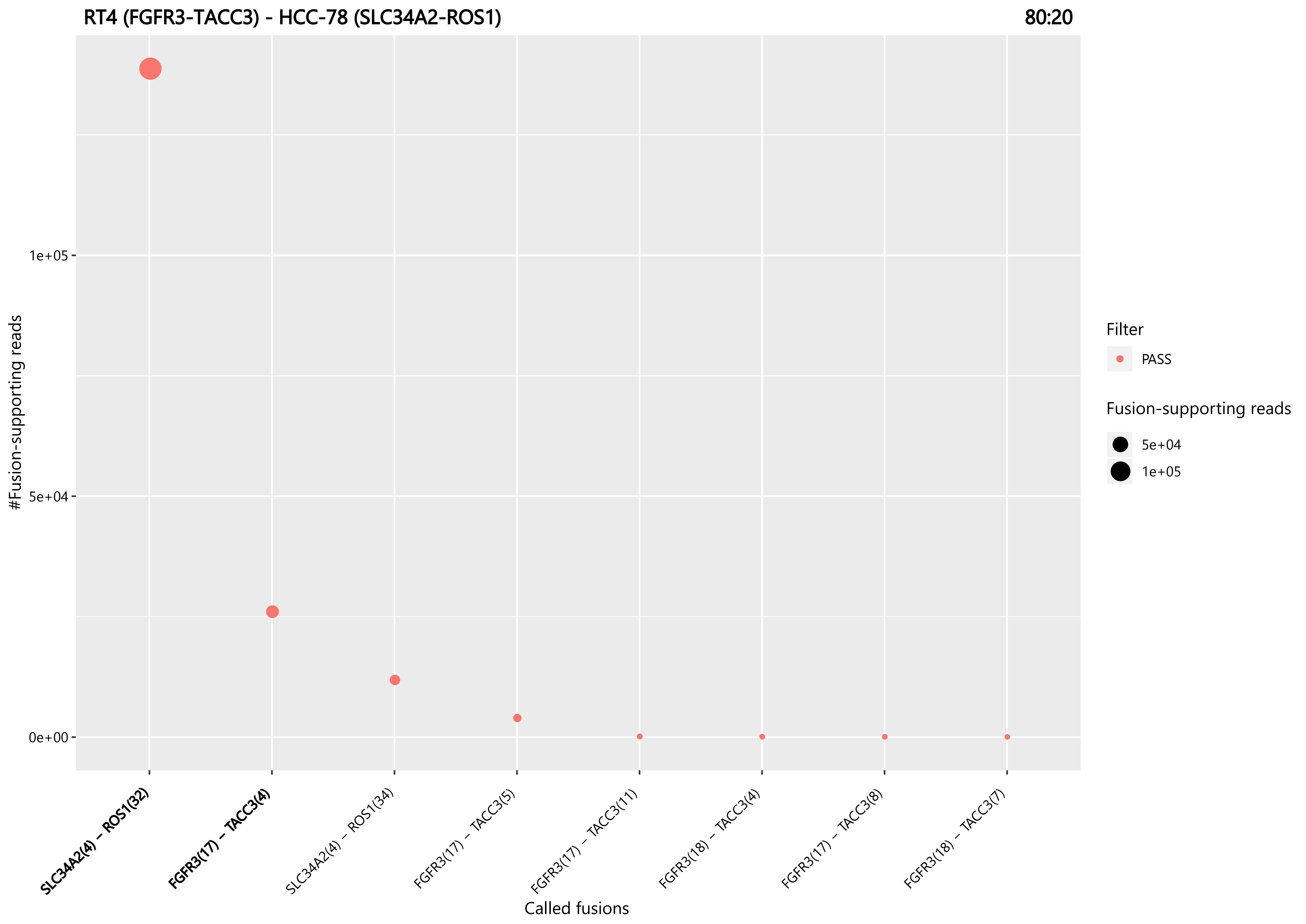
RT4 (FGFR3-TACC3) - HCC-78 (SLC34A2-ROS1)
80:20
#Fusion-supporting reads
1e+05
5e+04
0e+00
SLC34A2(4) – ROS1(32)
FGFR3(17) – TACC3(4)
SLC34A2(4) – ROS1(34)
FGFR3(17) – TACC3(5)
FGFR3(17) – TACC3(11)
FGFR3(18) – TACC3(4)
FGFR3(17) – TACC3(8)
FGFR3(18) – TACC3(7)
Called fusions
Filter
PASS
Fusion-supporting reads
5e+04
1e+05

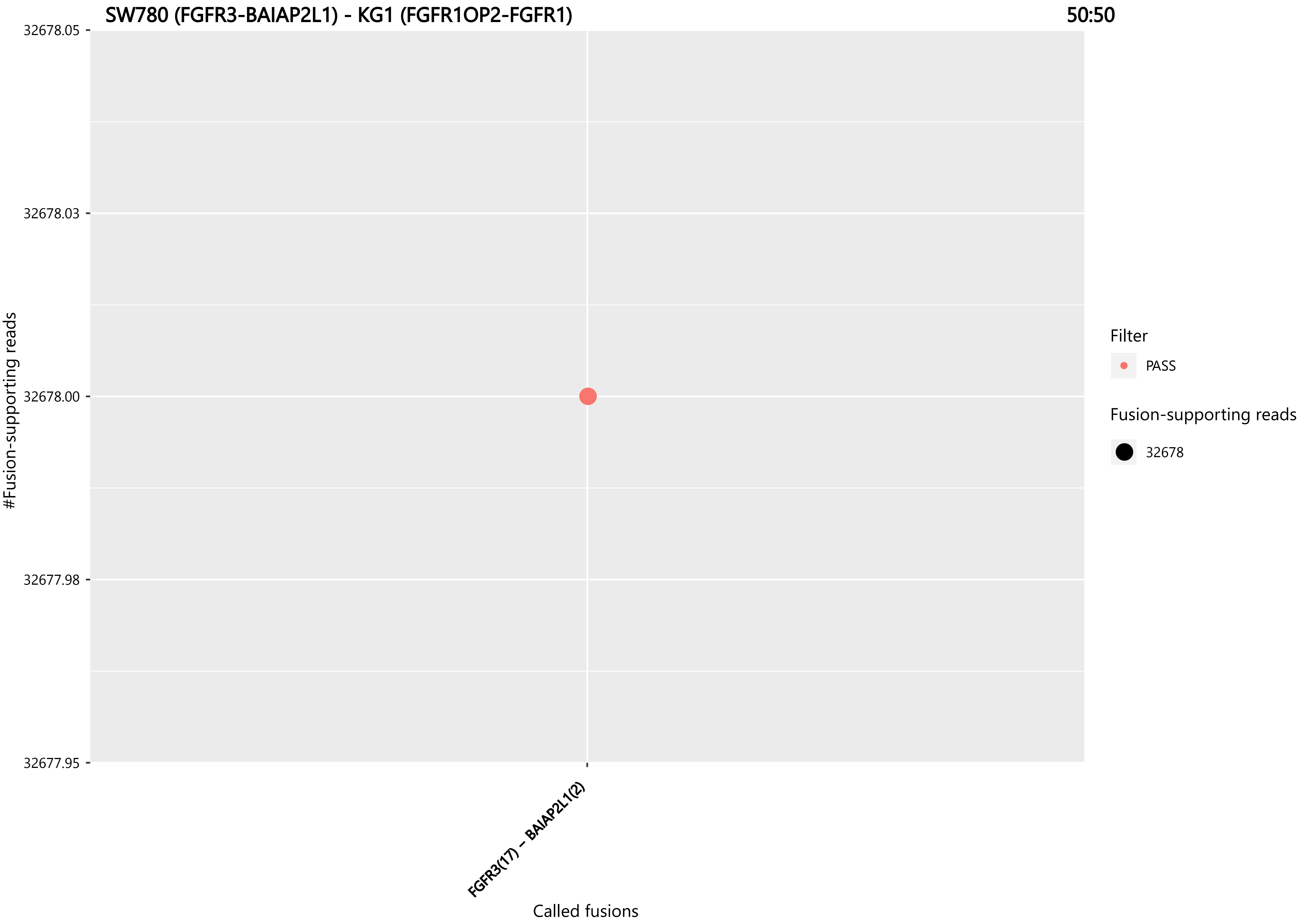
SW780 (FGFR3-BAIAP2L1) - KG1 (FGFR1OP2-FGFR1)
50:50
#Fusion-supporting reads
32678.05
32678.03
32678.00
32677.98
32677.95
FGFR3(17) – BAIAP2L1(2)
Called fusions
Filter
PASS
Fusion-supporting reads
32678

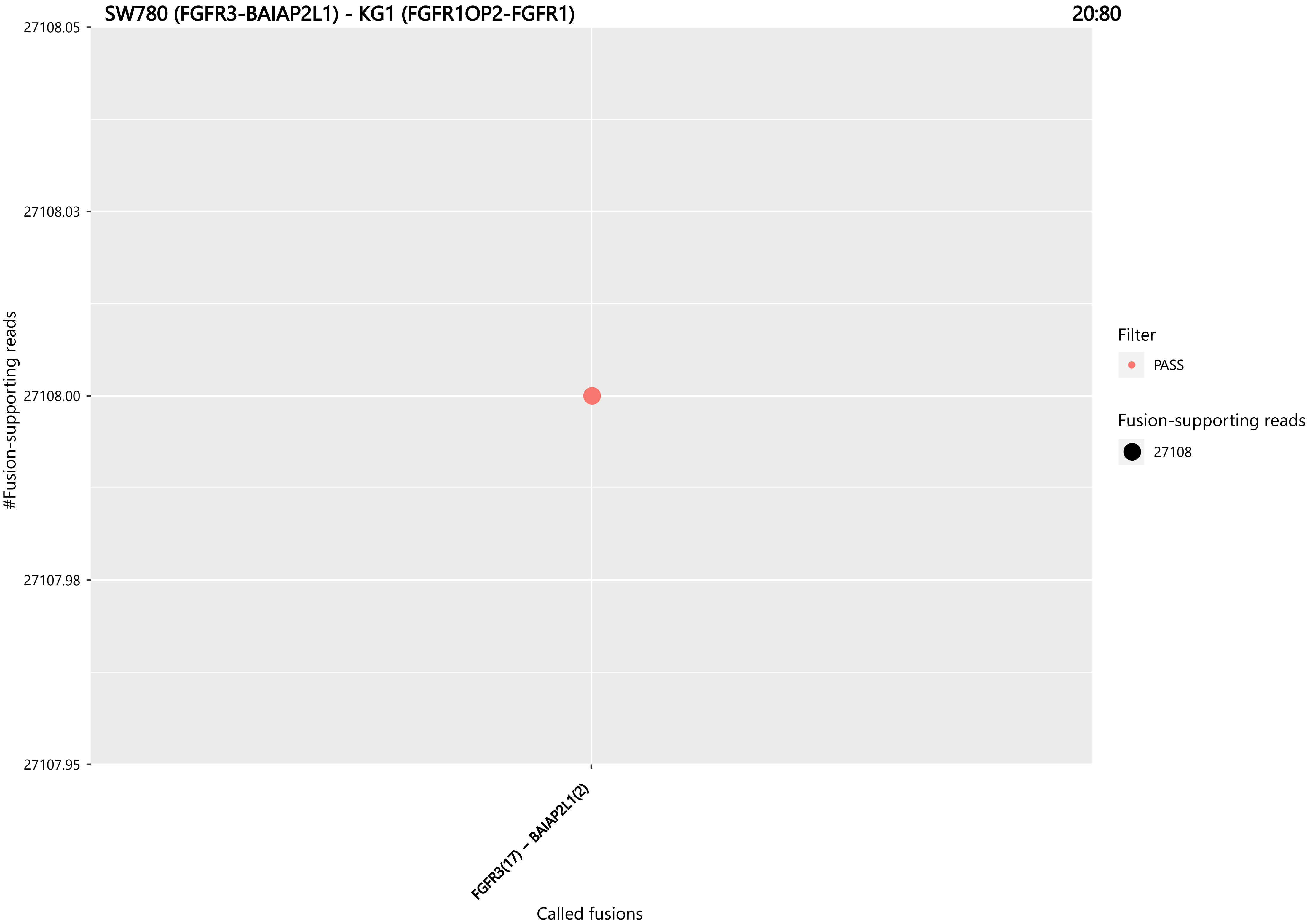

SW780 (FGFR3-BAIAP2L1) - KG1 (FGFR1OP2-FGFR1)
20:80
27108.05
27108.03
27108.00
27107.98
27107.95
#Fusion-supporting reads
FGFR3(17) – BAIAP2L1(2)
Called fusions
Filter
PASS
Fusion-supporting reads
27108

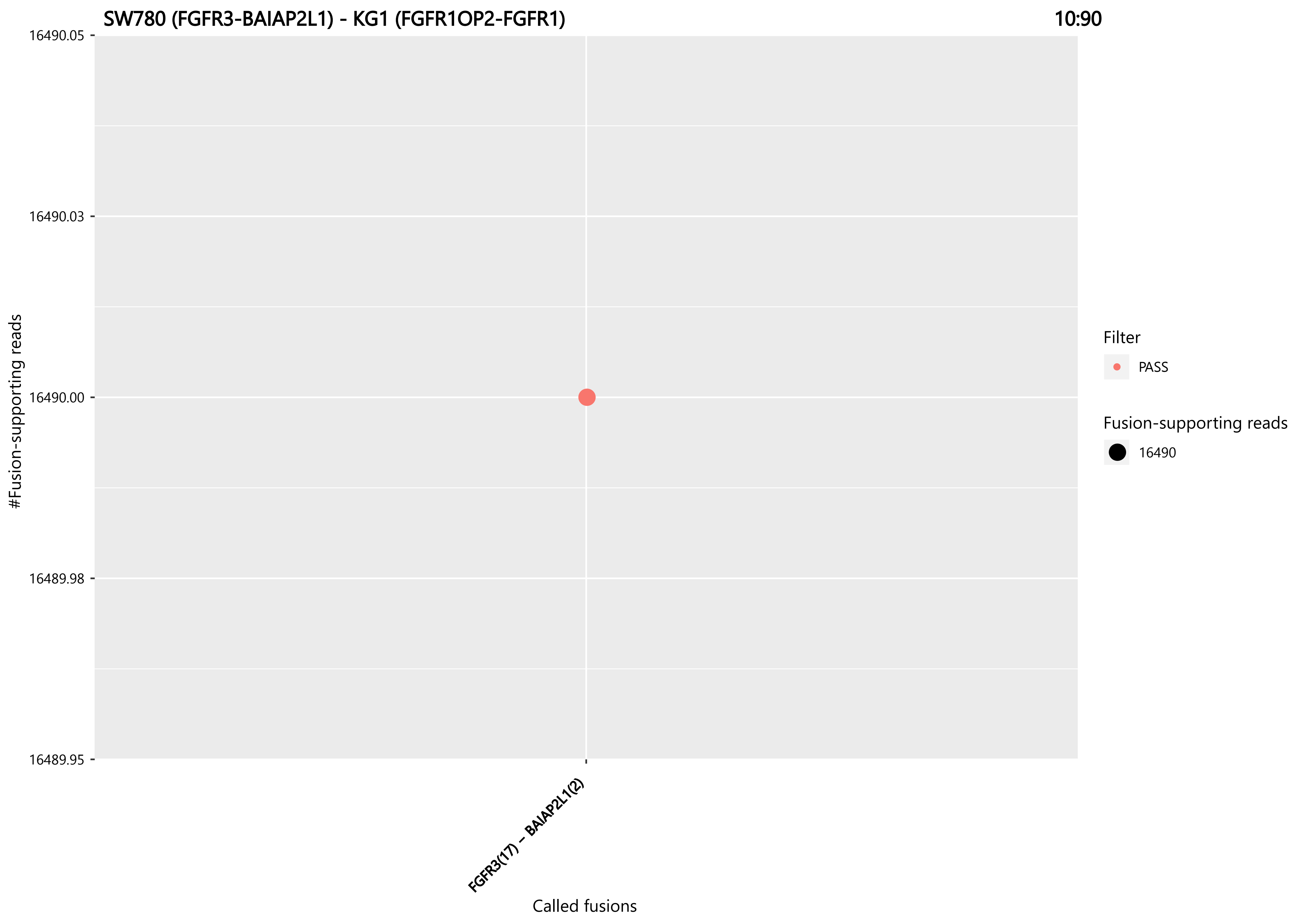

SW780 (FGFR3-BAIAP2L1) - KG1 (FGFR1OP2-FGFR1)
10:90
16490.05
16490.03
16490.00
16489.98
16489.95
#Fusion-supporting reads
FGFR3(17) – BAIAP2L1(2)
Called fusions
Filter
PASS
Fusion-supporting reads
16490

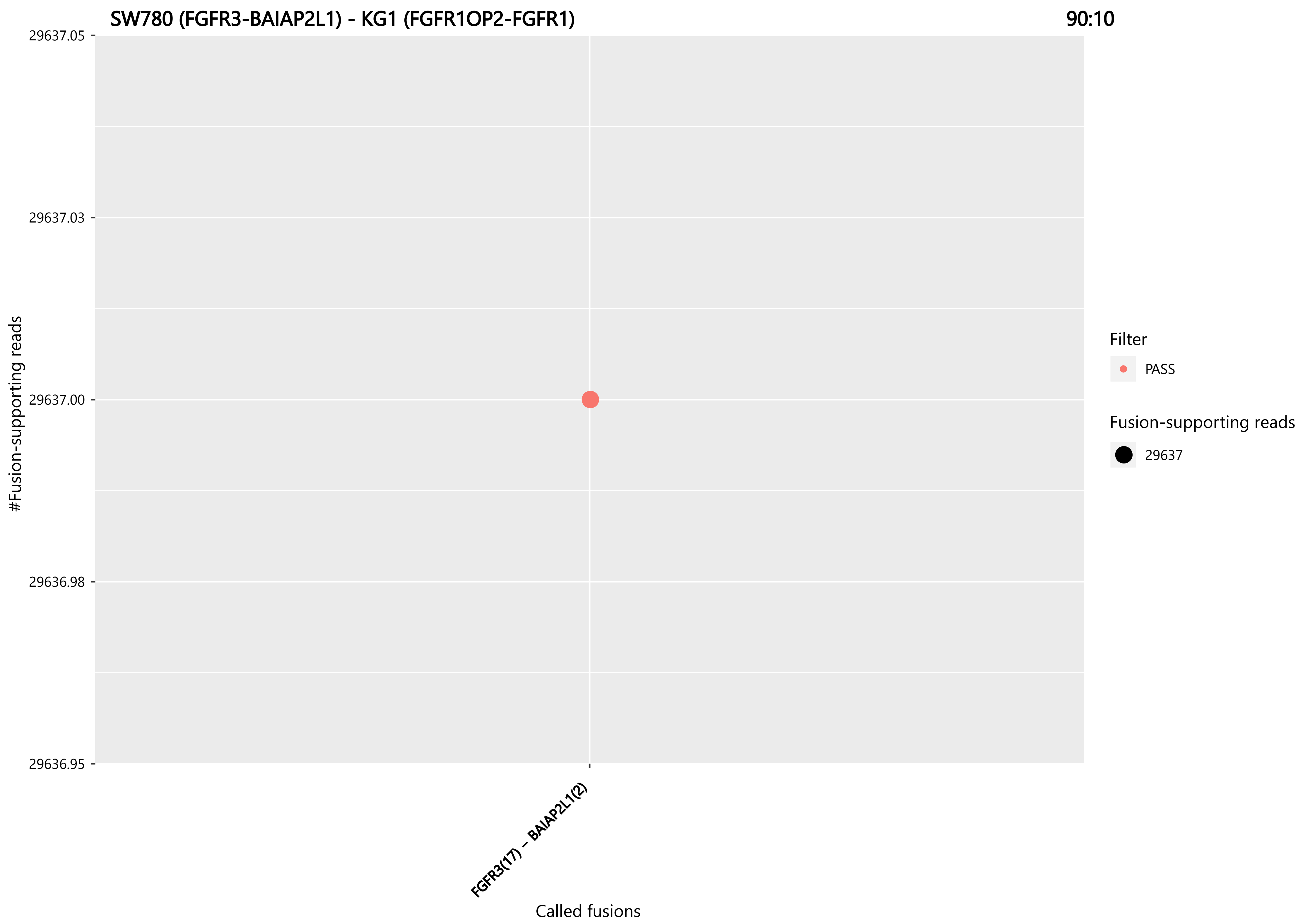
SW780 (FGFR3-BAIAP2L1) - KG1 (FGFR1OP2-FGFR1)
90:10
29637.05
29637.03
29637.00
29636.98
29636.95
#Fusion-supporting reads
FGFR3(17) – BAIAP2L1(2)
Called fusions
Filter
PASS
Fusion-supporting reads
29637

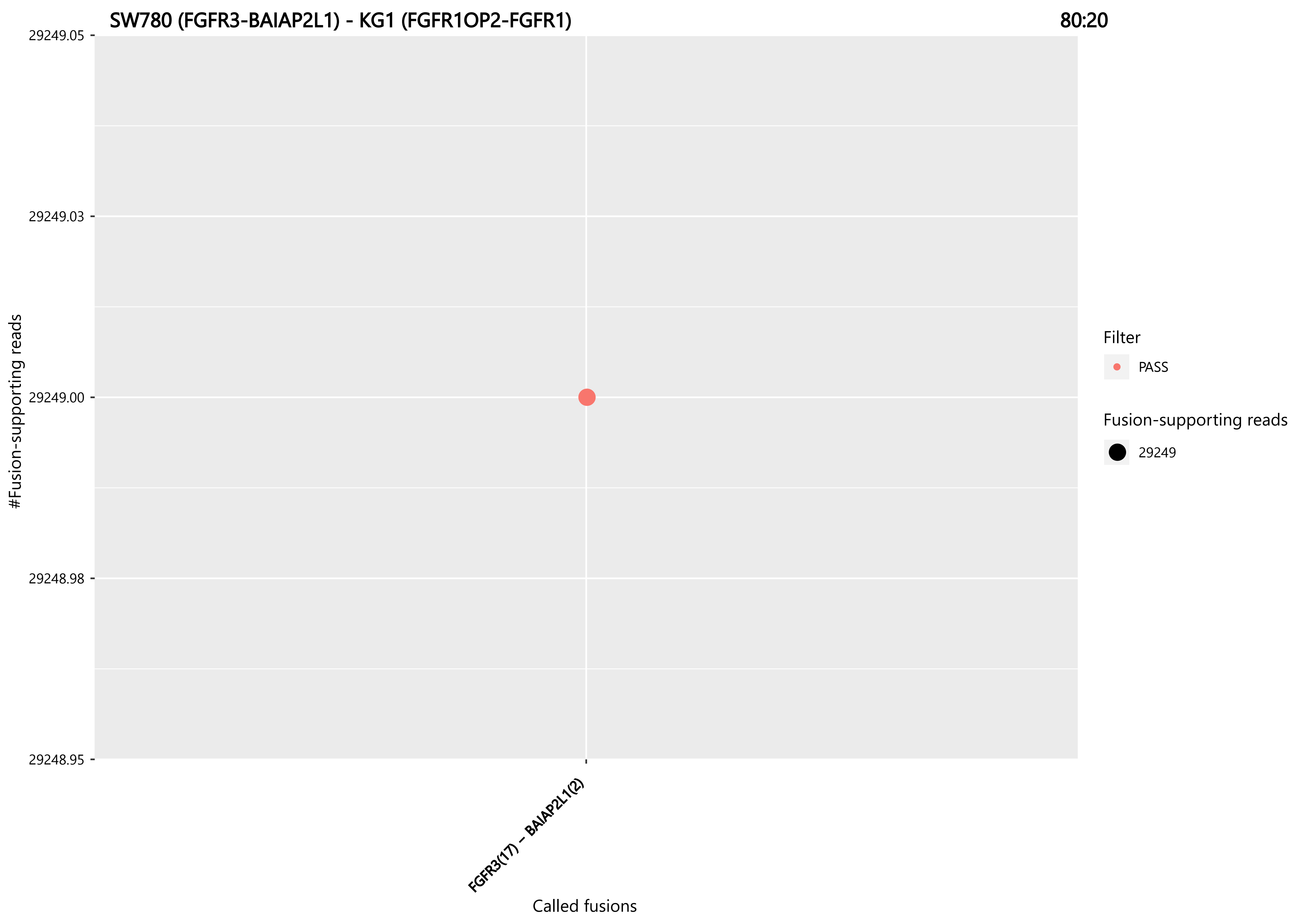

SW780 (FGFR3-BAIAP2L1) - KG1 (FGFR1OP2-FGFR1)
80:20
29249.05
29249.03
29249.00
29248.98
29248.95
#Fusion-supporting reads
FGFR3(17) – BAIAP2L1(2)
Called fusions
Filter
PASS
Fusion-supporting reads
29249

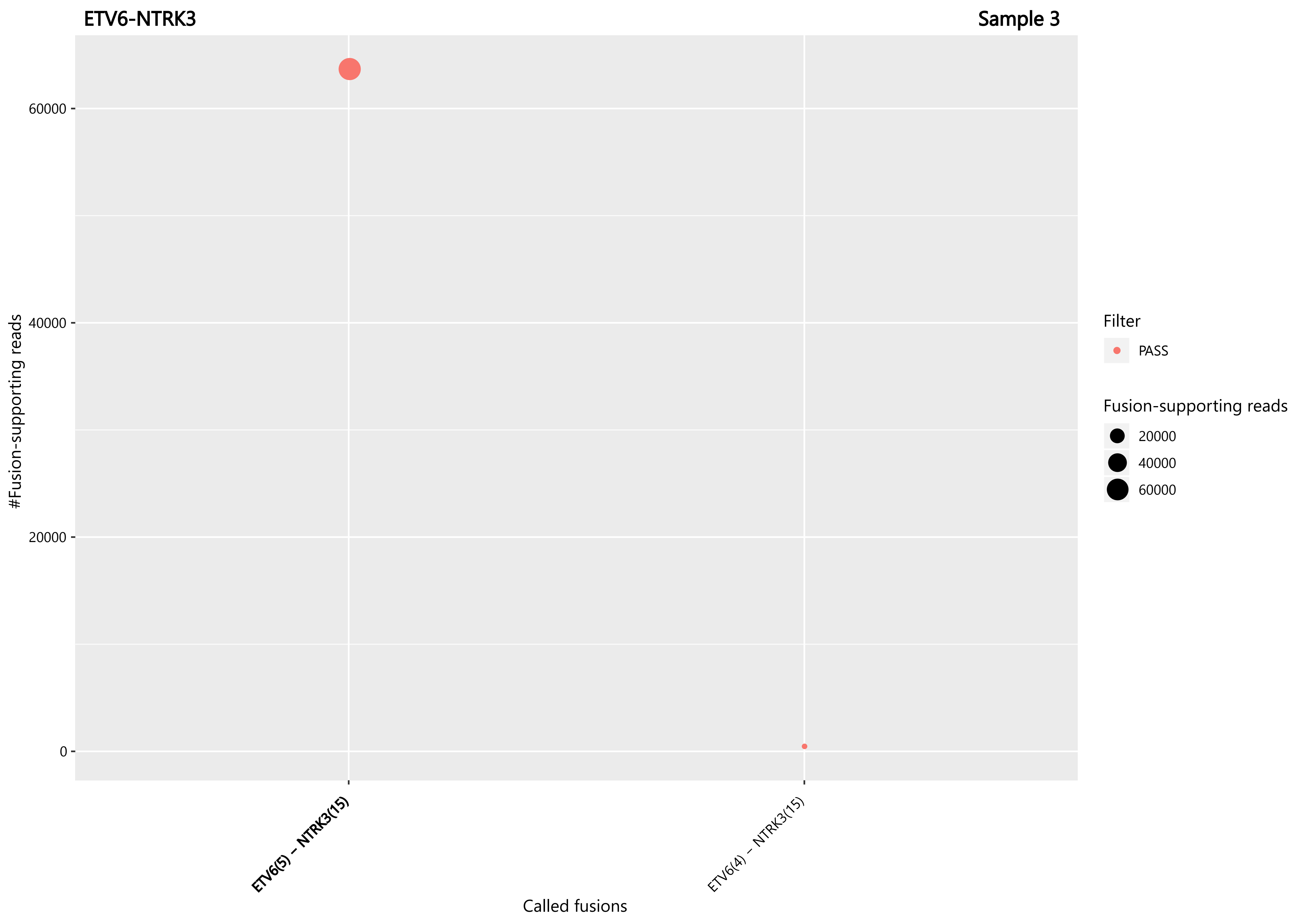

ETV6-NTRK3
Sample 3
#Fusion-supporting reads
60000
40000
20000
0
ETV6(5) – NTRK3(15)
ETV6(4) – NTRK3(15)
Called fusions
Filter
PASS
Fusion-supporting reads
20000
40000
60000

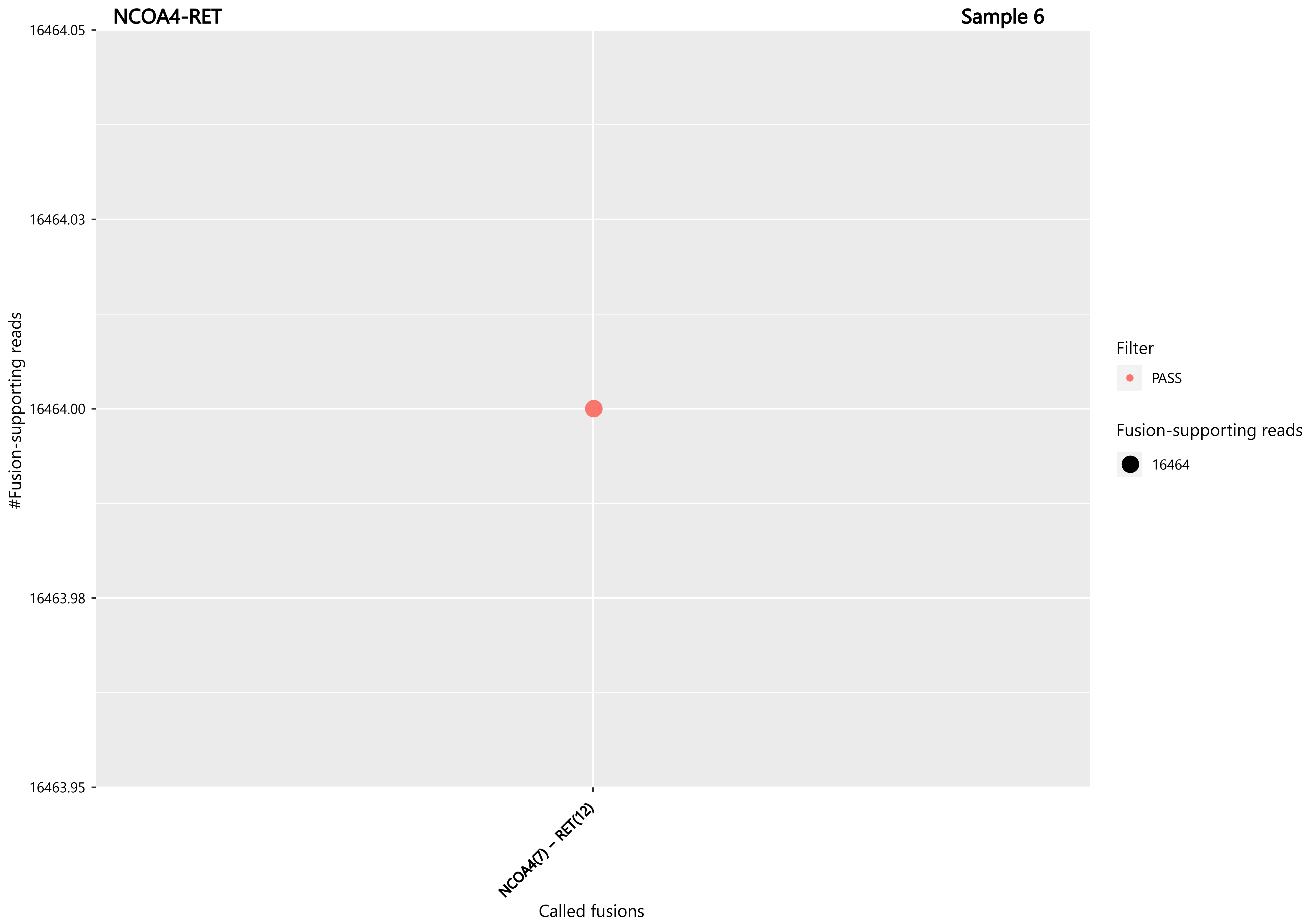

NCOA4-RET
Sample 6
#Fusion-supporting reads
16464.05
16464.03
16464.00
16463.98
16463.95
NCOA4(7) – RET(12)
Called fusions
Filter
PASS
Fusion-supporting reads
16464

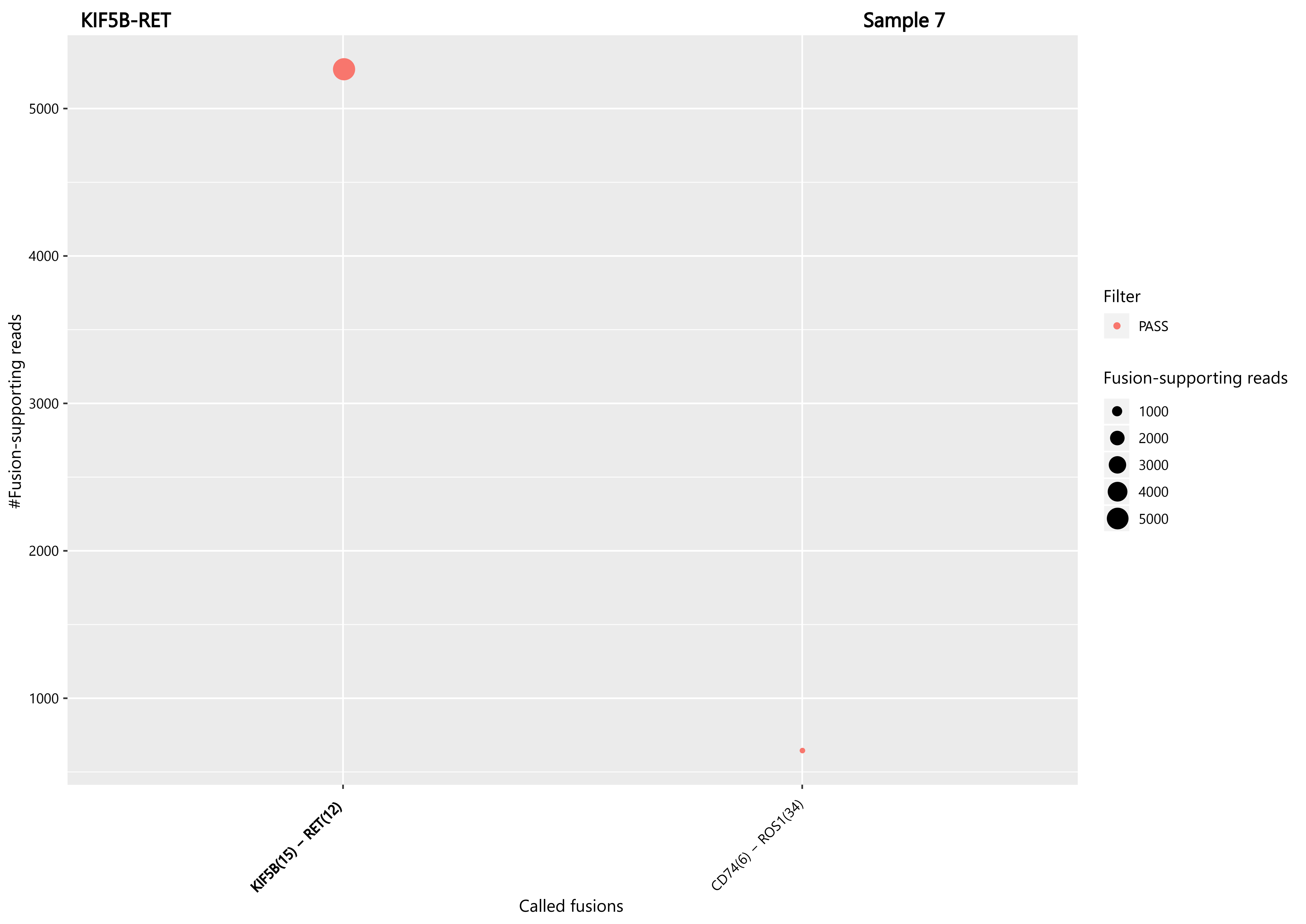
KIF5B-RET
Sample 7
#Fusion-supporting reads
5000
4000
3000
2000
1000
Called fusions
KIF5B(15) – RET(12)
CD74(6) – ROS1(34)
Filter
PASS
Fusion-supporting reads
1000
2000
3000
4000
5000

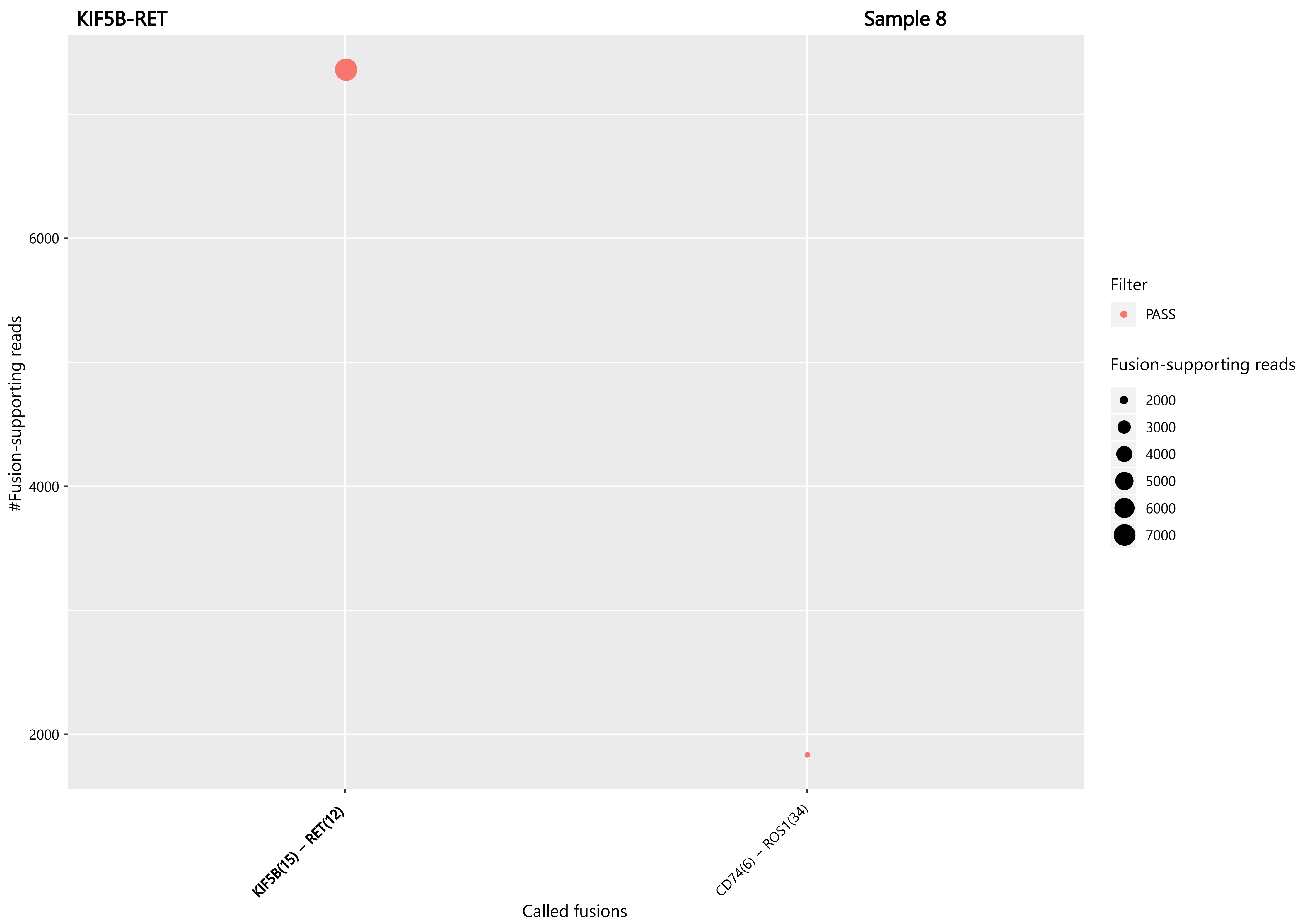
KIF5B-RET
Sample 8
#Fusion-supporting reads
6000
4000
2000
Called fusions
KIF5B(15) – RET(12)
CD74(6) – ROS1(34)
Filter
PASS
Fusion-supporting reads
2000
3000
4000
5000
6000
7000

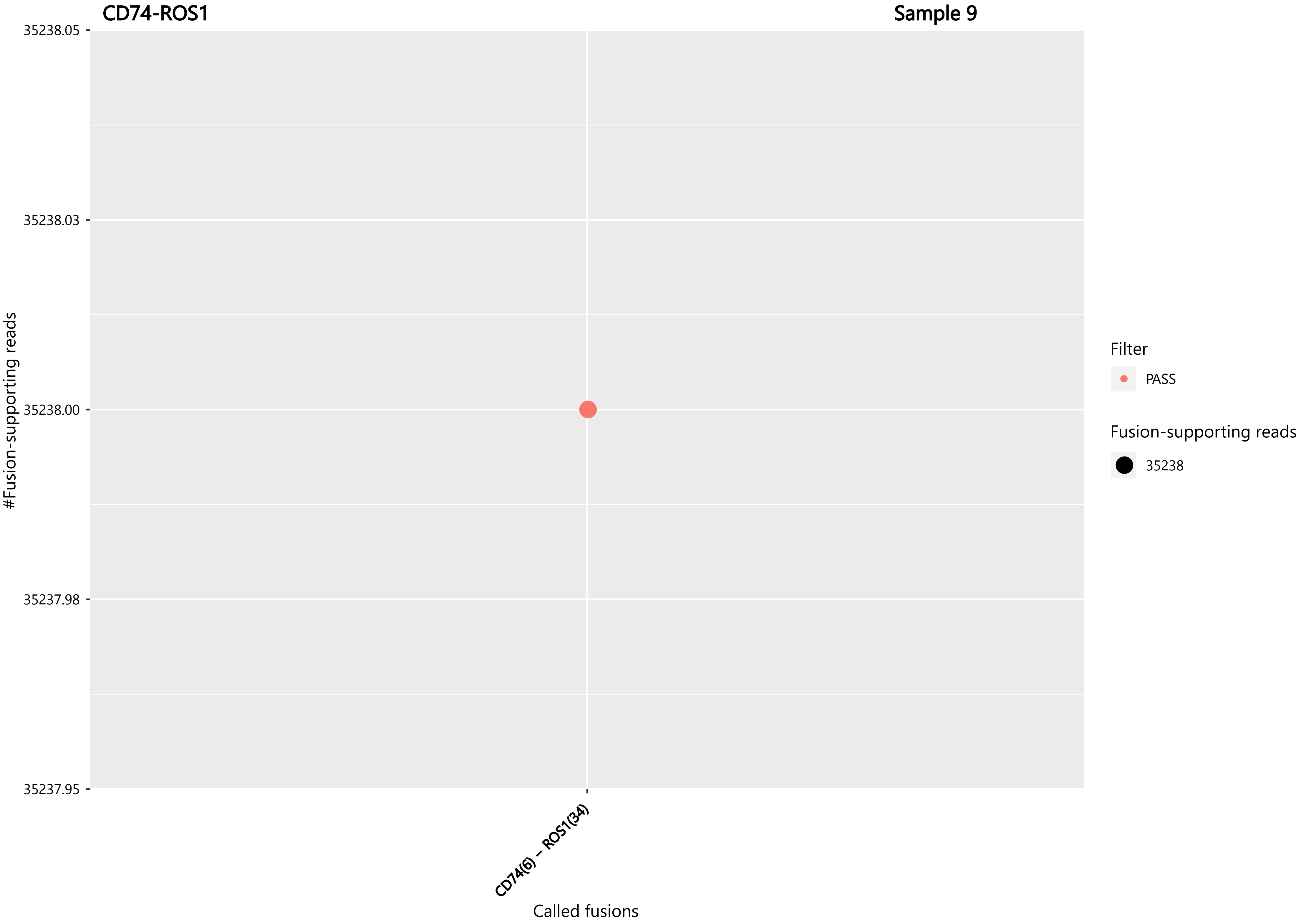

CD74-ROS1
Sample 9
35238.05
35238.03
35238.00
35237.98
35237.95
#Fusion-supporting reads
CD74(6) – ROS1(34)
Called fusions
Filter
PASS
Fusion-supporting reads
35238

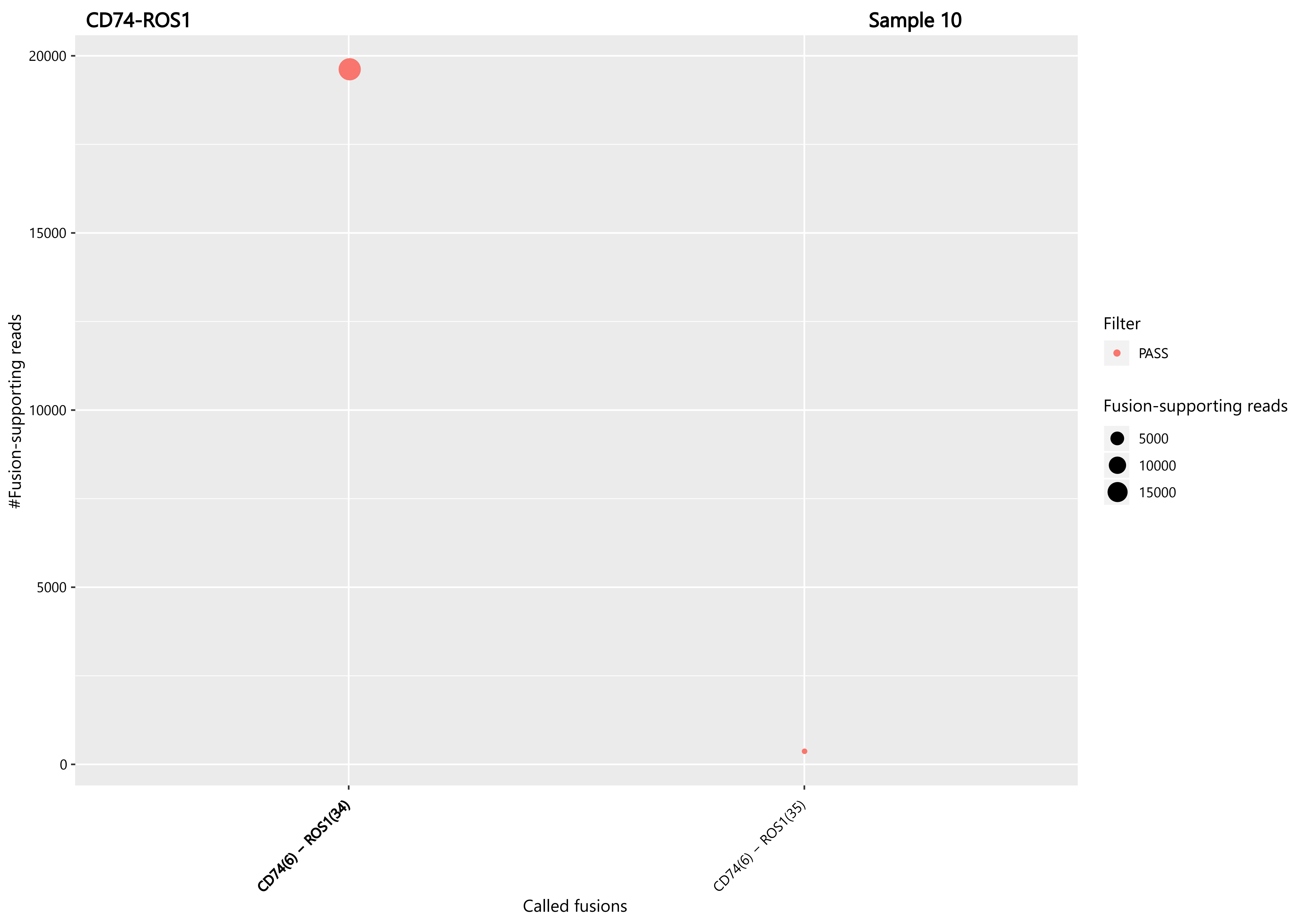
CD74-ROS1
Sample 10
#Fusion-supporting reads
20000
15000
10000
5000
0
CD74(6) – ROS1(34)
CD74(6) – ROS1(35)
Called fusions
Filter
PASS
Fusion-supporting reads
5000
10000
15000

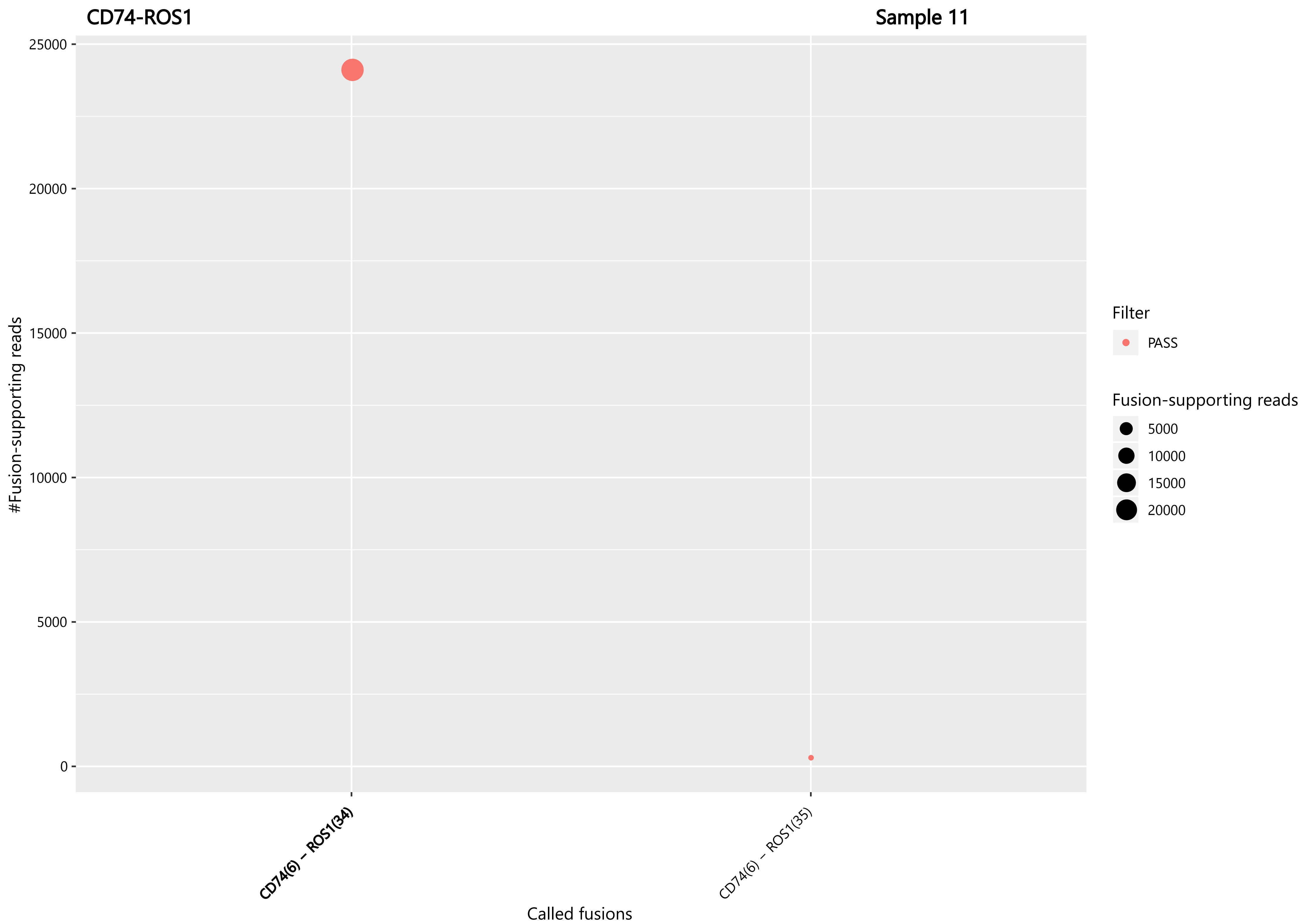

CD74-ROS1
Sample 11
#Fusion-supporting reads
25000
20000
15000
10000
5000
0
Called fusions
CD74(6) – ROS1(34)
CD74(6) – ROS1(35)
Filter
PASS
Fusion-supporting reads
5000
10000
15000
20000

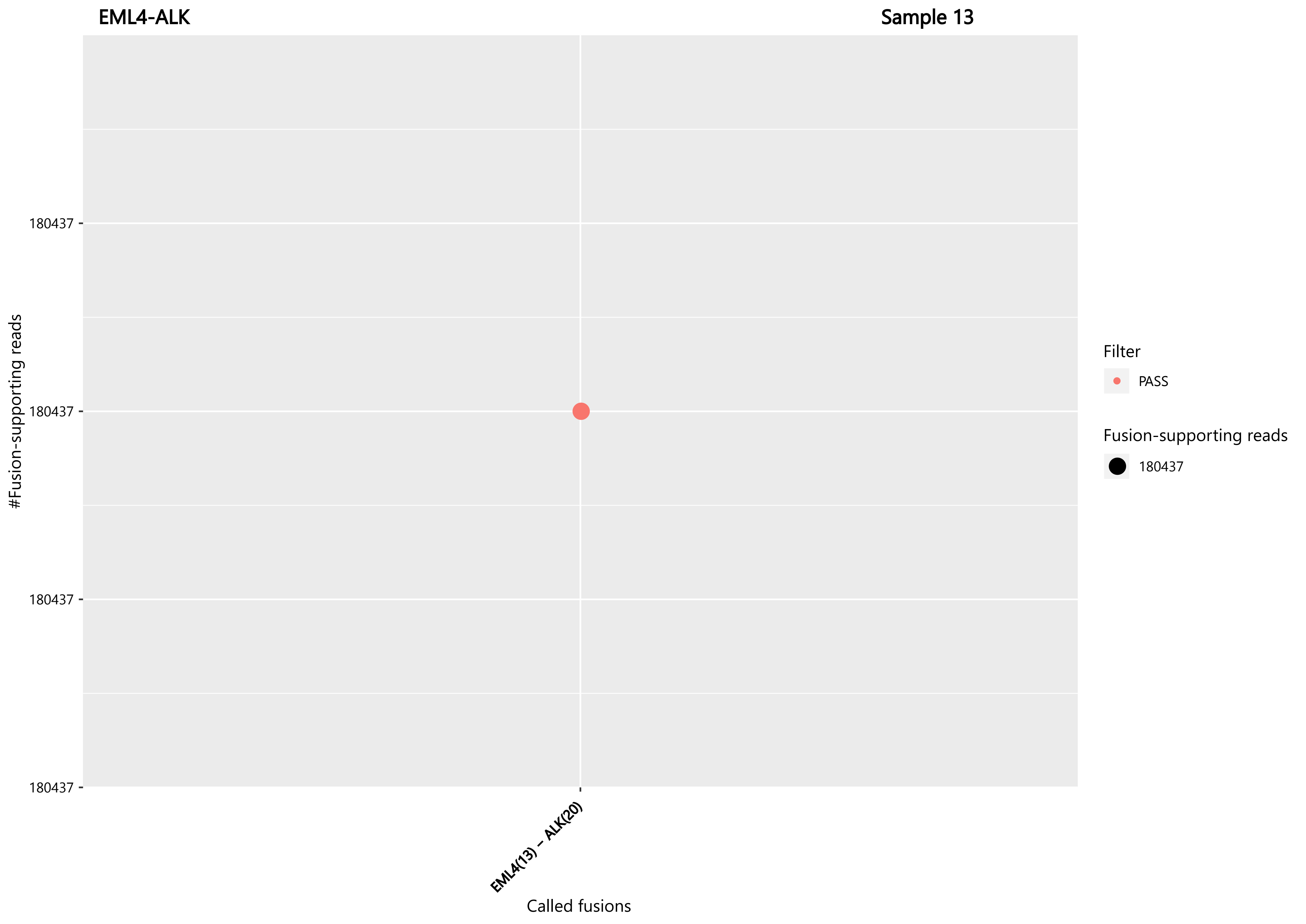
EML4-ALK
Sample 13
180437
180437
180437
180437
#Fusion-supporting reads
EML4(13) – ALK(20)
Called fusions
Filter
PASS
Fusion-supporting reads
180437

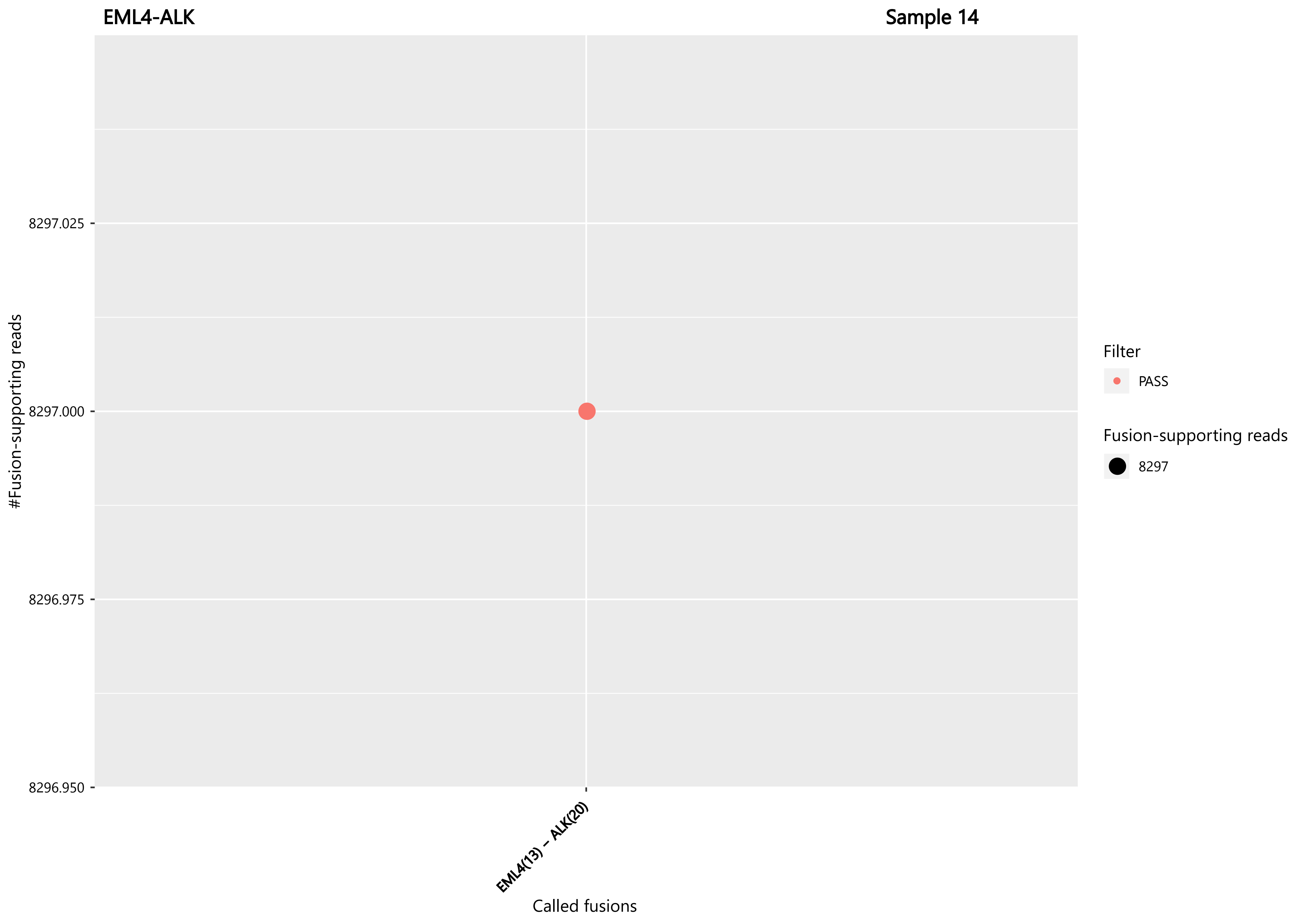

EML4-ALK
Sample 14
#Fusion-supporting reads
8297.025
8297.000
8296.975
8296.950
EML4(13) – ALK(20)
Called fusions
Filter
PASS
Fusion-supporting reads
8297

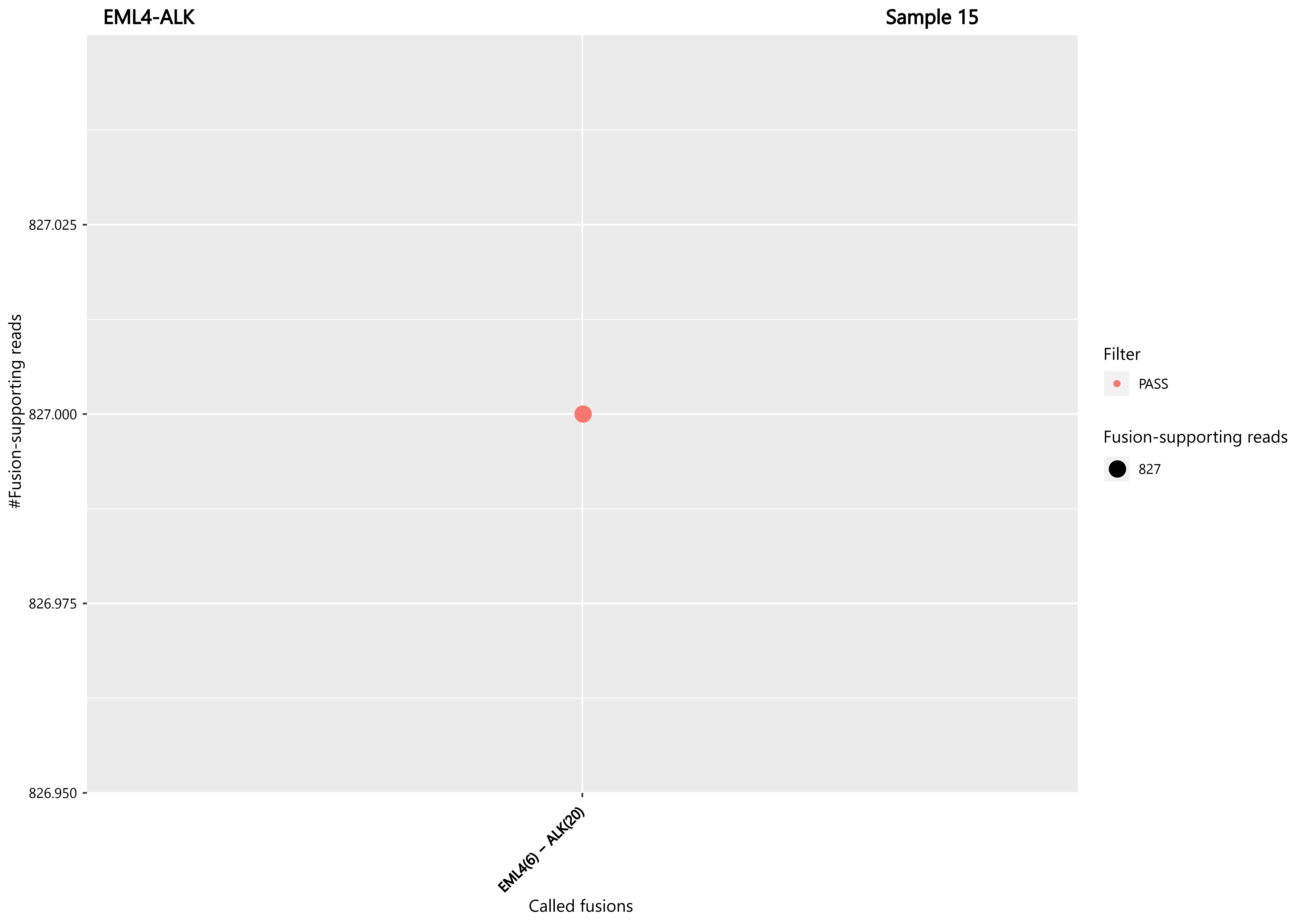

EML4-ALK
Sample 15
#Fusion-supporting reads
827.025
827.000
826.975
826.950
EML4(6) – ALK(20)
Called fusions
Filter
PASS
Fusion-supporting reads
827
