# Supplementary material for: Detection of gene fusions using targeted next-generation sequencing: a comparative evaluation
Source: BMC Med Genomics. 2021 Feb 27;14:62. doi: 10.1186/s12920-021-00909-y (PMC7912891; doi:10.1186/s12920-021-00909-y)
Supplement: Supplementary file 14 — Additional file 14: Fig. S14. Fusions detected with the TruSight Tumor 170 Assay (Illumina) for all samples. Metrics such as quality control scores, in-frame status or filter thresholds were plotted when available. In cases where the same fusion was identified more than once within the same sample, a unique numbering scheme was added at the end of the name to differentiate the candidate fusions. The numbering however, does not imply any special order or preference over the other fusions with the same name. The putative detected fusions were arranged in decreasing order based on the number of fusion-supporting reads. The expected fusion for each sample was highlighted in bold. [file 12920_2021_909_MOESM14_ESM.pdf]

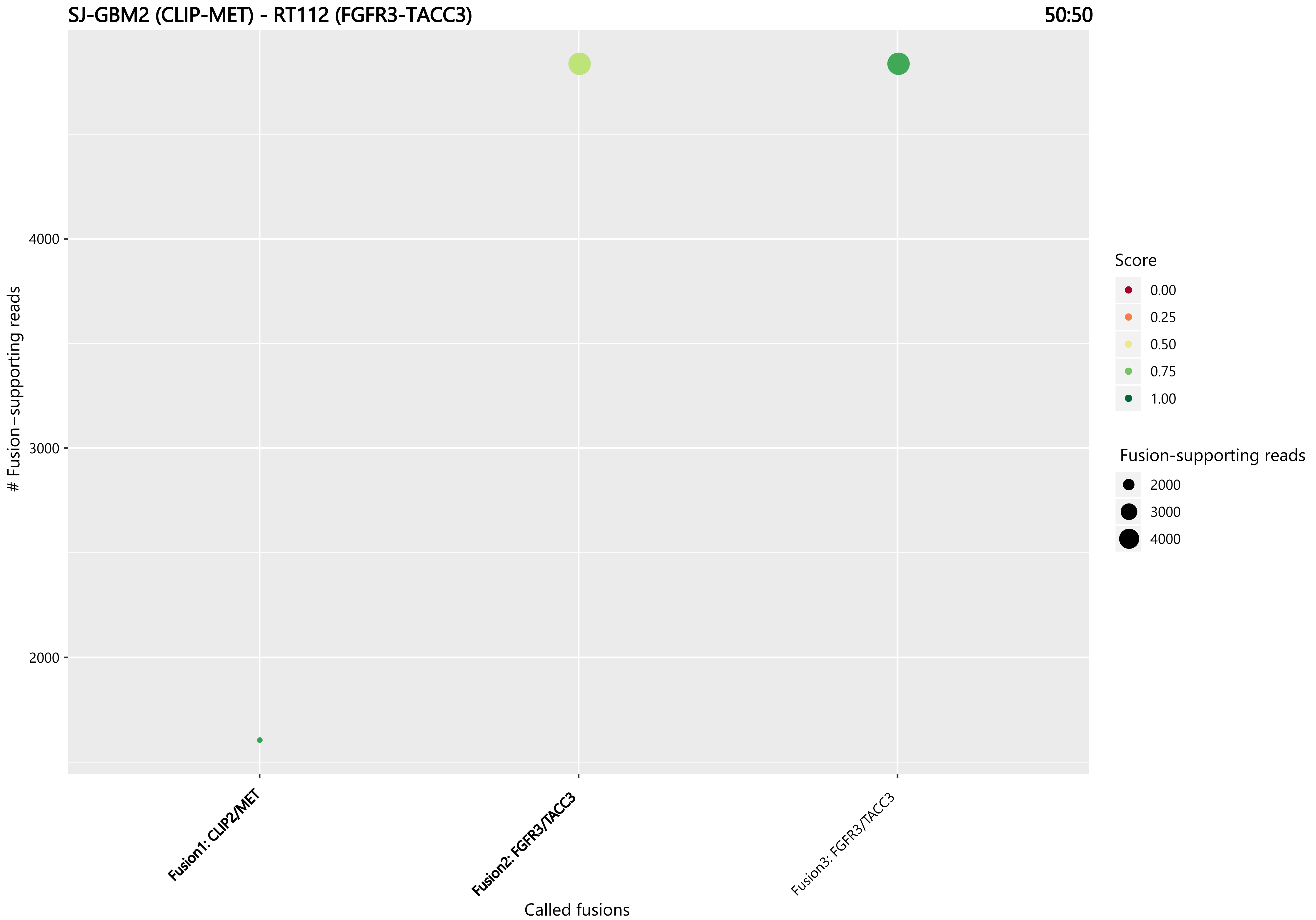

SJ-GBM2 (CLIP-MET) - RT112 (FGFR3-TACC3)
50:50
Fusion−supporting reads
4000
3000
2000
Called fusions
Fusion1: CLIP2/MET
Fusion2: FGFR3/TACC3
Fusion3: FGFR3/TACC3
Score
0.00
0.25
0.50
0.75
1.00
Fusion-supporting reads
2000
3000
4000

SJ-GBM2 (CLIP-MET) - RT112 (FGFR3-TACC3) **20:80**

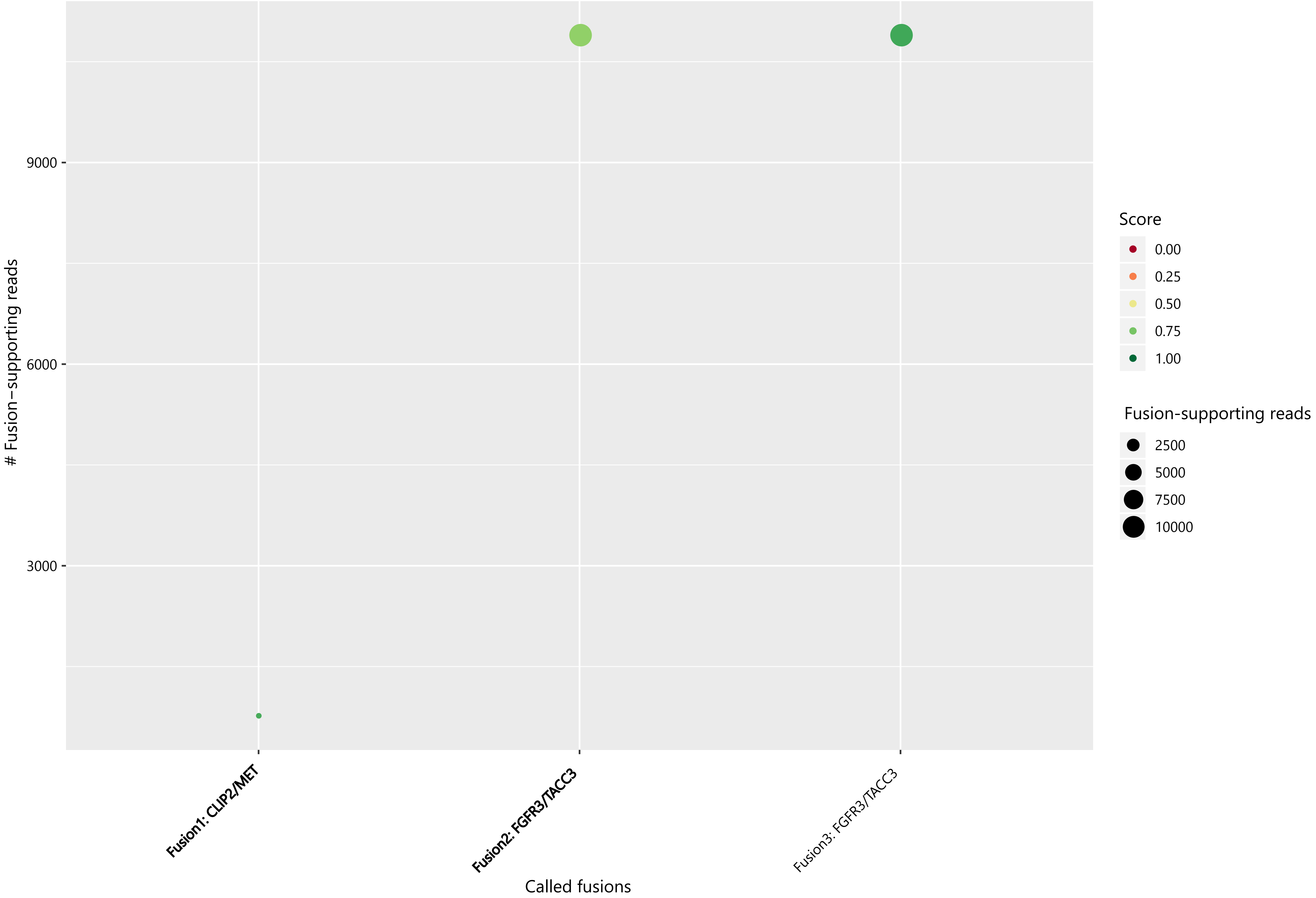

SJ-GBM2 (CLIP-MET) - RT112 (FGFR3-TACC3)

10:90

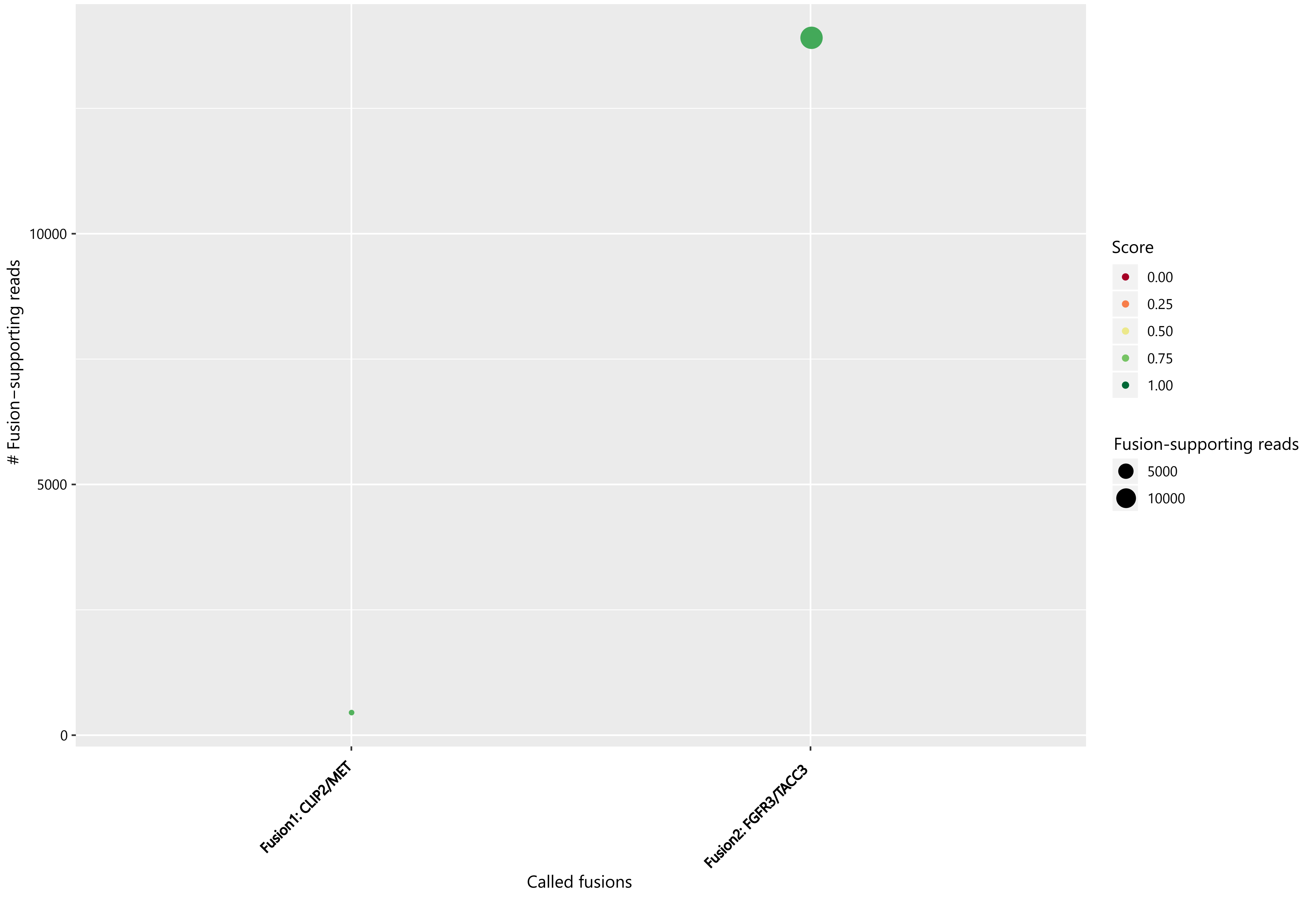

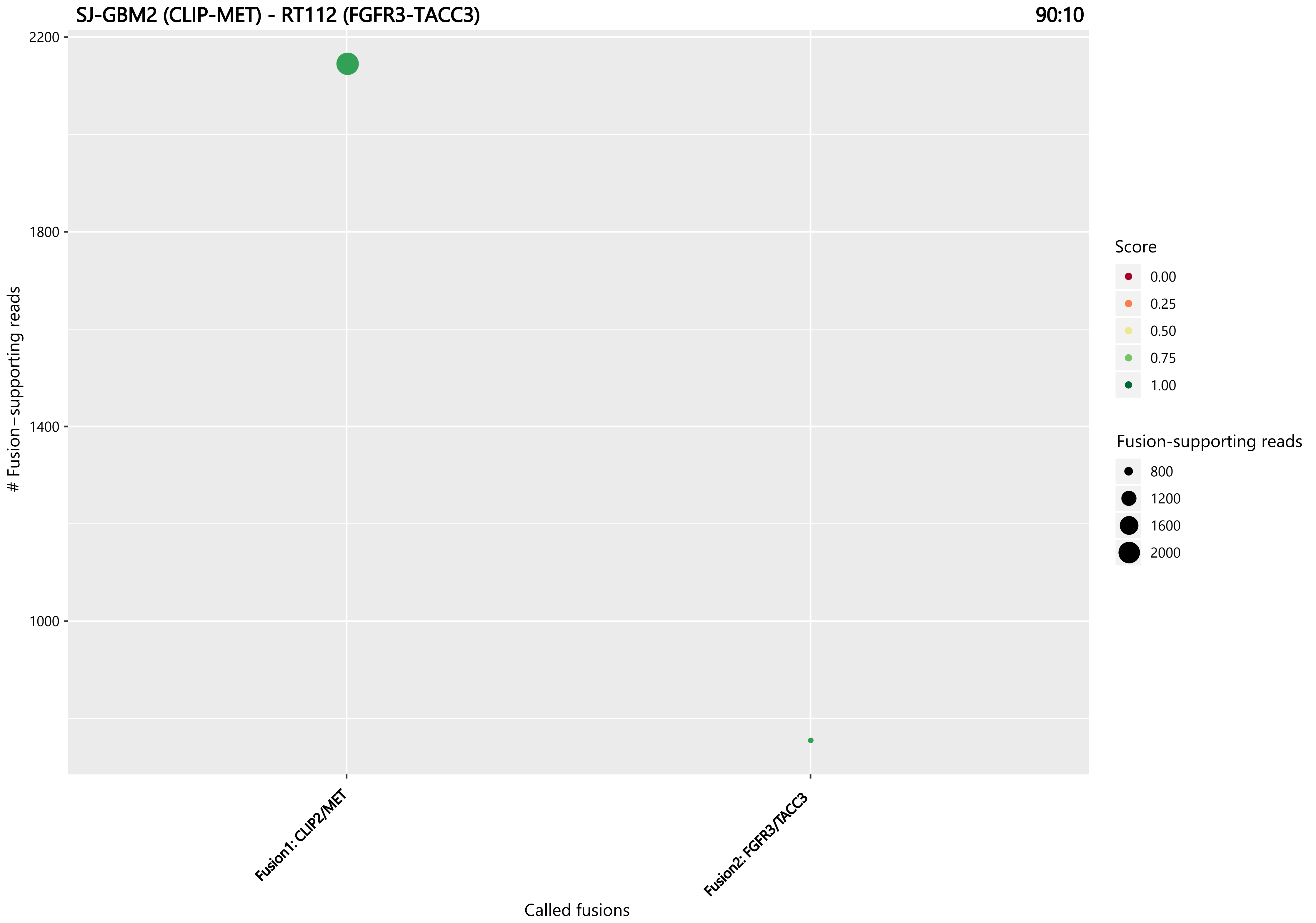
SJ-GBM2 (CLIP-MET) - RT112 (FGFR3-TACC3)
90:10
2200
1800
1400
1000
Fusion−supporting reads
Fusion1: CLIP2/MET
Fusion2: FGFR3/TACC3
Called fusions
Score
0.00
0.25
0.50
0.75
1.00
Fusion−supporting reads
800
1200
1600
2000

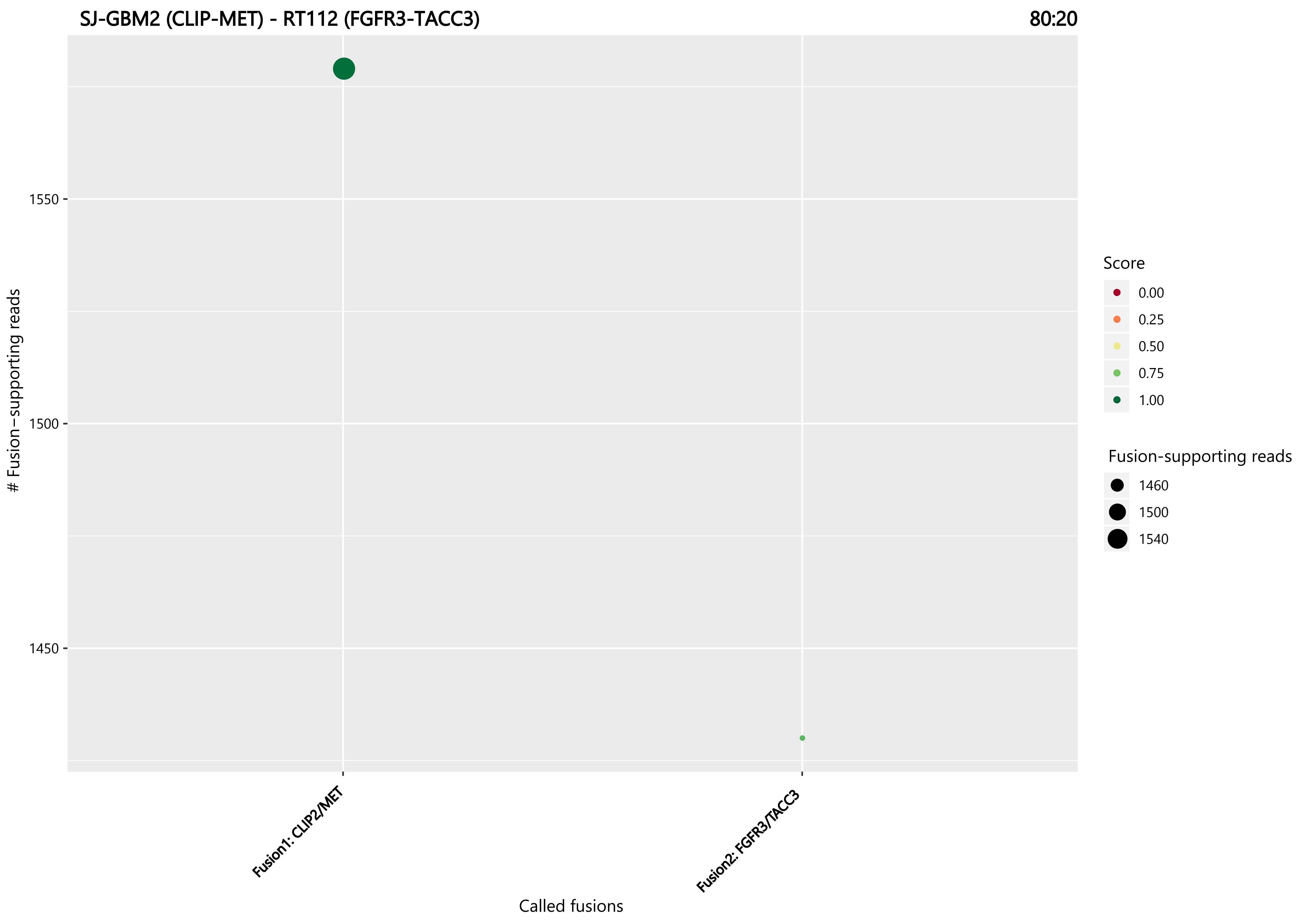

SJ-GBM2 (CLIP-MET) - RT112 (FGFR3-TACC3)
80:20
1550
1500
1450
Fusion−supporting reads
Fusion1: CLIP2/MET
Fusion2: FGFR3/TACC3
Called fusions
Score
0.00
0.25
0.50
0.75
1.00
Fusion-supporting reads
1460
1500
1540

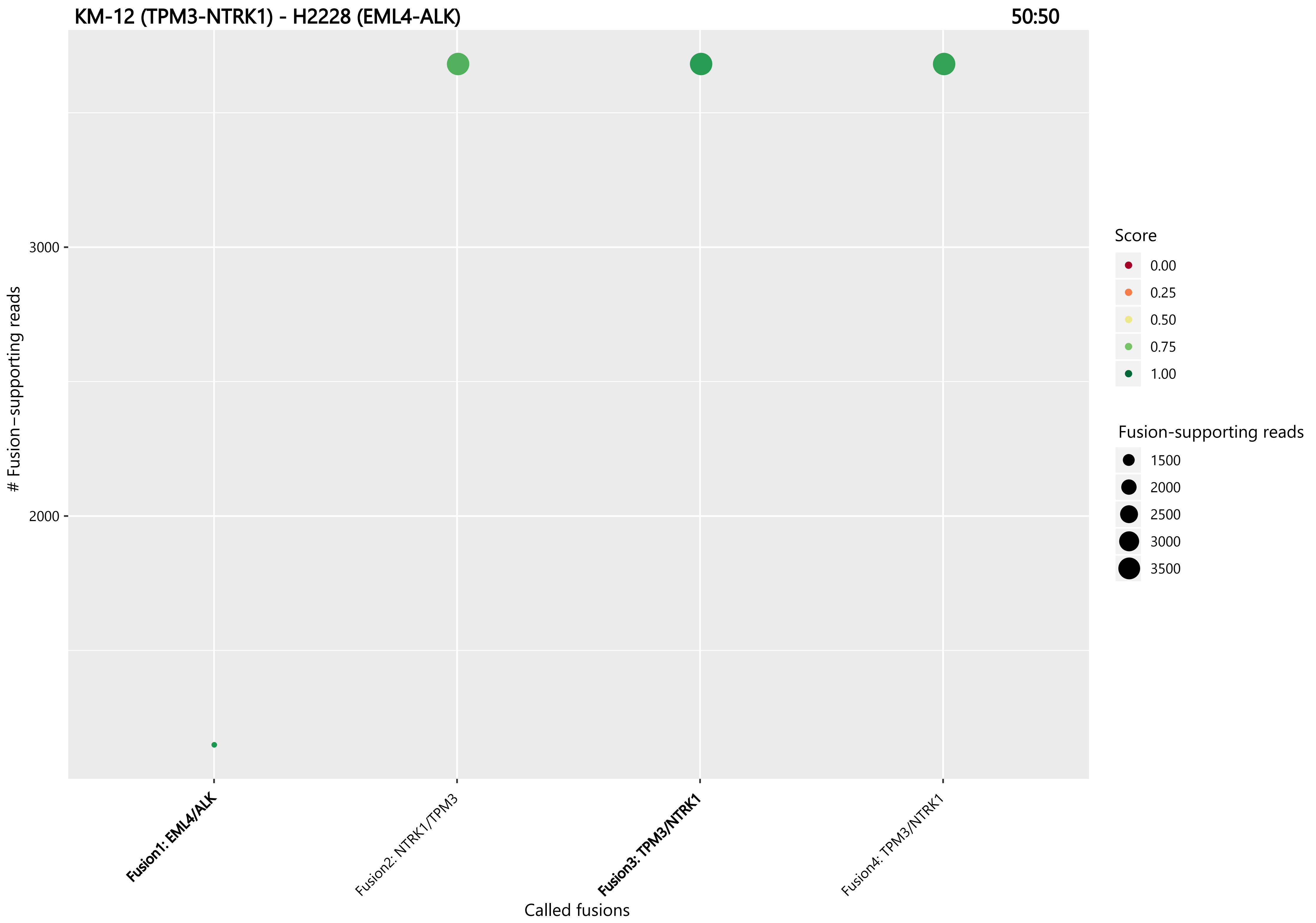
KM-12 (TPM3-NTRK1) - H2228 (EML4-ALK)
50:50
Fusion−supporting reads
3000
2000
Called fusions
Fusion1: EML4/ALK
Fusion2: NTRK1/TPM3
Fusion3: TPM3/NTRK1
Fusion4: TPM3/NTRK1
Score
0.00
0.25
0.50
0.75
1.00
Fusion-supporting reads
1500
2000
2500
3000
3500

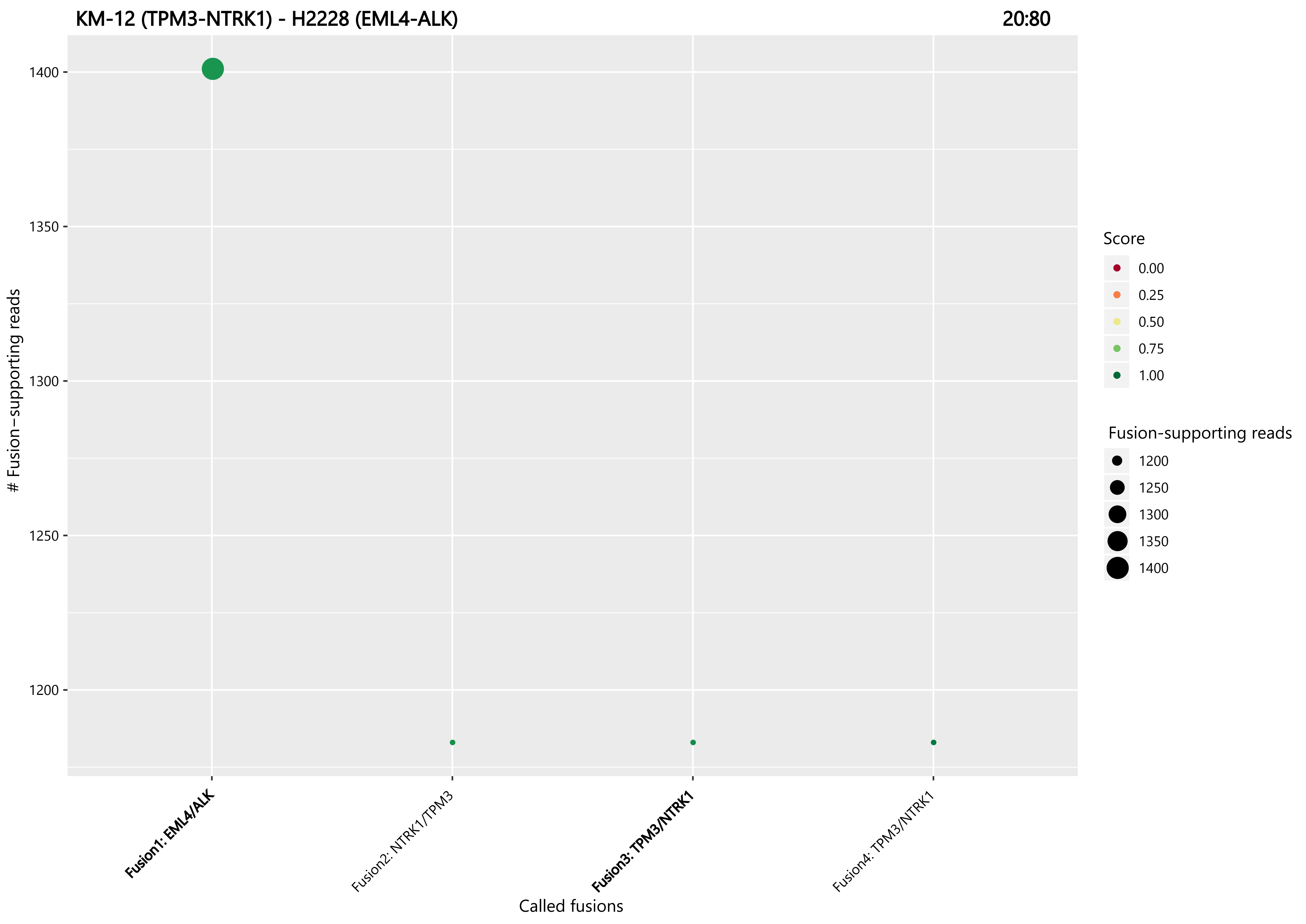
KM-12 (TPM3-NTRK1) - H2228 (EML4-ALK)
20:80
Fusion−supporting reads
1400
1350
1300
1250
1200
Score
0.00
0.25
0.50
0.75
1.00
Fusion-supporting reads
1200
1250
1300
1350
1400
Fusion1: EML4/ALK
Fusion2: NTRK1/TPM3
Fusion3: TPM3/NTRK1
Fusion4: TPM3/NTRK1
Called fusions

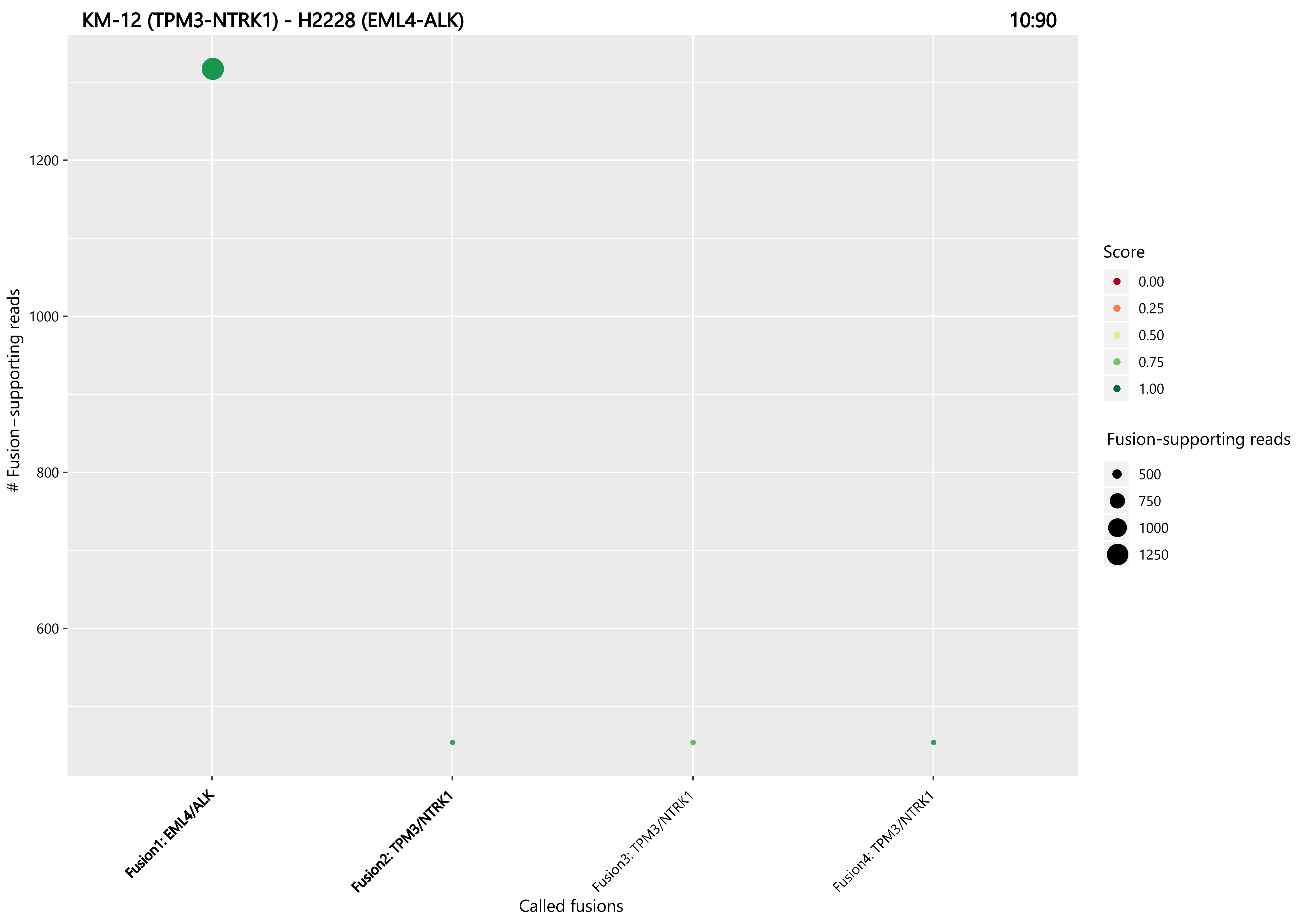

KM-12 (TPM3-NTRK1) - H2228 (EML4-ALK)
10:90
Fusion−supporting reads
1200
1000
800
600
Called fusions
Fusion1: EML4/ALK
Fusion2: TPM3/NTRK1
Fusion3: TPM3/NTRK1
Fusion4: TPM3/NTRK1
Score
0.00
0.25
0.50
0.75
1.00
Fusion-supporting reads
500
750
1000
1250

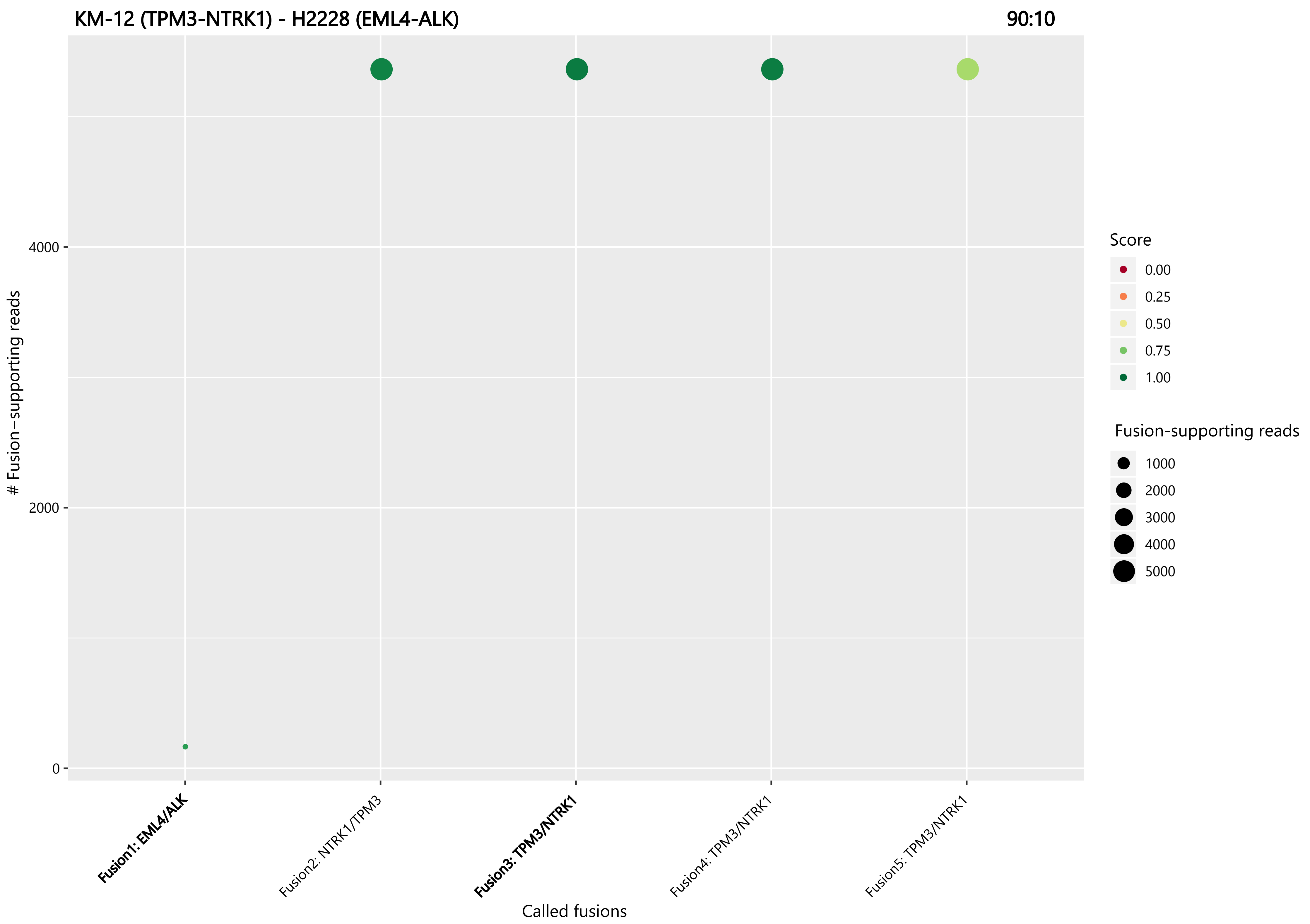

KM-12 (TPM3-NTRK1) - H2228 (EML4-ALK)
90:10
Fusion−supporting reads
4000
2000
0
Score
0.00
0.25
0.50
0.75
1.00
Fusion-supporting reads
1000
2000
3000
4000
5000
Fusion1: EML4/ALK
Fusion2: NTRK1/TPM3
Fusion3: TPM3/NTRK1
Fusion4: TPM3/NTRK1
Fusion5: TPM3/NTRK1
Called fusions

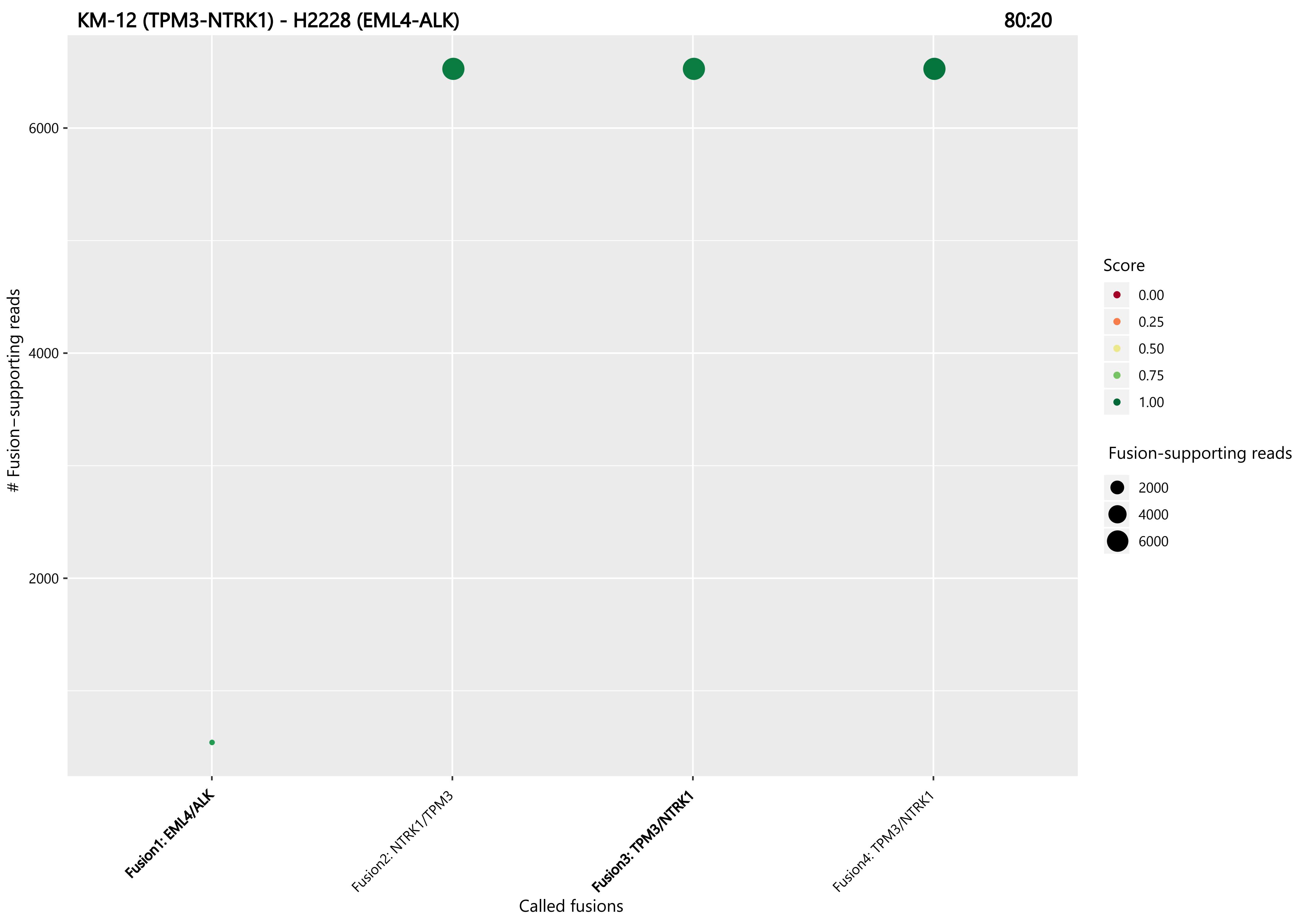
KM-12 (TPM3-NTRK1) - H2228 (EML4-ALK)
80:20
Fusion−supporting reads
6000
4000
2000
Fusion1: EML4/ALK
Fusion2: NTRK1/TPM3
Fusion3: TPM3/NTRK1
Fusion4: TPM3/NTRK1
Called fusions
Score
0.00
0.25
0.50
0.75
1.00
Fusion-supporting reads
2000
4000
6000

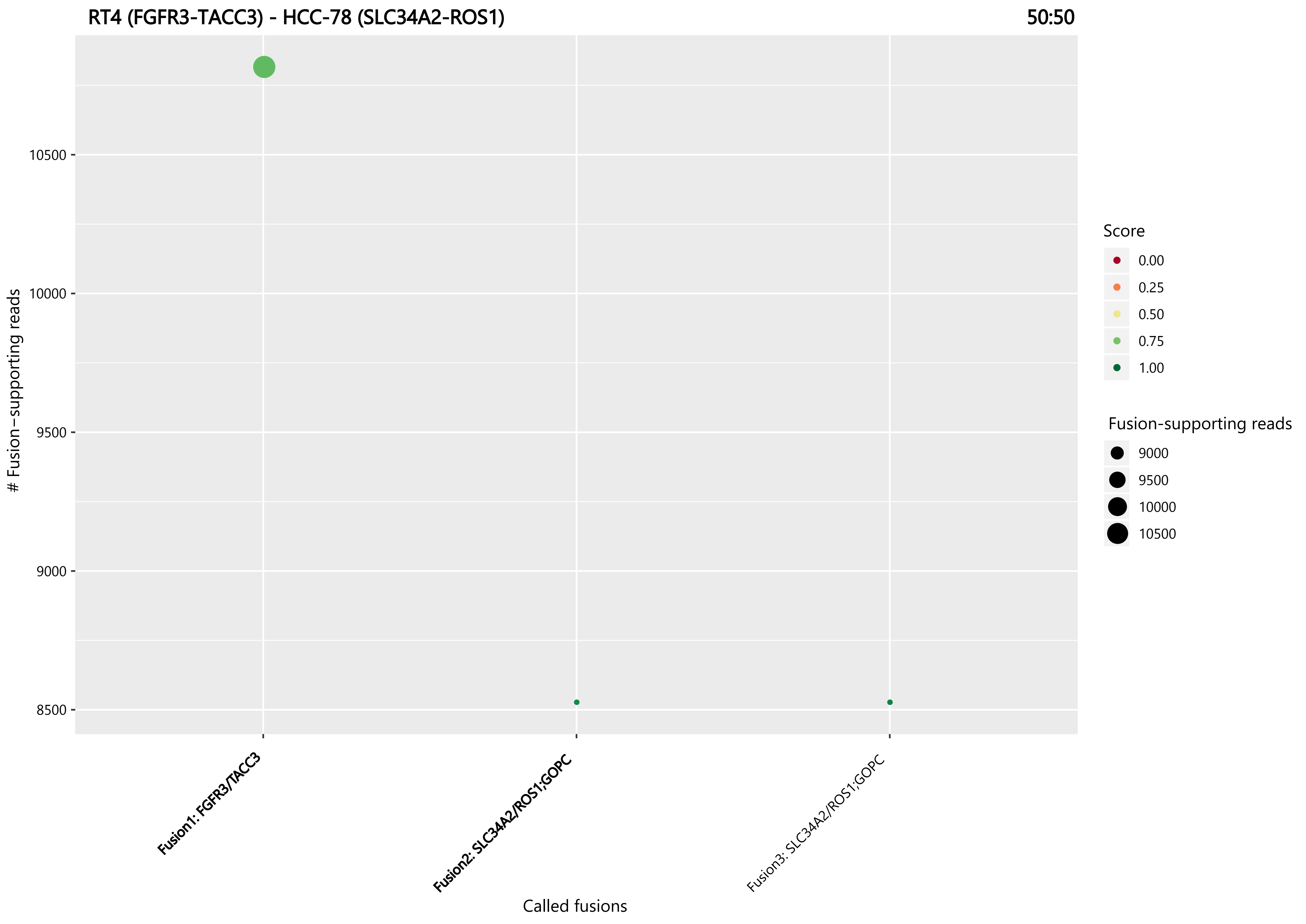
RT4 (FGFR3-TACC3) - HCC-78 (SLC34A2-ROS1)
50:50
Fusion−supporting reads
10500
10000
9500
9000
8500
Called fusions
Fusion1: FGFR3/TACC3
Fusion2: SLC34A2/ROS1;GOPC
Fusion3: SLC34A2/ROS1;GOPC
Score
0.00
0.25
0.50
0.75
1.00
Fusion-supporting reads
9000
9500
10000
10500

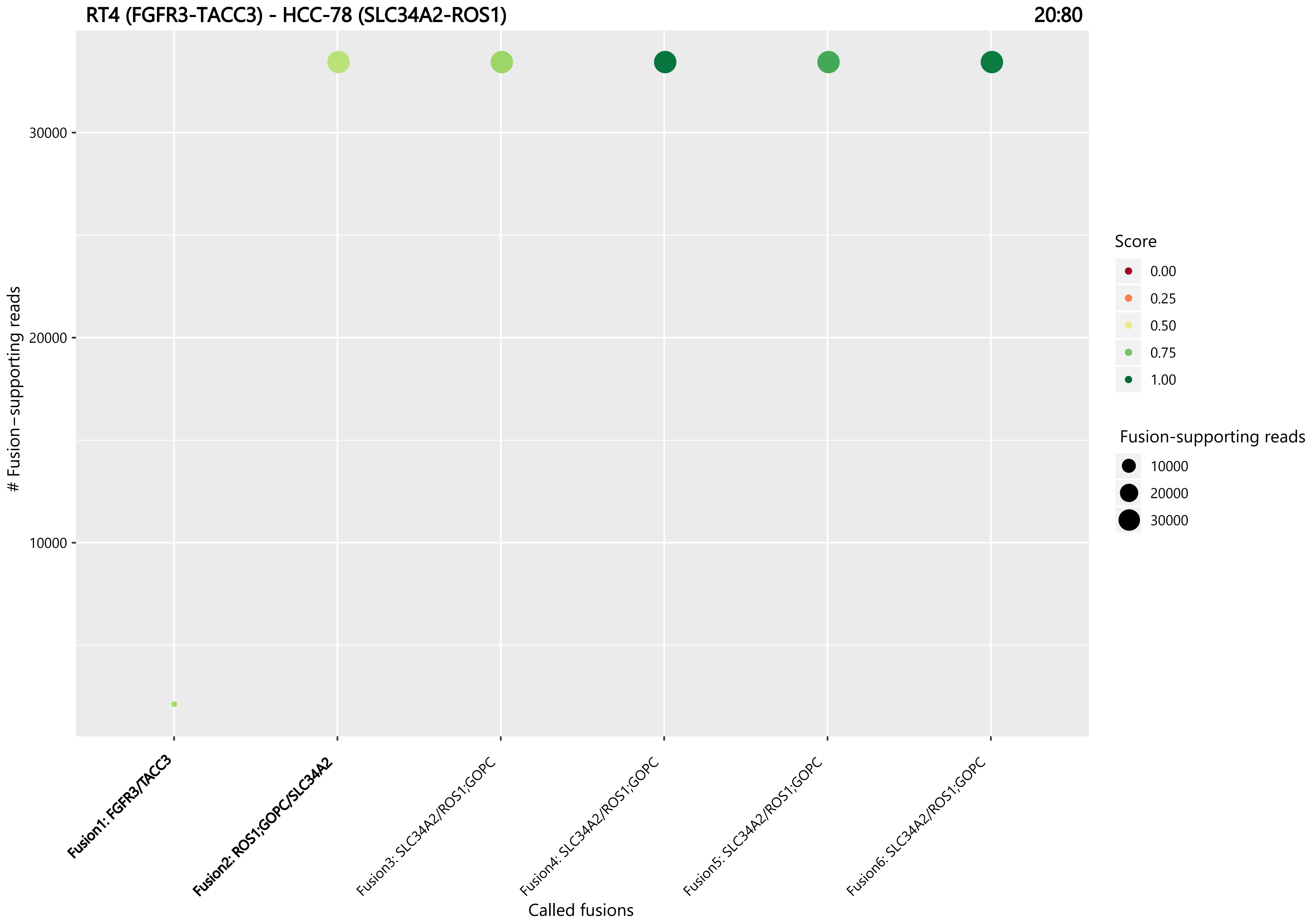
RT4 (FGFR3-TACC3) - HCC-78 (SLC34A2-ROS1)
20:80
Fusion−supporting reads
30000
20000
10000
Fusion1: FGFR3/TACC3
Fusion2: ROS1;GOPC/SLC34A2
Fusion3: SLC34A2/ROS1;GOPC
Fusion4: SLC34A2/ROS1;GOPC
Fusion5: SLC34A2/ROS1;GOPC
Fusion6: SLC34A2/ROS1;GOPC
Called fusions
Score
0.00
0.25
0.50
0.75
1.00
Fusion-supporting reads
10000
20000
30000

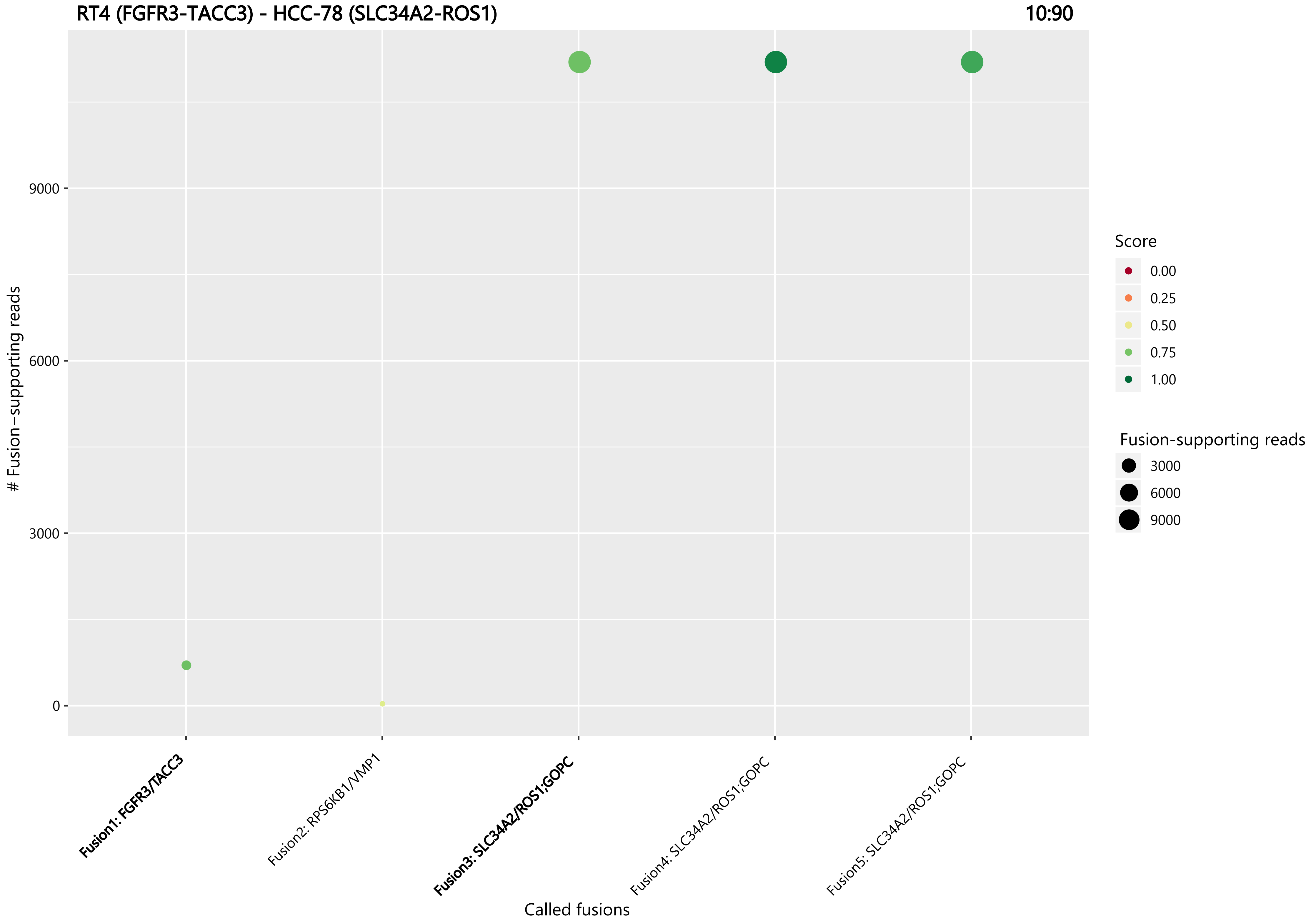

RT4 (FGFR3-TACC3) - HCC-78 (SLC34A2-ROS1)
10:90
Fusion−supporting reads
9000
6000
3000
0
Called fusions
Fusion1: FGFR3/TACC3
Fusion2: RPS6KB1/VMP1
Fusion3: SLC34A2/ROS1;GOPC
Fusion4: SLC34A2/ROS1;GOPC
Fusion5: SLC34A2/ROS1;GOPC
Score
0.00
0.25
0.50
0.75
1.00
Fusion-supporting reads
3000
6000
9000

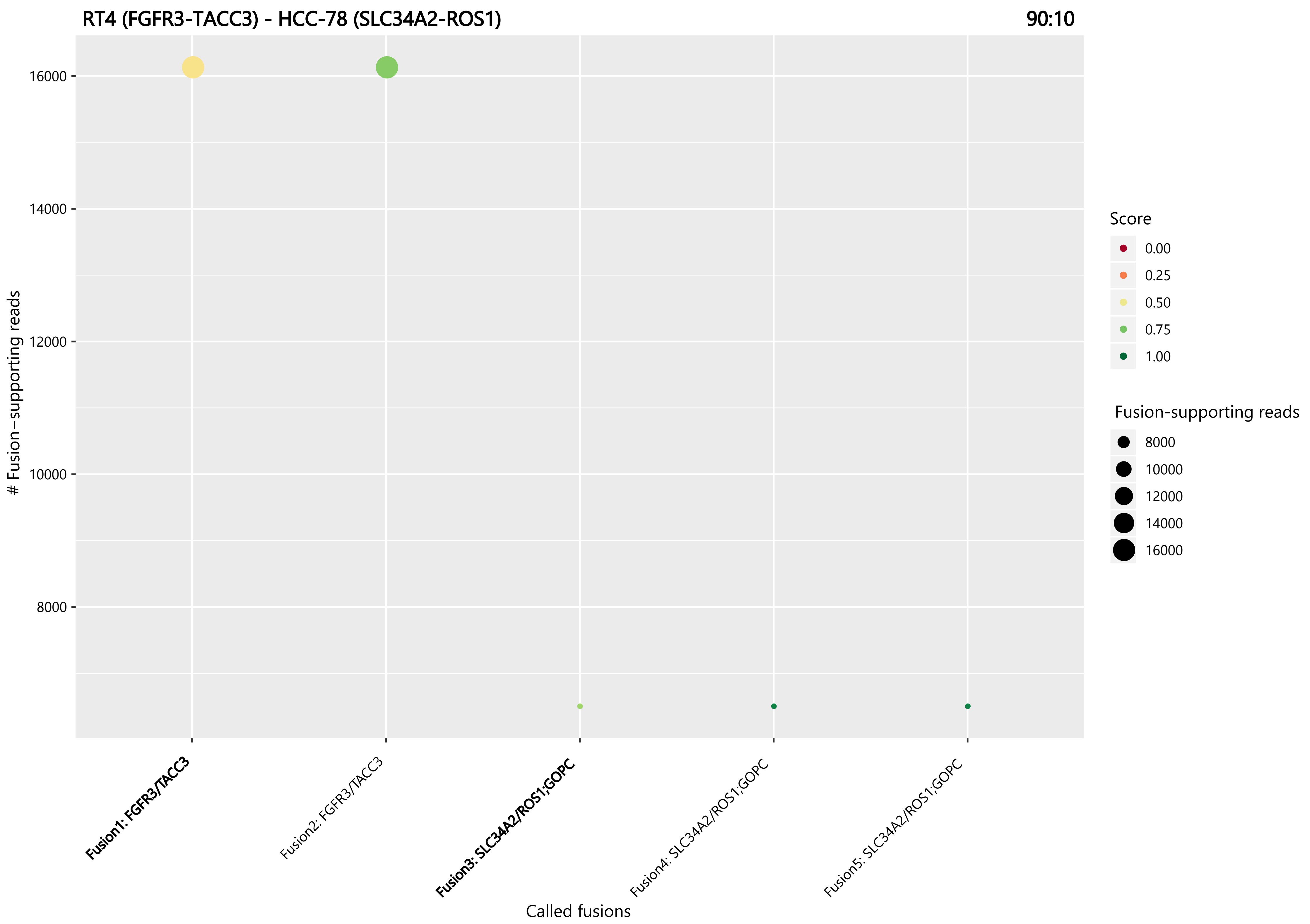
RT4 (FGFR3-TACC3) - HCC-78 (SLC34A2-ROS1)
90:10
Fusion−supporting reads
16000
14000
12000
10000
8000
Called fusions
Fusion1: FGFR3/TACC3
Fusion2: FGFR3/TACC3
Fusion3: SLC34A2/ROS1;GOPC
Fusion4: SLC34A2/ROS1;GOPC
Fusion5: SLC34A2/ROS1;GOPC
Score
0.00
0.25
0.50
0.75
1.00
Fusion-supporting reads
8000
10000
12000
14000
16000

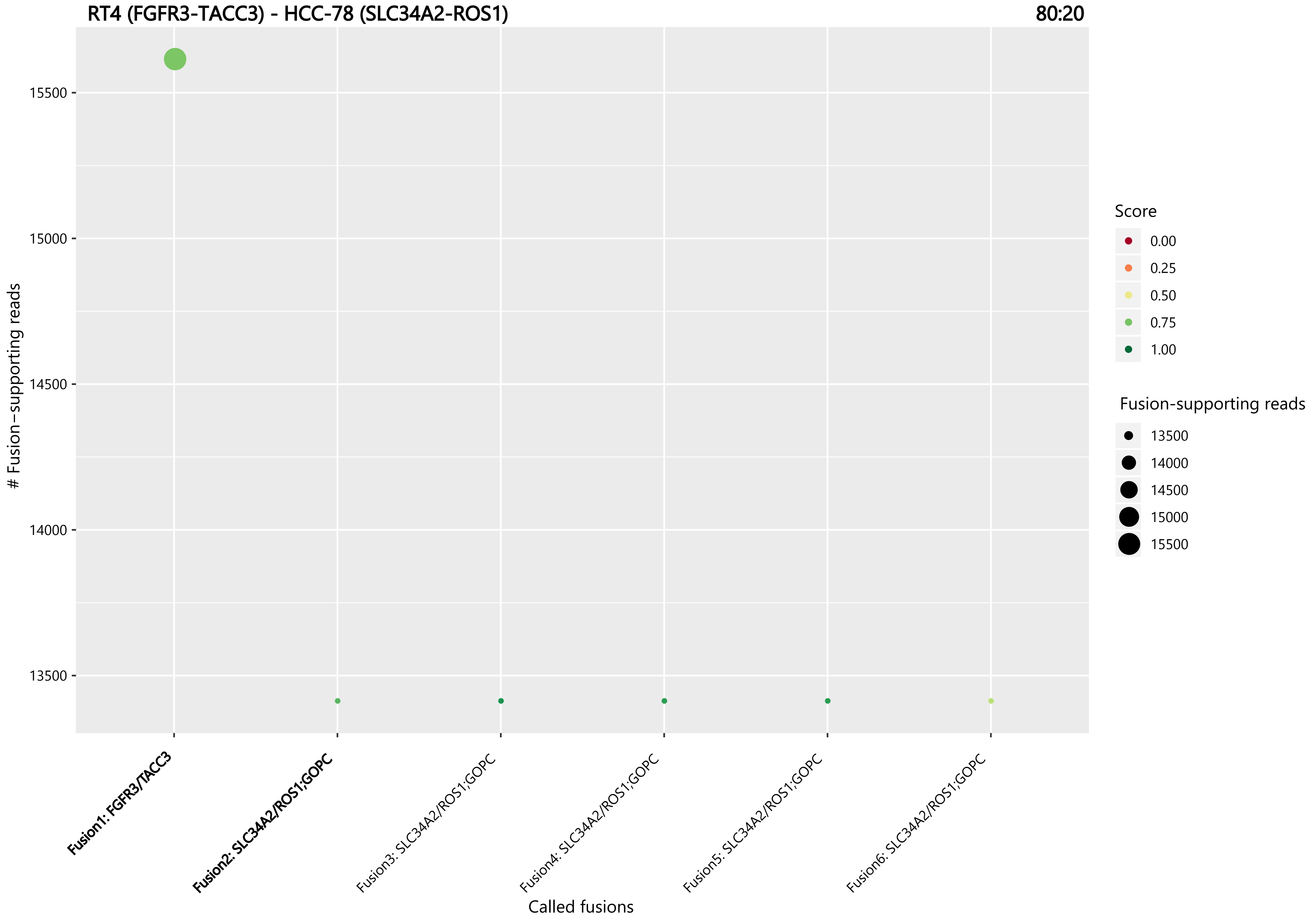

RT4 (FGFR3-TACC3) - HCC-78 (SLC34A2-ROS1)
80:20
15500
15000
14500
14000
13500
Fusion−supporting reads
Fusion1: FGFR3/TACC3
Fusion2: SLC34A2/ROS1;GOPC
Fusion3: SLC34A2/ROS1;GOPC
Fusion4: SLC34A2/ROS1;GOPC
Fusion5: SLC34A2/ROS1;GOPC
Fusion6: SLC34A2/ROS1;GOPC
Called fusions
Score
0.00
0.25
0.50
0.75
1.00
Fusion-supporting reads
13500
14000
14500
15000
15500

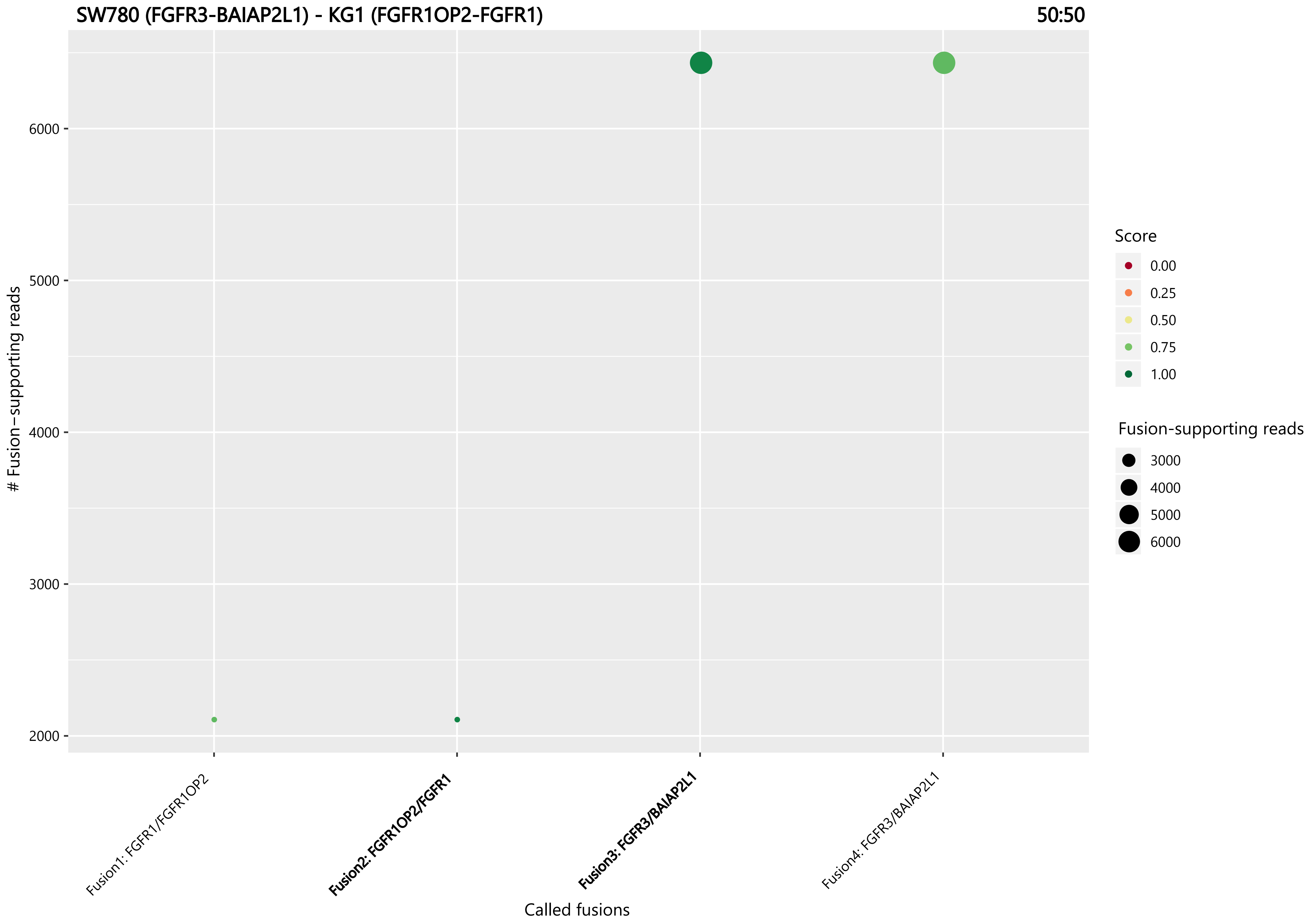

SW780 (FGFR3-BAIAP2L1) - KG1 (FGFR1OP2-FGFR1)
50:50
Fusion−supporting reads
6000
5000
4000
3000
2000
Fusion1: FGFR1/FGFR1OP2
Fusion2: FGFR1OP2/FGFR1
Fusion3: FGFR3/BAIAP2L1
Fusion4: FGFR3/BAIAP2L1
Called fusions
Score
0.00
0.25
0.50
0.75
1.00
Fusion-supporting reads
3000
4000
5000
6000

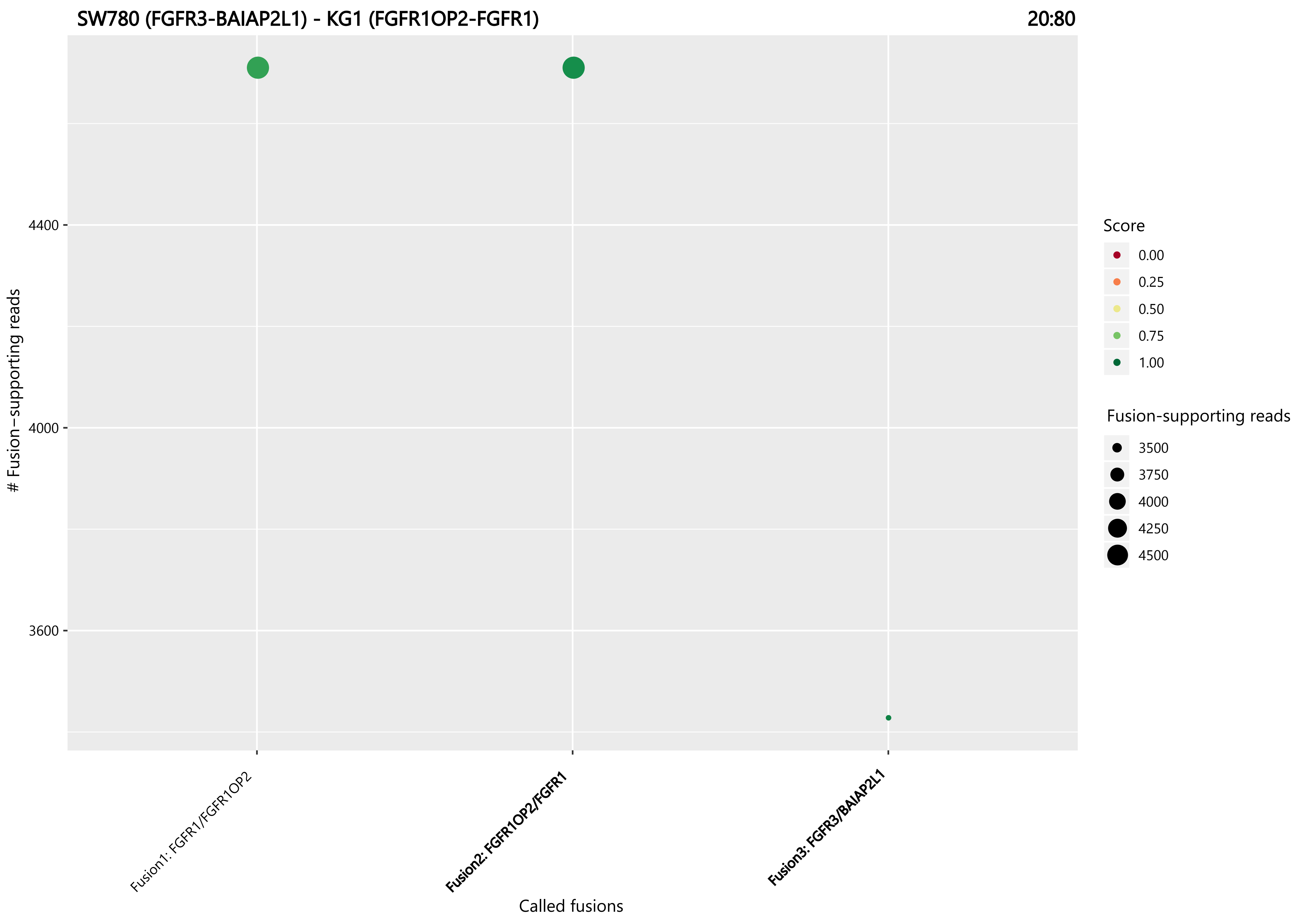
SW780 (FGFR3-BAIAP2L1) - KG1 (FGFR1OP2-FGFR1)
20:80
Fusion−supporting reads
4400
4000
3600
Called fusions
Fusion1: FGFR1/FGFR1OP2
Fusion2: FGFR1OP2/FGFR1
Fusion3: FGFR3/BAIAP2L1
Score
0.00
0.25
0.50
0.75
1.00
Fusion-supporting reads
3500
3750
4000
4250
4500

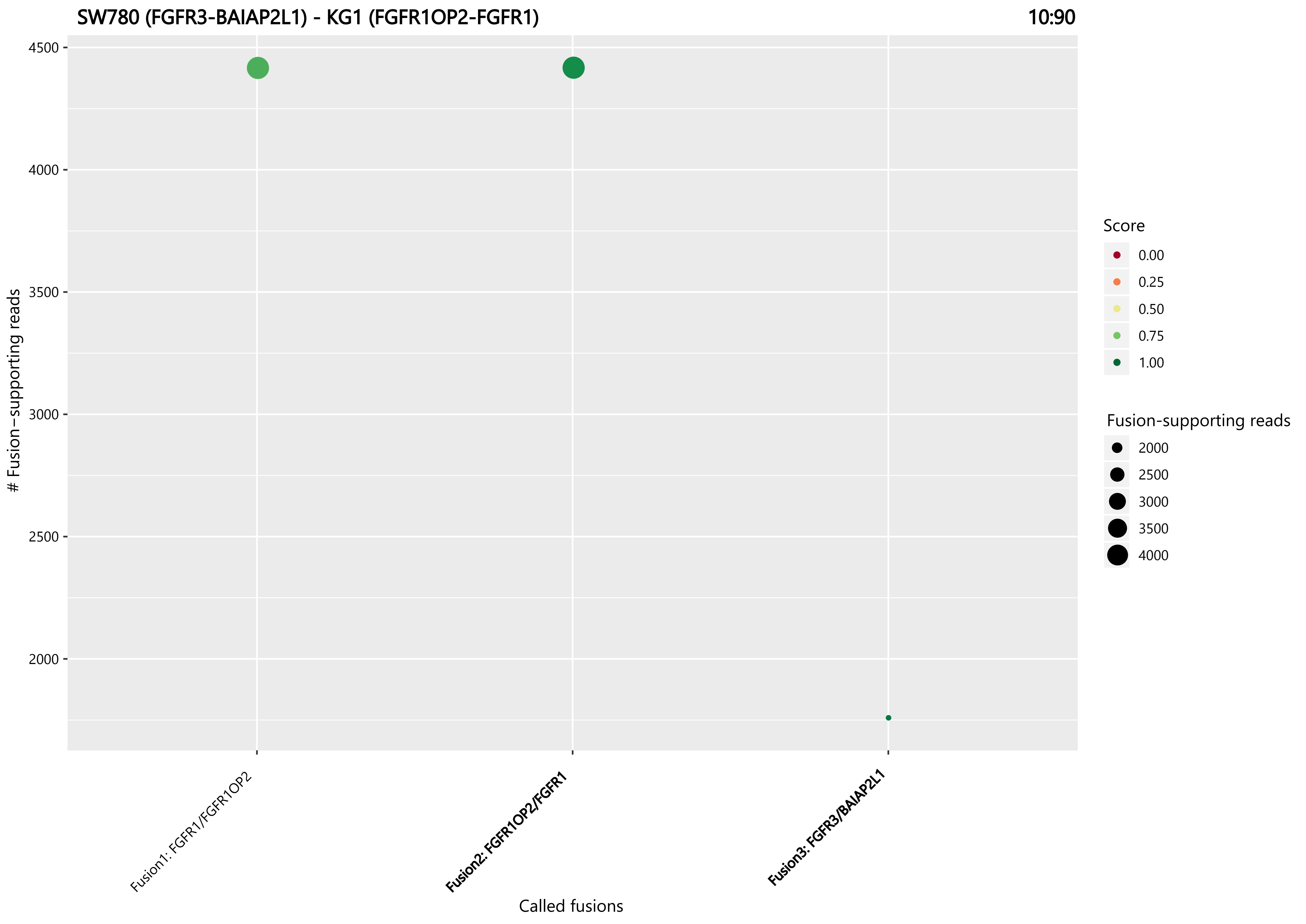

SW780 (FGFR3-BAIAP2L1) - KG1 (FGFR1OP2-FGFR1)
10:90
Fusion−supporting reads
4500
4000
3500
3000
2500
2000
Fusion1: FGFR1/FGFR1OP2
Fusion2: FGFR1OP2/FGFR1
Fusion3: FGFR3/BAIAP2L1
Called fusions
Score
0.00
0.25
0.50
0.75
1.00
Fusion-supporting reads
2000
2500
3000
3500
4000

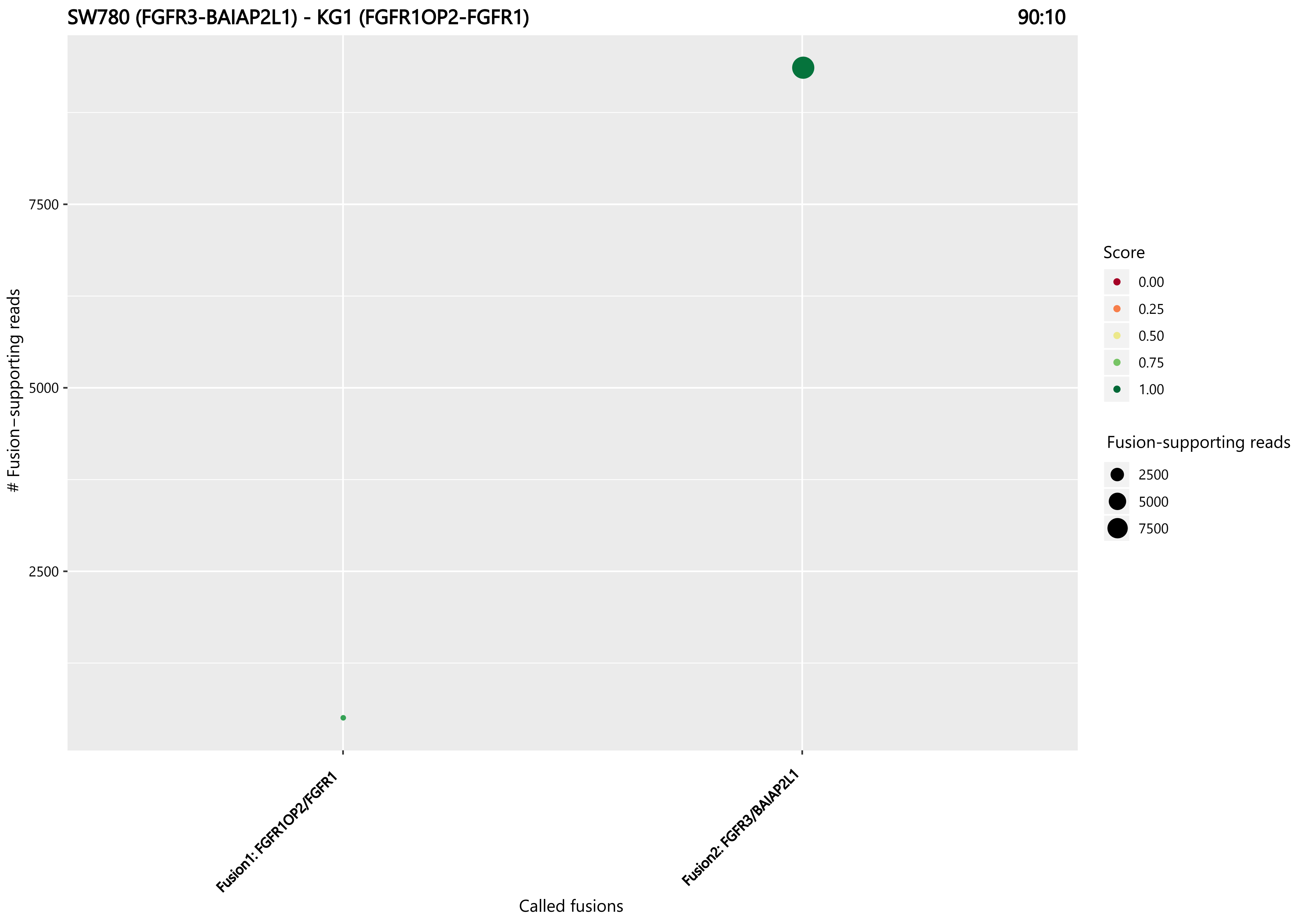

SW780 (FGFR3-BAIAP2L1) - KG1 (FGFR1OP2-FGFR1)
90:10
Fusion−supporting reads
7500
5000
2500
Fusion1: FGFR1OP2/FGFR1
Fusion2: FGFR3/BAIAP2L1
Called fusions
Score
0.00
0.25
0.50
0.75
1.00
Fusion-supporting reads
2500
5000
7500

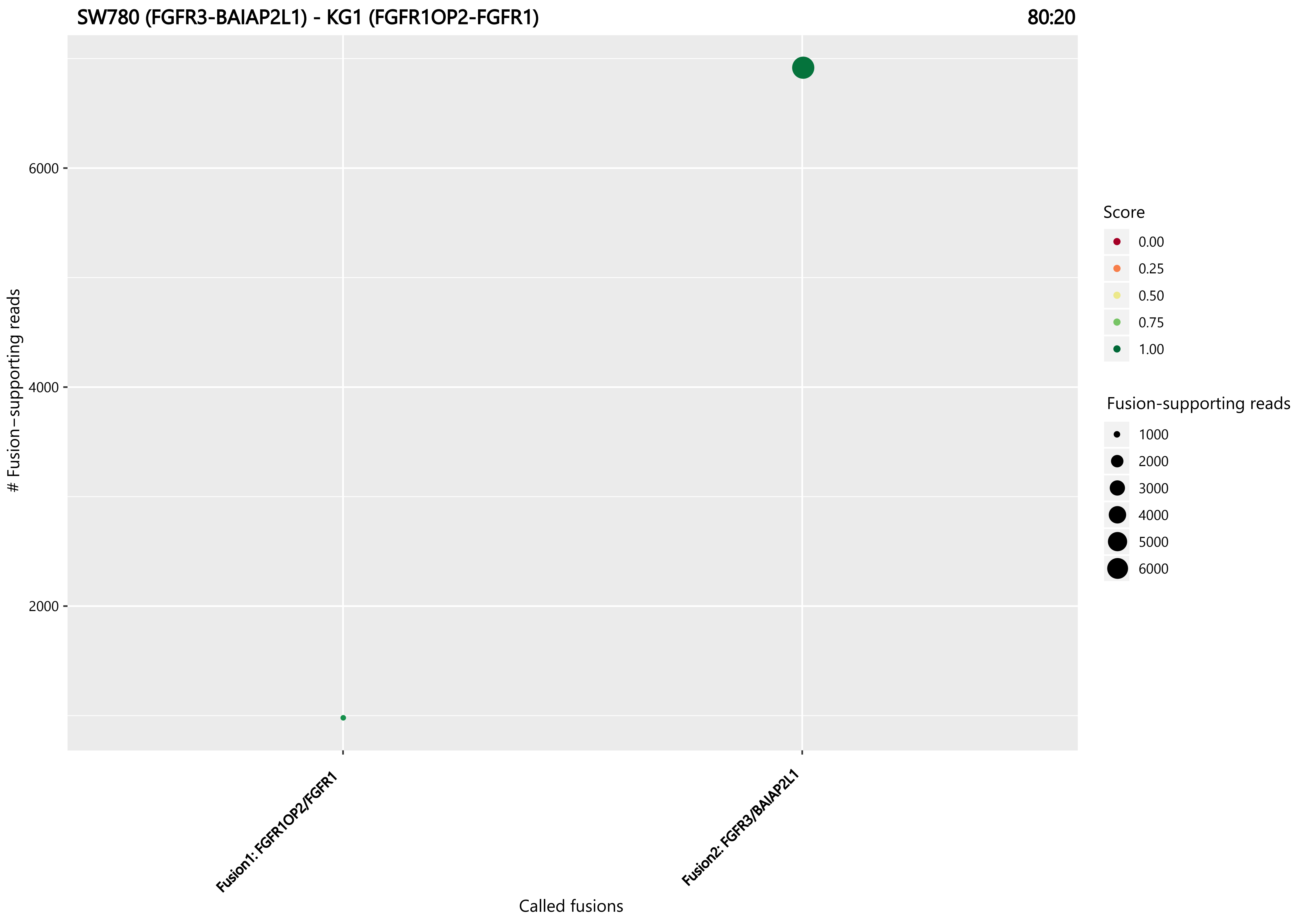

SW780 (FGFR3-BAIAP2L1) - KG1 (FGFR1OP2-FGFR1)
80:20
Fusion−supporting reads
6000
4000
2000
Called fusions
Fusion1: FGFR1OP2/FGFR1
Fusion2: FGFR3/BAIAP2L1
Score
0.00
0.25
0.50
0.75
1.00
Fusion-supporting reads
1000
2000
3000
4000
5000
6000

KIA1549-BRAF

Sample 1

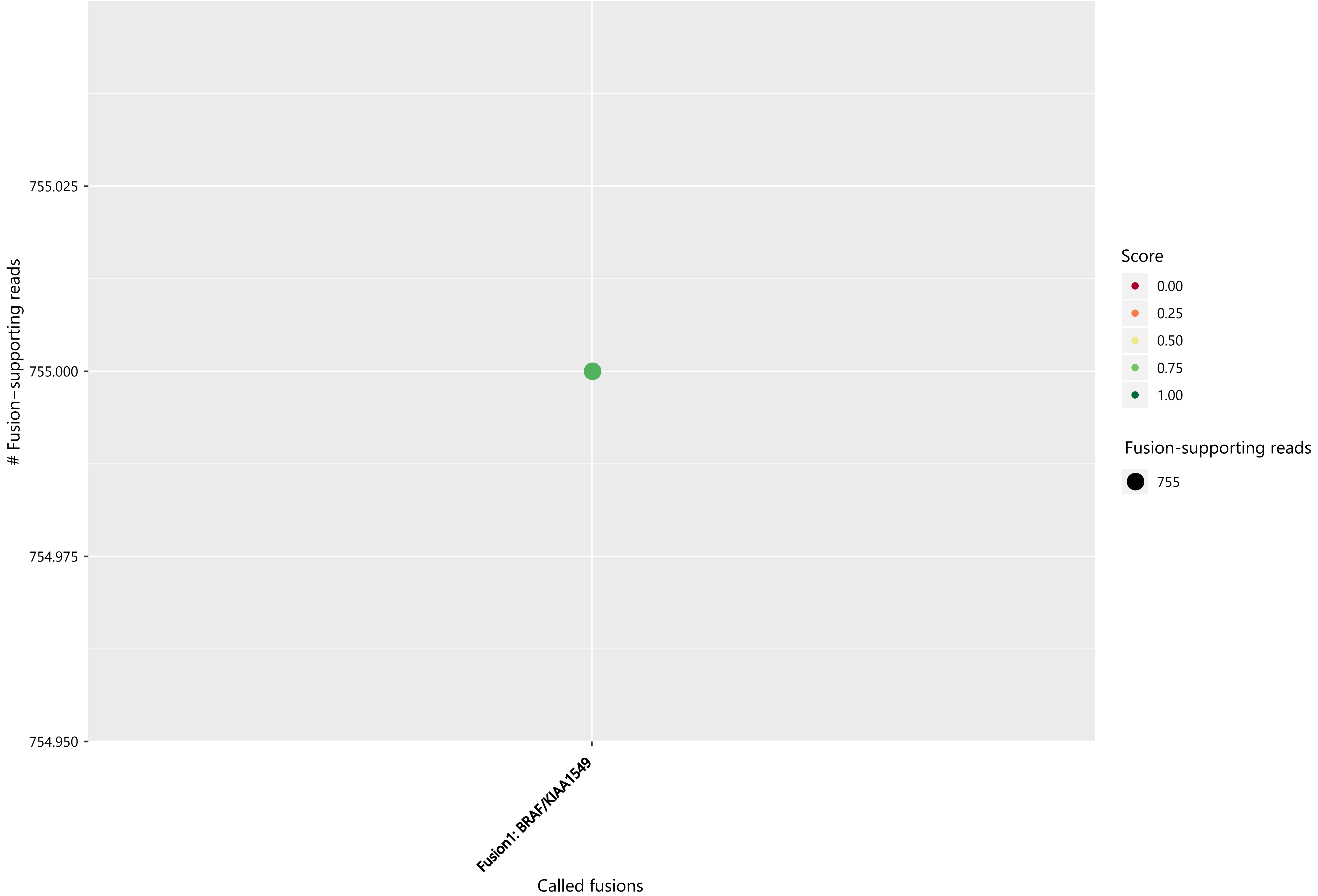

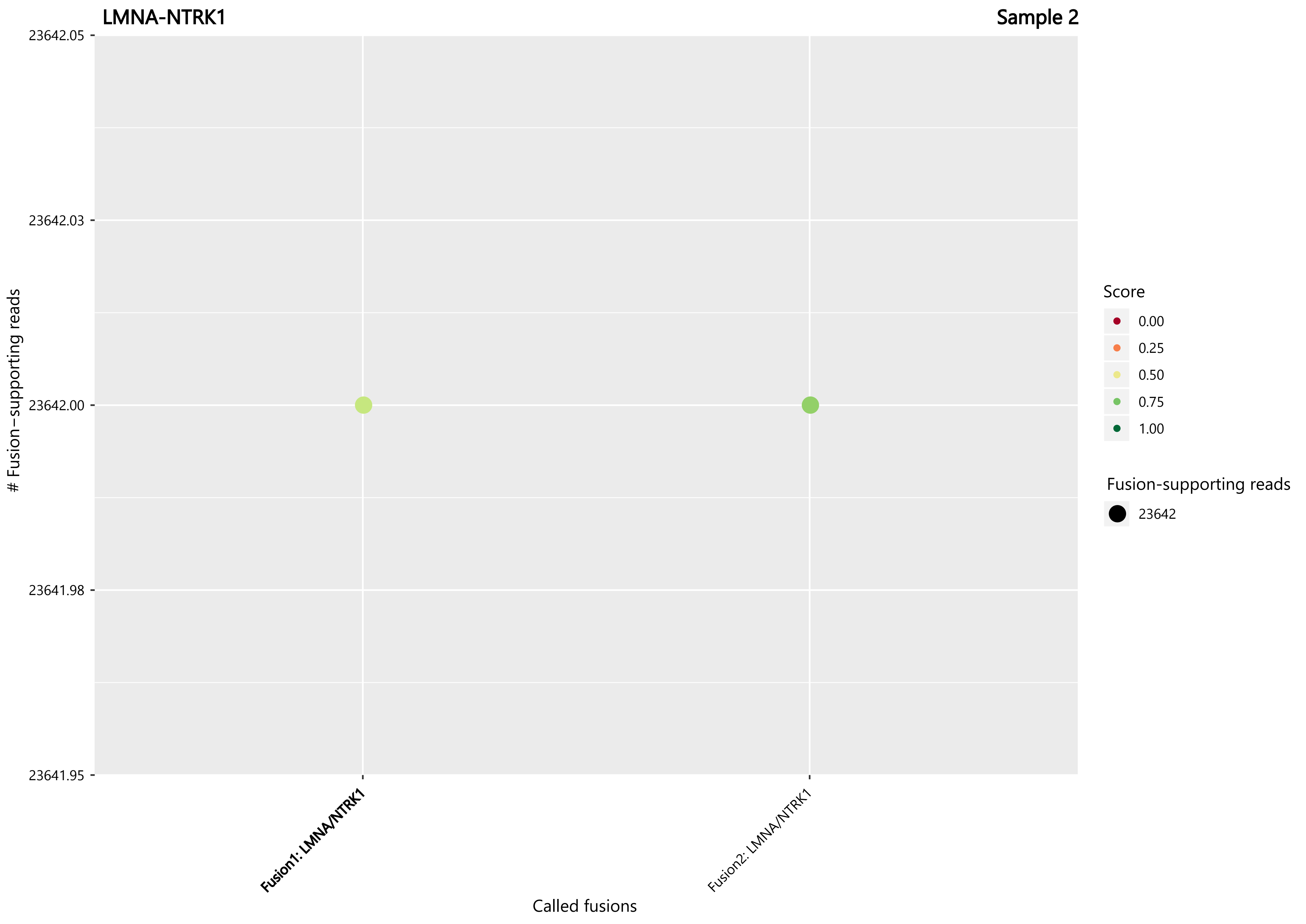

LMNA-NTRK1
Sample 2
Fusion−supporting reads
23642.05
23642.03
23642.00
23641.98
23641.95
Fusion1: LMNA/NTRK1
Fusion2: LMNA/NTRK1
Called fusions
Score
0.00
0.25
0.50
0.75
1.00
Fusion-supporting reads
23642

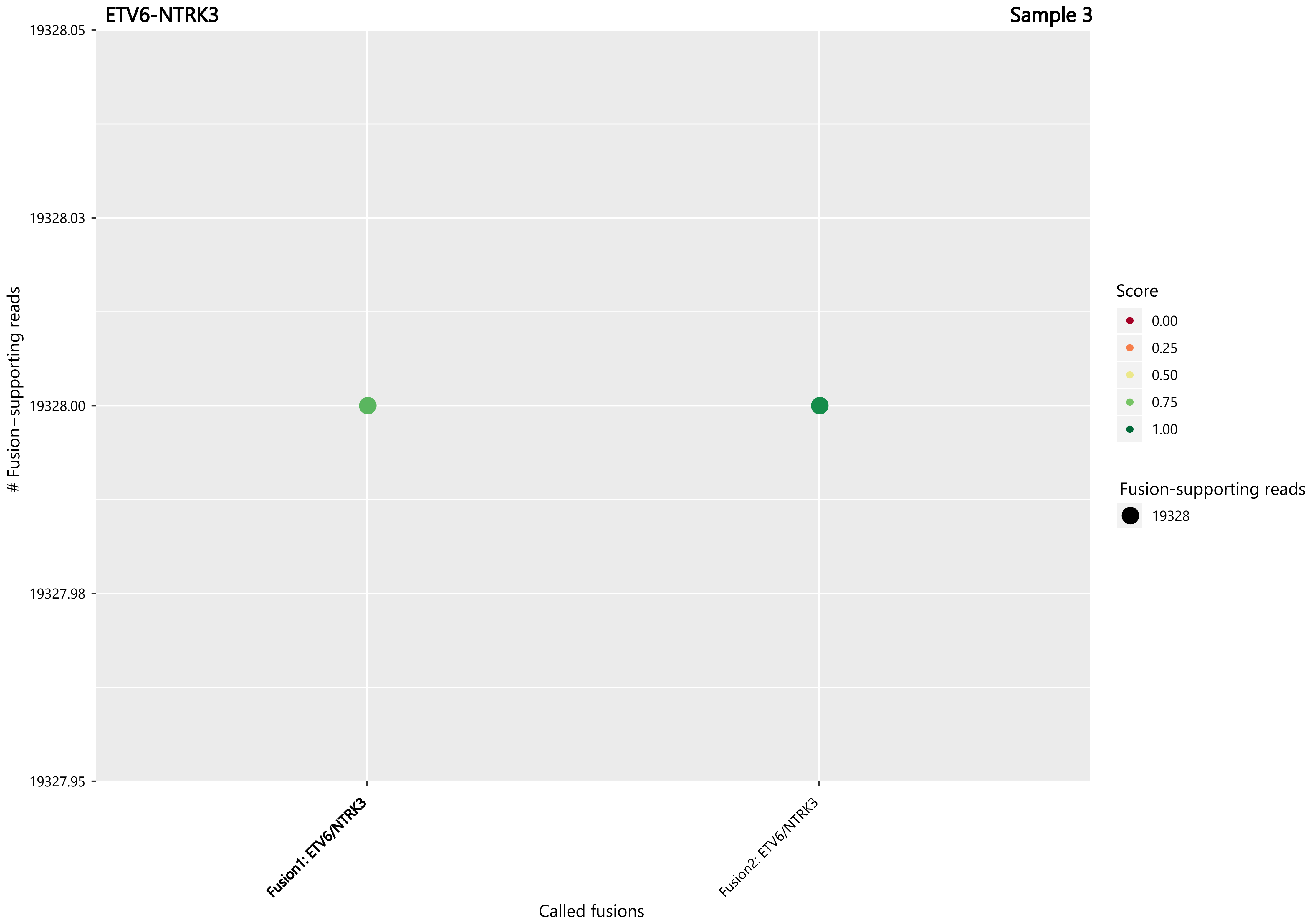
ETV6-NTRK3
Sample 3
19328.05
19328.03
19328.00
19327.98
19327.95
Fusion−supporting reads
Fusion1: ETV6/NTRK3
Fusion2: ETV6/NTRK3
Called fusions
Score
0.00
0.25
0.50
0.75
1.00
Fusion-supporting reads
19328

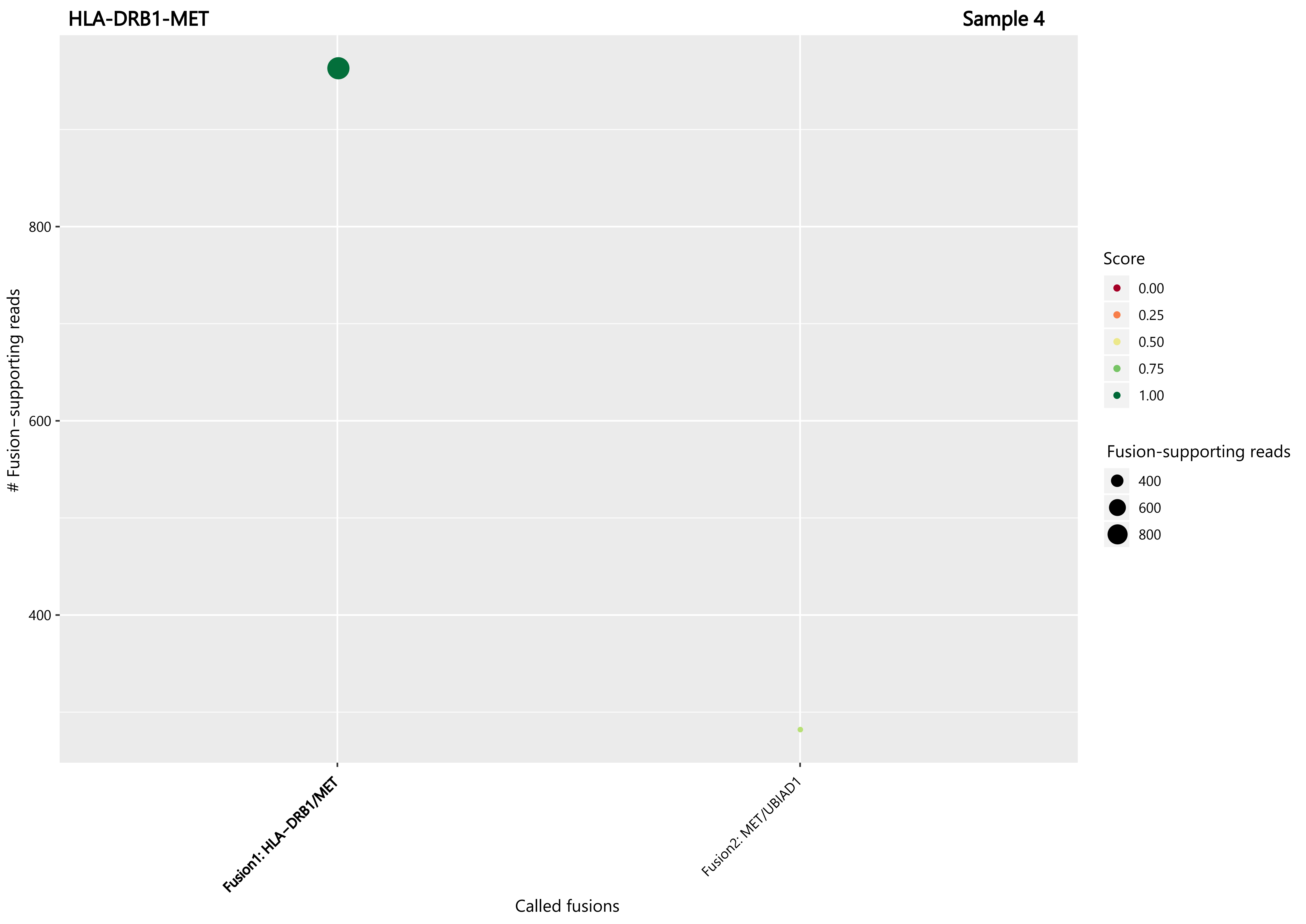
HLA-DRB1-MET
Sample 4
800
600
400
Fusion−supporting reads
Fusion1: HLA−DRB1/MET
Fusion2: MET/UBIAD1
Called fusions
Score
0.00
0.25
0.50
0.75
1.00
Fusion-supporting reads
400
600
800

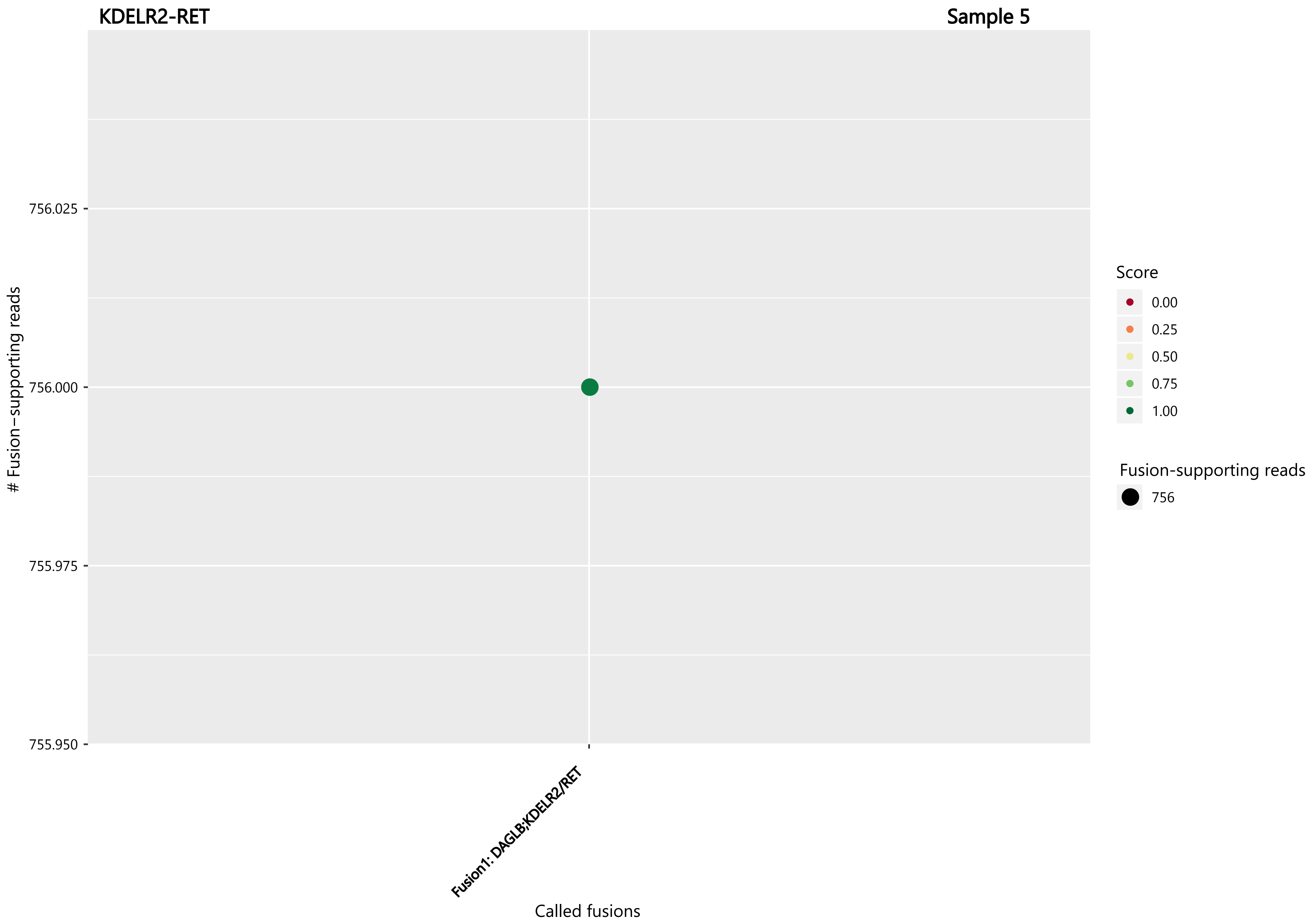

KDELR2-RET
Sample 5
756.025
756.000
755.975
755.950
Fusion−supporting reads
Fusion1: DAGLB;KDELR2/RET
Called fusions
Score
0.00
0.25
0.50
0.75
1.00
Fusion-supporting reads
756

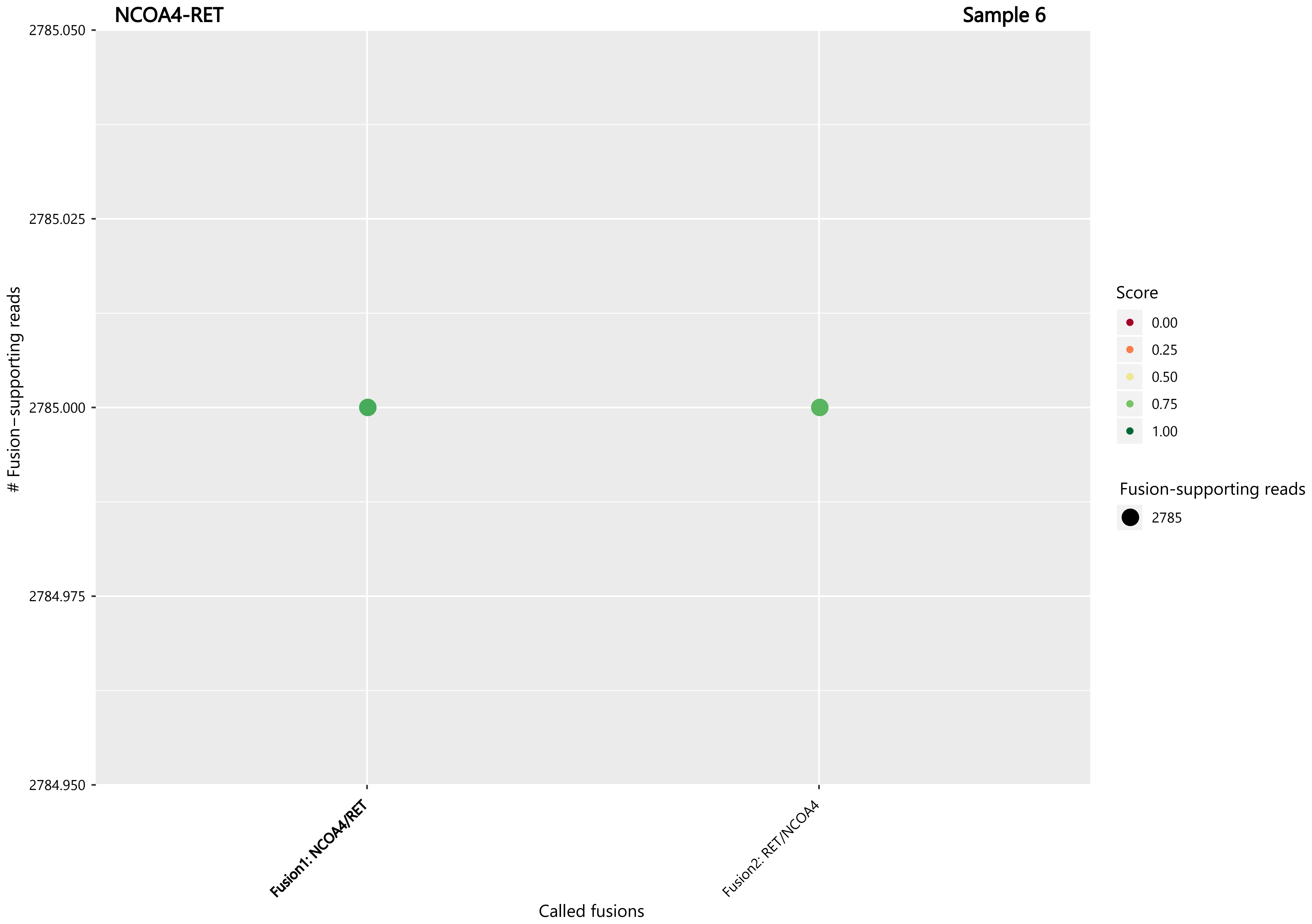

NCOA4-RET
Sample 6
Fusion−supporting reads
2785.050
2785.025
2785.000
2784.975
2784.950
Fusion1: NCOA4/RET
Fusion2: RET/NCOA4
Called fusions
Score
0.00
0.25
0.50
0.75
1.00
Fusion-supporting reads
2785

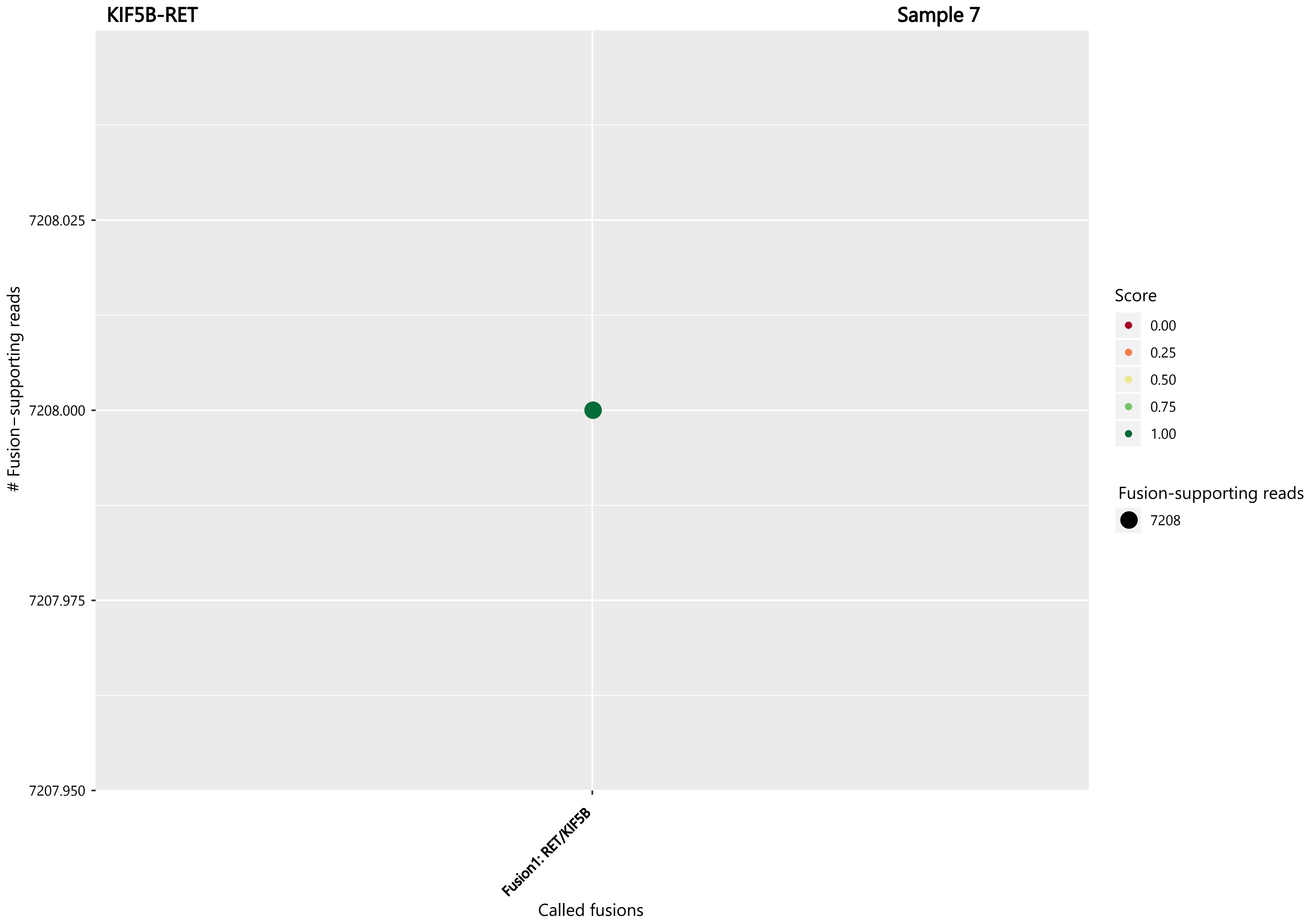

KIF5B-RET
Sample 7
Fusion−supporting reads
7208.025
7208.000
7207.975
7207.950
Fusion1: RET/KIF5B
Called fusions
Score
0.00
0.25
0.50
0.75
1.00
Fusion-supporting reads
7208

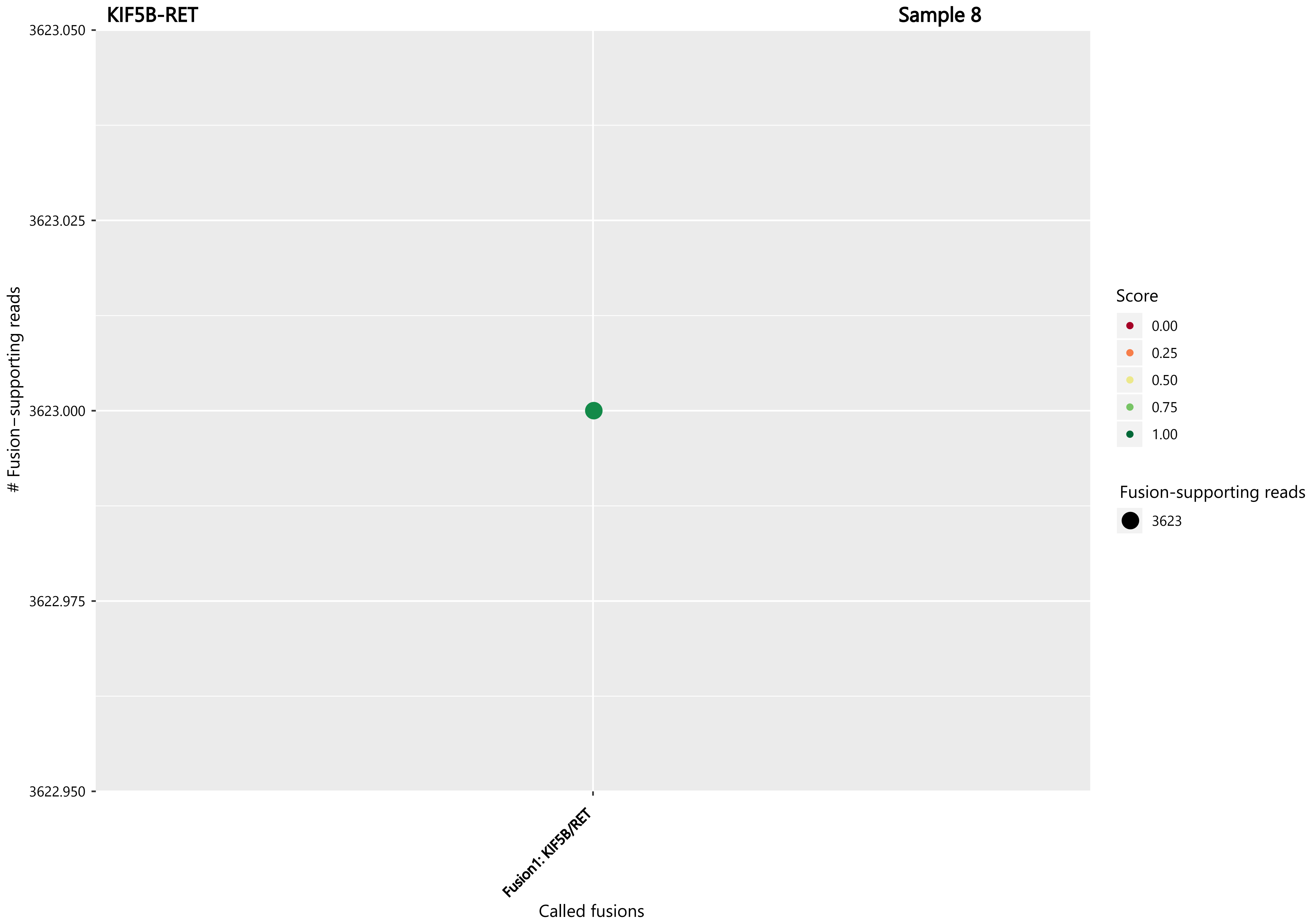
KIF5B-RET
Sample 8
3623.050
3623.025
3623.000
3622.975
3622.950
Fusion−supporting reads
Fusion1: KIF5B/RET
Called fusions
Score
0.00
0.25
0.50
0.75
1.00
Fusion-supporting reads
3623

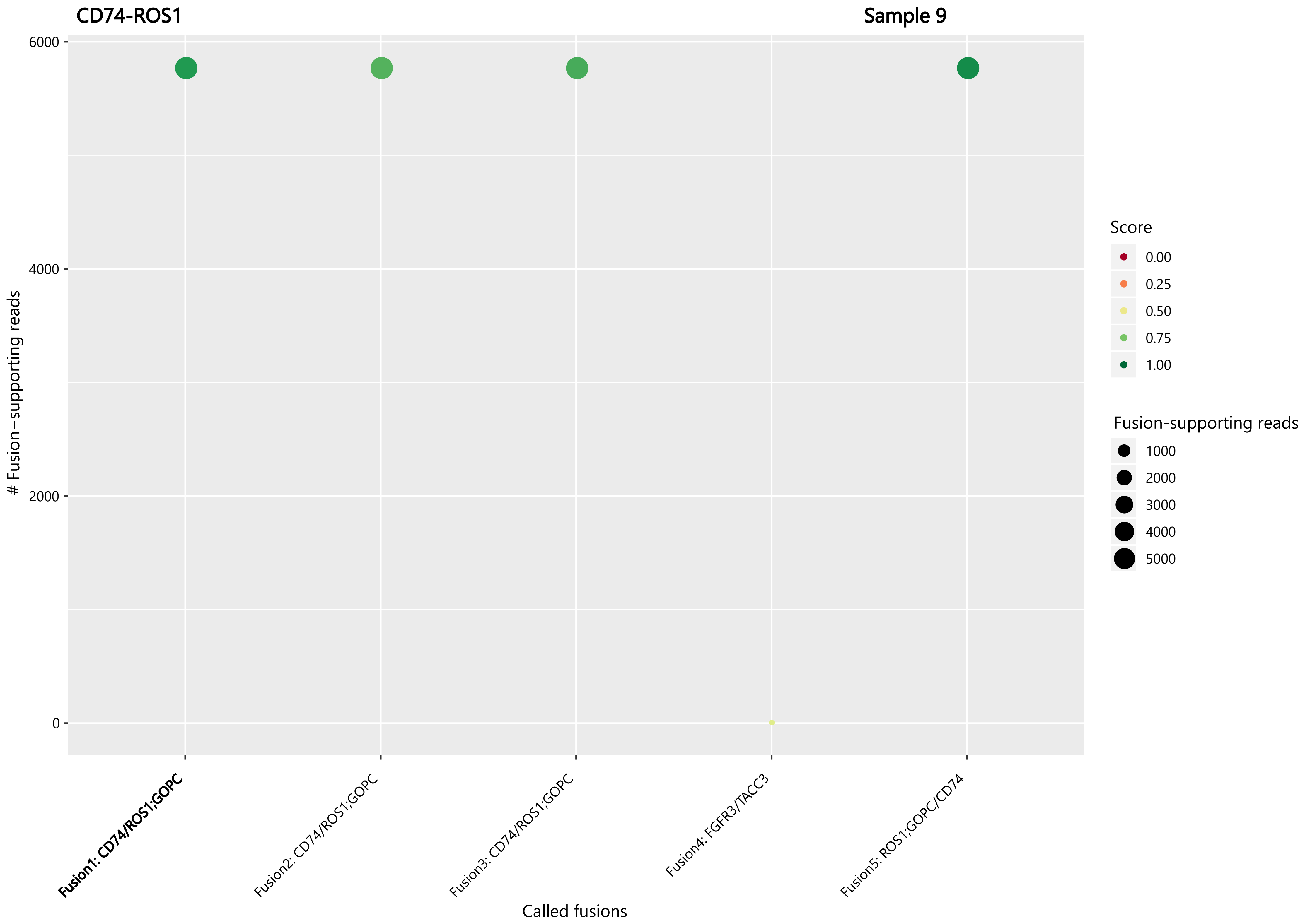

CD74-ROS1
Sample 9
6000
4000
2000
0
Fusion−supporting reads
Score
0.00
0.25
0.50
0.75
1.00
Fusion-supporting reads
1000
2000
3000
4000
5000
Fusion1: CD74/ROS1;GOPC
Fusion2: CD74/ROS1;GOPC
Fusion3: CD74/ROS1;GOPC
Fusion4: FGFR3/TACC3
Fusion5: ROS1;GOPC/CD74
Called fusions

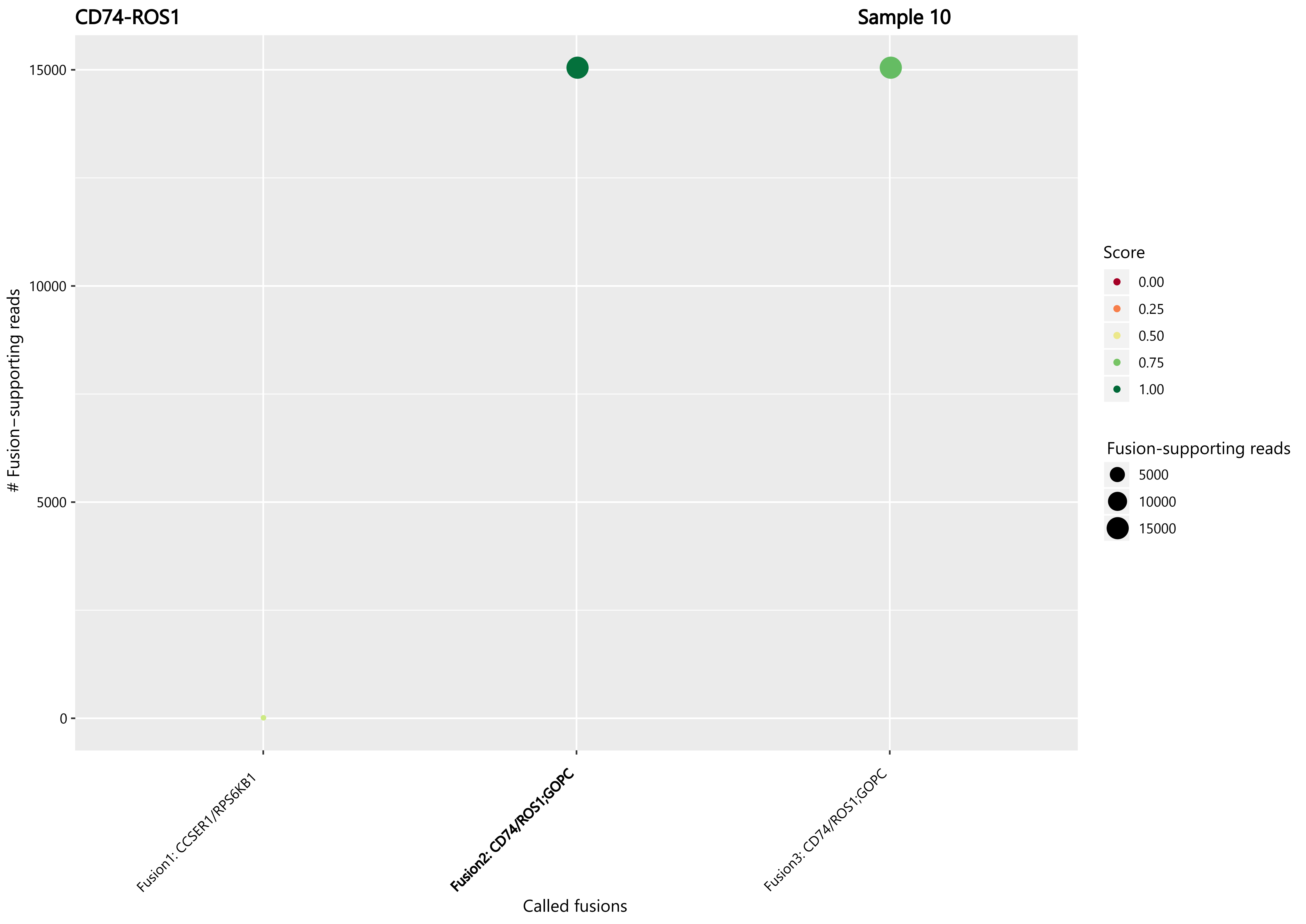

CD74-ROS1
Sample 10
15000
10000
5000
0
Fusion−supporting reads
Fusion1: CCSER1/RPS6KB1
Fusion2: CD74/ROS1;GOPC
Fusion3: CD74/ROS1;GOPC
Called fusions
Score
0.00
0.25
0.50
0.75
1.00
Fusion-supporting reads
5000
10000
15000

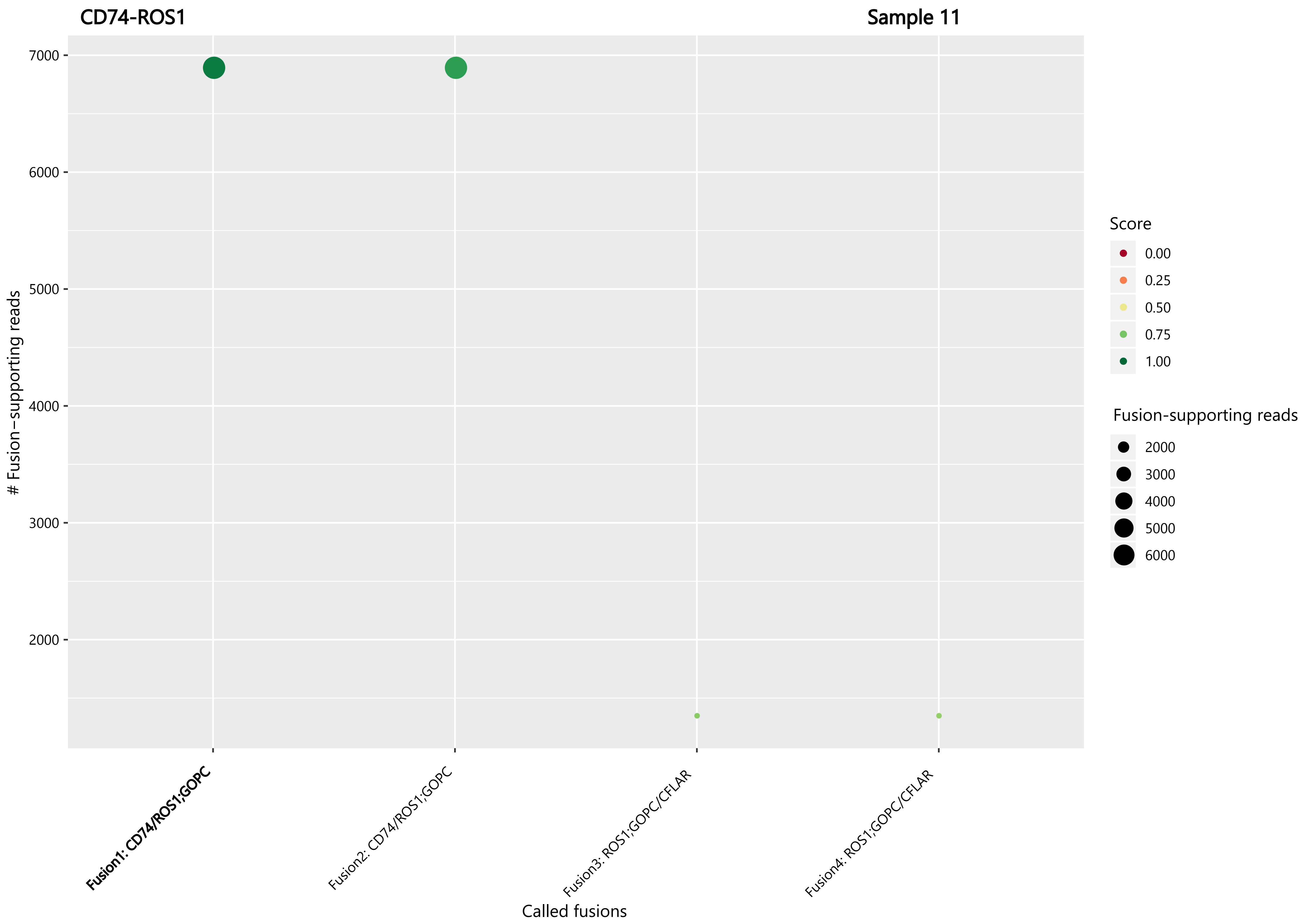

CD74-ROS1
Sample 11
Fusion−supporting reads
7000
6000
5000
4000
3000
2000
Fusion1: CD74/ROS1;GOPC
Fusion2: CD74/ROS1;GOPC
Fusion3: ROS1;GOPC/CFLAR
Fusion4: ROS1;GOPC/CFLAR
Called fusions
Score
0.00
0.25
0.50
0.75
1.00
Fusion-supporting reads
2000
3000
4000
5000
6000

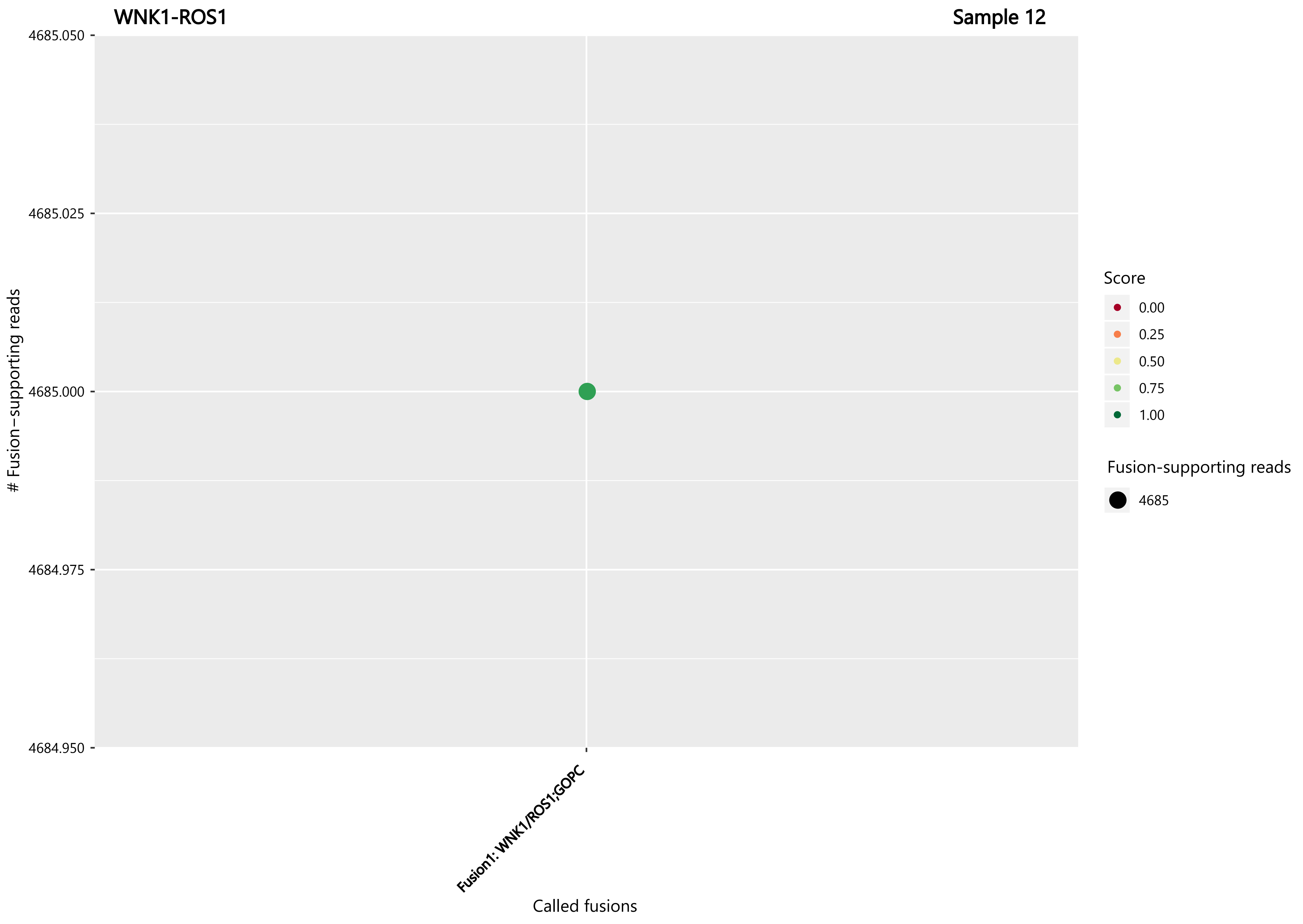

WNK1-ROS1
Sample 12
4685.050
4685.025
4685.000
4684.975
4684.950
Fusion−supporting reads
Fusion1: WNK1/ROS1;GOPC
Called fusions
Score
0.00
0.25
0.50
0.75
1.00
Fusion-supporting reads
4685

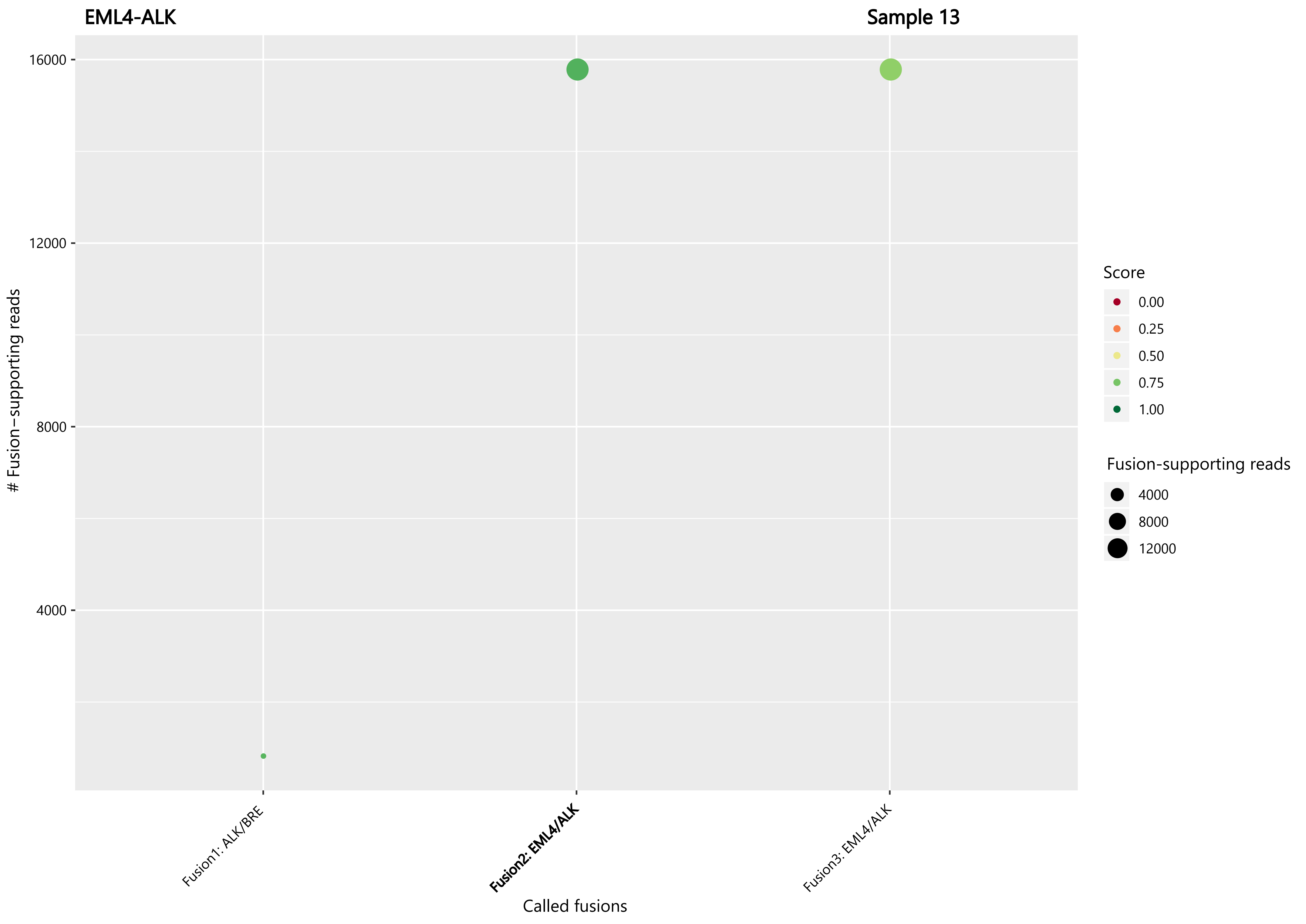
EML4-ALK
Sample 13
16000
12000
8000
4000
Fusion−supporting reads
Fusion1: ALK/BRE
Fusion2: EML4/ALK
Fusion3: EML4/ALK
Called fusions
Score
0.00
0.25
0.50
0.75
1.00
Fusion-supporting reads
4000
8000
12000

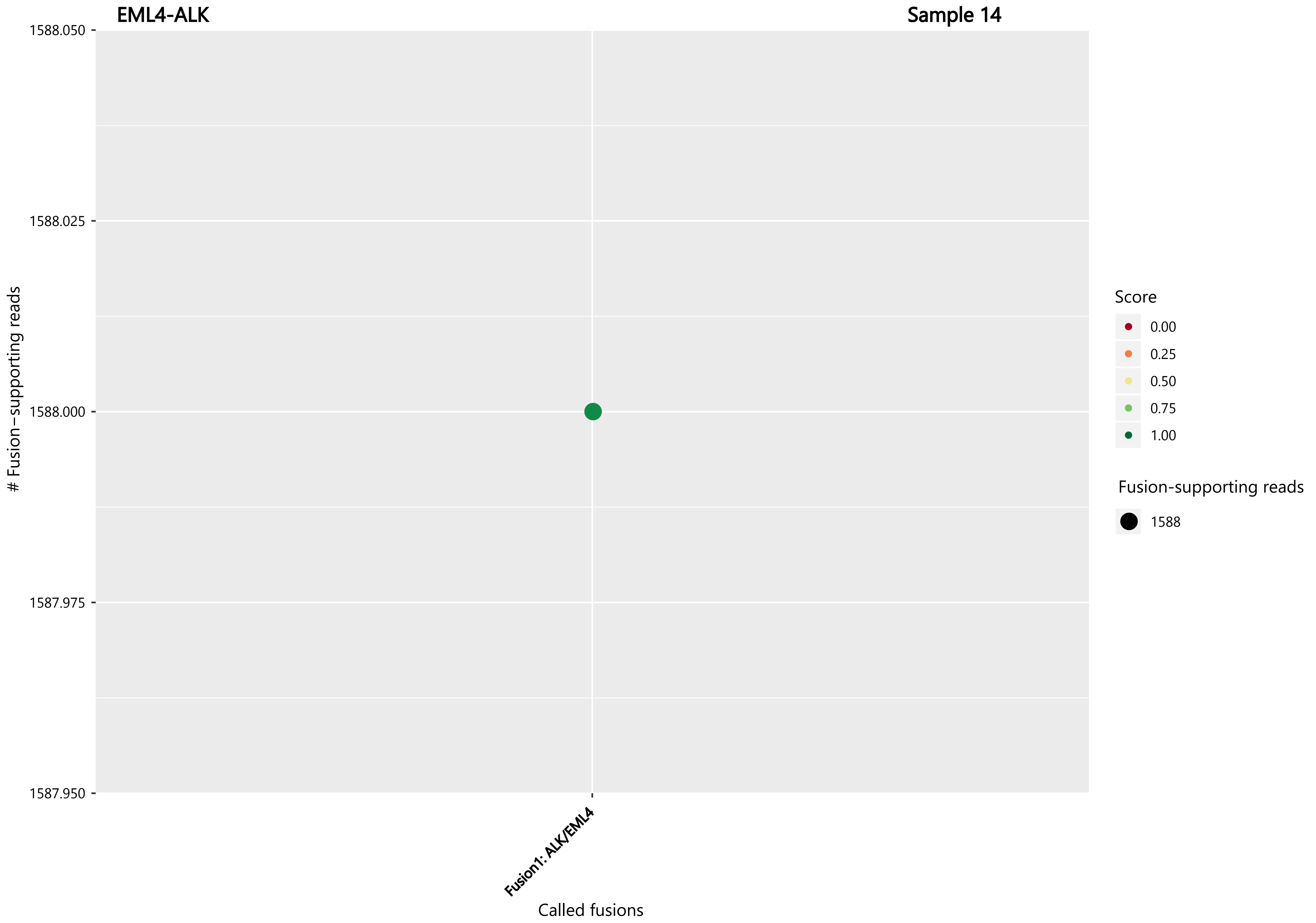

EML4-ALK
Sample 14
1588.050
1588.025
1588.000
1587.975
1587.950
Fusion−supporting reads
Fusion1: ALK/EML4
Called fusions
Score
0.00
0.25
0.50
0.75
1.00
Fusion-supporting reads
1588

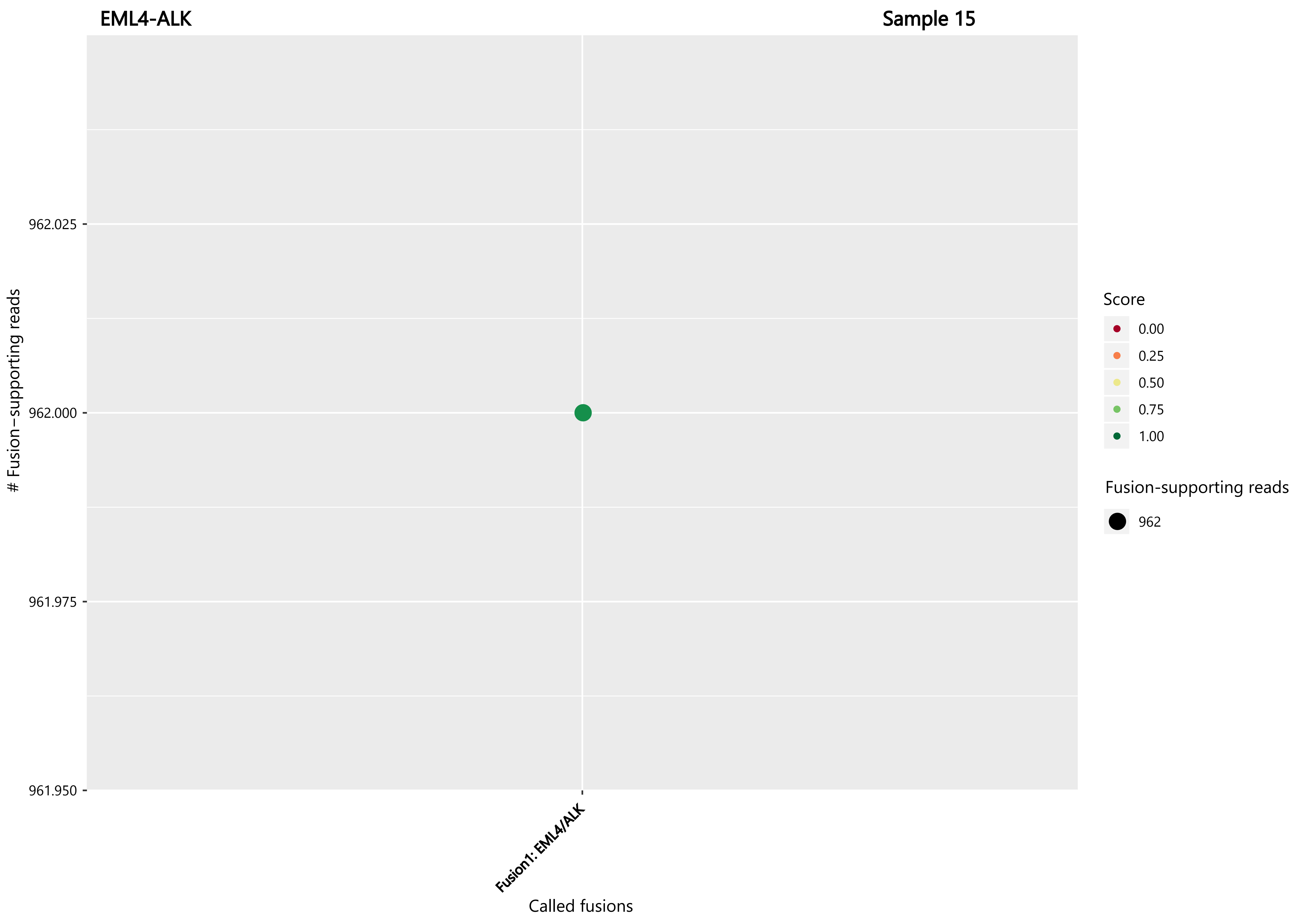
EML4-ALK
Sample 15
Fusion−supporting reads
962.025
962.000
961.975
961.950
Fusion1: EML4/ALK
Called fusions
Score
0.00
0.25
0.50
0.75
1.00
Fusion-supporting reads
962

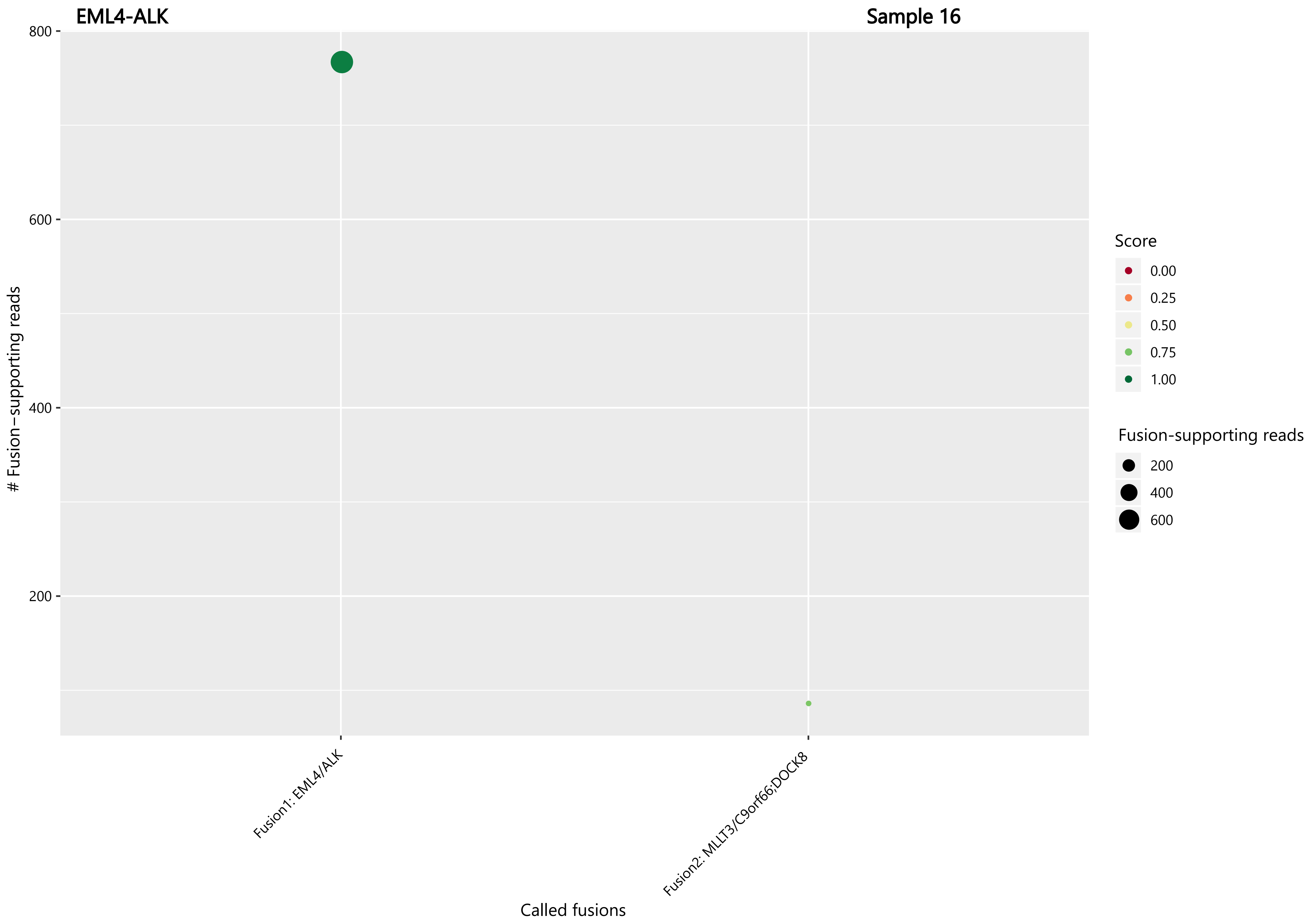
EML4-ALK
Sample 16
800
600
400
200
Fusion−supporting reads
Fusion1: EML4/ALK
Fusion2: MLLT3/C9orf66;DOCK8
Called fusions
Score
0.00
0.25
0.50
0.75
1.00
Fusion-supporting reads
200
400
600

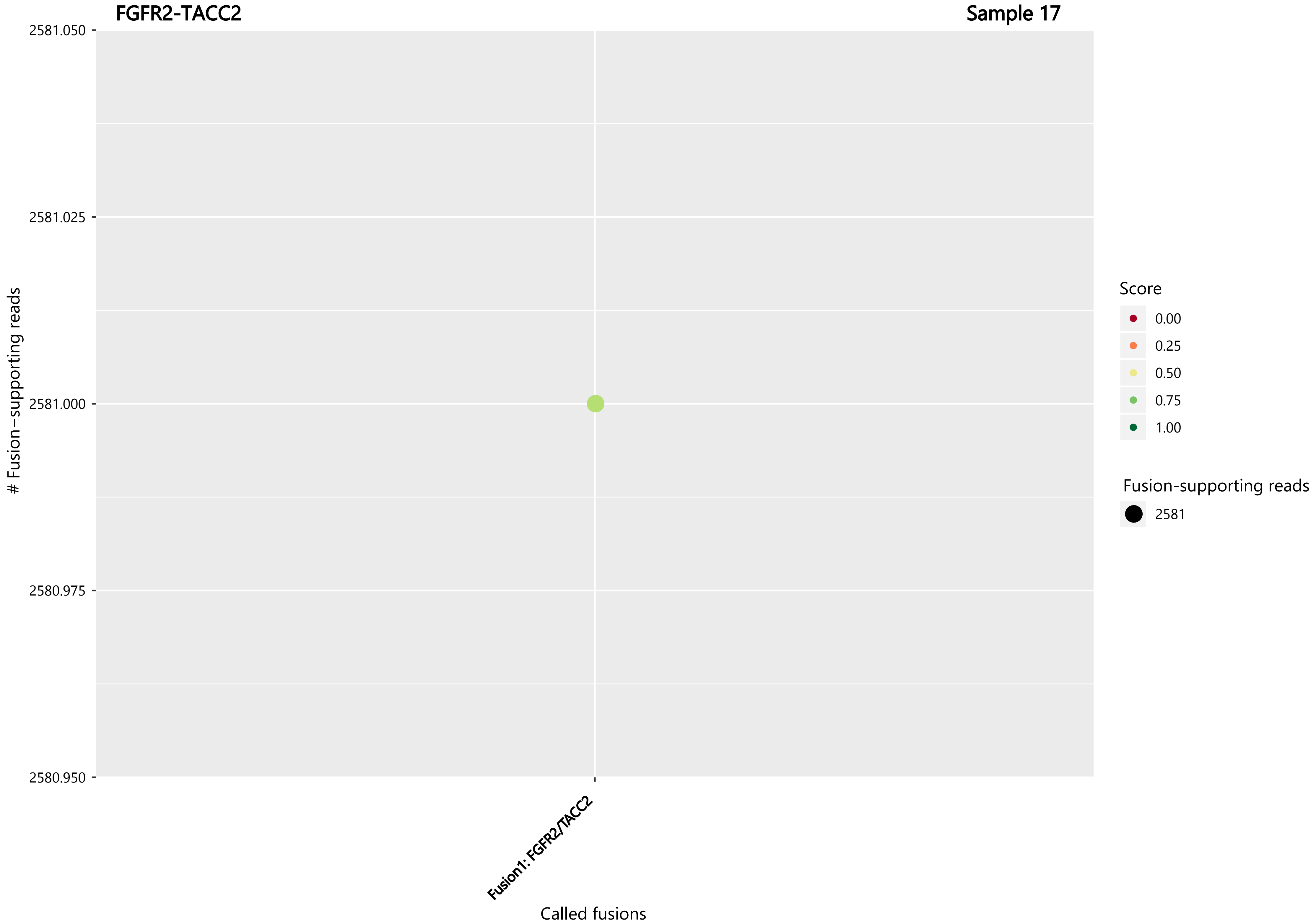

FGFR2-TACC2
Sample 17
2581.050
2581.025
2581.000
2580.975
2580.950
Fusion−supporting reads
Fusion1: FGFR2/TACC2
Called fusions
Score
0.00
0.25
0.50
0.75
1.00
Fusion-supporting reads
2581

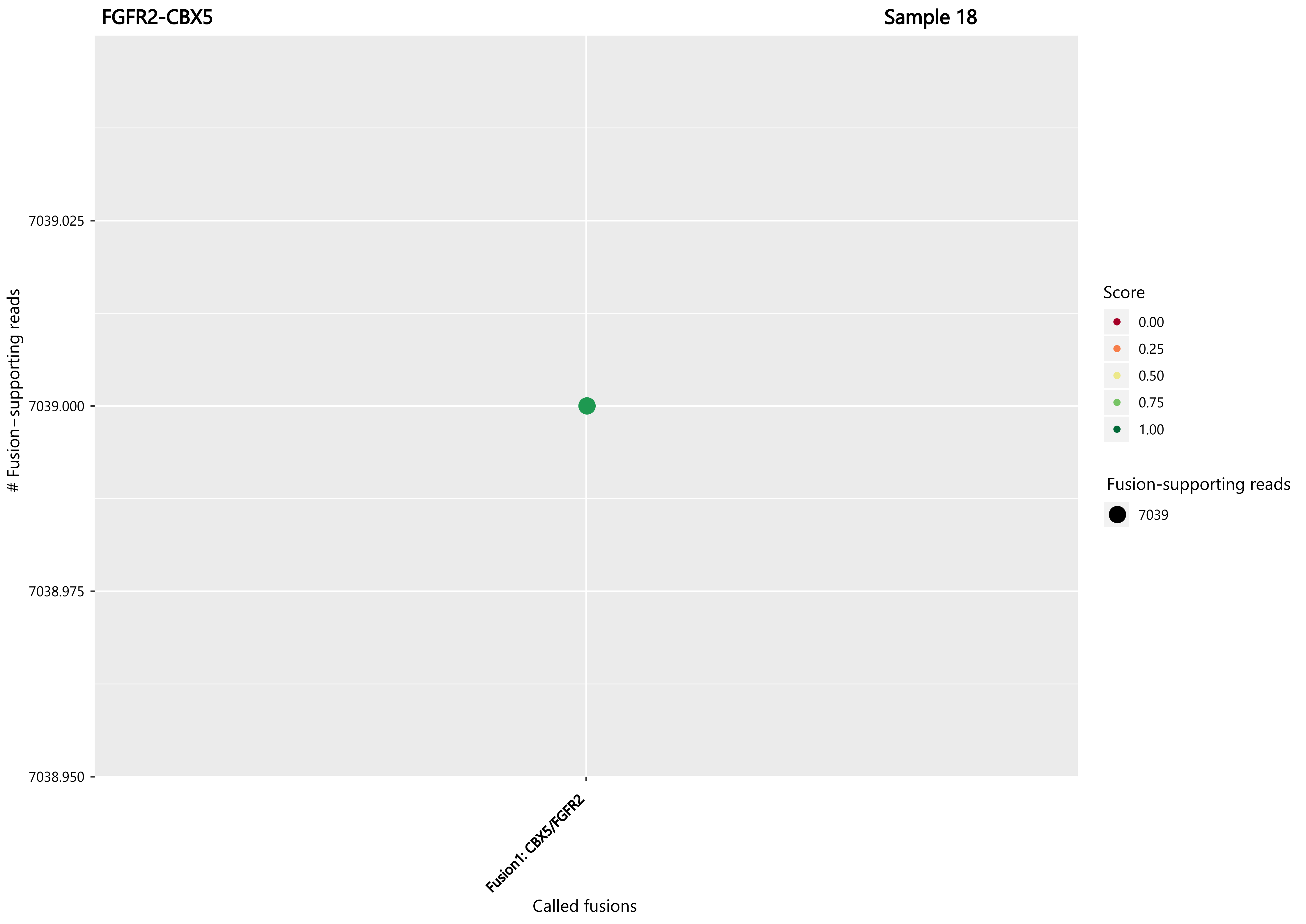
FGFR2-CBX5
Sample 18
Fusion−supporting reads
7039.025
7039.000
7038.975
7038.950
Fusion1: CBX5/FGFR2
Called fusions
Score
0.00
0.25
0.50
0.75
1.00
Fusion-supporting reads
7039
