# Supplementary material for: Detection of gene fusions using targeted next-generation sequencing: a comparative evaluation
Source: BMC Med Genomics. 2021 Feb 27;14:62. doi: 10.1186/s12920-021-00909-y (PMC7912891; doi:10.1186/s12920-021-00909-y)
Supplement: Supplementary file 15 — Additional file 15: Fig. S15. Fusions detected with the SureSelect XT HS Custom Panel (Agilent) (v4.0.1.46) for all samples. Metrics such as quality control scores, in-frame status or filter thresholds were plotted when available. In cases where the same fusion was identified more than once within the same sample, a unique numbering scheme was added at the end of the name to differentiate the candidate fusions. The numbering however, does not imply any special order or preference over the other fusions with the same name. The putative detected fusions were arranged in decreasing order based on the number of fusion-supporting reads. The expected fusion for each sample was highlighted in bold. [file 12920_2021_909_MOESM15_ESM.pdf]

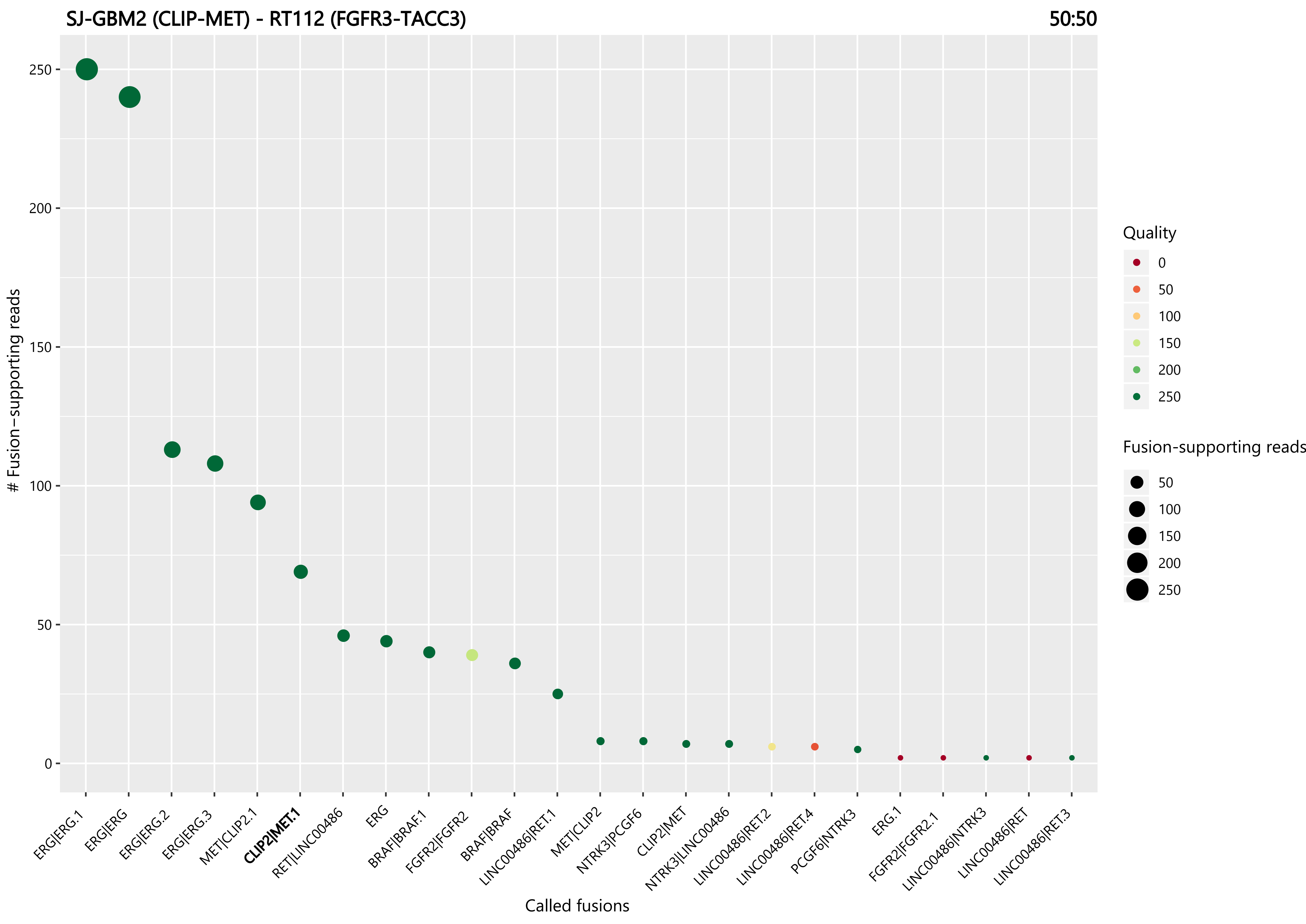
SJ-GBM2 (CLIP-MET) - RT112 (FGFR3-TACC3)
50:50
Fusion−supporting reads
0
50
100
150
200
250
Called fusions
ERG|ERG.1
ERG|ERG
ERG|ERG.2
ERG|ERG.3
MET|CLIP2.1
CLIP2|MET.1
RET|LINC00486
ERG
BRAF|BRAF.1
FGFR2|FGFR2
BRAF|BRAF
LINC00486|RET.1
MET|CLIP2
NTRK3|PCGF6
CLIP2|MET
NTRK3|LINC00486
LINC00486|RET.2
LINC00486|RET.4
PCGF6|NTRK3
ERG.1
FGFR2|FGFR2.1
LINC00486|NTRK3
LINC00486|RET
LINC00486|RET.3
Quality
0
50
100
150
200
250
Fusion-supporting reads
50
100
150
200
250

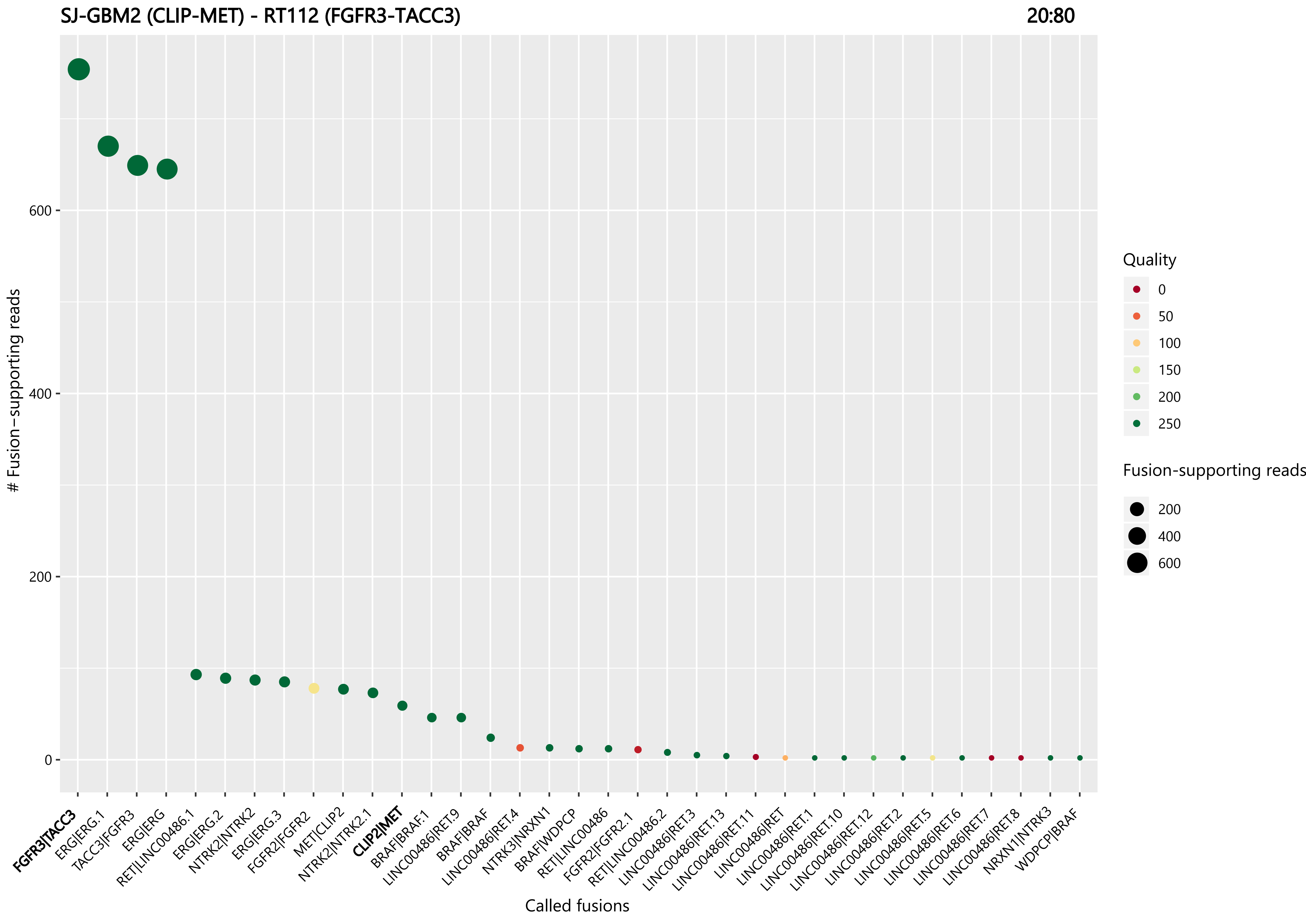

SJ-GBM2 (CLIP-MET) - RT112 (FGFR3-TACC3)
20:80
Fusion−supporting reads
600
400
200
0
Called fusions
FGFR3|TACC3
ERG|ERG.1
TACC3|FGFR3
ERG|ERG
RET|LINC00486.1
ERG|ERG.2
NTRK2|NTRK2
ERG|ERG.3
FGFR2|FGFR2
MET|CLIP2
NTRK2|NTRK2.1
CLIP2|MET
BRAF|BRAF.1
LINC00486|RET.9
BRAF|BRAF
LINC00486|RET.4
NTRK3|NRXN1
BRAF|WDPCP
RET|LINC00486
FGFR2|FGFR2.1
RET|LINC00486.2
LINC00486|RET.3
LINC00486|RET.13
LINC00486|RET.11
LINC00486|RET
LINC00486|RET.1
LINC00486|RET.10
LINC00486|RET.12
LINC00486|RET.2
LINC00486|RET.5
LINC00486|RET.6
LINC00486|RET.7
LINC00486|RET.8
NRXN1|NTRK3
WDPCP|BRAF
Quality
0
50
100
150
200
250
Fusion-supporting reads
200
400
600

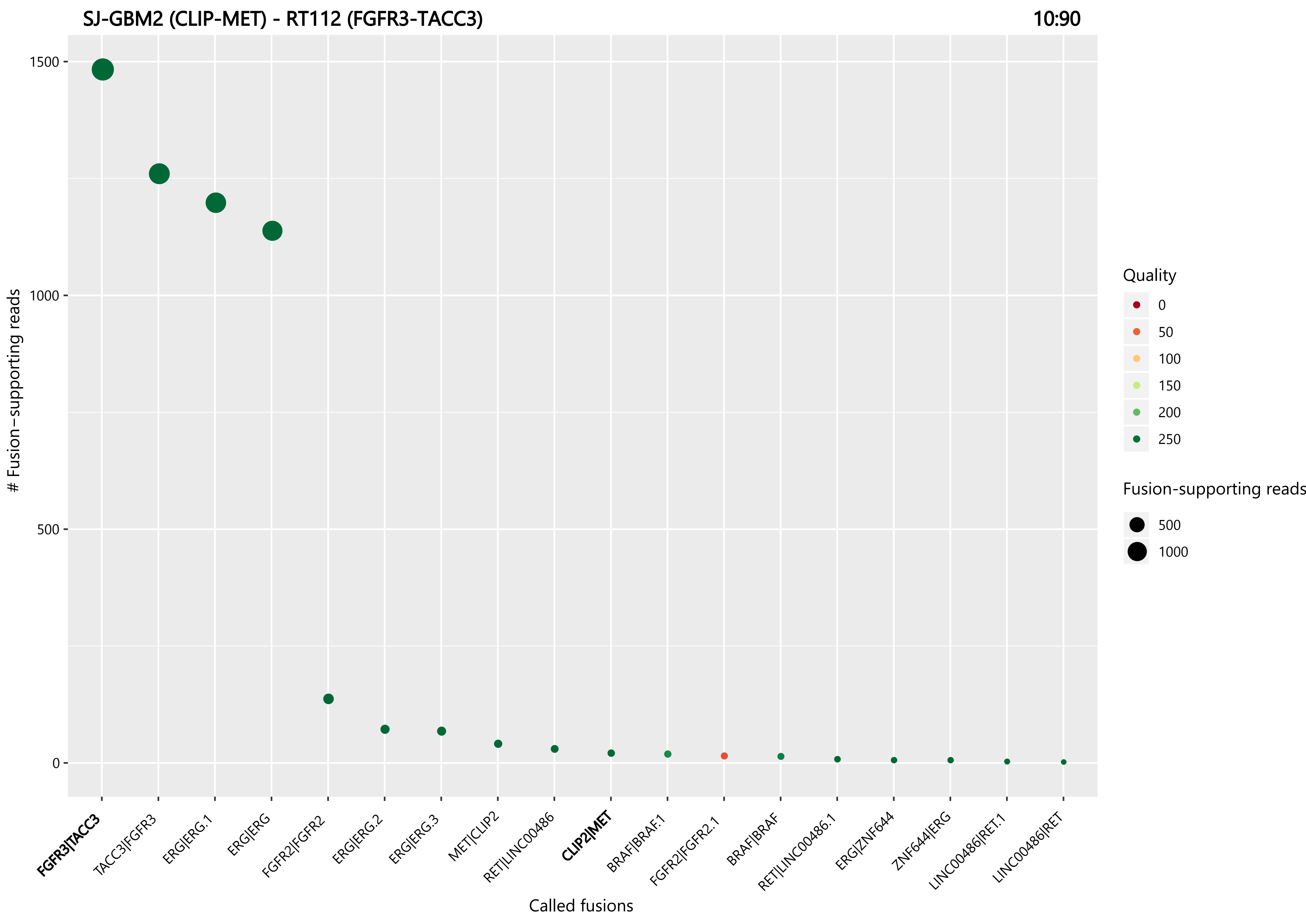
SJ-GBM2 (CLIP-MET) - RT112 (FGFR3-TACC3)
10:90
1500
1000
500
0
Fusion−supporting reads
FGFR3|TACC3
TACC3|FGFR3
ERG|ERG.1
ERG|ERG
FGFR2|FGFR2
ERG|ERG.2
ERG|ERG.3
MET|CLIP2
RET|LINC00486
CLIP2|MET
BRAF|BRAF.1
FGFR2|FGFR2.1
BRAF|BRAF
RET|LINC00486.1
ERG|ZNF644
ZNF644|ERG
LINC00486|RET.1
LINC00486|RET
Called fusions
Quality
0
50
100
150
200
250
Fusion-supporting reads
500
1000

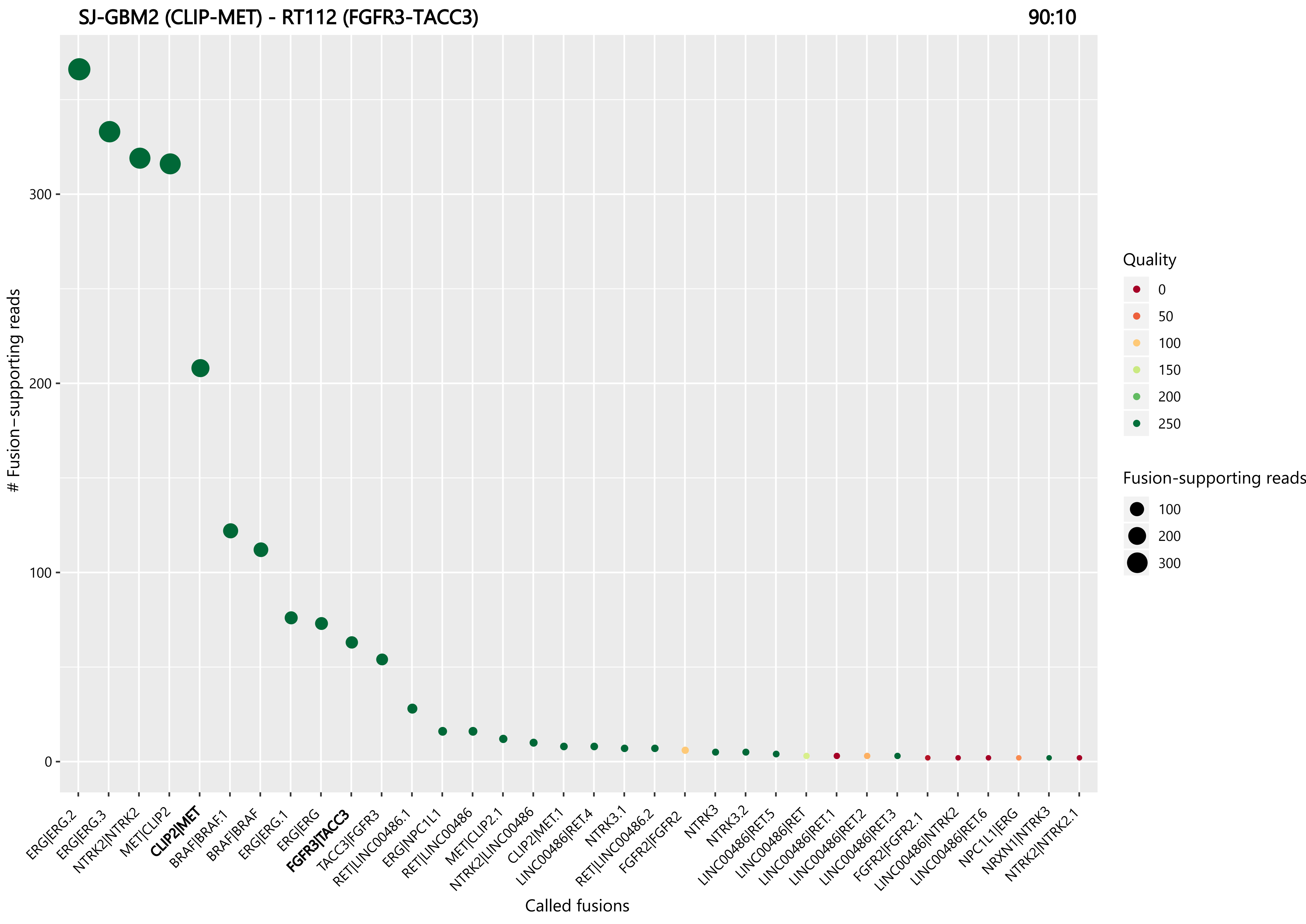
SJ-GBM2 (CLIP-MET) - RT112 (FGFR3-TACC3)
90:10
Fusion−supporting reads
300
200
100
0
Called fusions
ERG|ERG.2
ERG|ERG.3
NTRK2|NTRK2
MET|CLIP2
CLIP2|MET
BRAF|BRAF.1
BRAF|BRAF
ERG|ERG.1
ERG|ERG
FGFR3|TACC3
TACC3|FGFR3
RET|LINC00486.1
ERG|NPC1L1
RET|LINC00486
MET|CLIP2.1
NTRK2|LINC00486
CLIP2|MET.1
LINC00486|RET.4
NTRK3.1
RET|LINC00486.2
FGFR2|FGFR2
NTRK3
NTRK3.2
LINC00486|RET.5
LINC00486|RET
LINC00486|RET.1
LINC00486|RET.2
LINC00486|RET.3
FGFR2|FGFR2.1
LINC00486|NTRK2
LINC00486|RET.6
NPC1L1|ERG
NRXN1|NTRK3
NTRK2|NTRK2.1
Quality
0
50
100
150
200
250
Fusion-supporting reads
100
200
300

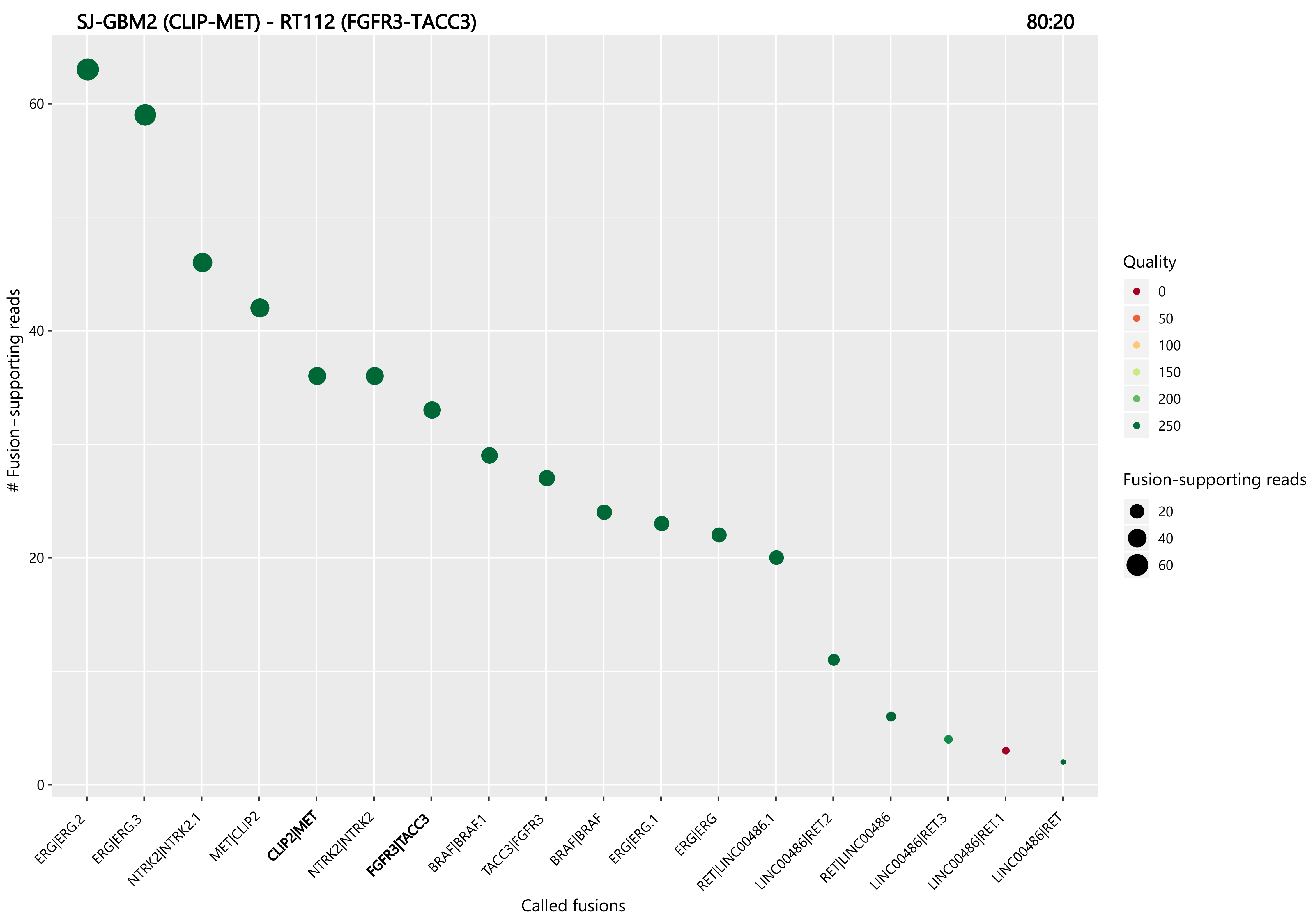

SJ-GBM2 (CLIP-MET) - RT112 (FGFR3-TACC3)
80:20
Fusion−supporting reads
60
40
20
0
ERG|ERG.2
ERG|ERG.3
NTRK2|NTRK2.1
MET|CLIP2
CLIP2|MET
NTRK2|NTRK2
FGFR3|TACC3
BRAF|BRAF.1
TACC3|FGFR3
BRAF|BRAF
ERG|ERG.1
ERG|ERG
RET|LINC00486.1
LINC00486|RET.2
RET|LINC00486
LINC00486|RET.3
LINC00486|RET.1
LINC00486|RET
Called fusions
Quality
0
50
100
150
200
250
Fusion-supporting reads
20
40
60

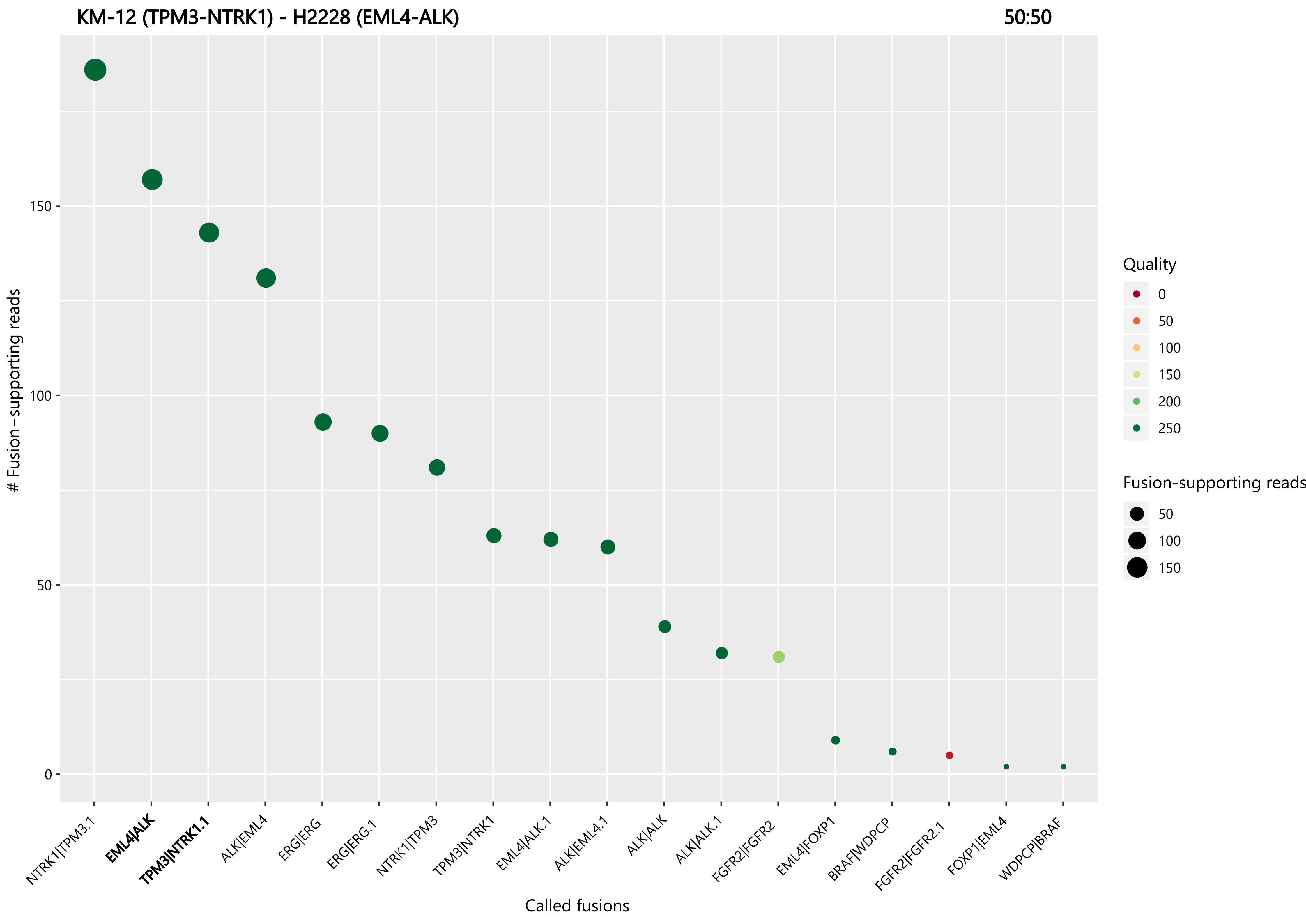

KM-12 (TPM3-NTRK1) - H2228 (EML4-ALK)
50:50
Fusion−supporting reads
150
100
50
0
Called fusions
NTRK1|TPM3.1
EML4|ALK
TPM3|NTRK1.1
ALK|EML4
ERG|ERG
ERG|ERG.1
NTRK1|TPM3
TPM3|NTRK1
EML4|ALK.1
ALK|EML4.1
ALK|ALK
ALK|ALK.1
FGFR2|FGFR2
EML4|FOXP1
BRAF|WDPCP
FGFR2|FGFR2.1
FOXP1|EML4
WDPCP|BRAF
Quality
0
50
100
150
200
250
Fusion-supporting reads
50
100
150

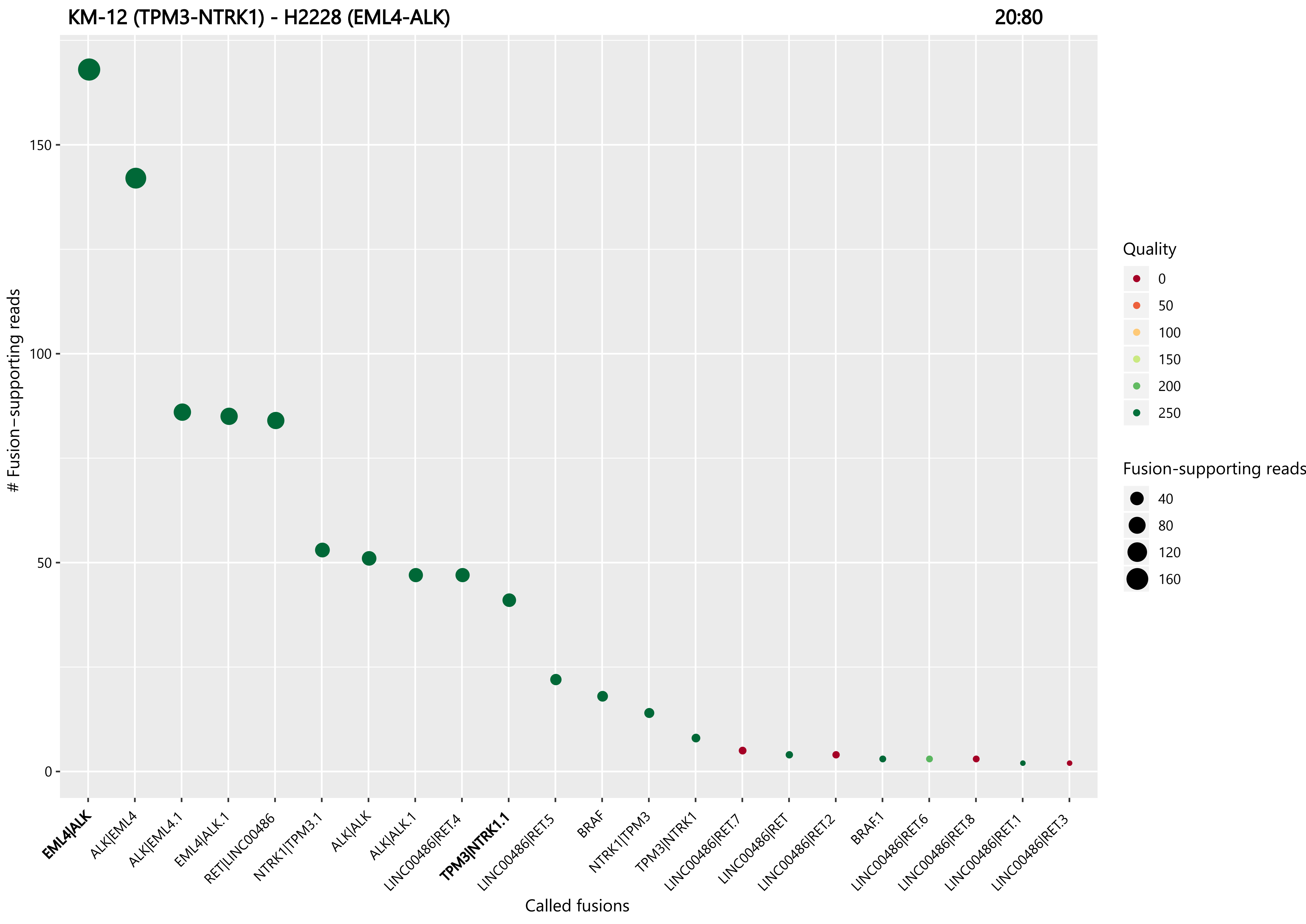

KM-12 (TPM3-NTRK1) - H2228 (EML4-ALK)
20:80
Fusion−supporting reads
150
100
50
0
Called fusions
EML4|ALK
ALK|EML4
ALK|EML4.1
EML4|ALK.1
RET|LINC00486
NTRK1|TPM3.1
ALK|ALK
ALK|ALK.1
LINC00486|RET.4
TPM3|NTRK1.1
LINC00486|RET.5
BRAF
NTRK1|TPM3
TPM3|NTRK1
LINC00486|RET.7
LINC00486|RET
LINC00486|RET.2
BRAF.1
LINC00486|RET.6
LINC00486|RET.8
LINC00486|RET.1
LINC00486|RET.3
Quality
0
50
100
150
200
250
Fusion-supporting reads
40
80
120
160

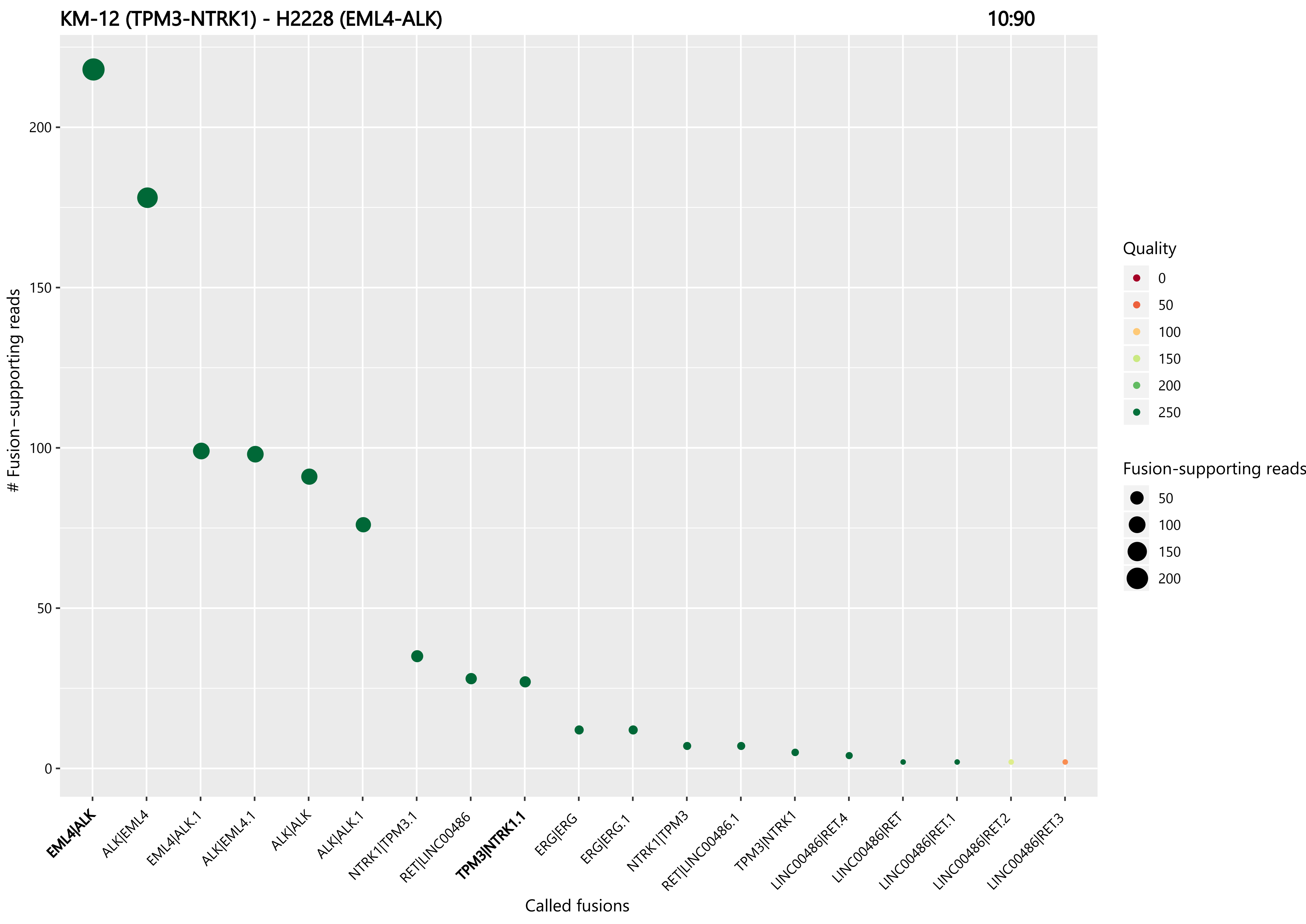
KM-12 (TPM3-NTRK1) - H2228 (EML4-ALK)
10:90
Fusion−supporting reads
200
150
100
50
0
Called fusions
EML4|ALK
ALK|EML4
EML4|ALK.1
ALK|EML4.1
ALK|ALK
ALK|ALK.1
NTRK1|TPM3.1
RET|LINC00486
TPM3|NTRK1.1
ERG|ERG
ERG|ERG.1
NTRK1|TPM3
RET|LINC00486.1
TPM3|NTRK1
LINC00486|RET.4
LINC00486|RET
LINC00486|RET.1
LINC00486|RET.2
LINC00486|RET.3
Quality
0
50
100
150
200
250
Fusion-supporting reads
50
100
150
200

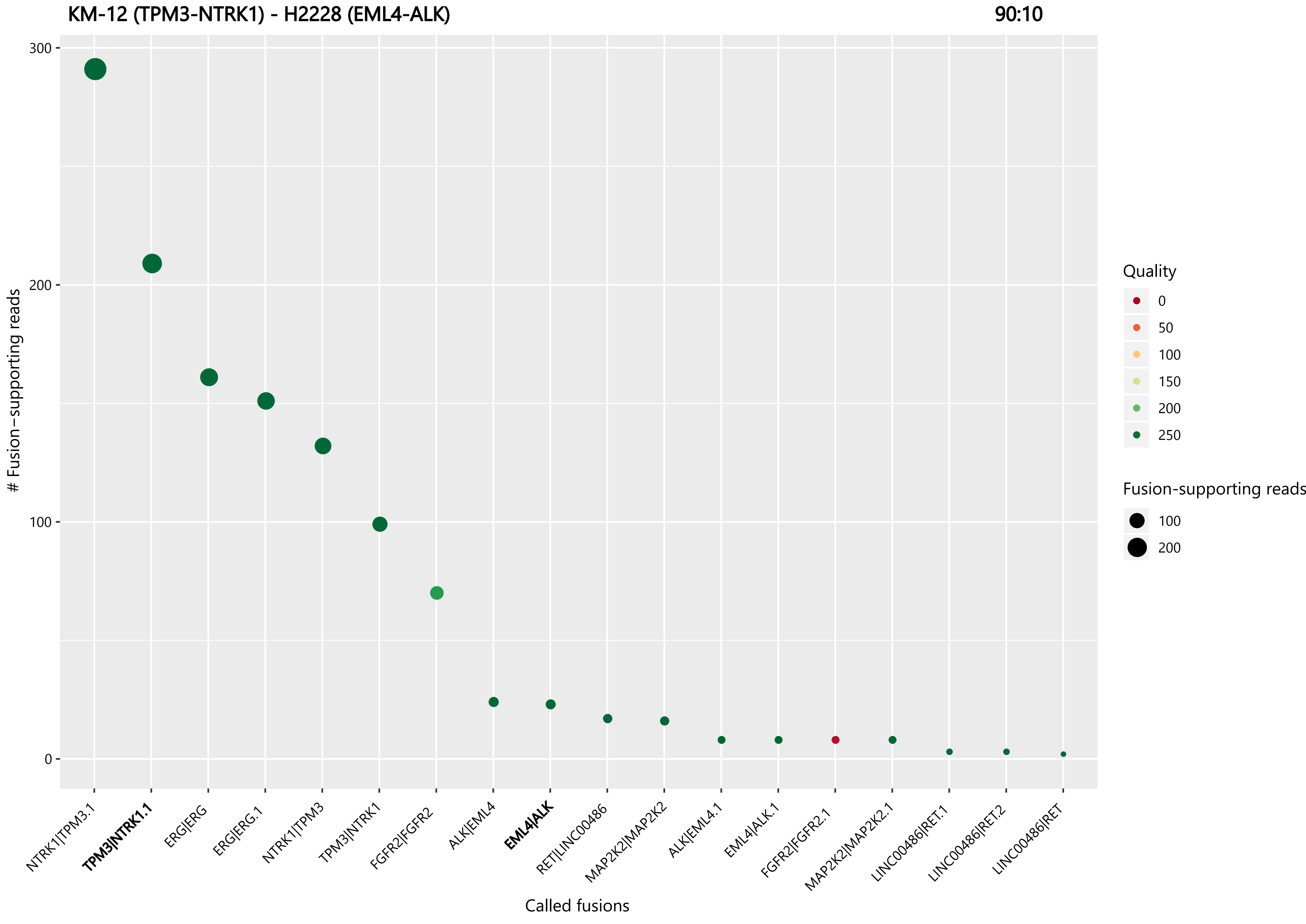

KM-12 (TPM3-NTRK1) - H2228 (EML4-ALK)
90:10
Fusion−supporting reads
300
200
100
0
Called fusions
NTRK1|TPM3.1
TPM3|NTRK1.1
ERG|ERG
ERG|ERG.1
NTRK1|TPM3
TPM3|NTRK1
FGFR2|FGFR2
ALK|EML4
EML4|ALK
RET|LINC00486
MAP2K2|MAP2K2
ALK|EML4.1
EML4|ALK.1
FGFR2|FGFR2.1
MAP2K2|MAP2K2.1
LINC00486|RET.1
LINC00486|RET.2
LINC00486|RET
Quality
0
50
100
150
200
250
Fusion-supporting reads
100
200

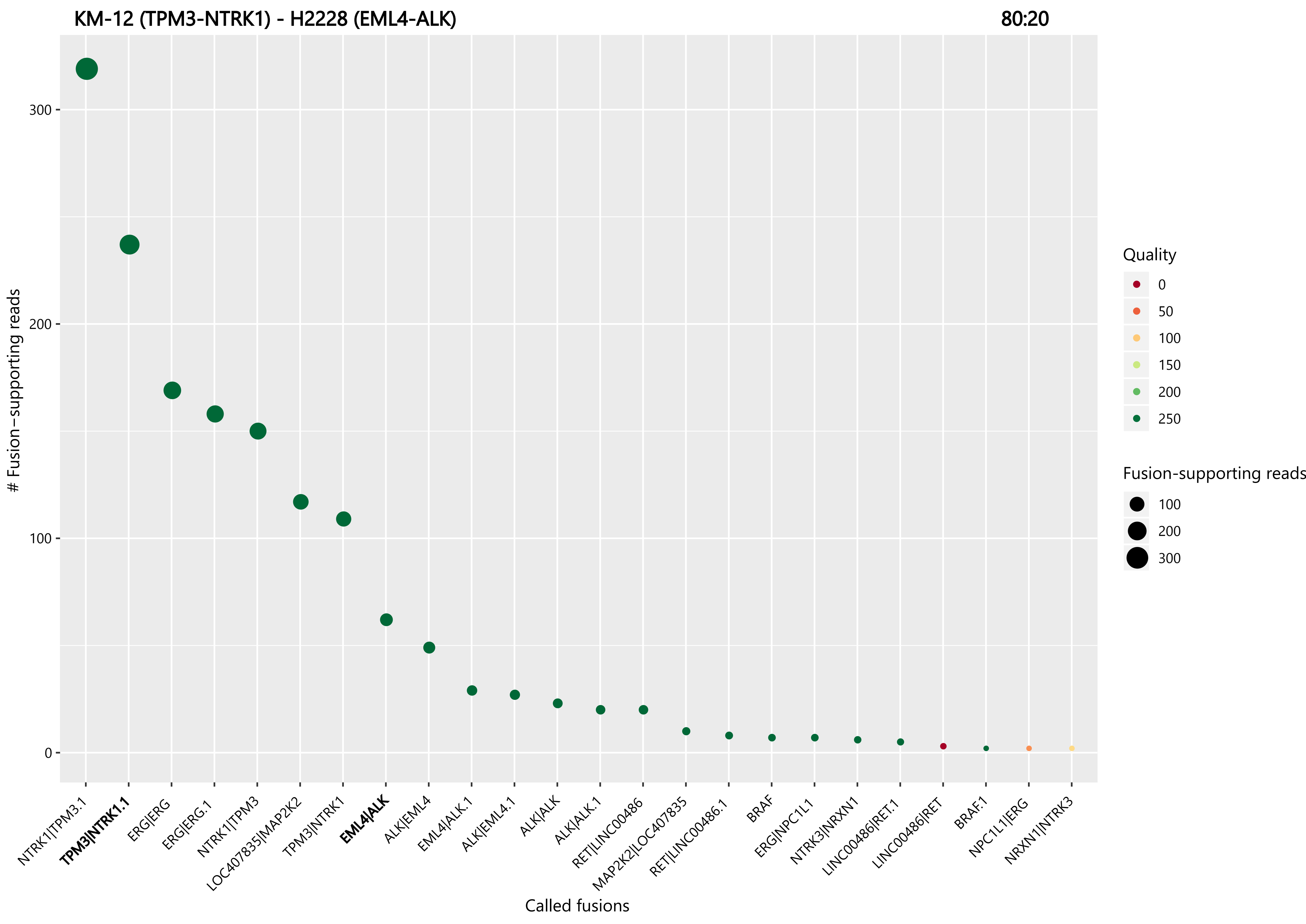
KM-12 (TPM3-NTRK1) - H2228 (EML4-ALK)
80:20
Fusion−supporting reads
300
200
100
0
NTRK1|TPM3.1
TPM3|NTRK1.1
ERG|ERG
ERG|ERG.1
NTRK1|TPM3
LOC407835|MAP2K2
TPM3|NTRK1
EML4|ALK
ALK|EML4
EML4|ALK.1
ALK|EML4.1
ALK|ALK
ALK|ALK.1
RET|LINC00486
MAP2K2|LOC407835
RET|LINC00486.1
BRAF
ERG|NPC1L1
NTRK3|NRXN1
LINC00486|RET.1
LINC00486|RET
BRAF.1
NPC1L1|ERG
NRXN1|NTRK3
Called fusions
Quality
0
50
100
150
200
250
Fusion-supporting reads
100
200
300

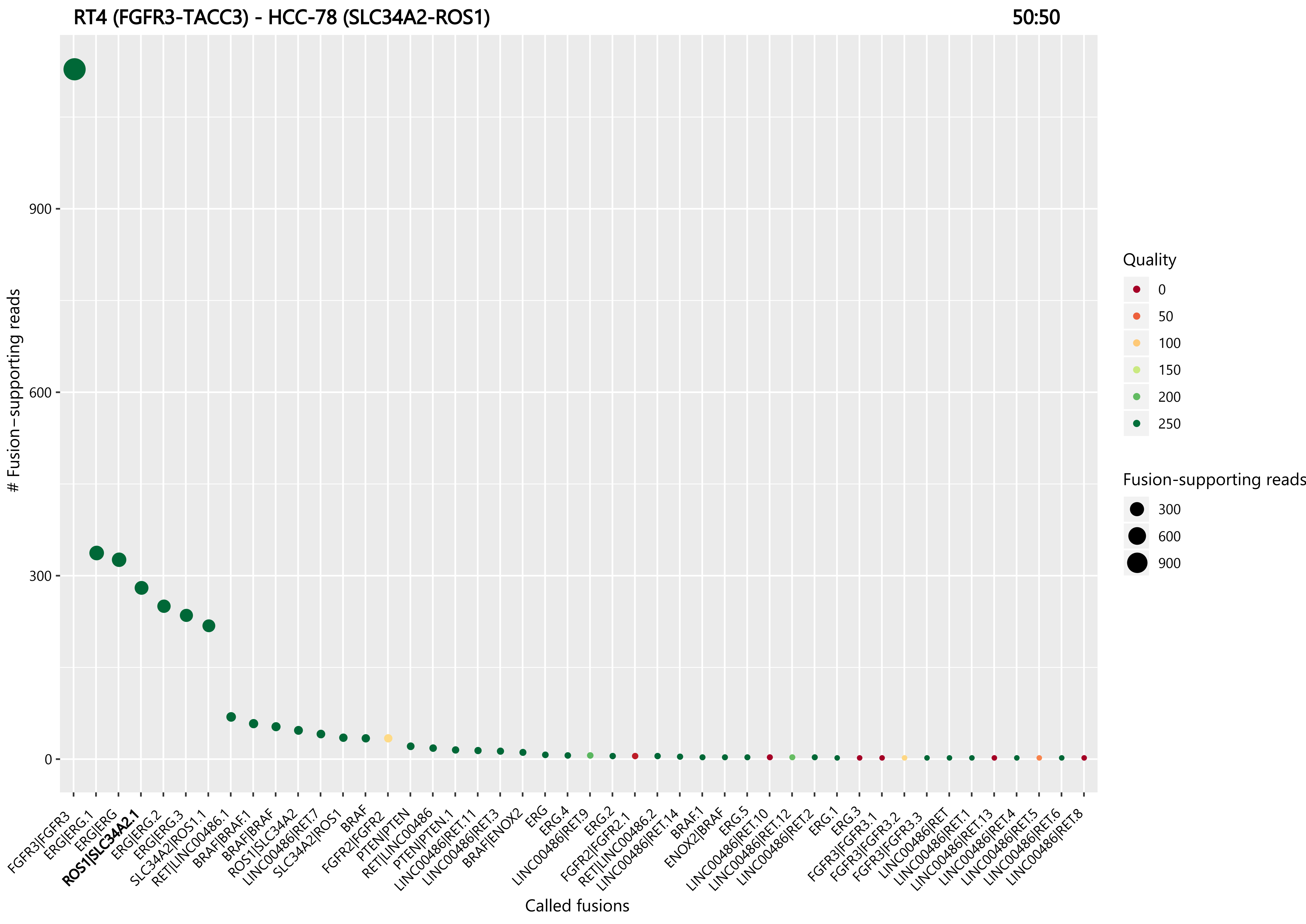

RT4 (FGFR3-TACC3) - HCC-78 (SLC34A2-ROS1)
50:50
Fusion−supporting reads
900
600
300
0
Called fusions
FGFR3|FGFR3
ERG|ERG.1
ERG|ERG
ROS1|SLC34A2.1
ERG|ERG.2
ERG|ERG.3
SLC34A2|ROS1.1
RET|LINC00486.1
BRAF|BRAF.1
BRAF|BRAF
ROS1|SLC34A2
LINC00486|RET.7
SLC34A2|ROS1
BRAF
FGFR2|FGFR2
PTEN|PTEN
RET|LINC00486
PTEN|PTEN.1
LINC00486|RET.11
LINC00486|RET.3
BRAF|ENOX2
ERG
ERG.4
LINC00486|RET.9
ERG.2
FGFR2|FGFR2.1
RET|LINC00486.2
LINC00486|RET.14
BRAF.1
ENOX2|BRAF
ERG.5
LINC00486|RET.10
LINC00486|RET.12
LINC00486|RET.2
ERG.1
ERG.3
FGFR3|FGFR3.1
FGFR3|FGFR3.2
FGFR3|FGFR3.3
LINC00486|RET
LINC00486|RET.1
LINC00486|RET.13
LINC00486|RET.4
LINC00486|RET.5
LINC00486|RET.6
LINC00486|RET.8
Quality
0
50
100
150
200
250
Fusion-supporting reads
300
600
900

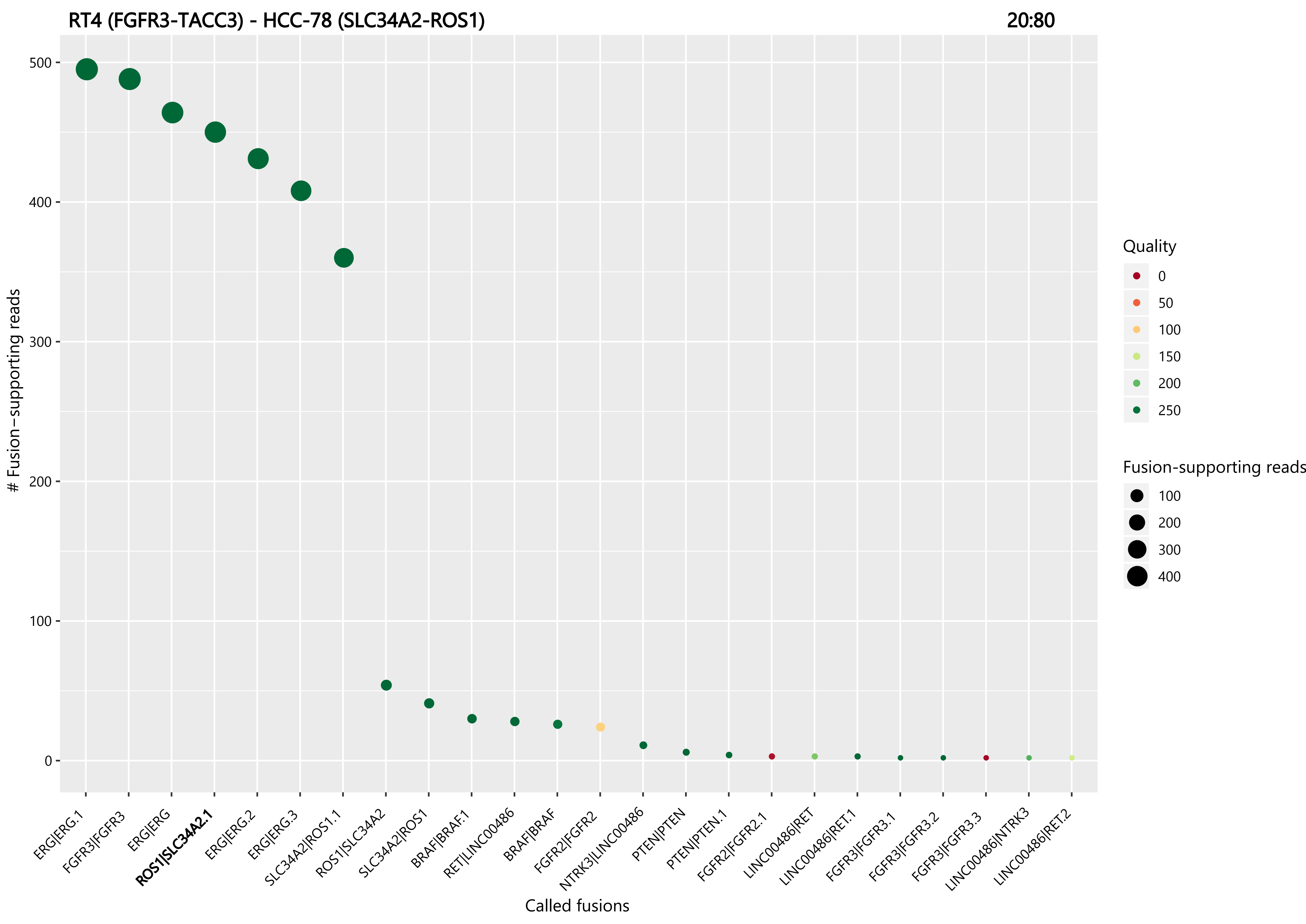

RT4 (FGFR3-TACC3) - HCC-78 (SLC34A2-ROS1)
20:80
Fusion−supporting reads
500
400
300
200
100
0
Called fusions
ERG|ERG.1
FGFR3|FGFR3
ERG|ERG
ROS1|SLC34A2.1
ERG|ERG.2
ERG|ERG.3
SLC34A2|ROS1.1
ROS1|SLC34A2
SLC34A2|ROS1
BRAF|BRAF.1
RET|LINC00486
BRAF|BRAF
FGFR2|FGFR2
NTRK3|LINC00486
PTEN|PTEN
PTEN|PTEN.1
FGFR2|FGFR2.1
LINC00486|RET
LINC00486|RET.1
FGFR3|FGFR3.1
FGFR3|FGFR3.2
FGFR3|FGFR3.3
LINC00486|NTRK3
LINC00486|RET.2
Quality
0
50
100
150
200
250
Fusion-supporting reads
100
200
300
400

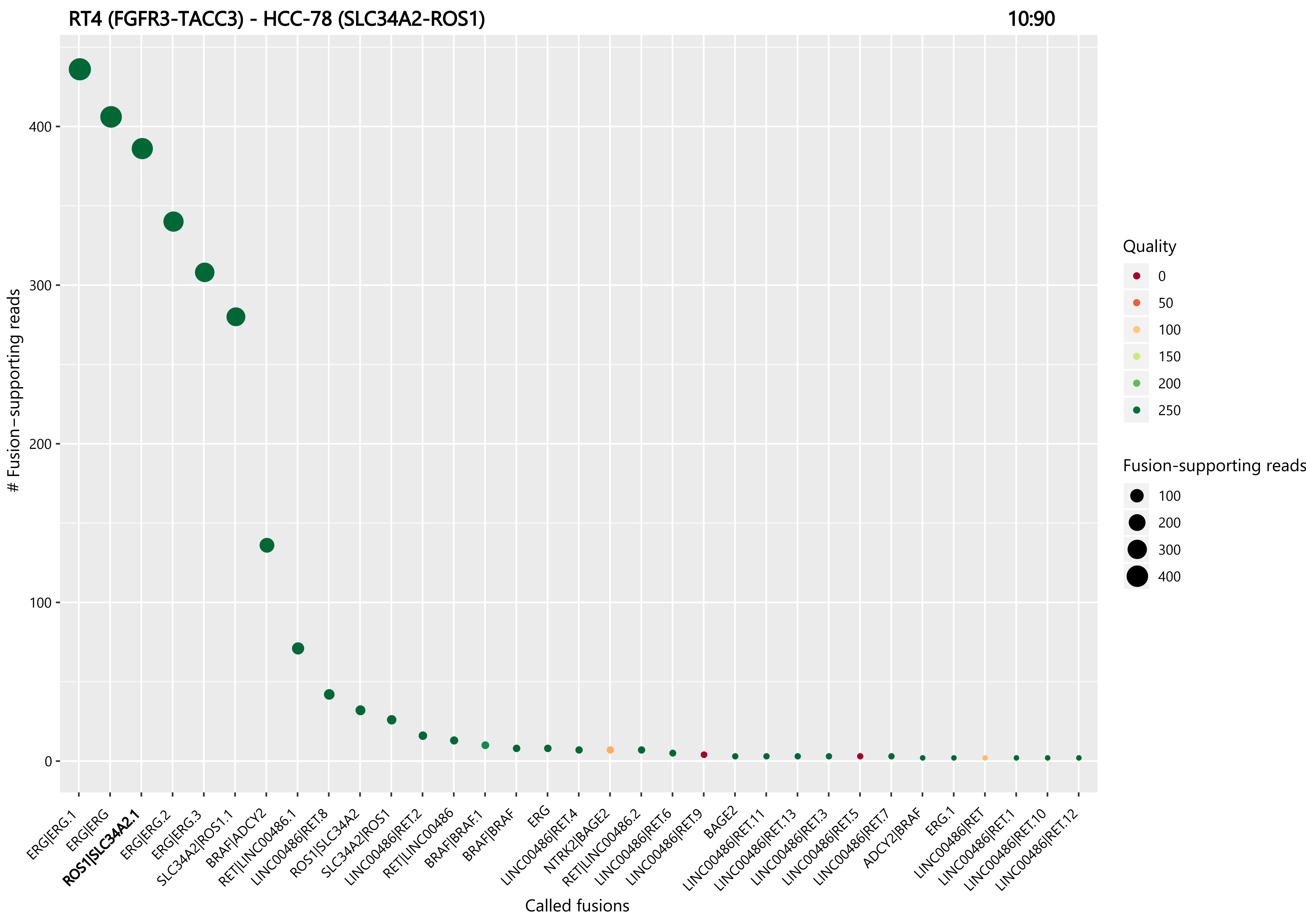

RT4 (FGFR3-TACC3) - HCC-78 (SLC34A2-ROS1)
10:90
Fusion−supporting reads
0
100
200
300
400
Called fusions
ERG|ERG.1
ERG|ERG
ROS1|SLC34A2.1
ERG|ERG.2
ERG|ERG.3
SLC34A2|ROS1.1
BRAF|ADCY2
RET|LINC00486.1
LINC00486|RET.8
ROS1|SLC34A2
SLC34A2|ROS1
LINC00486|RET.2
RET|LINC00486
BRAF|BRAF.1
BRAF|BRAF
ERG
LINC00486|RET.4
NTRK2|BAGE2
RET|LINC00486.2
LINC00486|RET.6
LINC00486|RET.9
BAGE2
LINC00486|RET.11
LINC00486|RET.13
LINC00486|RET.3
LINC00486|RET.5
LINC00486|RET.7
ADCY2|BRAF
ERG.1
LINC00486|RET
LINC00486|RET.1
LINC00486|RET.10
LINC00486|RET.12
Quality
0
50
100
150
200
250
Fusion-supporting reads
100
200
300
400

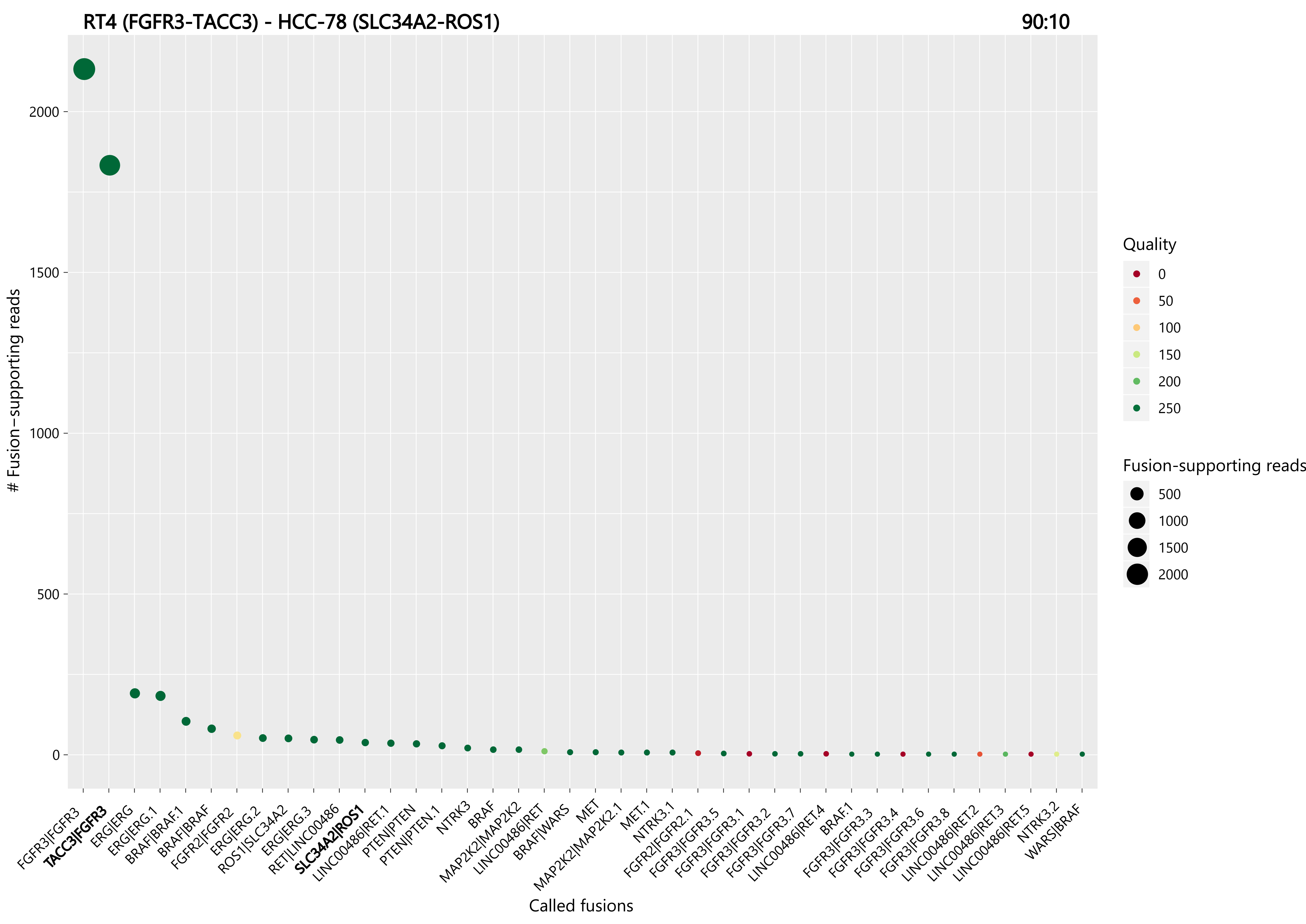
RT4 (FGFR3-TACC3) - HCC-78 (SLC34A2-ROS1)
90:10
Fusion−supporting reads
2000
1500
1000
500
0
Called fusions
FGFR3|FGFR3
TACC3|FGFR3
ERG|ERG
ERG|ERG.1
BRAF|BRAF.1
BRAF|BRAF
FGFR2|FGFR2
ERG|ERG.2
ROS1|SLC34A2
ERG|ERG.3
RET|LINC00486
SLC34A2|ROS1
LINC00486|RET.1
PTEN|PTEN
PTEN|PTEN.1
NTRK3
BRAF
MAP2K2|MAP2K2
LINC00486|RET
BRAF|WARS
MET
MAP2K2|MAP2K2.1
MET.1
NTRK3.1
FGFR2|FGFR2.1
FGFR3|FGFR3.5
FGFR3|FGFR3.1
FGFR3|FGFR3.2
FGFR3|FGFR3.7
LINC00486|RET.4
BRAF.1
FGFR3|FGFR3.3
FGFR3|FGFR3.4
FGFR3|FGFR3.6
FGFR3|FGFR3.8
LINC00486|RET.2
LINC00486|RET.3
LINC00486|RET.5
NTRK3.2
WARS|BRAF
Quality
0
50
100
150
200
250
Fusion-supporting reads
500
1000
1500
2000

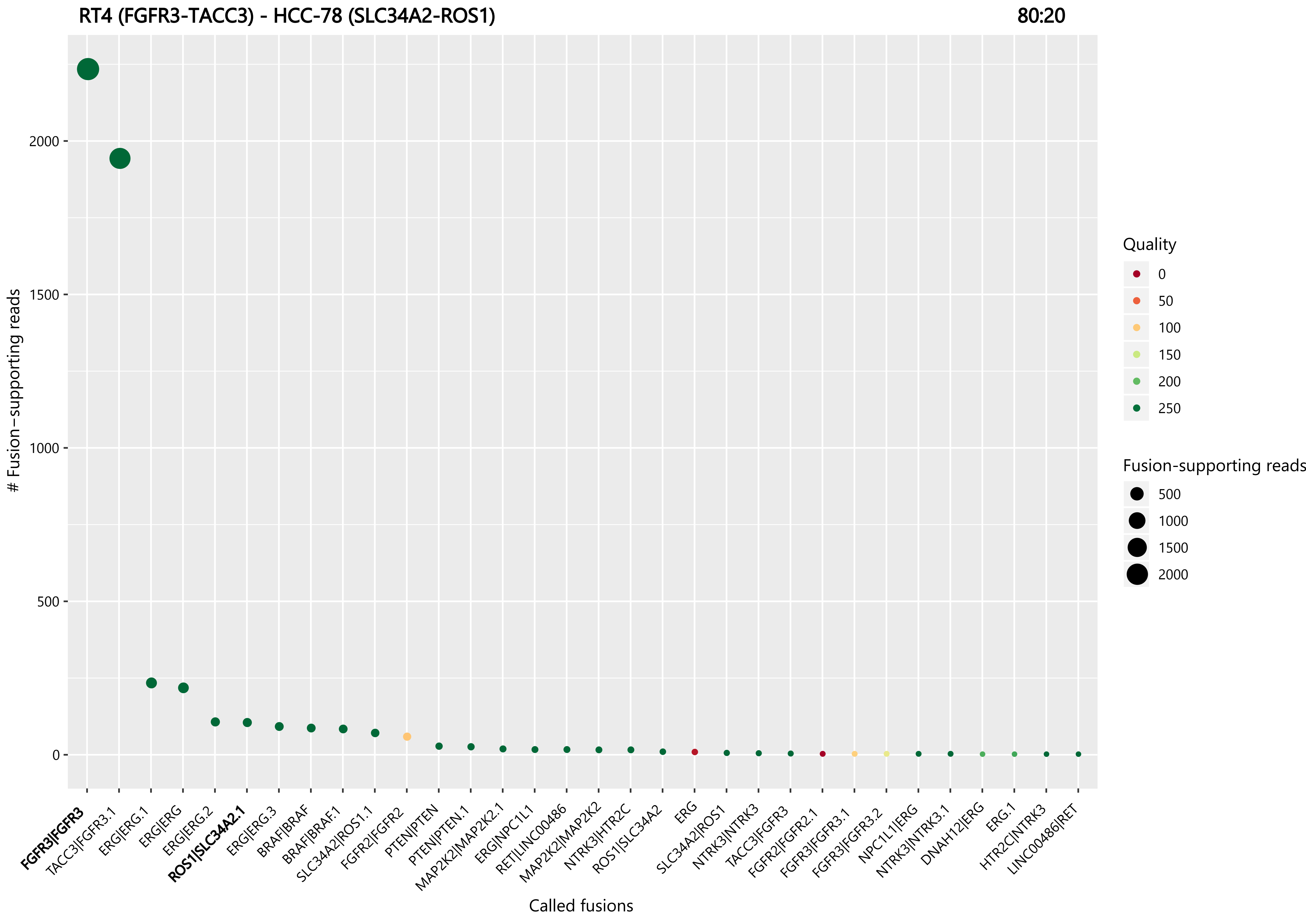

RT4 (FGFR3-TACC3) - HCC-78 (SLC34A2-ROS1)
80:20
Fusion−supporting reads
0
500
1000
1500
2000
Called fusions
FGFR3|FGFR3
TACC3|FGFR3.1
ERG|ERG.1
ERG|ERG
ERG|ERG.2
ROS1|SLC34A2.1
ERG|ERG.3
BRAF|BRAF
BRAF|BRAF.1
SLC34A2|ROS1.1
FGFR2|FGFR2
PTEN|PTEN
PTEN|PTEN.1
MAP2K2|MAP2K2.1
ERG|NPC1L1
RET|LINC00486
MAP2K2|MAP2K2
NTRK3|HTR2C
ROS1|SLC34A2
ERG
SLC34A2|ROS1
NTRK3|NTRK3
TACC3|FGFR3
FGFR2|FGFR2.1
FGFR3|FGFR3.1
FGFR3|FGFR3.2
NPC1L1|ERG
NTRK3|NTRK3.1
DNAH12|ERG
ERG.1
HTR2C|NTRK3
LINC00486|RET
Quality
0
50
100
150
200
250
Fusion-supporting reads
500
1000
1500
2000

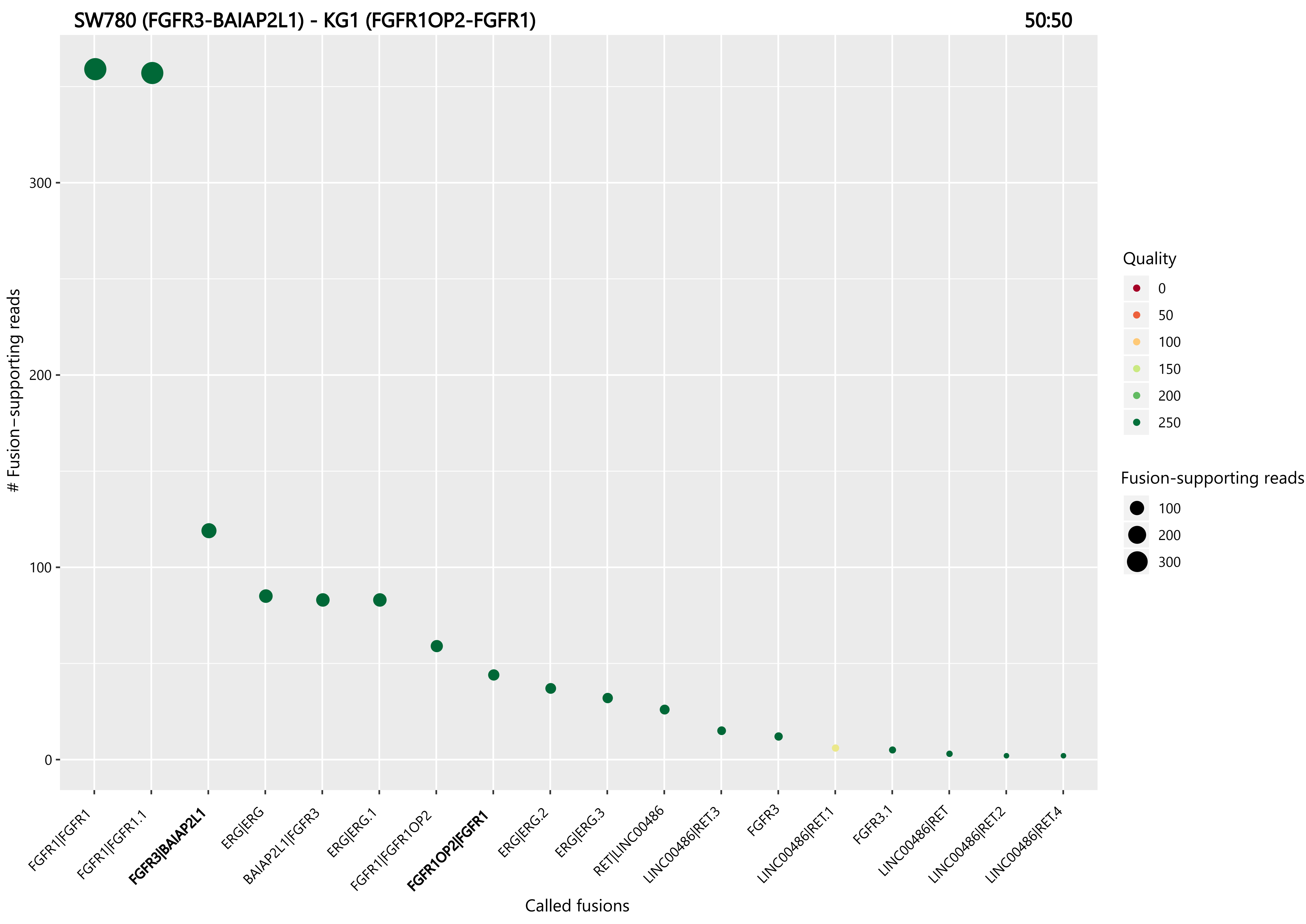

SW780 (FGFR3-BAIAP2L1) - KG1 (FGFR1OP2-FGFR1)
50:50
Fusion−supporting reads
300
200
100
0
Called fusions
FGFR1|FGFR1
FGFR1|FGFR1.1
FGFR3|BAIAP2L1
ERG|ERG
BAIAP2L1|FGFR3
ERG|ERG.1
FGFR1|FGFR1OP2
FGFR1OP2|FGFR1
ERG|ERG.2
ERG|ERG.3
RET|LINC00486
LINC00486|RET.3
FGFR3
LINC00486|RET.1
FGFR3.1
LINC00486|RET
LINC00486|RET.2
LINC00486|RET.4
Quality
0
50
100
150
200
250
Fusion-supporting reads
100
200
300

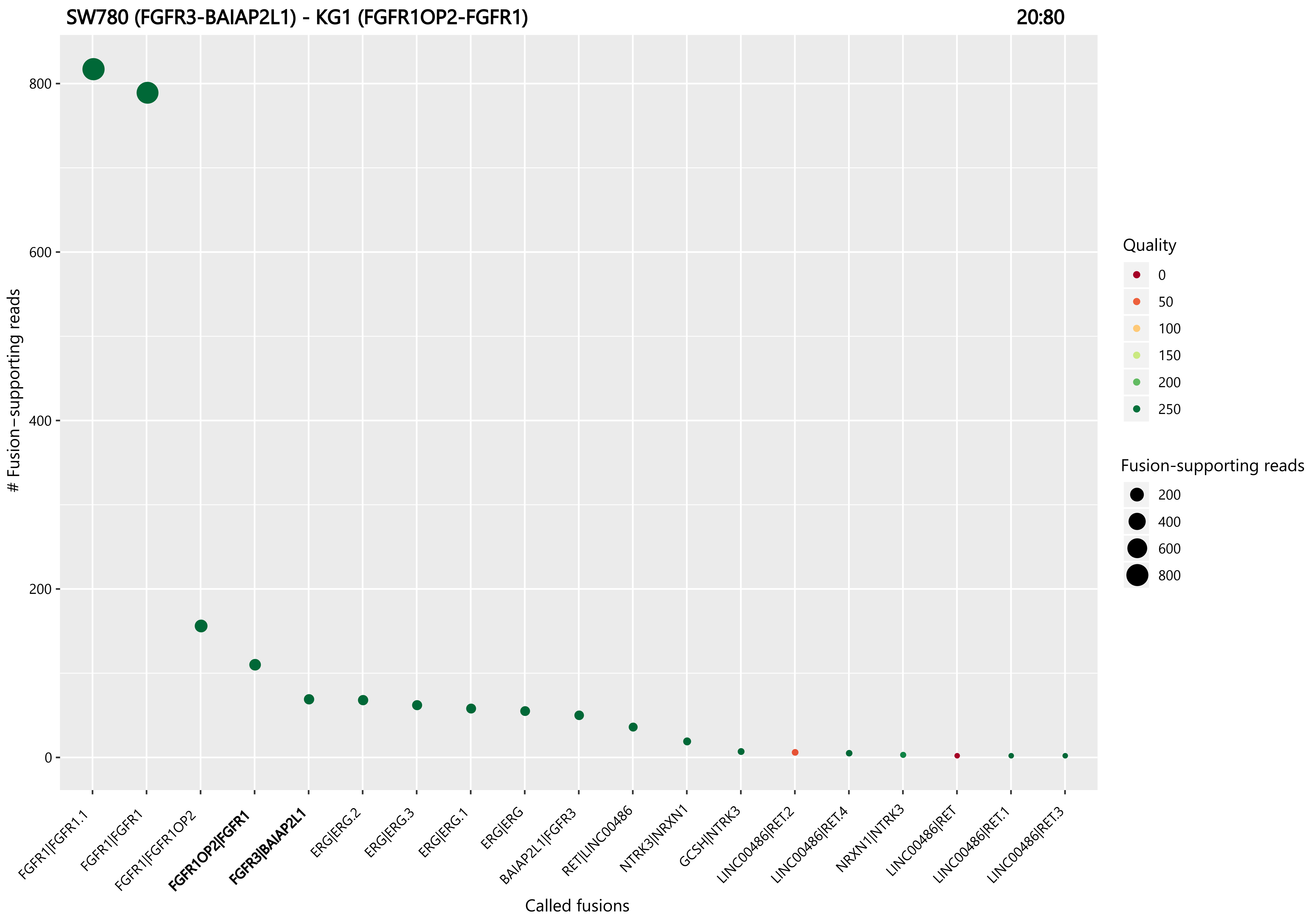

SW780 (FGFR3-BAIAP2L1) - KG1 (FGFR1OP2-FGFR1)
20:80
Fusion−supporting reads
800
600
400
200
0
Called fusions
FGFR1|FGFR1.1
FGFR1|FGFR1
FGFR1|FGFR1OP2
FGFR1OP2|FGFR1
FGFR3|BAIAP2L1
ERG|ERG.2
ERG|ERG.3
ERG|ERG.1
ERG|ERG
BAIAP2L1|FGFR3
RET|LINC00486
NTRK3|NRXN1
GCSH|NTRK3
LINC00486|RET.2
LINC00486|RET.4
NRXN1|NTRK3
LINC00486|RET
LINC00486|RET.1
LINC00486|RET.3
Quality
0
50
100
150
200
250
Fusion-supporting reads
200
400
600
800

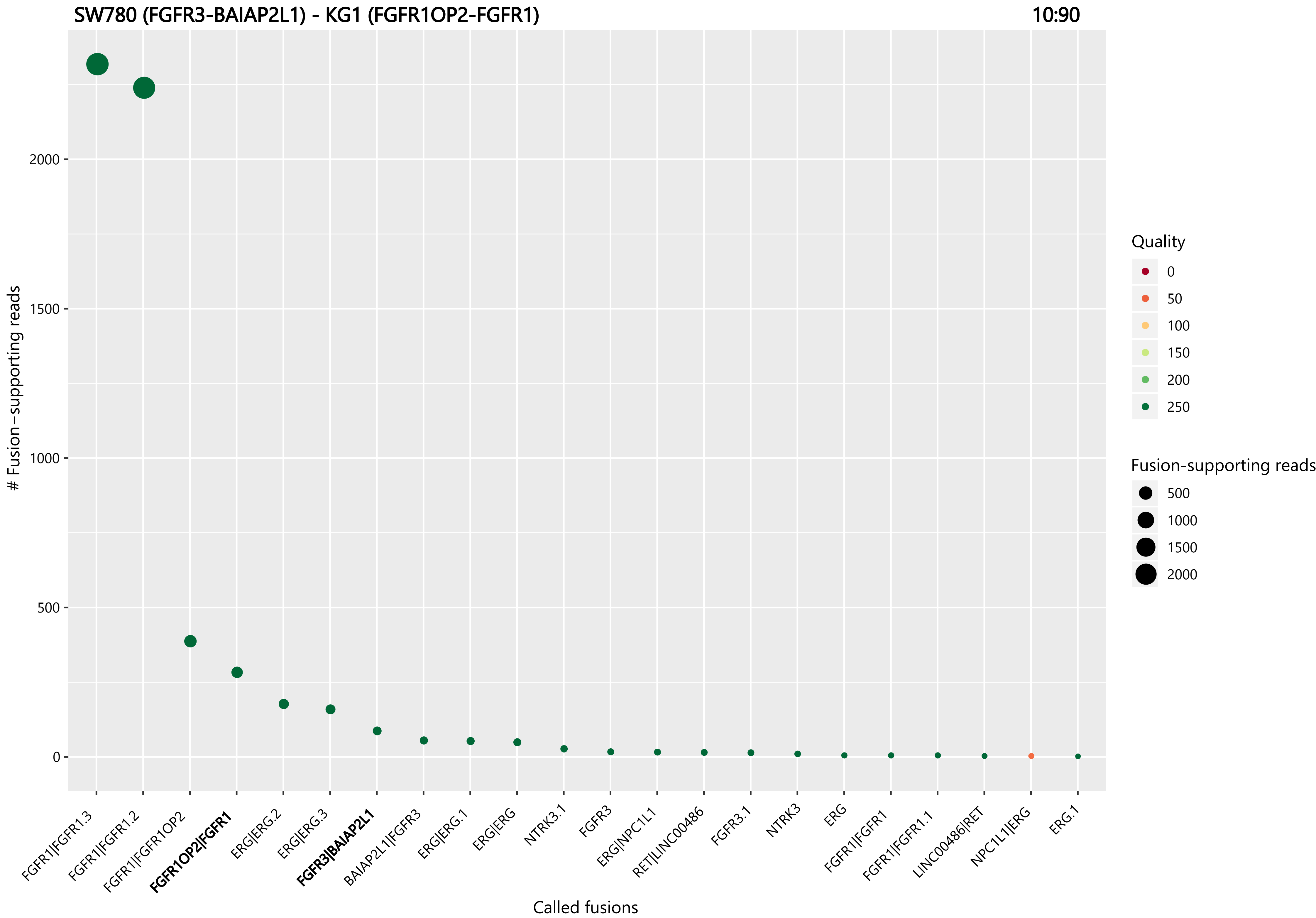

SW780 (FGFR3-BAIAP2L1) - KG1 (FGFR1OP2-FGFR1)
10:90
Fusion−supporting reads
2000
1500
1000
500
0
Called fusions
FGFR1|FGFR1.3
FGFR1|FGFR1.2
FGFR1|FGFR1OP2
FGFR1OP2|FGFR1
ERG|ERG.2
ERG|ERG.3
FGFR3|BAIAP2L1
BAIAP2L1|FGFR3
ERG|ERG.1
ERG|ERG
NTRK3.1
FGFR3
ERG|NPC1L1
RET|LINC00486
FGFR3.1
NTRK3
ERG
FGFR1|FGFR1
FGFR1|FGFR1.1
LINC00486|RET
NPC1L1|ERG
ERG.1
Quality
0
50
100
150
200
250
Fusion-supporting reads
500
1000
1500
2000

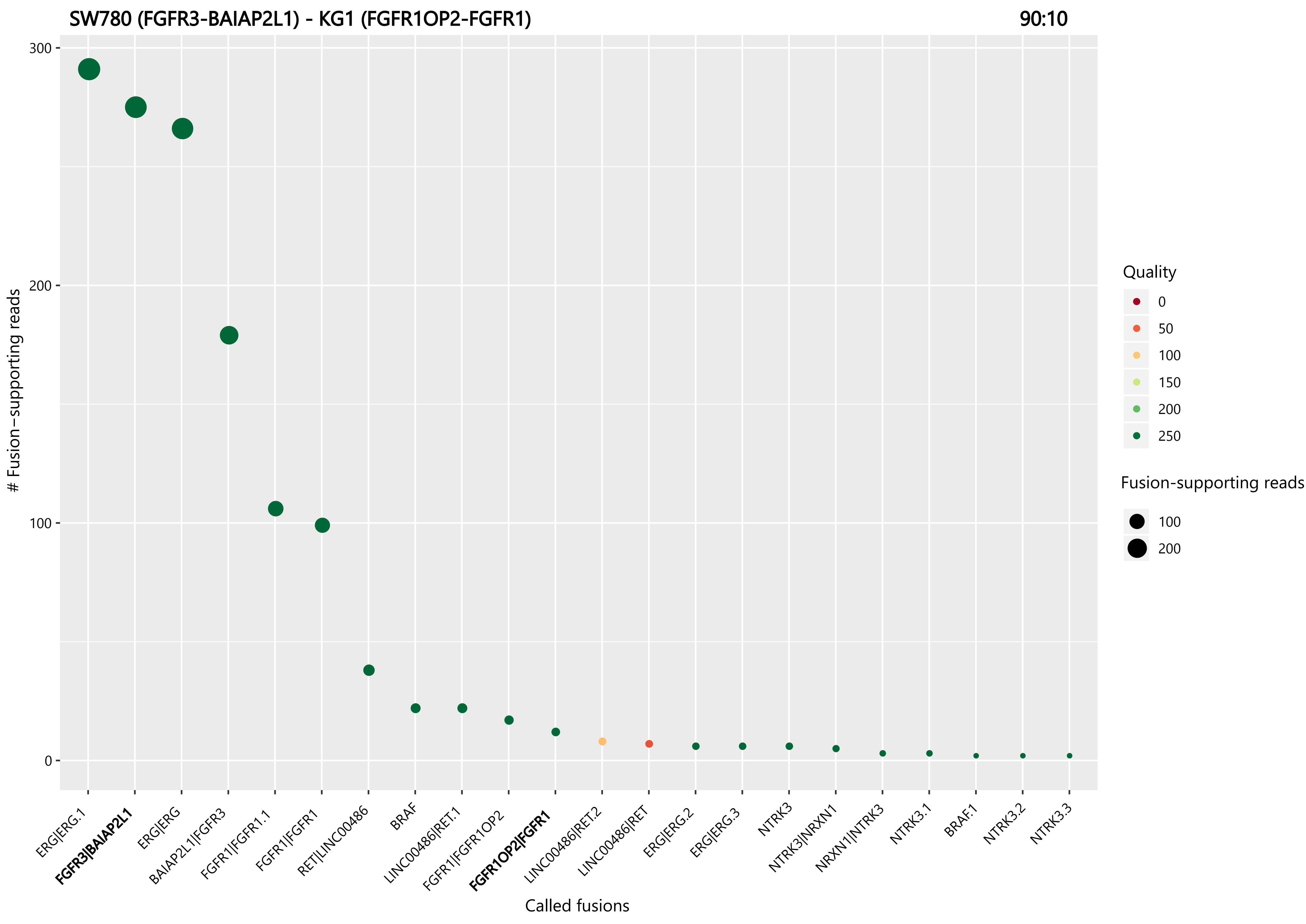

SW780 (FGFR3-BAIAP2L1) - KG1 (FGFR1OP2-FGFR1)
90:10
Fusion−supporting reads
300
200
100
0
Called fusions
ERG|ERG.1
FGFR3|BAIAP2L1
ERG|ERG
BAIAP2L1|FGFR3
FGFR1|FGFR1.1
FGFR1|FGFR1
RET|LINC00486
BRAF
LINC00486|RET.1
FGFR1|FGFR1OP2
FGFR1OP2|FGFR1
LINC00486|RET.2
LINC00486|RET
ERG|ERG.2
ERG|ERG.3
NTRK3
NTRK3|NRXN1
NRXN1|NTRK3
NTRK3.1
BRAF.1
NTRK3.2
NTRK3.3
Quality
0
50
100
150
200
250
Fusion-supporting reads
100
200

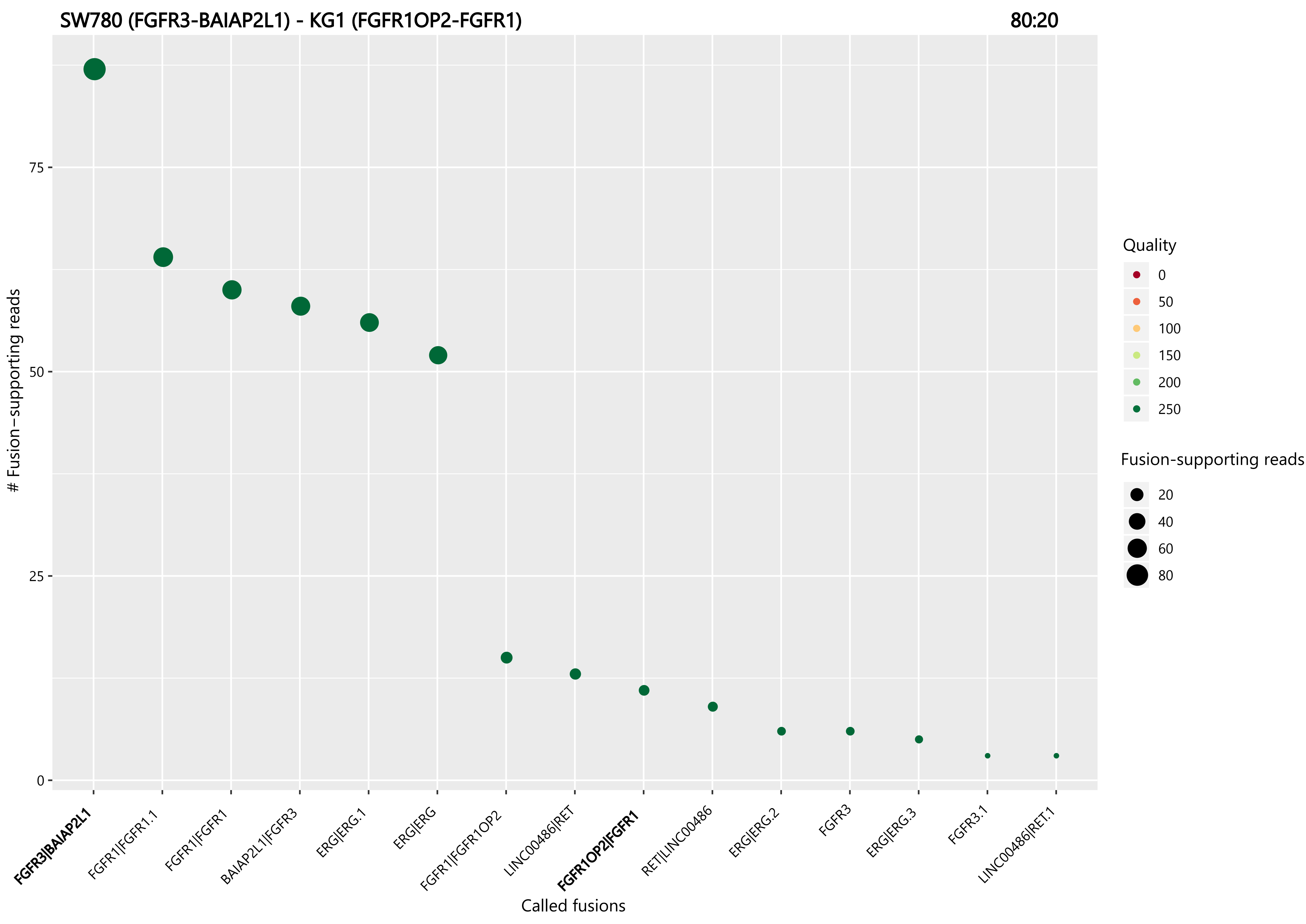
SW780 (FGFR3-BAIAP2L1) - KG1 (FGFR1OP2-FGFR1)
80:20
Fusion−supporting reads
0
25
50
75
Called fusions
FGFR3|BAIAP2L1
FGFR1|FGFR1.1
FGFR1|FGFR1
BAIAP2L1|FGFR3
ERG|ERG.1
ERG|ERG
FGFR1|FGFR1OP2
LINC00486|RET
FGFR1OP2|FGFR1
RET|LINC00486
ERG|ERG.2
FGFR3
ERG|ERG.3
FGFR3.1
LINC00486|RET.1
Quality
0
50
100
150
200
250
Fusion-supporting reads
20
40
60
80

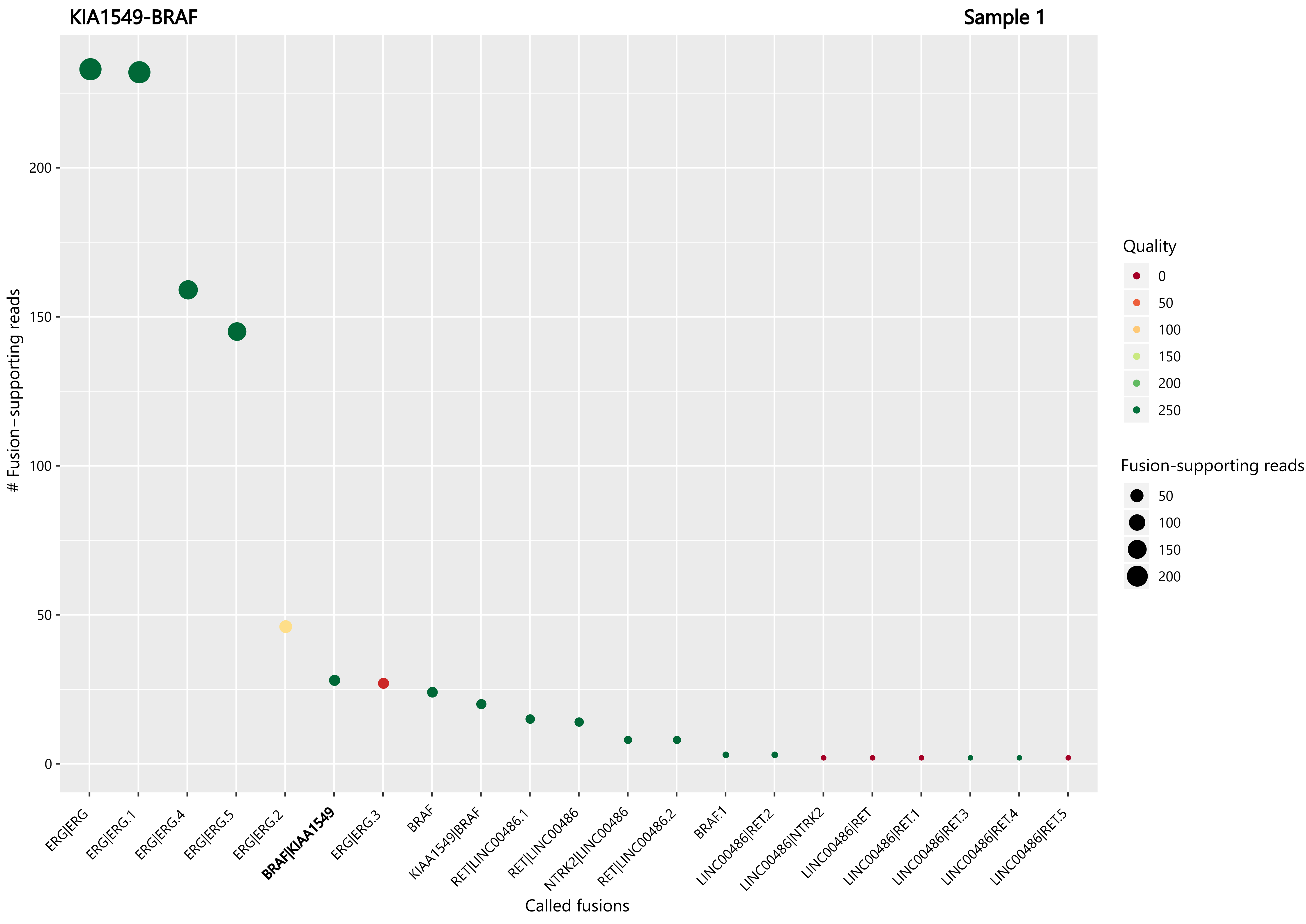

KIA1549-BRAF
Sample 1
Fusion−supporting reads
200
150
100
50
0
ERG|ERG
ERG|ERG.1
ERG|ERG.4
ERG|ERG.5
ERG|ERG.2
BRAF|KIAA1549
ERG|ERG.3
BRAF
KIAA1549|BRAF
RET|LINC00486.1
RET|LINC00486
NTRK2|LINC00486
RET|LINC00486.2
BRAF.1
LINC00486|RET.2
LINC00486|NTRK2
LINC00486|RET
LINC00486|RET.1
LINC00486|RET.3
LINC00486|RET.4
LINC00486|RET.5
Called fusions
Quality
0
50
100
150
200
250
Fusion-supporting reads
50
100
150
200

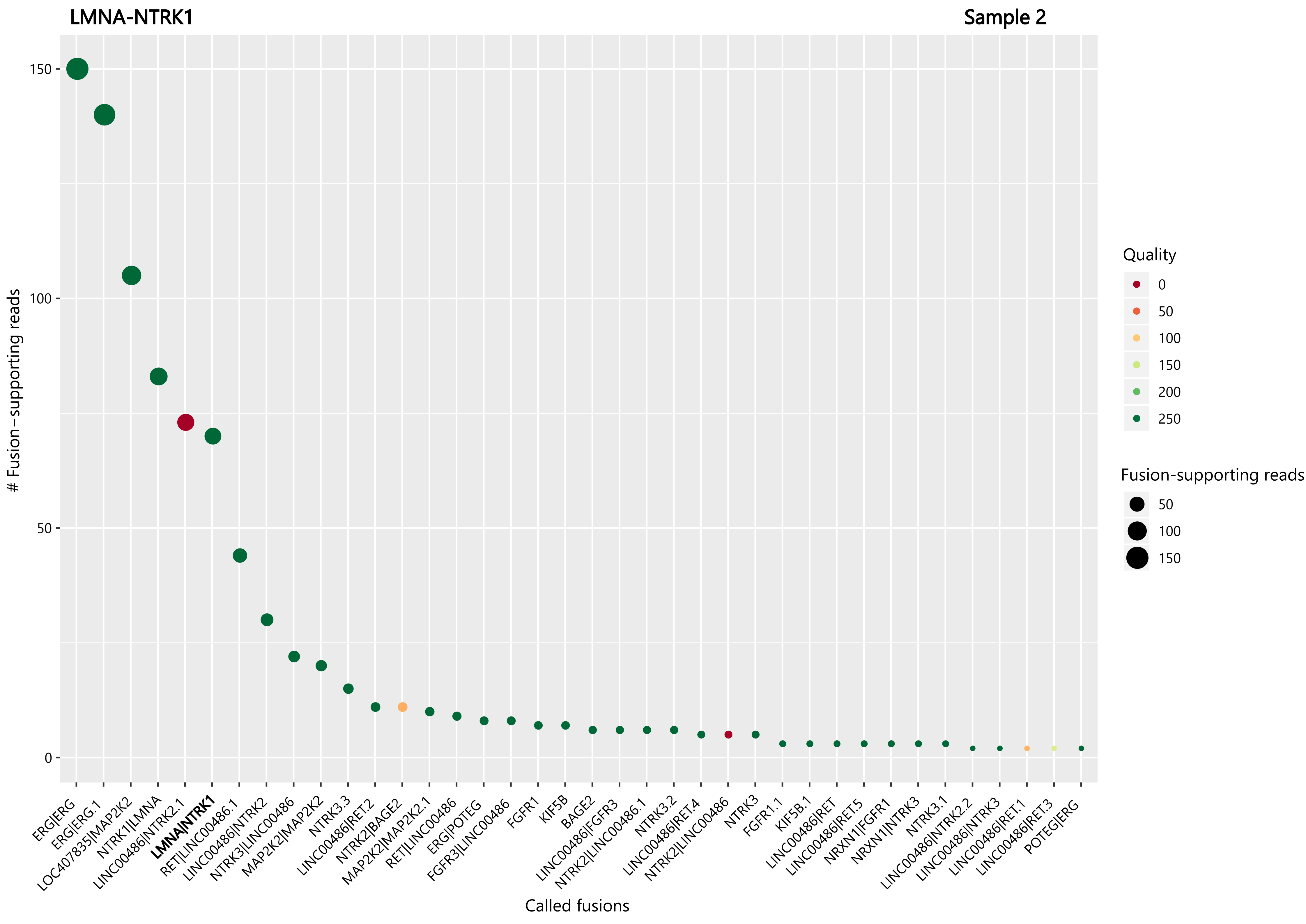
LMNA-NTRK1
Sample 2
Fusion−supporting reads
150
100
50
0
Called fusions
ERG|ERG
ERG|ERG.1
LOC407835|MAP2K2
NTRK1|LMNA
LINC00486|NTRK2.1
LMNA|NTRK1
RET|LINC00486.1
LINC00486|NTRK2
NTRK3|LINC00486
MAP2K2|MAP2K2
NTRK3.3
LINC00486|RET.2
NTRK2|BAGE2
MAP2K2|MAP2K2.1
RET|LINC00486
ERG|POTEG
FGFR3|LINC00486
FGFR1
KIF5B
BAGE2
LINC00486|FGFR3
NTRK2|LINC00486.1
NTRK3.2
LINC00486|RET.4
NTRK2|LINC00486
NTRK3
FGFR1.1
KIF5B.1
LINC00486|RET
LINC00486|RET.5
NRXN1|FGFR1
NRXN1|NTRK3
NTRK3.1
LINC00486|NTRK2.2
LINC00486|NTRK3
LINC00486|RET.1
LINC00486|RET.3
POTEG|ERG
Quality
0
50
100
150
200
250
Fusion-supporting reads
50
100
150

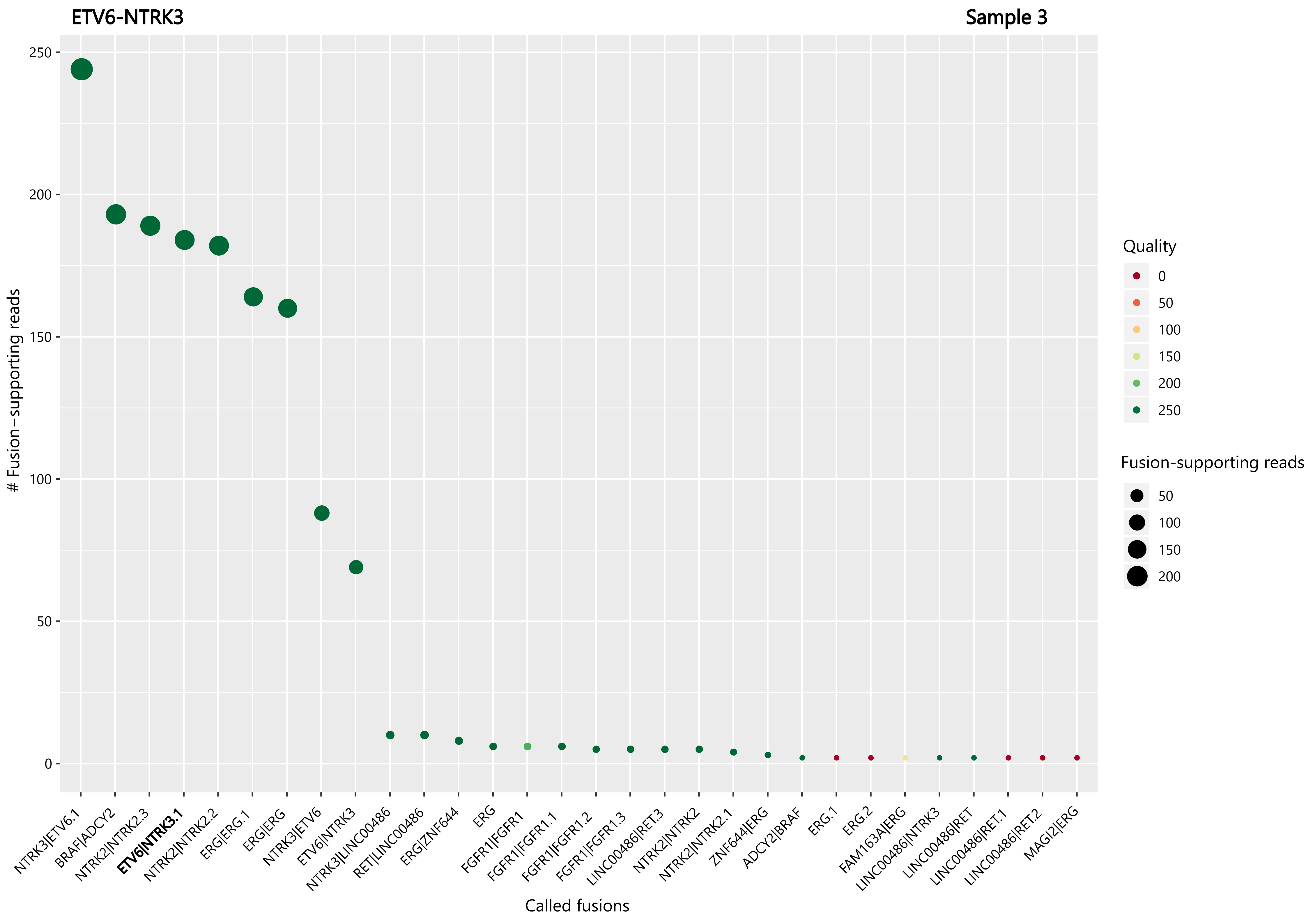
ETV6-NTRK3
Sample 3
Fusion−supporting reads
250
200
150
100
50
0
NTRK3|ETV6.1
BRAF|ADCY2
NTRK2|NTRK2.3
ETV6|NTRK3.1
NTRK2|NTRK2.2
ERG|ERG.1
ERG|ERG
NTRK3|ETV6
ETV6|NTRK3
NTRK3|LINC00486
RET|LINC00486
ERG|ZNF644
ERG
FGFR1|FGFR1
FGFR1|FGFR1.1
FGFR1|FGFR1.2
FGFR1|FGFR1.3
LINC00486|RET.3
NTRK2|NTRK2
NTRK2|NTRK2.1
ZNF644|ERG
ADCY2|BRAF
ERG.1
ERG.2
FAM163A|ERG
LINC00486|NTRK3
LINC00486|RET
LINC00486|RET.1
LINC00486|RET.2
MAGI2|ERG
Called fusions
Quality
0
50
100
150
200
250
Fusion-supporting reads
50
100
150
200

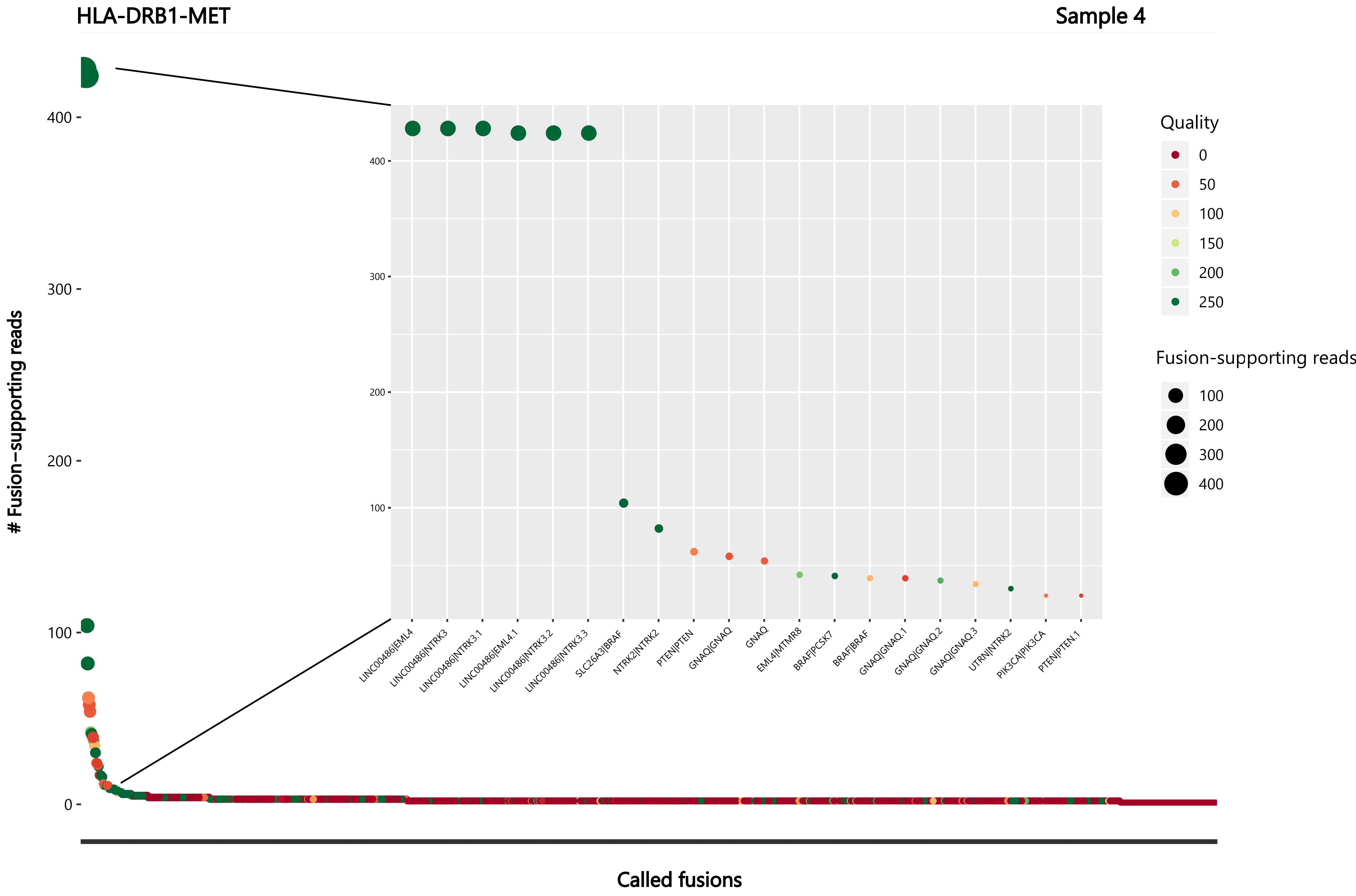
HLA-DRB1-MET
Sample 4
Fusion−supporting reads
Called fusions
400
300
200
100
0
LINC00486|EML4
LINC00486|NTRK3
LINC00486|NTRK3.1
LINC00486|EML4.1
LINC00486|NTRK3.2
LINC00486|NTRK3.3
SLC26A3|BRAF
NTRK2|NTRK2
PTEN|PTEN
GNAQ|GNAQ
GNAQ
EML4|MTMR8
BRAF|PCSK7
BRAF|BRAF
GNAQ|GNAQ.1
GNAQ|GNAQ.2
GNAQ|GNAQ.3
UTRN|NTRK2
PIK3CA|PIK3CA
PTEN|PTEN.1
Quality
0
50
100
150
200
250
Fusion-supporting reads
100
200
300
400

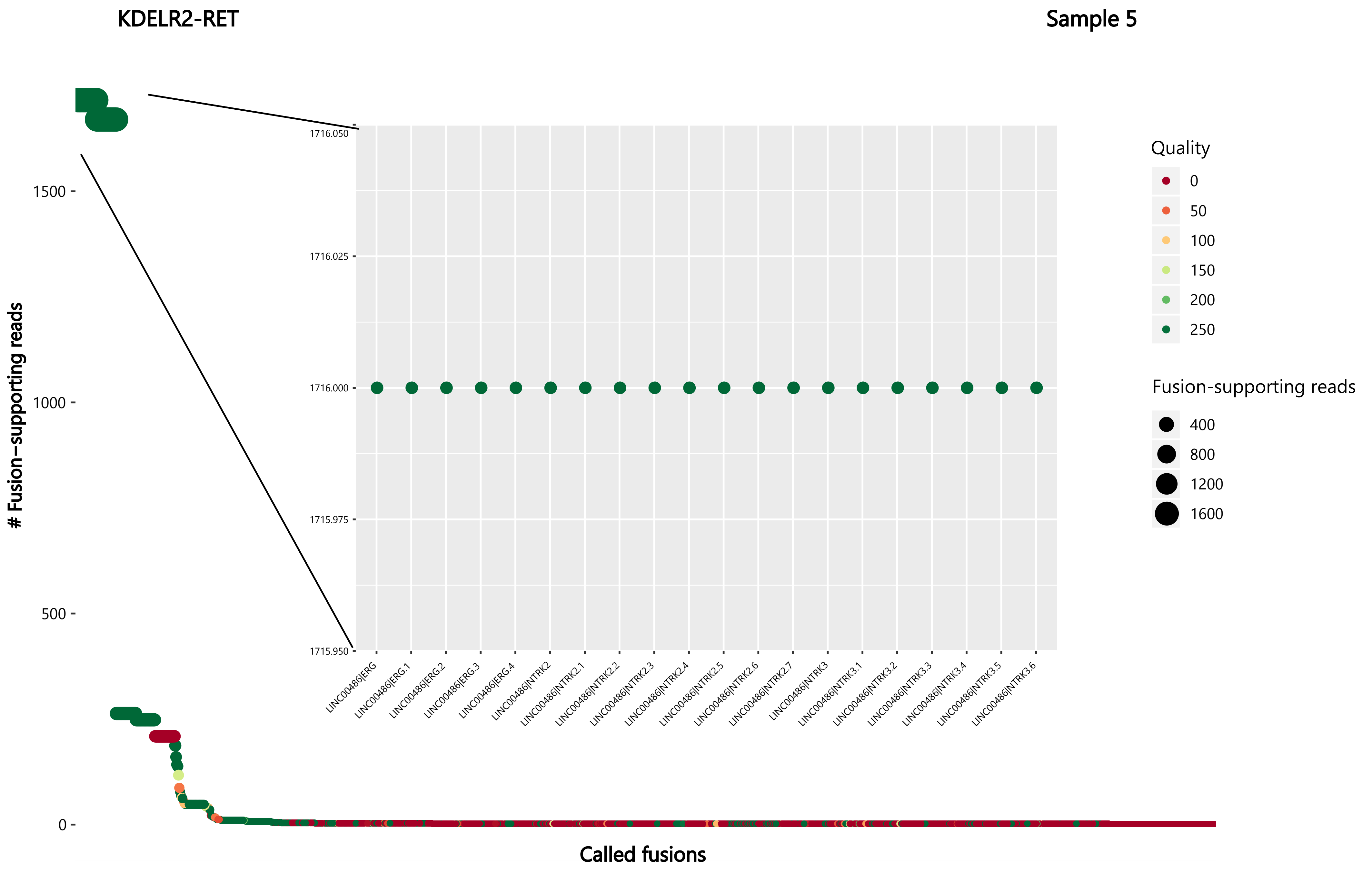

KDELR2-RET
Sample 5
Fusion−supporting reads
1500
1000
500
0
Called fusions
1716.050
1716.025
1716.000
1715.975
1715.950
LINC00486|ERG
LINC00486|ERG.1
LINC00486|ERG.2
LINC00486|ERG.3
LINC00486|ERG.4
LINC00486|NTRK2
LINC00486|NTRK2.1
LINC00486|NTRK2.2
LINC00486|NTRK2.3
LINC00486|NTRK2.4
LINC00486|NTRK2.5
LINC00486|NTRK2.6
LINC00486|NTRK2.7
LINC00486|NTRK3
LINC00486|NTRK3.1
LINC00486|NTRK3.2
LINC00486|NTRK3.3
LINC00486|NTRK3.4
LINC00486|NTRK3.5
LINC00486|NTRK3.6
Quality
0
50
100
150
200
250
Fusion-supporting reads
400
800
1200
1600

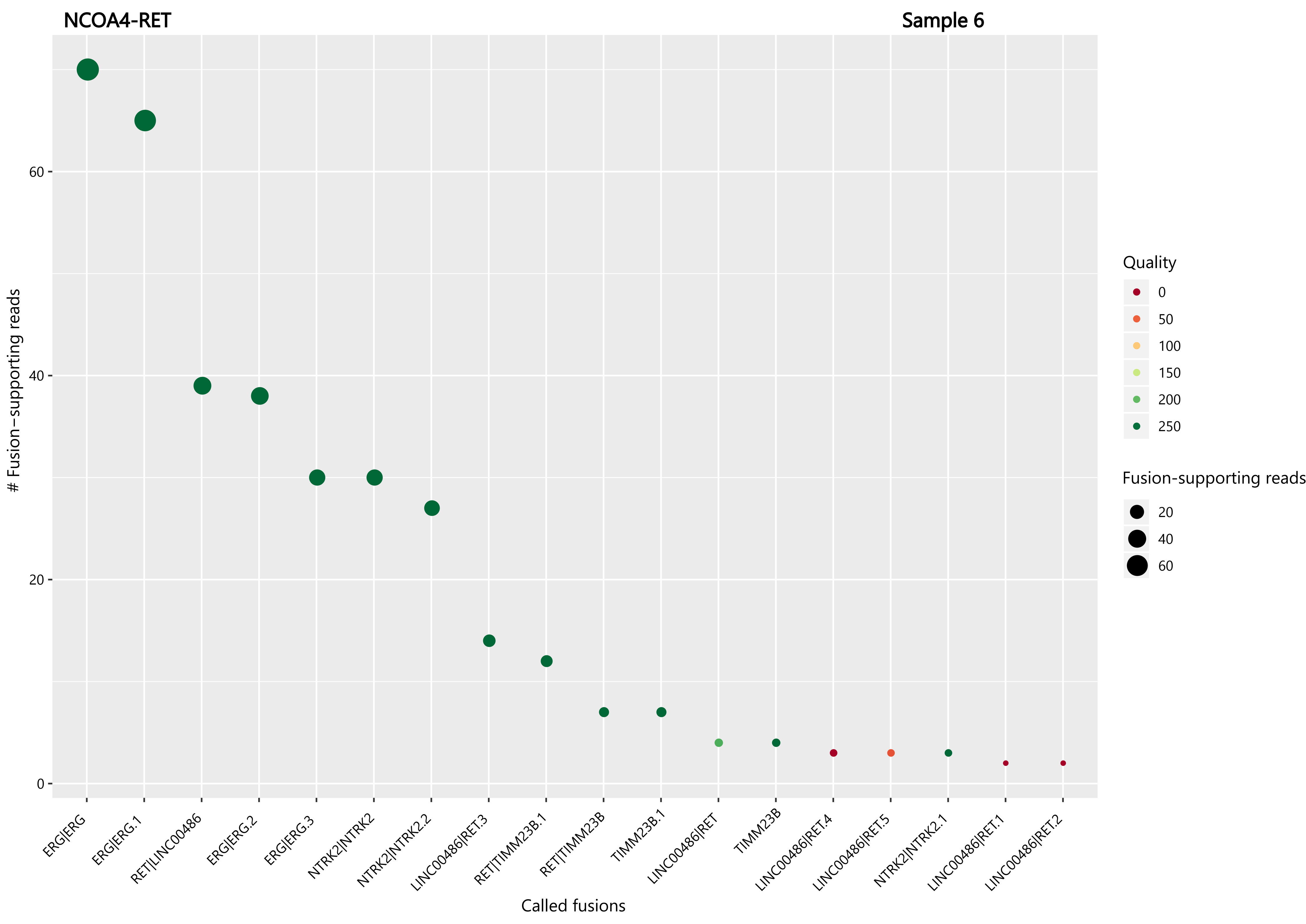

NCOA4-RET
Sample 6
Fusion−supporting reads
0
20
40
60
ERG|ERG
ERG|ERG.1
RET|LINC00486
ERG|ERG.2
ERG|ERG.3
NTRK2|NTRK2
NTRK2|NTRK2.2
LINC00486|RET.3
RET|TIMM23B.1
RET|TIMM23B
TIMM23B.1
LINC00486|RET
TIMM23B
LINC00486|RET.4
LINC00486|RET.5
NTRK2|NTRK2.1
LINC00486|RET.1
LINC00486|RET.2
Called fusions
Quality
0
50
100
150
200
250
Fusion-supporting reads
20
40
60

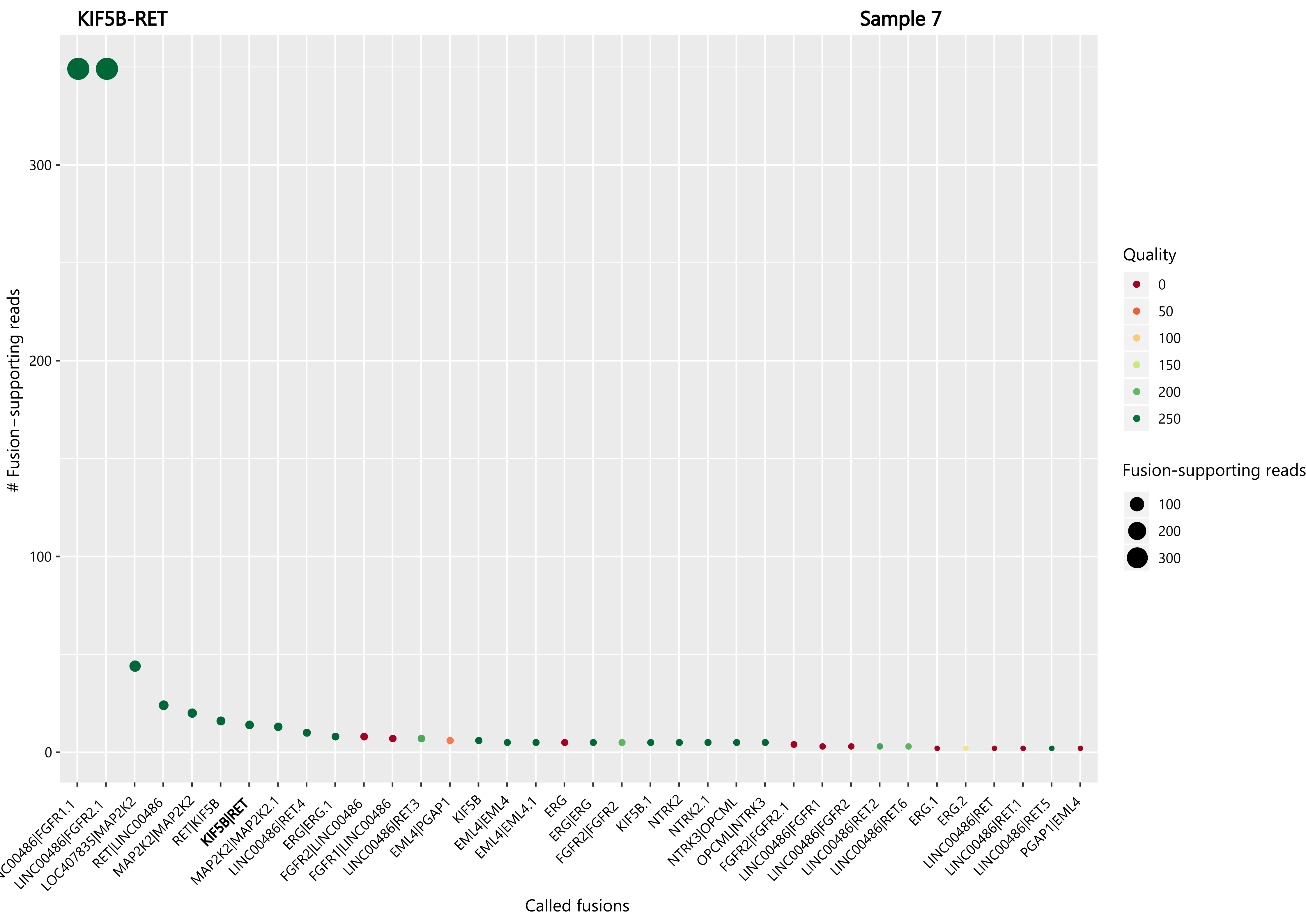

KIF5B-RET
Sample 7
Fusion−supporting reads
300
200
100
0
Called fusions
LINC00486|FGFR2.1
LOC407835|MAP2K2
RET|LINC00486
MAP2K2|MAP2K2
RET|KIF5B
KIF5B|RET
MAP2K2|MAP2K2.1
LINC00486|RET.4
ERG|ERG.1
FGFR2|LINC00486
FGFR1|LINC00486
LINC00486|RET.3
EML4|PGAP1
KIF5B
EML4|EML4
EML4|EML4.1
ERG
ERG|ERG
FGFR2|FGFR2
KIF5B.1
NTRK2
NTRK2.1
NTRK3|OPCML
OPCML|NTRK3
FGFR2|FGFR2.1
LINC00486|FGFR1
LINC00486|FGFR2
LINC00486|RET.2
LINC00486|RET.6
ERG.1
ERG.2
LINC00486|RET
LINC00486|RET.1
LINC00486|RET.5
PGAP1|EML4
Quality
0
50
100
150
200
250
Fusion-supporting reads
100
200
300

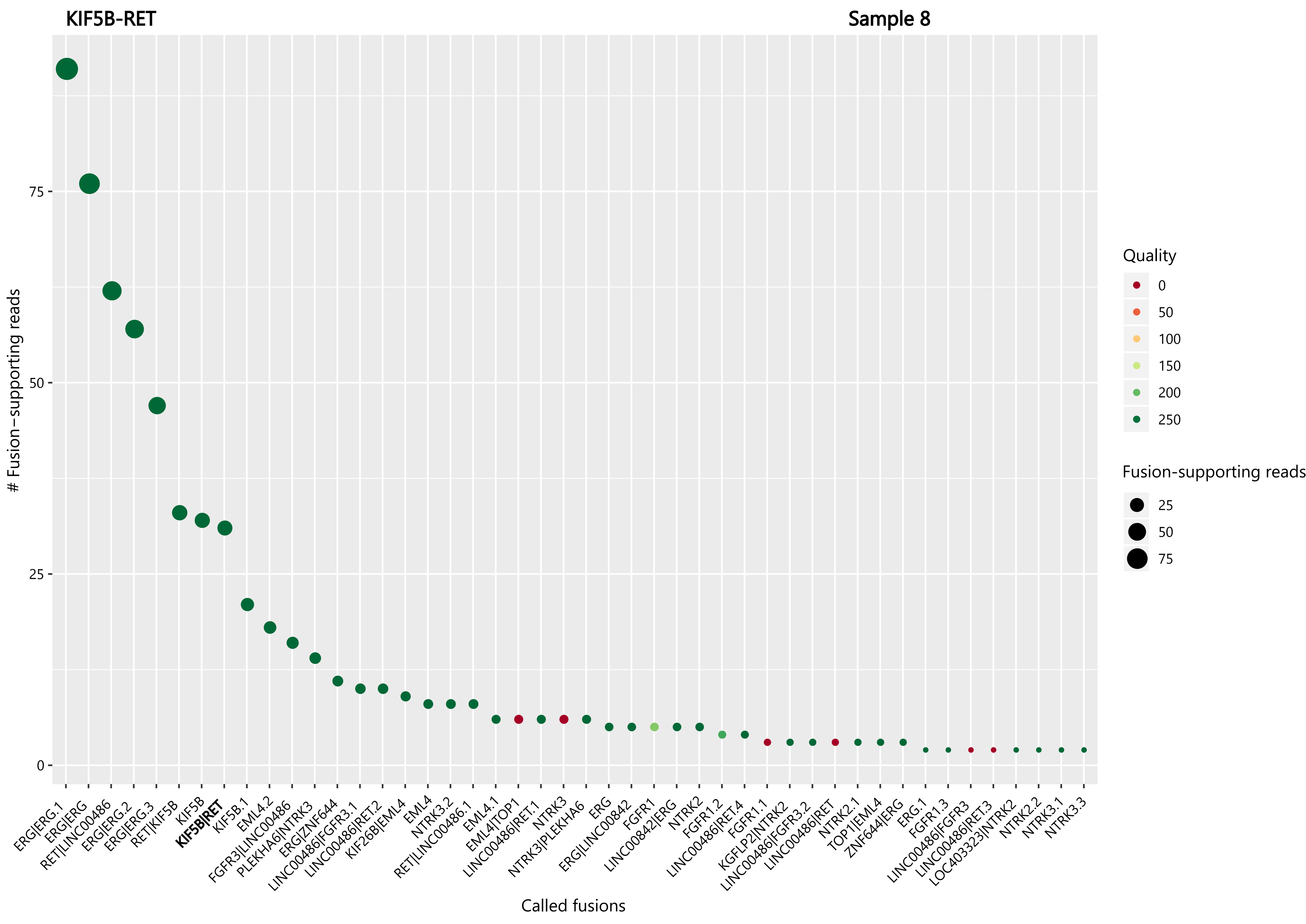

KIF5B-RET
Sample 8
Fusion−supporting reads
75
50
25
0
Called fusions
ERG|ERG.1
ERG|ERG
RET|LINC00486
ERG|ERG.2
ERG|ERG.3
RET|KIF5B
KIF5B
KIF5B|RET
KIF5B.1
EML4.2
FGFR3|LINC00486
PLEKHA6|NTRK3
ERG|ZNF644
LINC00486|FGFR3.1
LINC00486|RET.2
KIF26B|EML4
EML4
NTRK3.2
RET|LINC00486.1
EML4.1
EML4|TOP1
LINC00486|RET.1
NTRK3
NTRK3|PLEKHA6
ERG
ERG|LINC00842
FGFR1
LINC00842|ERG
NTRK2
FGFR1.2
LINC00486|RET.4
FGFR1.1
KGFLP2|NTRK2
LINC00486|FGFR3.2
LINC00486|RET
NTRK2.1
TOP1|EML4
ZNF644|ERG
ERG.1
FGFR1.3
LINC00486|FGFR3
LINC00486|RET.3
LOC403323|NTRK2
NTRK2.2
NTRK3.1
NTRK3.3
Quality
0
50
100
150
200
250
Fusion-supporting reads
25
50
75

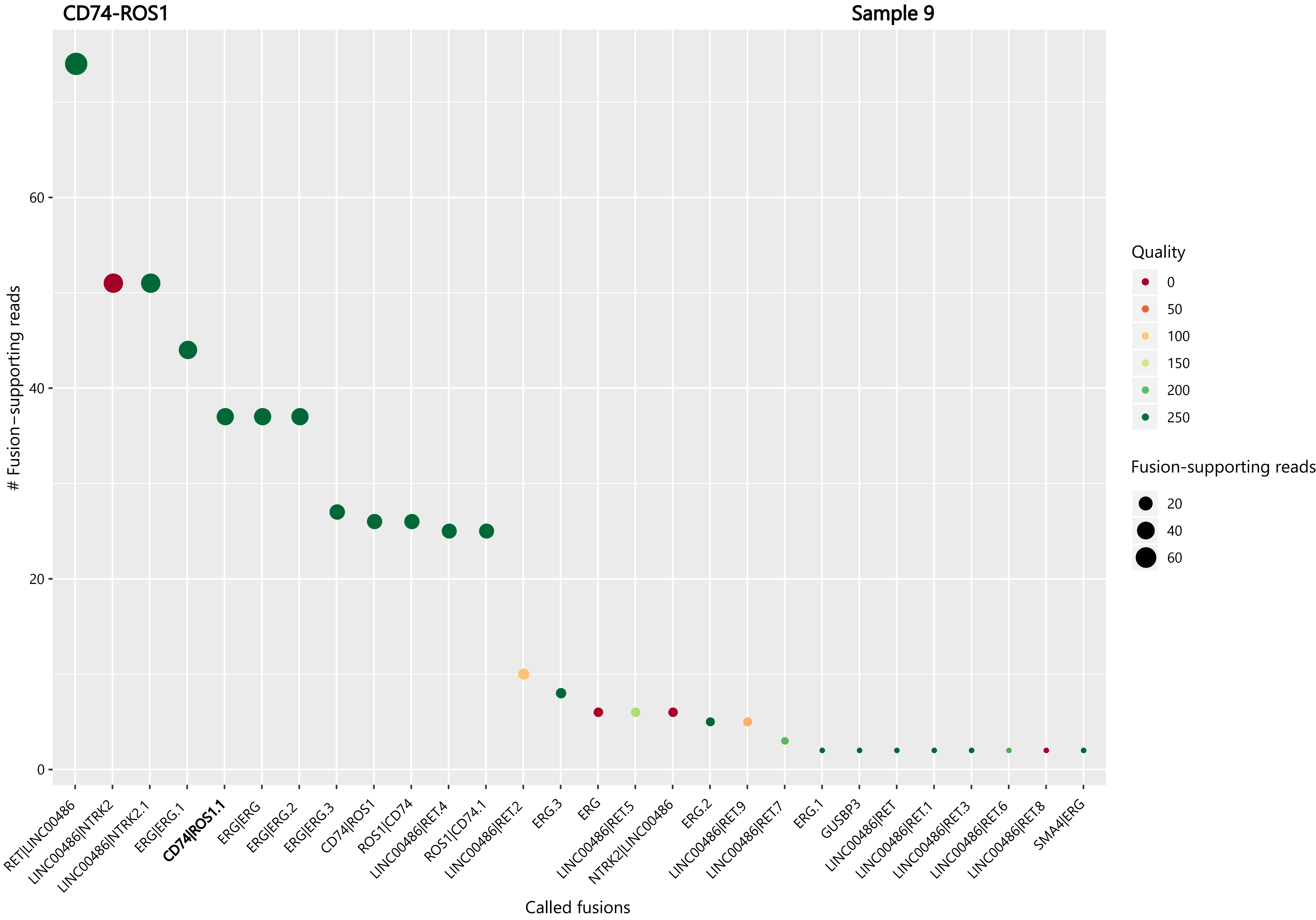
CD74-ROS1
Sample 9
Fusion−supporting reads
0
20
40
60
Called fusions
RET|LINC00486
LINC00486|NTRK2
LINC00486|NTRK2.1
ERG|ERG.1
CD74|ROS1.1
ERG|ERG
ERG|ERG.2
ERG|ERG.3
CD74|ROS1
ROS1|CD74
LINC00486|RET.4
ROS1|CD74.1
LINC00486|RET.2
ERG.3
ERG
LINC00486|RET.5
NTRK2|LINC00486
ERG.2
LINC00486|RET.9
LINC00486|RET.7
ERG.1
GUSBP3
LINC00486|RET
LINC00486|RET.1
LINC00486|RET.3
LINC00486|RET.6
LINC00486|RET.8
SMA4|ERG
Quality
0
50
100
150
200
250
Fusion-supporting reads
20
40
60

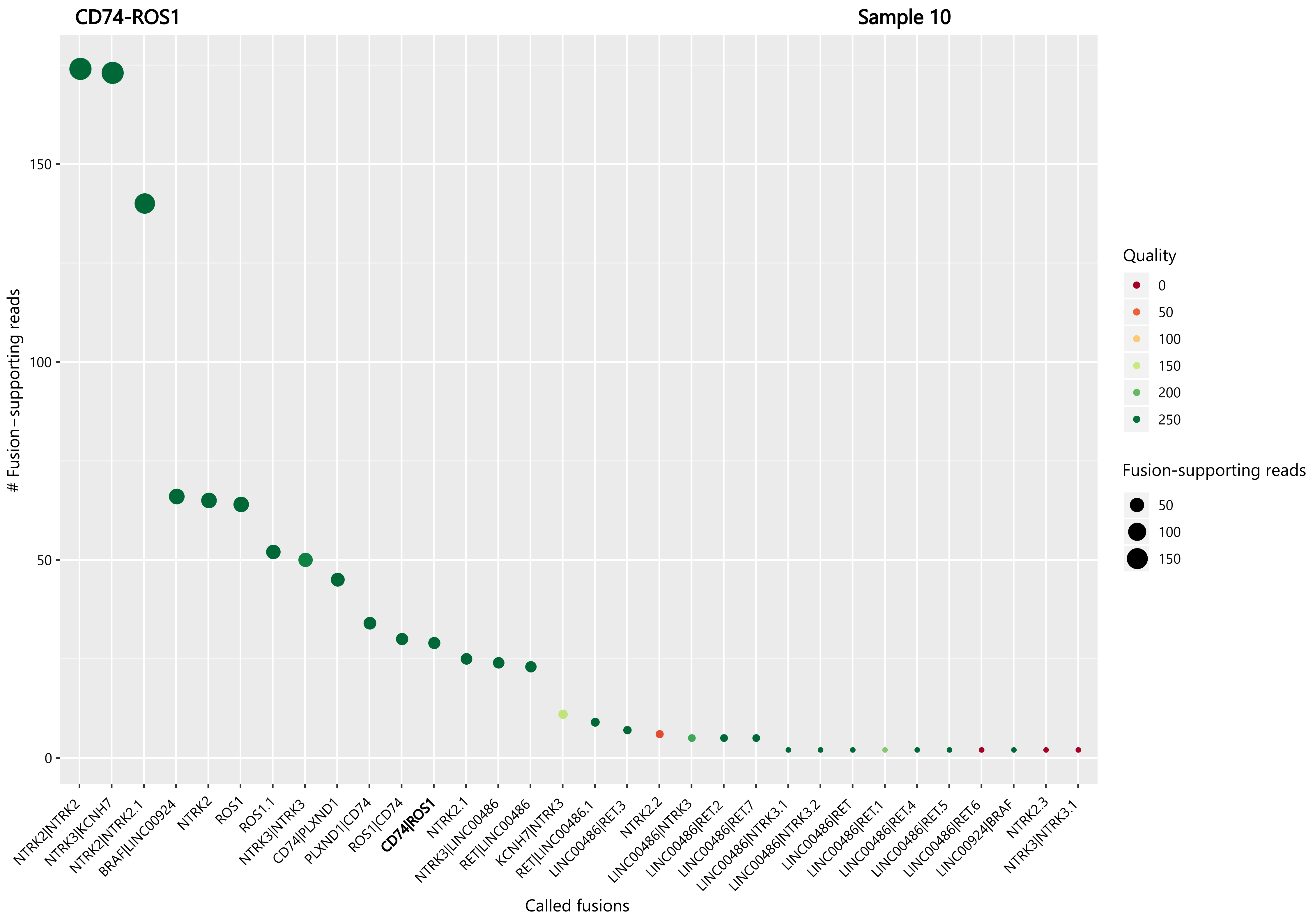

CD74-ROS1
Sample 10
Fusion−supporting reads
150
100
50
0
Called fusions
NTRK2|NTRK2
NTRK3|KCNH7
NTRK2|NTRK2.1
BRAF|LINC00924
NTRK2
ROS1
ROS1.1
NTRK3|NTRK3
CD74|PLXND1
PLXND1|CD74
ROS1|CD74
CD74|ROS1
NTRK2.1
NTRK3|LINC00486
RET|LINC00486
KCNH7|NTRK3
RET|LINC00486.1
LINC00486|RET.3
NTRK2.2
LINC00486|NTRK3
LINC00486|RET.2
LINC00486|RET.7
LINC00486|NTRK3.1
LINC00486|NTRK3.2
LINC00486|RET
LINC00486|RET.1
LINC00486|RET.4
LINC00486|RET.5
LINC00486|RET.6
LINC00924|BRAF
NTRK2.3
NTRK3|NTRK3.1
Quality
0
50
100
150
200
250
Fusion-supporting reads
50
100
150

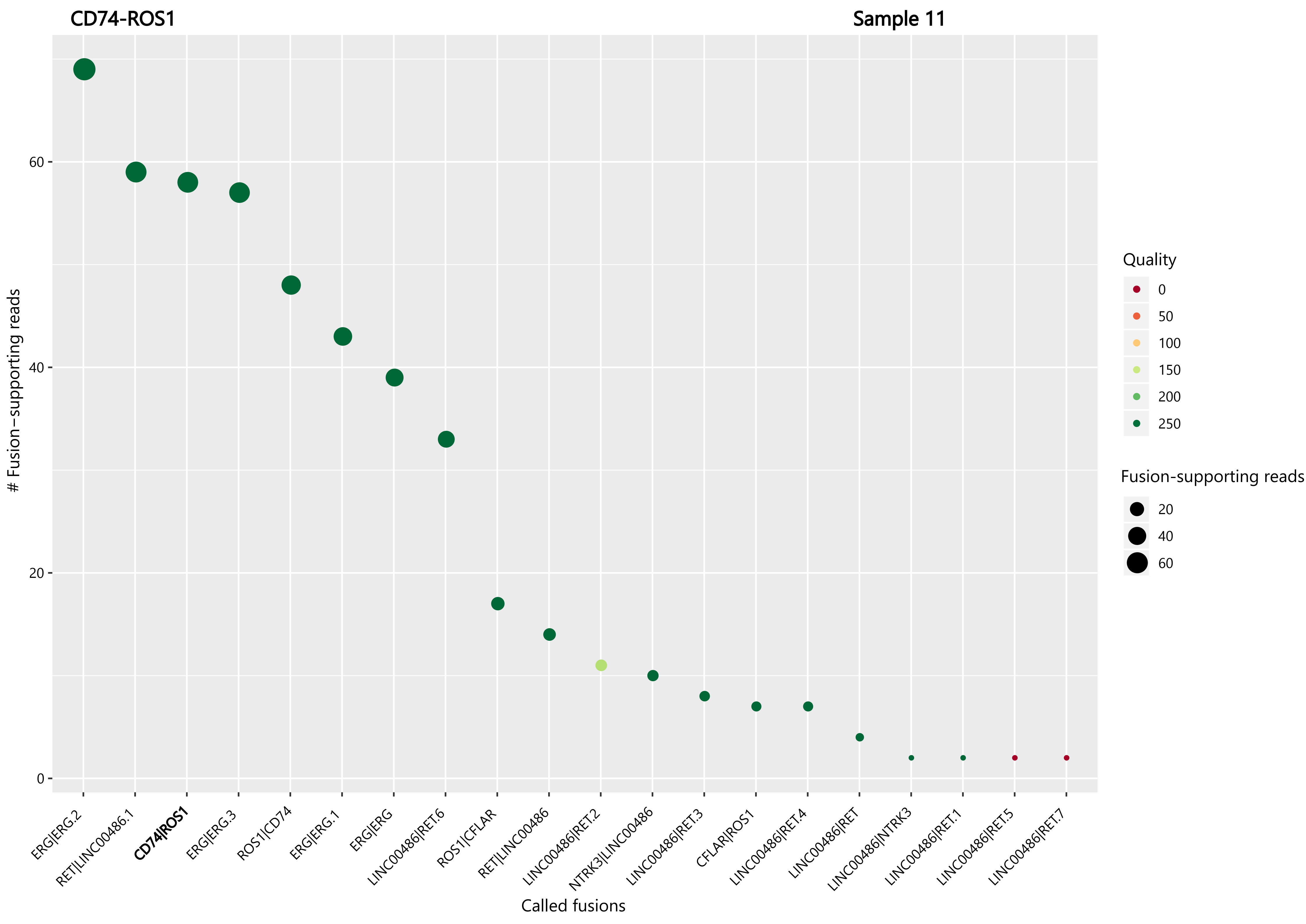

CD74-ROS1
Sample 11
Fusion−supporting reads
0
20
40
60
Called fusions
ERG|ERG.2
RET|LINC00486.1
CD74|ROS1
ERG|ERG.3
ROS1|CD74
ERG|ERG.1
ERG|ERG
LINC00486|RET.6
ROS1|CFLAR
RET|LINC00486
LINC00486|RET.2
NTRK3|LINC00486
LINC00486|RET.3
CFLAR|ROS1
LINC00486|RET.4
LINC00486|RET
LINC00486|NTRK3
LINC00486|RET.1
LINC00486|RET.5
LINC00486|RET.7
Quality
0
50
100
150
200
250
Fusion-supporting reads
20
40
60

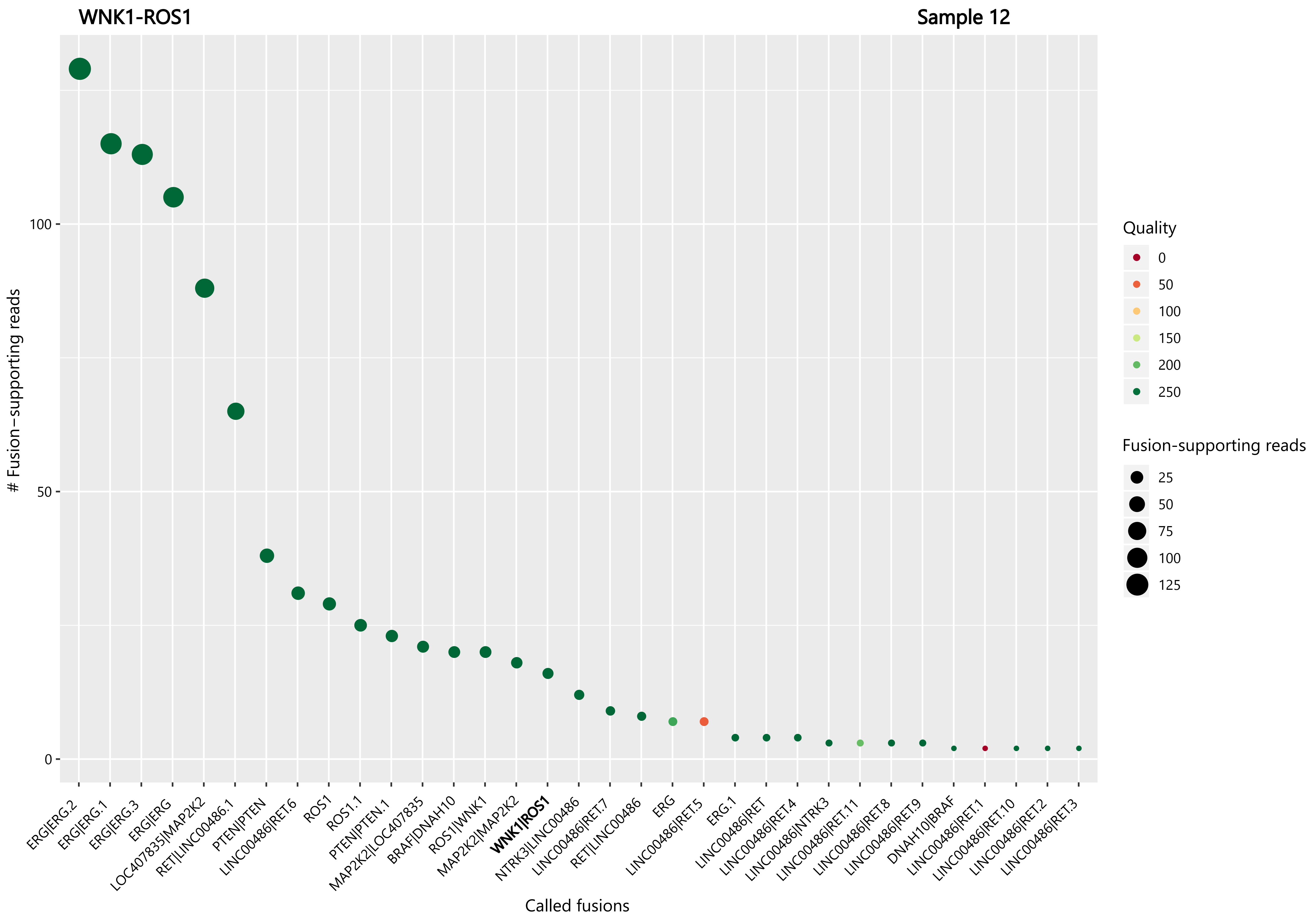

WNK1-ROS1
Sample 12
Fusion−supporting reads
100
50
0
Called fusions
ERG|ERG.2
ERG|ERG.1
ERG|ERG.3
ERG|ERG
LOC407835|MAP2K2
RET|LINC00486.1
PTEN|PTEN
LINC00486|RET.6
ROS1
ROS1.1
PTEN|PTEN.1
MAP2K2|LOC407835
BRAF|DNAH10
ROS1|WNK1
MAP2K2|MAP2K2
WNK1|ROS1
NTRK3|LINC00486
LINC00486|RET.7
RET|LINC00486
ERG
LINC00486|RET.5
ERG.1
LINC00486|RET
LINC00486|RET.4
LINC00486|NTRK3
LINC00486|RET.11
LINC00486|RET.8
LINC00486|RET.9
DNAH10|BRAF
LINC00486|RET.1
LINC00486|RET.10
LINC00486|RET.2
LINC00486|RET.3
Quality
0
50
100
150
200
250
Fusion-supporting reads
25
50
75
100
125

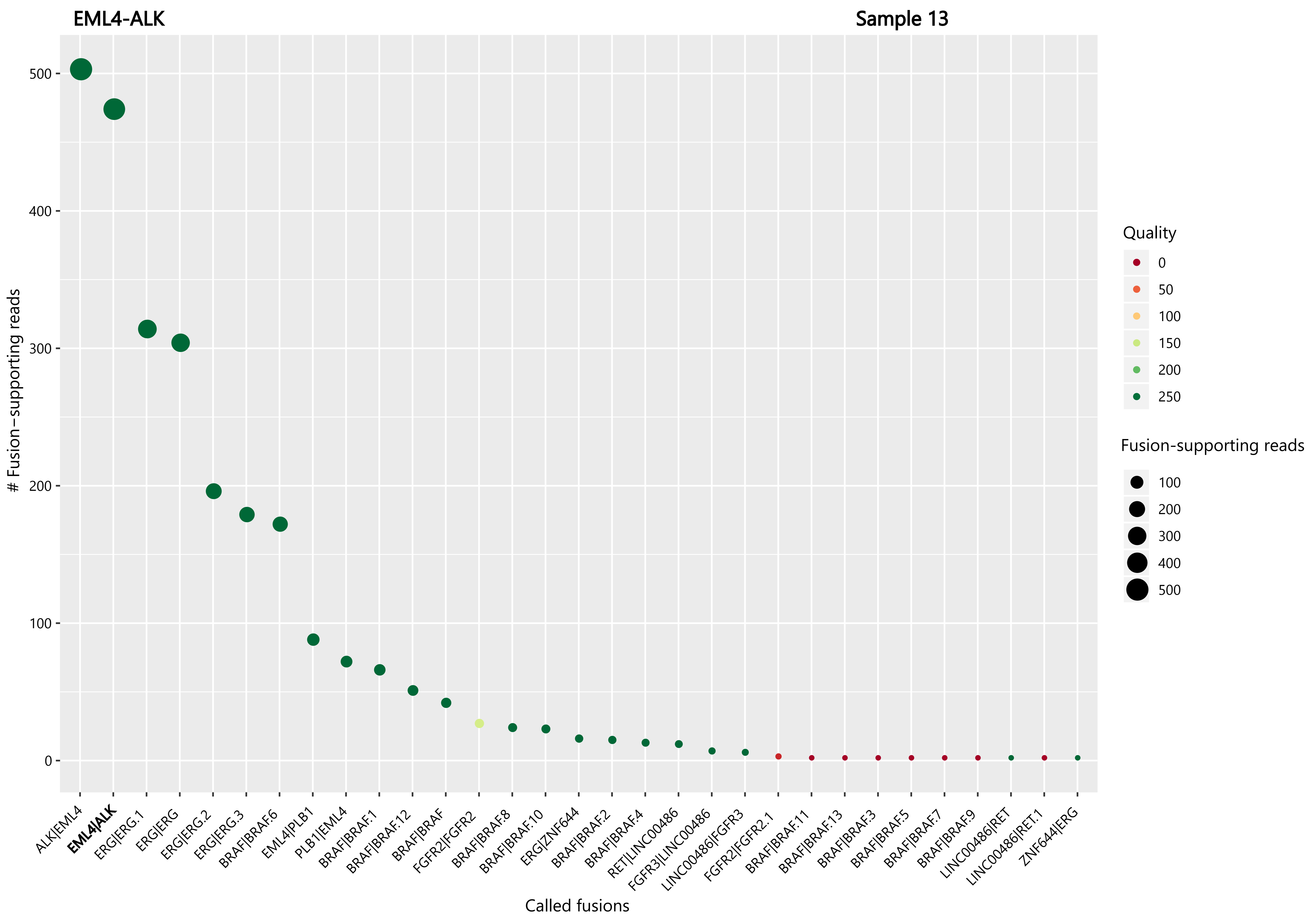
EML4-ALK
Sample 13
Fusion−supporting reads
0
100
200
300
400
500
ALK|EML4
EML4|ALK
ERG|ERG.1
ERG|ERG
ERG|ERG.2
ERG|ERG.3
BRAF|BRAF.6
EML4|PLB1
PLB1|EML4
BRAF|BRAF.1
BRAF|BRAF.12
BRAF|BRAF
FGFR2|FGFR2
BRAF|BRAF.8
BRAF|BRAF.10
ERG|ZNF644
BRAF|BRAF.2
BRAF|BRAF.4
RET|LINC00486
FGFR3|LINC00486
LINC00486|FGFR3
FGFR2|FGFR2.1
BRAF|BRAF.11
BRAF|BRAF.13
BRAF|BRAF.3
BRAF|BRAF.5
BRAF|BRAF.7
BRAF|BRAF.9
LINC00486|RET
LINC00486|RET.1
ZNF644|ERG
Called fusions
Quality
0
50
100
150
200
250
Fusion-supporting reads
100
200
300
400
500

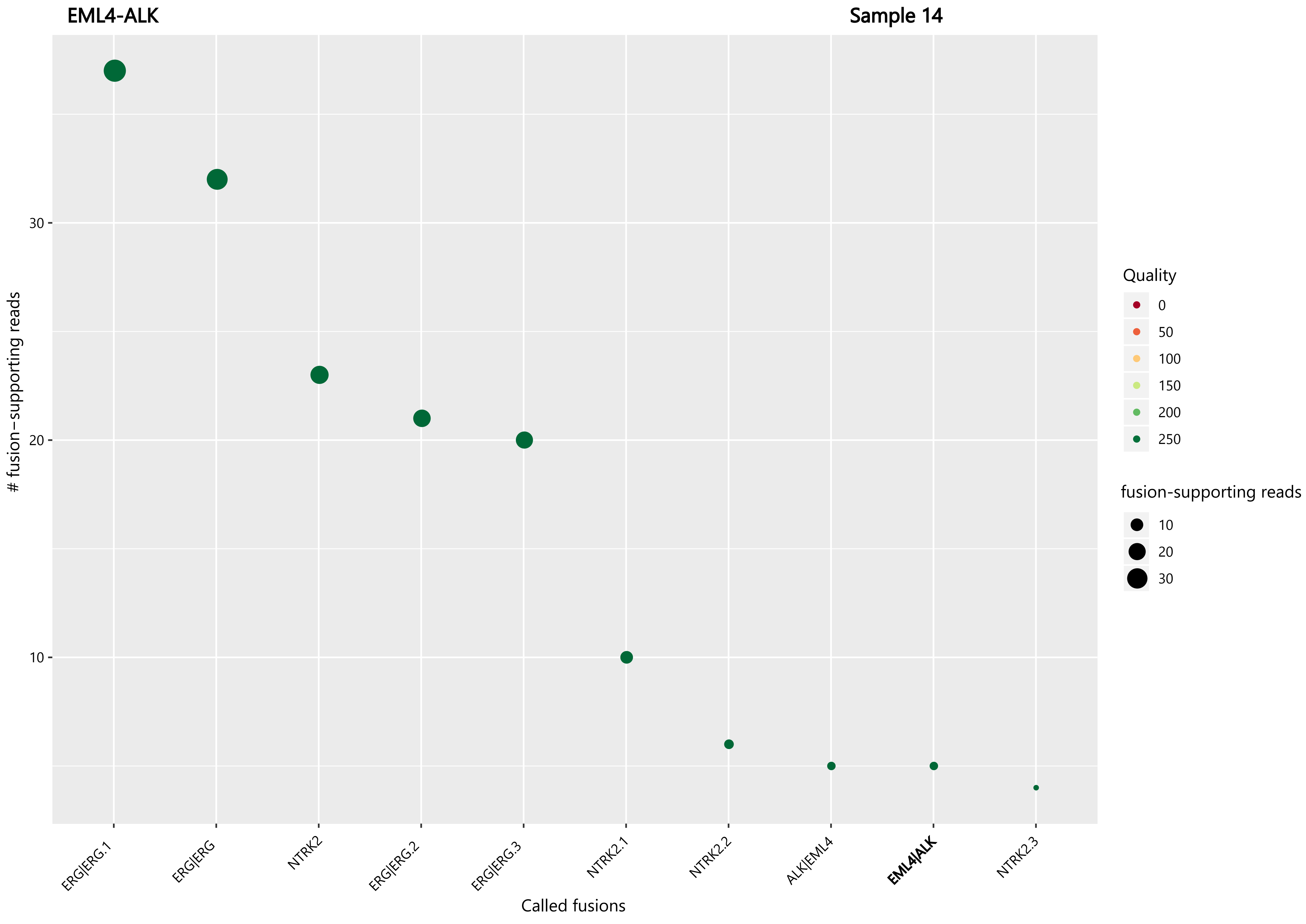
EML4-ALK
Sample 14
fusion−supporting reads
30
20
10
Called fusions
ERG|ERG.1
ERG|ERG
NTRK2
ERG|ERG.2
ERG|ERG.3
NTRK2.1
NTRK2.2
ALK|EML4
EML4|ALK
NTRK2.3
Quality
0
50
100
150
200
250
fusion-supporting reads
10
20
30

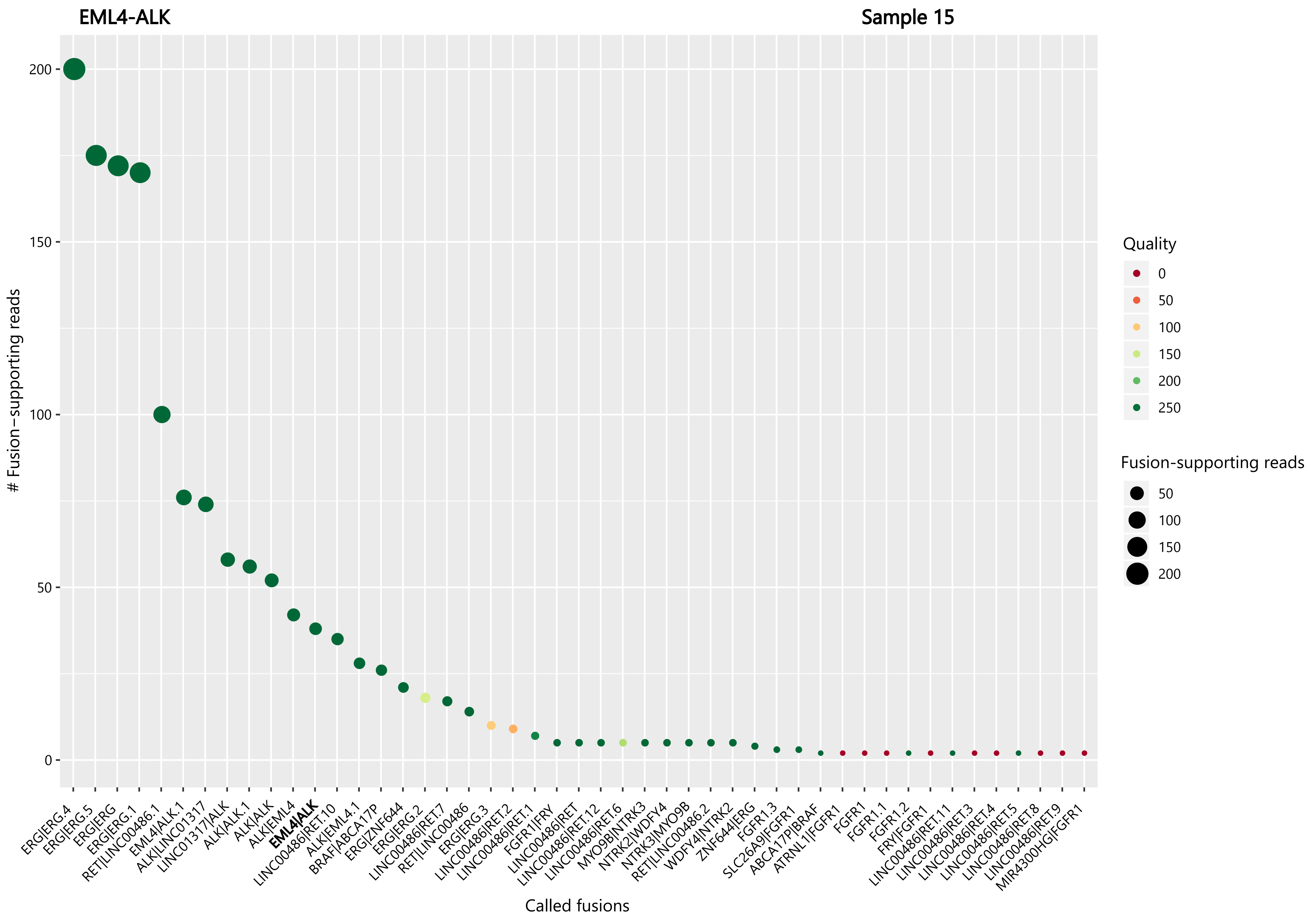
EML4-ALK
Sample 15
Fusion−supporting reads
Called fusions
Quality
Fusion-supporting reads
ERG|ERG.4
ERG|ERG.5
ERG|ERG
ERG|ERG.1
RET|LINC00486.1
EML4|ALK.1
ALK|LINC01317
LINC01317|ALK
ALK|ALK.1
ALK|ALK
ALK|EML4
EML4|ALK
LINC00486|RET.10
ALK|EML4.1
BRAF|ABCA17P
ERG|ZNF644
ERG|ERG.2
LINC00486|RET.7
RET|LINC00486
ERG|ERG.3
LINC00486|RET.2
LINC00486|RET.1
FGFR1|FRY
LINC00486|RET
LINC00486|RET.12
LINC00486|RET.6
MYO9B|NTRK3
NTRK2|WDFY4
NTRK3|MYO9B
RET|LINC00486.2
WDFY4|NTRK2
ZNF644|ERG
FGFR1.3
SLC26A9|FGFR1
ABCA17P|BRAF
ATRNL1|FGFR1
FGFR1
FGFR1.1
FGFR1.2
FRY|FGFR1
LINC00486|RET.11
LINC00486|RET.3
LINC00486|RET.4
LINC00486|RET.5
LINC00486|RET.8
LINC00486|RET.9
MIR4300HG|FGFR1

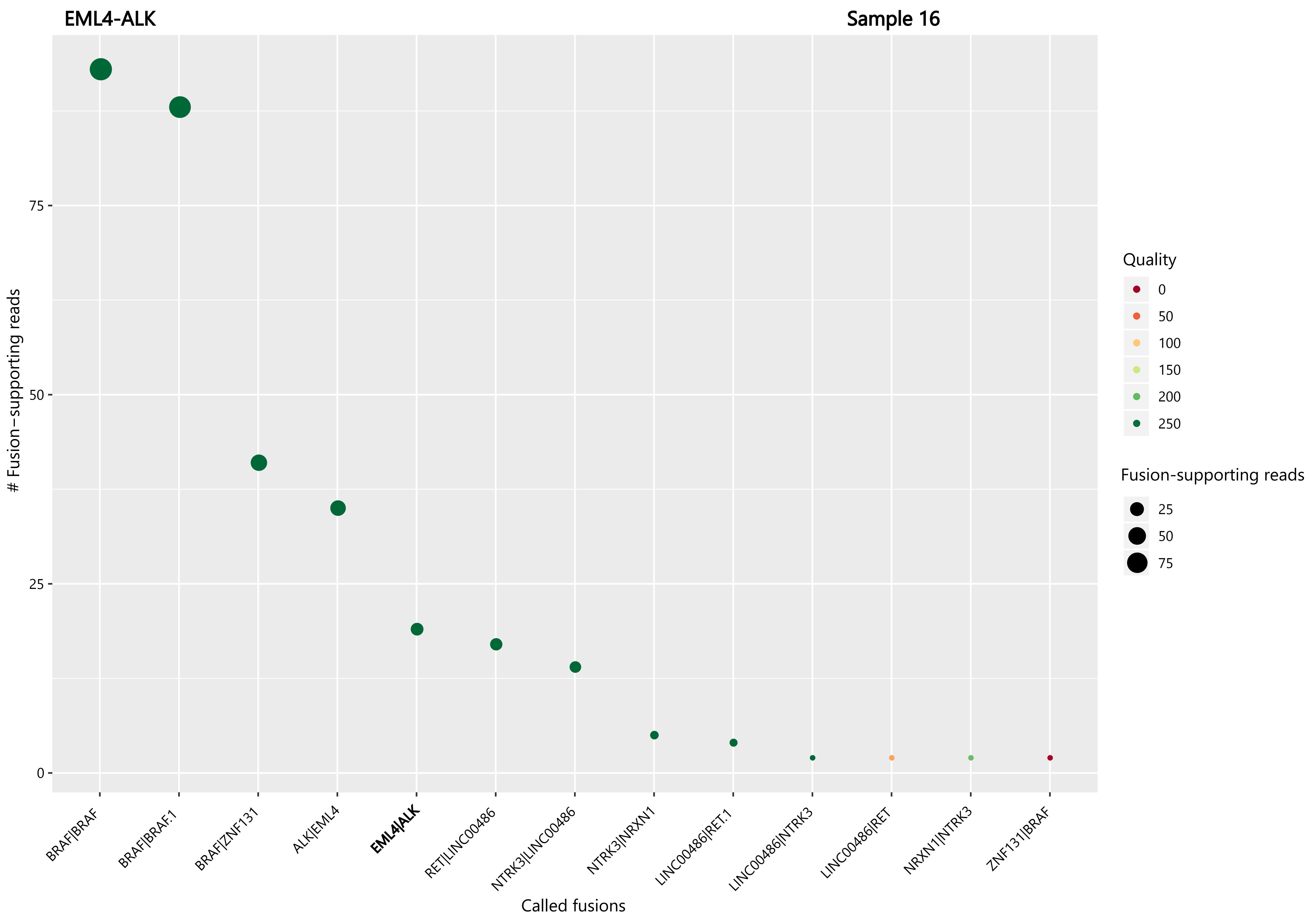

EML4-ALK
Sample 16
Fusion−supporting reads
0
25
50
75
Called fusions
BRAF|BRAF
BRAF|BRAF.1
BRAF|ZNF131
ALK|EML4
EML4|ALK
RET|LINC00486
NTRK3|LINC00486
NTRK3|NRXN1
LINC00486|RET.1
LINC00486|NTRK3
LINC00486|RET
NRXN1|NTRK3
ZNF131|BRAF
Quality
0
50
100
150
200
250
Fusion-supporting reads
25
50
75

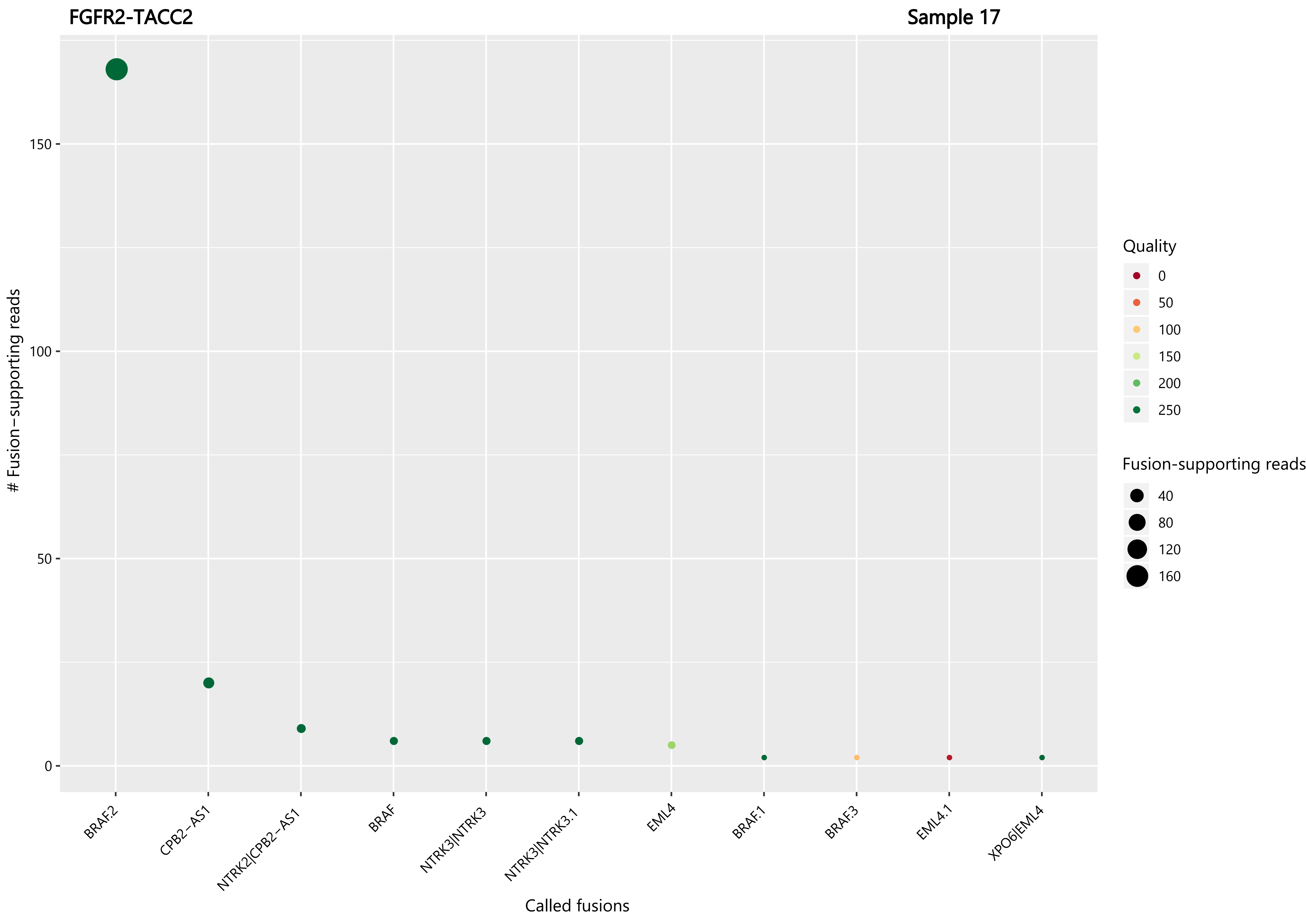
FGFR2-TACC2
Sample 17
Fusion−supporting reads
0
50
100
150
Called fusions
BRAF.2
CPB2−AS1
NTRK2|CPB2−AS1
BRAF
NTRK3|NTRK3
NTRK3|NTRK3.1
EML4
BRAF.1
BRAF.3
EML4.1
XPO6|EML4
Quality
0
50
100
150
200
250
Fusion-supporting reads
40
80
120
160

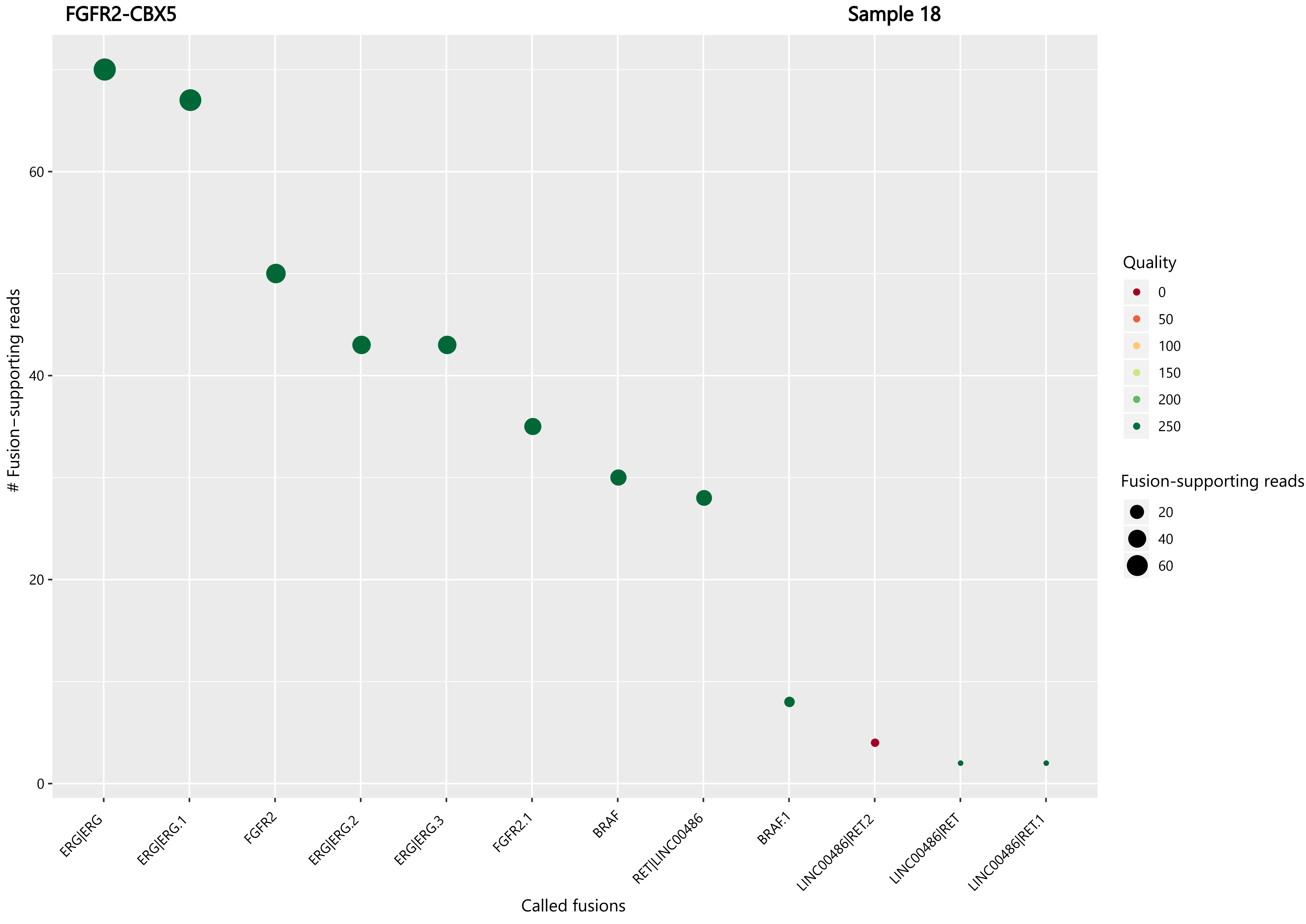

FGFR2-CBX5
Sample 18
Fusion−supporting reads
0
20
40
60
ERG|ERG
ERG|ERG.1
FGFR2
ERG|ERG.2
ERG|ERG.3
FGFR2.1
BRAF
RET|LINC00486
BRAF.1
LINC00486|RET.2
LINC00486|RET
LINC00486|RET.1
Called fusions
Quality
0
50
100
150
200
250
Fusion-supporting reads
20
40
60
