# Supplementary material for: Detection of gene fusions using targeted next-generation sequencing: a comparative evaluation
Source: BMC Med Genomics. 2021 Feb 27;14:62. doi: 10.1186/s12920-021-00909-y (PMC7912891; doi:10.1186/s12920-021-00909-y)
Supplement: Supplementary file 16 — Additional file 16: Fig. S16. Fusions detected with the SureSelect XT HS Custom Panel (Agilent) (v4.1.1.5) for all samples. Metrics such as quality control scores, in-frame status or filter thresholds were plotted when available. In cases where the same fusion was identified more than once within the same sample, a unique numbering scheme was added at the end of the name to differentiate the candidate fusions. The numbering however, does not imply any special order or preference over the other fusions with the same name. The putative detected fusions were arranged in decreasing order based on the number of fusion-supporting reads. The expected fusion for each sample was highlighted in bold. [file 12920_2021_909_MOESM16_ESM.pdf]

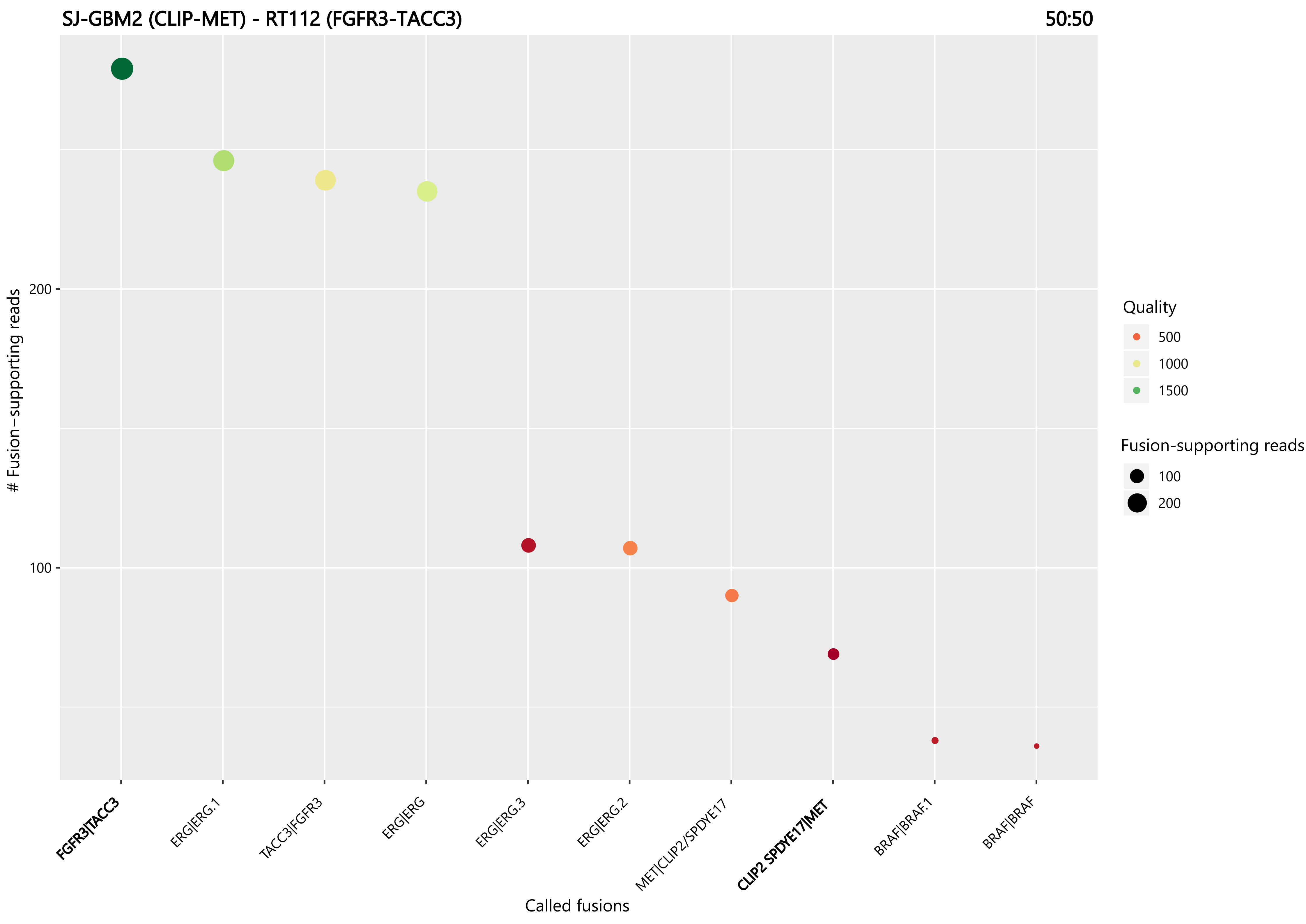

SJ-GBM2 (CLIP-MET) - RT112 (FGFR3-TACC3)
50:50
Fusion−supporting reads
200
100
Called fusions
FGFR3|TACC3
ERG|ERG.1
TACC3|FGFR3
ERG|ERG
ERG|ERG.3
ERG|ERG.2
MET|CLIP2/SPDYE17
CLIP2 SPDYE17|MET
BRAF|BRAF.1
BRAF|BRAF
Quality
500
1000
1500
Fusion-supporting reads
100
200

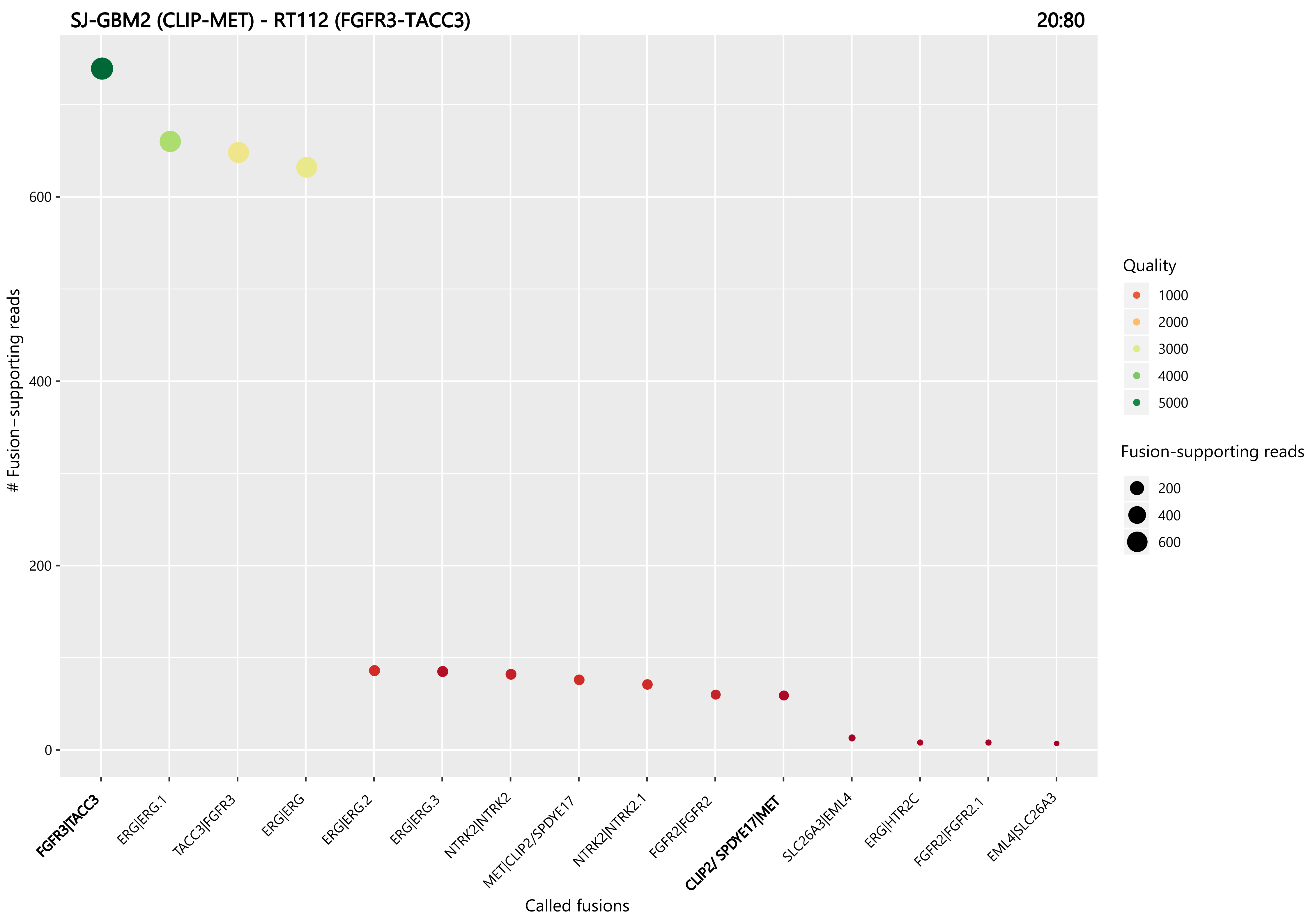

SJ-GBM2 (CLIP-MET) - RT112 (FGFR3-TACC3)
20:80
Fusion−supporting reads
600
400
200
0
Called fusions
FGFR3|TACC3
ERG|ERG.1
TACC3|FGFR3
ERG|ERG
ERG|ERG.2
ERG|ERG.3
NTRK2|NTRK2
MET|CLIP2/SPDYE17
NTRK2|NTRK2.1
FGFR2|FGFR2
CLIP2/ SPDYE17|MET
SLC26A3|EML4
ERG|HTR2C
FGFR2|FGFR2.1
EML4|SLC26A3
Quality
1000
2000
3000
4000
5000
Fusion-supporting reads
200
400
600

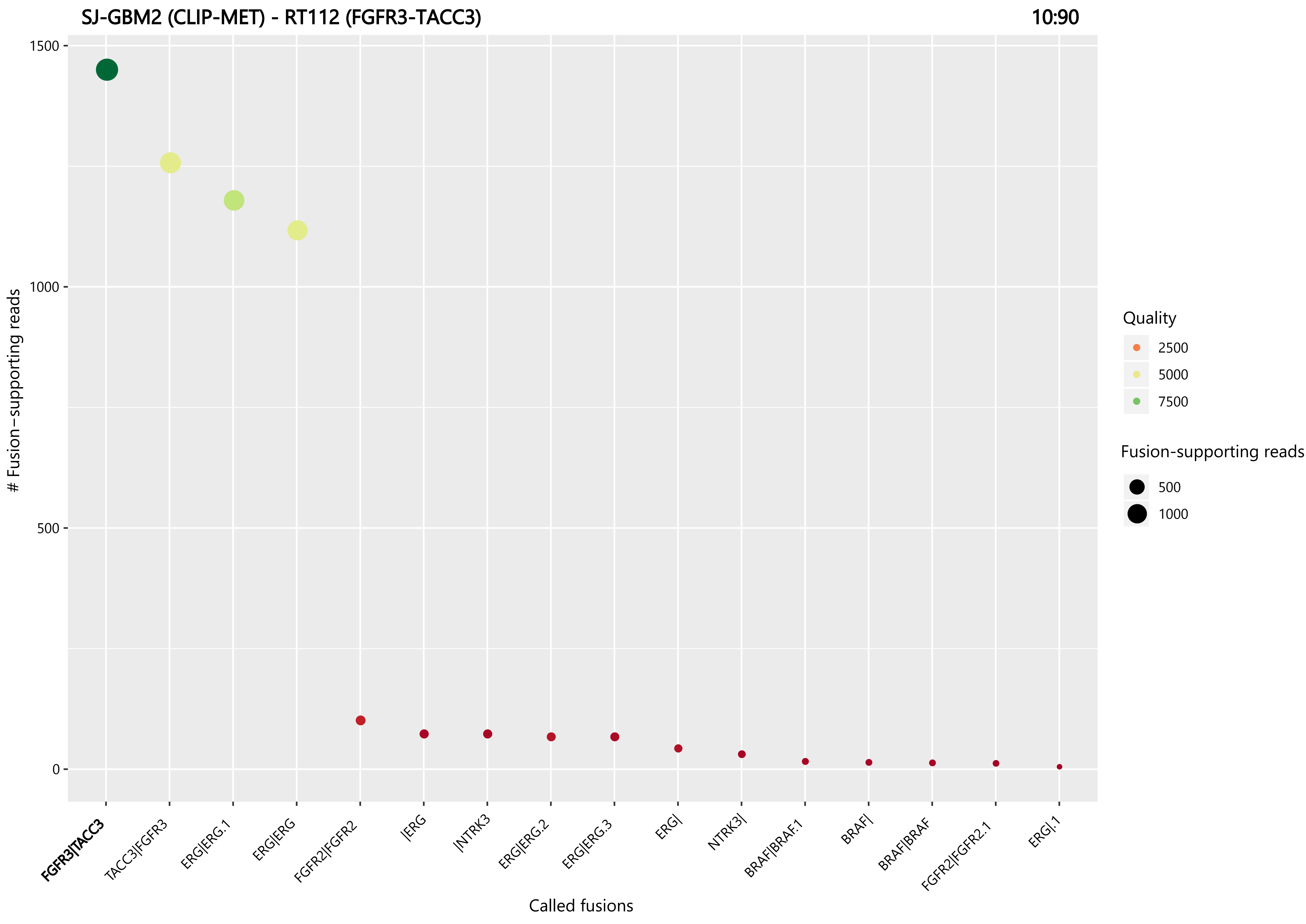

SJ-GBM2 (CLIP-MET) - RT112 (FGFR3-TACC3)
10:90
1500
1000
500
0
Fusion−supporting reads
FGFR3|TACC3
TACC3|FGFR3
ERG|ERG.1
ERG|ERG
FGFR2|FGFR2
|ERG
|NTRK3
ERG|ERG.2
ERG|ERG.3
ERG|
NTRK3|
BRAF|BRAF.1
BRAF|
BRAF|BRAF
FGFR2|FGFR2.1
ERG|.1
Called fusions
Quality
2500
5000
7500
Fusion-supporting reads
500
1000

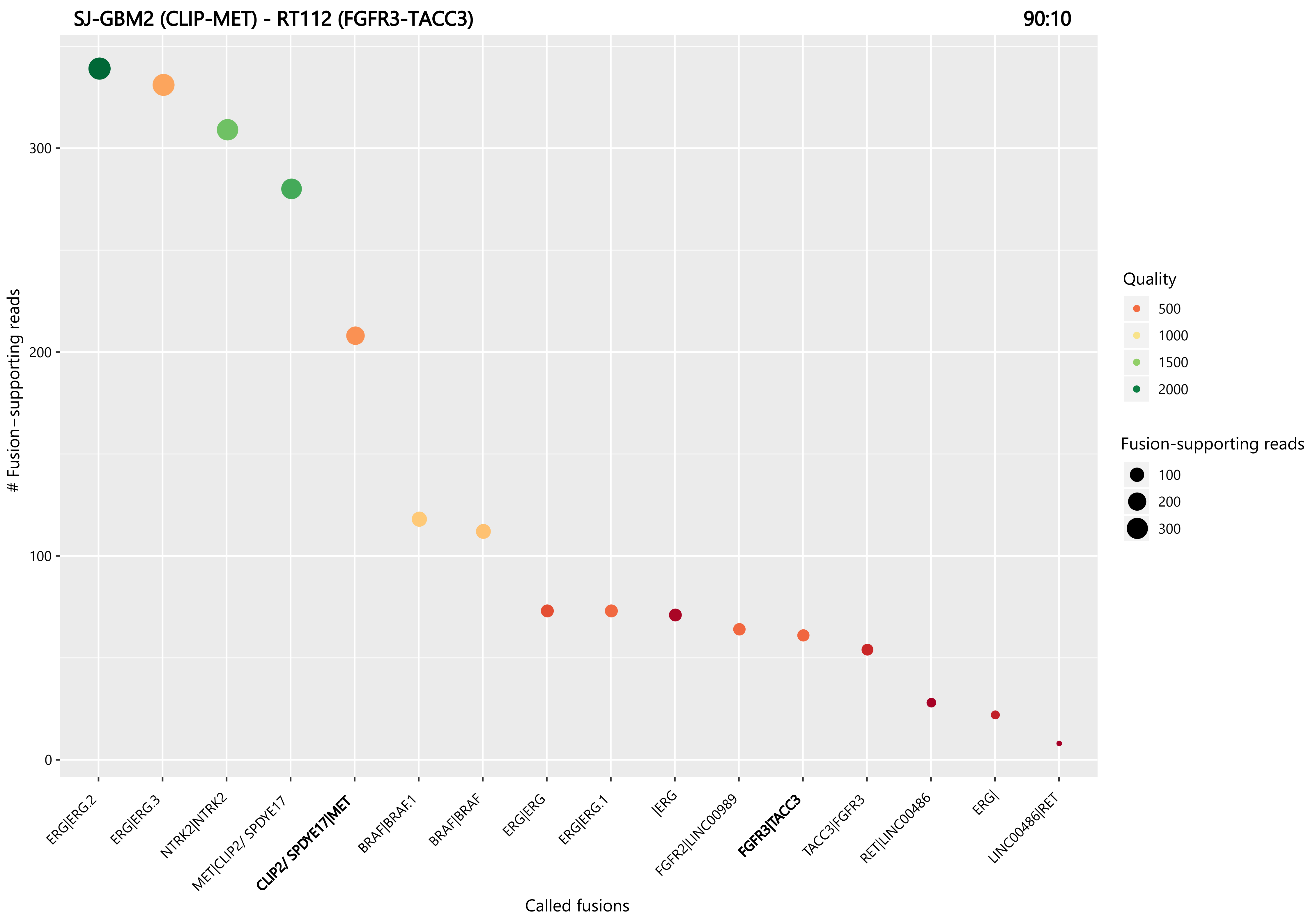

SJ-GBM2 (CLIP-MET) - RT112 (FGFR3-TACC3)
90:10
Fusion−supporting reads
0
100
200
300
ERG|ERG.2
ERG|ERG.3
NTRK2|NTRK2
MET|CLIP2/ SPDYE17
CLIP2/ SPDYE17|MET
BRAF|BRAF.1
BRAF|BRAF
ERG|ERG
ERG|ERG.1
|ERG
FGFR2|LINC00989
FGFR3|TACC3
TACC3|FGFR3
RET|LINC00486
ERG|
LINC00486|RET
Called fusions
Quality
500
1000
1500
2000
Fusion-supporting reads
100
200
300

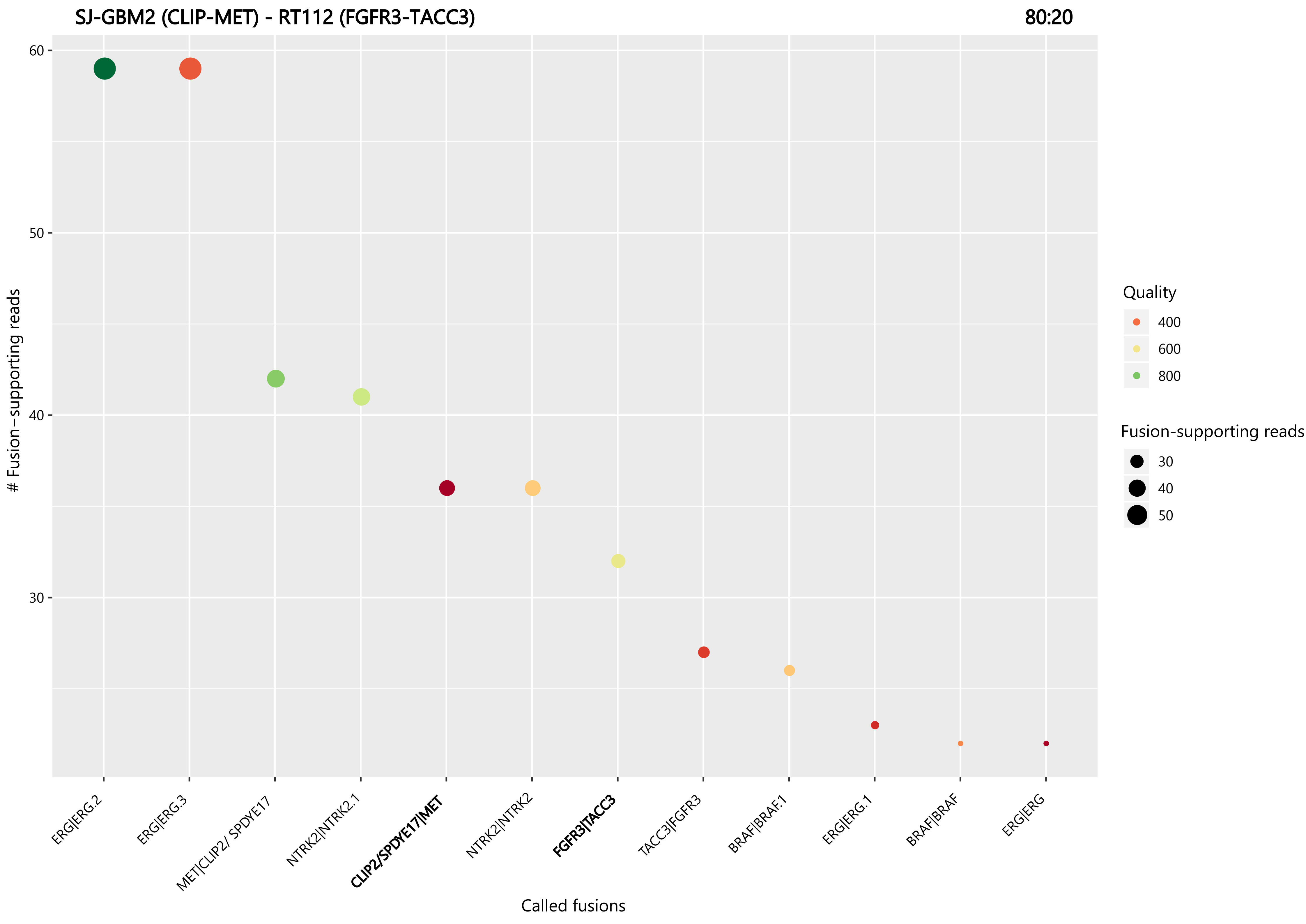

SJ-GBM2 (CLIP-MET) - RT112 (FGFR3-TACC3)
80:20
Fusion−supporting reads
60
50
40
30
Called fusions
ERG|ERG.2
ERG|ERG.3
MET|CLIP2/ SPDYE17
NTRK2|NTRK2.1
CLIP2/SPDYE17|MET
NTRK2|NTRK2
FGFR3|TACC3
TACC3|FGFR3
BRAF|BRAF.1
ERG|ERG.1
BRAF|BRAF
ERG|ERG
Quality
400
600
800
Fusion-supporting reads
30
40
50

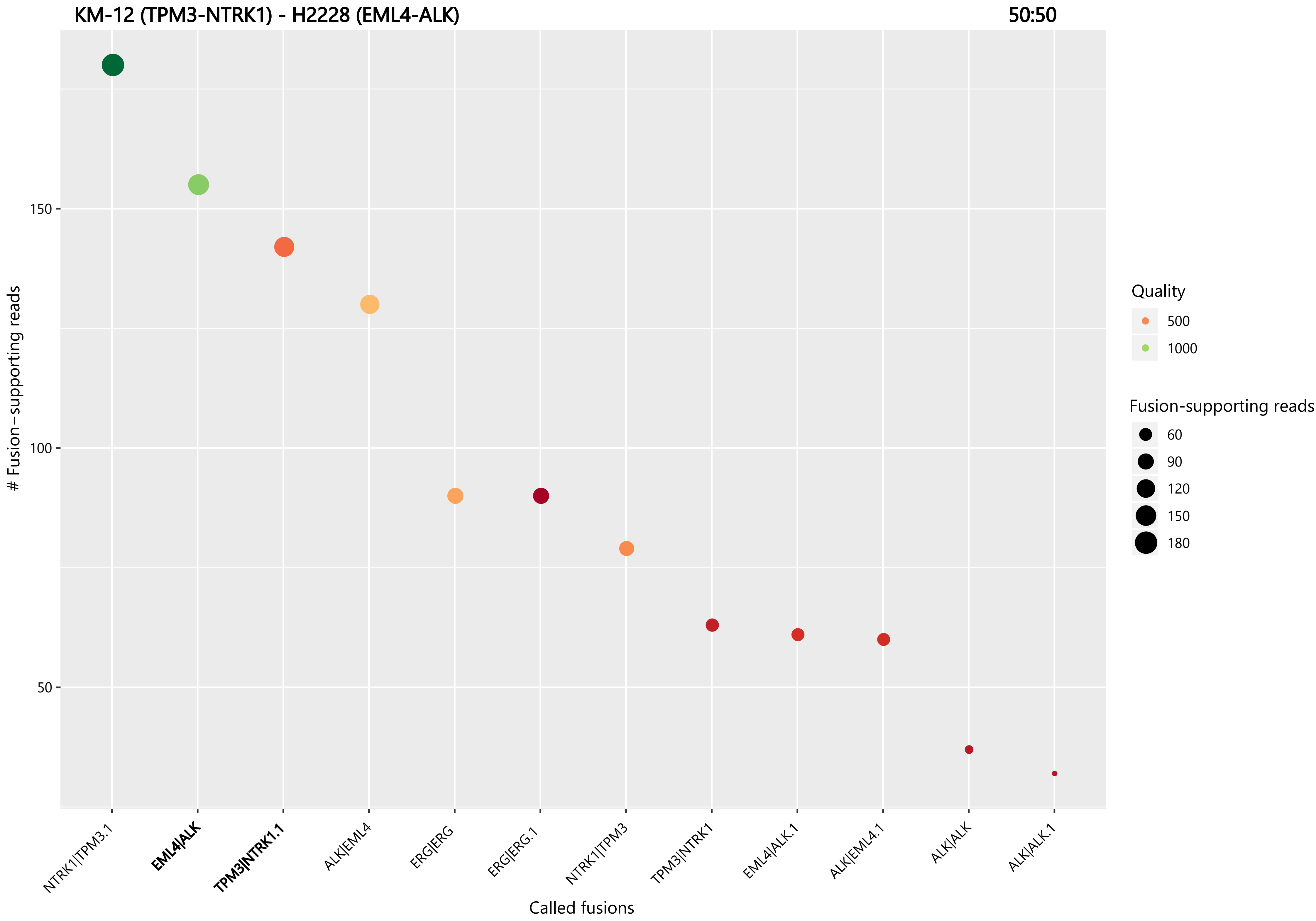
KM-12 (TPM3-NTRK1) - H2228 (EML4-ALK)
50:50
Fusion−supporting reads
150
100
50
Called fusions
NTRK1|TPM3.1
EML4|ALK
TPM3|NTRK1.1
ALK|EML4
ERG|ERG
ERG|ERG.1
NTRK1|TPM3
TPM3|NTRK1
EML4|ALK.1
ALK|EML4.1
ALK|ALK
ALK|ALK.1
Quality
500
1000
Fusion-supporting reads
60
90
120
150
180

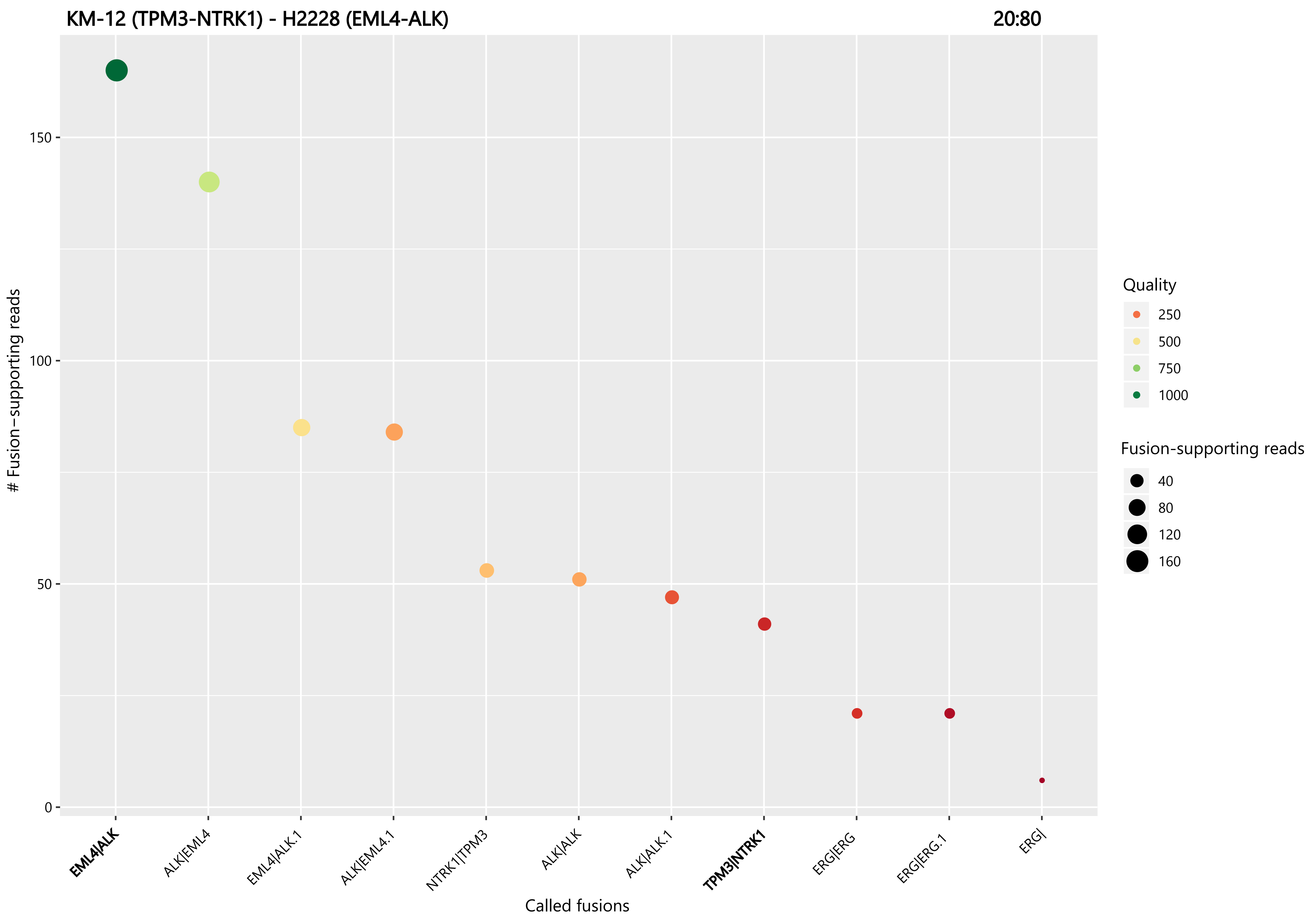

KM-12 (TPM3-NTRK1) - H2228 (EML4-ALK)
20:80
Fusion−supporting reads
150
100
50
0
EML4|ALK
ALK|EML4
EML4|ALK.1
ALK|EML4.1
NTRK1|TPM3
ALK|ALK
ALK|ALK.1
TPM3|NTRK1
ERG|ERG
ERG|ERG.1
ERG|
Called fusions
Quality
250
500
750
1000
Fusion-supporting reads
40
80
120
160

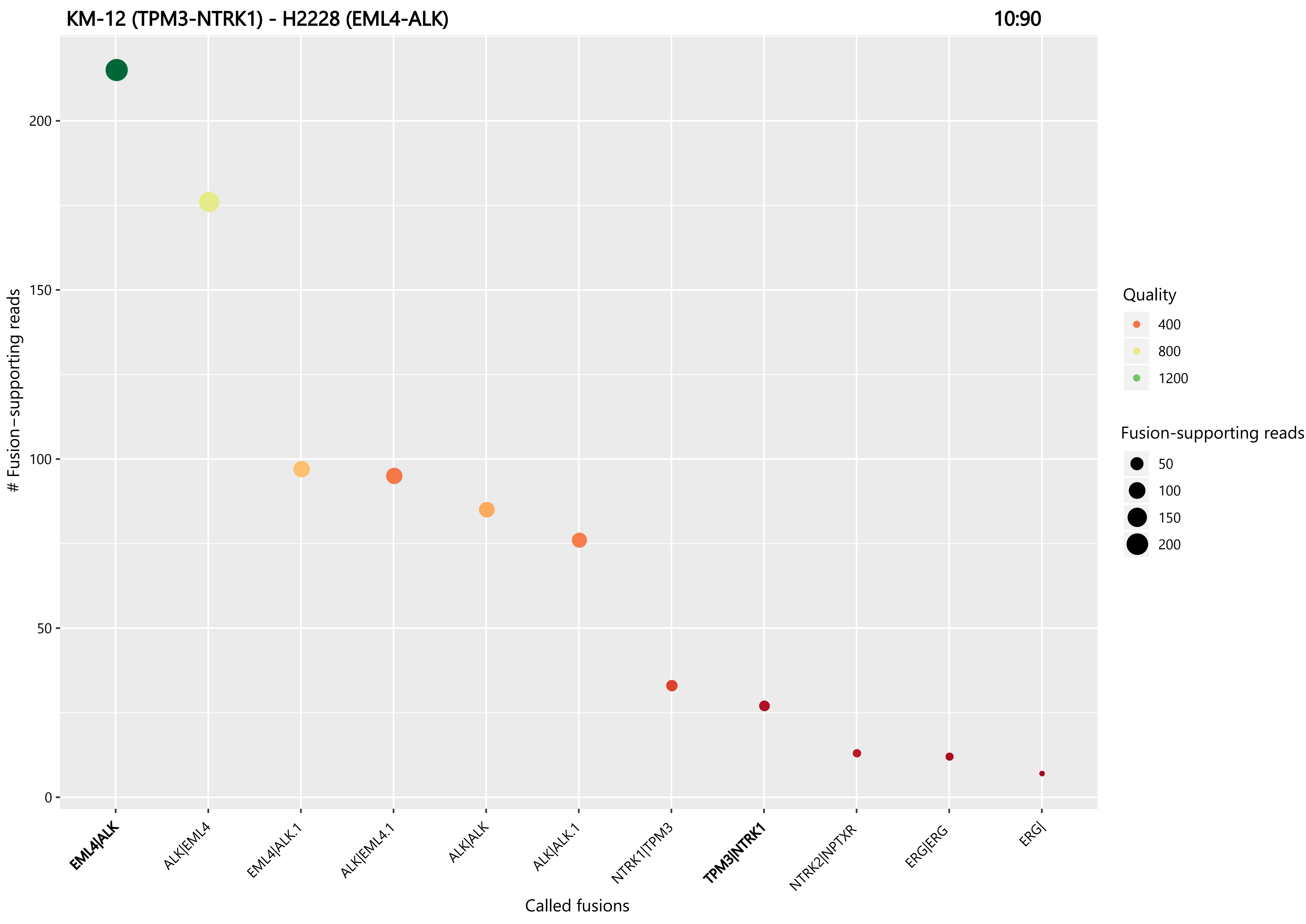
KM-12 (TPM3-NTRK1) - H2228 (EML4-ALK)
10:90
Fusion−supporting reads
0
50
100
150
200
Called fusions
EML4|ALK
ALK|EML4
EML4|ALK.1
ALK|EML4.1
ALK|ALK
ALK|ALK.1
NTRK1|TPM3
TPM3|NTRK1
NTRK2|NPTXR
ERG|ERG
ERG|
Quality
400
800
1200
Fusion-supporting reads
50
100
150
200

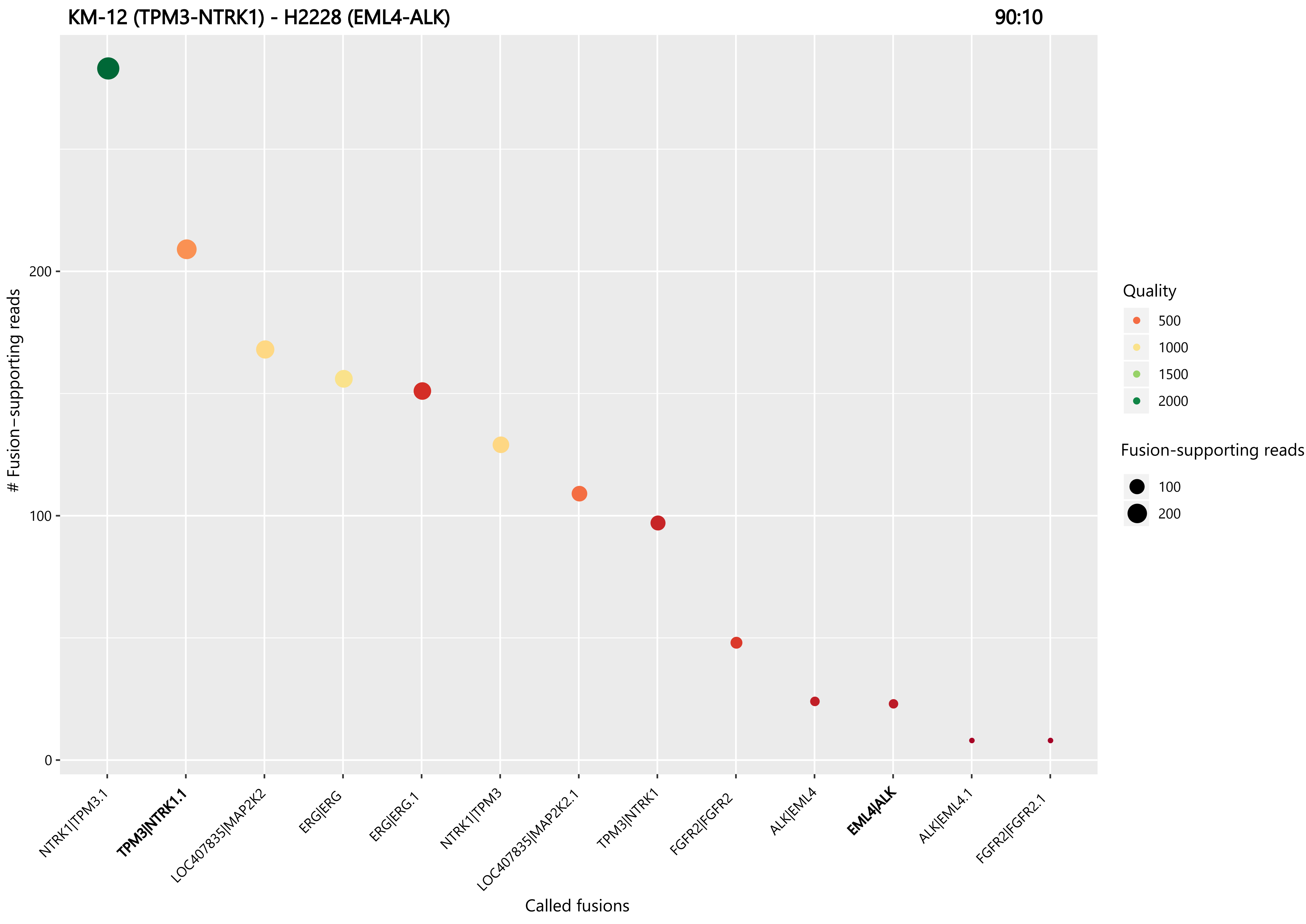

KM-12 (TPM3-NTRK1) - H2228 (EML4-ALK)
90:10
Fusion−supporting reads
0
100
200
Called fusions
NTRK1|TPM3.1
TPM3|NTRK1.1
LOC407835|MAP2K2
ERG|ERG
ERG|ERG.1
NTRK1|TPM3
LOC407835|MAP2K2.1
TPM3|NTRK1
FGFR2|FGFR2
ALK|EML4
EML4|ALK
ALK|EML4.1
FGFR2|FGFR2.1
Quality
500
1000
1500
2000
Fusion-supporting reads
100
200

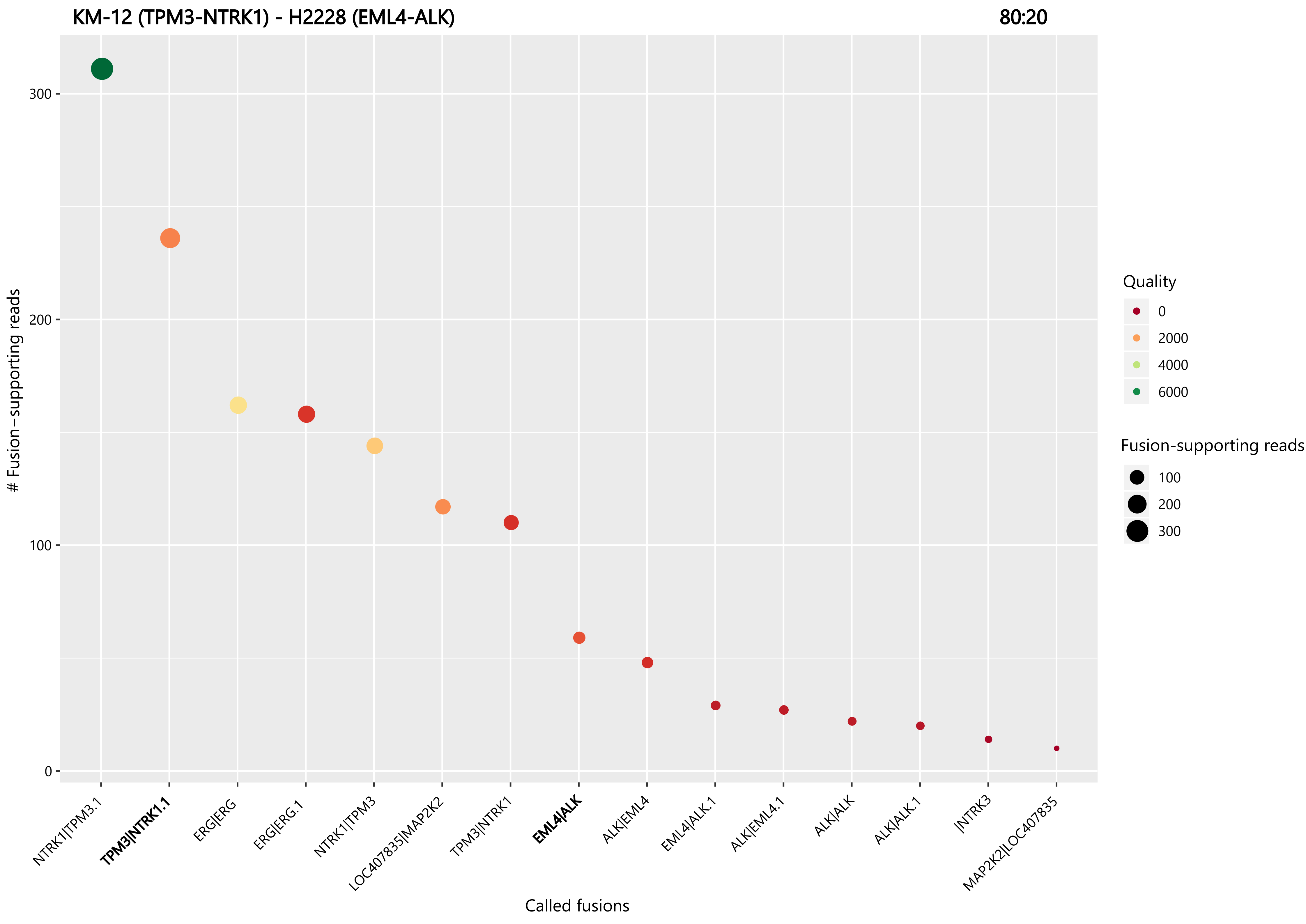
KM-12 (TPM3-NTRK1) - H2228 (EML4-ALK)
80:20
Fusion−supporting reads
300
200
100
0
NTRK1|TPM3.1
TPM3|NTRK1.1
ERG|ERG
ERG|ERG.1
NTRK1|TPM3
LOC407835|MAP2K2
TPM3|NTRK1
EML4|ALK
ALK|EML4
EML4|ALK.1
ALK|EML4.1
ALK|ALK
ALK|ALK.1
|NTRK3
MAP2K2|LOC407835
Called fusions
Quality
0
2000
4000
6000
Fusion-supporting reads
100
200
300

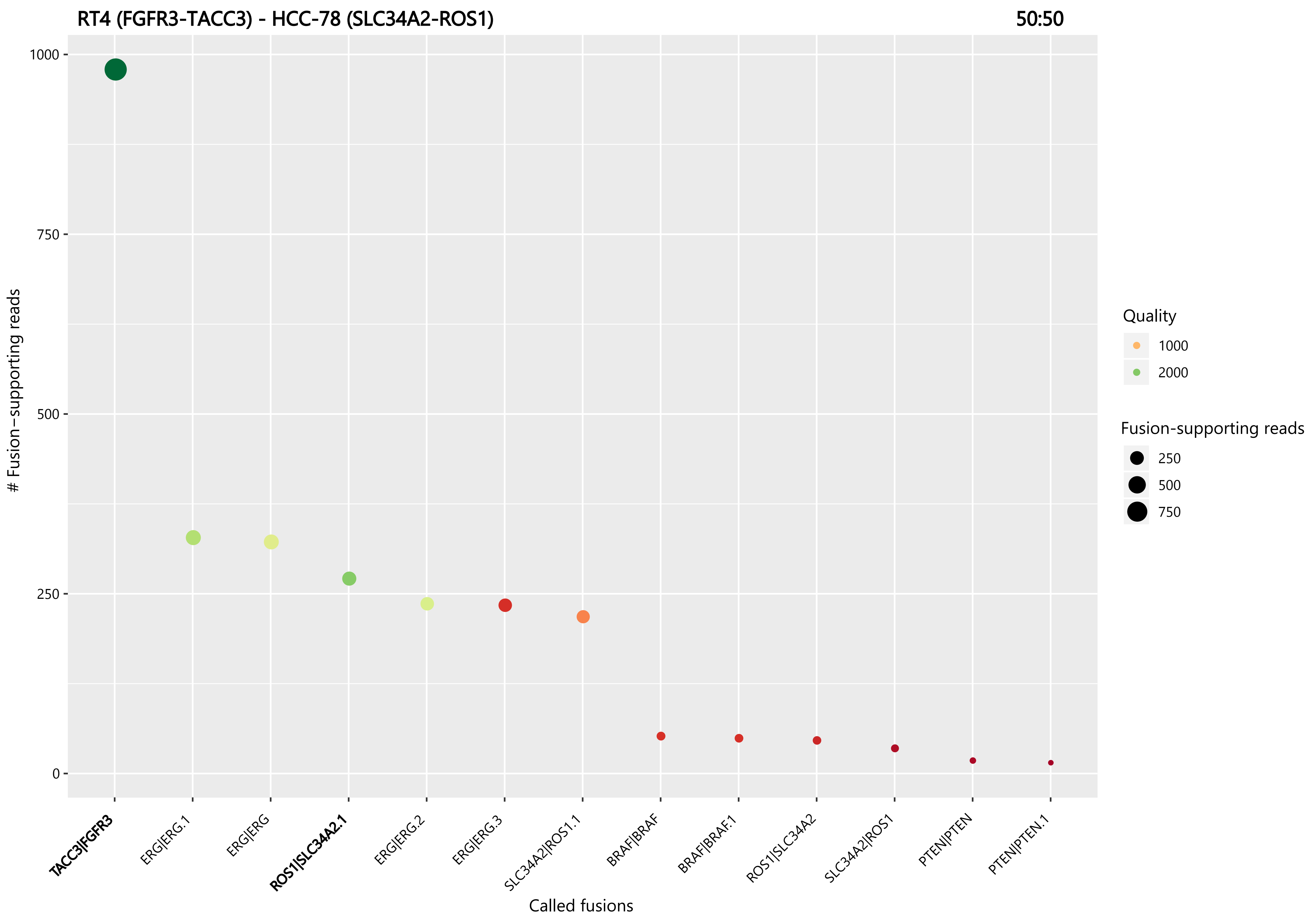
RT4 (FGFR3-TACC3) - HCC-78 (SLC34A2-ROS1)
50:50
1000
750
500
250
0
Fusion−supporting reads
TACC3|FGFR3
ERG|ERG.1
ERG|ERG
ROS1|SLC34A2.1
ERG|ERG.2
ERG|ERG.3
SLC34A2|ROS1.1
BRAF|BRAF
BRAF|BRAF.1
ROS1|SLC34A2
SLC34A2|ROS1
PTEN|PTEN
PTEN|PTEN.1
Called fusions
Quality
1000
2000
Fusion−supporting reads
250
500
750

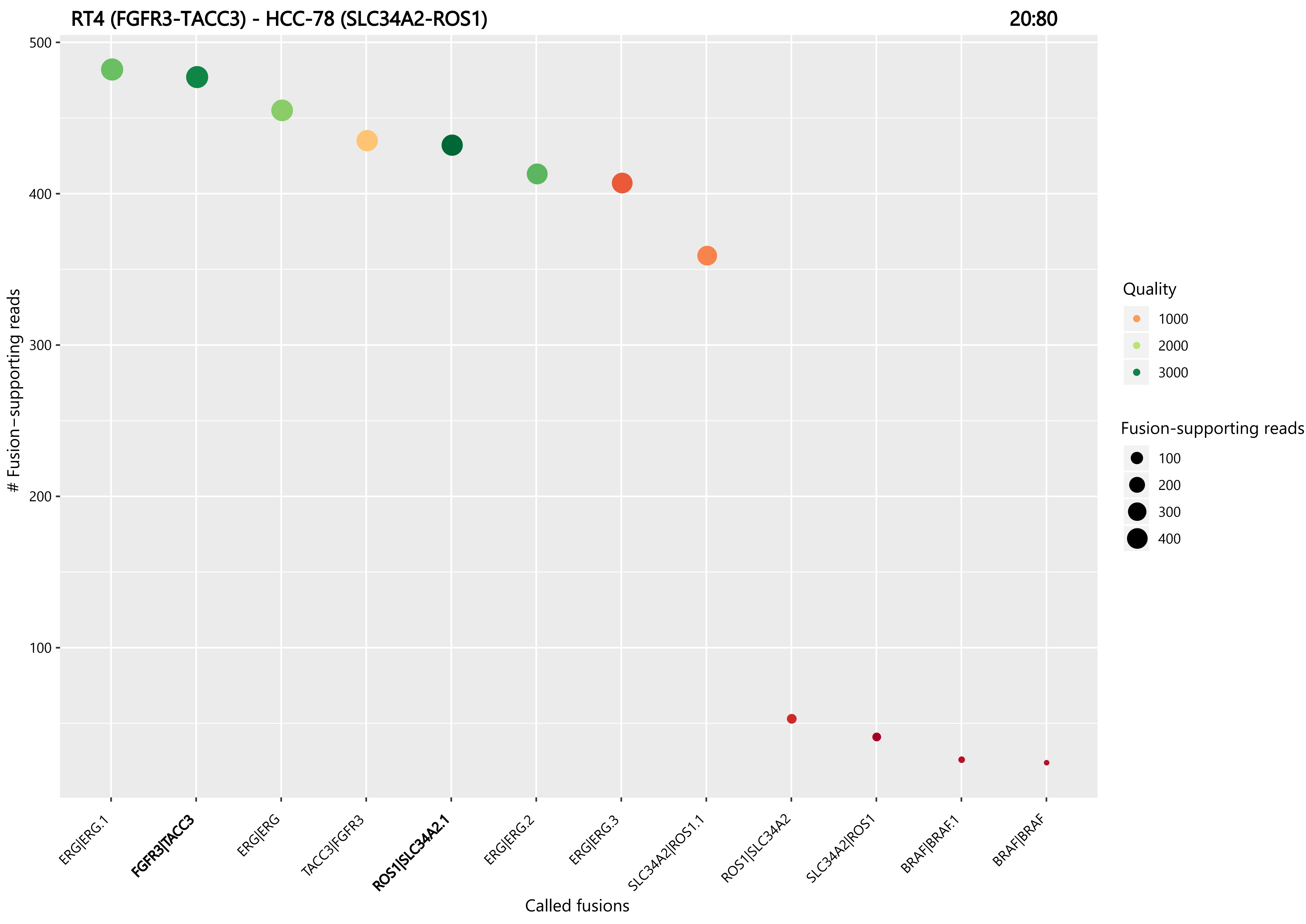

RT4 (FGFR3-TACC3) - HCC-78 (SLC34A2-ROS1)
20:80
Fusion−supporting reads
500
400
300
200
100
Called fusions
ERG|ERG.1
FGFR3|TACC3
ERG|ERG
TACC3|FGFR3
ROS1|SLC34A2.1
ERG|ERG.2
ERG|ERG.3
SLC34A2|ROS1.1
ROS1|SLC34A2
SLC34A2|ROS1
BRAF|BRAF.1
BRAF|BRAF
Quality
1000
2000
3000
Fusion-supporting reads
100
200
300
400

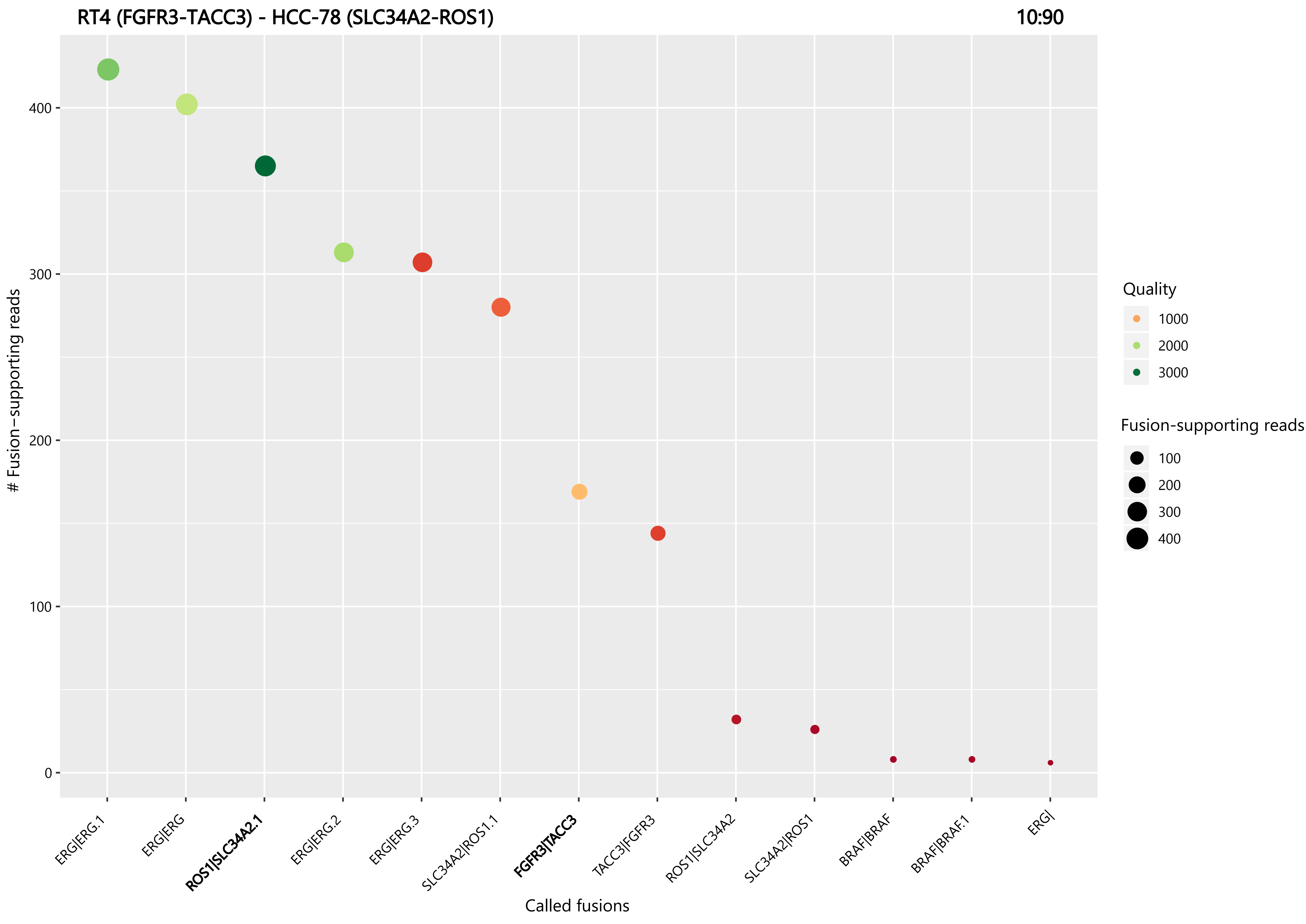
RT4 (FGFR3-TACC3) - HCC-78 (SLC34A2-ROS1)
10:90
Fusion−supporting reads
400
300
200
100
0
Called fusions
ERG|ERG.1
ERG|ERG
ROS1|SLC34A2.1
ERG|ERG.2
ERG|ERG.3
SLC34A2|ROS1.1
FGFR3|TACC3
TACC3|FGFR3
ROS1|SLC34A2
SLC34A2|ROS1
BRAF|BRAF
BRAF|BRAF.1
ERG|
Quality
1000
2000
3000
Fusion-supporting reads
100
200
300
400

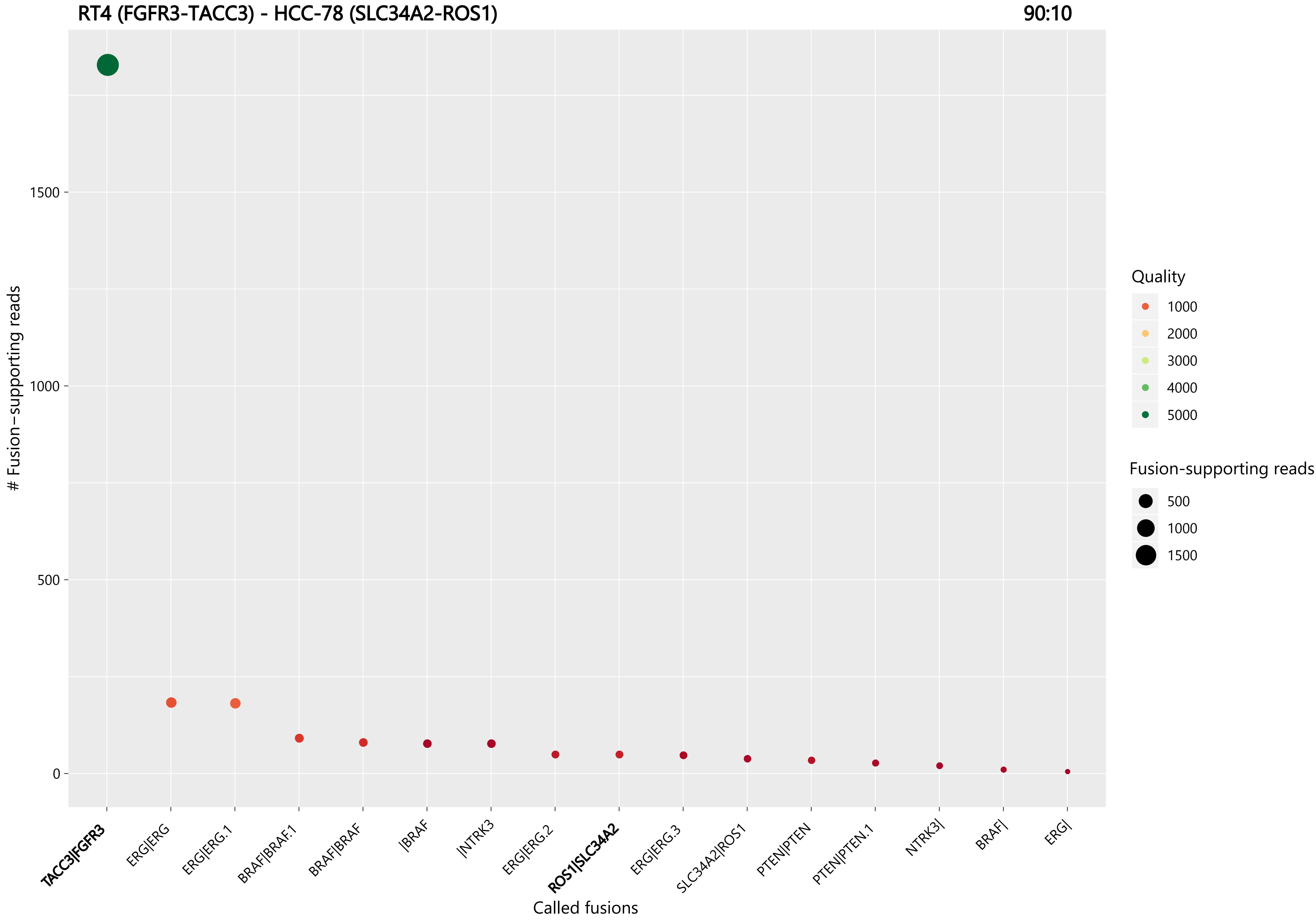

RT4 (FGFR3-TACC3) - HCC-78 (SLC34A2-ROS1)
90:10
Fusion−supporting reads
1500
1000
500
0
Called fusions
TACC3|FGFR3
ERG|ERG
ERG|ERG.1
BRAF|BRAF.1
BRAF|BRAF
|BRAF
|NTRK3
ERG|ERG.2
ROS1|SLC34A2
ERG|ERG.3
SLC34A2|ROS1
PTEN|PTEN
PTEN|PTEN.1
NTRK3|
BRAF|
ERG|
Quality
1000
2000
3000
4000
5000
Fusion-supporting reads
500
1000
1500

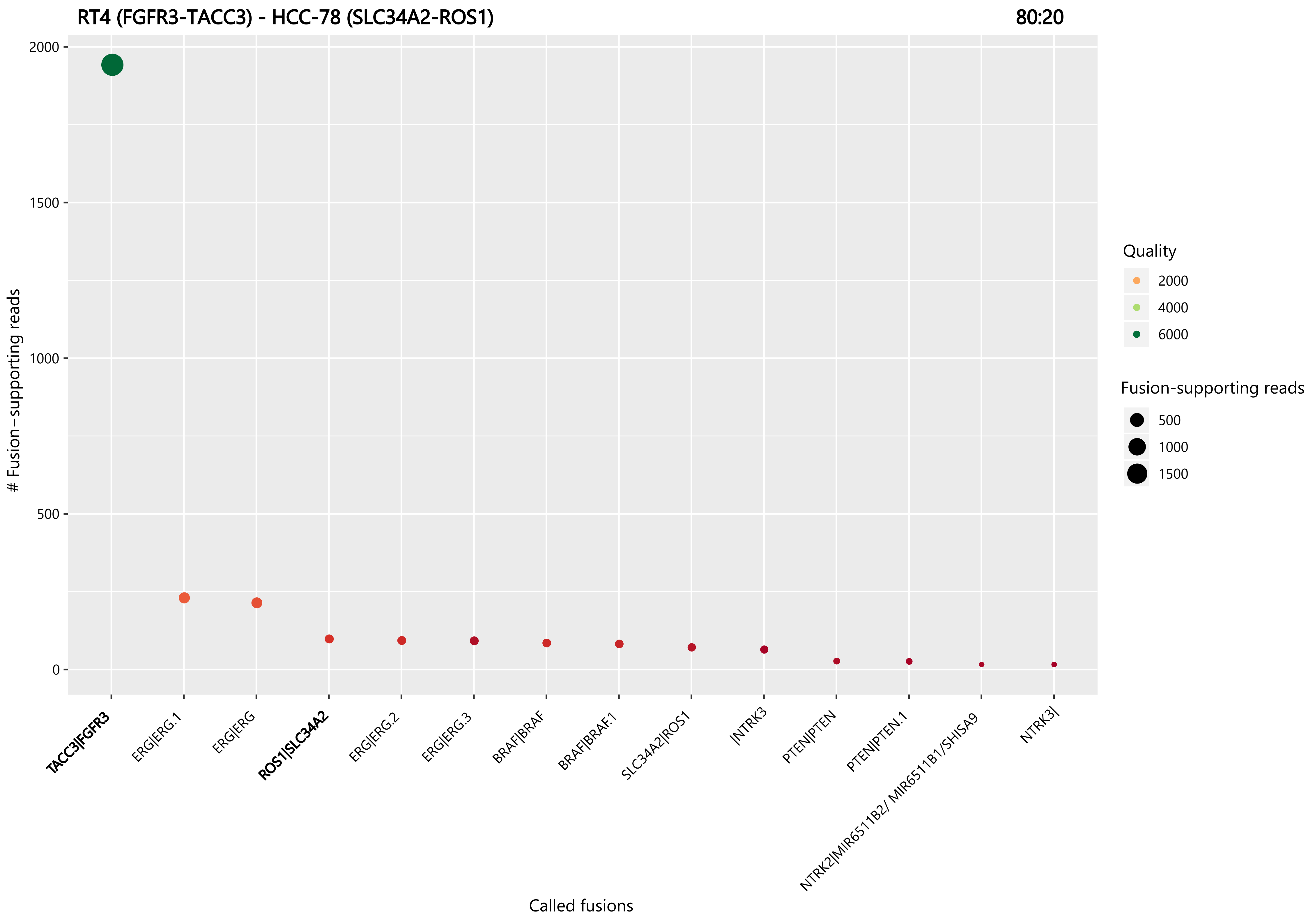

RT4 (FGFR3-TACC3) - HCC-78 (SLC34A2-ROS1)
80:20
Fusion−supporting reads
2000
1500
1000
500
0
Quality
2000
4000
6000
Fusion-supporting reads
500
1000
1500
TACC3|FGFR3
ERG|ERG.1
ERG|ERG
ROS1|SLC34A2
ERG|ERG.2
ERG|ERG.3
BRAF|BRAF
BRAF|BRAF.1
SLC34A2|ROS1
|NTRK3
PTEN|PTEN
PTEN|PTEN.1
NTRK2|MIR6511B2/ MIR6511B1/SHISA9
NTRK3|
Called fusions

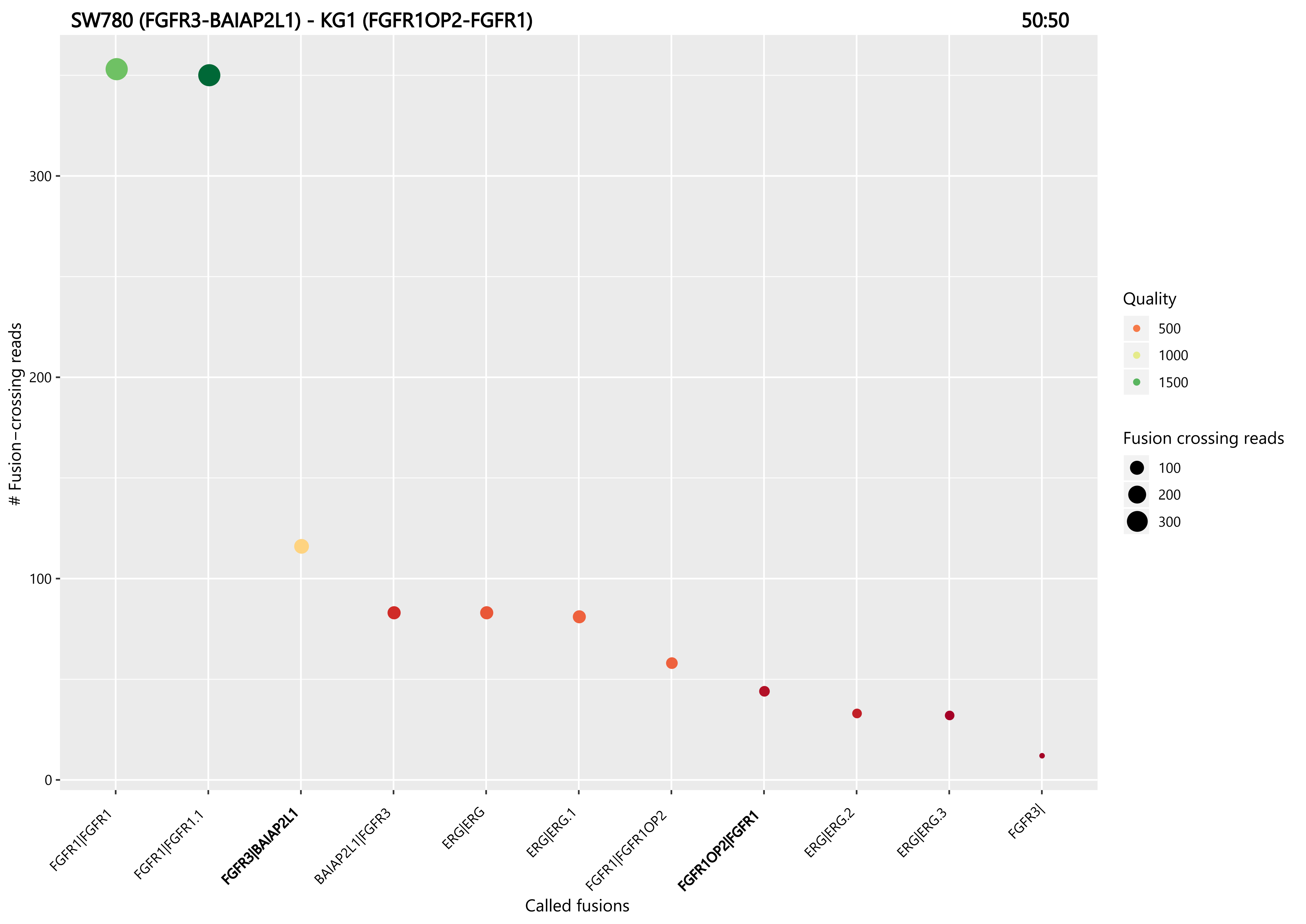

SW780 (FGFR3-BAIAP2L1) - KG1 (FGFR1OP2-FGFR1)
50:50
Fusion−crossing reads
300
200
100
0
FGFR1|FGFR1
FGFR1|FGFR1.1
FGFR3|BAIAP2L1
BAIAP2L1|FGFR3
ERG|ERG
ERG|ERG.1
FGFR1|FGFR1OP2
FGFR1OP2|FGFR1
ERG|ERG.2
ERG|ERG.3
FGFR3|
Called fusions
Quality
500
1000
1500
Fusion crossing reads
100
200
300

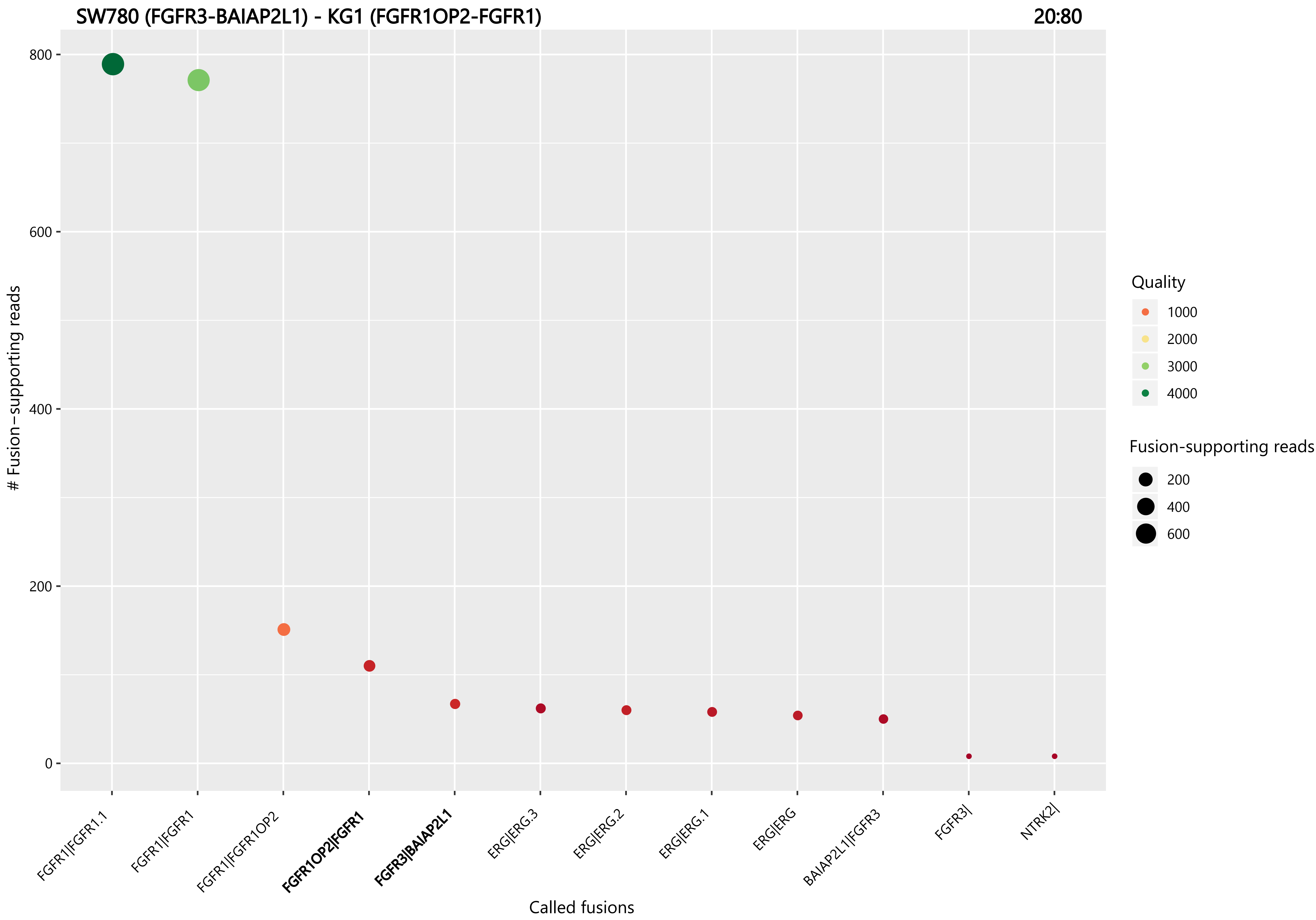
SW780 (FGFR3-BAIAP2L1) - KG1 (FGFR1OP2-FGFR1)
20:80
Fusion−supporting reads
800
600
400
200
0
FGFR1|FGFR1.1
FGFR1|FGFR1
FGFR1|FGFR1OP2
FGFR1OP2|FGFR1
FGFR3|BAIAP2L1
ERG|ERG.3
ERG|ERG.2
ERG|ERG.1
ERG|ERG
BAIAP2L1|FGFR3
FGFR3|
NTRK2|
Called fusions
Quality
1000
2000
3000
4000
Fusion-supporting reads
200
400
600

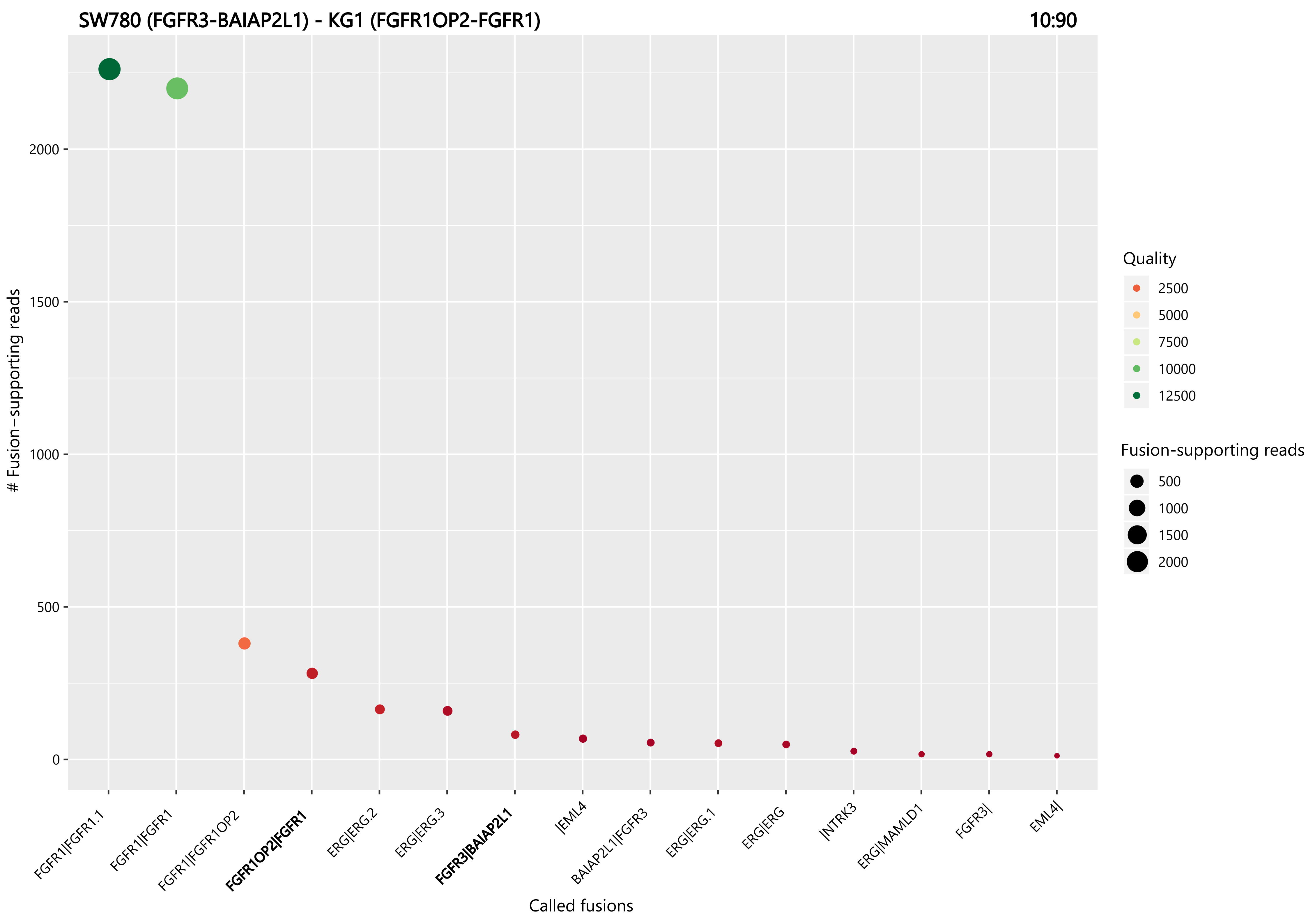
SW780 (FGFR3-BAIAP2L1) - KG1 (FGFR1OP2-FGFR1)
10:90
Fusion−supporting reads
2000
1500
1000
500
0
FGFR1|FGFR1.1
FGFR1|FGFR1
FGFR1|FGFR1OP2
FGFR1OP2|FGFR1
ERG|ERG.2
ERG|ERG.3
FGFR3|BAIAP2L1
|EML4
BAIAP2L1|FGFR3
ERG|ERG.1
ERG|ERG
|NTRK3
ERG|MAMLD1
FGFR3|
EML4|
Called fusions
Quality
2500
5000
7500
10000
12500
Fusion-supporting reads
500
1000
1500
2000

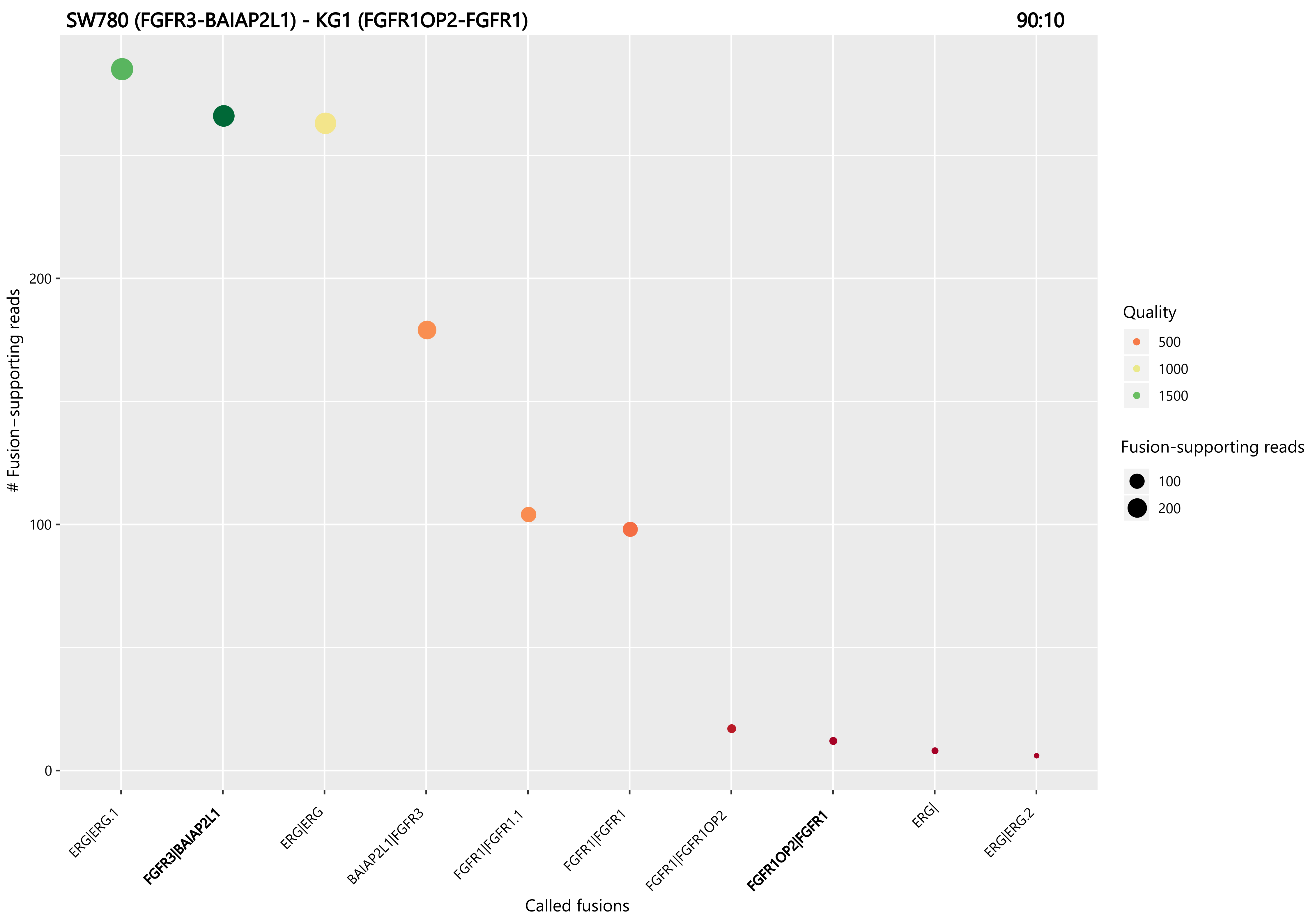
SW780 (FGFR3-BAIAP2L1) - KG1 (FGFR1OP2-FGFR1)
90:10
Fusion−supporting reads
0
100
200
Called fusions
ERG|ERG.1
FGFR3|BAIAP2L1
ERG|ERG
BAIAP2L1|FGFR3
FGFR1|FGFR1.1
FGFR1|FGFR1
FGFR1|FGFR1OP2
FGFR1OP2|FGFR1
ERG|
ERG|ERG.2
Quality
500
1000
1500
Fusion-supporting reads
100
200

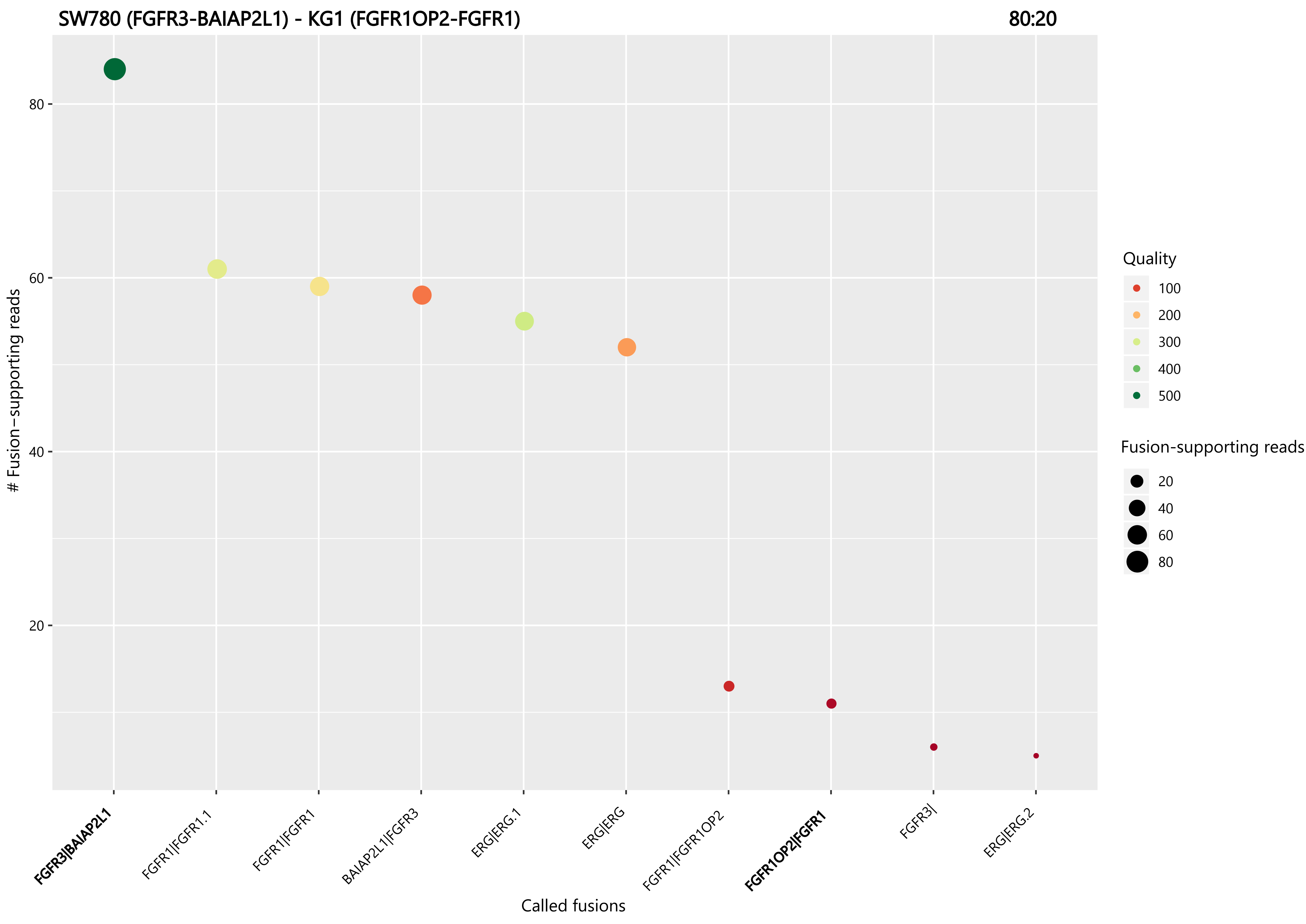

SW780 (FGFR3-BAIAP2L1) - KG1 (FGFR1OP2-FGFR1)
80:20
Fusion−supporting reads
80
60
40
20
FGFR3|BAIAP2L1
FGFR1|FGFR1.1
FGFR1|FGFR1
BAIAP2L1|FGFR3
ERG|ERG.1
ERG|ERG
FGFR1|FGFR1OP2
FGFR1OP2|FGFR1
FGFR3|
ERG|ERG.2
Called fusions
Quality
100
200
300
400
500
Fusion-supporting reads
20
40
60
80

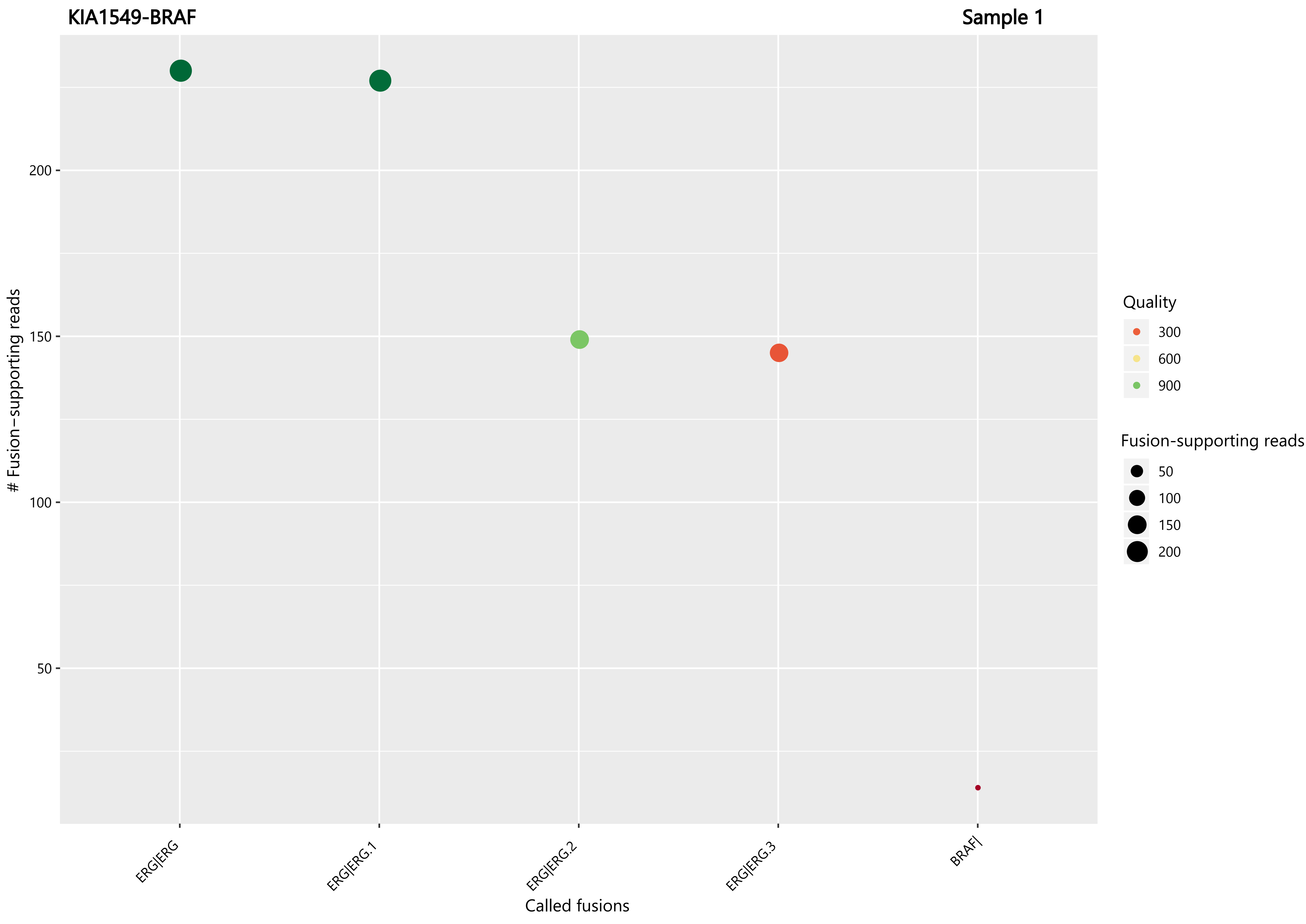

KIA1549-BRAF
Sample 1
Fusion−supporting reads
200
150
100
50
ERG|ERG
ERG|ERG.1
ERG|ERG.2
ERG|ERG.3
BRAF|
Called fusions
Quality
300
600
900
Fusion-supporting reads
50
100
150
200

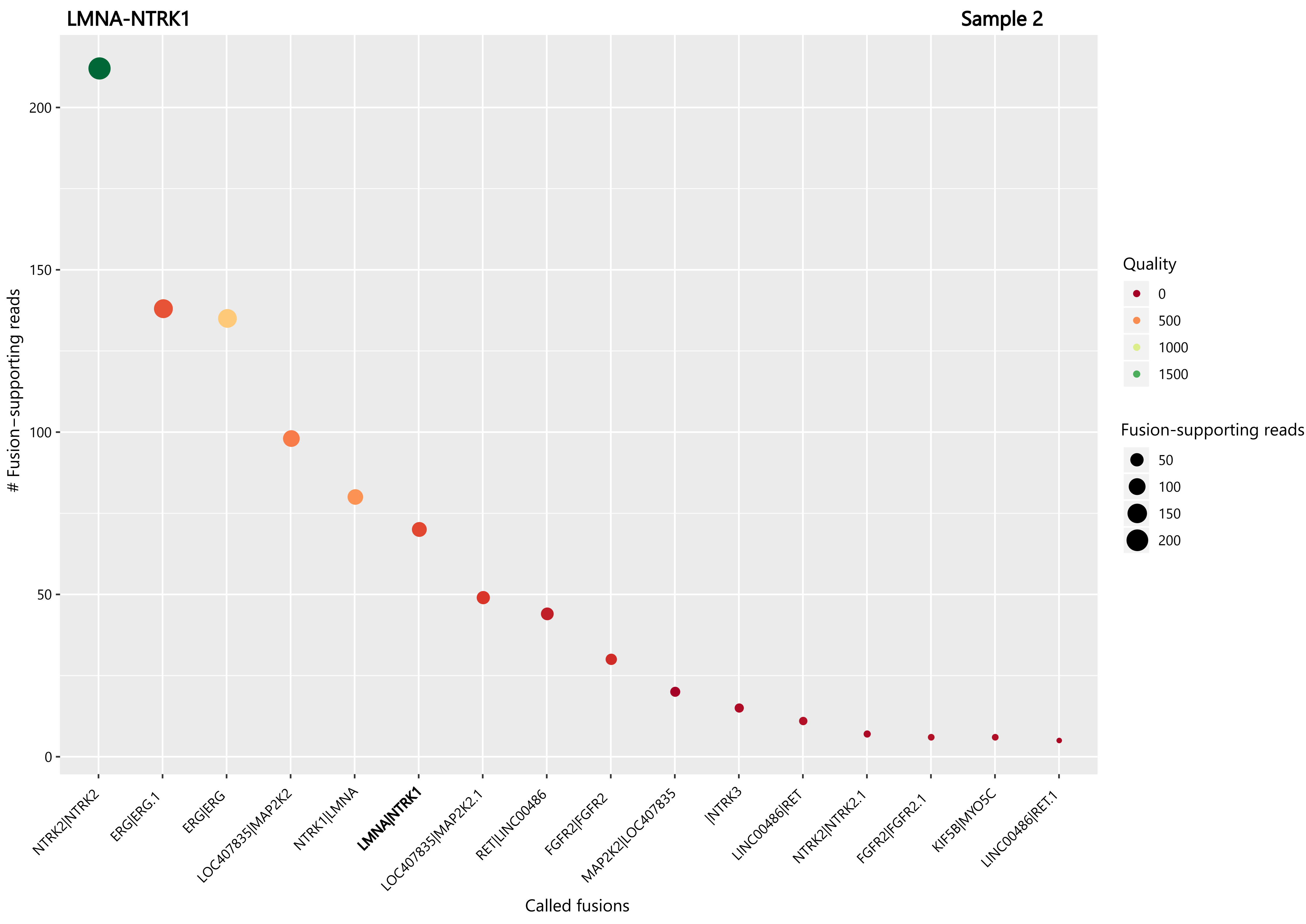
LMNA-NTRK1
Sample 2
Fusion−supporting reads
0
50
100
150
200
Called fusions
NTRK2|NTRK2
ERG|ERG.1
ERG|ERG
LOC407835|MAP2K2
NTRK1|LMNA
LMNA|NTRK1
LOC407835|MAP2K2.1
RET|LINC00486
FGFR2|FGFR2
MAP2K2|LOC407835
|NTRK3
LINC00486|RET
NTRK2|NTRK2.1
FGFR2|FGFR2.1
KIF5B|MYO5C
LINC00486|RET.1
Quality
0
500
1000
1500
Fusion-supporting reads
50
100
150
200

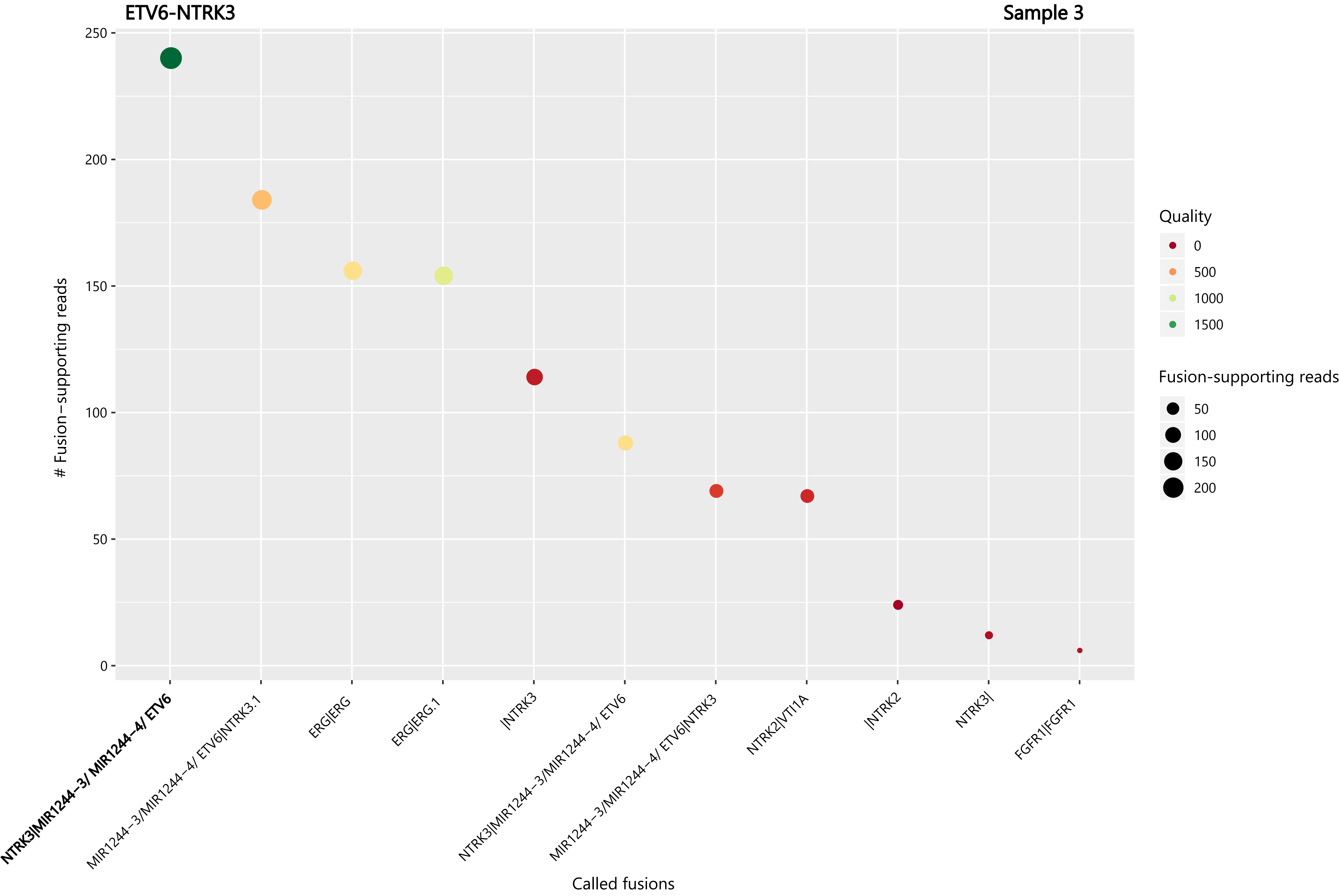

ETV6-NTRK3
Sample 3
Fusion−supporting reads
250
200
150
100
50
0
NTRK3|MIR1244−3/ MIR1244−4/ ETV6
MIR1244−3/MIR1244−4/ ETV6|NTRK3.1
ERG|ERG
ERG|ERG.1
|NTRK3
NTRK3|MIR1244−3/MIR1244−4/ ETV6
MIR1244−3/MIR1244−4/ ETV6|NTRK3
NTRK2|VTI1A
|NTRK2
NTRK3|
FGFR1|FGFR1
Called fusions
Quality
0
500
1000
1500
Fusion-supporting reads
50
100
150
200

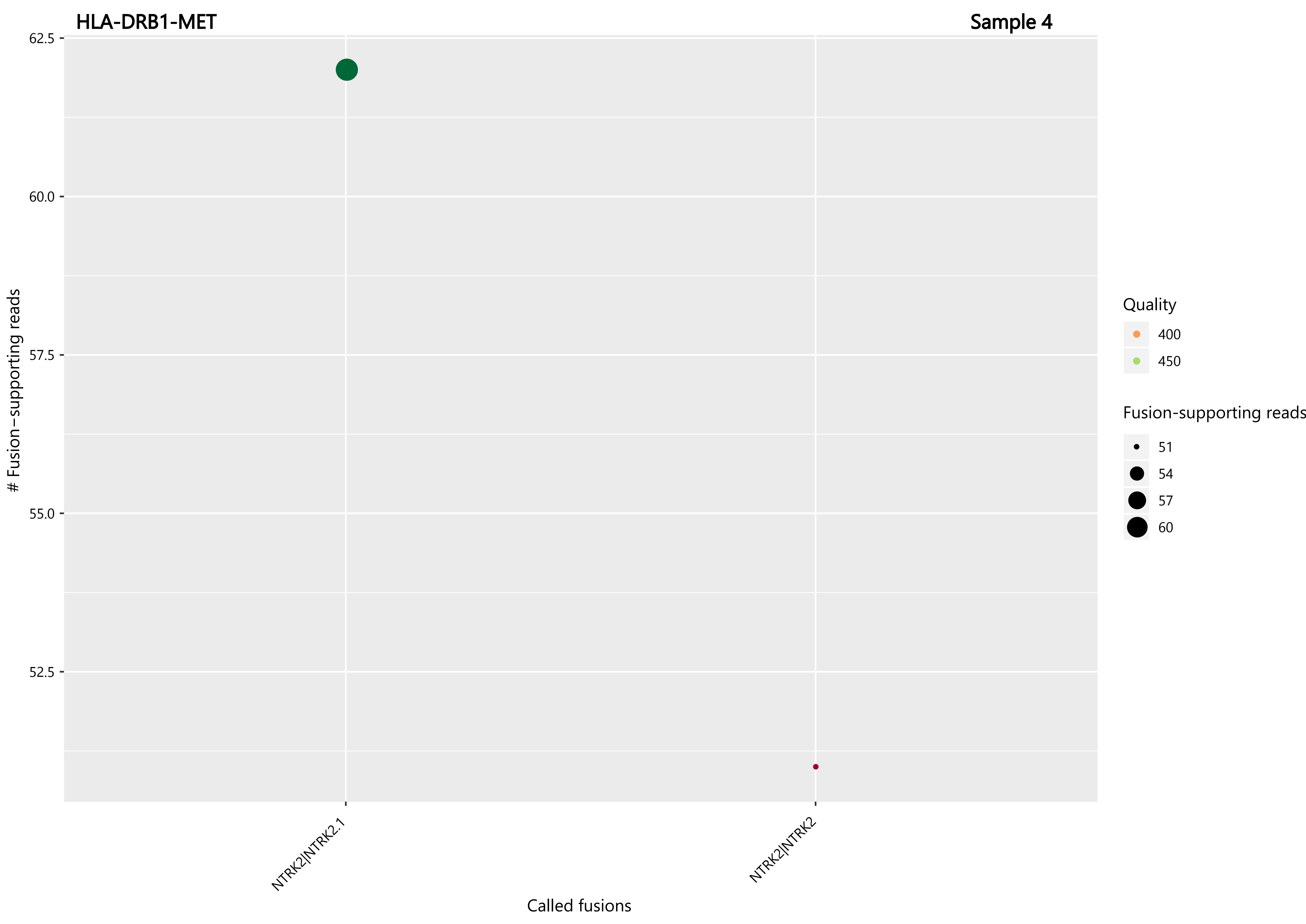

HLA-DRB1-MET
Sample 4
62.5
60.0
57.5
55.0
52.5
Fusion−supporting reads
NTRK2|NTRK2.1
NTRK2|NTRK2
Called fusions
Quality
400
450
Fusion-supporting reads
51
54
57
60

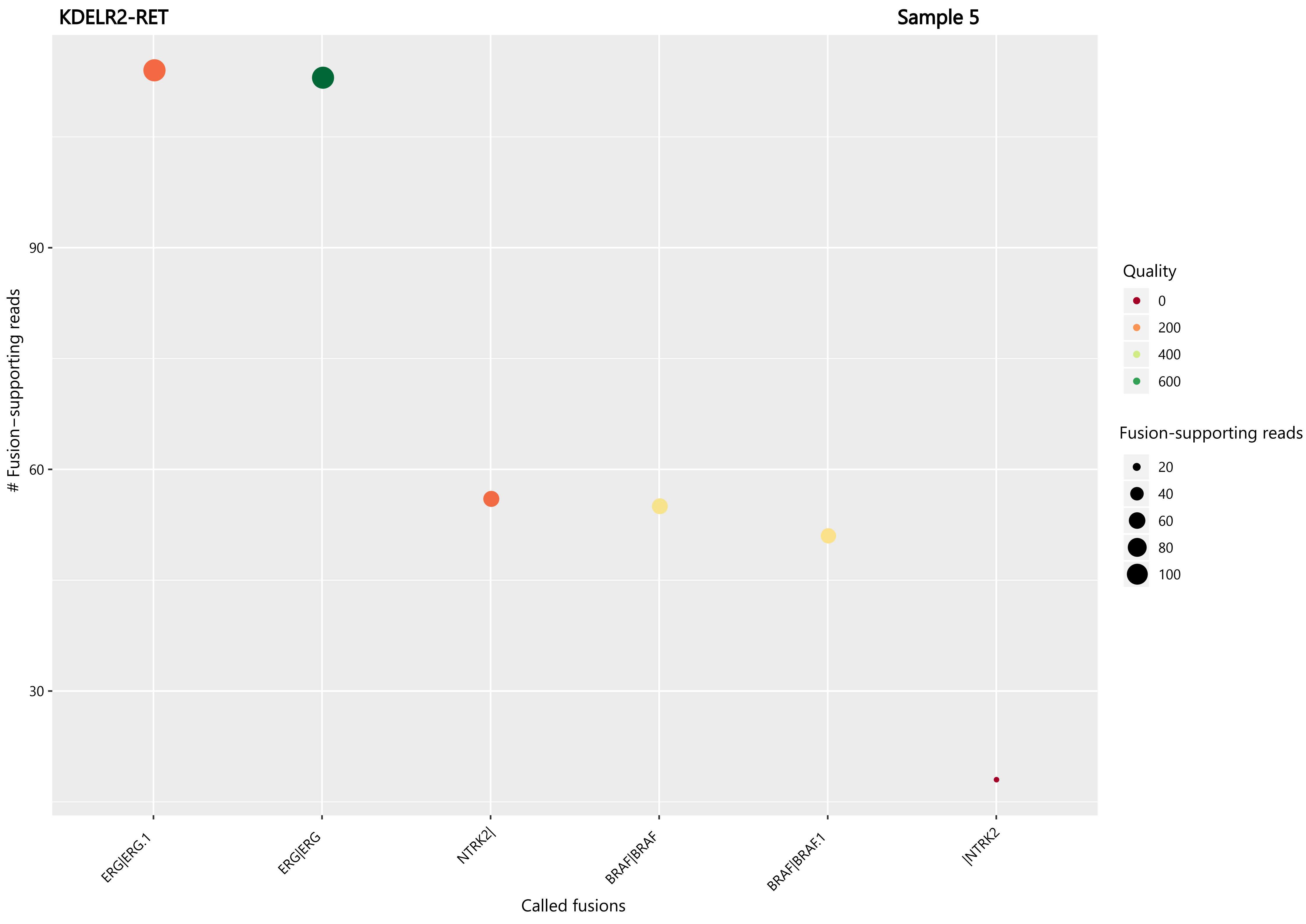

KDELR2-RET
Sample 5
Fusion−supporting reads
90
60
30
ERG|ERG.1
ERG|ERG
NTRK2|
BRAF|BRAF
BRAF|BRAF.1
|NTRK2
Called fusions
Quality
0
200
400
600
Fusion-supporting reads
20
40
60
80
100

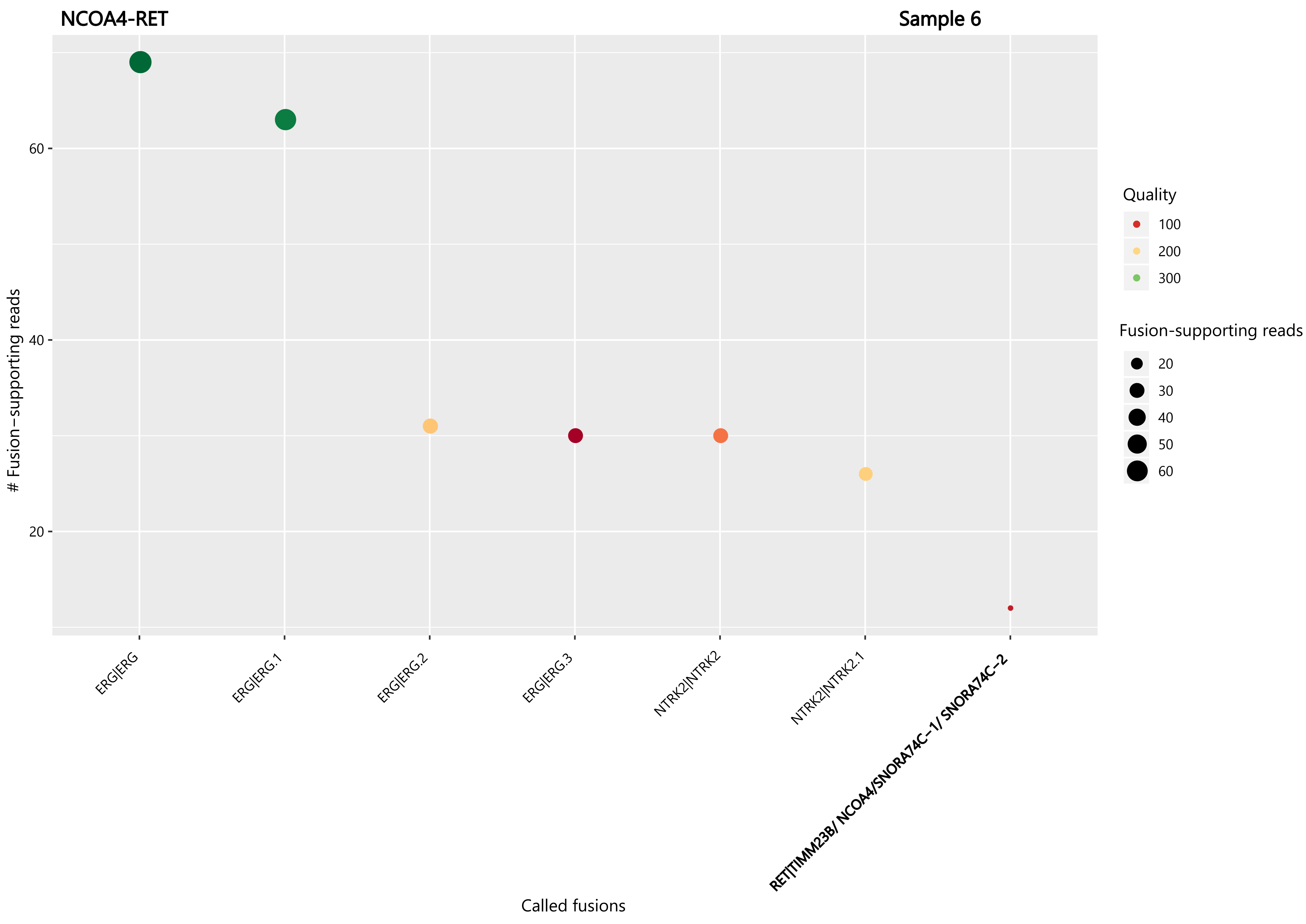

NCOA4-RET
Sample 6
Fusion−supporting reads
60
40
20
Called fusions
ERG|ERG
ERG|ERG.1
ERG|ERG.2
ERG|ERG.3
NTRK2|NTRK2
NTRK2|NTRK2.1
RET|TIMM23B/ NCOA4/SNORA74C−1/ SNORA74C−2
Quality
100
200
300
Fusion-supporting reads
20
30
40
50
60

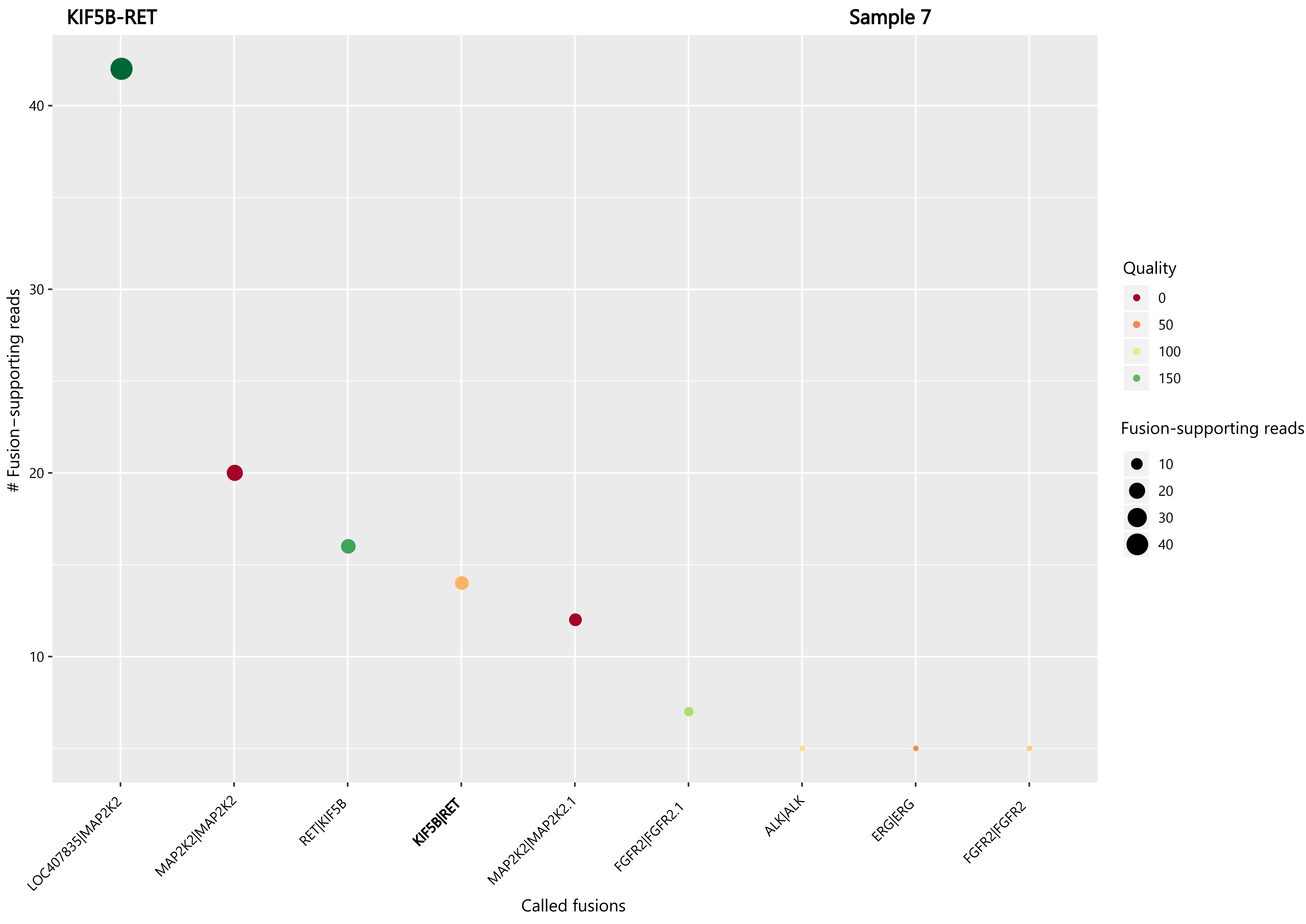
KIF5B-RET
Sample 7
Fusion−supporting reads
40
30
20
10
Called fusions
LOC407835|MAP2K2
MAP2K2|MAP2K2
RET|KIF5B
KIF5B|RET
MAP2K2|MAP2K2.1
FGFR2|FGFR2.1
ALK|ALK
ERG|ERG
FGFR2|FGFR2
Quality
0
50
100
150
Fusion-supporting reads
10
20
30
40

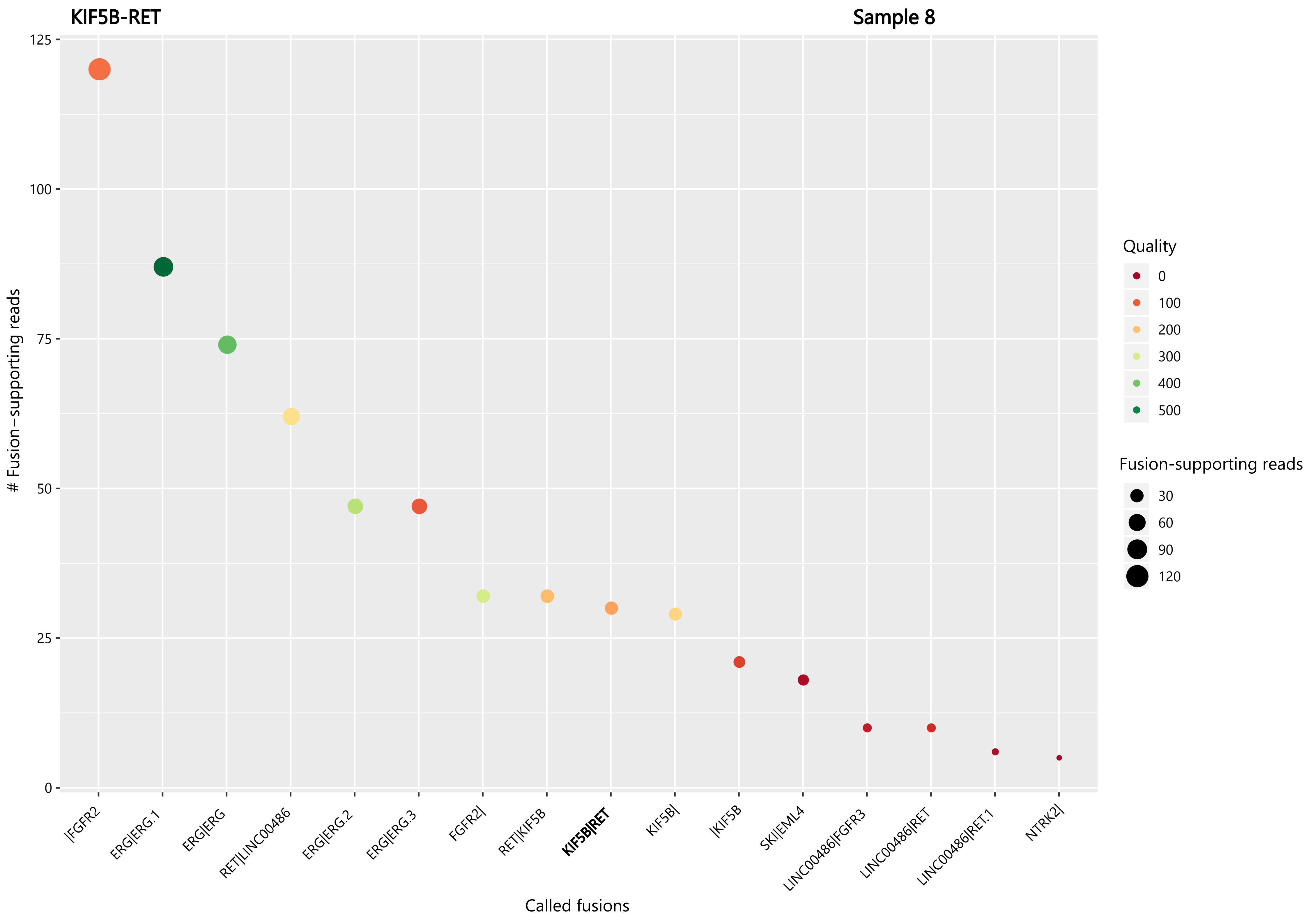

KIF5B-RET
Sample 8
125
100
75
50
25
0
Fusion−supporting reads
|FGFR2
ERG|ERG.1
ERG|ERG
RET|LINC00486
ERG|ERG.2
ERG|ERG.3
FGFR2|
RET|KIF5B
KIF5B|RET
KIF5B|
|KIF5B
SKI|EML4
LINC00486|FGFR3
LINC00486|RET
LINC00486|RET.1
NTRK2|
Called fusions
Quality
0
100
200
300
400
500
Fusion-supporting reads
30
60
90
120

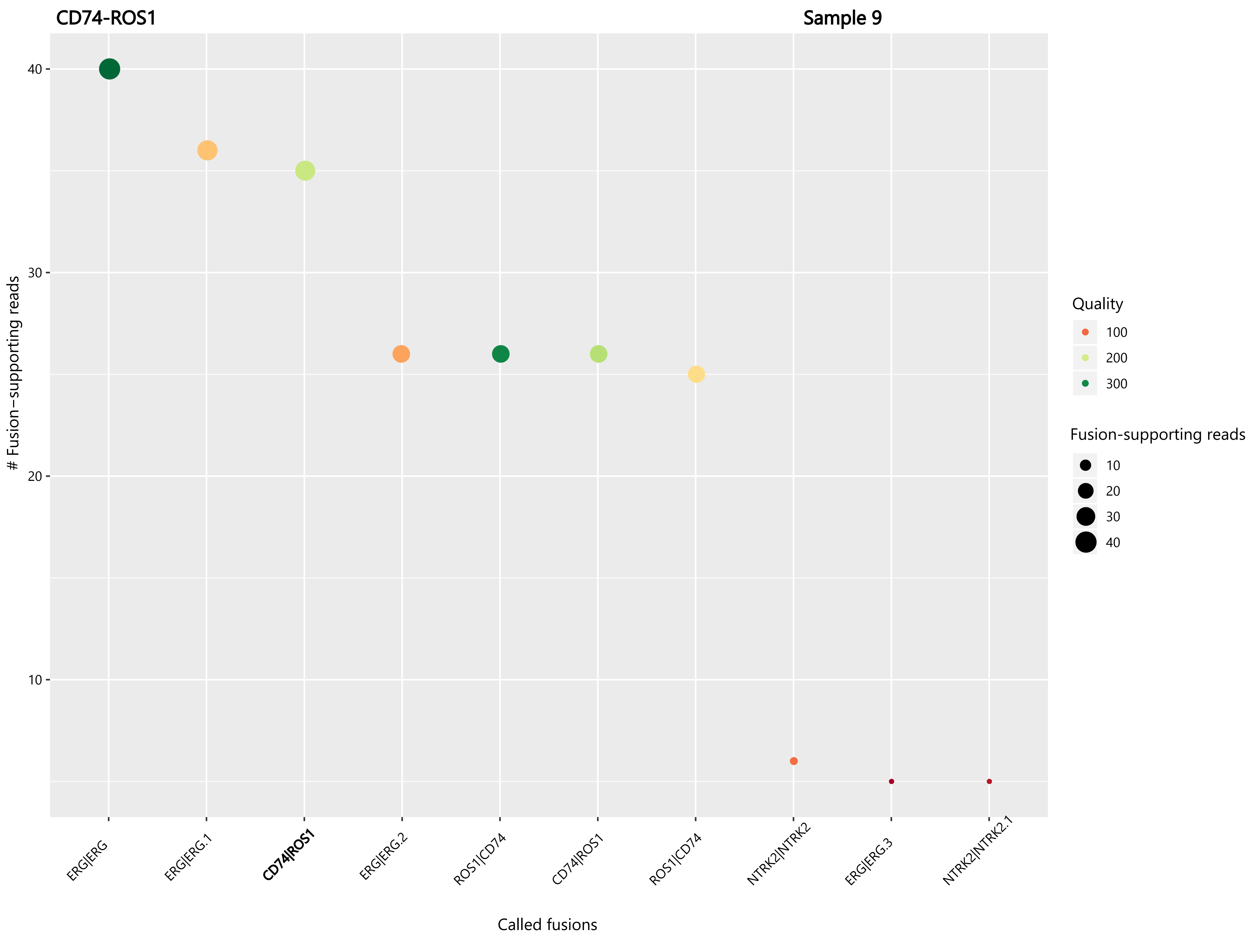

CD74-ROS1
Sample 9
Fusion−supporting reads
40
30
20
10
Called fusions
ERG|ERG
ERG|ERG.1
CD74|ROS1
ERG|ERG.2
ROS1|CD74
CD74|ROS1
ROS1|CD74
NTRK2|NTRK2
ERG|ERG.3
NTRK2|NTRK2.1
Quality
100
200
300
Fusion-supporting reads
10
20
30
40

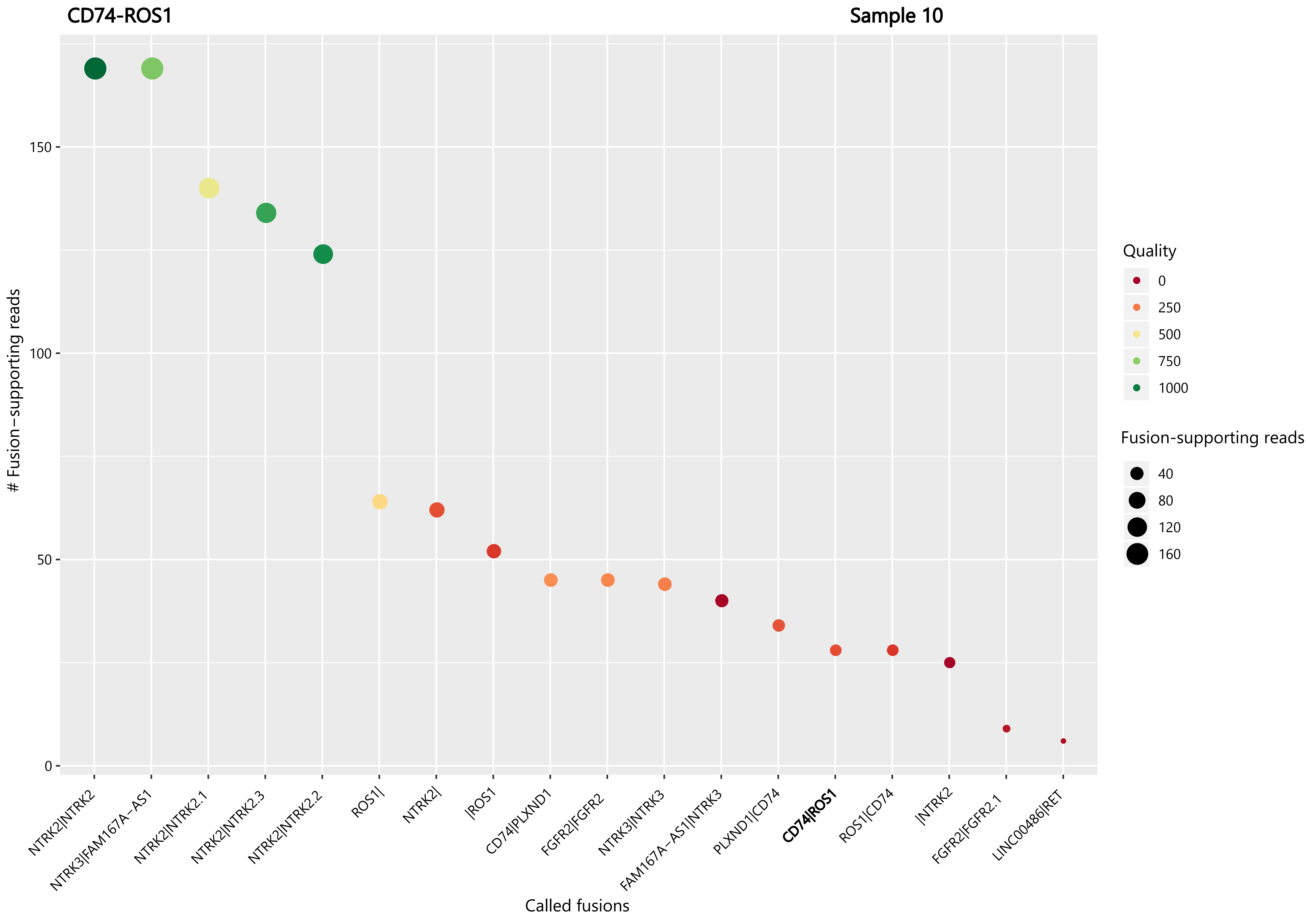

CD74-ROS1
Sample 10
Fusion−supporting reads
150
100
50
0
Called fusions
NTRK2|NTRK2
NTRK3|FAM167A−AS1
NTRK2|NTRK2.1
NTRK2|NTRK2.3
NTRK2|NTRK2.2
ROS1|
NTRK2|
|ROS1
CD74|PLXND1
FGFR2|FGFR2
NTRK3|NTRK3
FAM167A−AS1|NTRK3
PLXND1|CD74
CD74|ROS1
ROS1|CD74
|NTRK2
FGFR2|FGFR2.1
LINC00486|RET
Quality
0
250
500
750
1000
Fusion-supporting reads
40
80
120
160

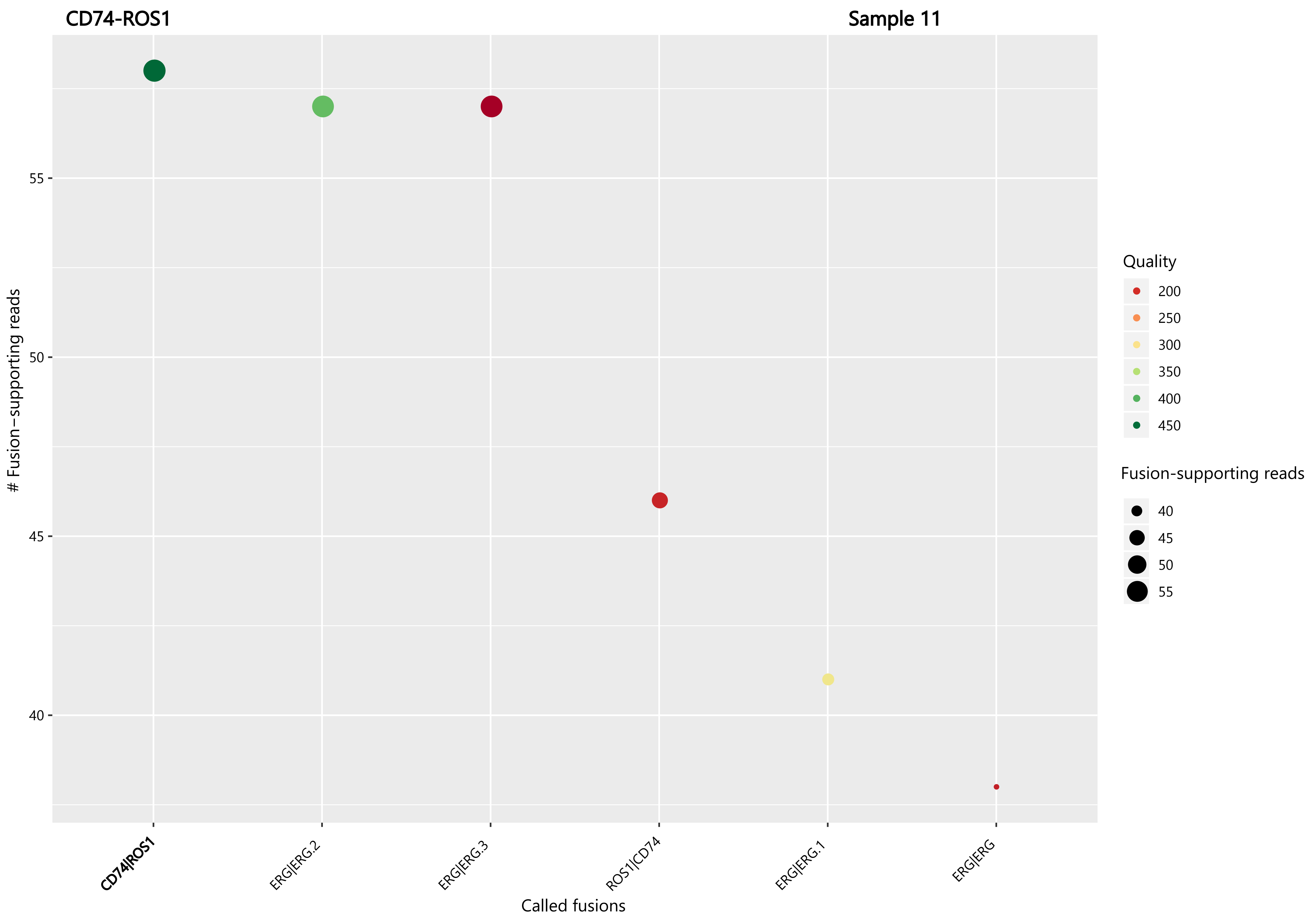

CD74-ROS1
Sample 11
Fusion−supporting reads
55
50
45
40
Called fusions
CD74|ROS1
ERG|ERG.2
ERG|ERG.3
ROS1|CD74
ERG|ERG.1
ERG|ERG
Quality
200
250
300
350
400
450
Fusion-supporting reads
40
45
50
55

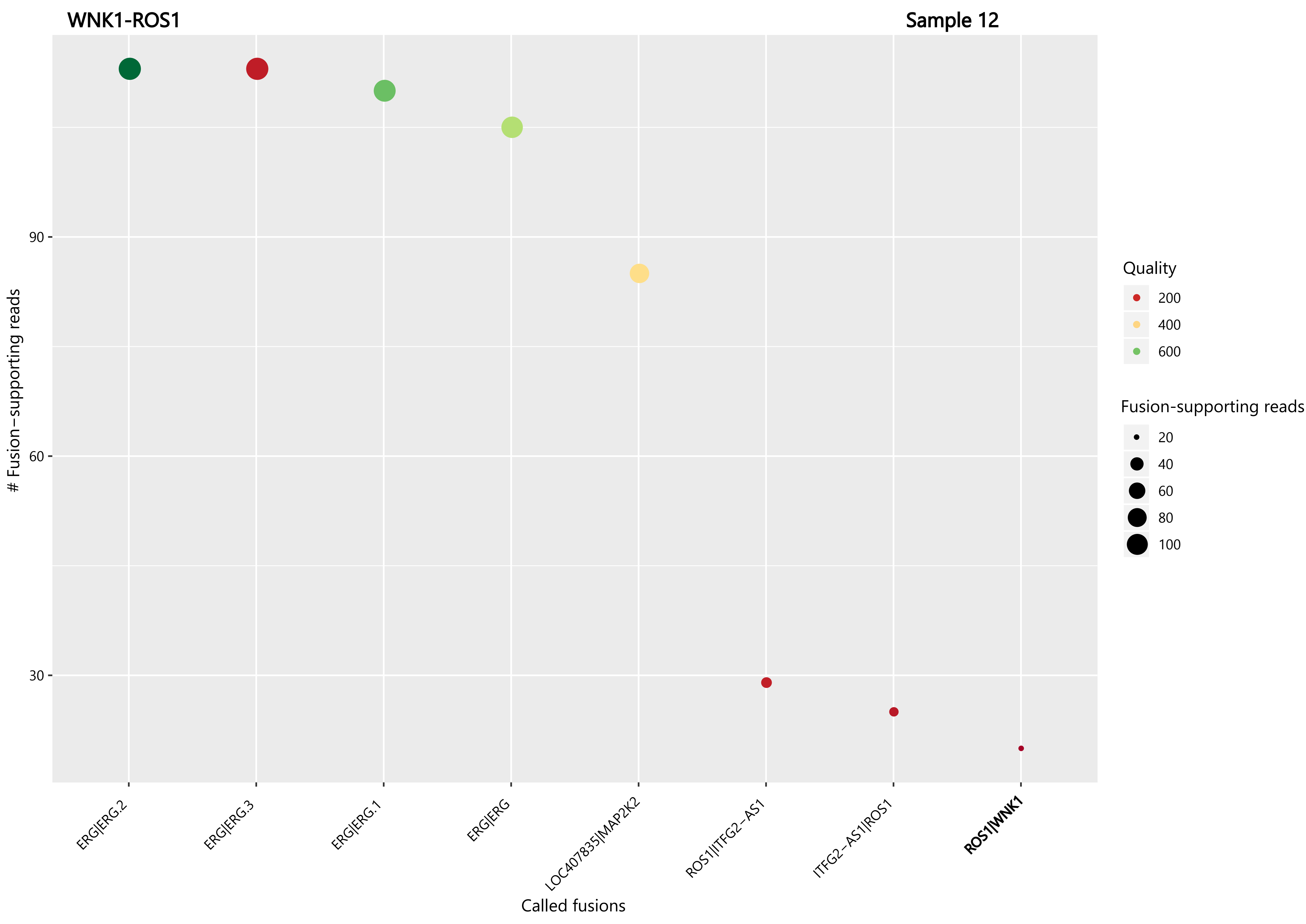

WNK1-ROS1
Sample 12
Fusion−supporting reads
90
60
30
ERG|ERG.2
ERG|ERG.3
ERG|ERG.1
ERG|ERG
LOC407835|MAP2K2
ROS1||ITFG2−AS1
ITFG2−AS1|ROS1
ROS1|WNK1
Called fusions
Quality
200
400
600
Fusion-supporting reads
20
40
60
80
100

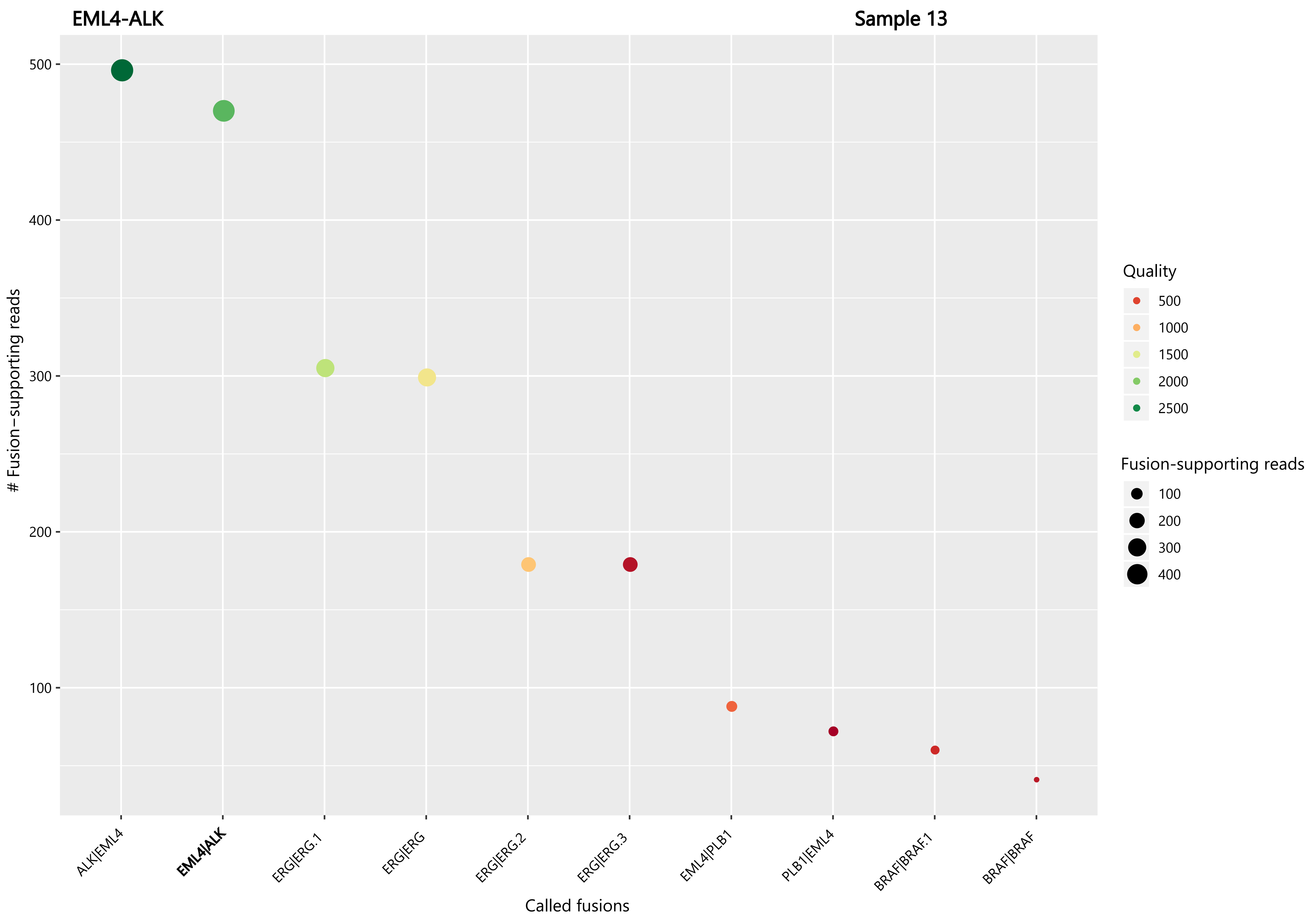

EML4-ALK
Sample 13
Fusion−supporting reads
500
400
300
200
100
Called fusions
ALK|EML4
EML4|ALK
ERG|ERG.1
ERG|ERG
ERG|ERG.2
ERG|ERG.3
EML4|PLB1
PLB1|EML4
BRAF|BRAF.1
BRAF|BRAF
Quality
500
1000
1500
2000
2500
Fusion-supporting reads
100
200
300
400

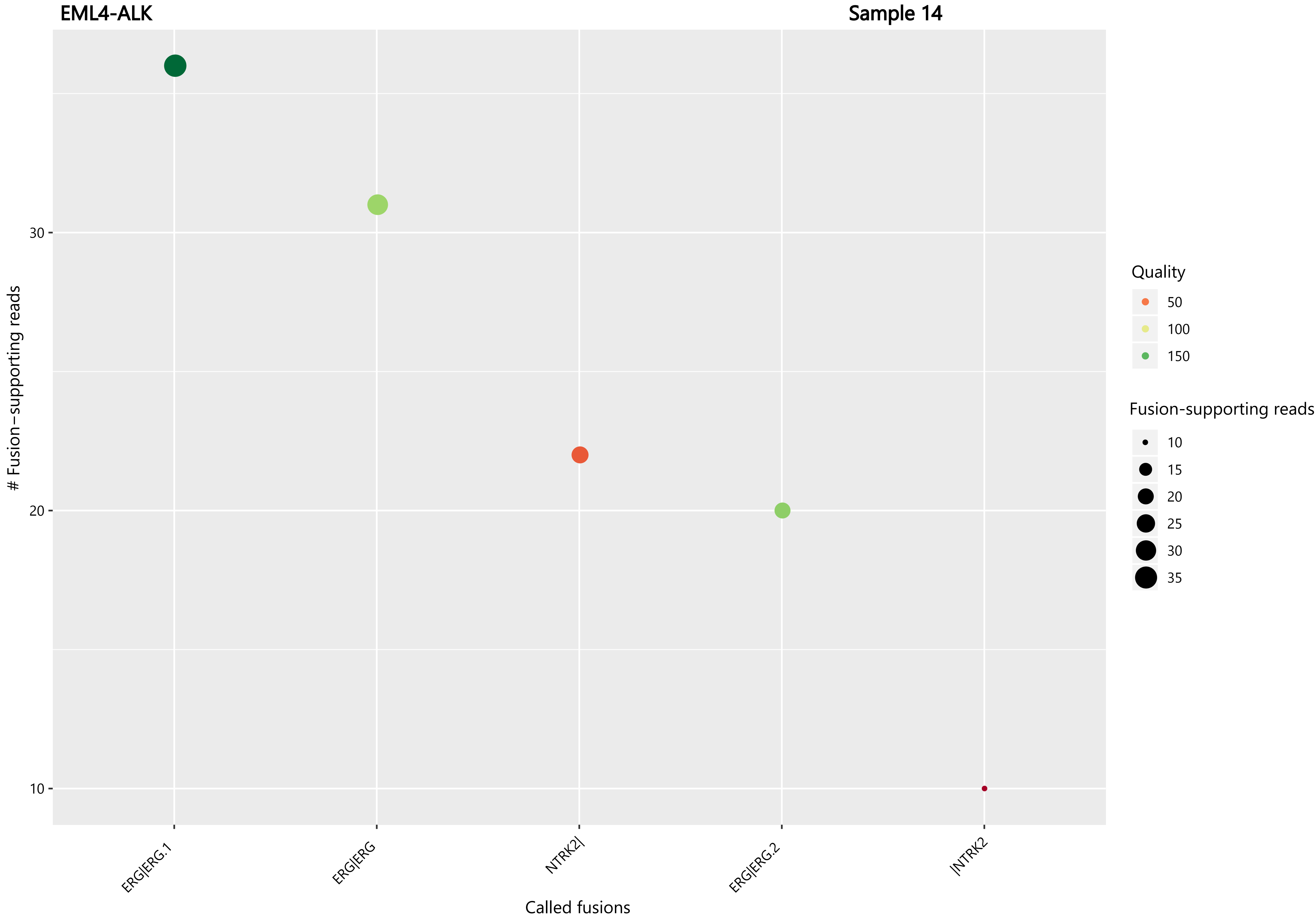

EML4-ALK
Sample 14
Fusion−supporting reads
30
20
10
ERG|ERG.1
ERG|ERG
NTRK2|
ERG|ERG.2
|NTRK2
Called fusions
Quality
50
100
150
Fusion-supporting reads
10
15
20
25
30
35

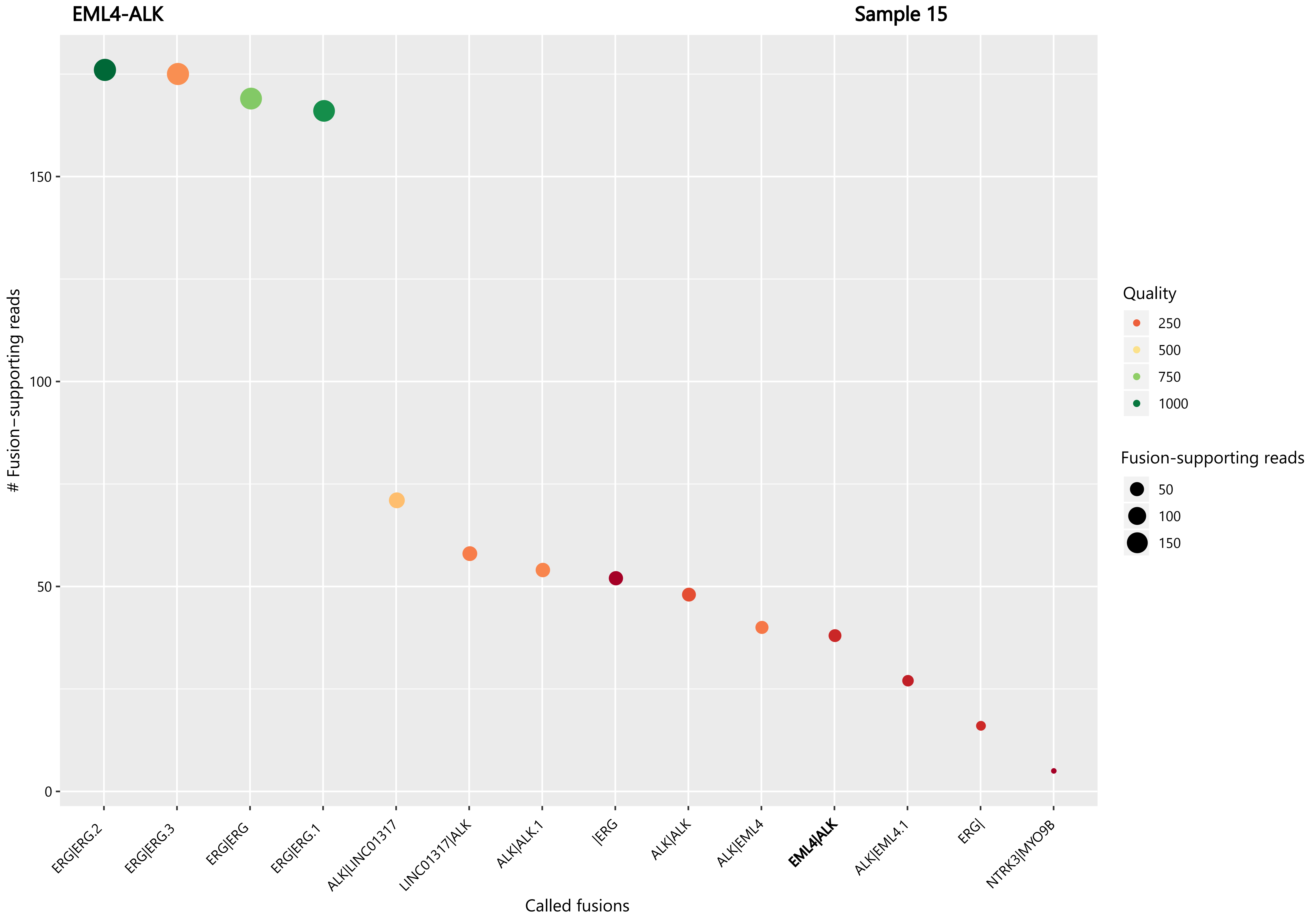
EML4-ALK
Sample 15
Fusion−supporting reads
150
100
50
0
Called fusions
ERG|ERG.2
ERG|ERG.3
ERG|ERG
ERG|ERG.1
ALK|LINC01317
LINC01317|ALK
ALK|ALK.1
|ERG
ALK|ALK
ALK|EML4
EML4|ALK
ALK|EML4.1
ERG|
NTRK3|MYO9B
Quality
250
500
750
1000
Fusion-supporting reads
50
100
150

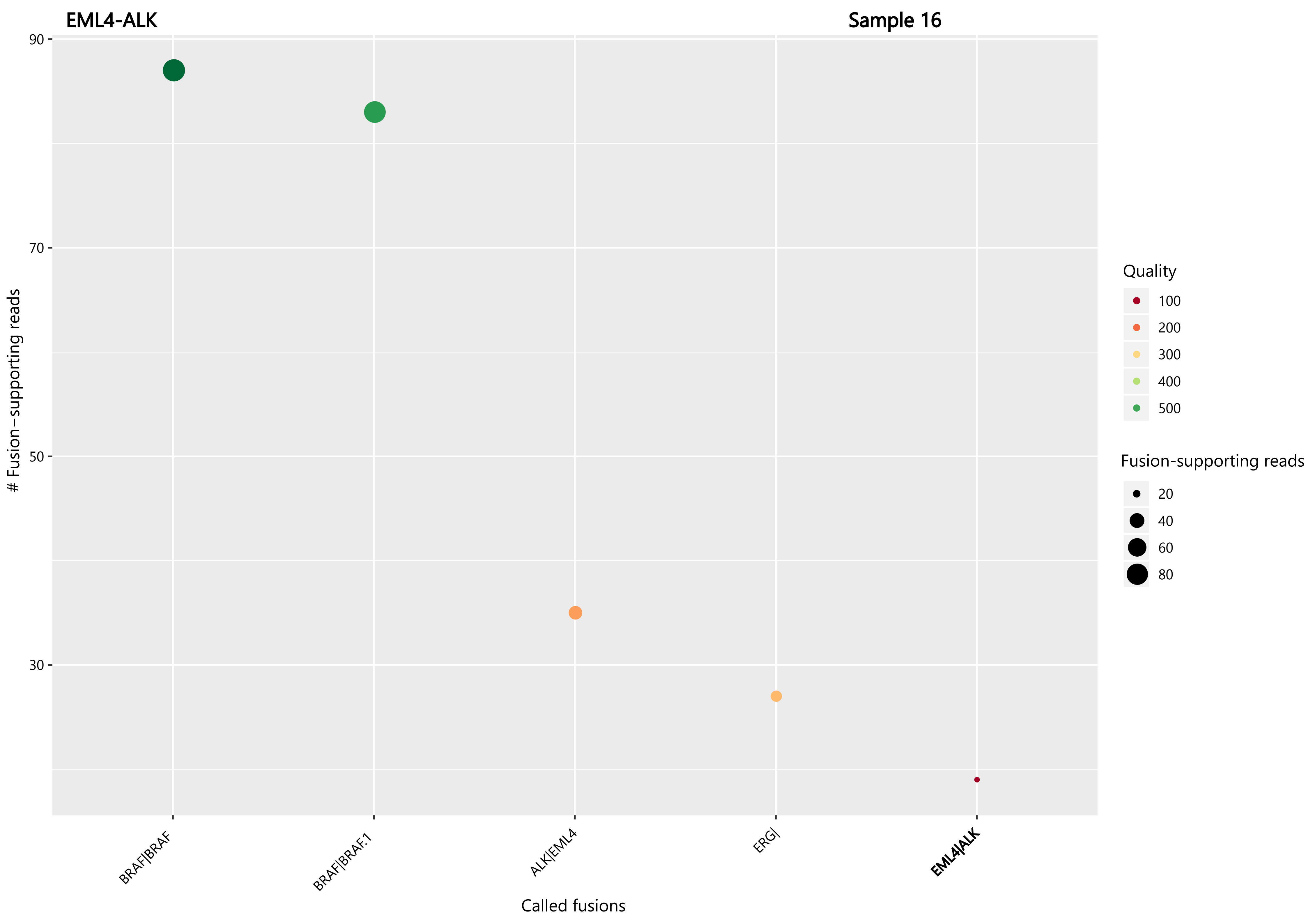

EML4-ALK
Sample 16
90
70
50
30
Fusion−supporting reads
BRAF|BRAF
BRAF|BRAF.1
ALK|EML4
ERG|
EML4|ALK
Called fusions
Quality
100
200
300
400
500
Fusion-supporting reads
20
40
60
80

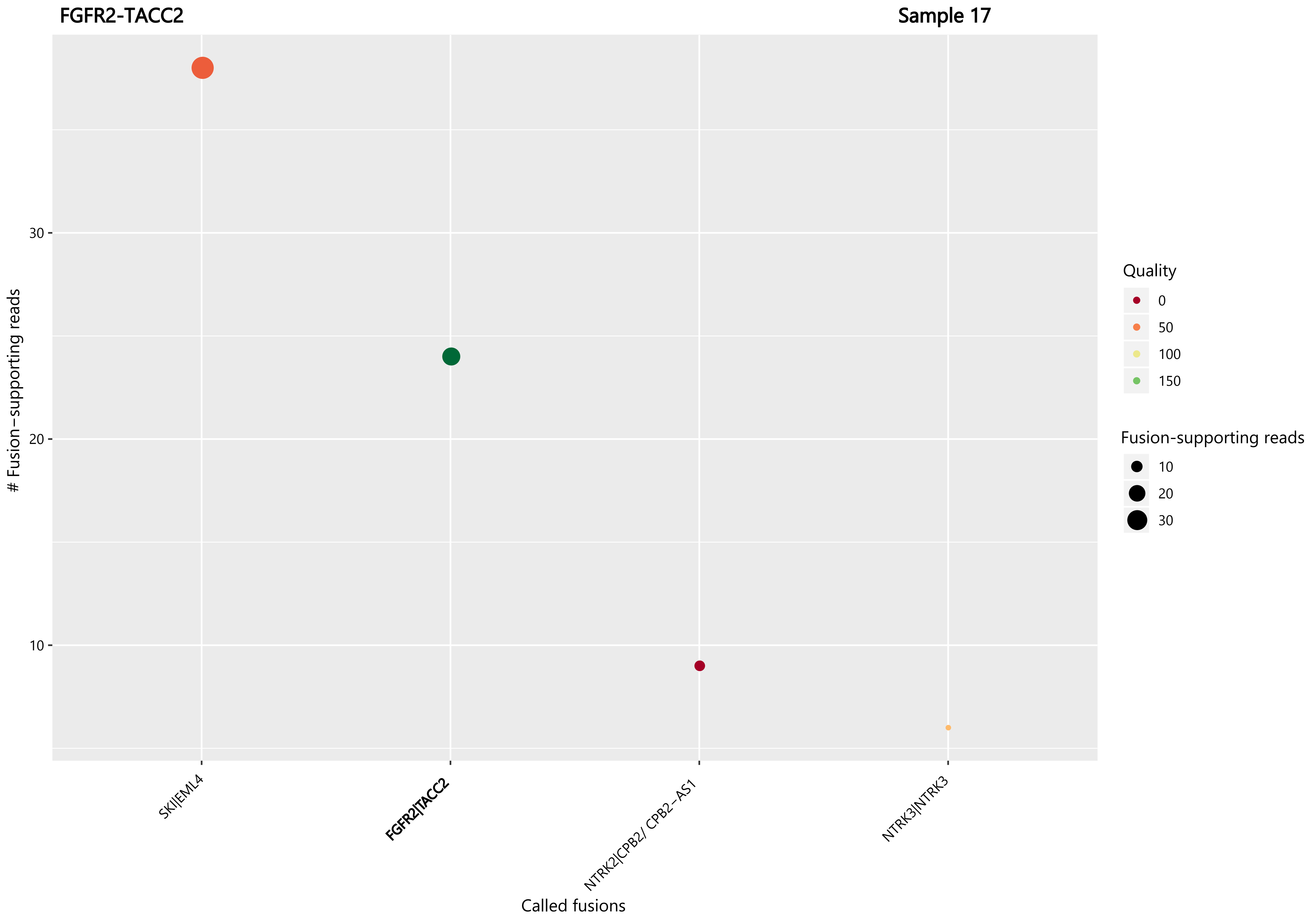

FGFR2-TACC2
Sample 17
Fusion−supporting reads
30
20
10
SKI|EML4
FGFR2|TACC2
NTRK2|CPB2/ CPB2−AS1
NTRK3|NTRK3
Called fusions
Quality
0
50
100
150
Fusion-supporting reads
10
20
30

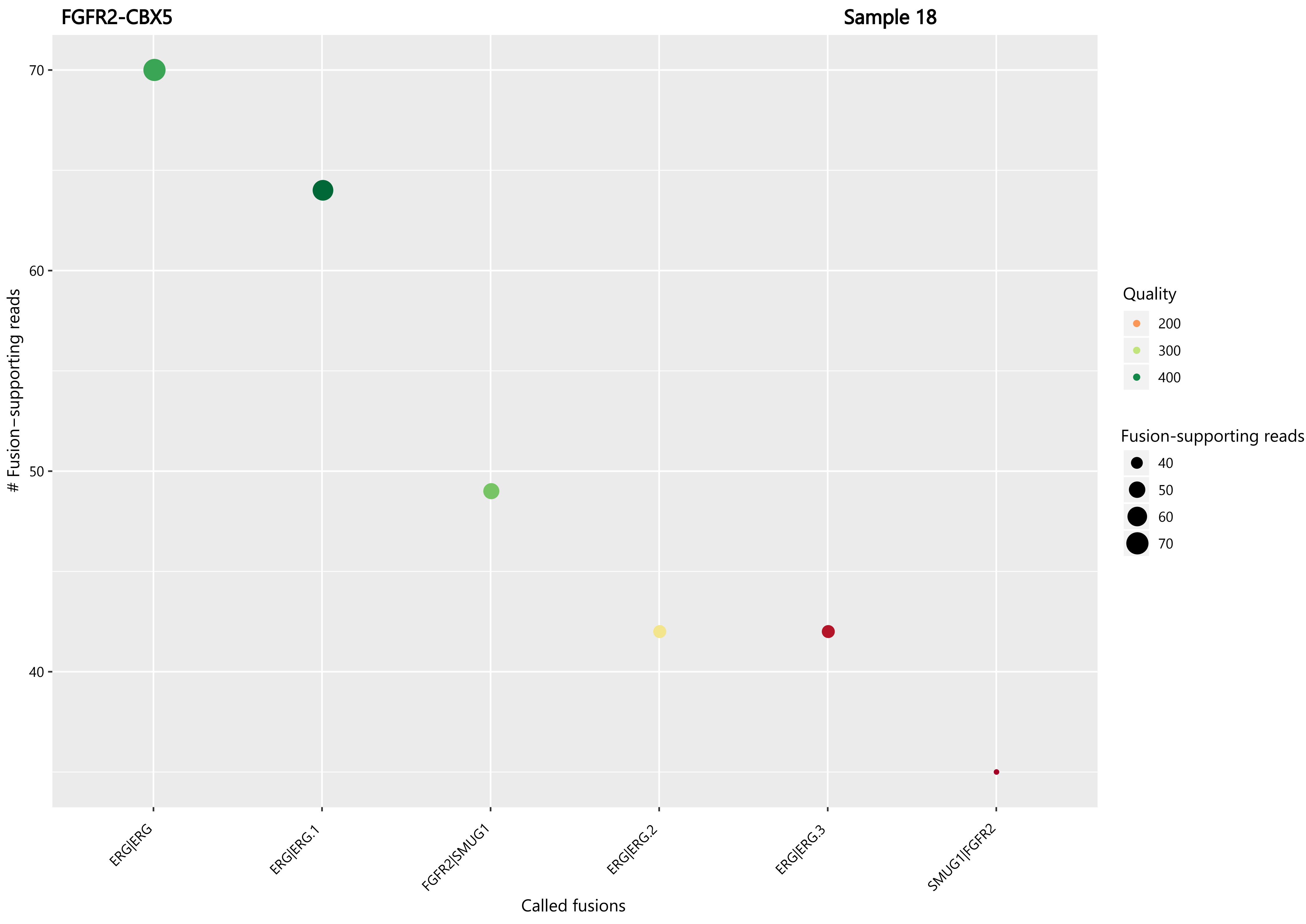
FGFR2-CBX5
Sample 18
Fusion−supporting reads
70
60
50
40
Quality
200
300
400
Fusion-supporting reads
40
50
60
70
ERG|ERG
ERG|ERG.1
FGFR2|SMUG1
ERG|ERG.2
ERG|ERG.3
SMUG1|FGFR2
Called fusions
