# Supplementary figures and images for: Detection of gene fusions using targeted next-generation sequencing: a comparative evaluation
Source: BMC Med Genomics. 2021 Feb 27;14:62. doi: 10.1186/s12920-021-00909-y (PMC7912891; doi:10.1186/s12920-021-00909-y)

A

SureSelect XT HS Custom Panel - v4.0.1.46

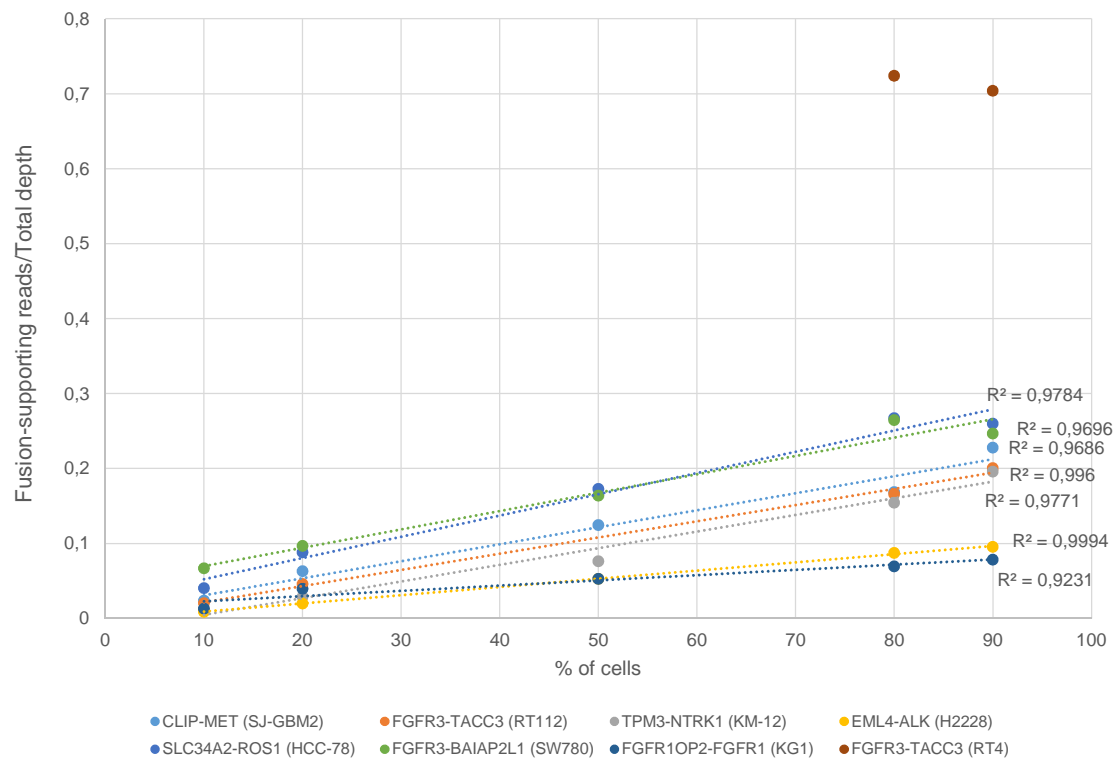

B

SureSelect XT HS Custom Panel - v4.1.1.5

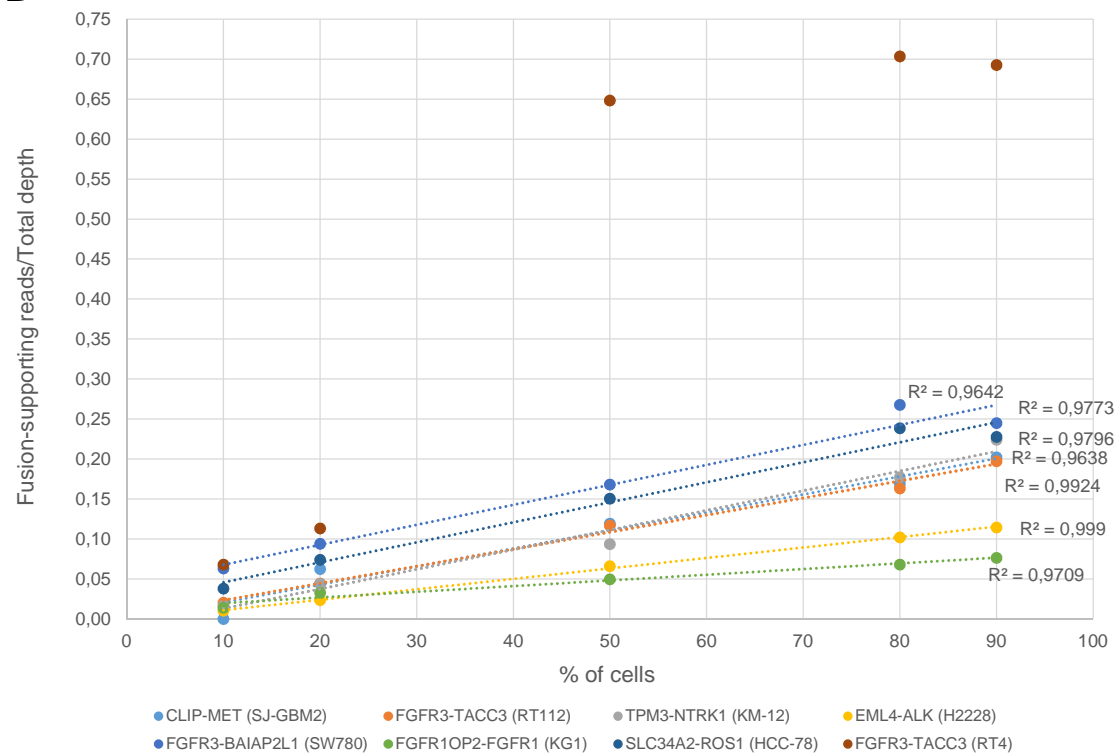

Supplement: Supplementary file 17 — Additional file 17: Fig. S1. Comparison of cell line dilutions and the number of fusion-supporting reads divided by the total read depth for the SureSelect XT HS Custom Panel (Agilent) v4.0.1.46 (A) and v4.1.1.5 (B). Depicted are the gene fusion-supporting reads divided by the total read depth of the 10%, 20%, 50%, 80% and 90% tumor cell dilutions of the eight cell lines. For each cell line, except the RT4 cells, a regression line and the coefficient of determination (R2) is shown. In the RT4 cells in v4.0.1.46 the fusion was only called in the 80% and 90% dilution and in v4.1.1.5 the fusion was only called in one direction by the TACC3 gene and not the FGFR3 gene in the 50%, 80% and 90% dilution. Thus, the values are not corresponding to the other values as they are much higher. [file 12920_2021_909_MOESM17_ESM.pdf]
