# Supplementary material for: Detection of gene fusions using targeted next-generation sequencing: a comparative evaluation
Source: BMC Med Genomics. 2021 Feb 27;14:62. doi: 10.1186/s12920-021-00909-y (PMC7912891; doi:10.1186/s12920-021-00909-y)
Supplement: Supplementary file 18 — Additional file 18: Fig. S2. Results of all five assays and their respective data analysis tools for the FFPE samples. Shown are the number of true positive fusions detected, the number of fusion-supporting reads for this fusion, as well as the number of false positives and missed fusions identified per analysis and per FFPE sample. [file 12920_2021_909_MOESM18_ESM.pdf]

	Sample 1 KIAA1549-BRAF	Sample 2 LMNA-NTRK1	Sample 3 ETV6-NTRK3	Sample 4 HLA-DRB1-MET	Sample 5 KDELR2-RET	Sample 6 NCOA4-RET	Sample 7 KIF5B-RET	Sample 8 KIF5B-RET	Sample 9 CD74-ROS1	Sample 10 CD74-ROS1	Sample 11 CD74-ROS1	Sample 12 WNK1-ROS1	Sample 13 EML4-ALK	Sample 14 EML4-ALK	Sample 15 EML4-ALK	Sample 16 EML4-ALK	Sample 17 FGFR2-TACC2	Sample 18 FGFR2-CBX5
**Archer FusionPlex Lung Panel (Archer DX) v5.0.4)**																		
**True Positives**	1	1	1	1	1	1	1	0	1	1	1	1	1	1	1	1	1	1
**Fusion-supporting reads**	463	421	9871	127	22	473	10	0	20	1056	4000	106	7036	531	181	243	353	318
**False Positives**	0	0	0	0	1	1	0	1	0	0	3	1	3	0	1	1	0	1
**Missed Fusions**	0	0	0	0	0	0	0	1	0	0	0	0	0	0	0	0	0	0
**Archer FusionPlex Lung Panel (Archer DX) v5.1.3)**																		
**True Positives**	1	1	1	1	1	1	0	0	1	1	1	1	1	1	1	1	1	1
**Fusion-supporting reads**	467	2461	10792	123	27	495	0	0	20	1691	209	564	8000	680	79	356	345	342
**False Positives**	0	3	9	0	0	0	0	0	1	3	4	0	1	1	1	1	0	1
**Missed Fusions**	0	0	0	0	0	0	1	1	0	0	0	0	0	0	0	0	0	0
**QIAseq Targeted RNAscan Custom Panel (Qiagen)**																		
**True Positives**	1	1	1	1	1	1	1	1	1	1	1	1	1	1	1	1	1	1
**Fusion-supporting reads**	169	451	4098	375	26	132	325	35	238	534	314	77	773	109	31	44	98	144
**False Positives**	9	6	12	7	14	5	3	0	8	6	7	5	4	4	1	4	5	7
**Missed Fusions**	0	0	0	0	0	0	0	0	0	0	0	0	0	0	0	0	0	0
**Oncomine Focus Assay (Thermo Fisher Scientific)**																		
**True Positives**	0	0	1	0	0	1	1	1	1	1	1	0	1	1	1	1	0	0
**Fusion-supporting reads**	0	0	63678	0	0	16464	5266	7360	35238	19617	24108	0	180437	8297	827	12	0	0
**False Positives**	0	0	1	0	0	0	1	1	0	1	1	0	0	0	0	279	0	0
**Missed Fusions**	1	1	0	1	1	0	0	0	0	0	0	1	0	1	0	0	1	1
**TruSight 170 Assay (Illumina)**																		
**True Positives**	**1**	1	1	1	1	1	1	1	1	1	1	1	1	1	1	1	1	1
**Fusion-supporting reads**	**755**	23642	19328	963	756	2785	7208	3623	5767	15047	6892	4685	15782	1588	962	767	2581	7039
**False Positives**	**0**	0	0	1	0	0	0	0	4	2	3	0	2	0	0	1	0	0
**Missed Fusions**	**0**	0	0	0	0	0	0	0	0	0	0	0	0	0	0	0	0	0
**SureSelect XT HS Custom Panel (Agilent) v4.0.1.46**																		
**True Positives**	1	1	1	0	0	0	1	1	1	1	1	1	1	1	1	1	0	0
**Fusion-supporting reads**	23	70	184	0	0	0	14	31	37	29	58	16	474	5	38	19	0	0
**False Positives**	20	37	29	1438	1374	18	35	45	27	31	19	32	30	9	46	12	12	12
**Missed Fusions**	0	0	0	1	1	1	0	0	0	0	0	0	0	0	0	0	1	1
**SureSelect XT HS Custom Panel (Agilent) v4.1.1.5**																		
**True Positives**	0	1	1	0	0	1	1	1	1	1	1	1	1	0	1	1	1	0
**Fusion-supporting reads**	0	70	240	0	0	12	14	30	35	28	58	20	470	0	38	19	24	0
**False Positives**	5	15	10	2	6	6	8	15	9	17	5	7	9	5	13	4	3	6
**Missed Fusions**	1	0	0	1	1	0	0	0	0	0	0	0	0	1	0	0	0	1
